# Appendage- and Scaffold-Diverse Electrophilic and Photoreactive Probes for Integrated Phenotypic Screening–Target Identification Campaigns via a Minimalist Bifunctional Isocyanide

**DOI:** 10.1021/acsomega.4c06879

**Published:** 2024-10-02

**Authors:** Paul A. Jackson, Eleni Kisty, Vandan Pradhan, Christopher Swank, Luke Bohrer, Tammy L. Nolan, Eranthie Weerapana, David J. Lapinsky

**Affiliations:** † Graduate School of Pharmaceutical Sciences, Duquesne University, 600 Forbes Avenue, Pittsburgh, Pennsylvania 15282, United States; ‡ Department of Chemistry, Boston College, Merkert Chemistry Center, 2609 Beacon Street, Chestnut Hill, Massachusetts 02464, United States

## Abstract

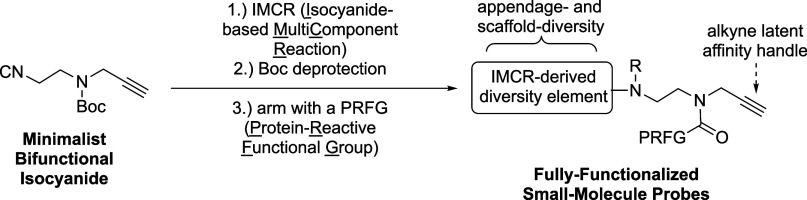

One of the grand challenges in chemical biology is identifying a small-molecule modulator for all proteins within a proteome. To expand the variety and number of ligandable proteins for drug discovery, the objective of this study was to synthesize and evaluate the protein target profiles of electrophilic and photoreactive fully functionalized small-molecule probes (FFSMPs) featuring increased scaffold-, appendage-, and protein-reactive functional group (PRFG) diversity. FFSMPs contain: (1) a protein-binding motif, (2) an electrophilic or photoreactive PRFG for target protein capture, and (3) a terminal alkyne for click chemistry-based proteomic applications. These compounds can be directly applied in phenotypic screening programs to identify ligand–protein pairs in cells unbiasedly. Herein, we highlight 17 examples from 34 structurally diverse FFSMPs featuring five electrophiles, three photoreactive groups, and 15 chemical scaffolds. Essential to the synthesis of the FFSMPs was a new minimalist bifunctional isocyanide in an “isocyanide-based multicomponent reaction–Boc deprotection–arming” synthetic sequence. To the best of our knowledge, this is the first report concerning the preparation of appendage- and scaffold-diverse FFSMPs for integrated phenotypic screening-target identification campaigns with the ability to examine either electrophilic or photoreactive PRFGs. In contrast, the status quo for such studies has been appendage-diverse FFSMPs comprised of a single chemical scaffold and a single PRFG, which limits efficient target protein capture and/or chemical space sampling significantly in the quest for discovering new drug targets and/or compounds with novel mechanisms of action. Phenotypic screening of the electrophilic members of our library identified several FFSMPs with potent antiproliferative activity against MCF10CA1a breast cancer cells. One of these FFSMPs (Compound **4a**) covalently targeted and potently inhibited protein disulfide isomerase A1 (PDIA1). This study supports the continued use of minimalist bifunctional isocyanides as valuable building blocks for preparing structurally diverse FFSMPs for integrated phenotypic screening–target identification campaigns.

## Introduction

Despite sequencing the human genome some 20 years ago,^1^ many genes linked to specific disease traits or phenotypes remain uncharacterized and are grossly understudied. Proteins coded by these genes have been designated as “dark proteome” members.^2^ According to one estimate, small-molecule chemical probes are currently unavailable for ∼96% of the human proteome.^3^ This scarcity of chemical probes as tool compounds to selectively modulate protein function causes an inability to study large areas of biological space, validate novel drug targets, enable future research, and discover better therapeutics.^4,5^

**Scheme 1:**
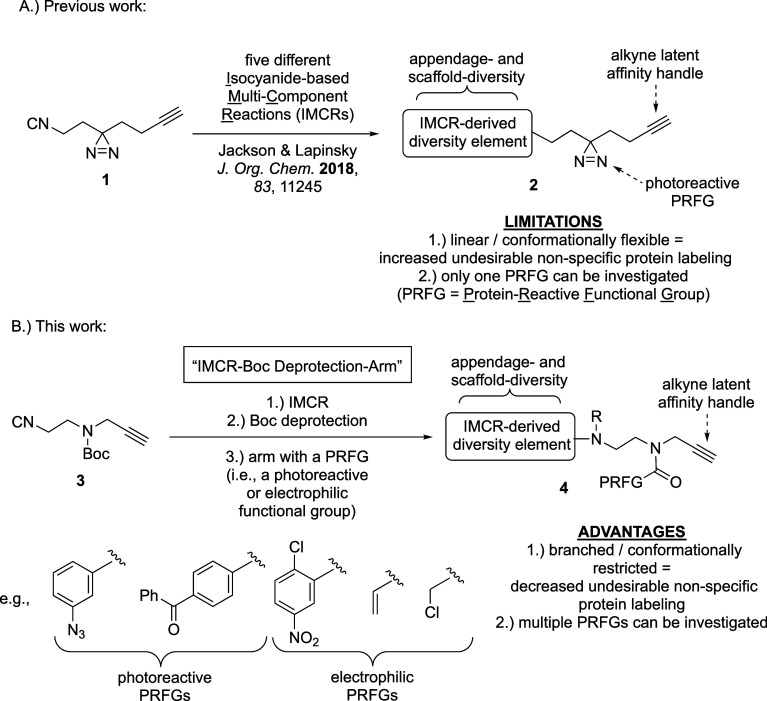
Our Synthetic Approaches Toward Structurally-Diverse Fully Functionalized Small-Molecule Probes (FFSMPs, **2** and **4**) for Integrated Phenotypic Screening–Target Identification Studies via Minimalist Bifunctional Isocyanides **1** and **3** (A) Previously reported minimalist bifunctional isocyanide **1**, which contains a photoreactive aliphatic diazirine for target protein capture via photoaffinity labeling and a terminal alkyne for click chemistry-based proteomics, was employed in five different isocyanide-based multi-component reactions (IMCRs) to produce appendage- and scaffold-diverse photoreactive FFSMPs **2**. (B) Application of minimalist bifunctional isocyanide **3** in a “IMCR–Boc deprotection–arming” synthetic sequence to create appendage- and scaffold-diverse photoreactive or electrophilic FFSMPs **4** containing a protein-reactive functional group (PRFG) of choice for target protein capture via photoaffinity or affinity labeling, respectively.

**Scheme 2:**
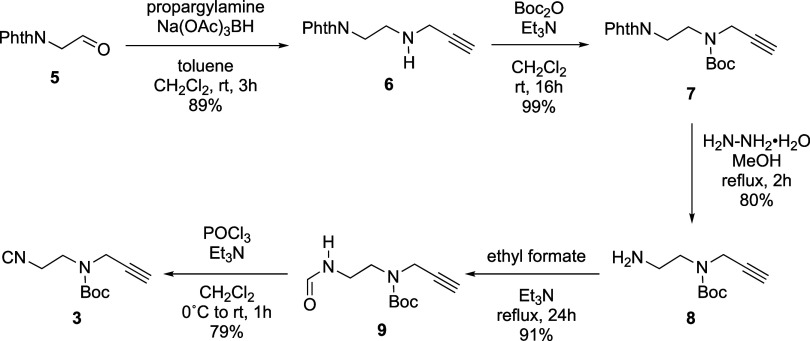
Synthesis of Minimalist Bifunctional Isocyanide **3** See Supporting Information for experimental details.

One approach to expand the landscape of drug targets accessible to small-molecules is using fully functionalized small-molecule probes (FFSMPs) in streamlined phenotypic screening–target identification campaigns.^6−17^ FFSMPs (Scheme 1; **2** and **4**) contain: (1) a protein-binding motif containing one or more structural-diversity elements to interact with unique subsets of protein targets, (2) an electrophilic^18^ or photoreactive^19^ functional group as a protein-reactive functional group (PRFG) to facilitate irreversible covalent bond formation between the probe and its interacting proteins via affinity or photoaffinity labeling, respectively, and (3) a terminal alkyne click chemistry handle for reporter tag conjugation to visualize, enrich, and identify probe-linked proteins.^20^ The direct integration of FFSMPs in phenotypic screening campaigns has enabled the discovery of bioactive chemical probes and their corresponding protein targets, and structurally diverse FFSMPs can capture proteins that currently lack small-molecule ligands.^21,22^ Additionally, FFSMPs can illuminate a diverse range of nontraditional or traditional druggable targets in cells while facilitating the discovery of compounds with unique mechanisms of action.^6−17^ Finally, FFSMPs can be used to map ligand-binding sites within proteins, validate drug–target engagement, and serve as imaging probes.^21,22^

To date and with minimal exceptions,^11,12,14−17,23^ the predominant type of chemical diversity/structural complexity pursued in generating FFSMP libraries has been appendage-diversity,^6−9,13^ wherein different peripheral structural entities are attached around a single chemical scaffold. However, such an FFSMP design strategy is not optimal for discovering new protein drug targets because the sampling of chemical space is relatively small and biased.^24,25^ It is well-established that the overall 3-D molecular shape, and thus functional diversity of a screening library, is highly contingent on chemical scaffold-diversity instead of appendage-diversity.^26−28^

With the current lack of scaffold-diverse FFSMPs in mind, we recently reported minimalist bifunctional isocyanide **1**, which was directly employed in five different isocyanide-based multicomponent reactions (IMCRs) to produce ten FFSMPs (**2**) containing eight different chemical scaffolds (Scheme 1A).^23^ Specifically, building block **1** was derived as an isocyanide variant of a well-known set of minimalist linkers developed by Yao and group.^29^ This set of minimalist linkers contains a photoreactive aliphatic diazirine functional group for target protein capture, a terminal alkyne click chemistry handle, and either a carboxylic acid, 1° aliphatic iodide, or 1° aliphatic amine for attachment to a small-molecule of interest.^29^ However, instead of simply attaching the minimalist linkers via heteroatom-alkylation or -acylation to produce FFSMPs, we utilized the isocyanide functional group within **1** in several different IMCRs involving a wide variety of commercially available carboxylic acids, 1° amines, aldehydes, and ketones.^23^ The resulting appendage- and scaffold-diverse FFSMPs (**2**) also included rationally incorporating different privileged structures^30,31^ to direct the FFSMP to sample biologically relevant chemical space.^32^ Additionally, Topliss moieties,^33,34^ which are aromatic rings or aliphatic side-chain appendages traditionally replaced as part of a medicinal chemistry optimization campaign to improve biological or physicochemical properties, were also rationally incorporated into FFSMPs **2**.

Finally, previously described FFSMPs libraries, with minimal exceptions,^7,10,13^ have typically investigated only a single electrophilic or photoreactive functional group as a protein-reactive functional group (PRFG),^6,8,9,11,12,14−17,23^ which can limit the potential discovery of new drug targets due to suboptimal target protein capture efficiency and/or a relatively small sampling of chemical space. As a result, scaffold- and appendage-diverse FFSMPs **2**, derived from applying aliphatic diazirine-based bifunctional isocyanide **1** in several IMCRs, readily suffer from these disadvantages. Additionally, bifunctional isocyanide **1** is a linear and conformationally flexible building block that leads to the production of more linear and conformationally flexible photoreactive FFSMPs **2** (Scheme 1A), which are known to have a greater tendency to engage in undesirable nonspecific protein labeling compared to more branched and conformationally restricted FFSMPs.^35^ Therefore, to address these prominent disadvantages, we hypothesized: (1) an improved minimalist and branched bifunctional isocyanide, **3**, featuring a shorter chain length and replacement of the aliphatic diazirine in **1** with a Boc carbamate as a disguised 2° aliphatic amine synthetic branching point and (2) the application of **3** in a general three-step “IMCR–Boc deprotection–arming” synthetic sequence to generate more branched and conformationally restricted appendage- and scaffold-diverse FFSMPs **4** (Scheme 1B) featuring late-stage diversification of an unmasked 2° aliphatic amine with an electrophile or photoreactive PRFG of choice via N-acylation.

Herein, we present 17 examples out of 34 structurally diverse FFSMPs with 15 chemical scaffolds, five electrophiles (S_N_2-, S_N_Ar-, Michael/conjugate addition-, SuFEx-, and strain-release-based reactivity), and three photoreactive groups (aryl azides, diazirines, and benzophenones). The chemical structures of all FFSMPs produced during this work (i.e., derived from minimalist bifunctional isocyanide **3**) can be found within the Supporting Information and are color-coded to indicate: (1) a terminal alkyne for click chemistry-based proteomics,^20^ (2) an electrophilic PRFG for protein capture via affinity labeling,^18^ (3) a photoreactive PRFG for protein capture via photoaffinity labeling,^19^ (4) a privileged structure to sample biologically relevant chemical space,^32^ and (5) a moiety for Topliss-based medicinal chemistry optimization.^33,34^

## Results

### Synthesis of *tert*-Butyl (2-Isocyanoethyl)(prop-2-yn-1-yl)carbamate as a New Minimalist Bifunctional Isocyanide for Preparing Structurally Diverse FFSMPs via IMCRs

To experimentally test our hypothesized “IMCR–Boc deprotection–arming” synthetic strategy to access structurally diverse FFSMPs **4** (Scheme 1B), we first had to produce bifunctional isocyanide **3** (i.e., (2-isocyanoethyl)(prop-2-yn-1-yl)carbamate) on an appreciable scale. As depicted in Scheme 2, bifunctional isocyanide **3** was produced in gram quantities and 51% overall yield using five synthetic steps starting from known aldehyde **5**.^36^ Aldehyde **5** was first subjected to reductive amination with propargylamine and then Boc-protected to provide carbamate **7** in 88% yield over two steps. Next, the phthalimide protecting group was removed to give 1° aliphatic amine **8**, which was then converted to target isocyanide **3** via a standard sequence of formamide formation and dehydration.^37^

### Preparation of Structurally Diverse Electrophilic and Photoreactive FFSMPs by Employing (2-Isocyanoethyl)(prop-2-yn-1-yl)carbamate in Different IMCRs

**Scheme 3:**
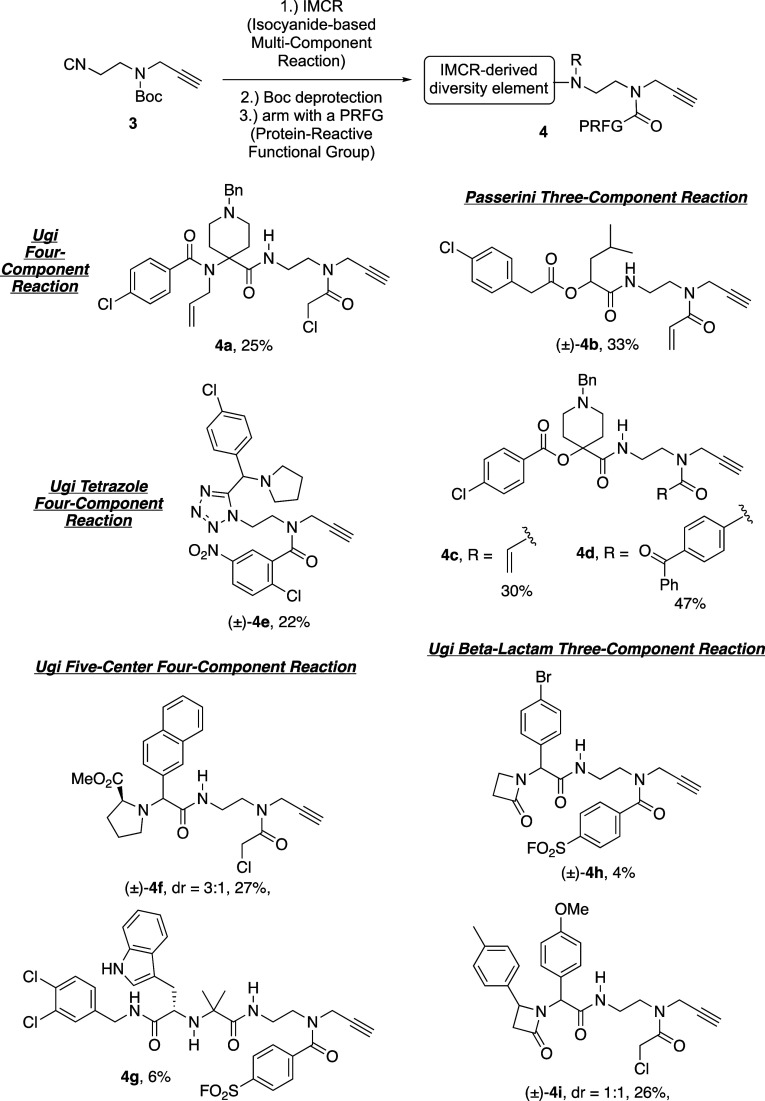
Select Examples of Appendage- and Scaffold-Diverse Fully Functionalized Small-Molecule Probes (FFSMPs, **4**) for Integrated Phenotypic Screening–Target Identification Campaigns Synthesized using Minimalist Bifunctional Isocyanide **3** in a Three-Step Reaction Sequence Involving an Isocyanide-based Multi-Component Reaction (IMCR), Boc Deprotection, and Arming the Requisite 2° Aliphatic Amine with a Protein-Reactive Functional Group (PRFG) of Choice Yields reported are those over the three-step “IMCR–Boc deprotection–arming” synthetic sequence. See Supporting Information for synthetic schemes, experimental details, and 14 other examples of FFSMPs.

With bifunctional isocyanide **3** in hand and analogous to our previous report concerning bifunctional isocyanide **1**,^23^ we utilized building block **3** in several different IMCRs, followed by Boc deprotection and N-acylation with an electrophilic or photoreactive protein-reactive functional group (PRFG) building block of choice. This effort provided 23 different FFSMPs (**4**) (see Supporting Information), of which nine examples are depicted in Scheme 3 and will now be discussed briefly.

It is well-known that the classical Ugi four-component reaction (U-4CR) can provide access to many secondary chemical scaffolds with extensive application to drug discovery.^38,39^ For example, FFSMP **4a** was designed to feature aromatic Topliss moieties at the carboxylic acid and oxo-component positions within the U-4CR adduct. Additionally, 1-benzyl-4-piperidone was chosen as the oxo-component for this U-4CR, as benzyl piperidines can serve a dual role as a privileged structure and an area for aromatic ring Topliss optimization.^31^

We next hypothesized that isocyanide **3** could be successfully employed in a Passerini three-component reaction (*p*-3CR). In contrast to the Ugi-4CR, which produces alpha-aminoacyl amide derivatives (e.g., **4a**), the *p*-3CR is an alternative and well-established IMCR that produces alpha-hydroxy carboxamides with known applications in natural product synthesis.^40^ Three examples of electrophilic (i.e., acrylamides (±)-**4b** and **4c**) and photoreactive (i.e., benzophenone **4d**) FFSMPs generated using bifunctional isocyanide **3** in a *p*-3CR–Boc deprotection–arming sequence are depicted in Scheme 3. Of note, to demonstrate the late-stage incorporation of either an electrophilic or photoreactive PRFG on an identical chemical scaffold, electrophilic FFSMP **4c** and photoreactive FFSMP **4d** were generated by using the acid chloride derivatives of acrylic and 4-benzoylbenzoic acid, respectively, during the final arming step after Boc deprotection.

In addition to U-4CR- and *p*-3CR-derived FFSMPs, we also employed bifunctional isocyanide **3** in three other well-described IMCRs: the Ugi tetrazole four-component reaction (UT-4CR) to give FFSMP (±)-**4e**, the Ugi five-center-four-component reaction (U-5C-4CR) to give FFSMPs (±)-**4f** and **4g**, and the Ugi beta-lactam three-component reaction (UBL-3CR) to give FFSMPs (±)-**4h** and (±)-**4i**. As select examples, chloronitro-containing S_N_Ar-based FFSMP (±)-**4e** originated from a UT-4CR involving TMS-N_3_, isocyanide **3**, pyrrolidine as the 2° amine input, and 4-chlorobenzaldehyde as an aromatic Topliss moiety. Interestingly, the U-5C-4CR can serve as the basis for generating either iminodicarboxamide- or methyl ester-containing FFSMPs (e.g., **4g** and (±)-**4f**, respectively) by using MeOH as the reaction solvent with or without the inclusion of a 1° aliphatic amine (e.g., 3,4-dichlorobenzylamine toward FFSMP **4g**) as a fifth component, respectively.^41^ Finally, the UBL-3CR was used to produce FFSMPs (±)-**4h** and (±)-**4i** that could uniquely form two covalent bonds to target proteins, thus aiding in ligand-binding site mapping due to the presence of a PRFG of choice and a privileged electrophilic beta-lactam ring.^42^

### Preparation of Structurally Diverse Electrophilic and Photoreactive FFSMPs by Employing (2-Isocyanoethyl)(prop-2-yn-1-yl)carbamate in a “Sequential/Union of MCRs” Strategy Involving the U-4CR

**Scheme 4:**
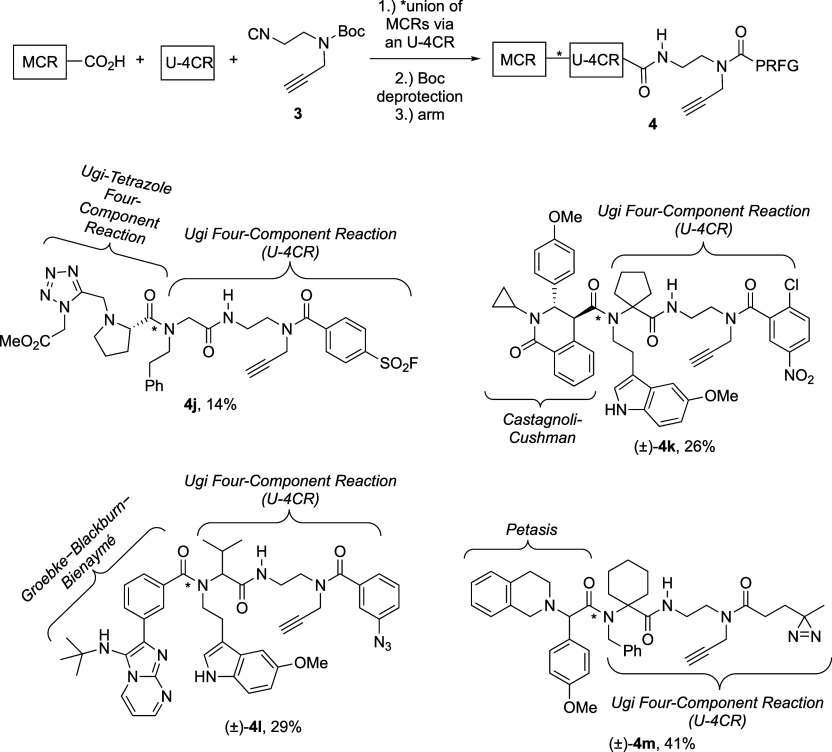
Modular Synthesis of Structurally Diverse Electrophilic (SuFEx-based **4j** and S_N_Ar-based (±)-**4k**) and Photoreactive (Aryl Azide (±)-**4l** and Aliphatic Diazirine (±)-**4m**) Fully Functionalized Small-Molecule Probes (FFSMPs) by Employing Bifunctional Isocyanide **3** Sequentially/in a Union of Multicomponent Reactions (MCR) Strategy In brief, a carboxylic acid building block was first produced by an MCR. This carboxylic acid building block was then used alongside a 1° aliphatic amine, an aldehyde, or a ketone, and bifunctional isocyanide **3** in a three-step “Ugi four-component reaction (U-4CR)–Boc deprotection–arming” synthetic sequence. Reported yields of the FFSMPs are from the overall three-step “U-4CR–Boc deprotection–arming” synthetic sequence. PRFG = protein-reactive functional group. *The amide bond made during the U-4CR “union of MCRs” step. See Supporting Information for synthetic schemes and experimental details.

Next, we turned our investigation toward increasing the structural complexity of FFSMPs using synthetic strategies that can be used sequentially/in-union with the U-4CR.^43^ Specifically, the production of FFSMPs **4j**–(±)-**4m** (as depicted in Scheme 4) first involved the synthesis of a known carboxylic acid derivative using a multicomponent reaction (MCR). This carboxylic acid then served as an input alongside a 1° aliphatic amine, an aldehyde, or a ketone, and bifunctional isocyanide **3** in a U-4CR. Finally, Boc deprotection followed by N-acylation with a desired photoreactive or electrophilic PRFG provided the target FFSMPs in appreciable yields. Specifically, FFSMPs **4j**–(±)-**4m** were derived from known carboxylic acid derivatives produced by UT-4CR,^44^ Castagnoli–Cushman,^45^ Groebke–Blackburn–Bienaymé,^46^ and Petasis reactions,^47^ respectively.

### Preparation of Structurally Diverse FFSMPs by Employing (2-Isocyanoethyl)(prop-2-yn-1-yl)carbamate in a “U-4CR-intramolecular Cyclization” Strategy

**Scheme 5:**
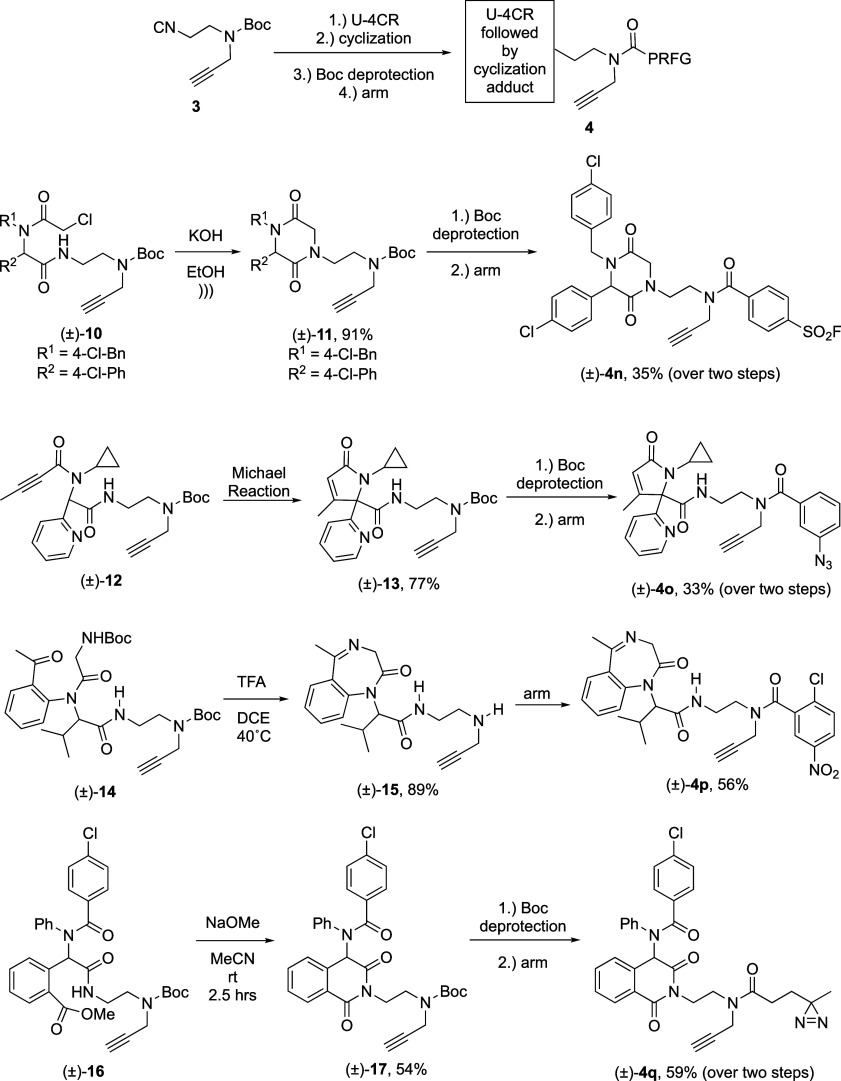
Modular Synthesis of Structurally Diverse Electrophilic (SuFEx-based (±)-**4n** and S_N_Ar-based (±)-**4p**) and Photoreactive (Aryl Azide (±)-**4o** and aliphatic Diazirine (±)-**4q**) Fully Functionalized Small-Molecule Probes (FFSMPs) by Employing bifunctional Isocyanide by **3** in a “Ugi Four-Component Reaction (U-4CR)–Intramolecular Cyclization–Boc Deprotection–Arming” Synthetic Sequence See Supporting Information for synthetic schemes and experimental details.

As a final demonstration of the synthetic value of bifunctional isocyanide **3** in producing scaffold-, appendage-, and PRFG-diverse FFSMPs, it is well-established that U-4CR products can be used to generate a wide range of novel cyclic or acyclic scaffolds via divergent secondary transformations.^48^ Specifically, Scheme 5 shows several known sequential U-4CR-intramolecular cyclization strategies,^49−53^ all of which use minimalist bifunctional isocyanide **3** to produce an initial U-4CR adduct. Interestingly, U-4CR adducts (±)-**10**, (±)-**12**, and (±)-**14** are FFSMPs themselves, given these compounds contain a terminal alkyne click chemistry handle and an electrophilic alpha-chloroacetamide, a butynamide Michael acceptor, and a photoreactive acetophenone^54^ as PRFGs, respectively. In turn, U-4CR adducts (±)-**10**, (±)-**12**, (±)-**14**, and (±)-**16** were then cyclized under acidic or basic conditions to give privileged structure 2,5-diketopiperazine^49^ (±)-**11**, alpha,beta-unsaturated lactam^50^ (±)-**13**, privileged structure 1,4-benzodiazepine^51,52^ (±)-**15**, and isoquinoline-1,3(2*H*,4*H*)-dione^53^ (±)-**17**, respectively. Per our established strategy, Boc deprotection and arming with a PRFG gave structurally diverse FFSMPs (±)-**4n** - (±)-**4q** in yields ranging from 33 to 59% over the final two steps.

### Phenotypic Screening and Chemoproteomic Target Identification Studies Involving Electrophilic Structurally Diverse FFSMPs

To evaluate the bioactivity and potential of our FFSMPs (**4**) to modify protein targets directly in living cells covalently, the electrophilic members of our FFSMP library were evaluated in a phenotypic screen for antiproliferative activity against MCF10CA1a breast cancer cells. MCF10CA1a cells are highly aggressive cancer cells derived from the normal human mammary epithelial cell line, MCF10A,^55^ via expression of constitutively active H-RAS followed by in vivo passaging.^56^

**Figure 1:**
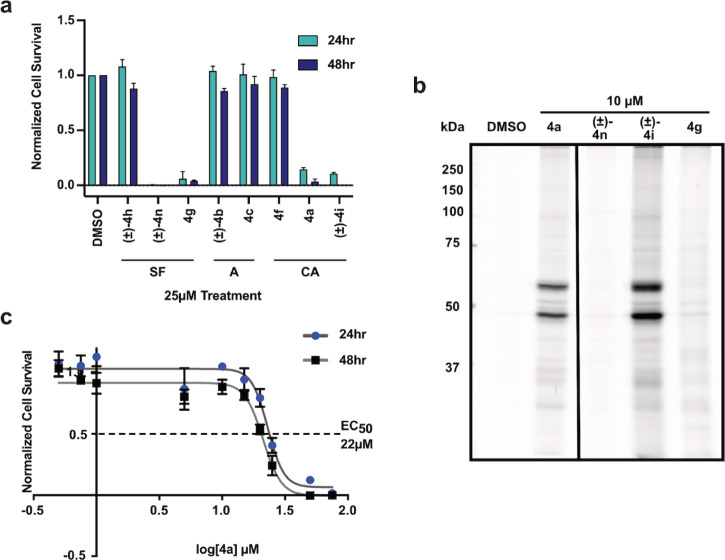
Biological characterization of select electrophilic fully functionalized small-molecule probes (FFSMPs) in MCF10CA1a cells. (a) Cell proliferation screen of eight of the most potent electrophilic FFSMP library members upon incubation with MCF10CA1a cells at 25 μM concentrations for 24 and 48 h. Cell survival was assessed by the MTT reagent relative to a DMSO control. SF = sulfonyl fluoride, A = acrylamide, CA = alpha-chloro amide. (b) In-gel fluorescence assay of proteins covalently modified by each of four electrophilic FFSMPs upon treatment of live cells with 10 μM of FFSMP for 24 h. (c) Concentration-dependent cell survival data for electrophilic FFSMP **4a** upon treatment of MCF10CA1a cells at 24 and 48 h with 0.5–75 μM concentrations.

Phenotypic screening was performed at 25 μM FFSMP concentrations as both 24- and 48-h treatments revealed several compounds with potent antiproliferative activity (Figure 1a). The four most potent FFSMPs (i.e., (±) alpha-chloroacetamides **4a** and (±)-**4i**, and aryl sulfonyl fluorides **4g** and (±)-**4n**) were selected for further characterization.

The general protein-labeling promiscuity of FFSMPs **4a**, **4g**, (±)-**4i**, and (±)-**4n** was visualized by using in-gel fluorescence. Briefly, MCF10CA1a cells treated with each of the FFSMPs were lysed, and then a fluorophore was appended to probe-labeled proteins using copper(I)-catalyzed azide–alkyne cycloaddition (CuAAC).^57^ Probe-labeled proteins were then visualized upon gel electrophoresis and in-gel fluorescence imaging. In-gel fluorescence studies displayed a similar protein-labeling pattern for two of the four FFSMPs (i.e., alpha-chloro amides **4a** and (±)-**4i**), with dominant labeling of protein bands around ∼50 kDa (Figure 1b). Surprisingly, the two aryl sulfonyl fluoride FFSMPs, **4g** and (±)-**4n**, displayed minimal covalent protein labeling by in-gel fluorescence despite possessing potent antiproliferative activity. The observed antiproliferative activity for these latter two FFSMPs could potentially arise from covalent modification of a low-abundance protein target that is not easily visualized by gel or is unstable upon modification. Alternatively, the bioactivity of FFSMPs **4g** and (±)-**4n** could result from noncovalent interactions of these compounds with a cellular protein target.

For further drug target characterization, we focused on alpha-chloroamide containing U-4CR-derived FFSMP **4a** due to its stereochemical simplicity, potent antiproliferative activity, and selective targeting of two distinct protein bands at ∼50 kDa (Figure 1b). Concentration-dependent analysis of the effects of FFSMP **4a** on cell proliferation demonstrated an EC_50_ value of ∼22 μM (Figure 1c). We utilized structurally similar *p*-3CR-derived FFSMP **4c** as a negative control in our chemoproteomic target identification experiments given that this FFSMP displayed a relative lack of antiproliferative activity.

**Figure 2:**
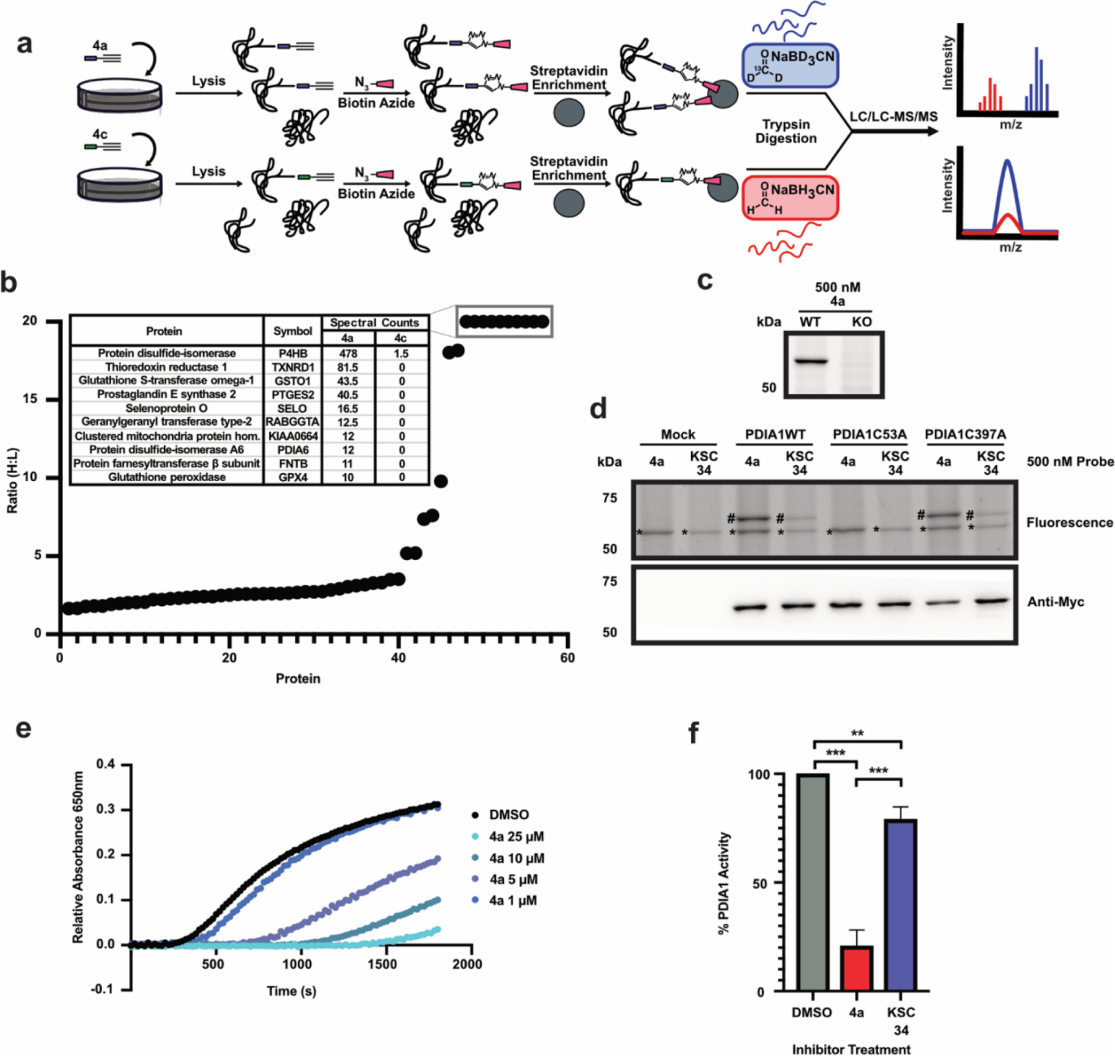
Characterization of the protein targets of electrophilic fully functionalized small-molecule probe (FFSMP) **4a**. (a) Workflow for quantitative proteomic analysis to identify protein targets of biologically active FFSMP **4a** versus biologically inactive FFSMP **4c** as a negative control. MCF10CA1a cells were treated with 500 nM of FFSMP **4a** or **4c** for 1 h. The cells were then lysed and subjected to CuAAC with biotin-azide, followed by FFSMP-modified protein enrichment using streptavidin beads. Peptides resulting from on-bead trypsin digestion were isotopically labeled by reductive di-methylation (ReDiMe) with light (biologically inactive FFSMP **4c**) and heavy (biologically active FFSMP **4a**) formaldehyde, respectively. The isotopically tagged peptides were analyzed by LC/LC-MS/MS to afford peptide identification and quantification in the form of heavy:light (H:L) isotopic ratios. (b) H:L ratios for proteins identified in two biological replicates of **4a**-:**4c**-treated samples. The inset displays average spectral counts for those proteins displaying H:L ratios of ∼20, which indicates a “singleton” protein that is only found in the heavy **4a** sample. (c) In-gel fluorescence demonstrating electrophilic FFSMP **4a** (500 nM for 1 h) labeling in SKOV-3 wildtype (WT) and PDIA1 knockout (KO) cells. (d) In-gel fluorescence displaying electrophilic FFSMP **4a** (500 nM for 1 h) labeling in PDIA1 wildtype (WT) and active-site cysteine mutant-expressing (C53A and C397A) HEK293T cells. Electrophilic FFSMP **4a** protein labeling was compared to that of KSC-34, a previously characterized PDIA1 covalent inhibitor. Covalent labeling of endogenous PDIA1 (*) and overexpressed PDIA1 (#) was observed. Overexpression of PDIA1 was confirmed by anti-myc Western blot. (e) Insulin reductase assay to monitor PDIA1 activity upon treatment with DMSO and electrophilic FFSMP **4a**. (f) PDIA1 activity upon treatment of KSC-34 and biologically active electrophilic FFSMP **4a** (5 μM; 30 min).

To determine the bioactive protein target(s) of FFSMP **4a**, quantitative mass-spectrometry experiments were pursued to identify proteins selectively modified by **4a**, relative to biologically inactive FFSMP **4c** as a negative control (Figure 2a). Briefly, MCF10CA1a cells were treated with biologically active **4a** or biologically inactive **4c** at 500 nM concentrations for 1 h. The cells were then lysed, and biotin was appended to probe-labeled proteins using biotin-azide and CuAAC. Biotinylated proteins were then enriched on streptavidin resin and subjected to on-bead trypsin digestion. To enable quantitative comparisons of the protein targets of **4a** and **4c**, peptide mixtures were subjected to reductive dimethylation (ReDiMe)^58^ with heavy (**4a**) or light (**4c**) formaldehyde, respectively. The resulting peptide mixtures were then analyzed by tandem mass spectrometry (LC-LC/MS-MS) to identify and quantify the abundance of each labeled protein in **4a**- and **4c**-treated samples. Protein targets selective for biologically active **4a** are expected to display high heavy:light (H:L) ratios. In turn, several proteins were identified with H/L ratio values of ∼20, indicating that these proteins were only labeled by biologically active **4a** and not by biologically inactive **4c** (Figure 2b). The resulting mass spectrometry analysis identified ∼50 proteins (Table S1), of which several displayed H:L ratio values of 20, indicating that these proteins were “singleton” hits that were only labeled by biologically active **4a** and not by biologically inactive **4c** (Figure 2b). Proteins that were selectively modified by FFSMP **4a** included several well-characterized proteins that are known to contain highly reactive cysteine or selenocysteine residues, including protein disulfide isomerase 1 (PDI),^59^ thioredoxin reductase 1 (TSNRD1),^60^ and glutathione S-transferase omega 1 (GSTO1)^61^ (Figure 2b). Many of these proteins have been previously implicated in cancer pathogenesis, and the antiproliferative activity observed for FFSMP **4a** likely stems from the covalent modification of multiple protein targets within the MCF10CA1a cell line.

To confirm our mass spectrometry data and validate the ability of biologically active FFSMP **4a** to covalently modify cysteine-containing proteins, we further characterized PDIA1 as a target. Specifically, PDIA1 is an oxidoreductase enzyme primarily localized to the endoplasmic reticulum that facilitates disulfide-bond formation, reduction, and isomerization within nascent proteins.^59^ It is known that PDIA1 contains two active-site domains, classified as **a** and **a’**, each of which contain two highly reactive cysteines within a CGHC motif.^59,62^ Additionally, the two N-terminal cysteines, C53 and C397, within each CGHC motif are known to be highly reactive.^63^

To confirm PDIA1 as a target of FFSMP **4a**, protein labeling by this FFSMP in a SKOV-3 cell line deficient in PDIA1 was evaluated. Specifically, FFSMP **4a** labeling of the ∼50 kDa band present in wildtype SKOV-3 cells was abolished in the PDIA1-deficient SKOV-3 cells, thus confirming PDIA1 as a predominant protein target of biologically active FFSMP **4a** (Figure 2c). To determine the specific cysteine that is targeted by antiproliferative FFSMP **4a**, wildtype PDIA1 and cysteine mutants (C53A and C397A) were overexpressed in HEK293T cells, and protein labeling by FFSMP **4a** was evaluated. Specifically, no protein labeling was observed for the C53A mutant, confirming that protein labeling by FFSMP **4a** was selectively occurring at the N-terminal cysteine of the CGHC motif in the **a** domain of PDIA1 (Figure 2d). Interestingly, this protein labeling is similar to the activity of a previously reported **a**-site inhibitor of PDIA1, KSC-34.^64^

Lastly, the effects of FFSMP **4a** on PDIA1 enzyme activity *in vitro* was evaluated using an assay that monitors the aggregation of insulin induced by PDIA1-mediated reduction of disulfide bonds within insulin.^65^ Specifically, FFSMP **4a** was shown to inhibit PDIA1 at low μM concentrations (Figure 2e) and displayed a higher inhibition potency than KSC-34 as an alternative a-site inhibitor of PDIA1 (Figure 2f).^64^ Collectively, these experimental data confirm that FFSMP **4a** covalently modifies C53 of PDIA1, resulting in the inhibition of PDIA1 enzyme activity, which could contribute to the antiproliferative phenotype observed for FFSMP **4a** in MCF10CA1a cells.

## Discussion

Here, we show that minimalist bifunctional isocyanide **3** is a valuable building block for creating FFSMPs (**4**) with significant appendage, scaffold, and PRFG-variation for combined phenotypic screening–target identification campaigns. Seventeen examples from a total of thirty-four FFSMPs are presented here, namely, by employing isocyanide **3** in five different IMCRs (i.e., U-4CR, *p*-3CR, UT-4CR, U-5C-4CR, and UBL-3CR), as well as a sequential/union of MCRs strategy featuring carboxylic acids derived from four different MCRs (i.e., UT-4CR, Castagnoli–Cushman, Groebke–Blackburn–Bienaymé, and Petasis reactions) and a U-4CR-intramolecular cyclization strategy. Subsequent Boc deprotection and arming with four different electrophiles (i.e., chloronitrobenzoyl as an S_N_Ar electrophile, aryl sulfonyl fluoride as a SuFEx electrophile, alpha-chloroacetamide as an S_N_2-reactive electrophile, and acrylamide as a conjugate addition electrophile/Michael acceptor) and three different photoreactive groups (i.e., diradical-producing benzophenone, nitrene-producing aryl azide, and carbene-producing aliphatic diazirine) gave affinity labeling- and photoaffinity labeling-based FFSMPs, respectively. Subsequent phenotypic screening of the electrophilic FFSMP library members led to the identification of four compounds (i.e., **4a**, **4g**, (±)-**4i**, and (±)-**4n**) possessing appreciable antiproliferative activity against MCF10CA1a breast cancer cells. In turn, chemoproteomic target identification methods were employed to discern protein disulfide isomerase A1 (PDIA1) as a major protein target of U-4CR-derived FFSMP **4a**. In vitro characterization demonstrated that FFSMP **4a** can inhibit PDIA1 activity by covalently modifying C53.

Concerning fundamental biological research and biomedical applications, chemical tools that covalently modify proteins are of great interest.^66^ In particular, comprehensive characterization of protein–ligand interactions across a proteome at the preclinical stage allows for well-informed lead optimization cycles that can lower drug attrition.^67^ Over the past 15 years, combinatorial and modular methods to synthesize covalent small-molecule modulators of protein activity have proven valuable in developing new drug-like molecules, as well as activity- and affinity-based chemical probes for advanced chemoproteomic profiling.^68^ Such synthetic approaches that produce a structurally diverse panel of covalent chemical probes in a minimal number of steps can: (1) increase the chances of identifying a small-molecule chemical probe for a protein of interest and/or (2) be used in integrated phenotypic screening-target identification campaigns. The latter offers the intriguing possibility of uncovering covalent modifiers for proteins that have yet to be identified and/or targeted.

The aforementioned ligand-/protein-recognition element, a PRFG, and a reporter group are all present in FFSMPs regardless of the protein-labeling technique used (i.e., affinity labeling versus photoaffinity labeling). However, merging these three components into a single FFSMP is nontrivial and can require a lot of synthetic effort, especially when it is necessary to create several different FFSMP variations to identify the best chemical probe for a given application. An ideal FFSMP synthesis approach should have a minimal number of purification steps, while permitting simple diversification of the chemical scaffold. Therefore, modular MCR approaches have been suggested and pursued, primarily to simplify the synthesis of FFSMPs.^68^ In these approaches, activity- and affinity-based FFSMPs are prepared in a time- and atom-efficient manner from various probe fragments using readily available and/or simple-to-synthesize building blocks. When MCRs are employed, for instance, it is simple to prepare various versions of a lead FFSMP by parallel synthesis, specifically by swapping out one structural entity (e.g., a privileged structure substituent, Topliss optimization moiety, or a different PRFG) with a structural entity that has various desired and/or optimized chemical properties. Modular MCRs are a very alluring method to explore a large volume of biologically relevant chemical space in a time-efficient manner, namely, by quickly screening various ligand-PRFG combinations. This is especially true when FFSMP libraries are pursued for integrated phenotypic screening–target identification campaigns. These investigations aim to identify individual FFSMPs with distinct biological properties that may involve novel therapeutic targets or mechanisms of action. Given protein binding and pharmacological potency are investigated concurrently with chemical biology function, this experimental strategy may increase the effectiveness of finding FFSMPs.^69^ Finally, FFSMPs with click chemistry handles enable quick probe evaluation by orthogonal assays carried out simultaneously, such as by affinity enrichment and mass spectrometry. These orthogonal experiments are especially beneficial for FFSMPs that utilize photoaffinity labeling, which frequently results in poor cross-linking yields and/or probe-protein adducts that may not be acceptable for intact mass spectrometry analysis.^69^

A wide range of protein-reactive functional groups (PRFGs) have been reported over the years for covalent small-molecules, including: (1) electrophiles with various geometries, chemical compositions, and covalent bond-forming chemistries that can specifically react with a single type of amino acid residue,^70^ and (2) photoreactive functional groups, which typically form either highly reactive carbene, nitrene, or biradical species upon UV-irradiation.^19^ In contrast to constructing electrophilic activity-based probes, choosing a PRFG while designing photoreactive affinity-based FFSMPs is often more complicated and less straightforward. For instance, to ensure that a protein and a FFSMP make a covalent bond effectively, the PRFG must be placed close to an appropriately reactive amino acid of the protein, necessitating knowledge of the target protein’s three-dimensional structure. In brief, it is strongly advised to synthesize and test numerous FFSMPs with various PRFGs until a suitable FFSMP is obtained that has the desired protein profile in terms of target engagement, selectivity, and/or cross-linking site(s), rate, and yield/efficiency, especially in the absence of structural information about a target protein or when target proteins are unknown, as in phenotypic screening.^69,71^ As one example concerning electrophilic chemical probes and traditional affinity labeling, it is well-known that cysteines exhibit a wide range of chemical reactivity.^72^ As a result, the reactivity of an electrophilic FFSMP that targets a cysteine must be balanced to allow efficient labeling of the target protein while avoiding being overly reactive to cause unintended nonselective labeling of numerous off-target proteins.^73^ Analogously, slow photoactivation and/or low photoaffinity labeling yields often call for investigating different photoreactive PRFGs to improve protein-labeling efficiency and/or target protein capture above the analytical detection limit.^71^ Because the optimal photoreactive PRFG can vary based on the physicochemical and intrinsic photochemical characteristics of the FFSMP and the nature of the probe’s association with a target protein, variability in photo-cross-linking yields is frequently observed with photoaffinity labeling.^19^

In addition to giving a more accurate picture of a compound’s actual target engagement profile and ligand–protein interactions, the chance that multiple FFSMP structure–activity relationships (SAR) may not match is another reason to screen alternative PRFGs.^69^ For example, the most pharmacologically potent FFSMP may not necessarily be the most efficient at covalently labeling its target protein for a given application (e.g., protein imaging).^69^ It is also known that despite the advantage that photoreactive FFSMPs gain from being less dependent on specific amino acid targeting due to the promiscuous chemical reactivity of carbenes, diradicals, and nitrenes, covalent protein labeling efficiency levels are frequently relatively lower and more nonspecific when using photoaffinity labeling versus affinity labeling as orthogonal experimental strategies.^19^ Additionally, photoaffinity labeling has a distinct advantage over affinity labeling using electrophilic FFSMPs in that weak or transient probe–protein interactions can be captured and identified covalently. This may allow a more accurate representation of a small-molecule’s noncovalently engaged on- and off-targets. Finally, context-dependent factors can play a role in determining the photoaffinity labeling cross-linking yield. These factors include the proximity and orientation of a photoreactive group to an appropriate amino acid within a target protein, as well as the rate of desired covalent bond formation between an FFSMP and a protein versus undesired reactive species-quenching reactions.^19^

Conducting a chemical reactivity evaluation of electrophilic covalent binders from the start of the drug discovery process is crucial concerning FFSMP library creation, PRFG selection, and discovering significant starting points for covalent drug development.^18^ Since nonspecific, reactivity-driven protein binding is the most common confounding factor for electrophilic covalent-acting compounds, it is vital to consider this at the very beginning and throughout the optimization process of a covalent chemical probe. In particular, the ability to adjust the chemical reactivity of the electrophile within an FFSMP gives a variable that may be changed to improve the protein selectivity and pharmacological effectiveness. The reactivity of FFSMPs with electrophilic PRFGs should be carefully tuned to be stable under physiological aqueous conditions and sufficiently reactive in the ligand-binding site of targeted proteins to achieve an excellent protein target selectivity. Finally, the reactivity profile of electrophilic compounds can be influenced by factors other than inherent chemical reactivity, such as chemoselectivity, mode of interaction with a nucleophile, molecular size, and the reversibility of covalent engagement.^18^

There is a constant need for FFSMP libraries of significantly greater size and chemical/structural diversity for phenotypic screening campaigns.^25^ In contrast to our initial study concerning the synthesis of FFSMPs **2** via bifunctional isocyanide **1**,^23^ the PRFG and the reporter group are generally integrated in separate/distinct MCR inputs/building blocks in the vast majority of FFSMP libraries synthesized by MCRs to date.^68^ However, since at least one MCR component must contain the reporter group and another MCR component must have the PRFG, such an FFSMP design strategy intrinsically limits the diversity of the building blocks that may be used to prepare the FFSMP library. Contrarily, bifunctional isocyanides **1** and **3** can facilitate IMCR-based FFSMP synthesis and provide access to structurally diverse FFSMPs **2** and **4**, namely because building blocks **1** and **3** directly or indirectly (i.e., introduced upon further synthetic manipulation) contain a PRFG and terminal alkyne reporter group within a single sterically minimized entity.

Projecting forward with the FFSMP synthesis method described herein, the most frequent last step in synthesizing a covalent compound is N-acylation of a 1° or 2° aliphatic or aromatic amine.^74^ With this in mind, a host of different electrophilic or photoreactive PRFG building blocks,^74^ in theory, could potentially be added via N-acylation of the requisite 2° aliphatic amine generated after the Boc deprotection step of the “IMCR–Boc deprotection–arming” sequence. However, chemoselectivity concerns may be a significant limitation of the method stated method. For example, traditional acid-mediated Boc deprotection conditions (i.e., CF_3_CO_2_H, CH_2_Cl_2_) were used to synthesize FFSMPs **4** via minimalist bifunctional isocyanide **3**. It is possible that such acidic reaction conditions could potentially: (1) unmask another relatively nucleophilic functional group (e.g., a 1° or 2° aliphatic or aromatic amine) somewhere else within the FFSMP, thus compromising the chemoselectivity of the final N-acylation PRFG-arming step that is linked to bifunctional isocyanide **3**, and/or (2) compromise potentially acid-sensitive structural entities within the designed FFSMP. This leads to the speculation that derivatives of minimalist bifunctional isocyanide **3** possessing alternative 2° aliphatic amine protecting groups to Boc (e.g., base-labile Fmoc, fluoride-labile Teoc, Pd-labile Alloc, etc.) might address such chemoselectivity problems.^75^ However, Cbz and benzyl amine protecting groups are likely to be poor choices here, as traditional hydrogenolysis deprotection conditions could also undesirably reduce the terminal alkyne latent affinity handle in the FFSMP to either an alkene or alkane, thus compromising subsequent CuAAC-based proteomic techniques.

## Conclusion

Appendage-diversity around a single chemical scaffold while examining a single PRFG has primarily been the status quo when pursuing FFSMPs,^6,8,9,11,12,14−17,23^ with minimal exceptions.^7,10,13,68^ However, if the desired goal is to discover new drug targets or compounds with novel mechanisms of action via phenotypic screening–target identification campaigns, such an FFSMP design strategy inherently limits chemical space sampling or efficient target protein capture significantly. In contrast, the general “IMCR–Boc deprotection–arming” synthetic approach described herein involving bifunctional isocyanide **3** (Scheme 1B) challenges the status quo by producing FFSMPs (**4**) with increased appendage- and scaffold-diversity, as well as the ability to investigate either electrophilic or photoreactive PRFGs via late-stage N-acylation. Such a synthetic strategy is expected to (1) rapidly produce structurally-diverse FFSMPs modularly and efficiently that sample a larger volume of chemical space in integrated phenotypic screening-target identification pursuits and/or (2) allow for the most effective experimentally-guided tailoring of FFSMPs and cross-linking protocols to specific protein targets. Concerning the latter, more research may be done on cross-linking yields, physicochemical properties, biochemical and cellular potencies, crystallography, and live-cell MS-based proteomics to determine the best FFSMP design.

Finally, synthetically valuable bifunctional isocyanide **3** addresses the two most prominent disadvantages concerning our previously reported^23^ bifunctional isocyanide (**1**) in the synthesis of structurally diverse FFSMPs (**2**) for streamlined phenotypic screening–target identification studies. Those two most prominent disadvantages once again being: (1) limited sampling of biologically relevant chemical space toward discovering new drug targets and/or small-molecules with potentially novel mechanisms of action and (2) the production of more linear/conformationally flexible FFSMPs (**2**) that possess a greater tendency to cause undesirable nonspecific protein labeling. We anticipate that bifunctional isocyanide **3** can help answer the call for FFSMP libraries of much greater size, chemical diversity (i.e., appendage-, scaffold-, and PRFG-diversity), and structural complexity to be used in drug and target discovery campaigns.^25^ Our primary motivation for pursuing structurally-diverse FFSMP libraries such as **2** and **4** is (1) to meet the current and future demand for new drug targets and small-molecules with novel mechanisms of action,^76^ and (2) to address the ambitious goal of Target 2035, which is to identify a small-molecule modulator for each protein within the human proteome by the year 2035.^77,78^

In short, we believe bifunctional isocyanide **3** represents a valuable addition to the growing toolbox of trifunctionalized building blocks for synthesizing structurally diverse FFSMPs^68^ given: (1) IMCRs can be easily automated one-pot chemical reactions that are ideal for combinatorial chemistry and high-speed parallel synthesis, in that they can be tailored to include desired chemical properties and probe structure–activity relationships,^79^ and (2) the sizable commercial availability of carboxylic acids, 1° or 2° amines, ketones, and aldehydes as standard IMCR inputs, as opposed to the restricted commercial availability of isocyanides.^80^ Similar to the discovery of U-4CR-based electrophilic FFSMP **4a** as a potent inhibitor of protein disulfide isomerase A1 (PDIA1) disclosed herein, we will continue to report our development and application of practical protein-reactive synthetic building blocks toward producing structurally-diverse FFSMPs, as well as outcomes from phenotypic screening and target identification studies involving such compounds.

## Supporting Information Available

General experimental procedures for organic synthesis, synthetic schemes, and experimental procedures for organic synthesis of the representative FFSMP examples depicted herein; characterization data for all novel compounds; NMR spectra for all novel compounds; experimental details concerning the biological testing of compounds and chemoproteomic target identification experiments; and the chemical structures of all FFSMPs produced during this work (i.e., derived from minimalist bifunctional isocyanide **3**) (PDF)

## Abbreviations

3-D: three-dimensional
Alloc: N-allyloxycarbonyl
Boc: *tert*-butyloxycarbonyl
Cbz: carboxybenzyl)
CuAAC: copper(I)-catalyzed azide–alkyne cycloaddition
DMSO: dimethyl sulfoxide
EC_50_: half maximal effective concentration
FFSMP: fully functionalized small-molecule probe
Fmoc: fluorenylmethoxycarbonyl
GSTO1: glutathione S-transferase omega 1
IMCR: isocyanide-based multicomponent reaction
KO: knockout
LC: liquid chromatography
MeOH: methanol
MCR: multicomponent reaction
MS: mass spectrometry
MTT: 3-(4,5-dimethylthiazol-2-yl)-2,5-diphenyltetrazolium bromide
NMR: nuclear magnetic resonance
P-3CR: Passerini three-component reaction
PDIA1: protein disulfide isomerase A1
PRFG: protein-reactive functional group
ReDiMe: reductive demethylation
SAR: structure–activity relationship
S_N_2: bimolecular nucleophilic substitution
S_N_Ar: nucleophilic aromatic substitution, SuFEx, sulfur(VI) fluoride exchange
Teoc: 2-(trimethylsilyl)ethoxycarbonyl
TMS-N_3_: trimethylsilyl azide
TSNRD1: thioredoxin reductase 1
U-4CR: Ugi four-component reaction
–5C-4CR: Ugi five-center-four-component reaction
UBL-3CR: Ugi beta-lactam three-component reaction
UT-4CR: Ugi tetrazole four-component reaction
UV: ultraviolet
WT: wildtype

## References

[ref1] Nurk S.; Koren S.; Rhie A.; Rautiainen M.; Bzikadze A. V.; Mikheenko A.; Vollger M. R.; Altemose N.; Uralsky L.; Gershman A.; et al. The complete sequence of a human genome. Science 2022, 376 (6588), 44–53. 10.1126/science.abj6987. 35357919 PMC9186530

[ref2] Oprea T. I.; Bologa C. G.; Brunak S.; Campbell A.; Gan G. N.; Gaulton A.; Gomez S. M.; Guha R.; Hersey A.; Holmes J.; et al. Unexplored therapeutic opportunities in the human genome. Nat. Rev. Drug Discovery 2018, 17 (5), 377 10.1038/nrd.2018.52. 29567993

[ref3] Antolin A. A.; Tym J. E.; Komianou A.; Collins I.; Workman P.; Al-Lazikani B. Objective Quantitative, Data-Driven Assessment of Chemical Probes. Cell Chem. Biol. 2018, 25 (2), 194–205.E5. 10.1016/j.chembiol.2017.11.004. 29249694 PMC5814752

[ref4] Arrowsmith C. H.; Audia J. E.; Austin C.; Baell J.; Bennett J.; Blagg J.; Bountra C.; Brennan P. E.; Brown P. J.; Bunnage M. E.; et al. The promise and peril of chemical probes. Nat. Chem. Biol. 2015, 11 (8), 536–541. 10.1038/nchembio.1867. 26196764 PMC4706458

[ref5] Schreiber S. L.; Kotz J. D.; Li M.; Aube J.; Austin C. P.; Reed J. C.; Rosen H.; White E. L.; Sklar L. A.; Lindsley C. W.; et al. Advancing Biological Understanding and Therapeutics Discovery with Small-Molecule Probes. Cell 2015, 161 (6), 1252–1265. 10.1016/j.cell.2015.05.023. 26046436 PMC4564295

[ref6] Cisar J. S.; Cravatt B. F. Fully functionalized small-molecule probes for integrated phenotypic screening and target identification. J. Am. Chem. Soc. 2012, 134 (25), 10385–10388. 10.1021/ja304213w. 22667687 PMC3426452

[ref7] Banerjee R.; Pace N. J.; Brown D. R.; Weerapana E. 1,3,5-Triazine as a modular scaffold for covalent inhibitors with streamlined target identification. J. Am. Chem. Soc. 2013, 135 (7), 2497–2500. 10.1021/ja400427e. 23379904

[ref8] Couvertier S. M.; Weerapana E. Cysteine-reactive chemical probes based on a modular 4-aminopiperidine scaffold. MedChemcomm 2014, 5 (3), 358–362. 10.1039/C3MD00289F.

[ref9] Kambe T.; Correia B. E.; Niphakis M. J.; Cravatt B. F. Mapping the protein interaction landscape for fully functionalized small-molecule probes in human cells. J. Am. Chem. Soc. 2014, 136 (30), 10777–10782. 10.1021/ja505517t. 25045785 PMC4120992

[ref10] Backus K. M.; Correia B. E.; Lum K. M.; Forli S.; Horning B. D.; Gonzalez-Paez G. E.; Chatterjee S.; Lanning B. R.; Teijaro J. R.; Olson A. J.; et al. Proteome-wide covalent ligand discovery in native biological systems. Nature 2016, 534 (7608), 570–574. 10.1038/nature18002. 27309814 PMC4919207

[ref11] Parker C. G.; Galmozzi A.; Wang Y.; Correia B. E.; Sasaki K.; Joslyn C. M.; Kim A. S.; Cavallaro C. L.; Lawrence R. M.; Johnson S. R.; et al. Ligand and Target Discovery by Fragment-Based Screening in Human Cells. Cell 2017, 168 (3), 527–541.e29. 10.1016/j.cell.2016.12.029. 28111073 PMC5632530

[ref12] Galmozzi A.; Parker C. G.; Kok B. P.; Cravatt B. F.; Saez E. Discovery of Modulators of Adipocyte Physiology Using Fully Functionalized Fragments. Methods Mol. Biol. 2018, 1787, 115–127. 10.1007/978-1-4939-7847-2_9. 29736714 PMC6010189

[ref13] Liu W.; Zhang Z.; Zhang Z. M.; Hao P.; Ding K.; Li Z. Integrated phenotypic screening and activity-based protein profiling to reveal potential therapy targets of pancreatic cancer. Chem. Commun. 2019, 55 (11), 1596–1599. 10.1039/C8CC08753A. 30656306

[ref14] Grant E. K.; Fallon D. J.; Hann M. M.; Fantom K. G. M.; Quinn C.; Zappacosta F.; Annan R. S.; Chung C. W.; Bamborough P.; Dixon D. P.; et al. A Photoaffinity-Based Fragment-Screening Platform for Efficient Identification of Protein Ligands. Angew. Chem., Int. Ed. 2020, 59 (47), 21096–21105. 10.1002/anie.202008361. 32745361

[ref15] Conway L. P.; Jadhav A. M.; Homan R. A.; Li W.; Rubiano J. S.; Hawkins R.; Lawrence R. M.; Parker C. G. Evaluation of fully-functionalized diazirine tags for chemical proteomic applications. Chem. Sci. 2021, 12 (22), 7839–7847. 10.1039/D1SC01360B. 34168837 PMC8188597

[ref16] Wang Y.; Dix M. M.; Bianco G.; Remsberg J. R.; Lee H. Y.; Kalocsay M.; Gygi S. P.; Forli S.; Vite G.; Lawrence R. M.; et al. Expedited mapping of the ligandable proteome using fully functionalized enantiomeric probe pairs. Nat. Chem. 2019, 11 (12), 1113–1123. 10.1038/s41557-019-0351-5. 31659311 PMC6874898

[ref17] West A. V.; Muncipinto G.; Wu H. Y.; Huang A. C.; Labenski M. T.; Jones L. H.; Woo C. M. Labeling Preferences of Diazirines with Protein Biomolecules. J. Am. Chem. Soc. 2021, 143 (17), 6691–6700. 10.1021/jacs.1c02509. 33876925 PMC11647638

[ref18] Gehringer M.; Laufer S. A. Emerging and Re-Emerging Warheads for Targeted Covalent Inhibitors: Applications in Medicinal Chemistry and Chemical Biology. J. Med. Chem. 2019, 62 (12), 5673–5724. 10.1021/acs.jmedchem.8b01153. 30565923

[ref19] West A. V.; Woo C. M. Photoaffinity Labeling Chemistries Used to Map Biomolecular Interactions. Isr. J. Chem. 2023, 63 (1–2), e202200081 10.1002/ijch.202200081.

[ref20] Parker C. G.; Pratt M. R. Click Chemistry in Proteomic Investigations. Cell 2020, 180 (4), 605–632. 10.1016/j.cell.2020.01.025. 32059777 PMC7087397

[ref21] Jones L. H.; Neubert H. Clinical chemoproteomics—Opportunities and obstacles. Sci. Transl. Med. 2017, 9 (386), eaaf7951 10.1126/scitranslmed.aaf7951. 28424333

[ref22] Moellering R. E.; Cravatt B. F. How chemoproteomics can enable drug discovery and development. Chem. Biol. 2012, 19 (1), 11–22. 10.1016/j.chembiol.2012.01.001. 22284350 PMC3312051

[ref23] Jackson P.; Lapinsky D. J. Appendage and Scaffold Diverse Fully Functionalized Small-Molecule Probes via a Minimalist Terminal Alkyne-Aliphatic Diazirine Isocyanide. J. Org. Chem. 2018, 83 (18), 11245–11253. 10.1021/acs.joc.8b01831. 30132667

[ref24] Deng Z. L.; Du C. X.; Li X.; Hu B.; Kuang Z. K.; Wang R.; Feng S. Y.; Zhang H. Y.; Kong D. X. Exploring the biologically relevant chemical space for drug discovery. J. Chem. Inf. Model. 2013, 53 (11), 2820–2828. 10.1021/ci400432a. 24125686

[ref25] Oeljeklaus J.; Kaschani F.; Kaiser M. Streamlining chemical probe discovery: Libraries of ″fully functionalized″ small molecules for phenotypic screening. Angew. Chem., Int. Ed. 2013, 52 (5), 1368–1370. 10.1002/anie.201207306. 23281006

[ref26] Sauer W. H.; Schwarz M. K. Molecular shape diversity of combinatorial libraries: A prerequisite for broad bioactivity. J. Chem. Inf. Comput. Sci. 2003, 43 (3), 987–1003. 10.1021/ci025599w. 12767158

[ref27] Galloway W. R.; Isidro-Llobet A.; Spring D. R. Diversity-oriented synthesis as a tool for the discovery of novel biologically active small molecules. Nat. Commun. 2010, 1, 80 10.1038/ncomms1081. 20865796

[ref28] O’Connell K. M. G.; Galloway W. R. J. D.; Spring D. R. The Basics of Diversity-Oriented Synthesis. In Diversity-Oriented Synthesis; John Wiley & Sons, Inc, 2013; pp. 1 26.

[ref29] Li Z.; Hao P.; Li L.; Tan C. Y.; Cheng X.; Chen G. Y.; Sze S. K.; Shen H. M.; Yao S. Q. Design and synthesis of minimalist terminal alkyne-containing diazirine photo-crosslinkers and their incorporation into kinase inhibitors for cell- and tissue-based proteome profiling. Angew. Chem., Int. Ed. 2013, 52 (33), 8551–8556. 10.1002/anie.201300683. 23754342

[ref30] Duarte C. D.; Barreiro E. J.; Fraga C. A. Privileged structures: A useful concept for the rational design of new lead drug candidates. Mini-Rev. Med. Chem. 2007, 7 (11), 1108–1119. 10.2174/138955707782331722. 18045214

[ref31] Welsch M. E.; Snyder S. A.; Stockwell B. R. Privileged scaffolds for library design and drug discovery. Curr. Opin. Chem. Biol. 2010, 14 (3), 347–361. 10.1016/j.cbpa.2010.02.018. 20303320 PMC2908274

[ref32] Kim J.; Kim H.; Park S. B. Privileged structures: Efficient chemical ″navigators″ toward unexplored biologically relevant chemical spaces. J. Am. Chem. Soc. 2014, 136 (42), 14629–14638. 10.1021/ja508343a. 25310802

[ref33] Topliss J. G. Utilization of operational schemes for analog synthesis in drug design. J. Med. Chem. 1972, 15 (10), 1006–1011. 10.1021/jm00280a002. 5069767

[ref34] Richter L. Topliss Batchwise Schemes Reviewed in the Era of Open Data Reveal Significant Differences between Enzymes and Membrane Receptors. J. Chem. Inf. Model. 2017, 57 (10), 2575–2583. 10.1021/acs.jcim.7b00195. 28934851

[ref35] Park H.; Koo J. Y.; Srikanth Y. V.; Oh S.; Lee J.; Park J.; Park S. B. Nonspecific protein labeling of photoaffinity linkers correlates with their molecular shapes in living cells. Chem. Commun. 2016, 52 (34), 5828–5831. 10.1039/C6CC01426G. 27043101

[ref36] Guo J.; Li W.; Xue W.; Ye X. S. Transition State-Based Sialyltransferase Inhibitors: Mimicking Oxocarbenium Ion by Simple Amide. J. Med. Chem. 2017, 60 (5), 2135–2141. 10.1021/acs.jmedchem.6b01644. 28165727

[ref37] Ugi I.; Fetzer U.; Eholzer U.; Knupfer H.; Offermann K. Isonitrile Syntheses. Angew. Chem., Int. Ed. 1965, 4 (6), 472–484. 10.1002/anie.196504721.

[ref38] Domling A. Recent developments in isocyanide based multicomponent reactions in applied chemistry. Chem. Rev. 2006, 106 (1), 17–89. 10.1021/cr0505728. 16402771

[ref39] Domling A.; Wang W.; Wang K. Chemistry and biology of multicomponent reactions. Chem. Rev. 2012, 112 (6), 3083–3135. 10.1021/cr100233r. 22435608 PMC3712876

[ref40] Reza Kazemizadeh A.; Ramazani A. Synthetic Applications of Passerini Reaction. Curr. Org. Chem. 2012, 16 (4), 418–450. 10.2174/138527212799499868.

[ref41] Khoury K.; Sinha M. K.; Nagashima T.; Herdtweck E.; Domling A. Efficient assembly of iminodicarboxamides by a ″truly″ four-component reaction. Angew. Chem., Int. Ed. 2012, 51 (41), 10280–10283. 10.1002/anie.201205366. PMC3874142 22968839

[ref42] Fisher J. F.; Mobashery S. Chapter 3. The β-Lactam (Azetidin-2-one) as a Privileged Ring in Medicinal Chemistry. In Privileged Scaffolds in Medicinal Chemistry, Drug Discovery; The Royal Society of Chemistry, 2015; pp. 64 97.

[ref43] Zarganes-Tzitzikas T.; Chandgude A. L.; Domling A. Multicomponent Reactions Union of MCRs and Beyond. Chem. Rec. 2015, 15 (5), 981–996. 10.1002/tcr.201500201. 26455350

[ref44] Madhavachary R.; Wang Q.; Domling A. With unprotected amino acids to tetrazolo peptidomimetics. Chem. Commun. 2017, 53 (61), 8549–8552. 10.1039/C7CC03370B. PMC5588861 28707691

[ref45] Lepikhina A.; Dar’in D.; Bakulina O.; Chupakhin E.; Krasavin M. Skeletal Diversity in Combinatorial Fashion: A New Format for the Castagnoli-Cushman Reaction. ACS Comb. Sci. 2017, 19 (11), 702–707. 10.1021/acscombsci.7b00118. 29019643

[ref46] Al-Tel T. H.; Semreen M. H.; Al-Qawasmeh R. A.; Schmidt M. F.; El-Awadi R.; Ardah M.; Zaarour R.; Rao S. N.; El-Agnaf O. Design, synthesis, and qualitative structure-activity evaluations of novel beta-secretase inhibitors as potential Alzheimer’s drug leads. J. Med. Chem. 2011, 54 (24), 8373–8385. 10.1021/jm201181f. 22044119

[ref47] Portlock D. E.; Ostaszewski R.; Naskar D.; West L. A tandem Petasis–Ugi multi component condensation reaction: Solution phase synthesis of six dimensional libraries. Tetrahedron Lett. 2003, 44 (3), 603–605. 10.1016/S0040-4039(02)02619-9.

[ref48] Koopmanschap G.; Ruijter E.; Orru R. V. Isocyanide-based multicomponent reactions towards cyclic constrained peptidomimetics. Beilstein J. Org. Chem. 2014, 10, 544–598. 10.3762/bjoc.10.50. 24605172 PMC3943360

[ref49] Marcaccini S.; Pepino R.; Pozo M. C. A facile synthesis of 2,5-diketopiperazines based on isocyanide chemistry. Tetrahedron Lett. 2001, 42 (14), 2727–2728. 10.1016/S0040-4039(01)00232-5.

[ref50] Li Z.; Kumar A.; Peshkov A.; Van der Eycken E. V. A domino Ugi/Michael approach for the synthesis of α,β-unsaturated γ-lactams. Tetrahedron Lett. 2016, 57 (7), 754–756. 10.1016/j.tetlet.2016.01.014.

[ref51] Huang Y.; Khoury K.; Chanas T.; Domling A. Multicomponent synthesis of diverse 1,4-benzodiazepine scaffolds. Org. Lett. 2012, 14 (23), 5916–5919. 10.1021/ol302837h. 23157402 PMC3732779

[ref52] Azuaje J.; Perez-Rubio J. M.; Yaziji V.; El Maatougui A.; Gonzalez-Gomez J. C.; Sanchez-Pedregal V. M.; Navarro-Vazquez A.; Masaguer C. F.; Teijeira M.; Sotelo E. Integrated Ugi-based assembly of functionally, skeletally, and stereochemically diverse 1,4-benzodiazepin-2-ones. J. Org. Chem. 2015, 80 (3), 1533–1549. 10.1021/jo502382q. 25560990

[ref53] Yuan D.; Duan Z.; Rao Y.; Ding M.-W. New efficient synthesis of isoquinoline-1,3(2H,4H)-diones and isoindolin-1-ones via sequential Ugi/cyclization reaction. Tetrahedron 2016, 72 (2), 338–346. 10.1016/j.tet.2015.11.051.

[ref54] van Scherpenzeel M.; Moret E. E.; Ballell L.; Liskamp R. M.; Nilsson U. J.; Leffler H.; Pieters R. J. Synthesis and evaluation of new thiodigalactoside-based chemical probes to label galectin-3. ChemBiochem 2009, 10 (10), 1724–1733. 10.1002/cbic.200900198. 19492387

[ref55] Soule H. D.; Maloney T. M.; Wolman S. R.; Peterson W. D. Jr.; Brenz R.; McGrath C. M.; Russo J.; Pauley R. J.; Jones R. F.; Brooks S. C. Isolation and characterization of a spontaneously immortalized human breast epithelial cell line, MCF-10. Cancer Res. 1990, 50 (18), 6075–6086. 1975513

[ref56] Imbalzano K. M.; Tatarkova I.; Imbalzano A. N.; Nickerson J. A. Increasingly transformed MCF-10A cells have a progressively tumor-like phenotype in three-dimensional basement membrane culture. Cancer Cell Int. 2009, 9, 7 10.1186/1475-2867-9-7. 19291318 PMC2666639

[ref57] Rostovtsev V. V.; Green L. G.; Fokin V. V.; Sharpless K. B. A stepwise huisgen cycloaddition process: Copper(I)-catalyzed regioselective ″ligation″ of azides and terminal alkynes. Angew. Chem., Int. Ed. 2002, 41 (14), 2596–2599. 10.1002/1521-3773(20020715)41:14<2596::AID-ANIE2596>3.0.CO;2-4. 12203546

[ref58] Boersema P. J.; Raijmakers R.; Lemeer S.; Mohammed S.; Heck A. J. Multiplex peptide stable isotope dimethyl labeling for quantitative proteomics. Nat. Protoc. 2009, 4 (4), 484–494. 10.1038/nprot.2009.21. 19300442

[ref59] Kozlov G.; Maattanen P.; Thomas D. Y.; Gehring K. A structural overview of the PDI family of proteins. FEBS J. 2010, 277 (19), 3924–3936. 10.1111/j.1742-4658.2010.07793.x. 20796029

[ref60] Zhong L.; Arner E. S.; Holmgren A. Structure and mechanism of mammalian thioredoxin reductase: The active site is a redox-active selenolthiol/selenenylsulfide formed from the conserved cysteine-selenocysteine sequence. Proc. Natl. Acad. Sci. U. S. A. 2000, 97 (11), 5854–5859. 10.1073/pnas.100114897. 10801974 PMC18523

[ref61] Menon D.; Board P. G. A role for glutathione transferase Omega 1 (GSTO1–1) in the glutathionylation cycle. J. Biol. Chem. 2013, 288 (36), 25769–25779. 10.1074/jbc.M113.487785. 23888047 PMC3764784

[ref62] Xu S.; Sankar S.; Neamati N. Protein disulfide isomerase: A promising target for cancer therapy. Drug Discovery Today 2014, 19 (3), 222–240. 10.1016/j.drudis.2013.10.017. 24184531

[ref63] Hatahet F.; Ruddock L. W. Protein disulfide isomerase: A critical evaluation of its function in disulfide bond formation. Antioxid. Redox Signaling 2009, 11 (11), 2807–2850. 10.1089/ars.2009.2466. 19476414

[ref64] Cole K. S.; Grandjean J. M. D.; Chen K.; Witt C. H.; O’Day J.; Shoulders M. D.; Wiseman R. L.; Weerapana E. Characterization of an A-Site Selective Protein Disulfide Isomerase A1 Inhibitor. Biochemistry 2018, 57 (13), 2035–2043. 10.1021/acs.biochem.8b00178. 29521097 PMC5884060

[ref65] Holmgren A. Thioredoxin catalyzes the reduction of insulin disulfides by dithiothreitol and dihydrolipoamide. J. Biol. Chem. 1979, 254 (19), 9627–9632. 10.1016/S0021-9258(19)83562-7. 385588

[ref66] Dalton S. E.; Campos S. Covalent Small Molecules as Enabling Platforms for Drug Discovery. ChemBiochem 2020, 21 (8), 1080–1100. 10.1002/cbic.201900674. 31833626

[ref67] Comess K. M.; McLoughlin S. M.; Oyer J. A.; Richardson P. L.; Stockmann H.; Vasudevan A.; Warder S. E. Emerging Approaches for the Identification of Protein Targets of Small Molecules - A Practitioners’ Perspective. J. Med. Chem. 2018, 61 (19), 8504–8535. 10.1021/acs.jmedchem.7b01921. 29718665

[ref68] van der Zouwen A. J.; Witte M. D. Modular Approaches to Synthesize Activity- and Affinity-Based Chemical Probes. Front. Chem. 2021, 9, 644811 10.3389/fchem.2021.644811. 33937194 PMC8082414

[ref69] Xu H.; Hett E. C.; Gopalsamy A.; Parikh M. D.; Geoghegan K. F.; Kyne R. E. Jr.; Menard C. A.; Narayanan A.; Robinson R. P.; Johnson D. S.; et al. A library approach to rapidly discover photoaffinity probes of the mRNA decapping scavenger enzyme DcpS. Mol. BioSyst. 2015, 11 (10), 2709–2712. 10.1039/C5MB00288E. 25959423

[ref70] deGruyter J. N.; Malins L. R.; Baran P. S. Residue-Specific Peptide Modification: A Chemist’s Guide. Biochemistry 2017, 56 (30), 3863–3873. 10.1021/acs.biochem.7b00536. 28653834 PMC5792174

[ref71] Fallon D. J.; Lehmann S.; Chung C. W.; Phillipou A.; Eberl C.; Fantom K. G. M.; Zappacosta F.; Patel V. K.; Bantscheff M.; Schofield C. J.; et al. One-Step Synthesis of Photoaffinity Probes for Live-Cell MS-Based Proteomics. Chemistry 2021, 27 (71), 17880–17888. 10.1002/chem.202102036. 34328642

[ref72] Weerapana E.; Wang C.; Simon G. M.; Richter F.; Khare S.; Dillon M. B.; Bachovchin D. A.; Mowen K.; Baker D.; Cravatt B. F. Quantitative reactivity profiling predicts functional cysteines in proteomes. Nature 2010, 468 (7325), 790–795. 10.1038/nature09472. 21085121 PMC3058684

[ref73] Zhang T.; Hatcher J. M.; Teng M.; Gray N. S.; Kostic M. Recent Advances in Selective and Irreversible Covalent Ligand Development and Validation. Cell Chem. Biol. 2019, 26 (11), 1486–1500. 10.1016/j.chembiol.2019.09.012. 31631011 PMC6886688

[ref74] Zanon P.; Yu F.; Musacchio P.; Lewald L.; Zollo M.; Krauskopf K.; Mrdović D.; Raunft P.; Maher T.; Cigler M., , Profiling the Proteome-Wide Selectivity of Diverse Electrophiles, ChemRxiv 2021; 10.26434/chemrxiv.14186561. PMC12580327 41168333

[ref75] Wuts P. G.; Greene T. W. Protection for the Amino Group. Greene’s Protective Groups in Organic Synthesis; Wiley, 2006; pp. 696 926.

[ref76] Muñoz-Torrero D.; Mangoni A. A.; Liu H.; Hulme C.; Rautio J.; Karaman R.; De Sousa M. E.; Prokai-Tatrai K.; Sabatier J.-M.; Siciliano C.; et al. Breakthroughs in Medicinal Chemistry: New Targets and Mechanisms, New Drugs, New Hopes–2. Molecules 2018, 23, 65 10.3390/molecules23010065. PMC6017245 29283400

[ref77] Muller S.; Ackloo S.; Al Chawaf A.; Al-Lazikani B.; Antolin A.; Baell J. B.; Beck H.; Beedie S.; Betz U. A. K.; Bezerra G. A.; et al. Target 2035 - update on the quest for a probe for every protein. RSC Med. Chem. 2022, 13 (1), 13–21. 10.1039/D1MD00228G. 35211674 PMC8792830

[ref78] Carter A. J.; Kraemer O.; Zwick M.; Mueller-Fahrnow A.; Arrowsmith C. H.; Edwards A. M. Target 2035: Probing the human proteome. Drug Discovery Today 2019, 24 (11), 2111–2115. 10.1016/j.drudis.2019.06.020. 31278990

[ref79] Hulme C.; Gore V. ″Multi-component reactions: Emerging chemistry in drug discovery″’from xylocain to crixivan’. Curr. Med. Chem. 2003, 10 (1), 51–80. 10.2174/0929867033368600. 12570721

[ref80] Giustiniano M.; Basso A.; Mercalli V.; Massarotti A.; Novellino E.; Tron G. C.; Zhu J. To each his own: Isonitriles for all flavors. Functionalized isocyanides as valuable tools in organic synthesis. Chem. Soc. Rev. 2017, 46 (5), 1295–1357. 10.1039/C6CS00444J. 27983738

