# Supplementary material for: Appendage- and Scaffold-Diverse Electrophilic and Photoreactive Probes for Integrated Phenotypic Screening–Target Identification Campaigns via a Minimalist Bifunctional Isocyanide
Source: ACS Omega. 2024 Oct 2;9(41):42557–70. doi: 10.1021/acsomega.4c06879 (PMC11483914; doi:10.1021/acsomega.4c06879)
Supplement: Supplementary file 1 — ao4c06879_si_001.pdf [file ao4c06879_si_001.pdf]

SUPPORTING INFORMATION

Appendage- and Scaffold-Diverse Electrophilic and Photoreactive Probes for Integrated Phenotypic Screening-Target Identification Campaigns Via a Minimalist Bifunctional Isocyanide

Paul A. Jackson[a§], Eleni Kisty[b§], Vandan Pradhan[a], Christopher Swank[a], Luke Bohrer[a], Tammy L. Nolan[a], Eranthie Weerapana[b*], and David J. Lapinsky[a*]

[§]These authors contributed equally to this work. *Corresponding authors.

[a]Graduate School of Pharmaceutical Sciences, Duquesne University, 600 Forbes Avenue, Pittsburgh, Pennsylvania, 15282, United States
[b]Department of Chemistry, Boston College, Merkert Chemistry Center, 2609 Beacon Street, Chestnut Hill, Massachusetts, 02464, United States

Index

General Experimental Procedures for Organic Synthesis……………………………………………..S3
Preparation of Aldehyde **5**……………………………………………………………………………..S4
Synthetic Procedure and NMR Spectra for 2˚ Amine **6**………………………………………………..S5
Synthetic Procedure and NMR Spectra for Carbamate **7**……………………………………………..S8
Synthetic Procedure and NMR Spectra for 1˚ Amine **8**………………………………………………S11
Synthetic Procedure and NMR Spectra for Formamide **9**……………………………………………S14
Synthetic Procedure and NMR Spectra for Isocyanide **3**……………………………………………S17
Synthetic Scheme, Procedures, and NMR Spectra for FFSMP **4a**…………………..……………..S20
Synthetic Scheme, Procedures, and NMR Spectra for FFSMP (±)-**4b**……………………..……S30
Synthetic Scheme, Procedures, and NMR Spectra for **4c** and **4d**………………………………..S40
Synthetic Scheme, Procedures, and NMR Spectra for FFSMP (±)-**4e**……………………..……S53
Synthetic Scheme, Procedures, and NMR Spectra for FFSMP (±)-**4f**……………………..……S63
Synthetic Scheme, Procedures, and NMR Spectra for FFSMP **4g**…………………...……………..S71
Synthetic Scheme, Procedures, and NMR Spectra for FFSMP (±)-**4h**……………………..……S82
Synthetic Scheme, Procedures, and NMR Spectra for FFSMP (±)-**4i**……………………..……S93
Synthetic Scheme, Procedures, and NMR Spectra for FFSMP **4j**…………………..……………S103
Synthetic Scheme, Procedures, and NMR Spectra for FFSMP (±)-**4k**……………………..……S113

Synthetic Scheme, Procedures, and NMR Spectra for FFSMP (±)-**4l**……..………………..……S123

Synthetic Scheme, Procedures, and NMR Spectra for FFSMP (±)-**4m**...……………….……S133

Synthetic Procedure and NMR Spectra for carbamate (±)-**10**...……………………………..S144

Synthetic Procedure and NMR Spectra for diketopiperazine (±)-**11**....…………………………S147

Synthetic Procedures and NMR Spectra for FFSMP (±)-**4n**....…………………………….……S150

Synthetic Procedure and NMR Spectra for lactam (±)-**13**....……………………………….……S156

Synthetic Procedures and NMR Spectra for FFSMP (±)-**4o**....……………………………….…S159

Synthetic Procedure and NMR Spectra for amide (±)-**14**....……………………………….……S165

Synthetic Procedure and NMR Spectra for benzodiazepine (±)-**15**....…………………………..S168

Synthetic Procedures and NMR Spectra for FFSMP (±)-**4p**....……………………………….…S171

Synthetic Procedure and NMR Spectra for amide (±)-**16**....……………………………….……S174

Synthetic Procedure and NMR Spectra for isoquinoline dione (±)-**17**....………………………..S177

Synthetic Procedures and NMR Spectra for FFSMP (±)-**4q**....……………………………….…S180

Biological Testing of Compounds and Chemoproteomic Experiments…………………………..S186

Chemical Structures of All FFSMPs Produced During This Work……………………………….S192

General Experimental Procedures for Organic Synthesis. Reaction conditions and yields were not optimized. All reactions were performed using oven- or flame-dried glassware fitted with rubber septa under a positive pressure of argon unless otherwise noted. Air- and moisture-sensitive liquids were transferred via syringe or stainless-steel cannula. All solvents and chemicals were purchased from Aldrich Chemical Co. or Fisher Scientific and used without further purification unless otherwise noted. Flash column chromatography was performed as described by Still and co-workers (Rapid chromatographic technique for preparative separations with moderate resolution. *J. Org. Chem.* **1978**, *43*, 2923) using Fisher S826-25 silica gel sorbent (70-230 mesh) and eluting solvent mixtures as specified. Analytical thin-layer chromatography (TLC) was performed using TLC Silica Gel 60 F254 plates obtained from EMD Chemicals. TLC plates were visualized by exposure to ultraviolet light, iodine, and/or submersion in potassium permanganate, para-anisaldehyde, or ninhydrin followed by brief heating with a heat gun (10–15 s). Proportions of solvents used for TLC are by volume. All temperatures reported are bath temperatures. ^{1}H and ^{13}C NMR spectra were recorded on either a Bruker 400 or 500 MHz spectrometer. Chemical shifts for ^{1}H and ^{13}C NMR spectra are reported as parts per million (δ ppm) relative to tetramethylsilane (0.00 ppm) as an internal standard or residual solvent ($CHCl_3$ = 7.26 ppm for ^{1}H NMR, 77.16 ppm for ^{13}C NMR; CD_3OD = 3.31 ppm for ^{1}H NMR, 49.0 ppm for ^{13}C NMR; DMSO = 2.50 ppm for ^{1}H NMR, 39.52 ppm for ^{13}C NMR). Coupling constants are measured in hertz (Hz). HRMS samples were analyzed at Old Dominion University (Norfolk, VA) by positive ion electrospray on a Bruker 12 Tesla APEX-Qe FTICR-MS with an Apollo II ion source. Melting points were not obtained for sticky solids that confounded capillary tube sample preparation for appropriate analysis. On the basis of ^{1}H and ^{13}C NMR spectra, all reported compounds herein were $\geq$95% pure.

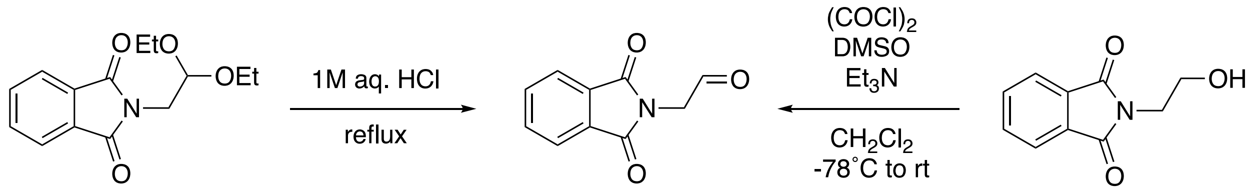

2-(2,2-diethoxyethyl) isoindoline-1,3-dione

5

2-(2-hydroxyethyl) isoindoline-1,3-dione

*2-(1,3-Dioxoisoindolin-2-yl)acetaldehyde (**5**).* Aldehyde **5** was prepared by either:

a.) deprotection of 2-(2,2-diethoxyethyl)isoindoline-1,3-dione according to Welin, E. R.; Warkentin, A. A.; Conrad, J. C.; MacMillan, D. W. C. Enantioselective α-Alkylation of Aldehydes by Photoredox Organocatalysis: Rapid Access to Pharmacophore Fragments from β-Cyanoaldehydes. *Angewandte Chemie International Edition* **2015**, *54* (33), 9668-9672.

or

b.) Swern oxidation of 2-(2-hydroxyethyl)isoindoline-1,3-dione according to Guo, J.; Li, W.; Xue, W.; Ye, X. S. Transition State-Based Sialyltransferase Inhibitors: Mimicking Oxocarbenium Ion by Simple Amide. *J. Med. Chem.* **2017**, *60* (5), 2135-2141.

^{1}H and ^{13}C NMR of aldehyde **5** were in agreement with those previously reported in Veale, E. B.; O'Brien, J. E.; McCabe, T.; Gunnlaugsson, T. The synthesis, N-alkylation and epimerisation study of a phthaloyl derived thiazolidine. *Tetrahedron* **2008**, *64* (28), 6794-6800.

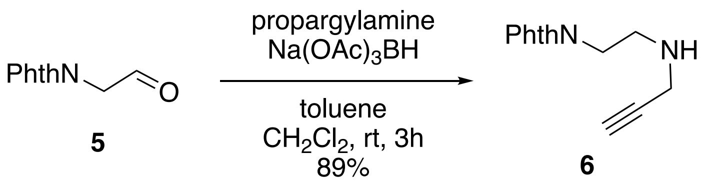

*2-(2-(Prop-2-yn-1-ylamino)ethyl)isoindoline-1,3-dione (**6**).* A flame-dried, 100 mL round-bottom flask equipped with a stirbar, septum, and argon inlet needle was charged with aldehyde **5** (946 mg, 5 mmol, 1 eq.) and CH_2Cl_2 (18 mL). Propargylamine (0.64 mL, 10 mmol, 2 eq.) was then added in a single portion and the mixture was allowed to stir for 10 minutes at room temperature. Next, $Na(OAc)_3BH$ (1.59 g, 7.5 mmol, 1.5 eq.) was added portion-wise over 10 minutes, analogous to that previously described in Abdel-Magid, A. F.; Carson, K. G.; Harris, B. D.; Maryanoff, C. A.; Shah, R. D. Reductive Amination of Aldehydes and Ketones with Sodium Triacetoxyborohydride. Studies on Direct and Indirect Reductive Amination Procedures1. *The Journal of Organic Chemistry* **1996**, *61* (11), 3849-3862. The resulting reaction mixture was then stirred at room temperature for 3 hours, wherein TLC indicated a complete reaction. Next, the reaction mixture was carefully quenched by slow addition of sat. aq. $NaHCO_3$ solution, wherein vigorous gas evolution was observed. The biphasic mixture was stirred until gas evolution ceased (~10 minutes) and then subsequently transferred to a separatory funnel. The layers were separated and the aqueous layer was extracted with EtOAc (3 x 100 mL). The organic layers were combined, dried ($MgSO_4$), filtered, concentrated, and purified by silica gel flash column chromatography (gradient elution with 50-90% EtOAc/hex) to provide secondary amine **6** (1.02 g, 89%) as an orange oil.

R_f = 0.26 (10% MeOH/$CHCl_3$)

^{1}H NMR (400 MHz, $CDCl_3$) δ 7.85-7.83 (m, 2H), 7.72-7.70 (m, 2H), 3.83 (t, J = 6.0 Hz, 2H), 3.45 (d, J = 2.4 Hz, 2H), 3.02 (t, J = 6.0 Hz, 2H), 2.19 (t, J = 2.4 Hz, 1H)

^{13}C NMR (101 MHz, $CDCl_3$) δ 168.7, 134.1, 132.2, 123.4, 82.0, 71.7, 46.5, 37.7, 37.5

HRMS (ESI+) m/z [M + Na] calcd for $C_{13}H_{12}N_2O_2Na$ 229.0972, found: 229.0970

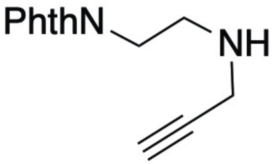

6

^{1}H NMR (400 MHz, $CDCl_3$ with TMS)

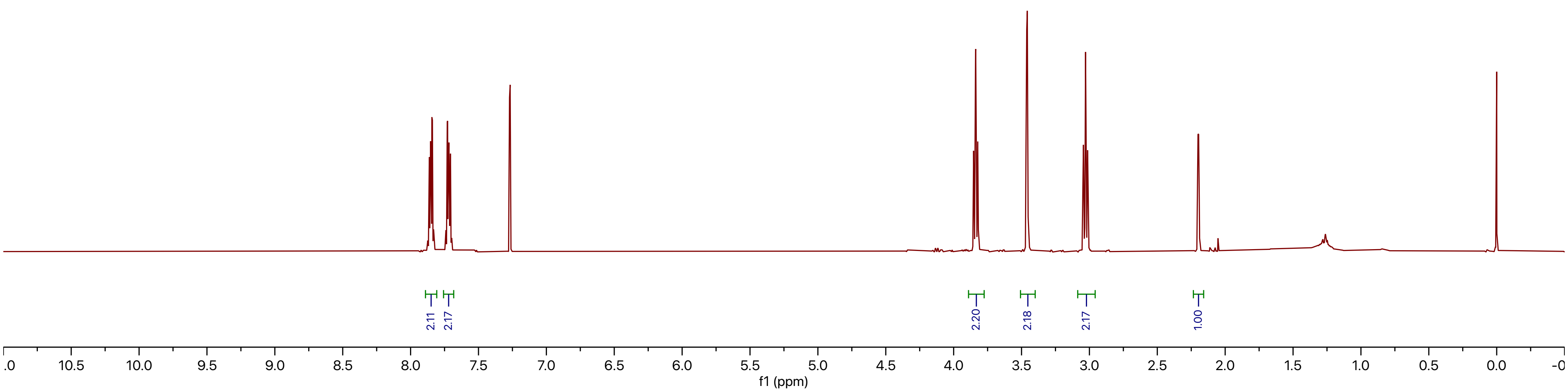

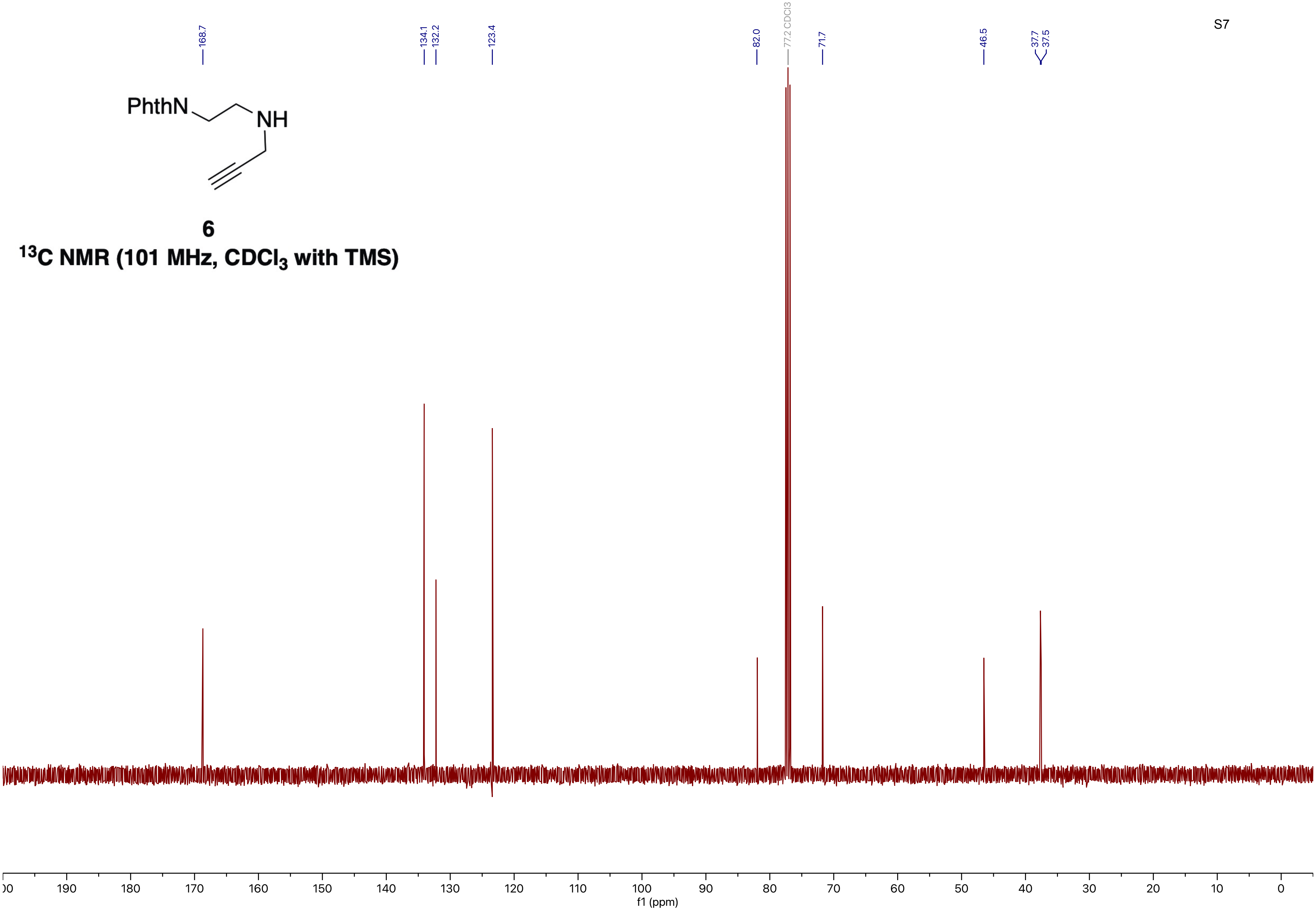

6

^{13}C NMR (101 MHz, $CDCl_3$ with TMS)

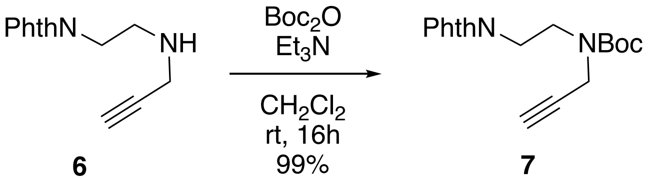

6 7

*tert-Butyl (2-(1,3-dioxoisoindolin-2-yl)ethyl)(prop-2-yn-1-yl)carbamate (**7**).* A flame-dried, 25 mL round-bottom flask equipped with a stirbar, septum, and argon inlet needle was charged with 2° amine **6** (700 mg, 3.1 mmol, 1 eq.) and CH_2Cl_2 (10 mL). Triethylamine (0.6 mL, 4.3 mmol, 1.4 eq.) was added all at once followed by addition of Boc_2O (873 mg, 4.0 mmol, 1.3 eq.) in a single portion. The reaction mixture was then stirred at room temperature for 16 hours then transferred to a separatory funnel. The organic layer was sequentially washed with 1M aq. HCl (30 mL), H_2O (30 mL), saturated aq. $NaHCO_3$ solution (30 mL), H_2O (30 mL), and brine (30 mL). The organic layer was then dried ($MgSO_4$), filtered, concentrated, and purified by silica gel flash column chromatography (elution with 25% EtOAc/hex) to provide carbamate **7** (996 mg, 99%) as a reddish-brown oil.

R_f = 0.50 (50% EtOAc/hexanes)

^{1}H NMR (500 MHz, $CDCl_3$, *peaks reported are for the major rotamer only) δ 7.83-7.82 (m, 2H), 7.72-7.67 (m, 2H), 4.15-4.06 (m, 2H), 3.85 (t, J = 6.0 Hz, 2H), 3.64 (t, J = 6.0 Hz, 2H), 2.11 (app s, 1H), 1.23 (s, 9H)

^{13}C NMR (125 MHz, $CDCl_3$, * peaks reported are for the major rotamer only) δ 168.2, 154.7, 134.2, 132.3, 123.4, 85.3, 79.3, 72.3, 44.2, 44.0, 35.6, 28.1

HRMS (ESI+) m/z [M + Na] calcd for $C_{18}H_{20}N_2O_4Na$ 351.1315, found 351.1316

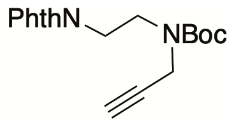

7

^{1}H NMR (500 MHz, $CDCl_3$ with TMS)

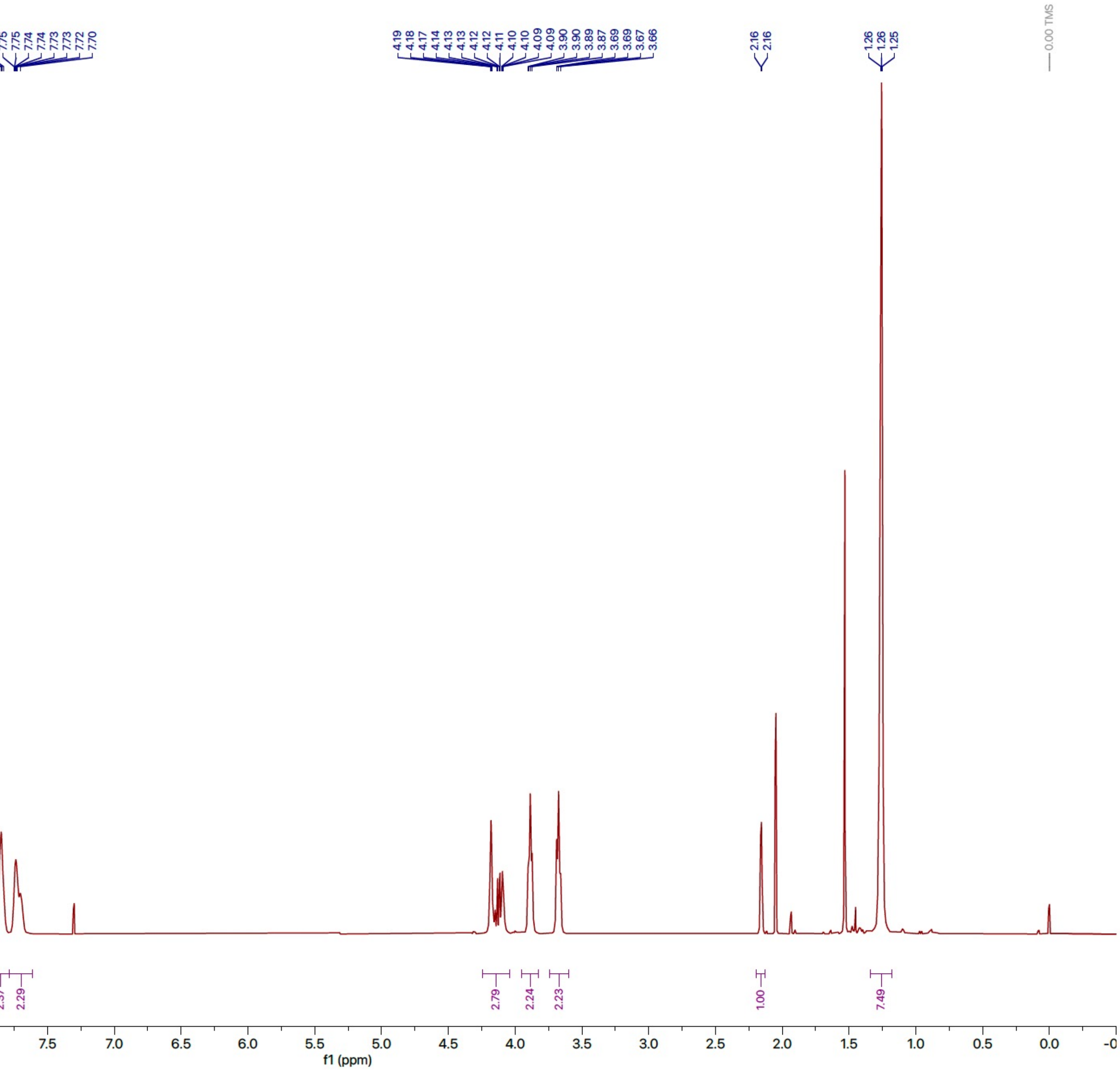

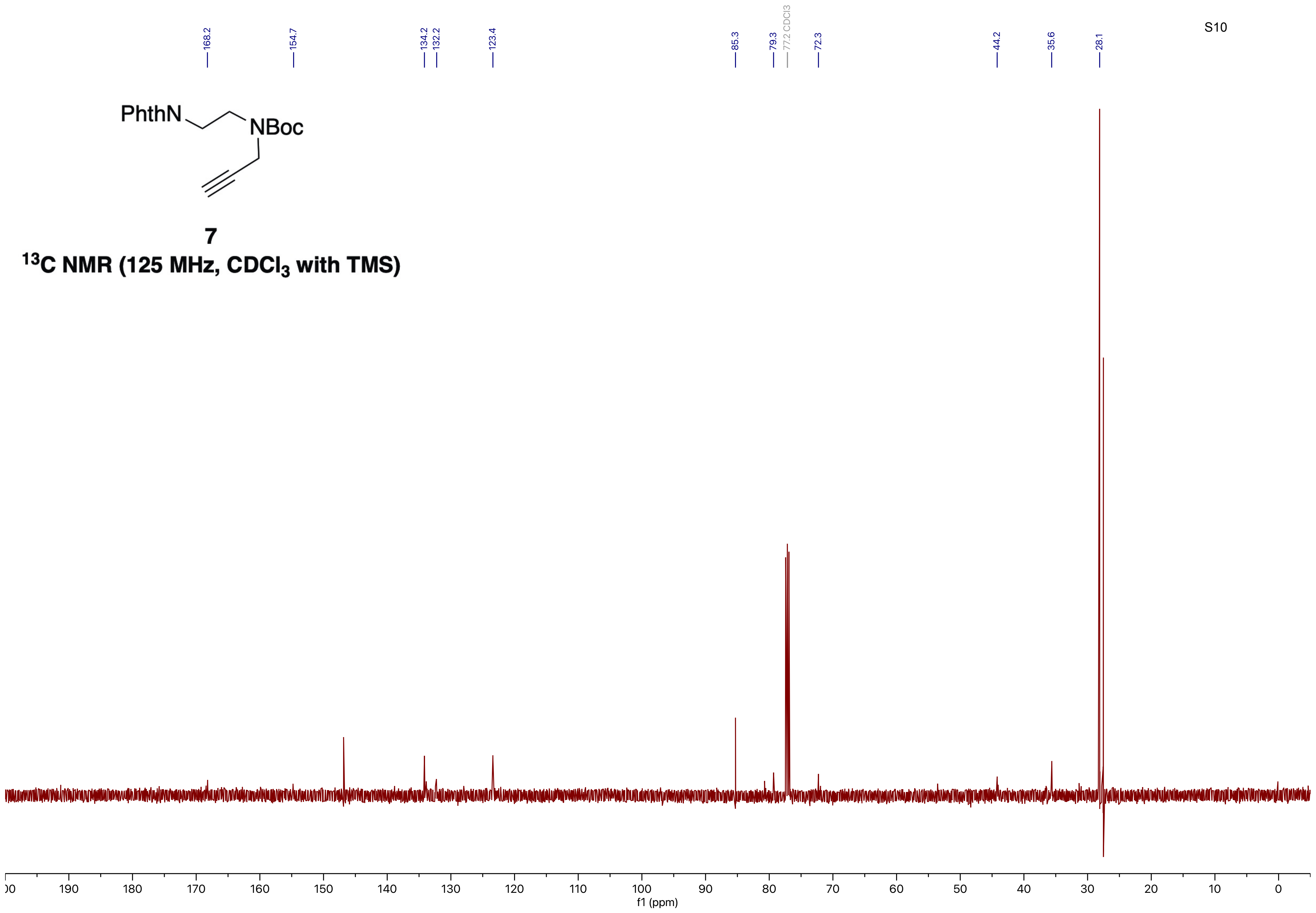

7

^{13}C NMR (125 MHz, $CDCl_3$ with TMS)

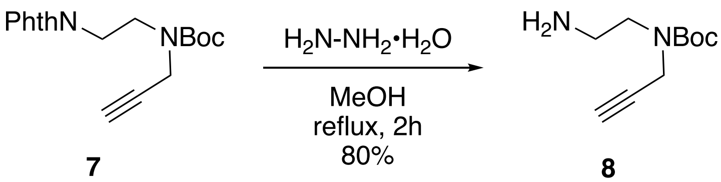

7 **8**

*tert-Butyl (2-aminoethyl)(prop-2-yn-1-yl)carbamate (**8**).* A 250 mL round-bottom flask equipped with a stirbar was charged with phthalimide **7** (6.8 g, 21 mmol, 1 eq.) and MeOH (68 mL). Next, hydrazine monohydrate (64% hydrazine, 2.0 mL, 41 mmol, 2 eq.) was added dropwise over 2 minutes to the phthalimide solution. The resulting reaction mixture was then refluxed at 70°C for 2 hours, wherein subsequent TLC analysis indicated a complete reaction. The reaction mixture was then cooled to room temperature and the white precipitate that formed was removed by vacuum filtration through Celite. The filter cake was then washed with cold MeOH (2 x 50 mL), then the filtrate was concentrated to give a white solid. This solid was then partitioned between H_2O (200 mL) and CH_2Cl_2 (200 mL), the layers were separated, and the aqueous layer was extracted with CH_2Cl_2 (3 x 100 mL). The combined organic layers were then washed with brine (50 mL), dried ($MgSO_4$), filtered, and concentrated to provide 1˚ amine **8** (3.3 g, 80%) as a yellow oil requiring no further purification.

R_f = 0.29 (10% MeOH/$CHCl_3$)

^{1}H NMR (500 MHz, $CDCl_3$, *peaks reported are for the major rotamer only) δ 4.06-3.97 (m, 2H), 3.32 (t, *J* = 6.5 Hz, 2H), 2.83 (tt, *J* = 1.0, 6.5 Hz, 2H), 2.17 (t, *J* = 2.5 Hz, 1H), 1.42 (s, 9H)

^{13}C NMR (125 MHz, $CDCl_3$, *peaks reported are for the major rotamer only) δ 155.2, 80.3, 79.8, 71.3, 50.0, 40.4, 36.9, 28.3

HRMS (ESI+) *m/z* [M + H] calcd for $C_{10}H_{18}N_2O_2H$ 199.1441, found: 199.1442

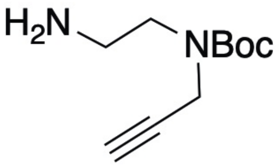

8

^{1}H NMR (500 MHz, $CDCl_3$ with TMS)

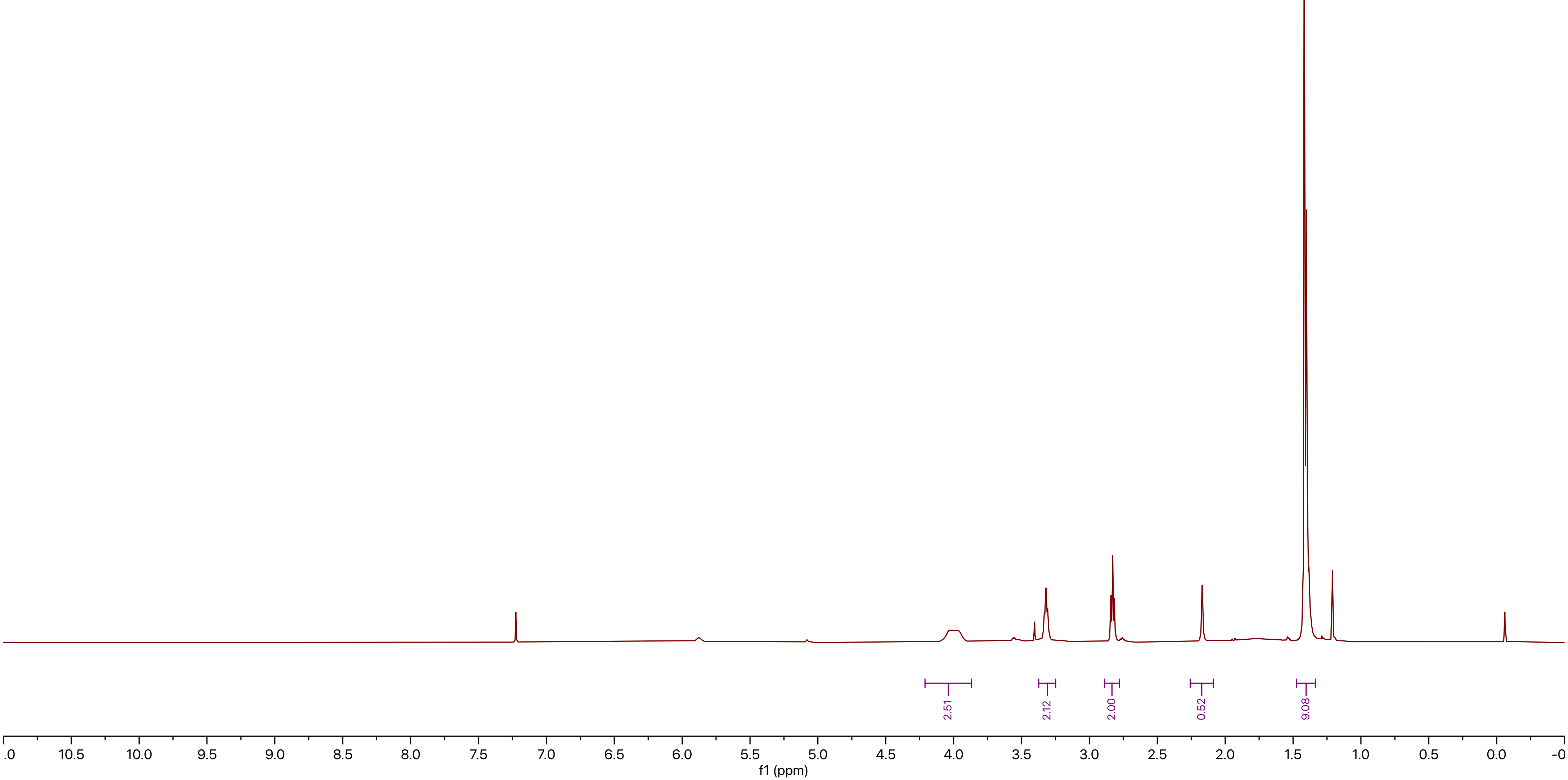

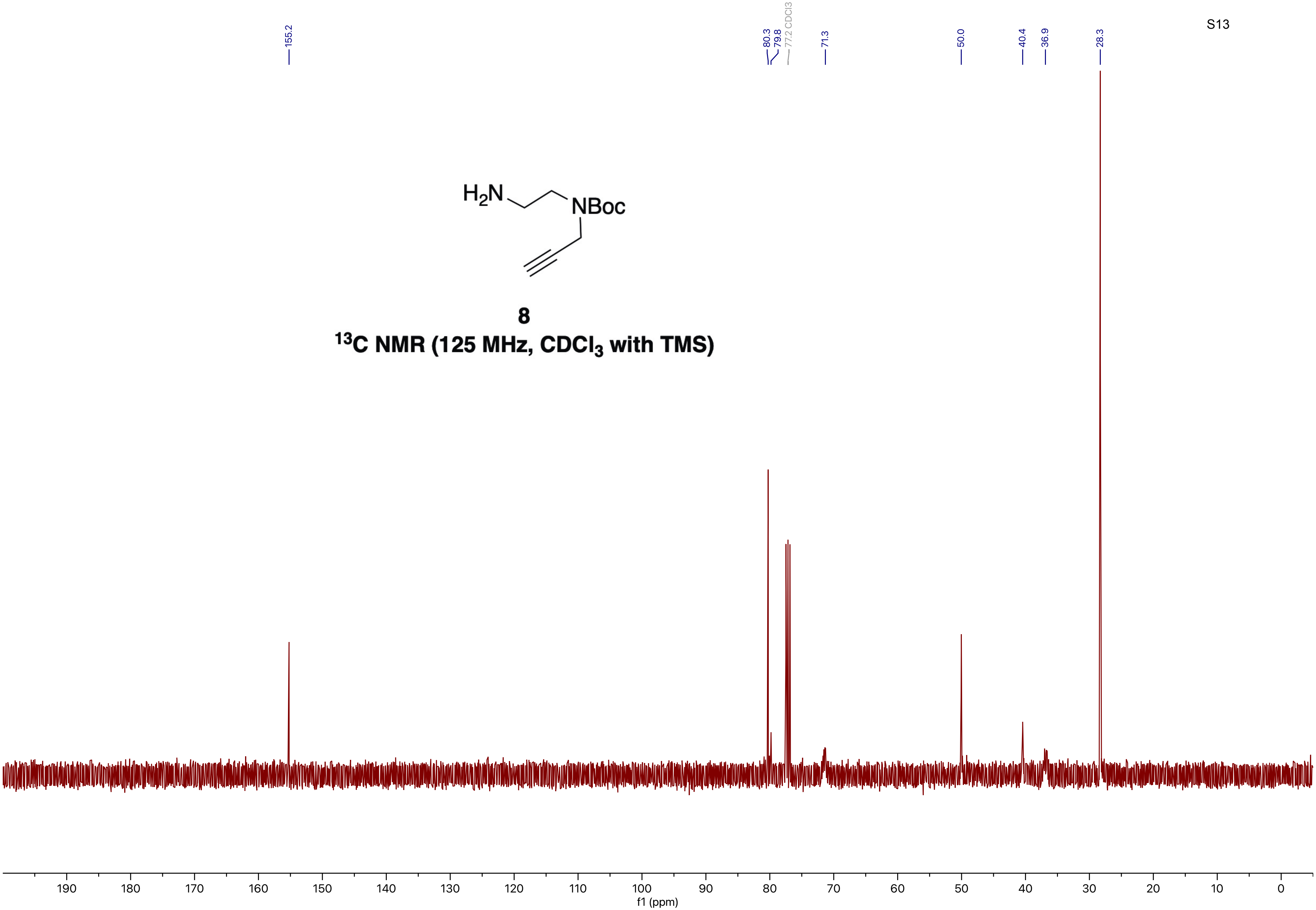
155.2
80.3
79.8
77.2 CDCl3
71.3
50.0
40.4
36.9
28.3
H2N
NBoc
8
13C NMR (125 MHz, CDCl3 with TMS)
190
180
170
160
150
140
130
120
110
100
90
80
70
60
50
40
30
20
10
0
f1 (ppm)

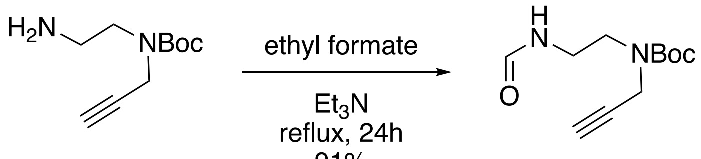

8 ethyl formate, Et_3N, reflux, 24h, 91% **9**

*tert-Butyl (2-formamidoethyl)(prop-2-yn-1-yl)carbamate (**9**).* A 50 mL round-bottom flask equipped with a stirbar was charged with 1° amine **8** (2.96 g, 14.9 mmol, 1 eq.), ethyl formate (12 mL, 149 mmol, 10 eq.), and Et_3N (2.5 mL, 17.9 mmol, 1.2 eq). A reflux condenser was then attached to the reaction flask and capped with an argon inlet adapter. The reaction mixture was then refluxed at 70°C for 24 hours, cooled to room temperature, concentrated under reduced pressure, and then purified by silica gel flash column chromatography (gradient elution with 75-100% EtOAc/hexanes) to provide formamide **9** (2.97 g, 91%) as a pale yellow oil.

R_f = 0.21 (85% EtOAc/hexanes)

^{1}H NMR (500 MHz, $CDCl_3$, *peaks reported are for the major rotamer only) δ 8.05 (bs, 1H), 4.83 (bs, 1H), 4.08-4.06 (m, 2H), 3.43-3.41 (m, 3H), 2.66 (t, *J* = 2.5 Hz, 1H), 1.47 (s, 9H)

^{13}C NMR (101 MHz, $CDCl_3$, *peaks reported are for the major rotamer only) δ 161.6, 155.5, 80.7, 79.3, 71.9, 45.6, 40.2, 37.0, 28.1

HRMS (ESI+) [M + Na] calcd for $C_{11}H_{18}N_2O_3Na$ 249.1210, found: 249.1208.

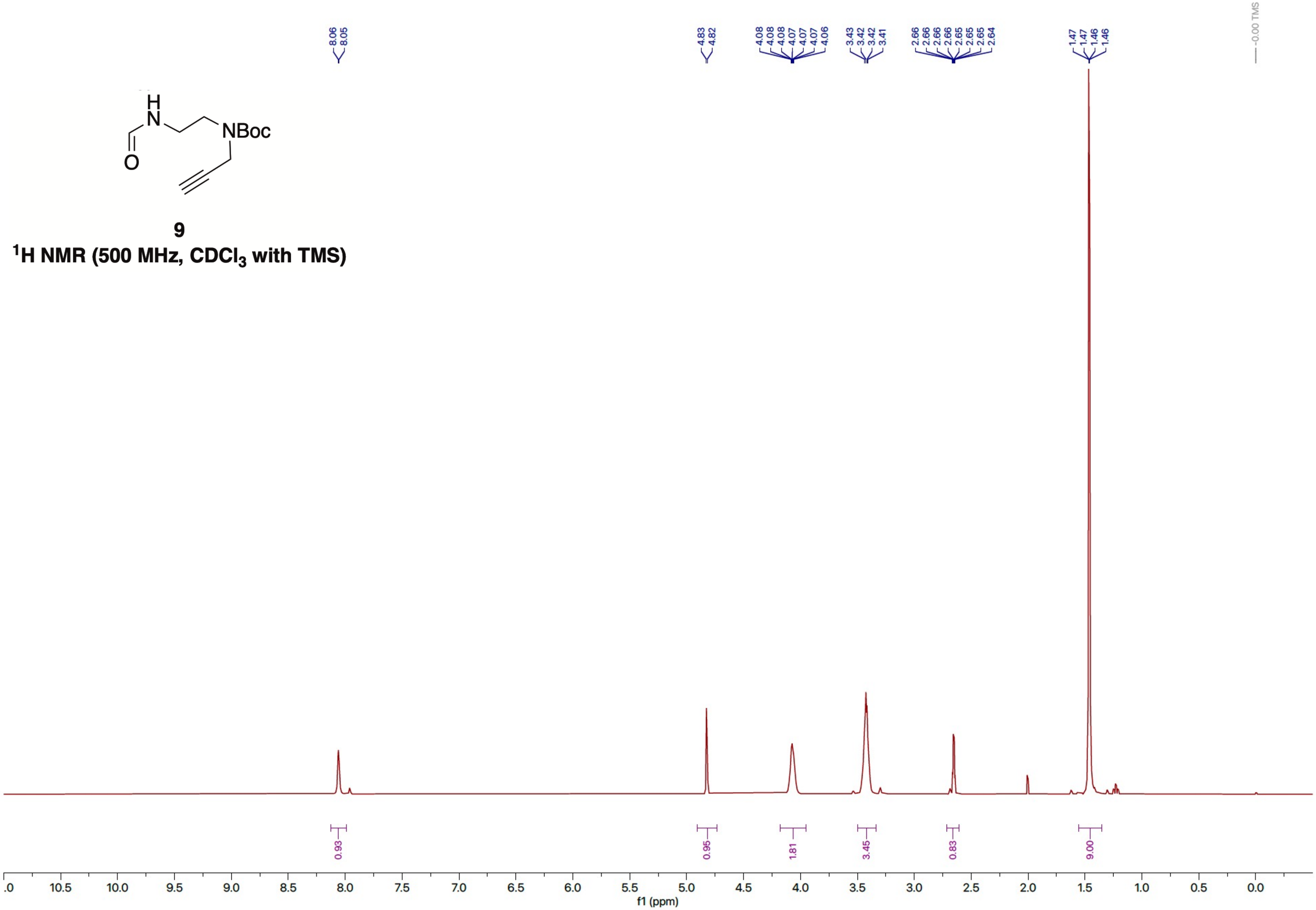

9

^{1}H NMR (500 MHz, $CDCl_3$ with TMS)

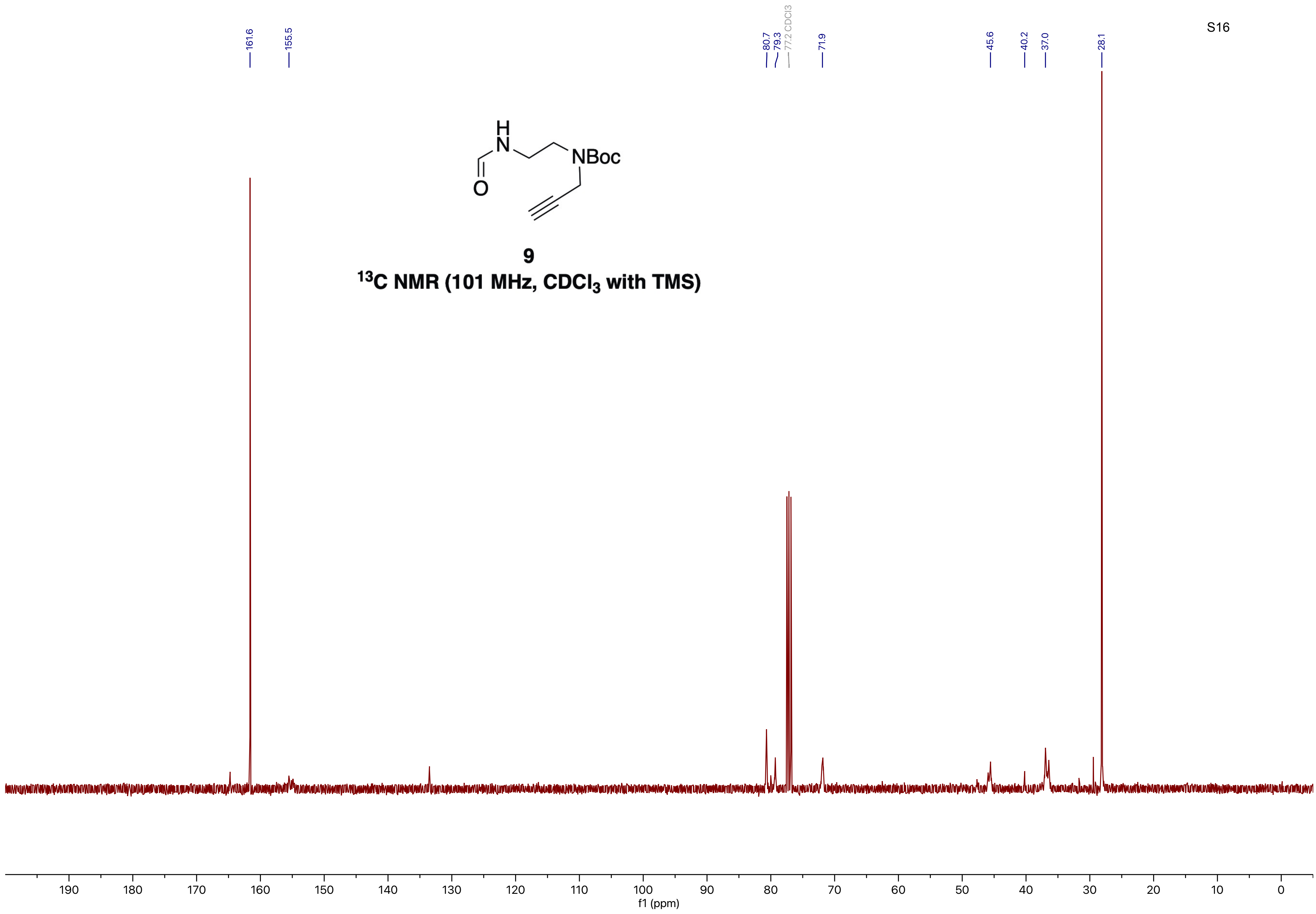

9

^{13}C NMR (101 MHz, $CDCl_3$ with TMS)

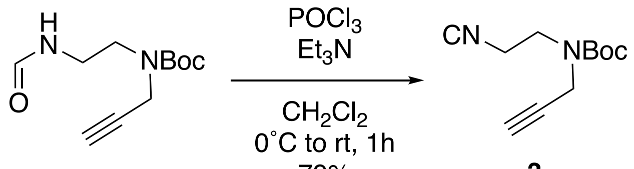

9 3

*tert-Butyl (2-isocyanoethyl)(prop-2-yn-1-yl)carbamate (**3**).* A 250 mL flame-dried, round-bottom flask equipped with a stirbar, septum, and argon inlet needle was charged with formamide **9** (1.87 g, 8.19 mmol, 1 eq.) and CH_2Cl_2 (50 mL, 0.15 M), then cooled to 0°C. Next, Et_3N (2.8 mL, 21 mmol, 2.5 eq.) was added all at once, followed by dropwise addition of $POCl_3$ (0.92 mL, 9.8 mmol, 1.2 eq.) over 1 minute. The ice bath was subsequently removed and the reaction was allowed to warm to room temperature and stirred for 1 hour, wherein TLC analysis indicated a complete reaction. Next, the reaction mixture was cooled to 0°C and slowly quenched by the addition of 10% (w/v) aq. Na_2CO_3 solution (50 mL) over 2 minutes. The ice bath was then removed and the biphasic mixture was allowed to warm to room temperature and stirred for 1.5 hours. Next, the biphasic mixture was diluted with CH_2Cl_2 (15 mL) and H_2O (15 mL), and then transferred to a separatory funnel. The layers were separated and the aqueous layer was extracted with CH_2Cl_2 (2 x 50 mL). The combined organic layers were then washed with brine (15 mL), dried ($MgSO_4$), filtered, concentrated, and purified by silica gel flash column chromatography (elution with 20% EtOAc/hexanes) to provide isocyanide **3** (1.33 g, 79%) as a pale yellow oil.

R_f = 0.80 (50% EtOAc/hexanes)

^{1}H NMR (500 MHz, $CDCl_3$, *peaks reported are for the major rotamer only) δ 4.17-4.12 (m, 2H), 3.62-3.61 (m, 4H), 2.29 (t, J = 2.5 Hz, 1H), 1.49 (s, 9H)

^{13}C NMR (125 MHz, $CDCl_3$, *peaks reported are for the major rotamer only) δ 158.2, 154.6, 81.4, 78.9, 72.7, 45.6, 40.1, 38.1, 28.3

HRMS (ESI+) [M + Na] calcd for $C_{11}H_{16}N_2O_2Na$ 231.1104, found 231.1105

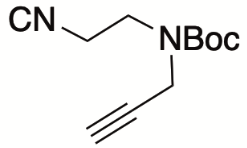

3

^{1}H NMR (500 MHz, $CDCl_3$ with TMS)

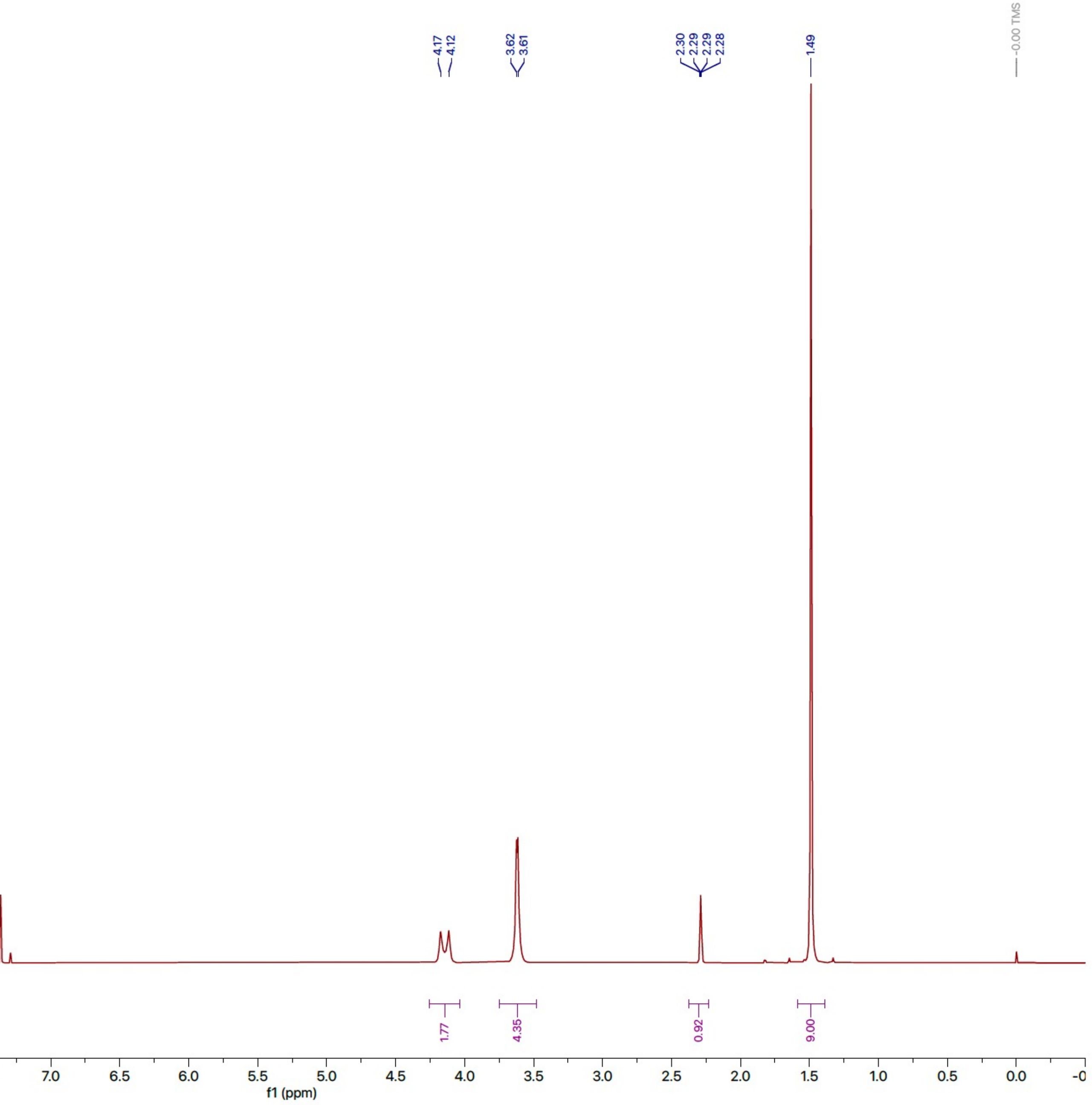

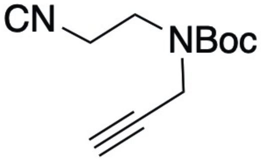

3

^{13}C NMR (125 MHz, $CDCl_3$ with TMS)

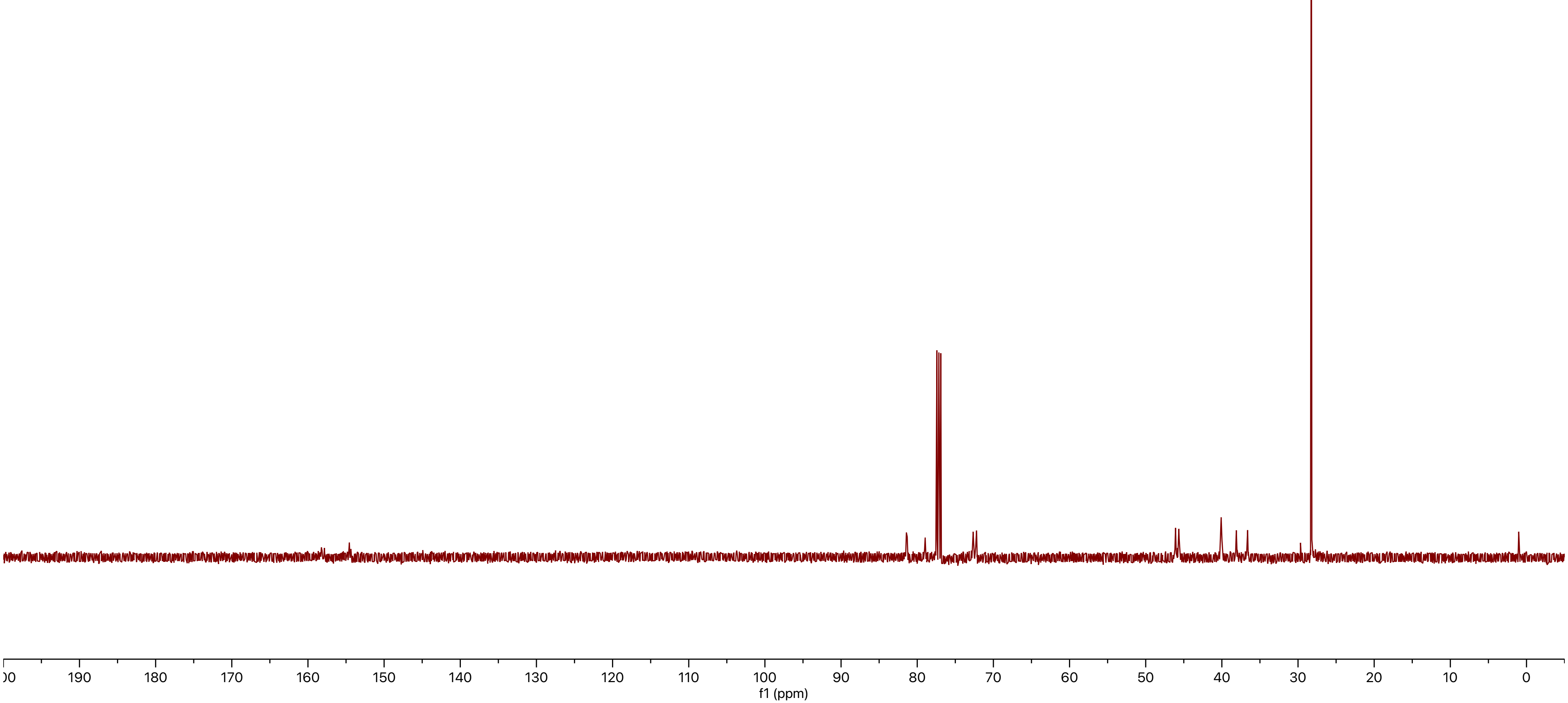

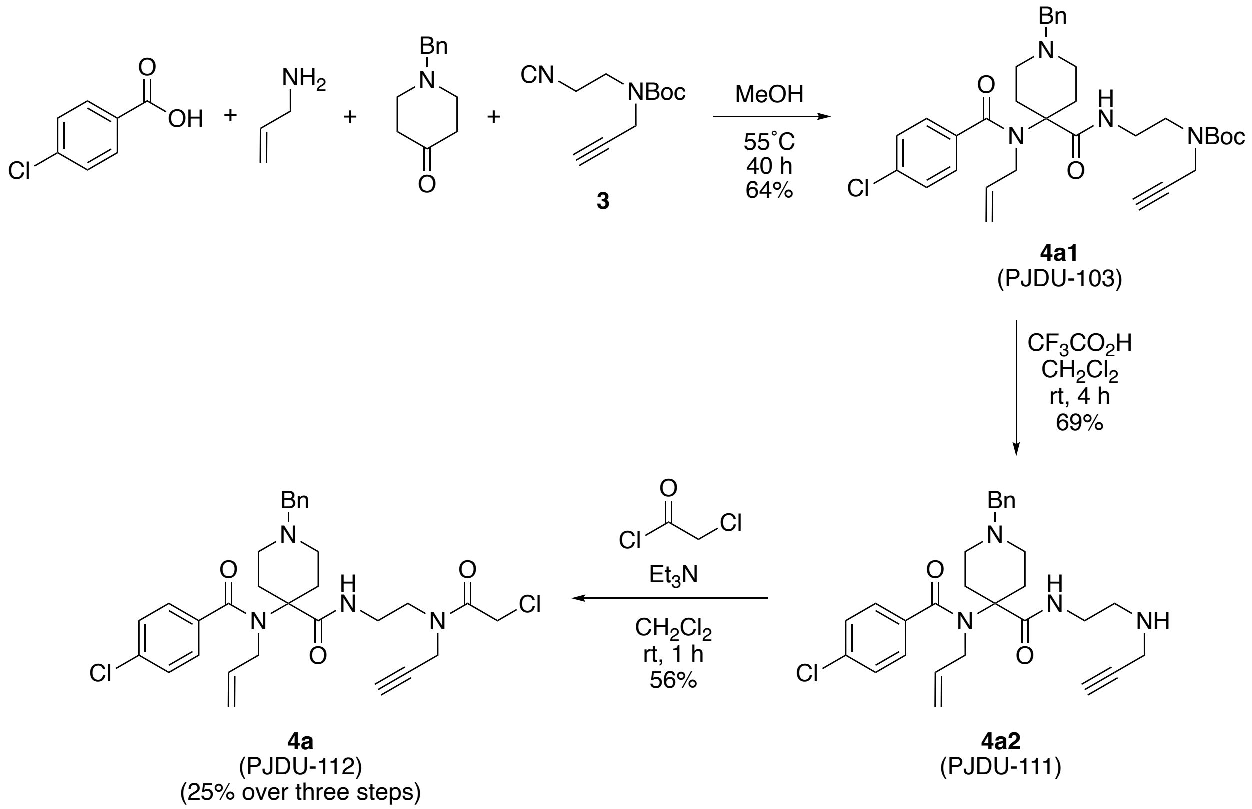

Scheme S1. *Synthesis of U-4CR-based FFSMP **4a** (PJDU-112).*

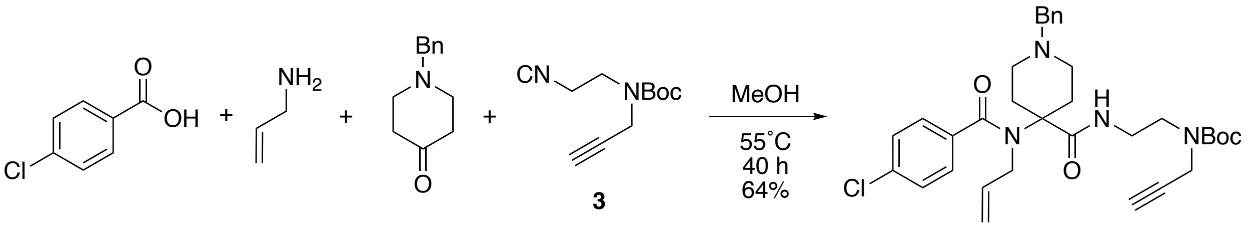

4a1
(PJDU-103)

*tert-Butyl (2-(4-(N-allyl-4-chlorobenzamido)-1-benzylpiperidine-4-carboxamido)ethyl)(prop-2-yn-1-yl)carbamate (**4a1**) (PJDU-103).* 1-Benzyl-4-piperidone (0.09 mL, 0.5 mmol, 1.05 eq.) was initially dissolved in MeOH (1mL, 0.5 M) and treated with Allylamine (0.06 mL, 0.7 mmol, 1.4 eq.). The reaction mixture was then stirred at room temperature for 15 minutes to allow the presumed imine to form. Next, 4-chlorobenzoic acid (78 mg, 0.5 mmol, 1.05 eq.) was added, followed immediately by the addition of isocyanide **3** (99 mg, 0.47 mmol, 1 eq.) as a solution in CH_2Cl_2 (0.5 mL). The mixture was then stirred at 55°C for 40 hours wherein TLC analysis indicated a complete reaction. The reaction mixture was then concentrated, dissolved in EtOAc, and then washed sequentially with sat. aq. $NaHCO_3$ solution, and brine. U-4CR adduct **4a1** (179 mg, 64%, PJDU-103) was obtained as a white sticky solid after silica gel column chromatography (gradient elution with 50-90% EtOAc/hexanes, then 100% EtOAc).

R_f = 0.12 (85% EtOAc/hexanes)

^{1}H NMR (500 MHz, $CDCl_3$, *peaks reported are for the major rotamer only) δ 7.51 (bs, 1H), 7.33 (app s, 4H), 7.29-7.27 (m, 4H), 7.24-7.23 (m, 1H), 5.80-5.74 (m, 1H), 5.04 (dd, *J* = 10.5, 1.0 Hz, 1H), 4.95 (d, *J* = 17.5 Hz, 1H), 4.06 (app bs, 2H), 3.93 (d, *J* = 6.0 Hz, 2H), 3.53 (s, 2H), 3.47 (app d, 4H), 2.59-2.51 (m, 4H), 2.46-2.43 (m, 2H), 2.31-2.25 (m, 2H), 2.22 (t, *J* = 2.5 Hz, 1H), 1.47 (s, 9H)

^{13}C NMR (125 MHz, $CDCl_3$, *peaks reported are for the major rotamer only) δ 174.1, 173.6, 158.8 155.4, 138.2, 136.3, 134.9, 129.2, 128.9, 128.8, 128.2, 127.1, 117.8, 80.7, 79.7, 71.7, 64.3, 62.9, 52.9, 50.4, 49.9, 46.2, 41.4, 38.7, 37.1, 32.6, 28.5

HRMS (ESI+) *m/z* [M + H] calcd for $C_{33}H_{42}ClN_4O_4$ 593.2889, found 593.2890

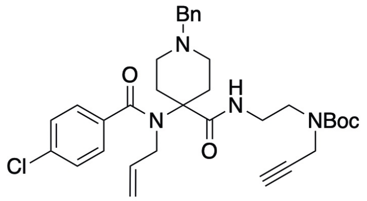

4a1
(PJDU-103)
^{1}H NMR (500 MHz, $CDCl_3$ with TMS)

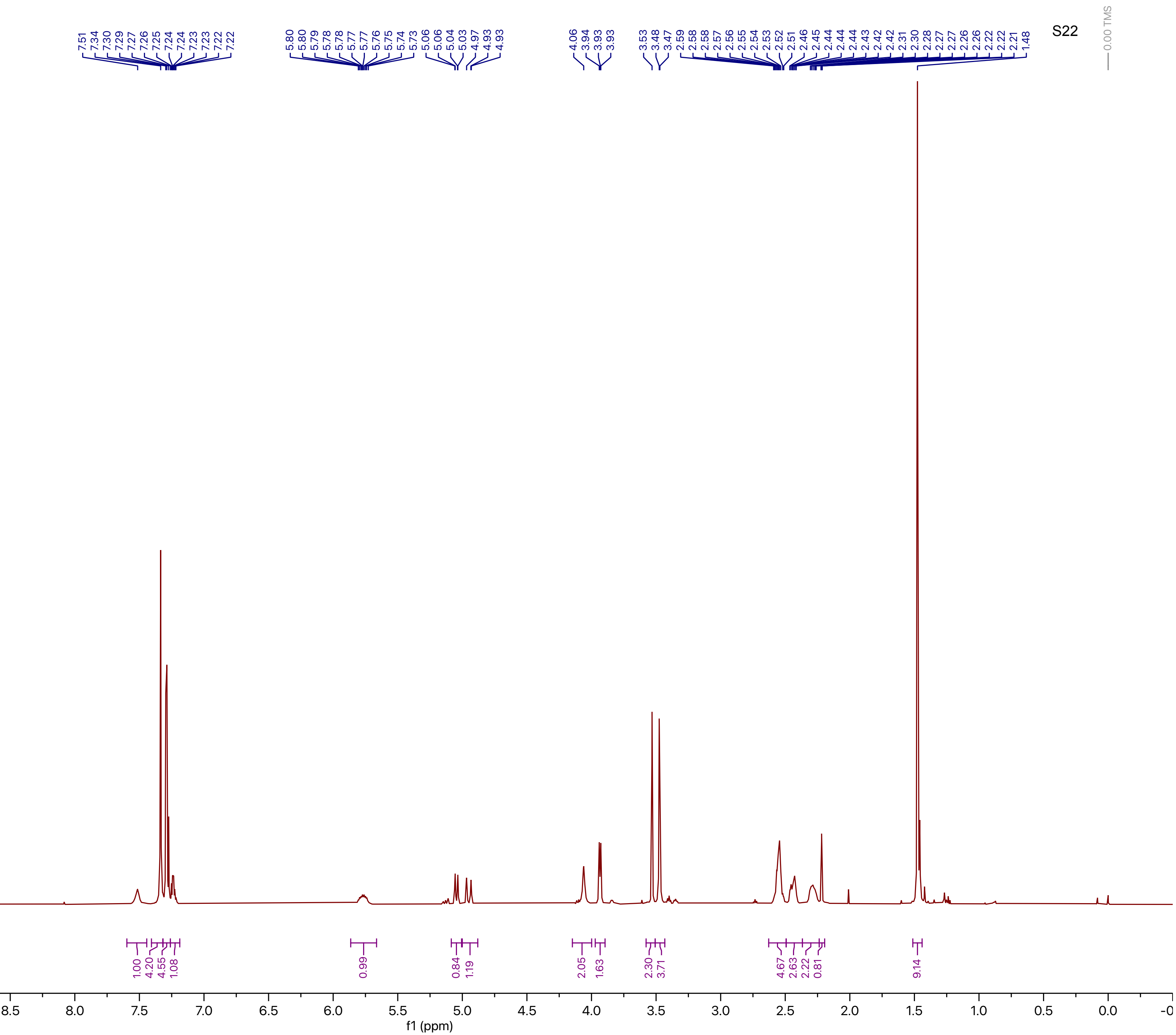

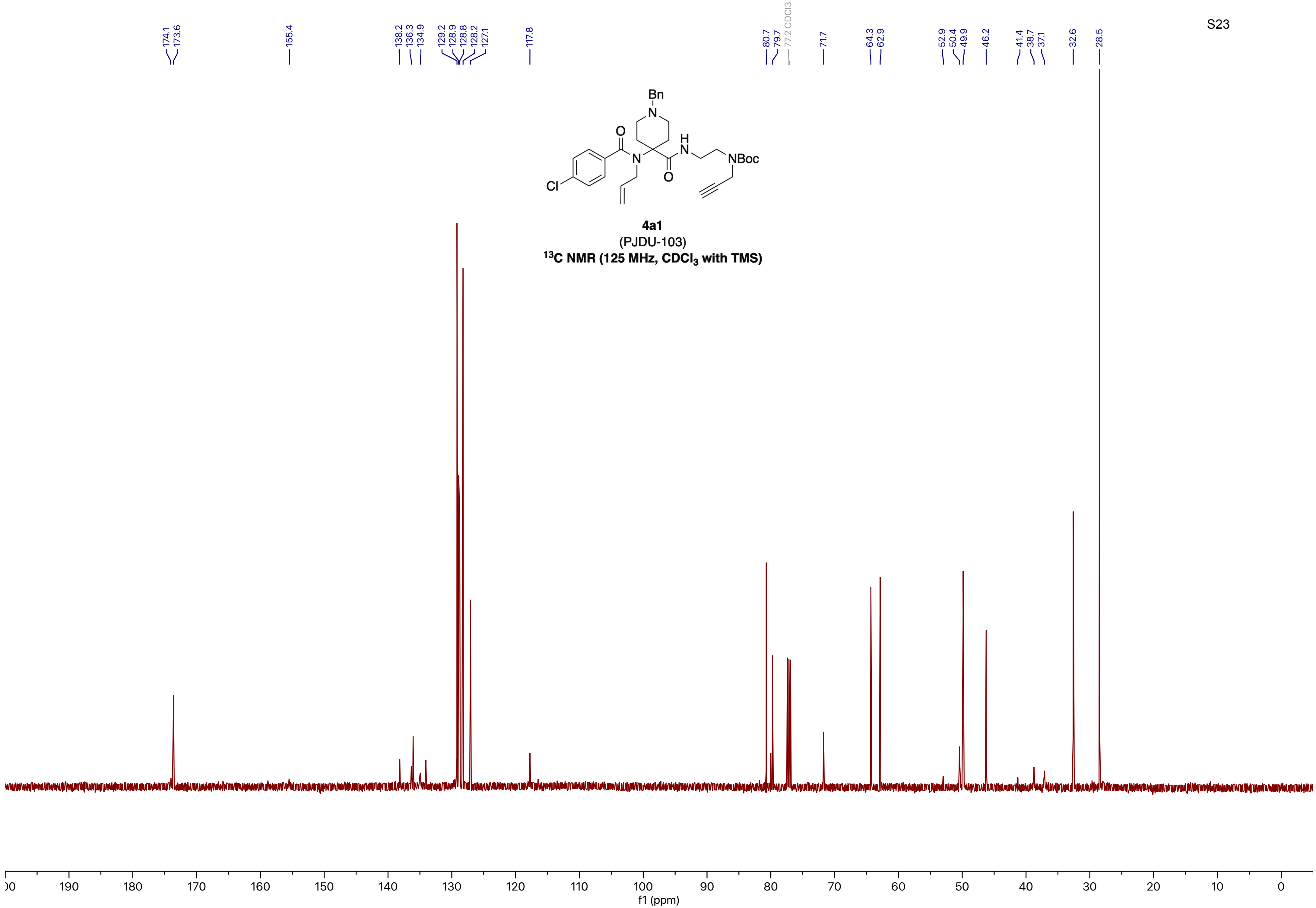

4a1
(PJDU-103)
^{13}C NMR (125 MHz, $CDCl_3$ with TMS)

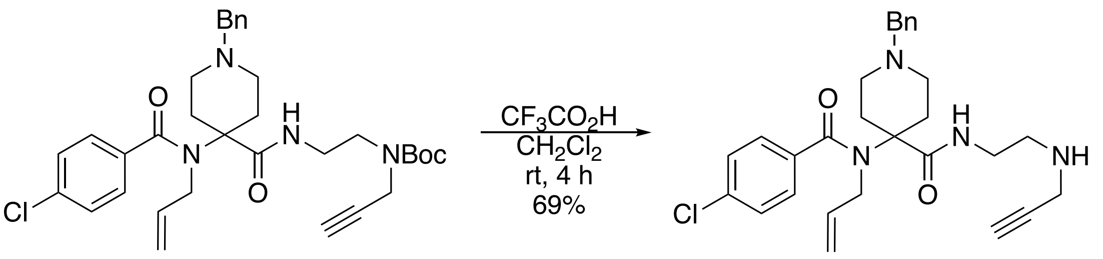

4a1 (PJDU-103) **4a2** (PJDU-111)

*4-(N-Allyl-4-chlorobenzamido)-1-benzyl-N-(2-(prop-2-yn-1-ylamino)ethyl)piperidine-4-carboxamide (**4a2**) (PJDU-111).* A flame-dried, round-bottom flask equipped with a stirbar, septum, and argon inlet needle was charged with Boc carbamate **4a1** (123 mg, 0.21 mmol, 1 eq.) and CH_2Cl_2 (2 mL, 0.1 M), then cooled to 0°C using a saturated ice-water bath. Trifluoroacetic acid (0.16 mL, 2.1 mmol, 10 eq.) was then added dropwise over 2 minutes. After the addition, the ice bath was removed and the reaction was allowed to warm to room temperature and stirred for 4 hours wherein TLC indicated complete consumption of the starting Boc carbamate substrate. The reaction was then quenched by dropwise addition of sat. aq. $NaHCO_3$ solution (~10 mL, vigorous gas evolution) until gas evolution ceased, then 1M aq. NaOH (~4 mL) was added to raise the pH to 8-9. The biphasic mixture was then transferred to a separatory funnel and diluted with CH_2Cl_2, the layers were separated, and the aqueous layer was extracted with CH_2Cl_2. The combined organic layers were then washed with brine, dried ($MgSO_4$), filtered, and concentrated to dryness to provide propargyl amine **4a2** (70 mg, 69%, PJDU-111) as a pale yellow oil requiring no further purification.

R_f = 0.05 (75% EtOAc/hexanes)

^{1}H NMR (500 MHz, $CDCl_3$, *peaks reported are for the major rotamer only) δ 7.43 (t, J = 5.2 Hz, 1H), 7.34-7.25 (m, 9H), 5.76 (qt, J = 6.0, 10.5, 17.5 Hz, 1H), 5.07 (dd, J = 1.0, 10.5 Hz, 1H), 5.00 (dd, J = 1, 17.5 Hz, 1H), 3.93-3.92 (m, 2H), 3.54 (s, 2H), 3.45 (s, 1H), 3.39 (d, J = 2.5 Hz, 2H), 3.37 (q, J = 6.0 Hz, 2H), 2.83 (t, J = 6.0 Hz, 2H), 2.57-2.55 (m, 4H), 2.48-2.43 (m, 2H), 2.29-2.22 (m, 2H), 2.19 (t, J = 2.5 Hz, 1H)

^{13}C NMR (125 MHz, $CDCl_3$, *peaks reported are for the major rotamer only) δ 174.1, 173.5, 137.7, 136.3, 135.8, 134.8, 129.3, 128.8, 128.7, 128.3, 127.2, 118.1, 82.1, 71.6, 64.2, 64.0, 62.9, 50.4, 49.8, 47.4, 39.5, 37.8, 32.4, 31.0

HRMS (ESI+) m/z [M + H] calcd for $C_{28}H_{33}ClN_4O_2H$ 493.2365, found 493.2362

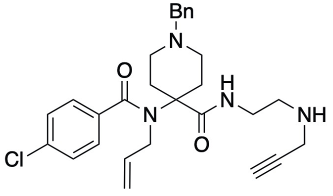

4a2
(PJDU-111)
^{1}H NMR (500 MHz, CDCl$_3$ with TMS)

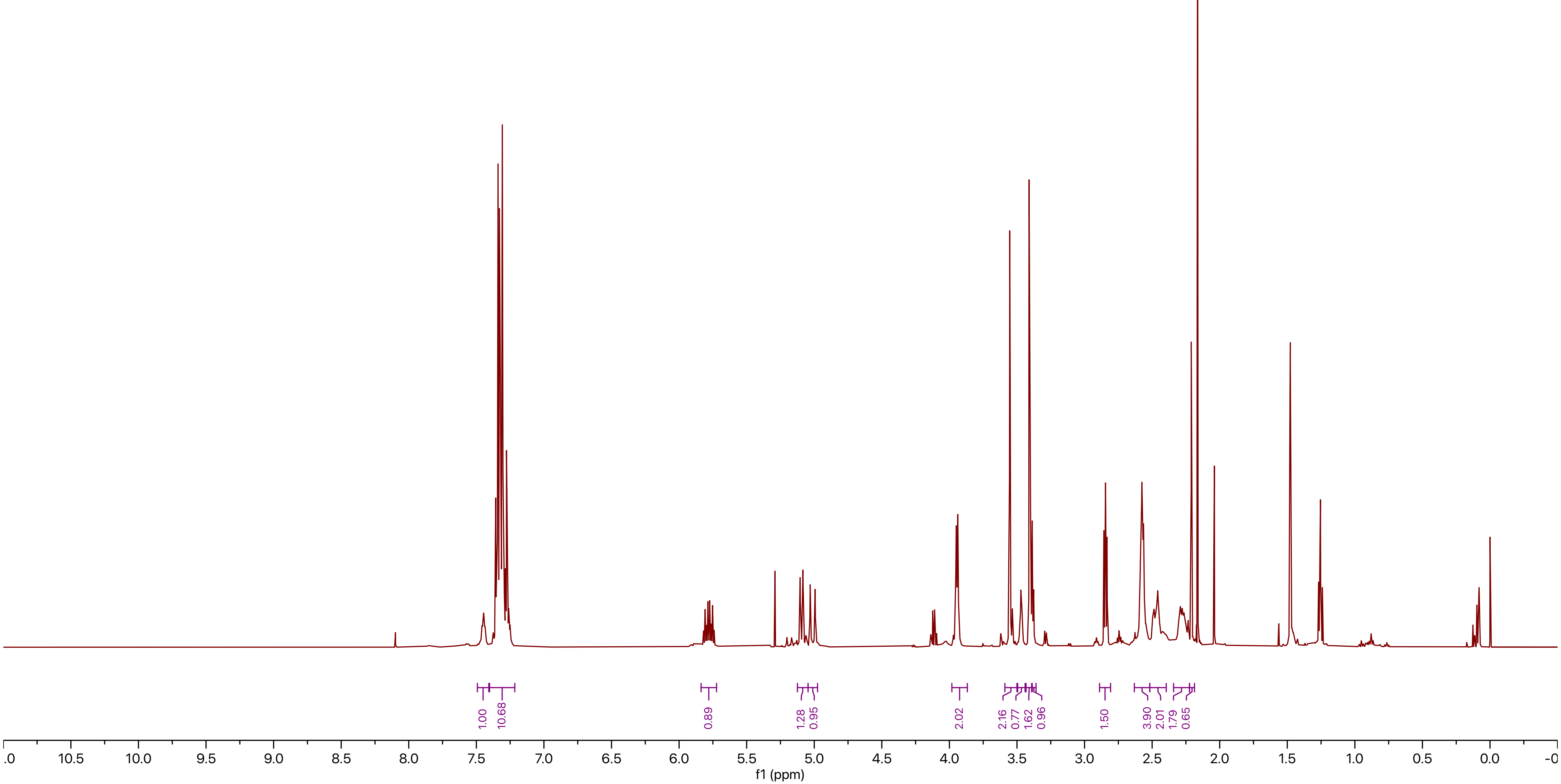

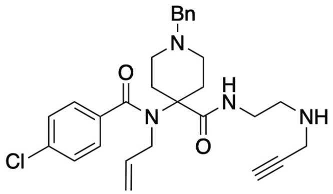

4a2
(PJDU-111)
^{13}C NMR (125 MHz, $CDCl_3$ with TMS)

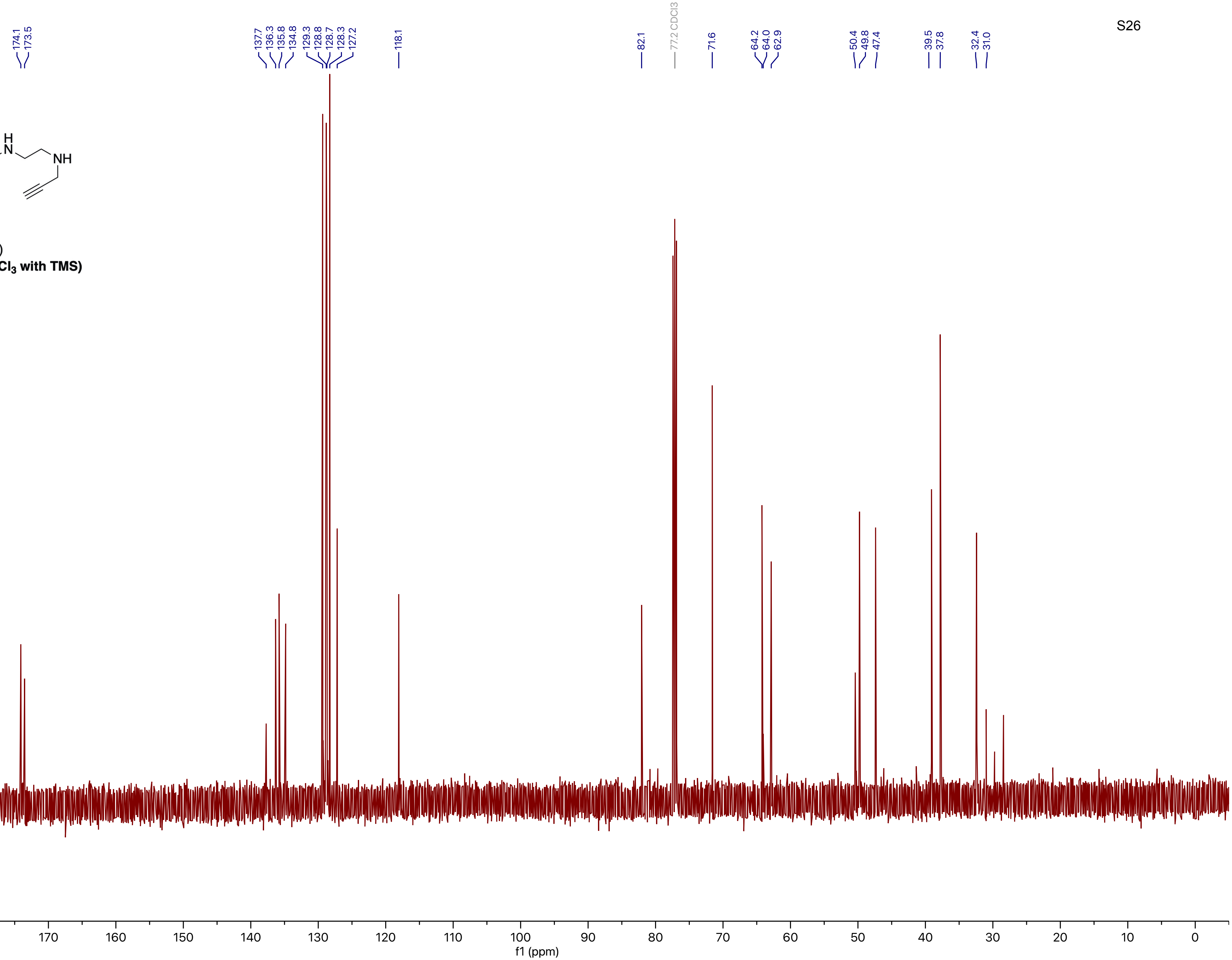

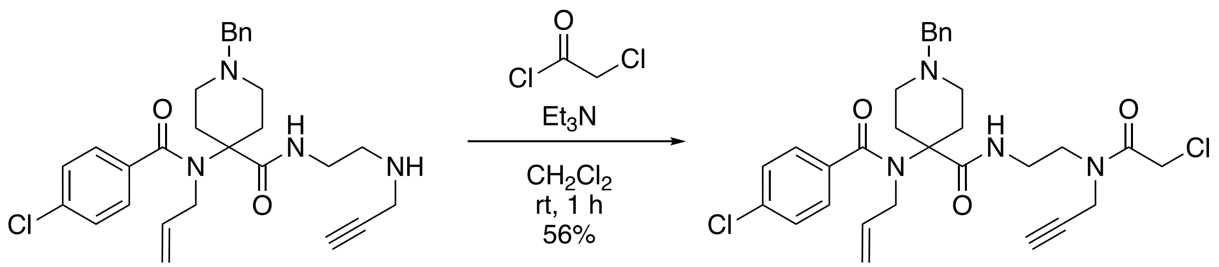

4a2
(PJDU-111)

4a
(PJDU-112)

*4-(N-Allyl-4-chlorobenzamido)-1-benzyl-N-(2-(2-chloro-N-(prop-2-yn-1-yl)acetamido)ethyl)piperidine-4-carboxamide (**4a**) (PJDU-112).* A flame-dried, round-bottom flask equipped with a stirbar, septum, and argon inlet needle was charged with the 2˚ propargyl amine **4a2** (70 mg, 0.14 mmol, 1 eq.; PJDU-111) and CH_2Cl_2 (1.5 mL), then cooled to 0°C with a saturated ice-water bath. Triethylamine (0.04 mL, 0.28 mmol, 2 eq.) was then added dropwise over 1 minute, followed by dropwise addition of chloroacetyl chloride (0.015 mL, 0.17 mmol, 1.2 eq.) over 1 minute. The ice bath was then removed and the reaction mixture was allowed to warm to room temperature. The reaction mixture was stirred for 1 hour wherein complete consumption of the starting 2˚ propargyl amine substrate was observed by TLC. Next, the reaction mixture was transferred to a separatory funnel and washed with H_2O. The aq. layer was then extracted with CH_2Cl_2. The combined organic layers were then washed with sat. aq. $NaHCO_3$ solution, dried ($MgSO_4$), filtered, and concentrated to produce a crude residue that was purified by silica gel flash column chromatography (gradient elution with 3-10% MeOH/$CHCl_3$) to provide 45 mg of FFSMP **4a** (56%, PJDU-112) as a pale yellow oil.

R_f = 0.68 (20% MeOH/$CHCl_3$)

^{1}H NMR (500 MHz, $CDCl_3$, *peaks reported are for the major rotamer only) δ 7.45-7.35 (m, 10H), 5.99-5.91 (m, 1H), 5.23-5.17 (m, 2H), 4.24-4.20 (m, 1H), 4.13-4.12 (m, 1H), 4.10 (s, 2H), 4.06 (s, 2H), 4.00-3.97 (m, 2H), 3.66-3.54 (m, 2H), 3.51-3.44 (m, 2H), 3.36-3.30 (m, 4H), 2.57-2.54 (m, 2H), 2.50-2.45 (m, 2H), 2.39 (t, J = 2.5 Hz, 1H)

^{13}C NMR (125 MHz, $CDCl_3$, *peaks reported are for the major rotamer only) δ 174.0, 173.8, 167.9, 136.9, 136.3, 135.1, 129.5, 129.1, 128.8, 128.8, 128.5, 128.4, 118.1, 78.8, 73.9, 72.8, 50.1, 49.7, 47.0, 46.0, 41.5, 38.0, 35.2, 32.2, 31.4, 28.6

HRMS (ESI+) m/z [M + H] calcd for $C_{30}H_{34}Cl_2N_4O_3H$ 569.2081, found 569.2092

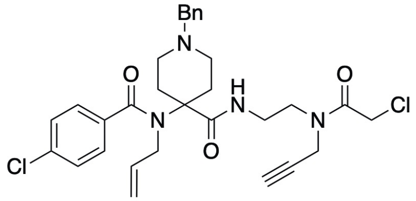

4a
(PJDU-112)
^{1}H NMR (500 MHz, $CDCl_3$ with TMS)

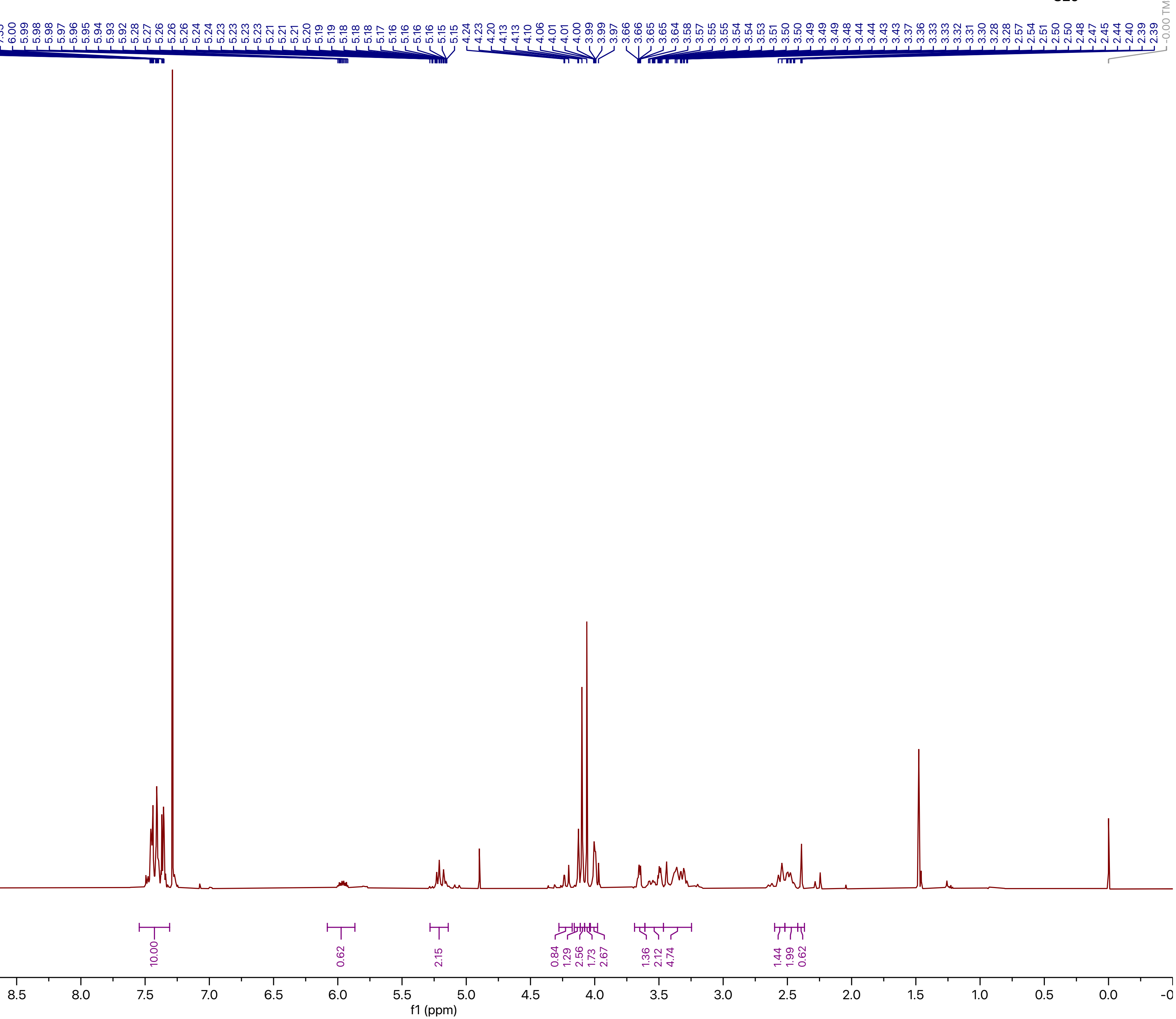

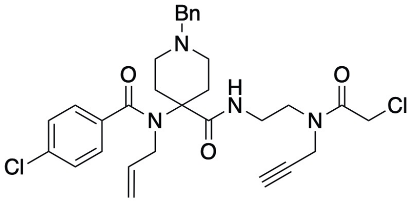

4a
(PJDU-112)
^{13}C NMR (125 MHz, $CDCl_3$ with TMS)

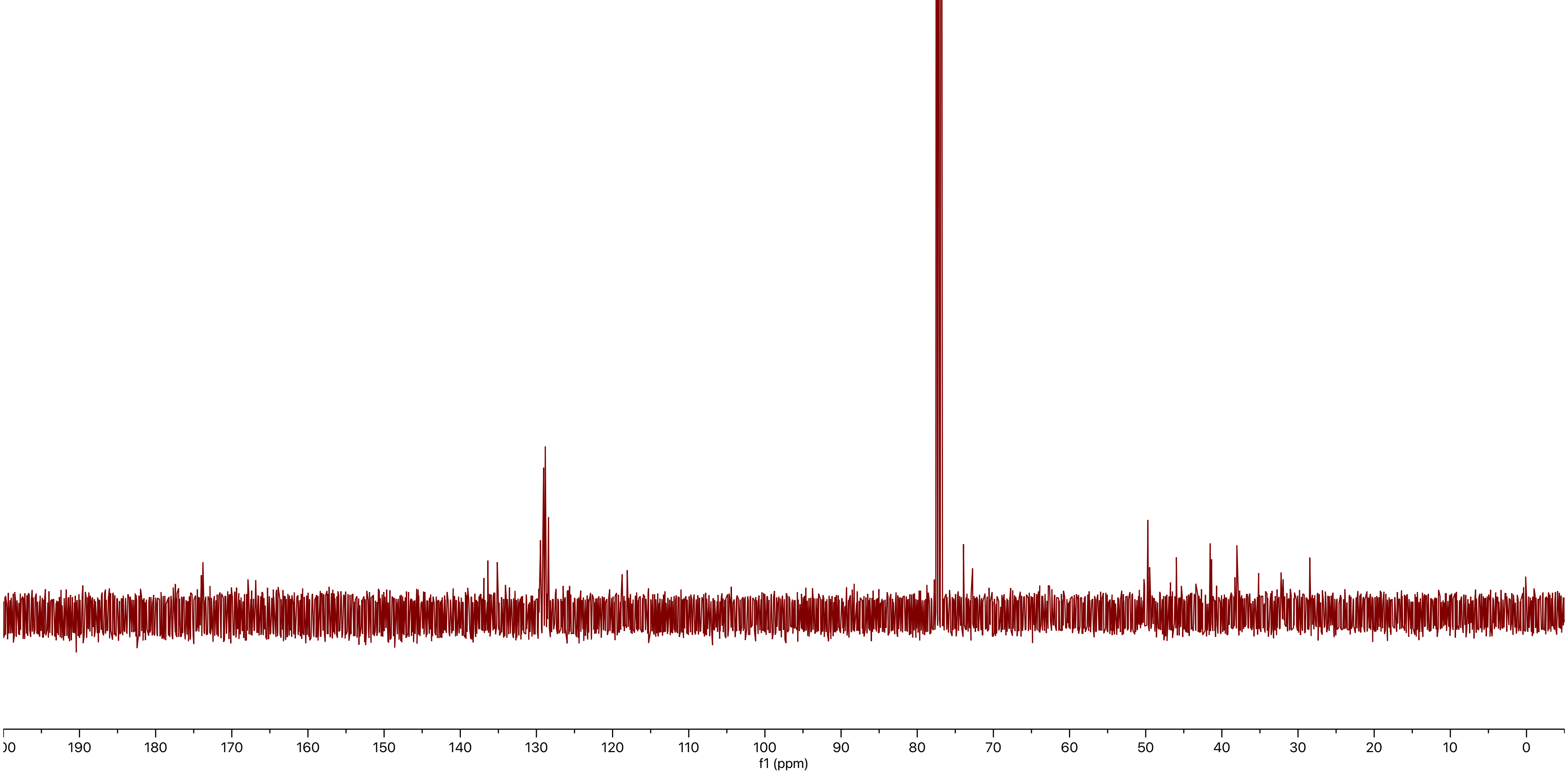

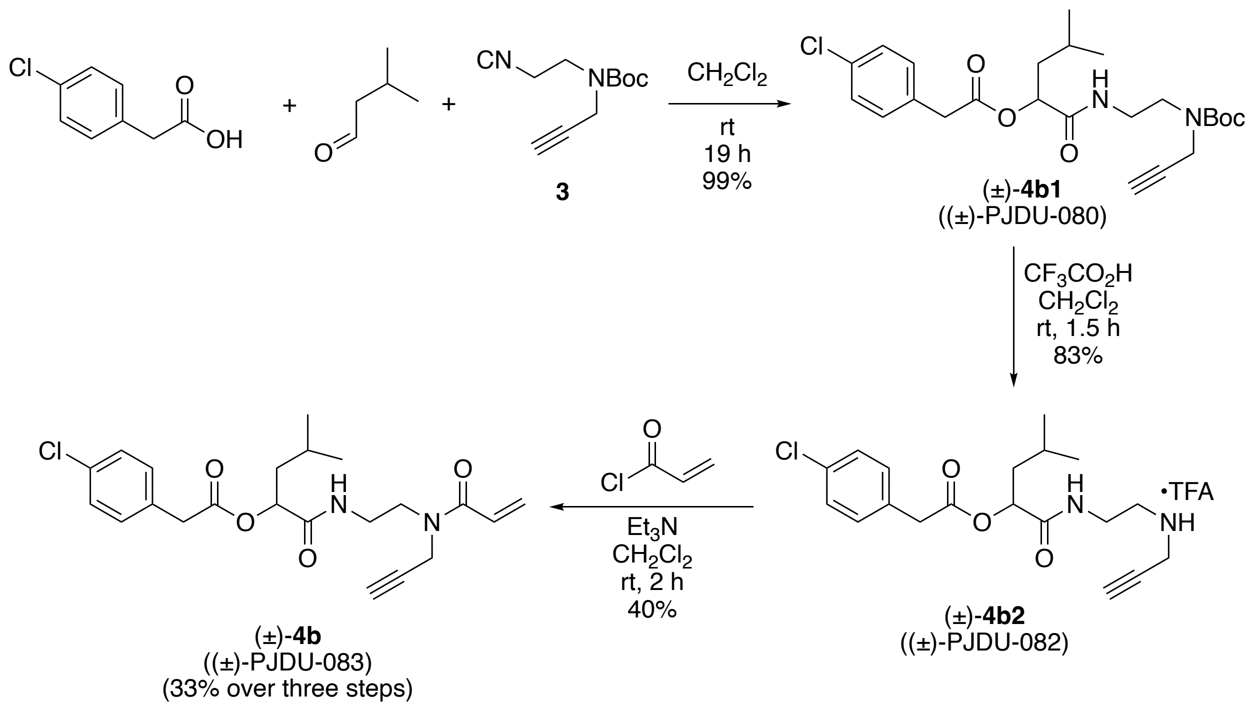

Scheme S2. *Synthesis of P-3CR-based FFSMP (±)-**4b** ((±)-PJDU-083).*

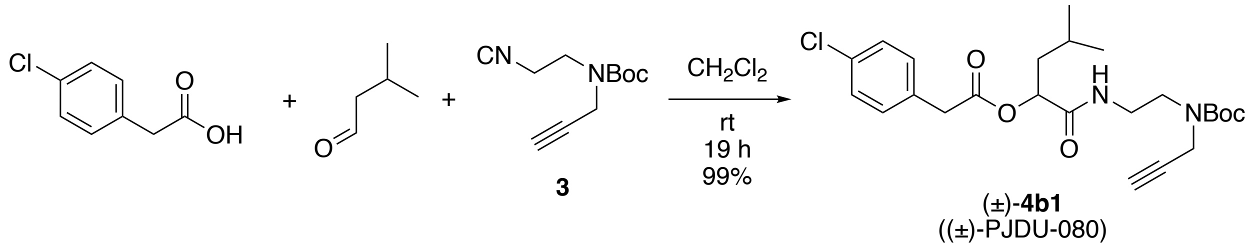

*1-((2-((tert-Butoxycarbonyl)(prop-2-yn-1-yl)amino)ethyl)amino)-4-methyl-1-oxopentan-2-yl 2-(4-chlorophenyl)acetate ((±)-**4b1**) ((±)-PJDU-080).* Isocyanide **3** (86 mg, 0.41 mmol, 1 eq.) was dissolved and stirred in CH_2Cl_2 (1 mL, 0.5 M), then isovaleraldehyde (0.05 mL, 0.46 mmol, 1.1 eq.) was added, followed immediately by the addition of 4-chlorophenyl acetic acid (78 mg, 0.45 mmol, 1.1 eq.). The reaction mixture was stirred at room temperature for 19 hours wherein complete consumption of the isocyanide was observed by TLC. The reaction mixture was then diluted with EtOAc and sequentially washed with sat. aq. $NaHCO_3$ solution, H_2O, and brine. The organic layer was then concentrated and purified by silica gel flash column chromatography (gradient elution with 40-50% EtOAc/hex) to provide ester (±)-**4b1** (190 mg, 99%, (±)-PJDU-080) as a pale yellow oil.

R_f = 0.40 (50% EtOAc/hexanes)

^{1}H NMR (400 MHz, $CDCl_3$, *peaks reported are for the major rotamer only) δ 7.32-7.30 (m, 2H), 7.26-7.23 (m, 2H), 7.08 (bs, 1H), 5.18 (t, *J* = 6.8 Hz, 1H), 4.01 (bs, 2H), 3.77-3.69 (m, 2H), 3.50-3.50 (m, 1H), 3.45-3.37 (m, 3H), 2.26 (t, *J* = 2.2 Hz, 1H), 1.69-1.66 (m, 2H), 1.60-1.50 (m, 1H), 1.49 (s, 9H), 0.86 (t, *J*, = 6.6 Hz, 6H)

^{13}C NMR (101 MHz, $CDCl_3$, *peaks reported are for the major rotamer only) δ 176.0, 170.6, 156.3, 133.2, 132.1, 130.8, 128.8, 81.1, 79.3, 73.0, 71.9, 45.7, 43.1, 40.8, 39.4, 37.4, 28.3, 24.5, 23.1, 21.6

HRMS (ESI+) *m/z* [M + Na] calcd for $C_{24}H_{33}ClN_2O_5Na$ 487.1970, found 487.1968

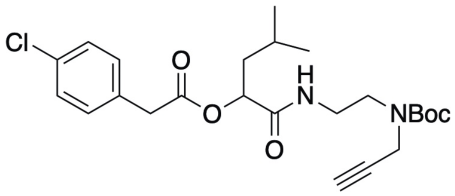

(±)-**4b1**
((±)-PJDU-080)
^{1}H NMR (400 MHz, $CDCl_3$ with TMS)

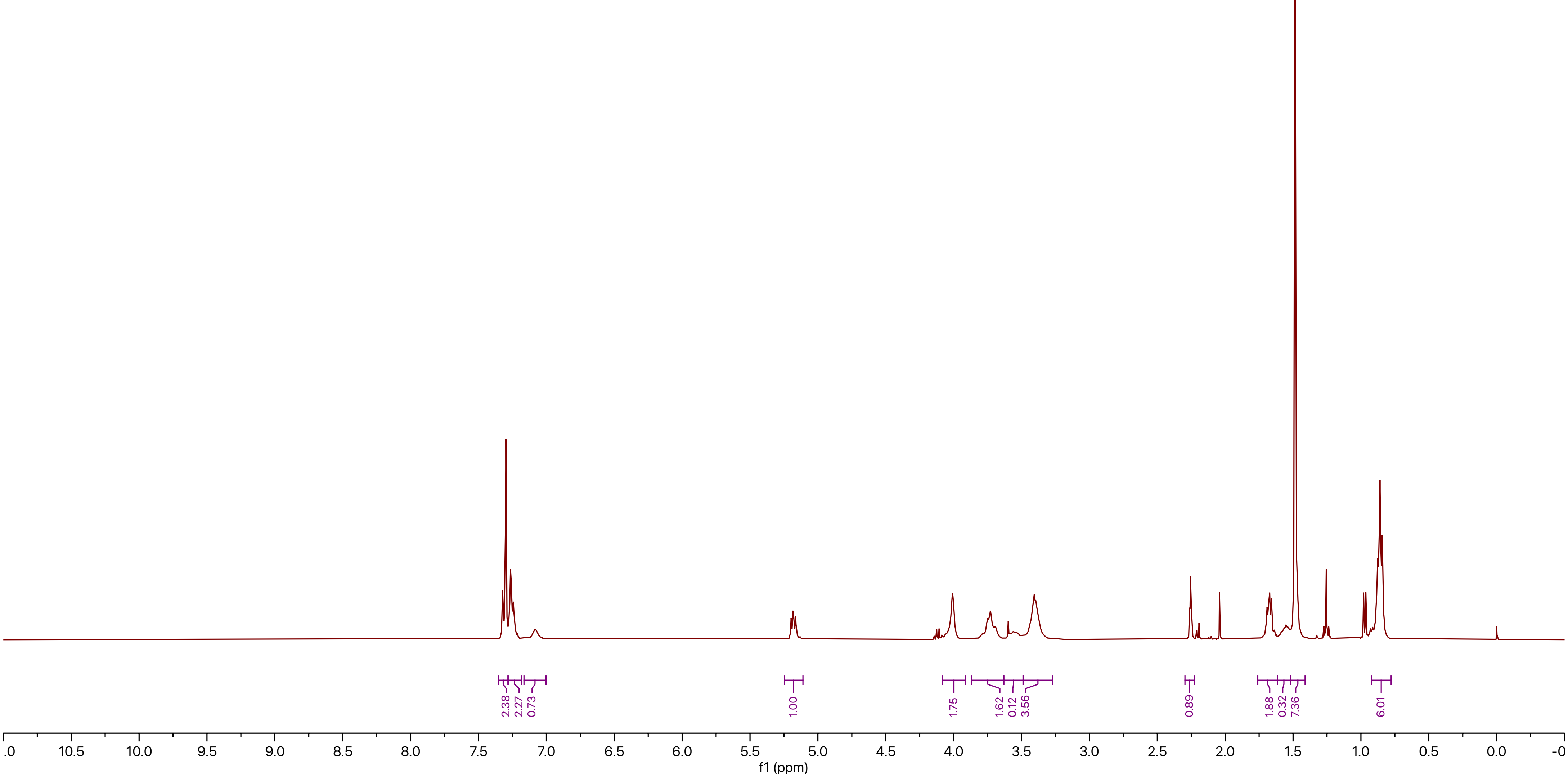

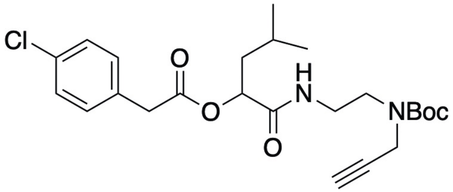

(±)-**4b1**
((±)-PJDU-080)
^{13}C NMR (101 MHz, $CDCl_3$ with TMS)

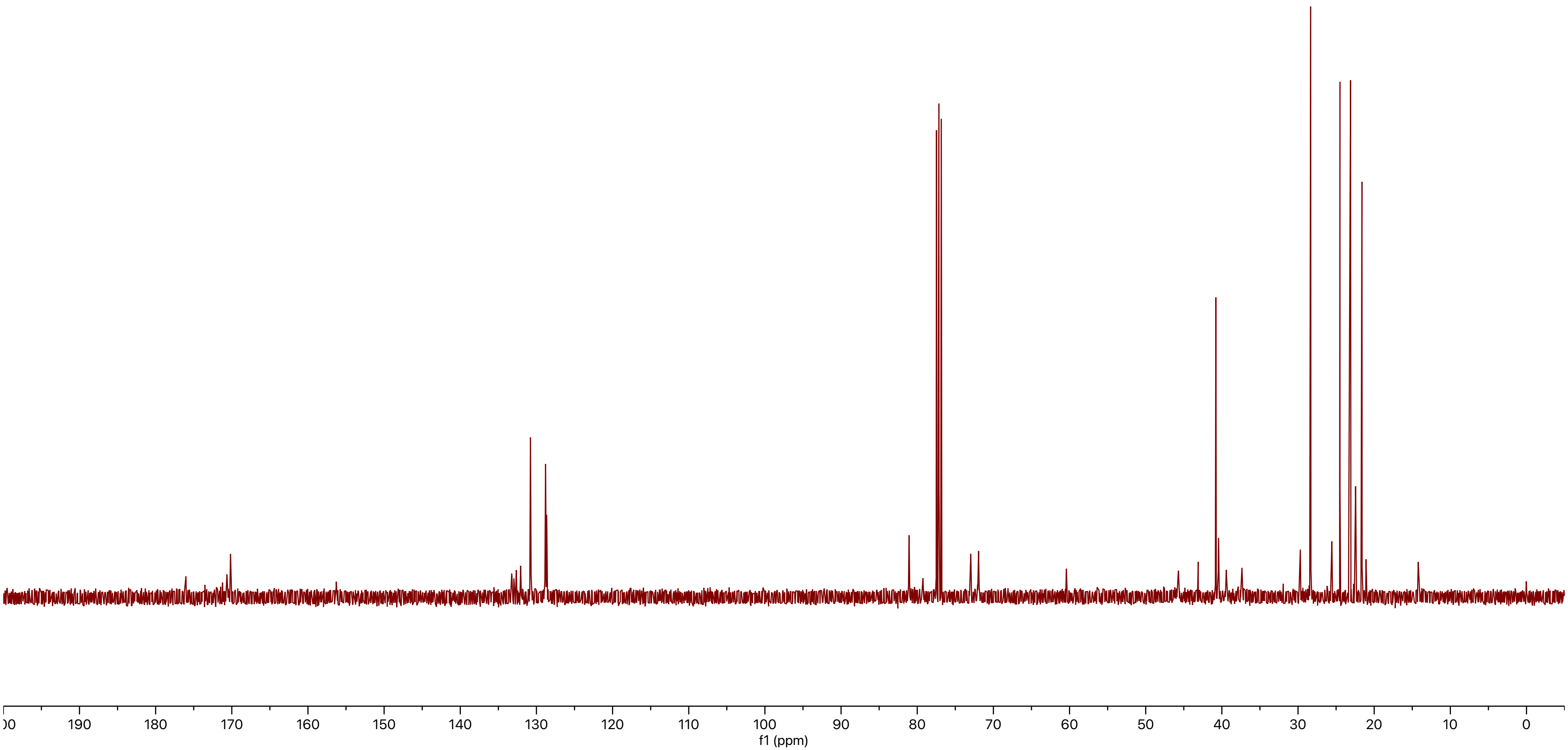

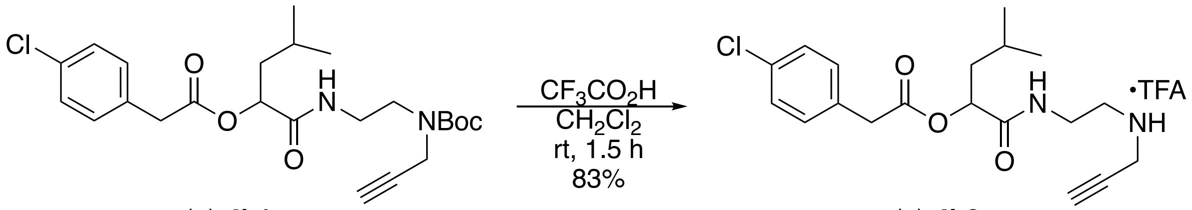

(±)-**4b1**
((±)-PJDU-080)

(±)-**4b2**
((±)-PJDU-082)

*4-Methyl-1-oxo-1-((2-(prop-2-yn-1-ylamino)ethyl)amino)pentan-2-yl 2-(4-chlorophenyl)acetate ((±)-**4b2**) ((±)-PJDU-082).* A 20 mL scintillation vial equipped with a stirbar, septum, and argon inlet needle was charged with Boc carbamate (±)-**4b1** (170 mg, 0.37 mmol, 1 eq.; (±)-PJDU-080) and CH_2Cl_2 (4 mL, 0.1 M). Trifluoroacetic acid (0.28 mL, 3.7 mmol, 10 eq.) was added dropwise over 1 minute and then the reaction mixture was stirred for 1.5 hours at room temperature, wherein TLC analysis indicated complete consumption of the starting Boc carbamate. The stirbar was removed and the crude mixture was concentrated under reduced pressure to provide 2° amine (±)-**4b2** (145 mg, 83%, (±)-PJDU-082) as the trifluoroacetate salt.

R_f = 0.35 (85% EtOAc/hexanes)

^{1}H NMR (400 MHz, $CDCl_3$, *peaks reported are for the major rotamer only) δ 7.84 (t, *J* = 5.2 Hz, 1 H), 7.21-7.17 (m, 3H), 7.13-7.08 (m, 3H), 4.91 (dd, *J* = 2.8, 10.0 Hz, 1H), 3.69 (d, *J* = 2.0 Hz, 2H), 3.60 (d, *J* = 2.0 Hz, 2H), 3.56-3.43 (m, 2H), 3.19 (app s, 2H), 2.46 (app s, 1H), 1.69-1.62 (m, 1H), 1.50-1.47 (m, 2H), 0.80 (d, 6.0 Hz, 3H), 0.74 (d, 6.0 Hz, 3H)

^{13}C NMR (101 MHz, $CDCl_3$, *peaks reported are for the major rotamer only) δ 172.8, 171.5, 133.4, 132.0, 130.9, 128.8, 78.2, 73.5, 72.5, 46.5, 40.2, 36.8, 36.1, 24.5, 23.0, 21.5, 21.4

HRMS (ESI+) *m*/*z* [M + H] calcd for $C_{19}H_{25}ClN_2O_3H$ 365.1626, found 365.1627

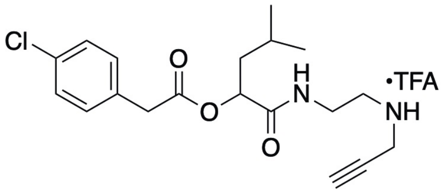

(±)-**4b2**
((±)-PJDU-082)
^{1}H NMR (400 MHz, $CDCl_3$ with TMS)

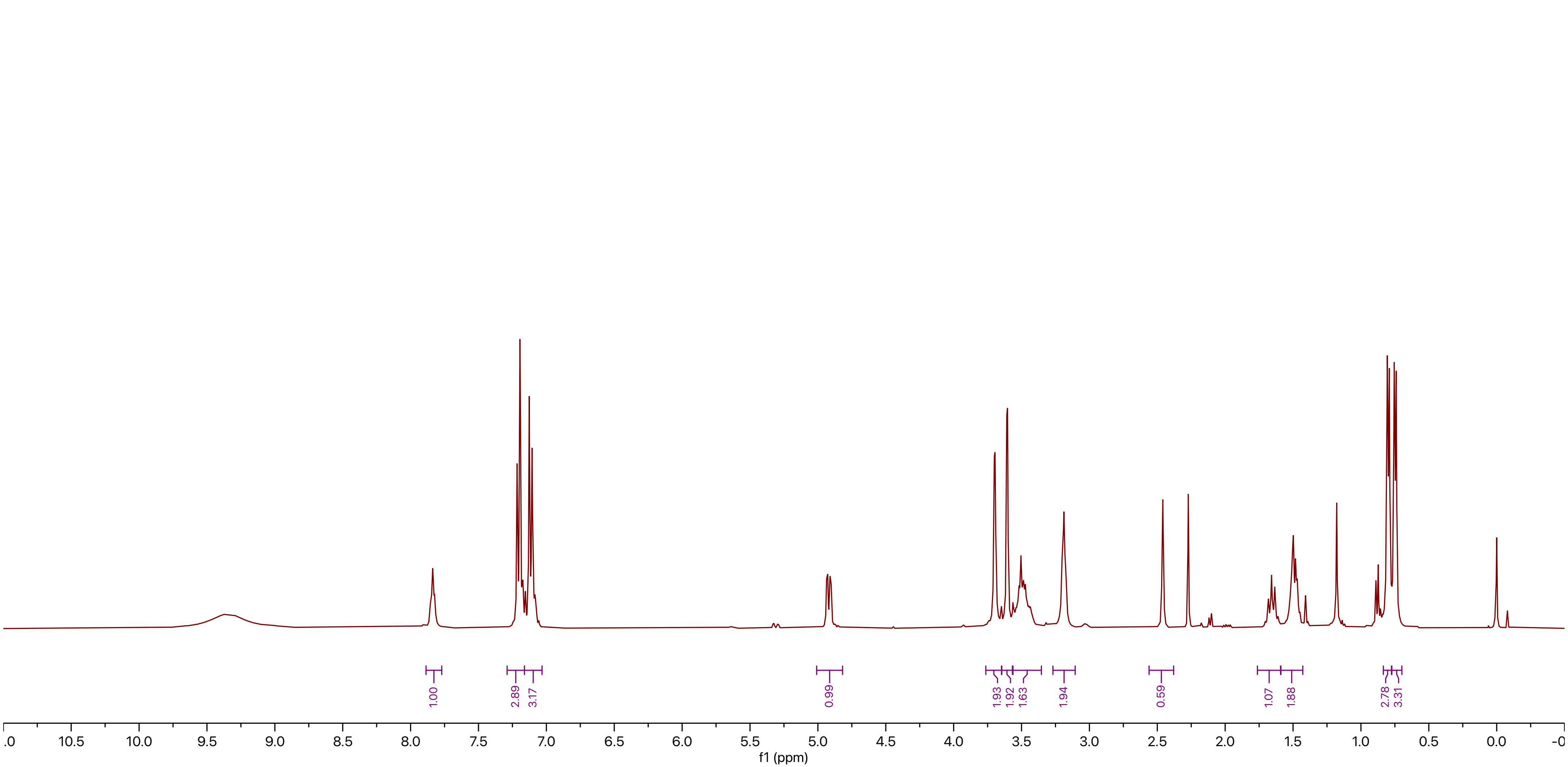

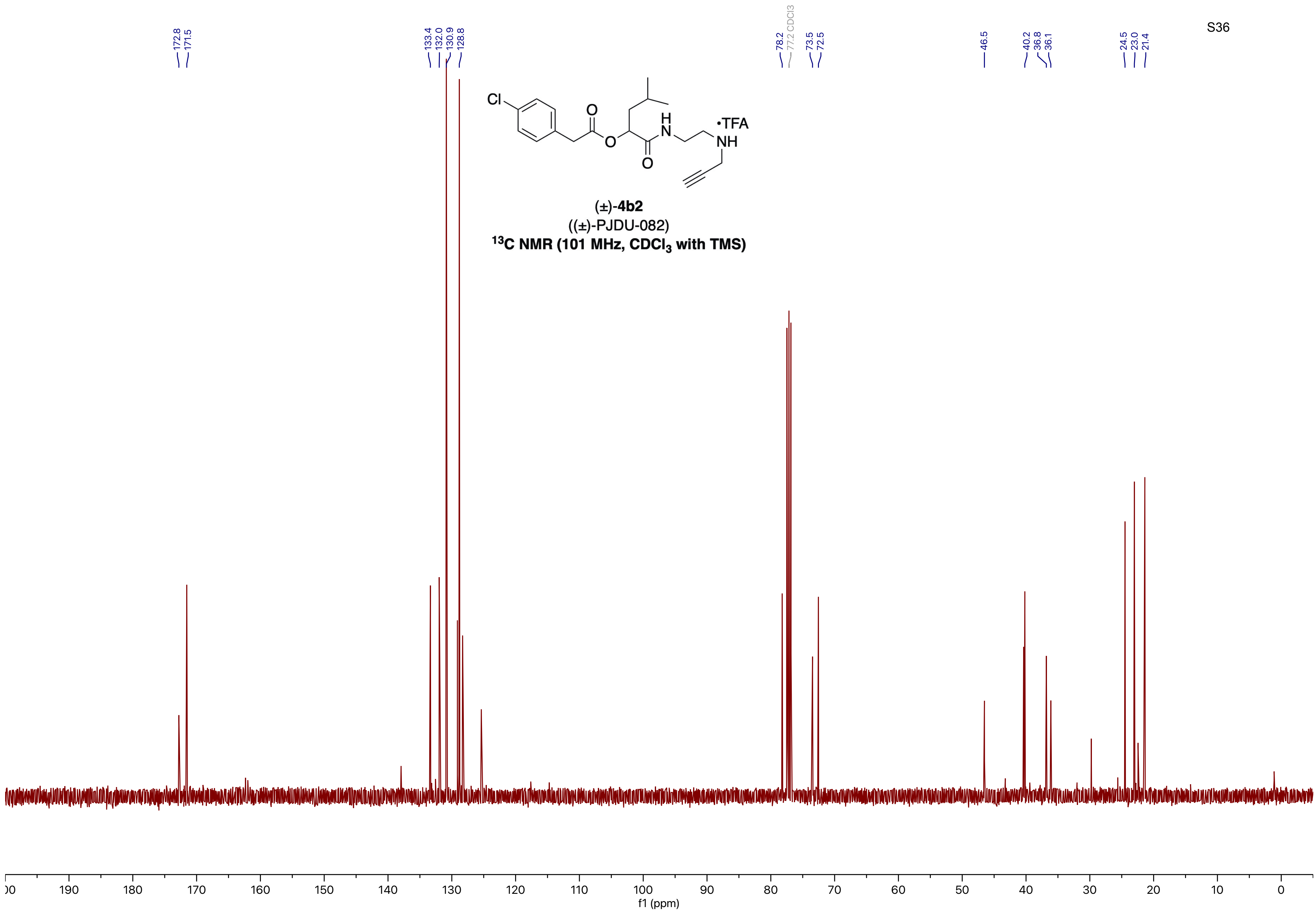

(±)-**4b2**
((±)-PJDU-082)
^{13}C NMR (101 MHz, CDCl$_3$ with TMS)

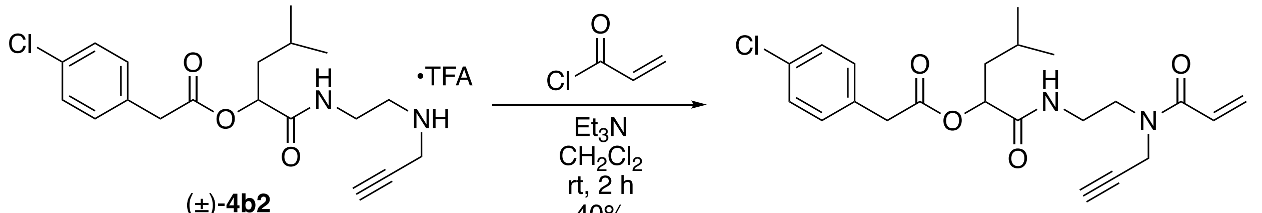

(±)-**4b2**
((±)-PJDU-082)

(±)-**4b**
((±)-PJDU-083)

*4-Methyl-1-oxo-1-((2-(N-(prop-2-yn-1-yl)acrylamido)ethyl)amino)pentan-2-yl 2-(4-chlorophenyl)acetate ((±)-**4b**) ((±)-PJDU-083).* A flame-dried, round-bottom flask equipped with a stirbar, septum, and argon inlet needle was charged with the TFA salt of 2˚ propargyl amine substrate (±)-**4b2** (145 mg, 0.38 mmol, 1 eq.; (±)-PJDU-082) and CH_2Cl_2 (7 mL, 0.05 M), then cooled to 0°C with a saturated ice-water bath. Triethylamine (0.16 mL, 1.1 mmol, 3 eq.) was then added dropwise over 1 minute, followed by dropwise addition of acryloyl chloride (0.05 mL, 0.6 mmol, 1.5 eq.) over 1 minute. The ice bath was then removed and the reaction mixture was allowed to warm to room temperature. The reaction mixture was stirred for 2 hours, wherein consumption of the starting 2˚ propargyl amine substrate was observed by TLC. Next, the reaction mixture was transferred to a separatory funnel and washed with H_2O. The aq. layer was then extracted with CH_2Cl_2. The combined organic layers were then washed with sat. aq. $NaHCO_3$ solution, dried ($MgSO_4$), filtered, and concentrated to produce a crude residue, which was purified by silica gel flash column chromatography (gradient elution with 30-50% EtOAc/hexanes) to give acrylamide-based FFSMP (±)-**4b** (63 mg, 40%, ((±)-PJDU-083)) as a pale yellow oil.

R_f = 0.17 (50% EtOAc/hexanes)

^{1}H NMR (400 MHz, $CDCl_3$, *peaks reported are for the major rotamer only) δ 7.31-7.23 (m, 4H), 7.10 (bs, 1H), 6.60 (dd, *J* = 10.4, 16.8 Hz, 1H), 6.32 (dd, *J* = 1.6, 16.8 Hz, 1H), 5.77 (dd, *J* = 1.6, 10.4 Hz, 1H), 5.14 (t, *J* = 6.6 Hz, 1H), 4.09 (t, *J* = 2.4 Hz, 2H), 3.76-3.70 (m, 2H), 3.64-3.55 (m, 2H), 3.42-3.38 (m, 2H), 2.34 (t, *J* = 2.4 Hz, 1H), 1.55-1.46 (m, 1H), 0.83 (d, *J* = 6.8 Hz, 3H), 0.81 (d, *J* = 6.8 Hz, 3H)

^{13}C NMR (101 MHz, $CDCl_3$, *peaks reported are for the major rotamer only) δ 171.0, 170.4, 168.2, 133.2, 132.2, 130.9, 129.0, 128.8, 127.3, 78.0, 73.6, 72.9, 46.4, 45.5, 40.8, 39.0, 29.8, 24.6, 23.2, 21.6

HRMS (ESI+) *m/z* [M + Na] calcd for $C_{22}H_{27}ClN_2O_4Na$ 441.1552, found 441.1548

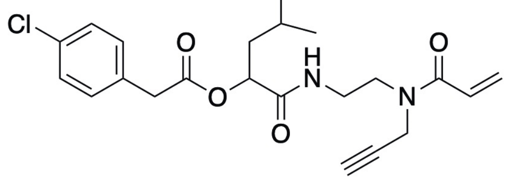

(±)-**4b**
((±)-PJDU-083)
^{1}H NMR (400 MHz, $CDCl_3$ with TMS)

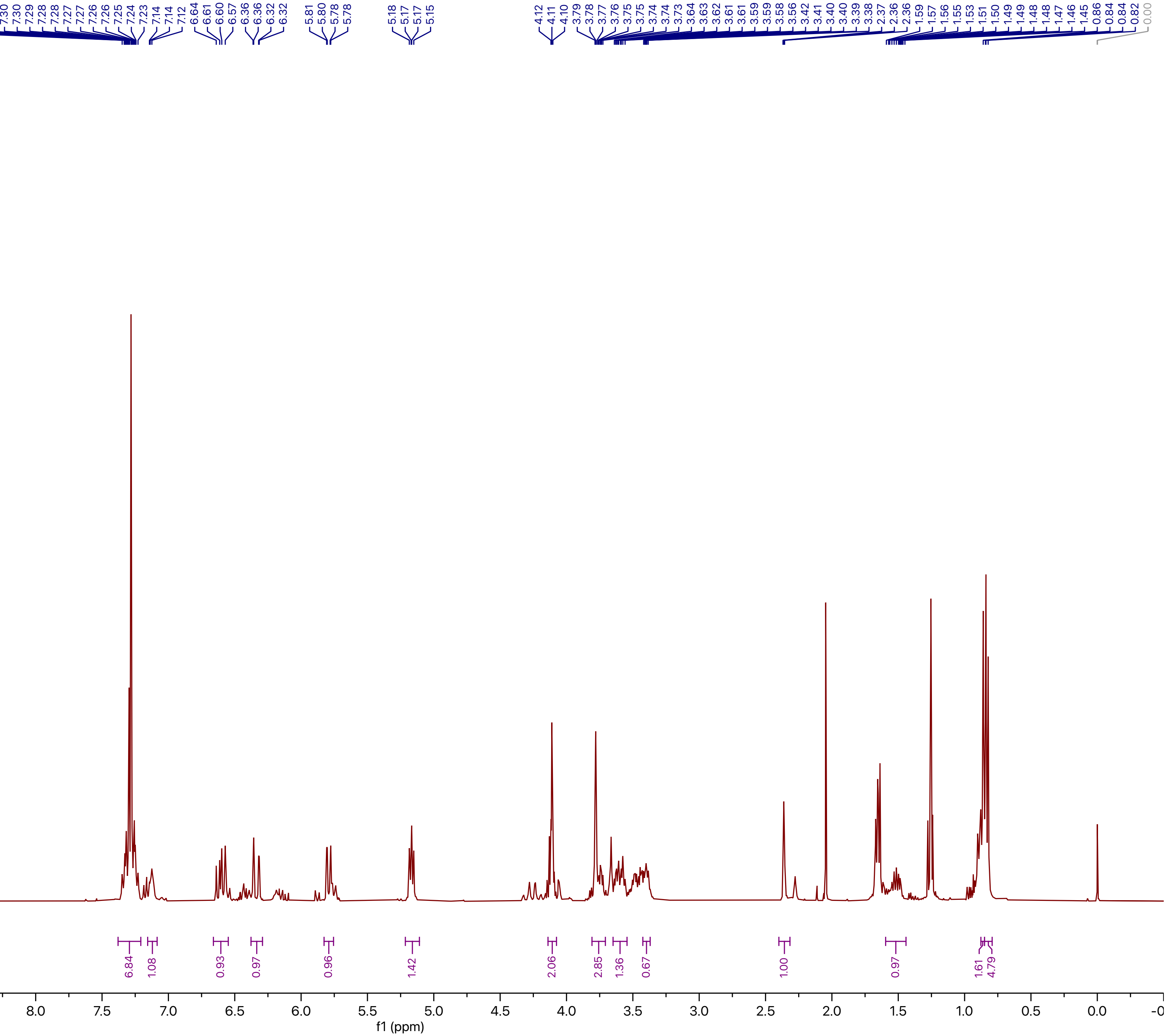

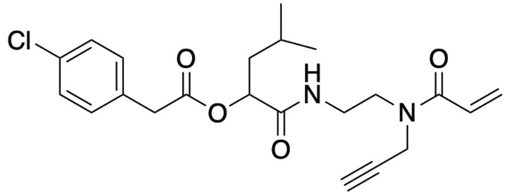

(±)-**4b**
((±)-PJDU-083)
^{13}C NMR (101 MHz, $CDCl_3$ with TMS)

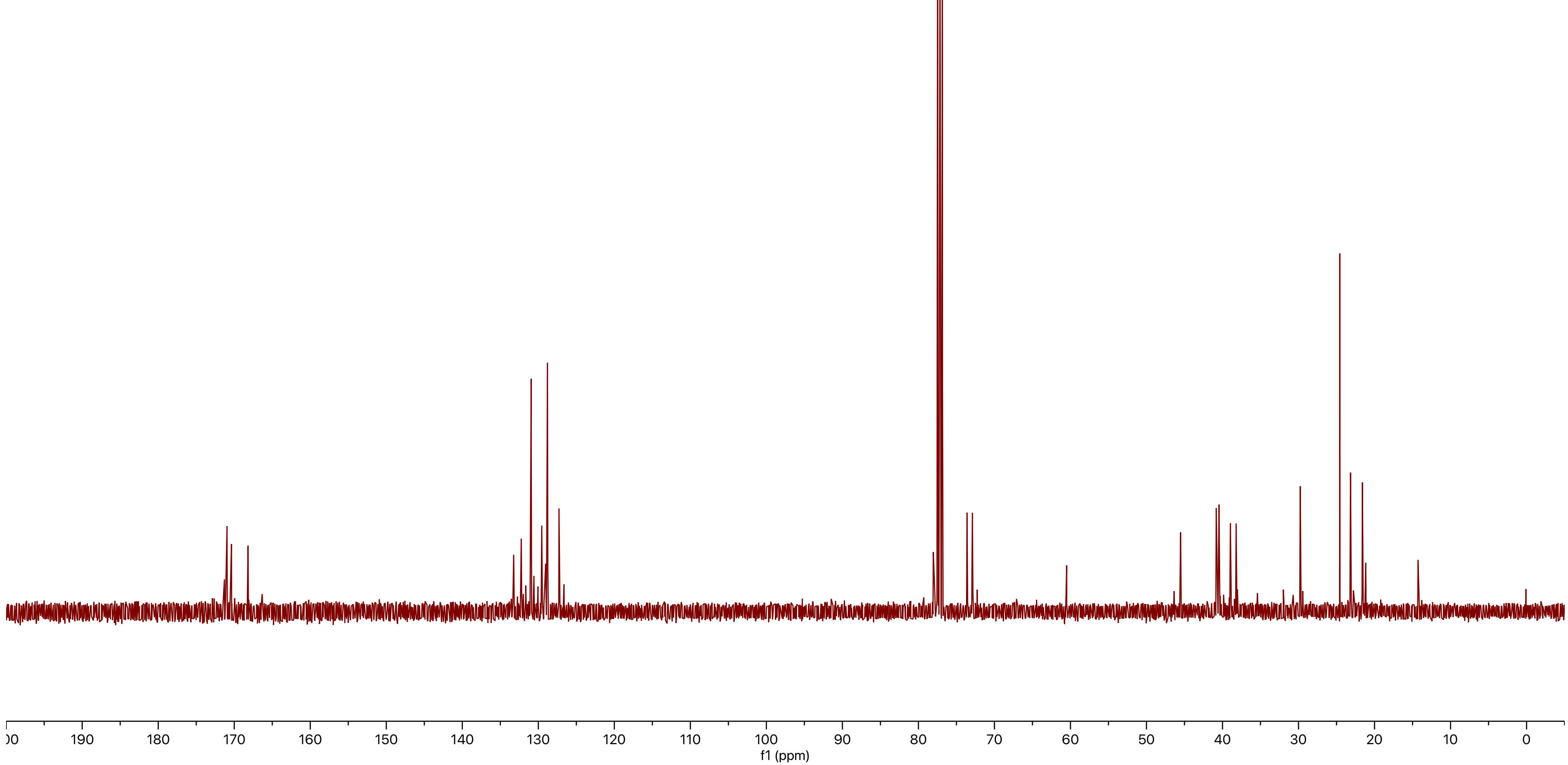

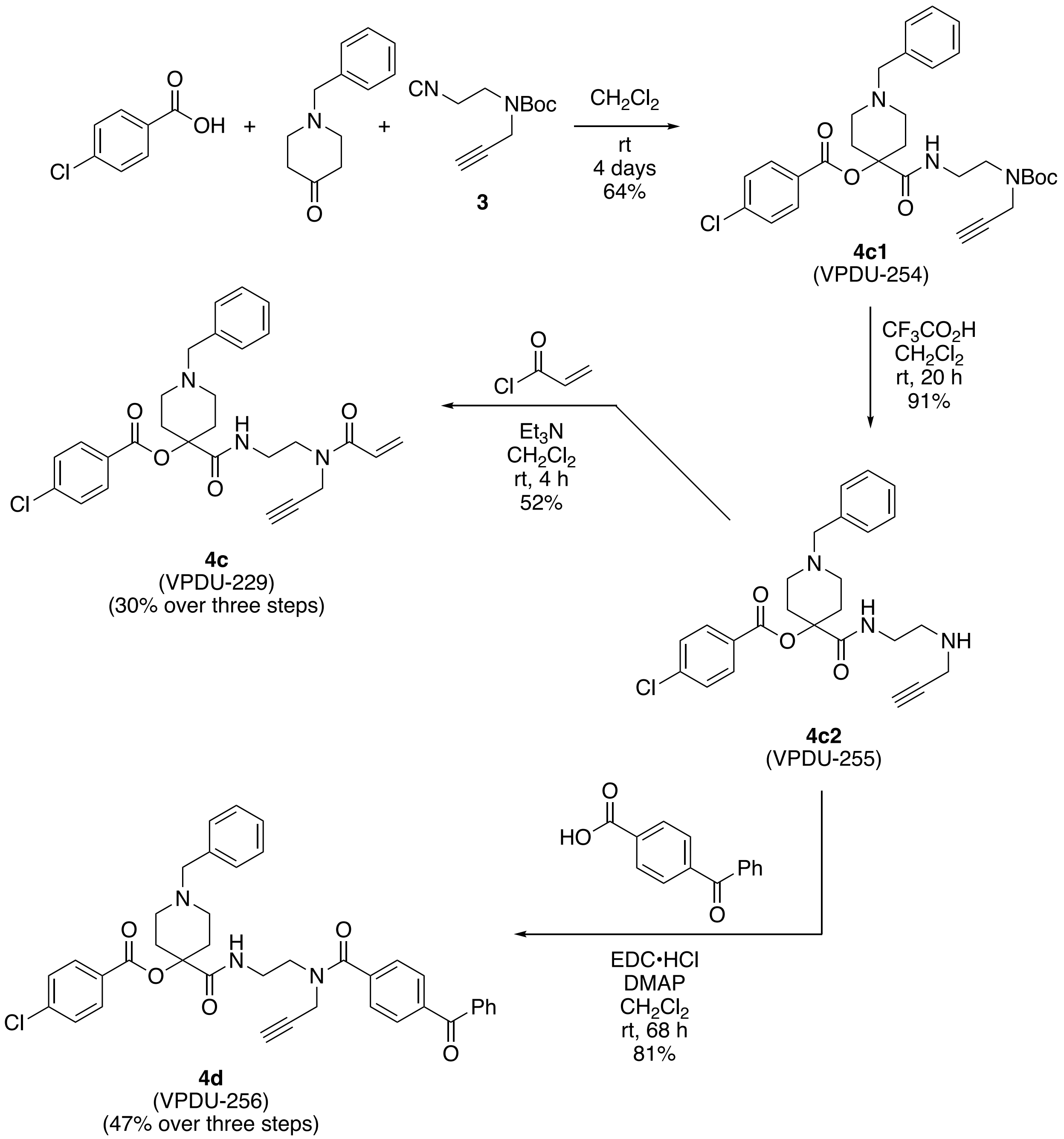

Scheme S3. *Synthesis of P-3CR-based FFSMPs* ***4c*** *(VPDU-229) and* ***4d*** *(VPDU-256).*

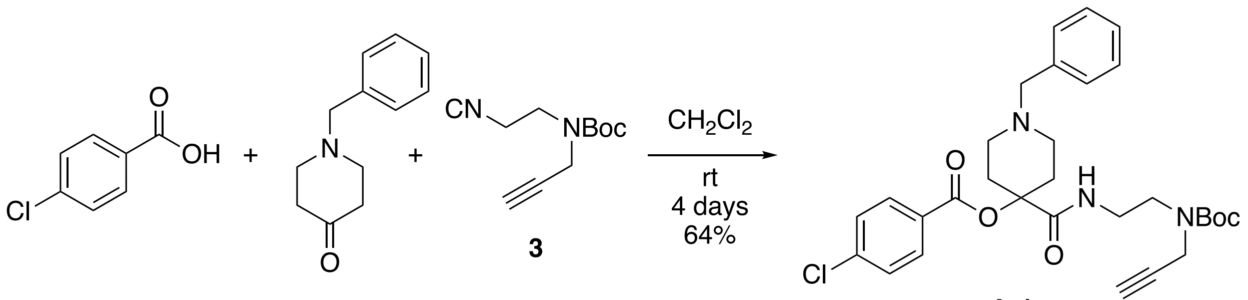

*1-Benzyl-4-((2-((tert-butoxycarbonyl)(prop-2-yn-1-yl)amino)ethyl)carbamoyl)piperidin-4-yl 4-chlorobenzoate (**4c1**) (VPDU-254).* Isocyanide **3** (224 mg, 1.09 mmol, 1 eq.) was dissolved and stirred in CH_2Cl_2 (3 mL), then 1-benzyl-4-piperidone (0.21 mL, 1.12 mmol, 1.1 eq.) was added, followed immediately by the addition of 4-chlorobenzoic acid (175 mg, 1.12 mmol, 1.1 eq.). The reaction mixture was stirred at room temperature for 4 days wherein complete consumption of the isocyanide was observed by TLC. The reaction mixture was then diluted with EtOAc and sequentially washed with sat. aq. $NaHCO_3$ solution, H_2O, and brine. The organic layer was then concentrated and purified by silica gel flash column chromatography (elution with 50% EtOAc/hexanes) to give ester **4c1** (385 mg, 64%, VPDU-254) as a white sticky solid.

R_f = 0.2 (50% EtOAc/hexanes)

^{1}H NMR (400 MHz, $CDCl_3$, *peaks reported are for the major rotamer only) δ 7.99 (d, J = 8.5 Hz, 2H), 7.43 (d, J = 8.6 Hz, 2H), 7.31 (d, J = 4.3 Hz, 4H), 7.29 – 7.20 (m, 1H), 6.91 (s, 1H), 4.08 – 3.89 (m, 2H), 3.49 (d, J = 17.0 Hz, 6H), 2.80 (d, J = 10.4 Hz, 2H), 2.30 (t, J = 11.5 Hz, 5H), 2.25 – 2.09 (m, 2H), 1.40 (s, 9H)

^{13}C NMR (126 MHz, $CDCl_3$, *peaks reported are for the major rotamer only) δ 172.1, 164.0, 156.2, 139.7, 138.2, 131.2, 129.5, 129.0, 128.8, 128.2, 127.1, 80.9, 80.3, 77.4, 71.8, 62.8, 48.8, 46.0, 37.4, 32.2, 29.7, 28.2

HRMS (ESI+) m/z [M + H] calcd for $C_{30}H_{36}ClN_3O_5H$ 554.2416, found 554.2414

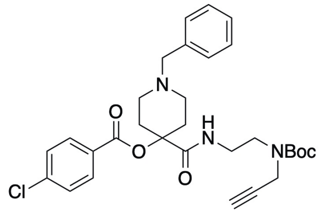

4c1
(VPDU-254)
^{1}H NMR (400 MHz, $CDCl_3$ with TMS)

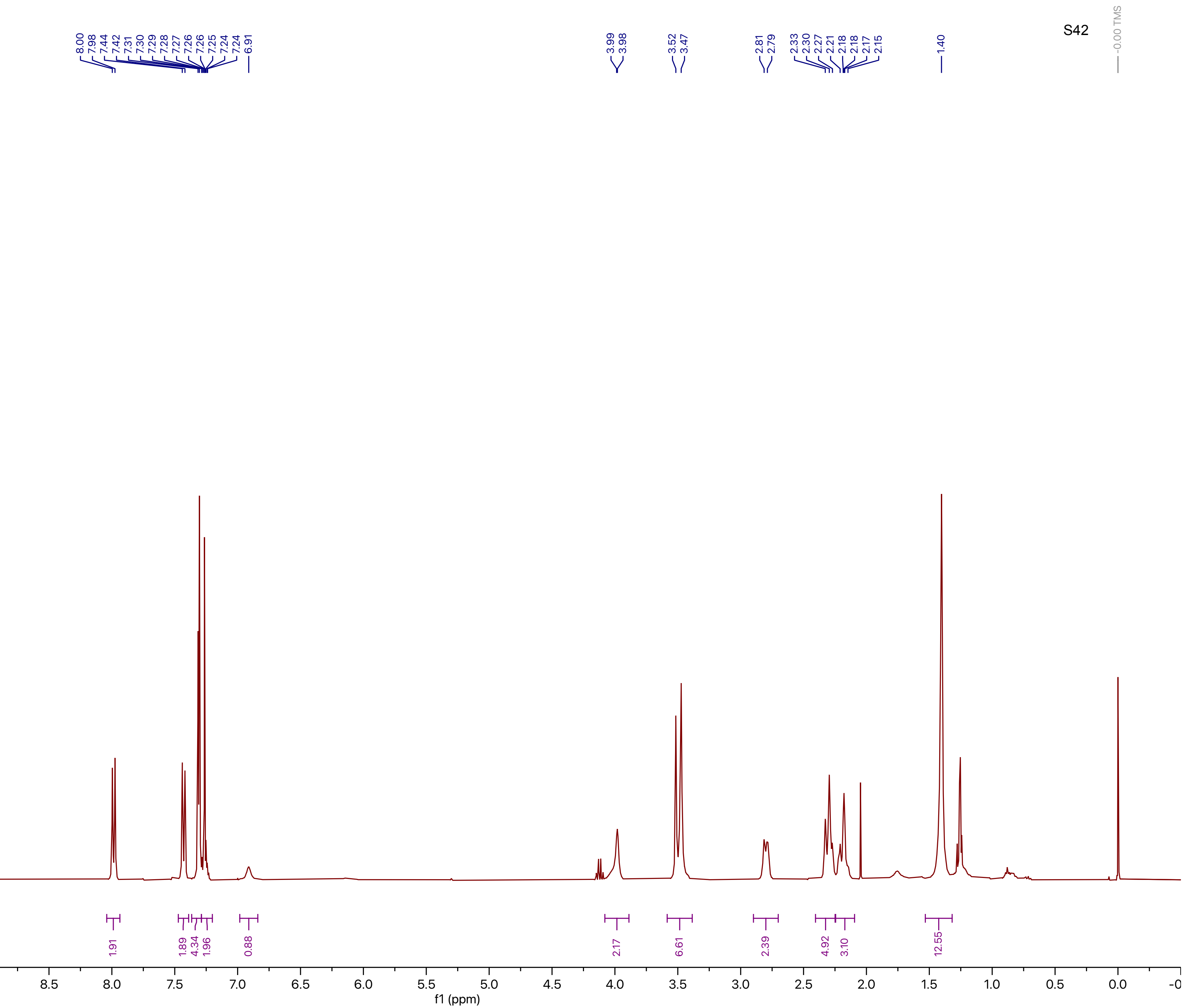

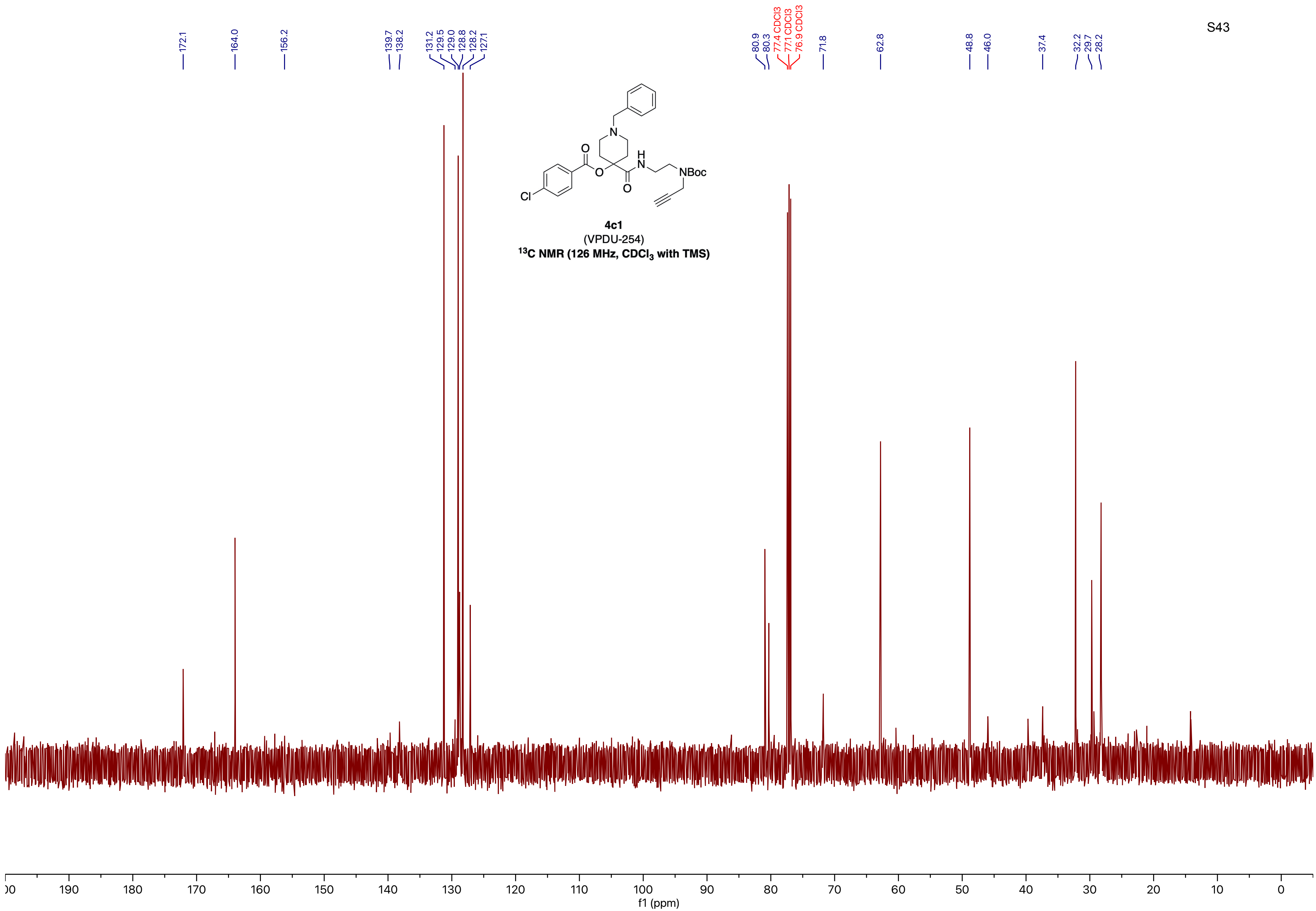
172.1
164.0
156.2
139.7
138.2
131.2
129.5
129.0
128.8
128.2
127.1
80.9
80.3
77.4 CDCl3
77.1 CDCl3
76.9 CDCl3
71.8
62.8
48.8
46.0
37.4
32.2
29.7
28.2
O
O
Cl
N
H
N
NBoc
O
4c1
(VPDU-254)
^{13}C NMR (126 MHz, CDCl$_3$ with TMS)
f1 (ppm)

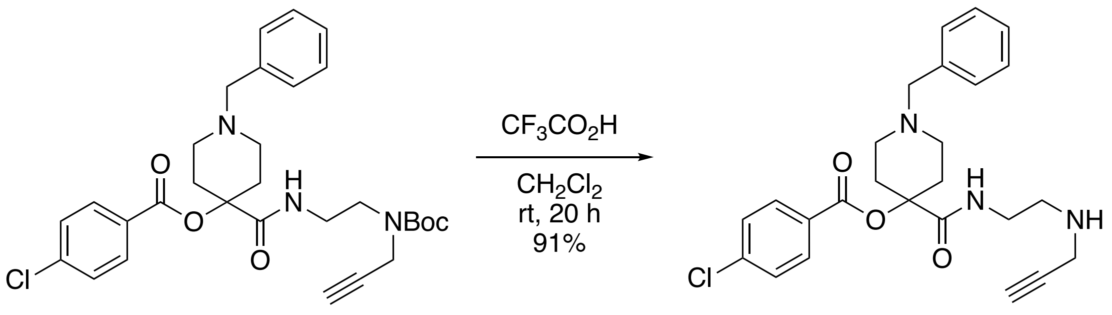

4c1
(VPDU-254)

4c2
(VPDU-255)

*1-Benzyl-4-((2-(prop-2-yn-1-ylamino)ethyl)carbamoyl)piperidin-4-yl 4-chlorobenzoate (**4c2**) (VPDU-255)*. A flame-dried, round-bottom flask equipped with a stirbar, septum, and argon inlet needle was charged with Boc carbamate substrate **4c1** (390 mg, 0.7 mmol, 1 eq.; VPDU-254) and CH_2Cl_2 (12 mL), then cooled to 0°C using a saturated ice-water bath. Trifluoroacetic acid (0.53 mL, 7 mmol, 10 eq.) was then added dropwise over 2 minutes. After the addition, the ice bath was removed and the reaction was allowed to warm to room temperature and stirred for 20 hours, wherein TLC indicated complete consumption of the starting Boc carbamate substrate. The reaction was then quenched by dropwise addition of sat. aq. $NaHCO_3$ solution (~10 mL, vigorous gas evolution) until gas evolution ceased, then 1M aq. NaOH (~4 mL) was added to raise the pH to 8-9. The biphasic mixture was then transferred to a separatory funnel and diluted with CH_2Cl_2, the layers were separated, and the aqueous layer was extracted with CH_2Cl_2. The combined organic layers were then washed with brine, dried ($MgSO_4$), filtered, and concentrated to dryness to provide propargyl amine **4c2** (297 mg, 91%, VPDU-255) as a viscous green oil requiring no additional purification.

R_f = 0.2 (10% MeOH/$CHCl_3$)

^{1}H NMR (500 MHz, $CDCl_3$, *peaks reported are for the major rotamer only) δ 8.03 – 7.93 (m, 2H), 7.47 – 7.36 (m, 2H), 7.36 – 7.27 (m, 4H), 7.27 – 7.19 (m, 1H), 6.49 (t, *J* = 5.5 Hz, 1H), 3.51 (s, 2H), 3.39 – 3.32 (m, 2H), 3.31 (d, *J* = 2.4 Hz, 2H), 2.86 – 2.72 (m, 4H), 2.37 – 2.25 (m, 4H), 2.25 – 2.18 (m, 2H), 2.16 (t, *J* = 2.4 Hz, 1H)

^{13}C NMR (126 MHz, $CDCl_3$, *peaks reported are for the major rotamer only) δ 171.9, 164.0, 139.7, 138.1, 131.1, 129.0, 128.8, 128.4, 128.2, 127.1, 81.8, 80.5, 71.5, 62.8, 48.8, 46.9, 38.7, 37.5, 32.2

HRMS (ESI+) *m/z* [M + H] calcd for $C_{25}H_{28}ClN_3O_3H$ 454.1892, found 454.1893

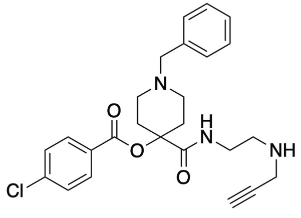

4c2
(VPDU-255)
^{1}H NMR (500 MHz, $CDCl_3$ with TMS)

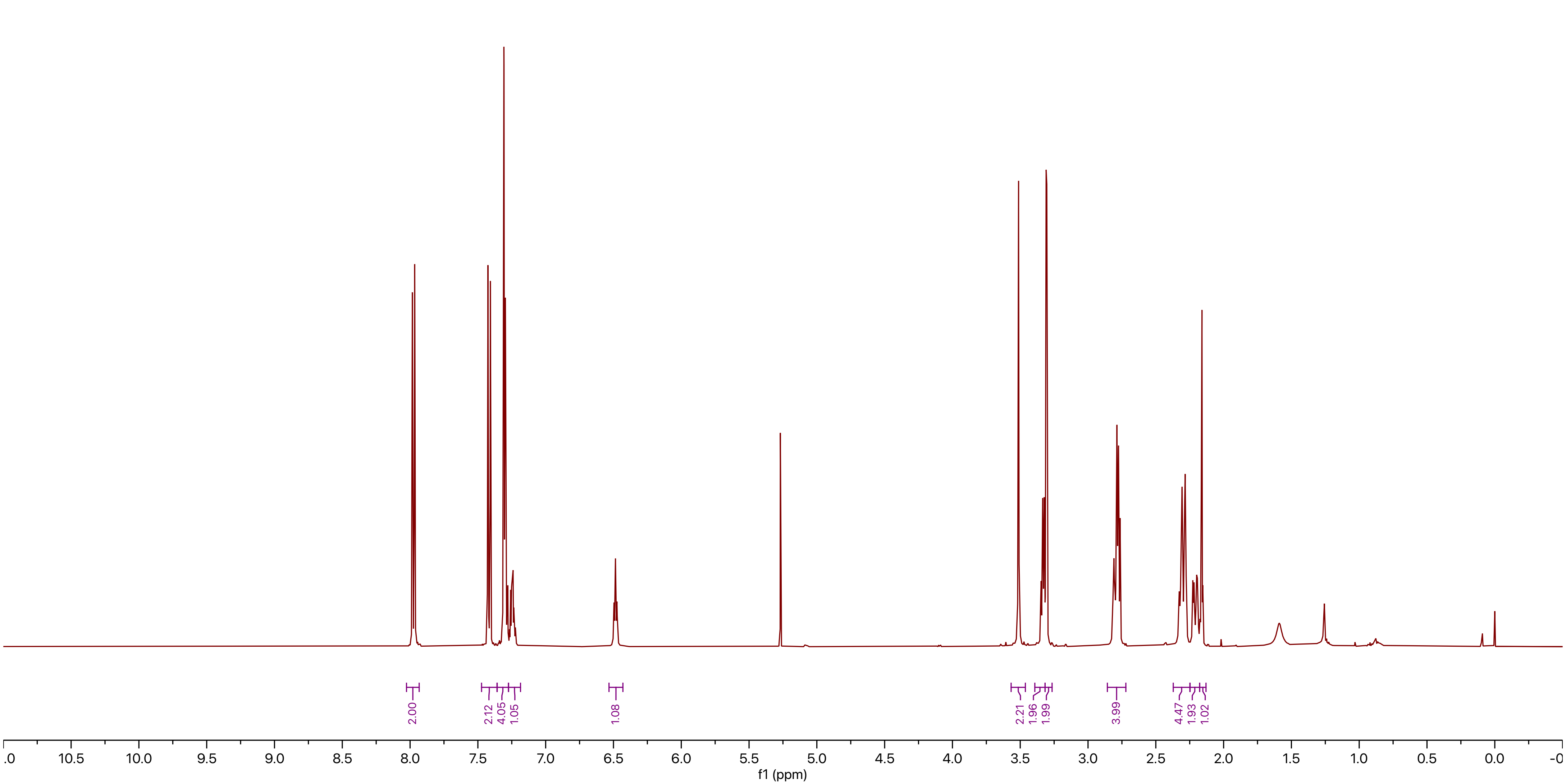

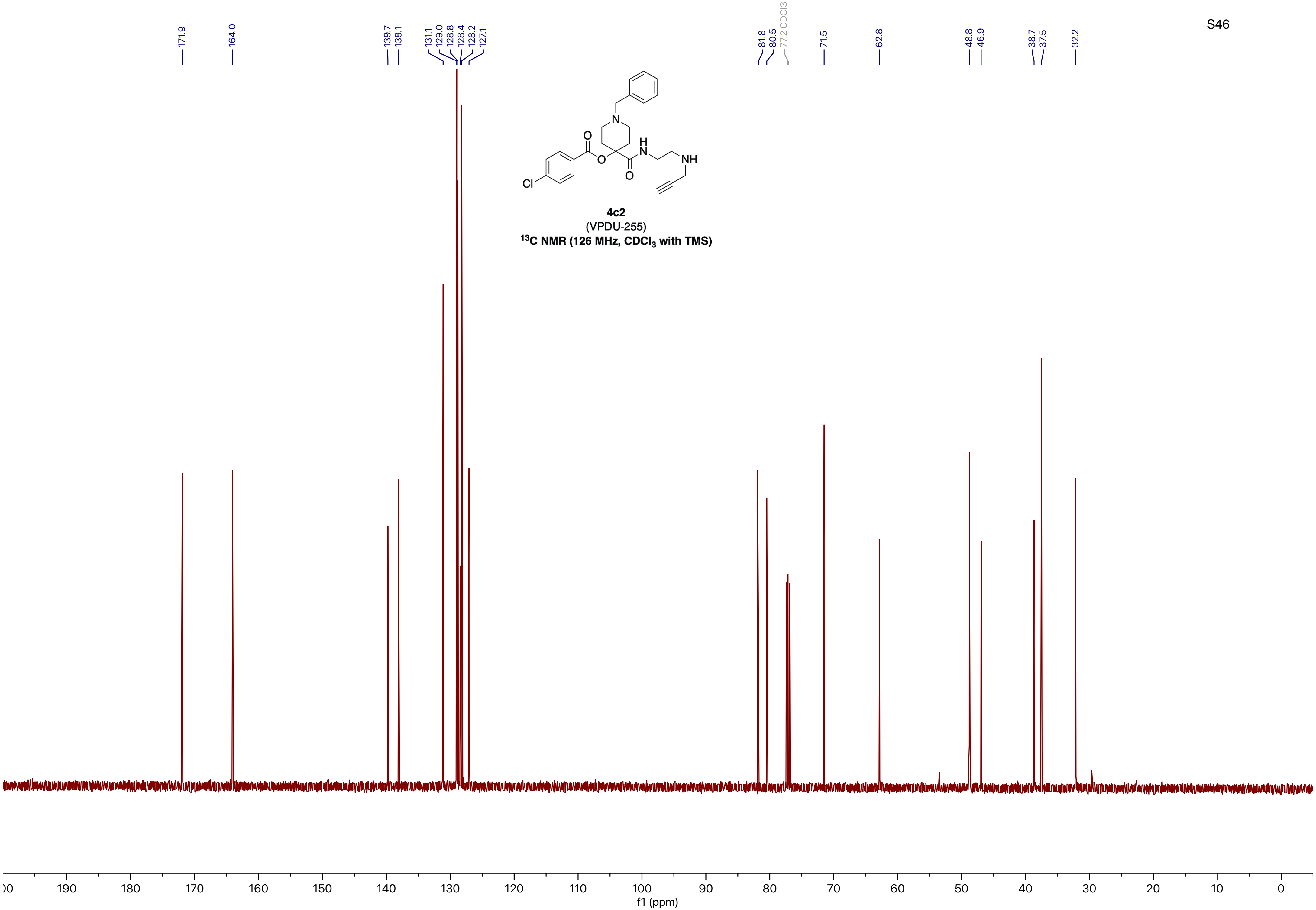

171.9
164.0
139.7
138.1
131.1
129.0
128.8
128.4
128.2
127.1
81.8
80.5
77.2 CDCl3
71.5
62.8
48.8
46.9
38.7
37.5
32.2
Cl
O
O
N
H
N
NH
O
4c2
(VPDU-255)
^{13}C NMR (126 MHz, $CDCl_3$ with TMS)
190
180
170
160
150
140
130
120
110
100
90
80
70
60
50
40
30
20
10
0
f1 (ppm)

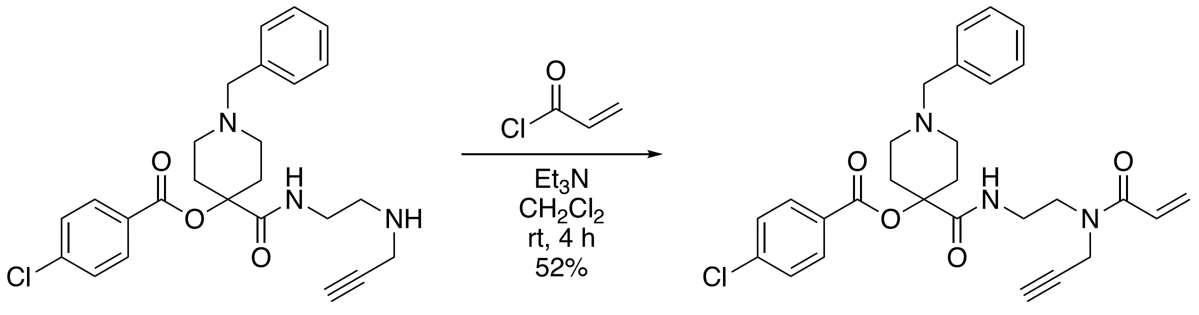

4c2
(VPDU-255)

4c
(VPDU-229)

*1-Benzyl-4-((2-(N-(prop-2-yn-1-yl)acrylamido)ethyl)carbamoyl)piperidin-4-yl 4-chlorobenzoate (**4c**) (VPDU-229).* A flame-dried, round-bottom flask equipped with a stirbar, septum, and argon inlet needle was charged with the 2˚ propargyl amine substrate **4c2** (253 mg, 0.56 mmol, 1 eq.; VPDU-255) and CH_2Cl_2 (11 mL), then cooled to 0°C with a saturated ice-water bath. Triethylamine (0.23 mL, 1.7 mmol, 3 eq.) was then added dropwise over 1 minute, followed by dropwise addition of acryloyl chloride (0.07 mL, 0.84 mmol, 1.5 eq.) over 1 minute. The ice bath was then removed and the reaction mixture was allowed to warm to room temperature. The reaction mixture was stirred for 4 hours, wherein consumption of the starting 2˚ propargyl amine substrate was observed by TLC. Next, the reaction mixture was transferred to a separatory funnel and washed with H_2O. The aq. layer was then extracted with CH_2Cl_2. The combined organic layers were then washed with sat. aq. $NaHCO_3$ solution, dried ($MgSO_4$), filtered, and concentrated to produce a crude residue that was purified by silica gel flash column chromatography (elution with 5% MeOH/$CHCl_3$) to give acrylamide-based FFSMP **4c** (148 mg, 52%, VPDU-229) as a pale yellow oil.

R_f = 0.3 (5% MeOH/$CHCl_3$)

^{1}H NMR (400 MHz, $CDCl_3$, *peaks reported are for the major rotamer only) δ 7.98 – 7.90 (m, 2H), 7.42 (dd, *J* = 8.2, 5.8 Hz, 2H), 7.35 – 7.24 (m, 5H), 7.06 (t, *J* = 4.8 Hz, 1H), 6.47 (dd, *J* = 16.7, 10.5 Hz, 1H), 6.14 (dd, *J* = 17.3, 10.2 Hz, 1H), 5.75 (ddd, *J* = 13.3, 10.3, 1.9 Hz, 1H), 4.09 (d, *J* = 2.5 Hz, 2H), 3.69 – 3.60 (m, 4H), 2.99 – 2.85 (m, 2H), 2.48 – 2.25 (m, 4H), 2.28 – 2.19 (m, 1H)

^{13}C NMR (126 MHz, $CDCl_3$, *peaks reported are for the major rotamer only) δ 172.1, 168.0, 163.9, 139.8, 131.3, 129.5, 129.4, 129.3, 128.8, 128.5, 128.4, 127.6, 126.8, 79.9, 78.1, 73.4, 62.3, 48.4, 45.5, 39.1, 38.1, 31.6

HRMS (ESI+) *m/z* [M + H] calcd for $C_{28}H_{30}ClN_3O_4\ H^+$ 508.1998, found 508.2002

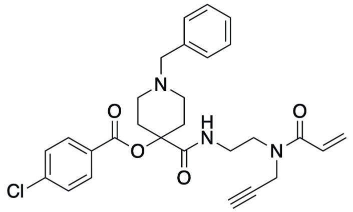

4c
(VPDU-229)
^{1}H NMR (400 MHz, $CDCl_3$ with TMS)

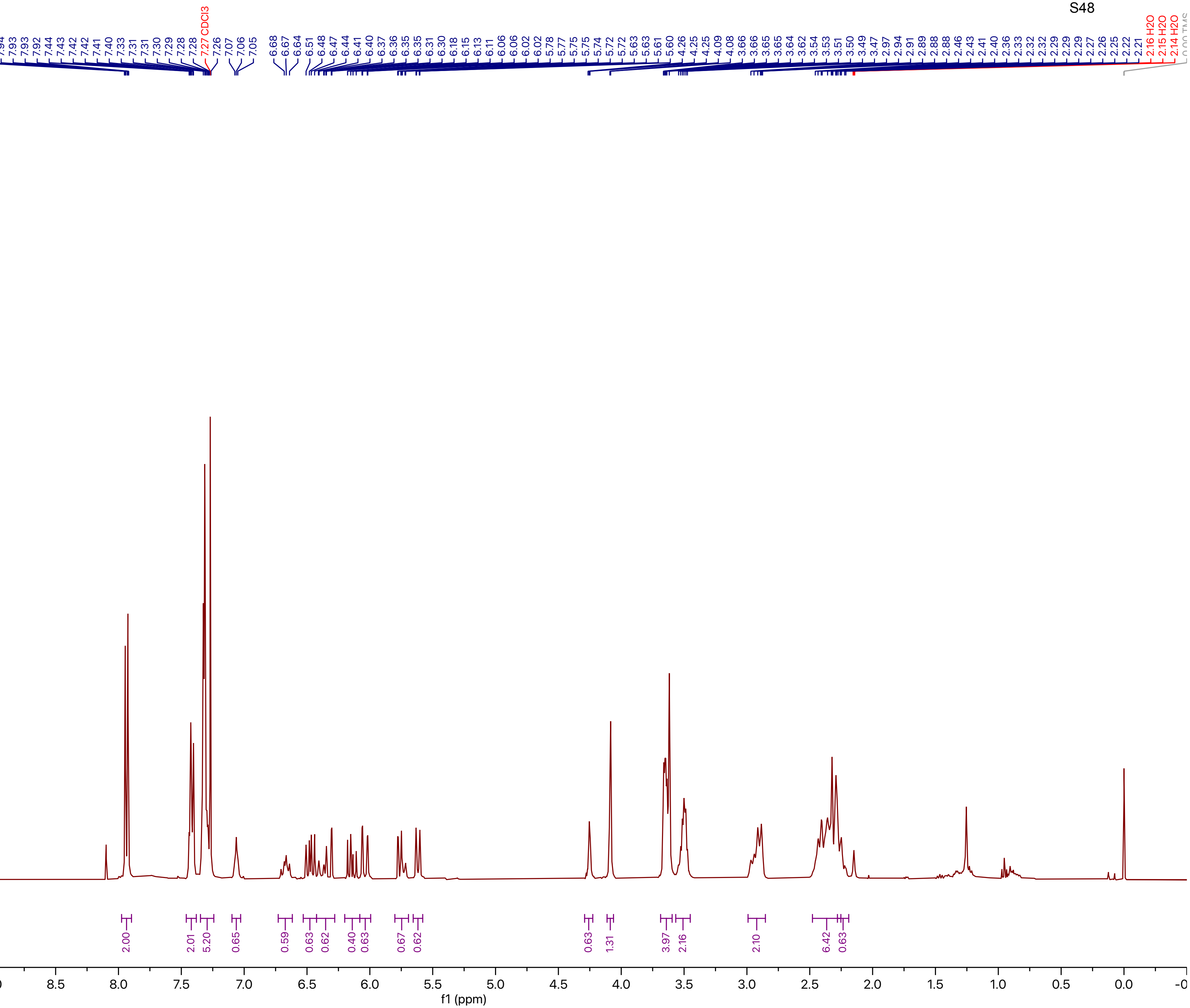

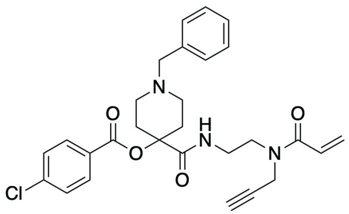

4c
(VPDU-229)
^{13}C NMR (126 MHz, $CDCl_3$ with TMS)

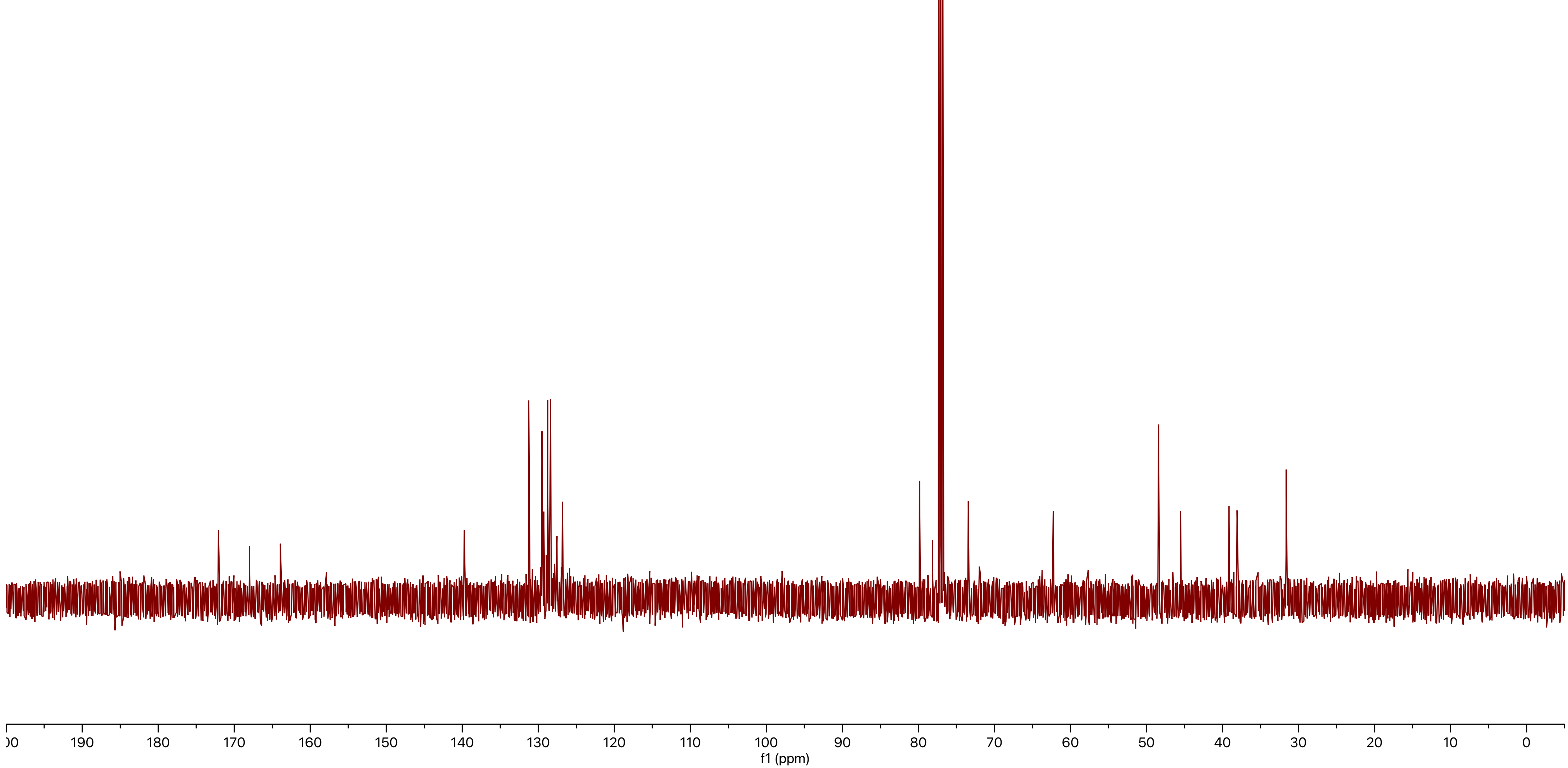

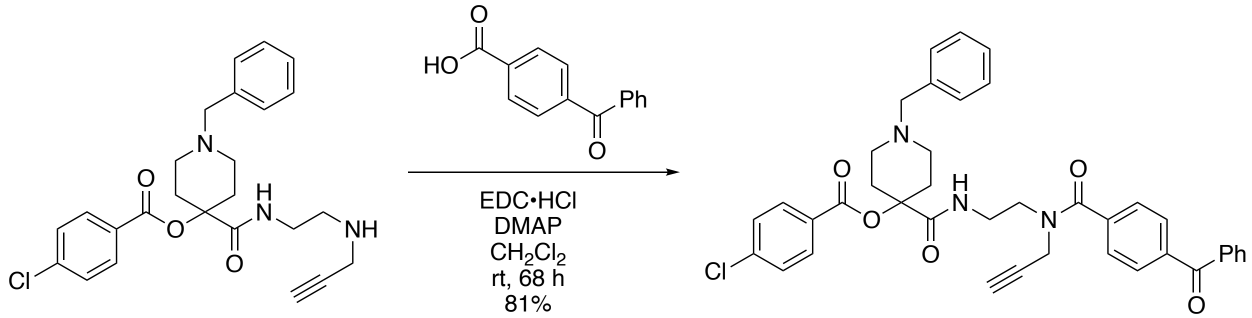

4c2
(VPDU-255)

4d
(VPDU-256)

*4-((2-(4-Benzoyl-N-(prop-2-yn-1-yl)benzamido)ethyl)carbamoyl)-1-benzylpiperidin-4-yl 4-chlorobenzoate (**4d**) (VPDU-256).* A flame-dried, round-bottom flask equipped with a stirbar, septum, and argon inlet needle was charged with 4-benzoylbenzoic acid (290 mg, 1.28 mmol, 1.5 eq.) and CH_2Cl_2 (10 mL). Next, EDC•HCl (326 mg, 1.7 mmol, 2 eq.) was added in a single portion followed by the addition of DMAP (11 mg, 0.09 mmol, 0.1 eq.) in a single portion. The propargyl amine substrate **4c2** (387 mg, 0.85 mmol, 1 eq.; VPDU-255) was then dissolved in CH_2Cl_2 (9 mL) and added to the reaction mixture via cannula. Afterward, the reaction mixture was stirred for 68 hours at room temperature, wherein TLC analysis indicated a complete reaction. Next, the reaction was diluted with EtOAc and sequentially washed with sat. aq. $NaHCO_3$ solution, H_2O, and brine. The organic layer was then dried ($MgSO_4$), filtered, concentrated, and purified by silica gel flash column chromatography (elution with 100% EtOAc) to provide FFSMP **4d** (456 mg, 81%, VPDU-256) as a white foam.

R_f = 0.25 (100% EtOAc)

^{1}H NMR (400 MHz, $CDCl_3$, *peaks reported are for the major rotamer only) δ 7.94 (d, J = 8.3 Hz, 2H), 7.80 – 7.71 (m, 4H), 7.59 (t, J = 7.4 Hz, 1H), 7.49 (dd, J = 18.8, 7.9 Hz, 4H), 7.44 – 7.16 (m, 8H), 4.06 (s, 2H), 3.84 – 3.77 (m, 2H), 3.64 – 3.52 (m, 2H), 3.50 (s, 2H), 2.79 (d, J = 10.1 Hz, 2H), 2.47 (d, J = 2.5 Hz, 1H), 2.27 (dq, J = 34.8, 10.8, 9.4 Hz, 6H)

^{13}C NMR (126 MHz, $CDCl_3$, *peaks reported are for the major rotamer only) δ 195.7, 172.5, 171.6, 163.9, 139.6, 138.9, 138.6, 138.1, 136.9, 132.9, 131.2, 130.1, 130.0, 129.0, 128.8, 128.6, 128.5, 128.2, 127.1, 126.9, 80.2, 78.3, 74.1, 62.8, 53.7, 48.8, 38.5, 32.2, 29.7

HRMS (ESI+) m/z [M + H] calcd for $C_{39}H_{36}ClN_3O_5$ H^+ 662.2416, found 662.2424

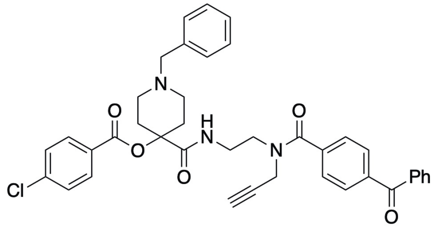

4d
(VPDU-256)
^{1}H NMR (400 MHz, $CDCl_3$ with TMS)

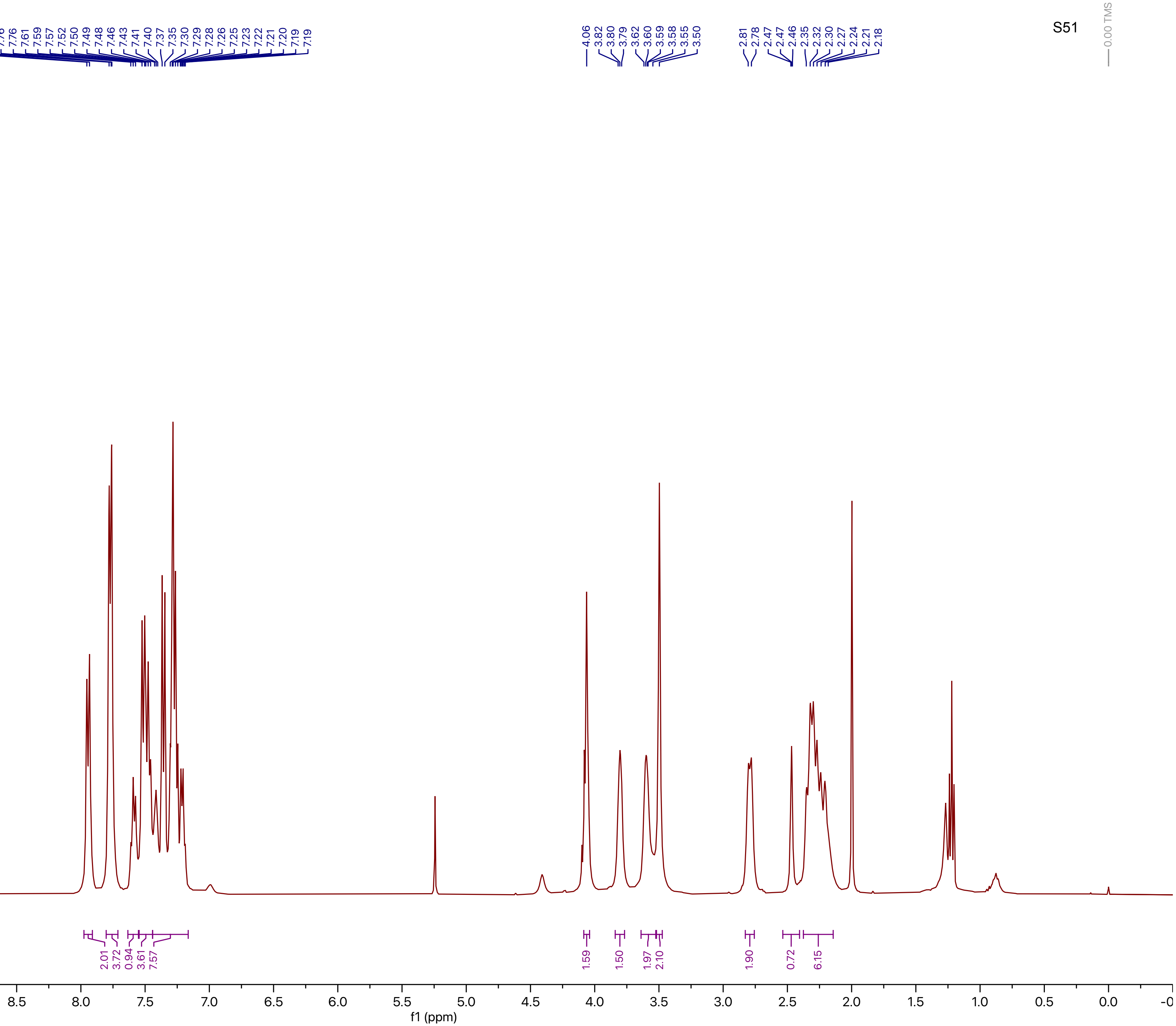

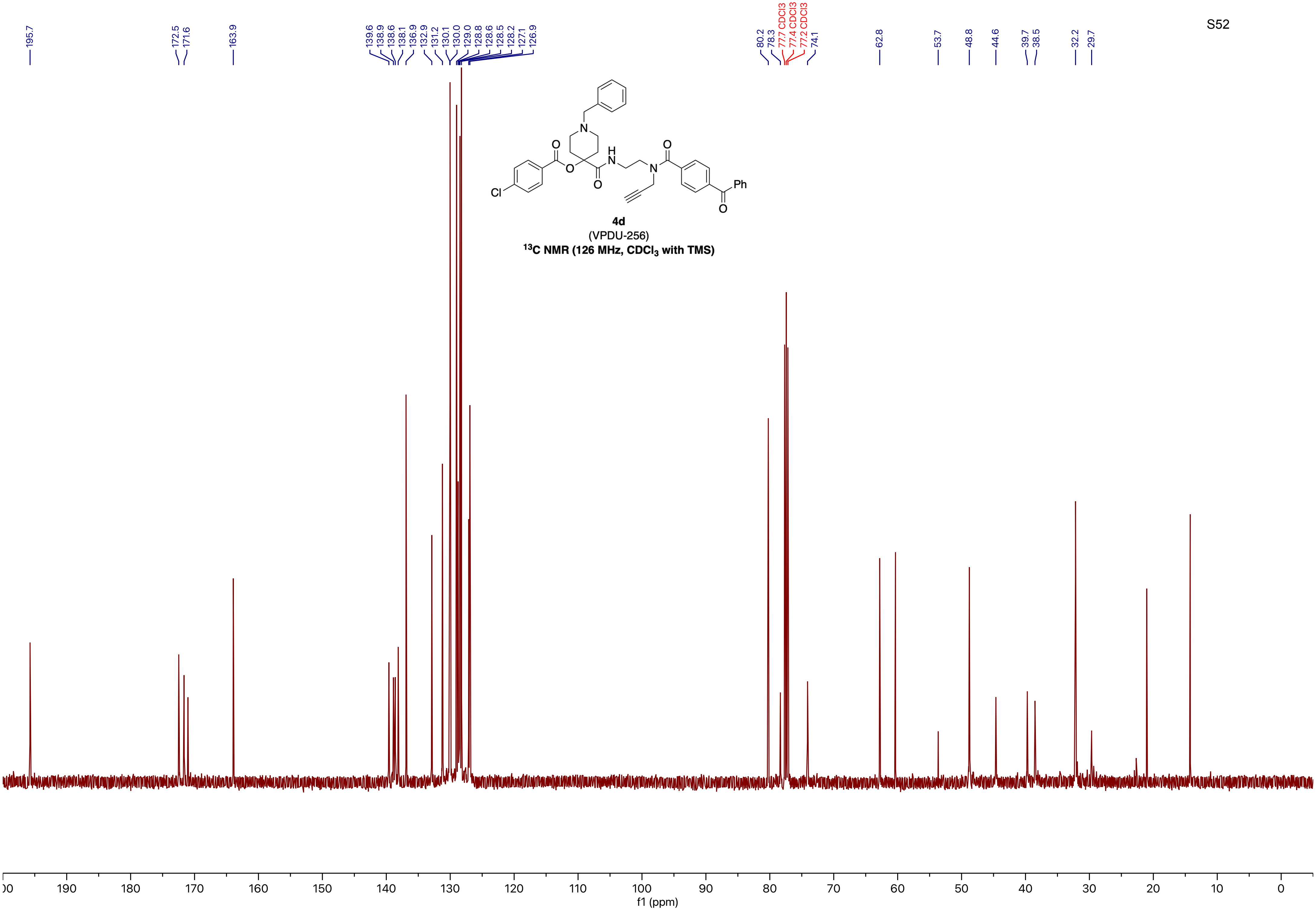

4d
(VPDU-256)
^{13}C NMR (126 MHz, $CDCl_3$ with TMS)

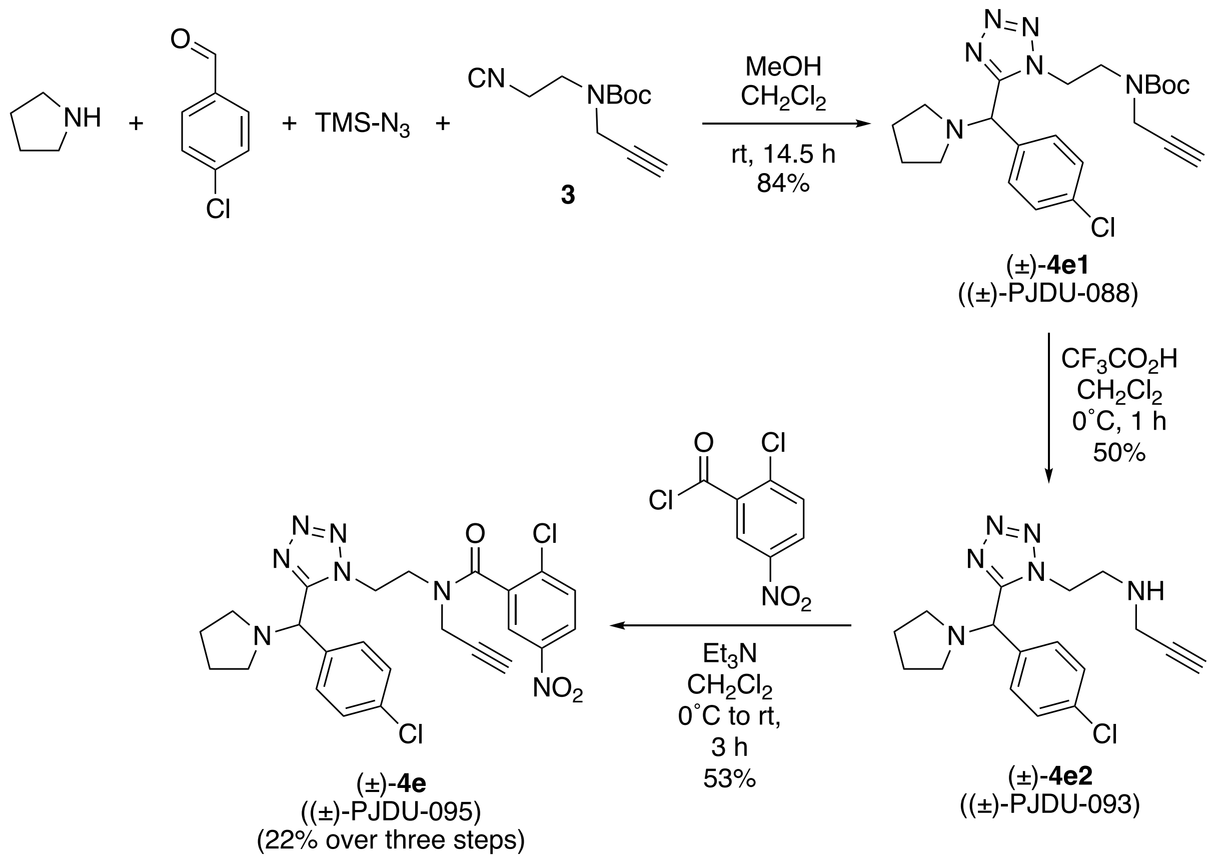

Scheme S4. *Synthesis of UT-4CR-based FFSMP (±)-**4e** ((±)-PJDU-095).*

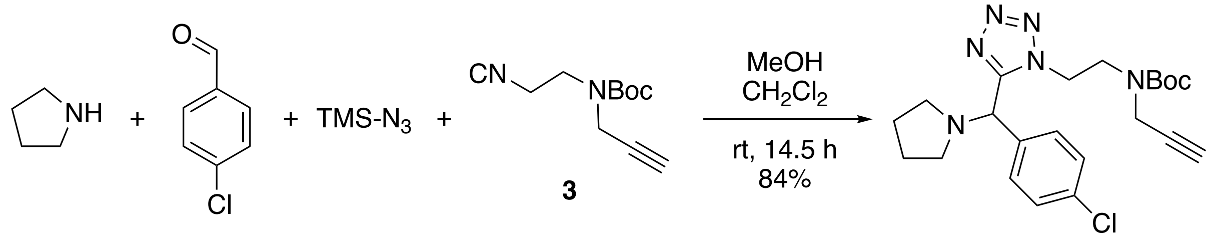

(±)-**4e1**
((±)-PJDU-088)

*(±)-tert-Butyl (2-(5-((4-chlorophenyl)(pyrrolidin-1-yl)methyl)-1H-tetrazol-1-yl)ethyl)(prop-2-yn-1-yl)carbamate ((±)-**4e1**) ((±)-PJDU-088).* 4-Chlorobenzaldehyde (55 mg, 0.39 mmol, 1.05 eq.) was initially dissolved in MeOH (0.8 mL) and then treated with pyrrolidine (0.035 mL, 0.39 mmol, 1.05 eq.). After stirring at room temperature for 15 minutes, isocyanide **3** (77 mg, 0.37 mmol, 1 eq.) was added all at once as a solution in CH_2Cl_2 (0.5 mL), followed immediately by dropwise addition of TMS-N_3 (0.055 mL, 0.39 mmol, 1.05 eq.). The reaction was then stirred at room temperature for 14.5 hours, wherein complete consumption of the isocyanide was observed by TLC. The reaction mixture was then concentrated and purified by silica gel flash chromatography (gradient elution with 10-40% EtOAc/hexanes) to provide UT-4CR adduct (±)-**4e1** (138 mg, 84%, (±)-PJDU-088) as an off-white, sticky solid.

R_f = 0.41 (50% EtOAc/hexanes)

^{1}H NMR (500 MHz, $CDCl_3$, *peaks reported are for the major rotamer only) δ 7.46 (d, *J* = 8.5 Hz, 2H), 7.32 (d, *J* = 8.5 Hz, 2H), 4.99 (s, 1H), 4.59 (td, *J* = 6.5, 3.6 Hz, 2H), 3.91 (s, 2H), 3.68 (t, *J* = 6.5 Hz, 2H), 2.64 – 2.53 (m, 2H), 2.53 – 2.39 (m, 2H), 2.18 (t, *J* = 2.5 Hz, 1H), 1.87 – 1.73 (m, 4H), 1.46 (s, 9H)

^{13}C NMR (101 MHz, $CDCl_3$, *peaks reported are for the major rotamer only) δ 155.4, 154.8, 135.1, 134.4, 129.9, 129.0, 81.4, 78.6, 72.1, 63.2, 52.4, 46.4, 45.4, 38.1, 28.3, 23.4

HRMS (ESI+) *m/z* [M + Na] calcd for $C_{22}H_{29}ClN_6O_2\ Na^+$ 467.1933, found 467.1933

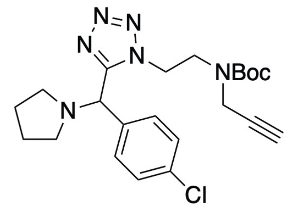

(±)-**4e1**
((±)-PJDU-088)
^{1}H NMR (500 MHz, $CDCl_3$ with TMS)

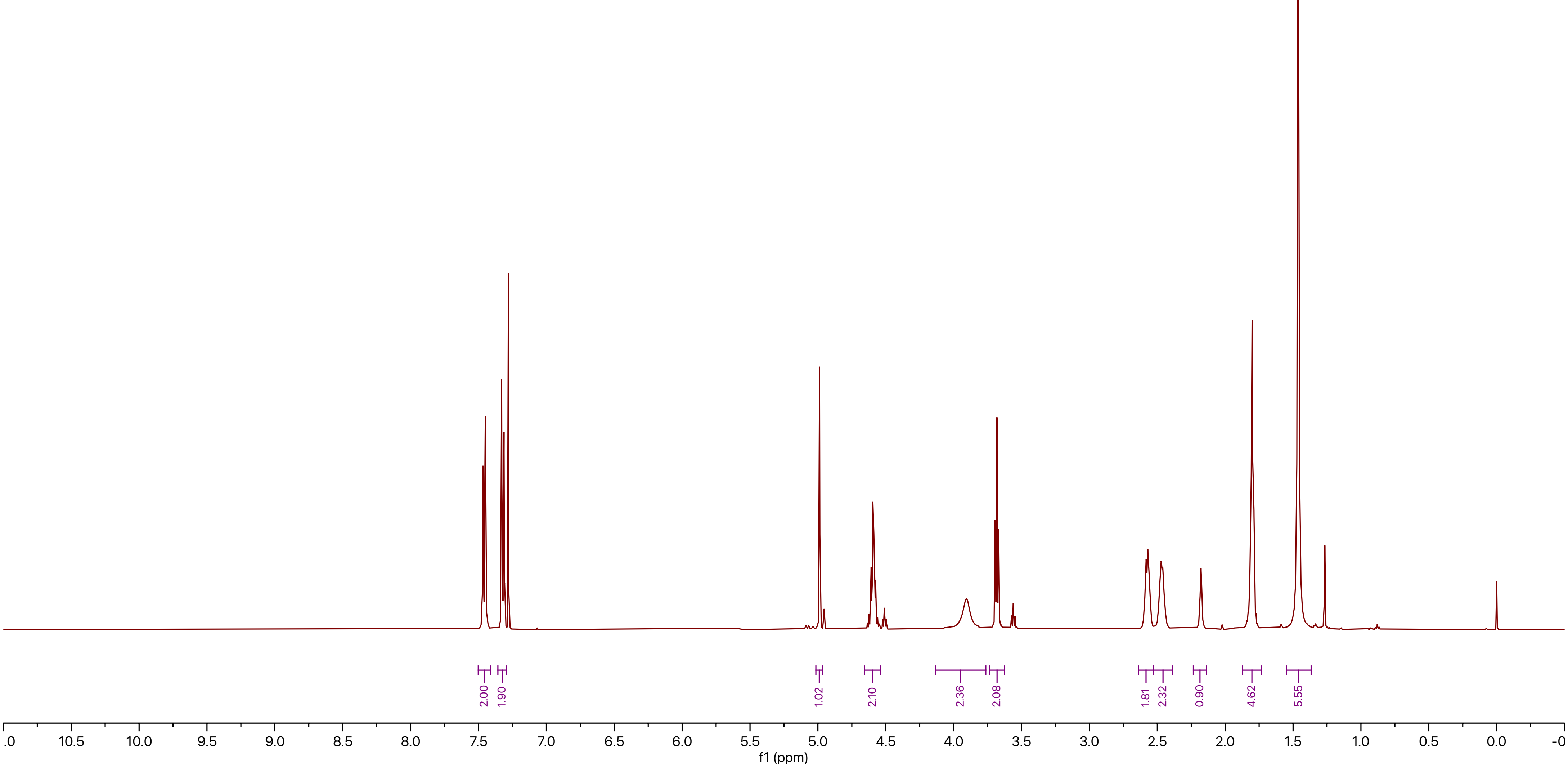

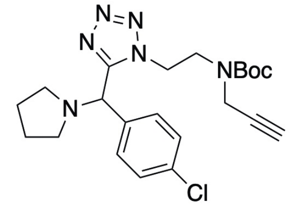

(±)-**4e1**
((±)-PJDU-088)
^{13}C NMR (101 MHz, $CDCl_3$ with TMS)

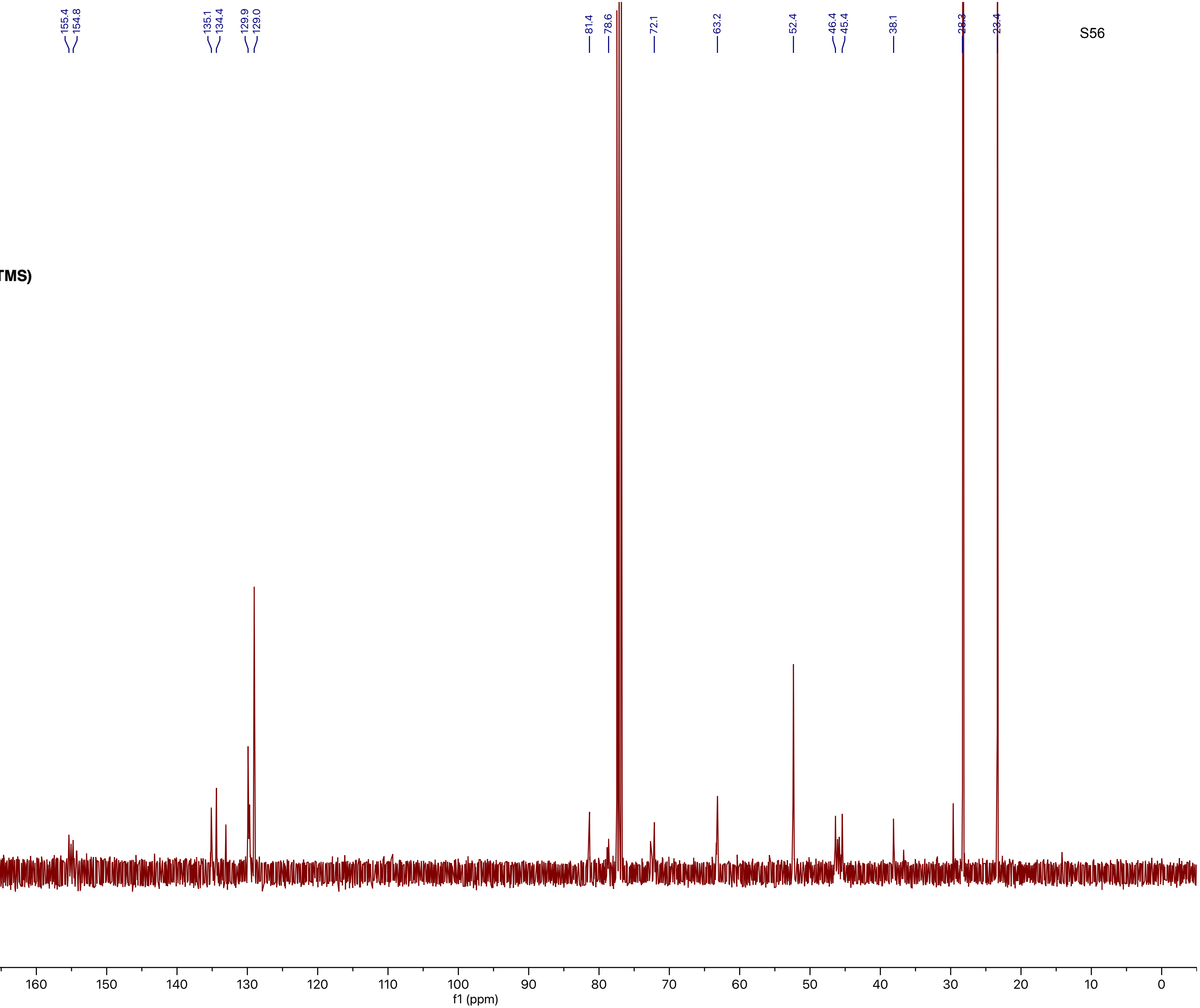

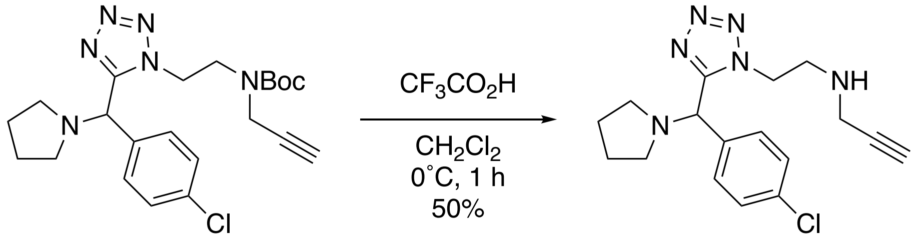

(±)-**4e1**
((±)-PJDU-088)

(±)-**4e2**
((±)-PJDU-093)

*(±)-N-(2-(5-((4-Chlorophenyl)(pyrrolidin-1-yl)methyl)-1H-tetrazol-1-yl)ethyl)prop-2-yn-1-amine ((±)-**4e2**) ((±)-PJDU-093).* A flame-dried, round-bottom flask equipped with a stirbar, septum, and argon inlet needle was charged with Boc carbamate substrate (±)-**4e1** (142 mg, 0.32 mmol, 1 eq.; (±)-PJDU-088) and CH_2Cl_2 (3.2 mL), then cooled to 0°C using a saturated ice-water bath. Trifluoroacetic acid (0.24 mL, 3.2 mmol, 10 eq.) was then added dropwise over 2 minutes. After the addition, the reaction was stirred at 0˚C for 1 hour, wherein TLC indicated complete consumption of the starting Boc carbamate substrate. The reaction was then quenched by dropwise addition of sat. aq. $NaHCO_3$ solution (~10 mL, vigorous gas evolution) until gas evolution ceased, then 1M aq. NaOH (~4 mL) was added to raise the pH to 8-9. The biphasic mixture was then transferred to a separatory funnel and diluted with CH_2Cl_2, the layers were separated, and the aqueous layer was extracted with CH_2Cl_2. The combined organic layers were then washed with brine, dried ($MgSO_4$), filtered, and concentrated to dryness, then purified by silica gel flash column chromatography (elution with 5% MeOH/$CHCl_3$) to give propargyl amine (±)-**4e2** (55 mg, 50%, (±)-PJDU-093) as a yellow oil.

R_f = 0.12 (5% MeOH/$CHCl_3$)

^{1}H NMR (500 MHz, $CDCl_3$, *peaks reported are for the major rotamer only) δ 7.45 (d, J = 8.3 Hz, 2H), 7.33 (d, J = 8.5 Hz, 2H), 5.02 (s, 1H), 4.50 (td, J = 6.2, 3.2 Hz, 2H), 3.38 (d, J = 2.4 Hz, 2H), 3.13 – 3.00 (m, 2H), 2.67 – 2.54 (m, 2H), 2.42 (ddt, J = 8.8, 4.3, 2.7 Hz, 2H), 2.21 (t, J = 2.4 Hz, 1H), 1.82 (td, J = 5.7, 4.8, 3.2 Hz, 4H)

^{13}C NMR (126 MHz, $CDCl_3$, *peaks reported are for the major rotamer only) δ 155.2, 135.3, 134.5, 129.8, 129.2, 81.3, 72.1, 63.6, 52.7, 47.7, 47.0, 38.0, 23.5

HRMS (ESI+) m/z [M + Na] calcd for $C_{17}H_{21}ClN_6$ Na^+ 367.1408, found 367.1413

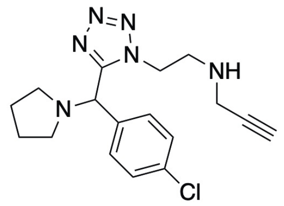

(±)-**4e2**
((±)-PJDU-093)
^{1}H NMR (500 MHz, $CDCl_3$ with TMS)

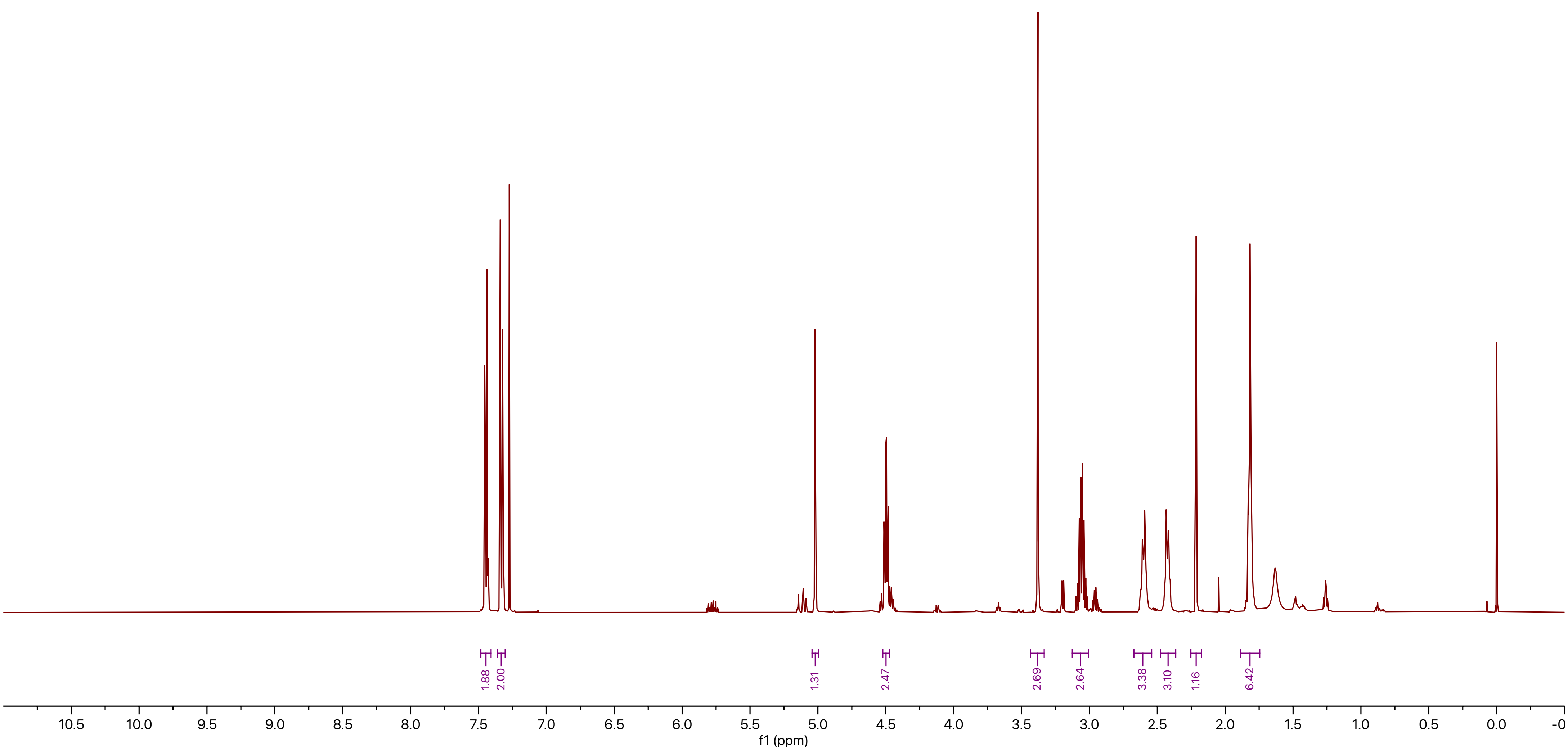

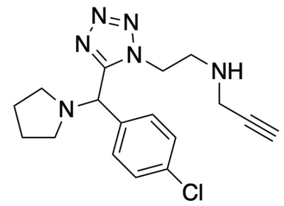

(±)-**4e2**
((±)-PJDU-093)
^{13}C NMR (126 MHz, $CDCl_3$ with TMS)

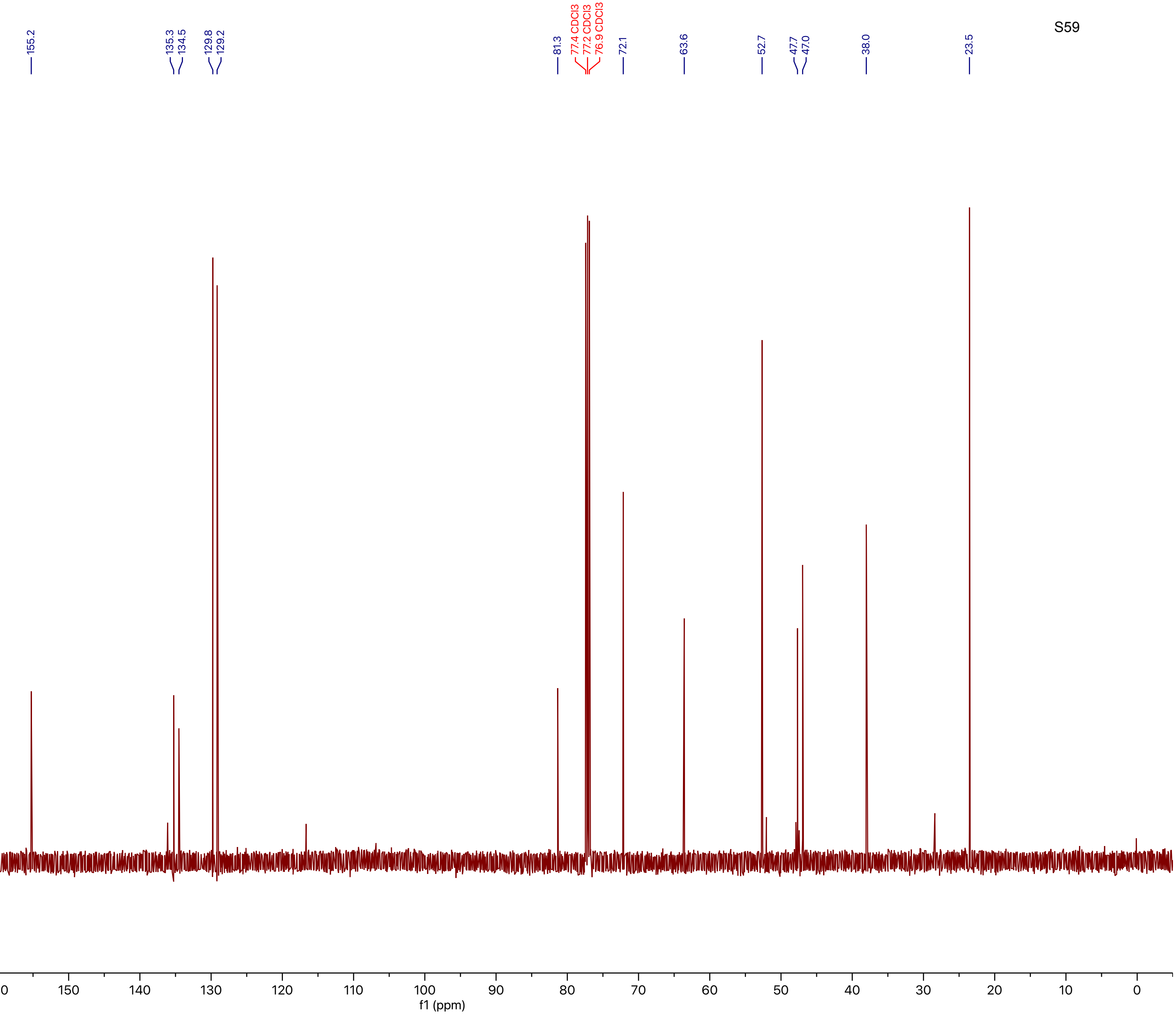

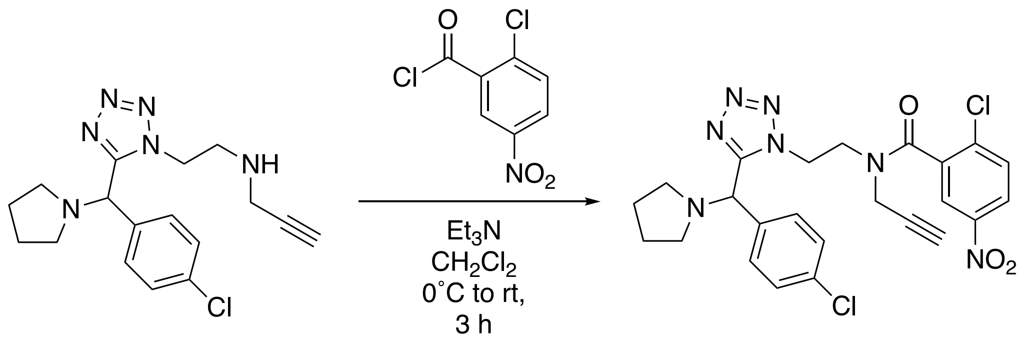

(±)-**4e2**
((±)-PJDU-093)

(±)-**4e**
((±)-PJDU-095)

*(±)-2-Chloro-N-(2-(5-((4-chlorophenyl)(pyrrolidin-1-yl)methyl)-1H-tetrazol-1-yl)ethyl)-5-nitro-N-(prop-2-yn-1-yl)benzamide ((±)-**4e**) ((±)-PJDU-095).* A flame-dried, 2-dram vial equipped with a stirbar, septum, and argon inlet needle was charged with 2˚ propargyl amine substrate (±)-**4e2** (20 mg, 0.06 mmol, 1 eq.; (±)-PJDU-093) and CH_2Cl_2 (1 mL), then cooled to 0°C with a saturated ice-water bath. Triethylamine (15 mL, 0.09 mmol, 1.5 eq.) was then added dropwise over 1 minute, followed by addition of a CH_2Cl_2 (1 mL) solution of 2-chloro-5-nitrobenzoyl chloride (19 mg, 0.09 mmol, 1.5 eq.; freshly prepared according to Xu, B.; Su, W. A Tandem Dehydrogenation-Driven Cross-Coupling between Cyclohexanones and Primary Amines for Construction of Benzoxazoles. *Angew Chem Int Ed Engl* **2022**, *61* (30), e202203365) via cannulation. The ice bath was then removed and the reaction mixture was allowed to warm to room temperature. The reaction mixture was stirred for 3 hours, wherein consumption of the starting 2˚ propargyl amine substrate was observed by TLC. Next, the reaction mixture was transferred to a separatory funnel and washed with H_2O. The aq. layer was then extracted with CH_2Cl_2. The combined organic layers were then washed with sat. aq. $NaHCO_3$ solution, dried ($MgSO_4$), filtered, and concentrated to produce a crude residue, which was purified by silica gel flash column chromatography (gradient elution with 25-50% EtOAc/hexanes) to give FFSMP (±)-**4e** (16 mg, 53%, ((±)-PJDU-095)) as a pale yellow oil.

R_f = 0.24 (50% EtOAc/hexanes)

^{1}H NMR (500 MHz, $CDCl_3$, *peaks reported are for the major rotamer only) δ 8.28 – 8.11 (m, 2H), 7.60 (d, J = 8.6 Hz, 1H), 7.56 – 7.47 (m, 2H), 7.34 (d, J = 8.4 Hz, 2H), 5.11 (s, 1H), 4.81 (t, J = 6.6 Hz, 2H), 4.01 – 3.57 (m, 4H), 2.62 (ddd, J = 8.9, 5.2, 2.9 Hz, 2H), 2.56 – 2.41 (m, 2H), 2.30 (t, J = 2.5 Hz, 1H), 1.83 (p, J = 3.1 Hz, 4H)

^{13}C NMR (126 MHz, $CDCl_3$, *peaks reported are for the major rotamer only) δ 166.7, 155.6, 147.0, 137.6, 135.1, 134.9, 131.2, 130.1, 129.3, 125.7, 123.6, 119.7, 76.9, 76.8, 74.8, 63.5, 52.5, 45.3, 44.7, 40.2, 23.7

HRMS (ESI+) m/z [M + H] calcd for $C_{24}H_{23}Cl_2N_7O_3$ H^+ 528.1312, found 528.1311

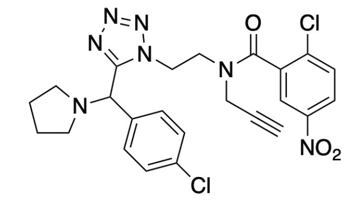

(±)-**4e**
((±)-PJDU-095)
^{1}H NMR (500 MHz, $CDCl_3$ with TMS)

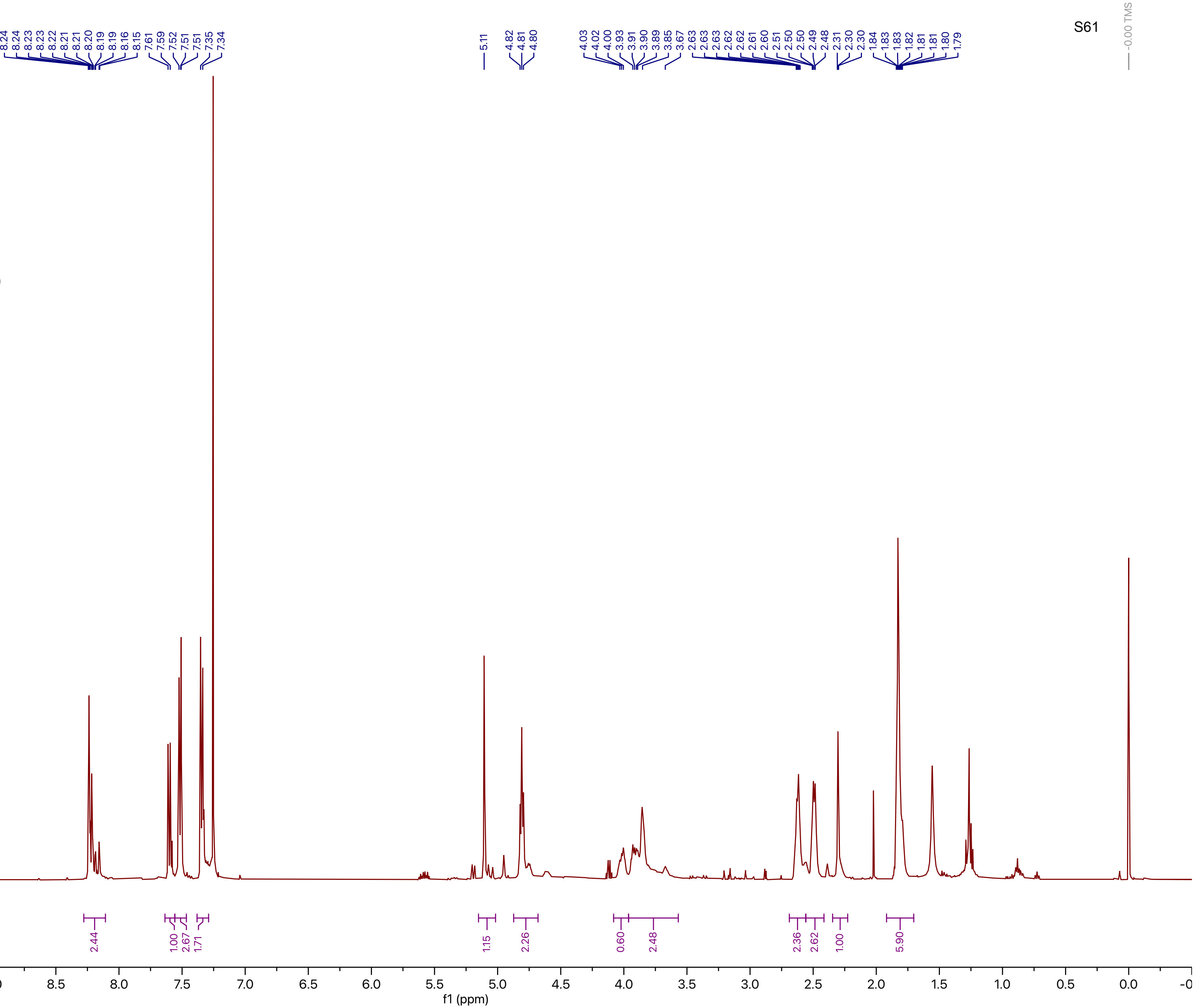

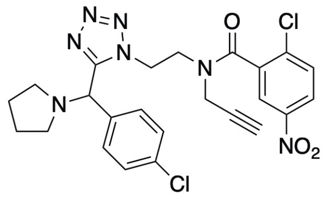

(±)-**4e**
((±)-PJDU-095)
^{13}C NMR (126 MHz, $CDCl_3$ with TMS)

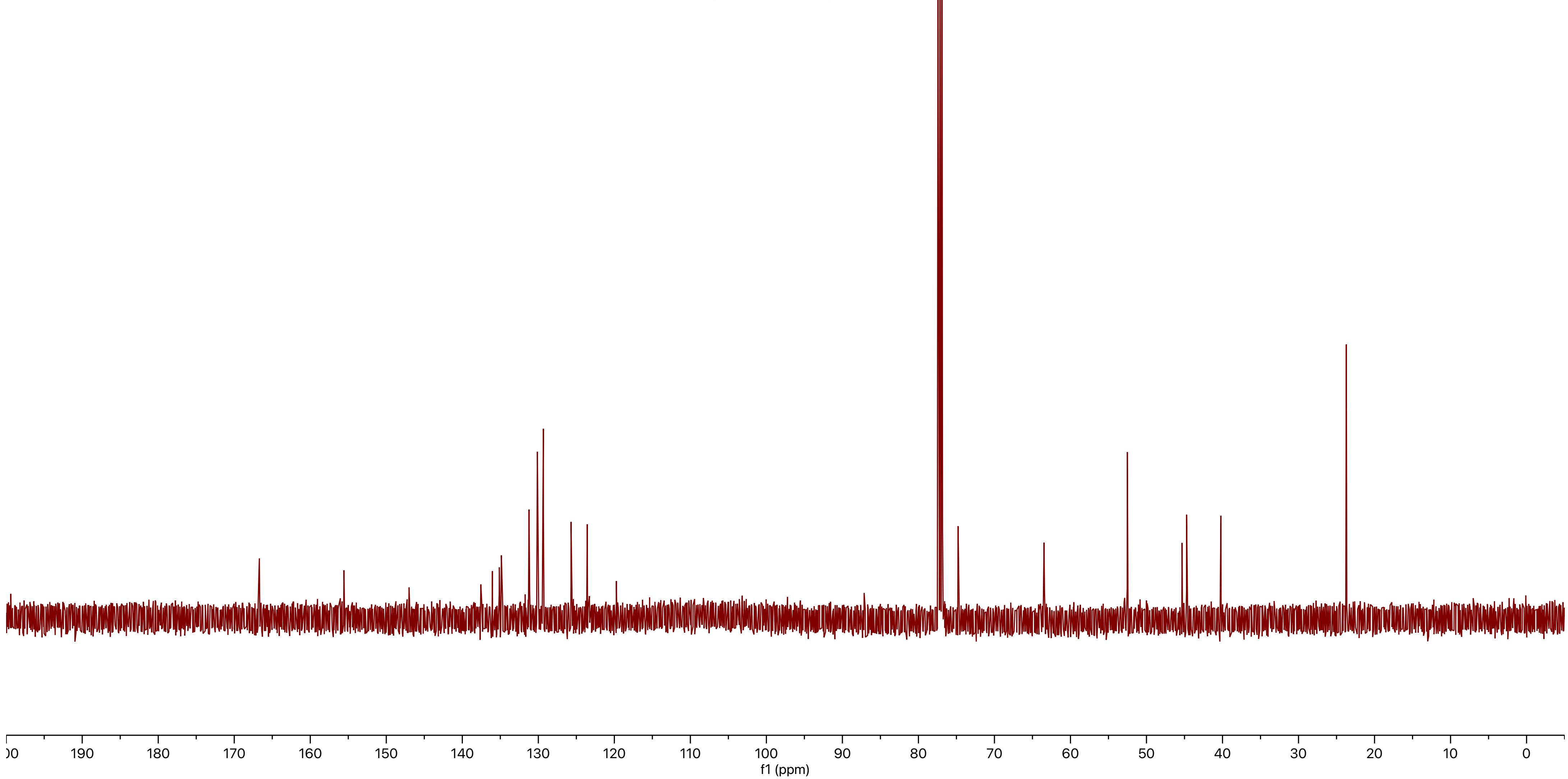

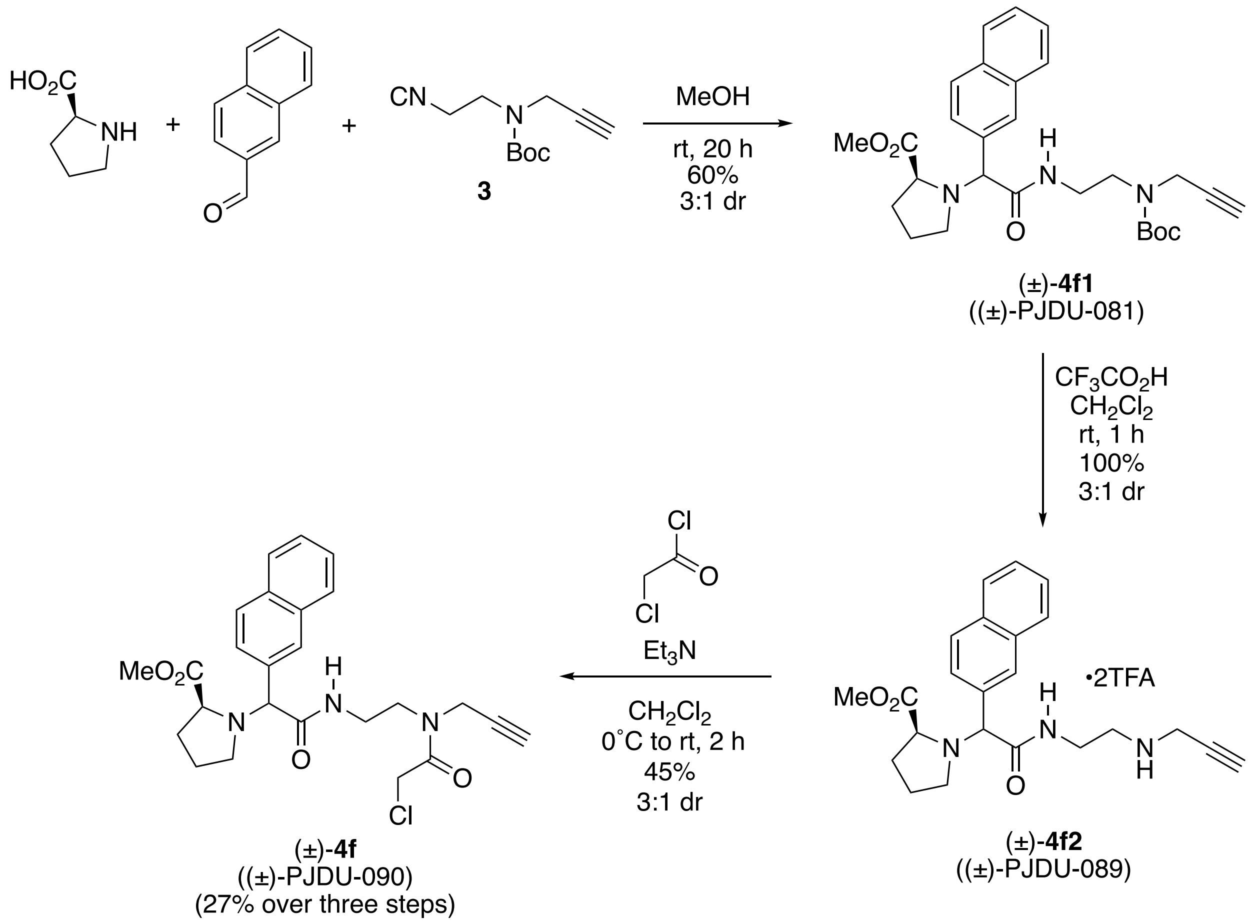

Scheme S5. *Synthesis of U-5C-4CR-based FFSMP (±)-**4f** ((±)-PJDU-90).*

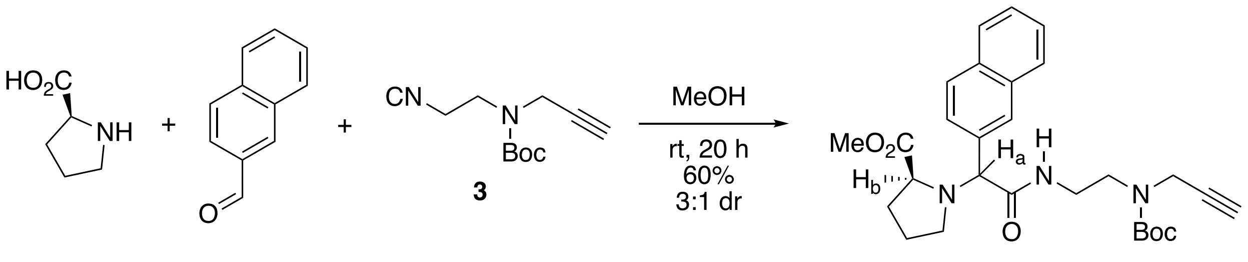

(±)-**4f1**
((±)-PJDU-081)

*(±)-Methyl (2-((2-((tert-butoxycarbonyl)(prop-2-yn-1-yl)amino)ethyl)amino)-1-(naphthalen-2-yl)-2-oxoethyl)-L-prolinate ((±)-**4f1**) ((±)-PJDU-081).* L-Proline (52 mg, 0.45 mmol, 1.1 eq.) was dissolved in MeOH (1 mL). Next, 2-napthaldehyde (71 mg, 0.45 mmol, 1.1 eq.) was added all at once and the mixture was stirred at room temperature for 15 minutes. Isocyanide **3** (86 mg, 0.41 mmol, 1 eq.) dissolved in CH_2Cl_2 (0.5 mL) was then added all at once and the reaction was stirred at room temperature for 20 hours, wherein a complete reaction was observed by TLC. The crude reaction mixture was concentrated via rotary evaporation and purified by silica gel flash column chromatography (gradient elution with 25-55% EtOAc/hex) to provide U5C-4CR product (±)-**4f1** (123 mg, 60%, 3:1 dr) as a light yellow, sticky solid. The diastereomeric ratio was determined by integration of the ^{1}H NMR resonances for H_a at 4.57 (minor) and 4.40 (major) ppm and the resonances for H_b at 3.24 (major) and 2.99 (minor) ppm.

R_f = 0.15 (50% EtOAc/hexanes)

1H NMR (400 MHz, $CDCl_3$, *peaks reported are for the major rotamer and major diastereomer only) δ 7.95 (t, J = 5.4 Hz, 1H), 7.86 – 7.69 (m, 4H), 7.52 – 7.32 (m, 3H), 4.40 (s, 1H), 4.24 – 3.85 (m, 2H), 3.74 – 3.46 (m, 6H), 3.38 (s, 2H), 3.24 (s, 1H), 2.73 (s, 1H), 2.33 (dd, J = 64.0, 28.2 Hz, 1H), 2.04 (d, J = 5.7 Hz, 1H), 1.86 (tdd, J = 14.0, 7.7, 3.7 Hz, 3H), 1.49 (s, 9H)

^{13}C NMR (101 MHz, $CDCl_3$, *peaks reported are for the major rotamer and major diastereomer only) δ 172.1, 171.8, 153.8, 133.2, 133.2, 128.9, 128.7, 128.3, 128.1, 128.0, 127.6, 127.6, 126.2, 80.7, 79.7, 73.3, 62.1, 51.5, 45.9, 37.9, 36.9, 31.9, 30.6, 29.7, 28.4, 24.1

HRMS (ESI+) *m/z* [M + H] calcd for $C_{28}H_{35}N_3O_5$ H^+ 494.2649, found 494.2650

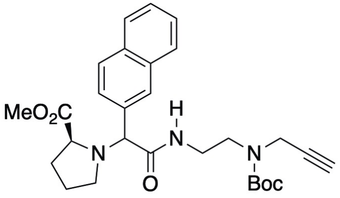

(±)-**4f1**
((±)-PJDU-081)
^{1}H NMR (400 MHz, $CDCl_3$ with TMS)

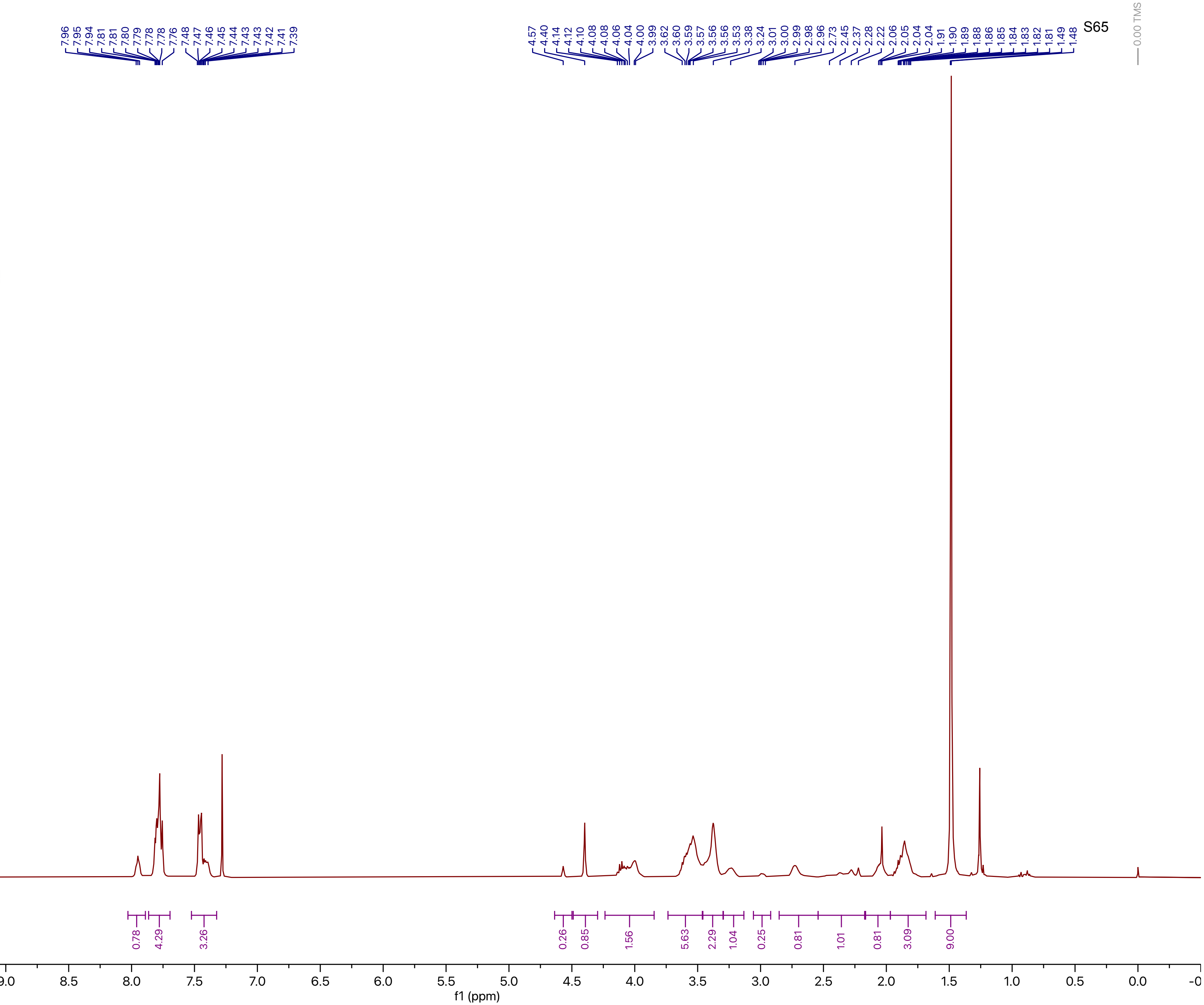

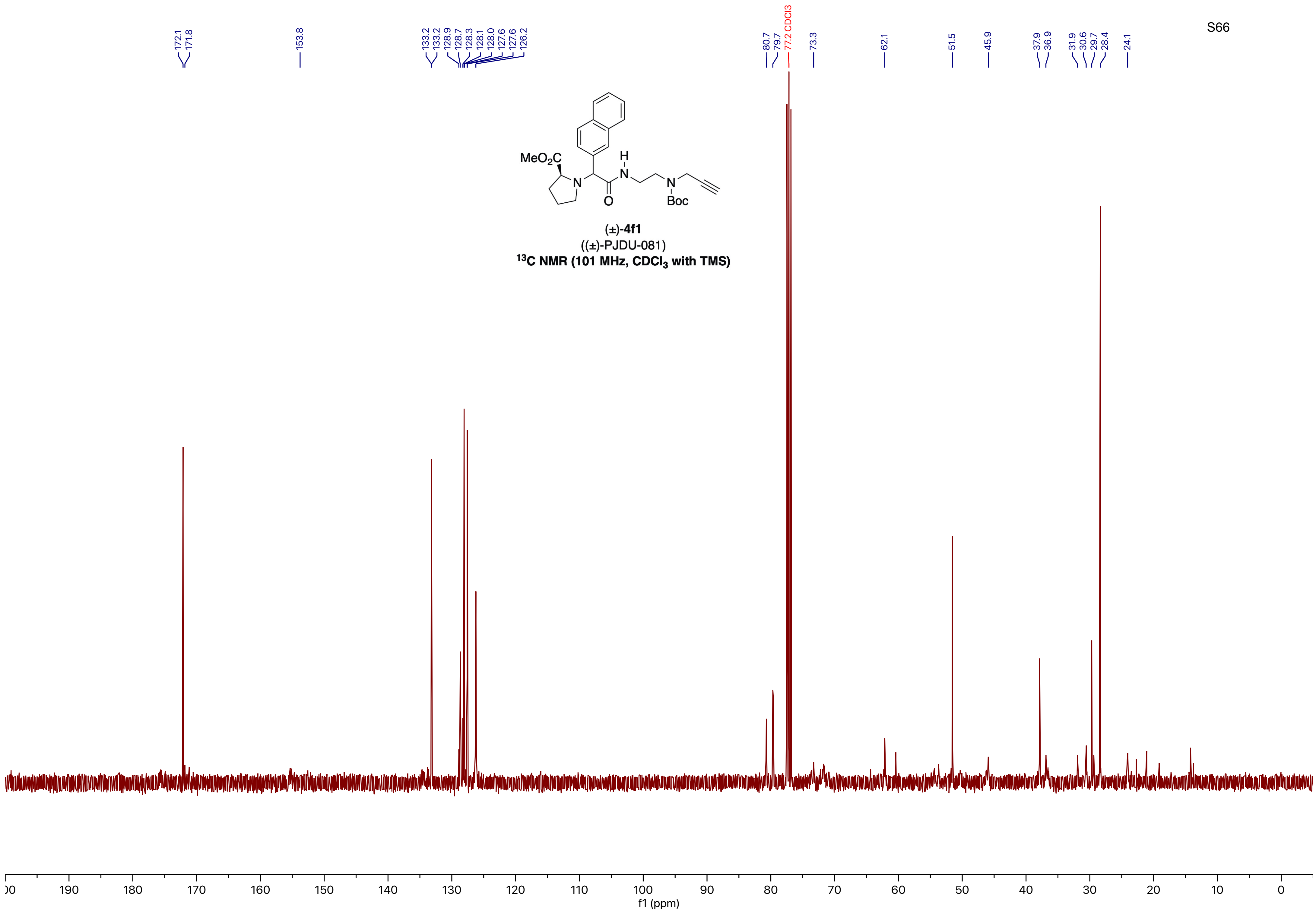

(±)-**4f1**
((±)-PJDU-081)
^{13}C NMR (101 MHz, $CDCl_3$ with TMS)

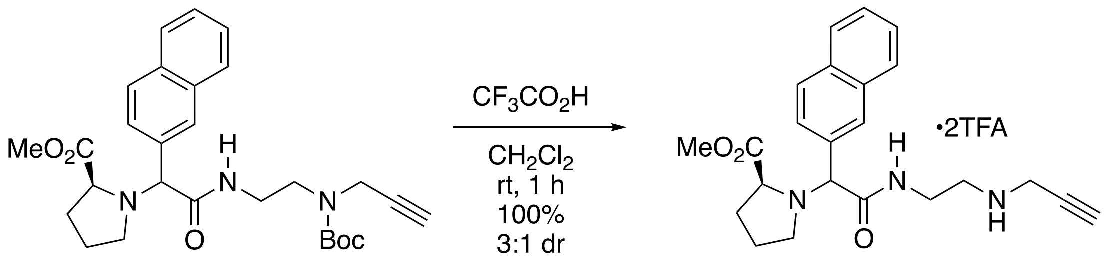

(±)-**4f1**
((±)-PJDU-081)

(±)-**4f2**
((±)-PJDU-089)

*(±)-Methyl (1-(naphthalen-2-yl)-2-oxo-2-((2-(prop-2-yn-1-ylamino)ethyl)amino)ethyl)-L-prolinate ((±)-**4f2**) ((±)-PJDU-089).* A flame-dried, round-bottom flask equipped with a stirbar, septum, and argon inlet needle was charged with Boc carbamate substrate (±)-**4f1** (120 mg, 0.24 mmol, 1 eq.; (±)-PJDU-081) and CH_2Cl_2 (2.5 mL). Trifluoroacetic acid (0.19 mL, 2.4 mmol, 10 eq.) was then added dropwise over 2 minutes. After the addition, the reaction was stirred at room temperature for 1 hour, wherein TLC indicated complete consumption of the starting Boc carbamate substrate. The reaction was then concentrated to dryness with the aid of toluene to give the crude di-trifluoroacetate salt of propargyl amine (±)-**4f2** (189 mg, 100%, (±)-PJDU-089) as a reddish-brown oil.

^{1}H NMR (400 MHz, $CDCl_3$) of the crude material clearly indicated disappearance of the Boc carbamate (i.e., disappearance of the 9H *t*-Bu signal at 1.49 ppm for Boc carbamate substrate (±)-**4f1**)

HRMS analysis of the crude material cleared for the free base form of propargyl amine (±)-**4f2** (HRMS (ESI+) *m*/*z* [M + H] calcd for $C_{23}H_{27}N_3O_3$ H^+ 394.2125, found 394.2126).

In turn, the crude di-trifluoroacetate salt of propargyl amine (±)-**4f2** was used as is for the subsequent N-acylation reaction and not characterized any further.

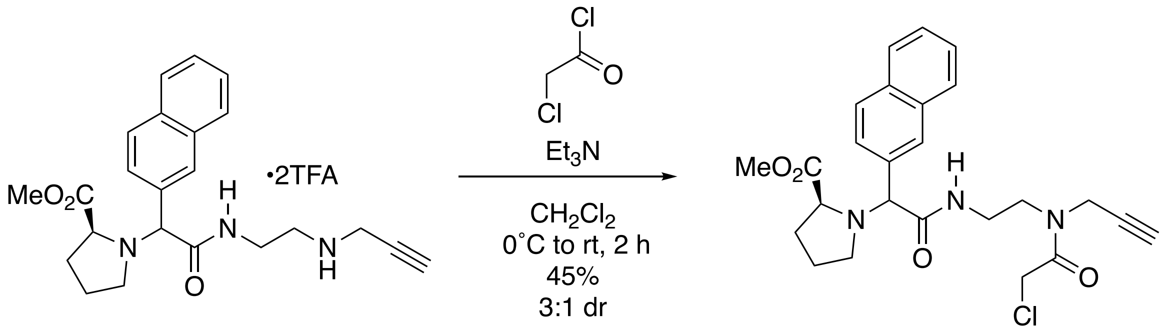

(±)-**4f2**
((±)-PJDU-089)

(±)-**4f**
((±)-PJDU-090)

*(±)-Methyl (2-((2-(2-chloro-N-(prop-2-yn-1-yl)acetamido)ethyl)amino)-1-(naphthalen-2-yl)-2-oxoethyl)-L-prolinate ((±)-**4f**) ((±)-PJDU-090).* A flame-dried, round-bottom flask equipped with a stirbar, septum, and argon inlet needle was charged with the crude di-trifluoroacetate salt of 2˚ propargyl amine (±)-**4f2** (138 mg, 0.27 mmol, 1 eq.; (±)-PJDU-089) and CH_2Cl_2 (3 mL), then cooled to 0°C with a saturated ice-water bath. Triethylamine (0.15 mL, 1.1 mmol, 4 eq.) was then added dropwise over 1 minute, followed by dropwise addition of chloroacetyl chloride (0.03 mL, 0.33 mmol, 1.2 eq.) over 1 minute. The ice bath was then removed and the reaction mixture was allowed to warm to room temperature. The reaction mixture was stirred for 2 hours and then transferred to a separatory funnel and washed with H_2O. The aq. layer was then extracted with CH_2Cl_2. The combined organic layers were then then washed with H_2O and brine sequentially, dried ($MgSO_4$), filtered, and concentrated to produce a crude residue that was purified by silica gel flash column chromatography (gradient elution with 60-90% EtOAc/hexanes) to provide 58 mg of FFSMP (±)-**4f** (45%, 3:1 dr) as a pale yellow oil.

R_f = 0.23 (75% EtOAc/hexanes)

1H NMR (400 MHz, $CDCl_3$, 1:1 mixture of diastereomers based on integration of these peaks*) δ 8.47 (s, 1H*, amide N-H), 8.17 (s, 1H*, amide NH), 7.93 – 7.65 (m, 4H), 7.49 (td, *J* = 6.2, 2.9 Hz, 2H), 7.36 (dd, *J* = 8.4, 1.8 Hz, 1H*), 7.29 (dd, *J* = 8.5, 1.9 Hz, 1H*), 4.51 (d, *J* = 8.8 Hz, 1H), 4.29 (qd, *J* = 17.6, 2.6 Hz, 1H), 4.20 – 4.07 (m, 2H), 4.06 – 3.85 (m, 1H), 3.85 – 3.48 (m, 4H), 3.41 (s, 3H*, methyl ester, 3.38 (s, 3H*, methyl ester), 3.36 – 3.21 (m, 1H), 2.88 – 2.70 (m, 1H), 2.45 (t, *J* = 2.5 Hz, 1H*, terminal alkyne), 2.36 (t, *J* = 2.4 Hz, 1H*, terminal alkyne), 2.18 – 1.71 (m, 5H)

^{13}C NMR (101 MHz, $CDCl_3$, 1:1 mixture of diastereomers, reported peaks are for the highest peak intensity for a given individual carbon) δ 167.4, 167.4, 166.9, 133.3, 128.9, 128.5, 128.2, 127.8, 126.7, 126.7, 126.6, 126.5, 126.2, 78.5, 73.1, 61.8, 55.3, 51.9, 46.6, 41.5, 37.6, 37.0, 34.9, 30.6, 24.5

HRMS (ESI+) *m/z* [M + H] calcd for $C_{25}H_{28}Cl_1N_3O_4\,H^+$ 470.1841, found 470.1844

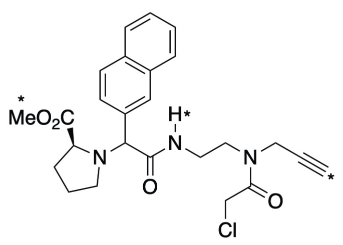

(±)-**4f**
((±)-PJDU-090)
^{1}H NMR (400 MHz, $CDCl_3$ with TMS)
- 1:1 mixture of diastereomers based on integration values for the following*:
a.) amide N-H triplet peaks at 8.47 and 8.17 ppm
b.) methyl ester singlet peaks at 3.41 and 3.38 ppm
c.) terminal alkyne triplet peaks at 2.45 and 2.36 ppm

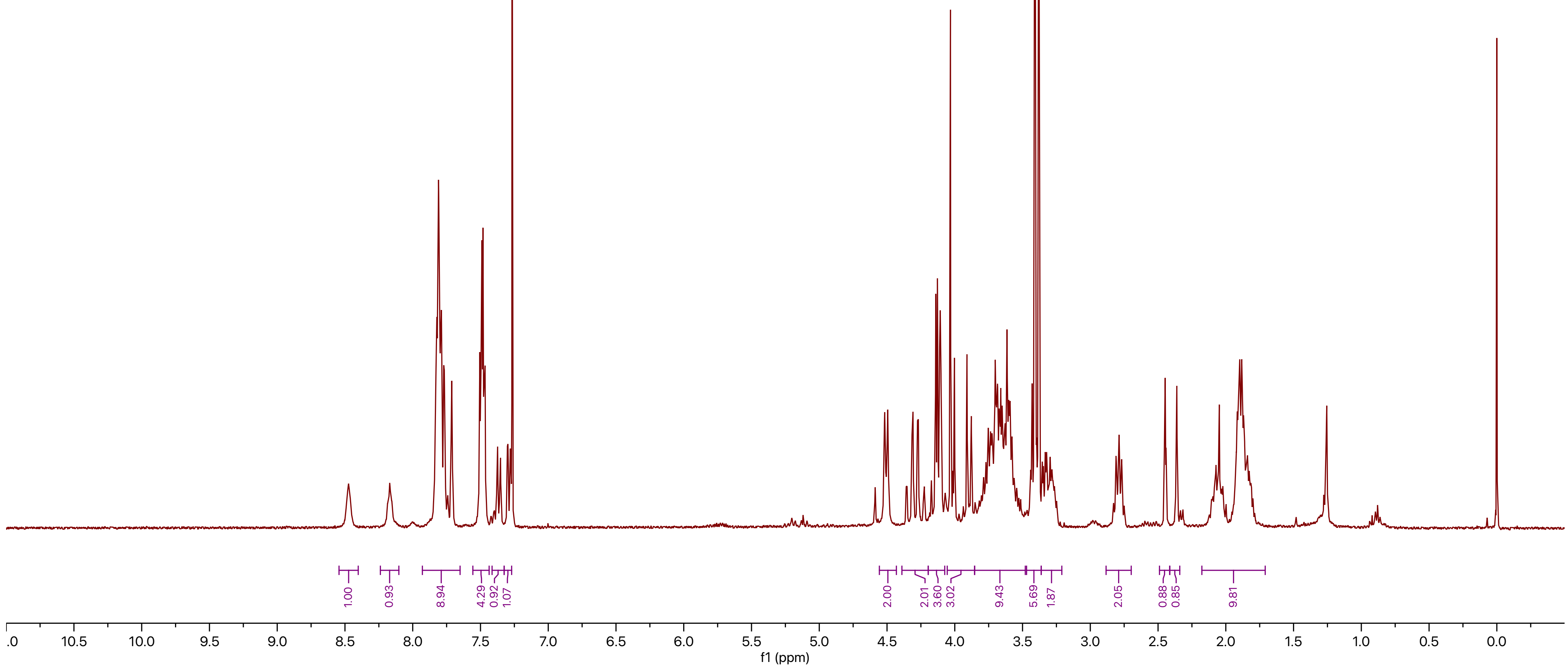

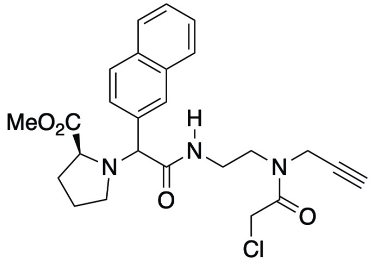

(±)-**4f**

((±)-PJDU-090)

^{13}C NMR (101 MHz, $CDCl_3$ with TMS)

- 1:1 mixture of diastereomers
- reported peaks are for the highest peak intensity for a given individual carbon

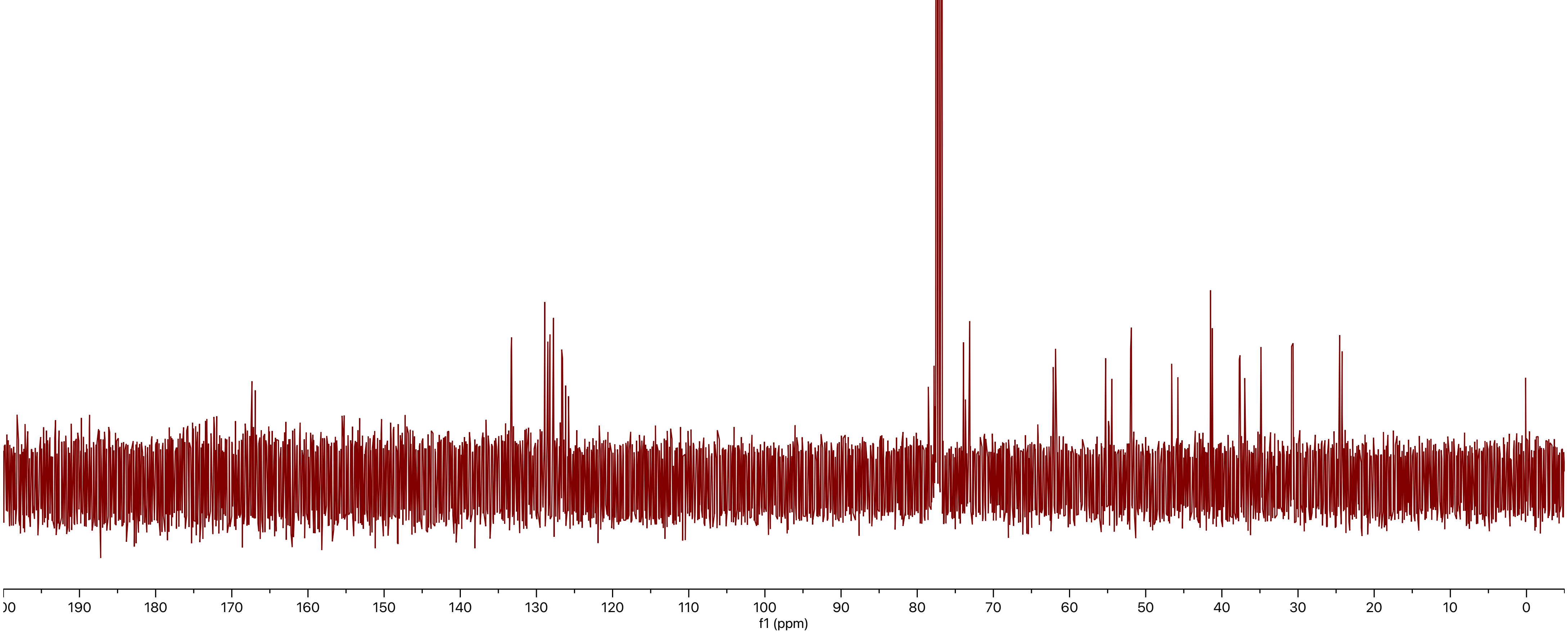

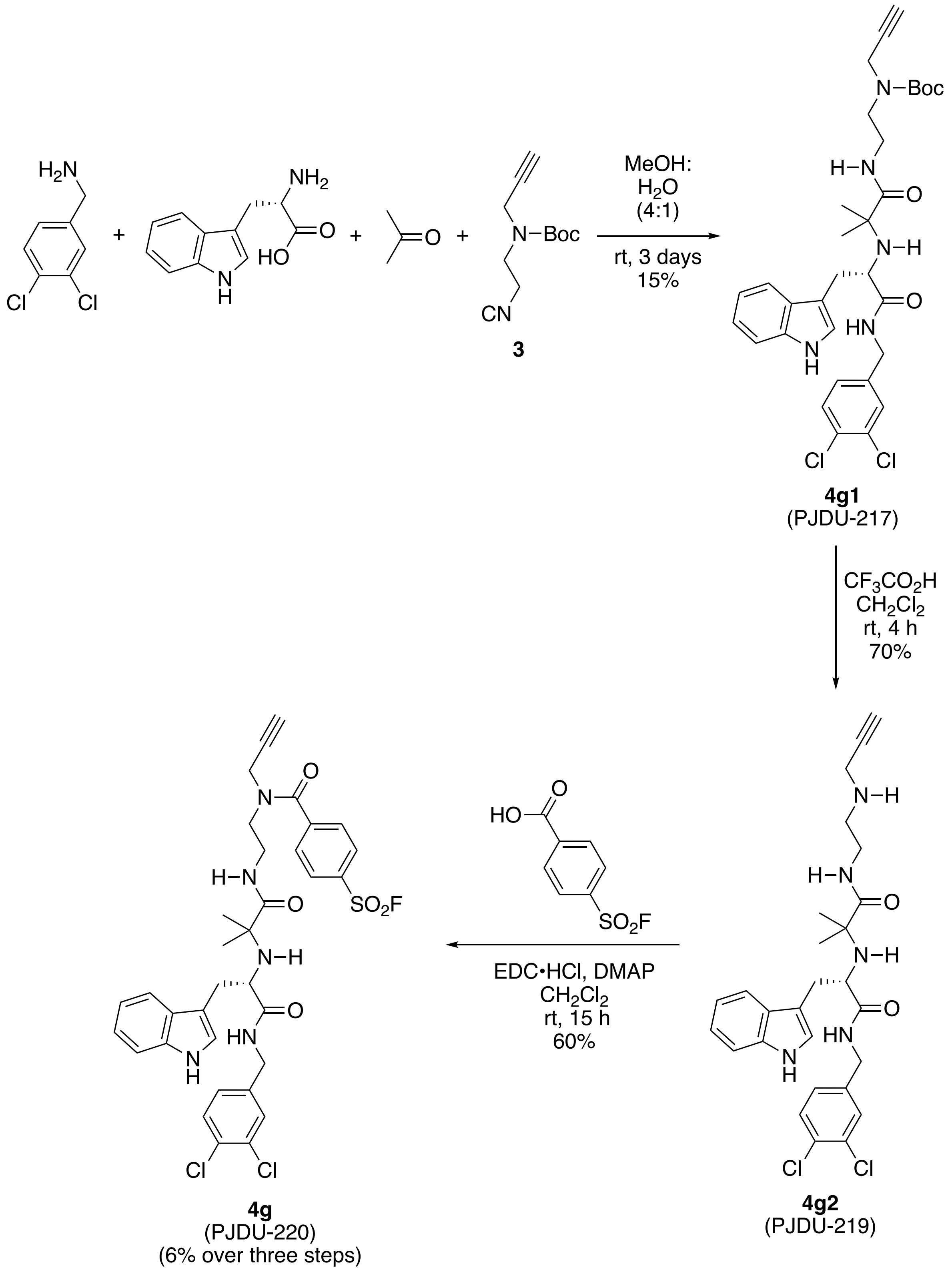

Scheme S6. *Synthesis of U-5C-4CR-based FFSMP* ***4g*** *(PJDU-220).*

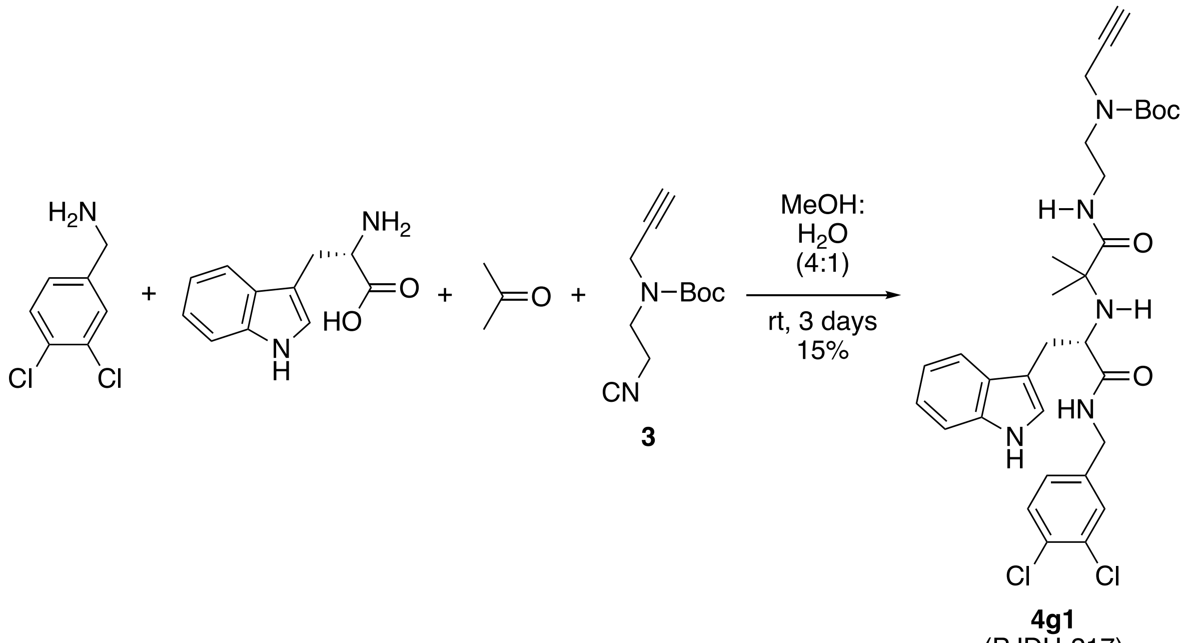

*tert-Butyl (S)-(2-(2-((1-((3,4-dichlorobenzyl)amino)-3-(1H-indol-3-yl)-1-oxopropan-2-yl)amino)-2-methylpropanamido)ethyl)(prop-2-yn-1-yl)carbamate (**4g1**) (PJDU-217).* A round-bottom flask equipped with a stir-bar was charged with isocyanide **3** (210 mg, 1.0 mmol, 1 eq.). MeOH and water (4:1, 10 mL) were added, followed by addition of L-tryptophan (216 mg, 1.05 mmol, 1.05 equiv). Next, acetone (0.08 mL, 1.1 mmol, 1.1 eq.) was added followed by addition of 3,4-dichlorobenzylamine (0.14 mL, 1.05 mmol, 1.05 eq.). The round-bottom flask was then capped with a septum and stirred at room temperature for 3 days. The reaction mixture was then concentrated under reduced pressure, diluted with CH_2Cl_2 (50 mL), dried ($MgSO_4$), and vacuum filtered through Celite to remove any remaining L-tryptophan. The filtrate was then concentrated under reduced pressure and purified by silica gel flash column chromatography (gradient elution with 50-80% EtOAc/hex) to give U-5C-4CR adduct **4g1** (93 mg, 15%; PJDU-217) as a clear colorless oil.

R_f = 0.26 (75% EtOAc/hexanes)

1H NMR (400 MHz, $CDCl_3$) δ 8.85 (s, 1H), 7.76 (s, 1H), 7.65 (d, *J* = 7.9 Hz, 1H), 7.36 (dd, *J* = 8.2, 4.8 Hz, 2H), 7.32 – 7.21 (m, 2H), 7.21 – 7.13 (m, 1H), 7.08 (td, *J* = 7.5, 1.1 Hz, 1H), 7.02 (d, *J* = 2.3 Hz, 1H), 7.00 – 6.84 (m, 1H), 4.38 (t, *J* = 5.2 Hz, 1H), 4.34 – 4.19 (m, 1H), 4.01 – 3.85 (m, 2H), 3.39 (d, *J* = 17.8 Hz, 2H), 3.31 – 3.09 (m, 3H), 2.99 (dd, *J* = 14.4, 8.4 Hz, 1H), 2.89 (s, 1H), 2.22 (t, *J* = 2.5 Hz, 1H), 1.95 (s, 1H), 1.46 (s, 9H), 1.13 (s, 3H), 0.92 (s, 3H)

^{13}C NMR (101 MHz, $CDCl_3$) δ 176.9, 175.9, 161.4, 156.3, 139.0, 136.5, 132.4, 131.1, 130.5, 129.7, 127.4, 127.1, 123.6, 122.3, 119.7, 118.9, 111.6, 81.2, 72.0, 60.5, 59.4, 58.6, 45.7, 42.2, 41.0, 39.2, 37.2, 28.4, 23.6, 21.1

HRMS (ESI+) *m/z* [M + Na] calcd for $C_{32}H_{39}Cl_2N_5O_4$ Na^+ 650.2271, found 650.2272

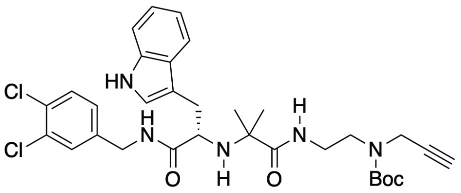

4g1
(PJDU-217)
^{1}H NMR (400 MHz, CDCl$_3$ with TMS)

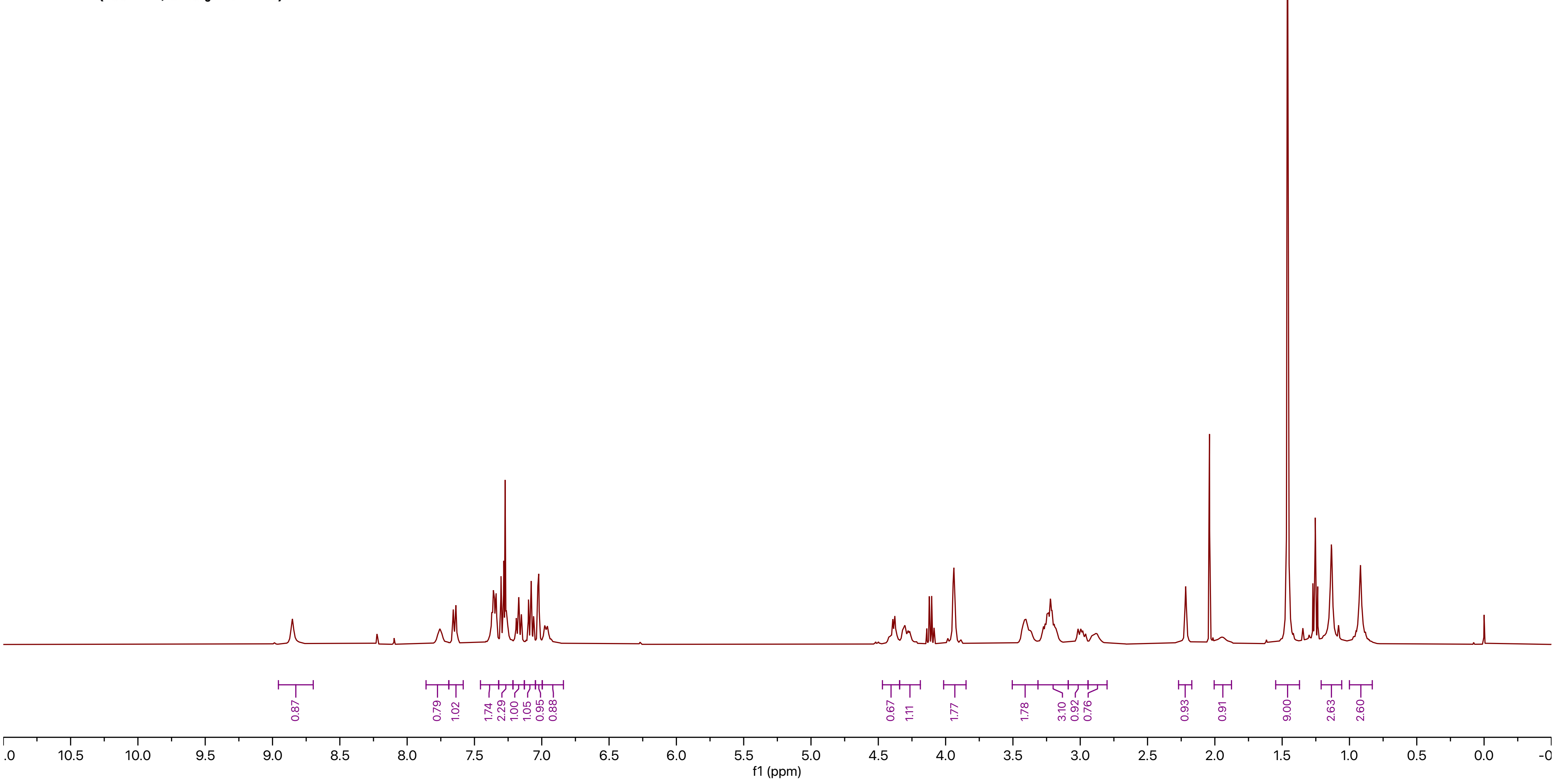

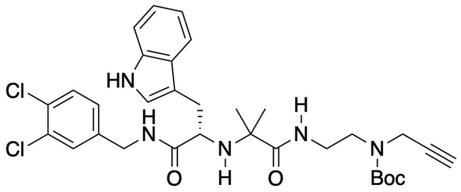

4g1
(PJDU-217)
^{13}C NMR (101 MHz, $CDCl_3$ with TMS)

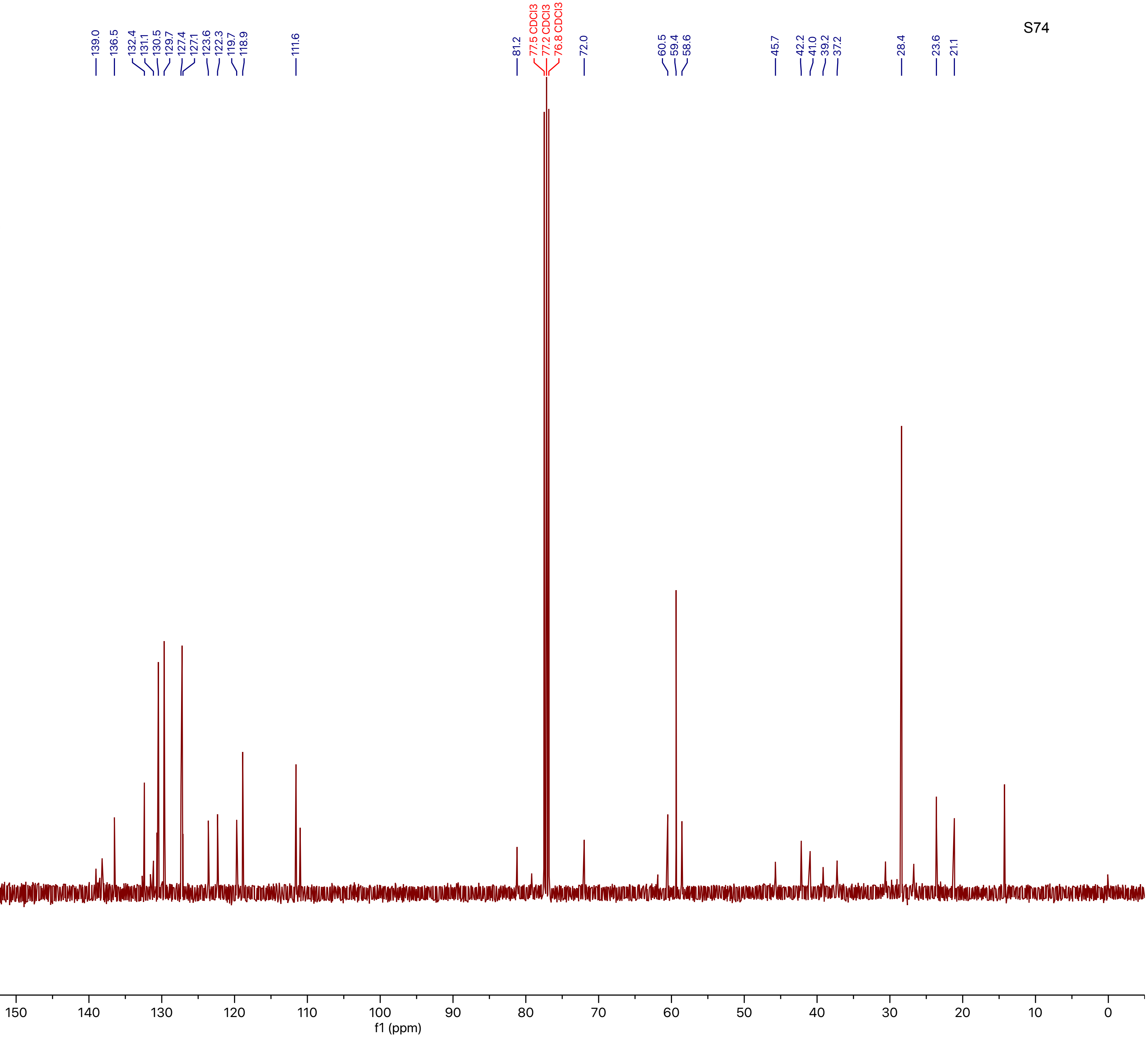

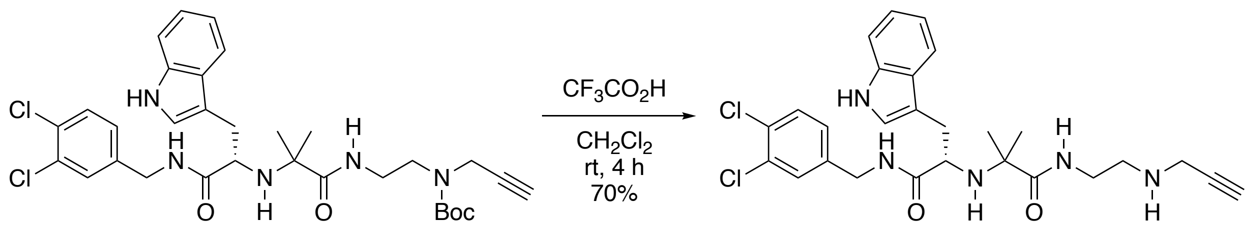

*(S)-N-(3,4-Dichlorobenzyl)-3-(1H-indol-3-yl)-2-((2-methyl-1-oxo-1-((2-(prop-2-yn-1-ylamino)ethyl)amino)propan-2-yl)amino)propanamide (**4g2**) (PJDU-219).* A flame-dried, round-bottom flask equipped with a stirbar, septum, and argon inlet needle was charged with Boc carbamate substrate **4g1** (90 mg, 0.14 mmol, 1 eq.; PJDU-217) and CH_2Cl_2 (1.4 mL). Trifluoroacetic acid (0.13 mL, 1.7 mmol, 12 eq.) was then added dropwise over 2 minutes. After the addition, the reaction was stirred at room temperature for 4 hours, wherein TLC indicated complete consumption of the starting Boc carbamate substrate. The reaction was then quenched by dropwise addition of sat. aq. $NaHCO_3$ (vigorous gas evolution) to the reaction mixture until gas evolution ceased (~10 mL), then 1M aq. NaOH (~4 mL) was added to raise the pH to 8-9 (checked with pH paper). This biphasic mixture was then transferred to a separatory funnel and diluted with CH_2Cl_2 (15 mL), the layers were separated, and the aqueous layer was extracted with CH_2Cl_2 (2 x 15 mL). The organic layers were combined, washed with brine (10 mL), dried ($MgSO_4$), gravity filtered, and concentrated under reduced pressure to give propargyl amine **4g2** (52 mg, 70%, PJDU-219) as a pale yellow oil that was used without further purification.

R_f = 0.35 (5% MeOH/ CH_2Cl_2)

1H NMR (500 MHz, $CDCl_3$) δ 8.64 (d, *J* = 2.4 Hz, 1H), 7.65 (d, *J* = 7.9 Hz, 1H), 7.37 (d, *J* = 7.9 Hz, 1H), 7.28 (d, *J* = 8.3 Hz, 1H), 7.22 – 7.13 (m, 3H), 7.12 – 7.07 (m, 1H), 7.03 (d, *J* = 2.3 Hz, 1H), 6.86 (dd, *J* = 8.2, 2.1 Hz, 2H), 4.34 – 4.17 (m, 2H), 3.37 (dd, *J* = 7.3, 5.7 Hz, 1H), 3.29 (d, *J* = 2.4 Hz, 2H), 3.20 – 3.01 (m, 3H), 2.88 – 2.74 (m, 1H), 2.53 (td, *J* = 5.9, 2.2 Hz, 2H), 2.20 (t, *J* = 2.4 Hz, 1H), 1.92 (m, 1H), 1.22 (s, 3H), 1.10 (s, 3H)

^{13}C NMR (126 MHz, $CDCl_3$) δ 176.8, 175.8, 161.3, 138.6, 136.4, 132.5, 131.3, 130.8, 129.6, 127.4, 127.2, 123.7, 122.4, 119.9, 119.0, 111.2, 81.7, 72.0, 59.4, 58.7, 47.4, 42.4, 38.6, 37.7, 31.0, 28.0, 23.8

HRMS (ESI+) *m/z* [M + Na] calcd for $C_{27}H_{31}Cl_2N_5O_2\ Na^+$ 550.1747, found 550.1752

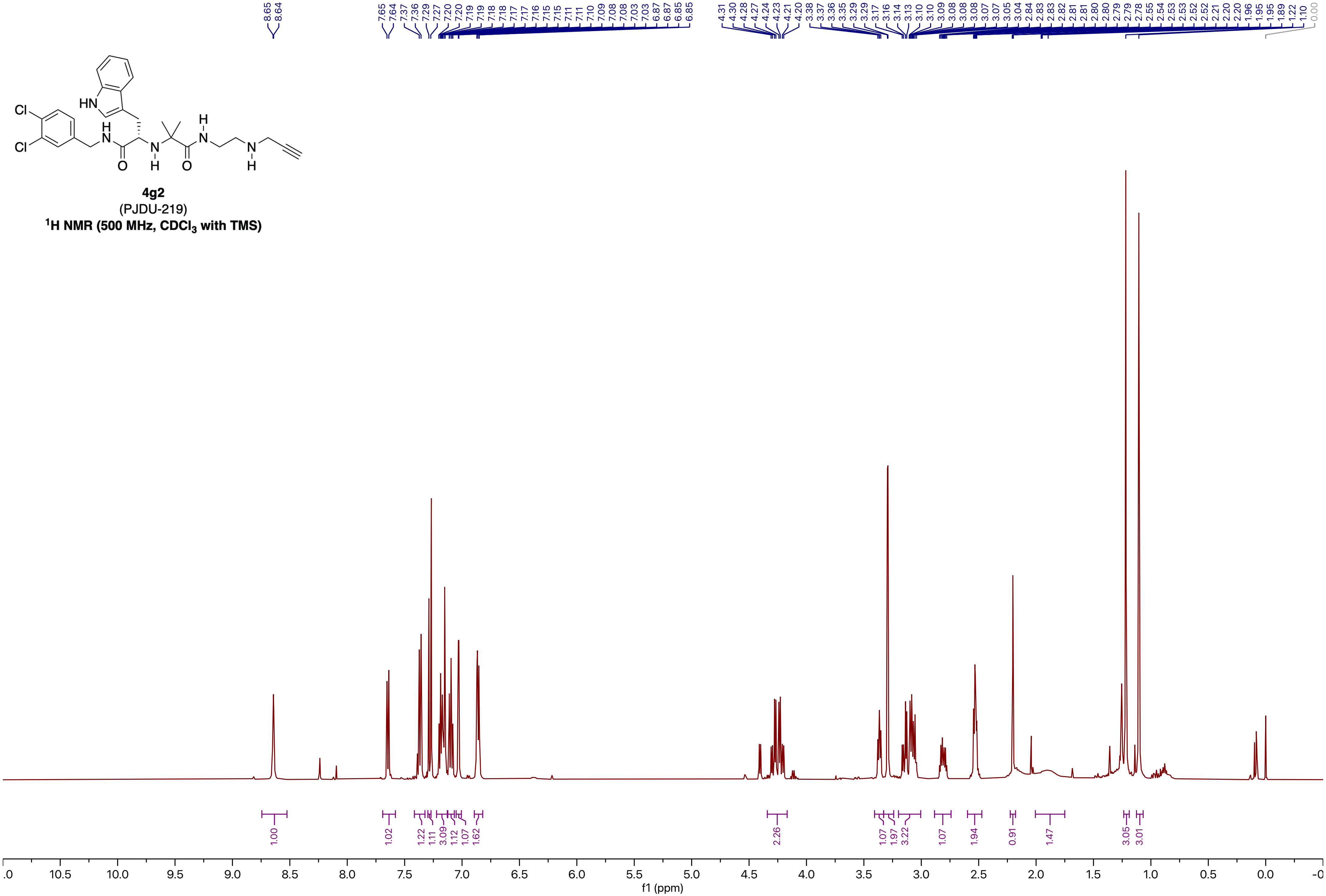

4g2
(PJDU-219)
^{1}H NMR (500 MHz, $CDCl_3$ with TMS)

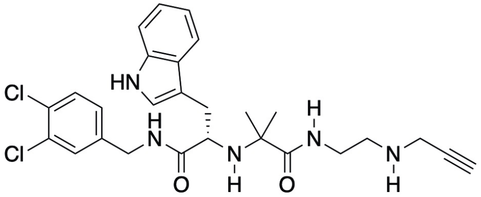

4g2
(PJDU-219)
^{13}C NMR (126 MHz, $CDCl_3$ with TMS)

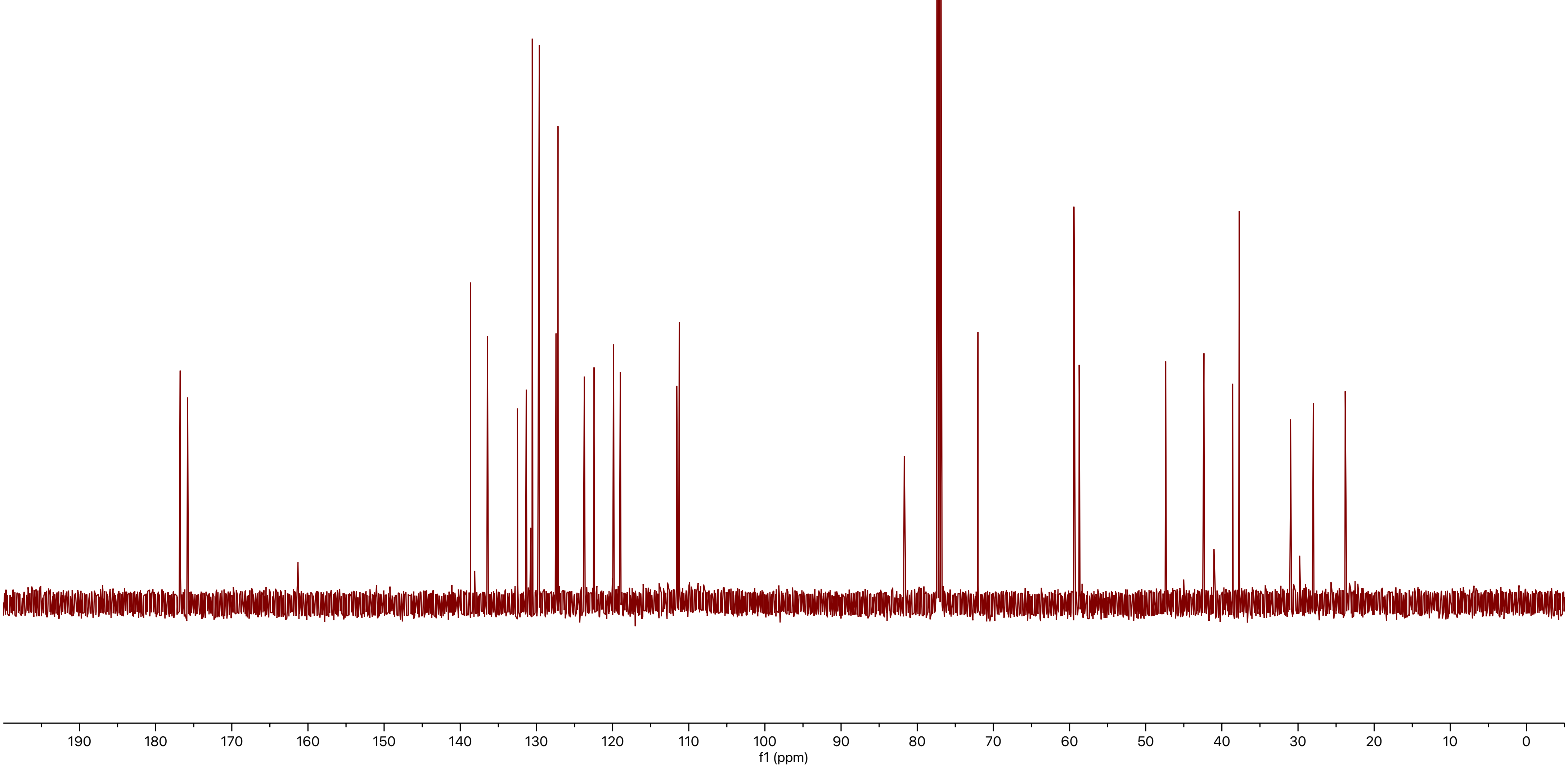

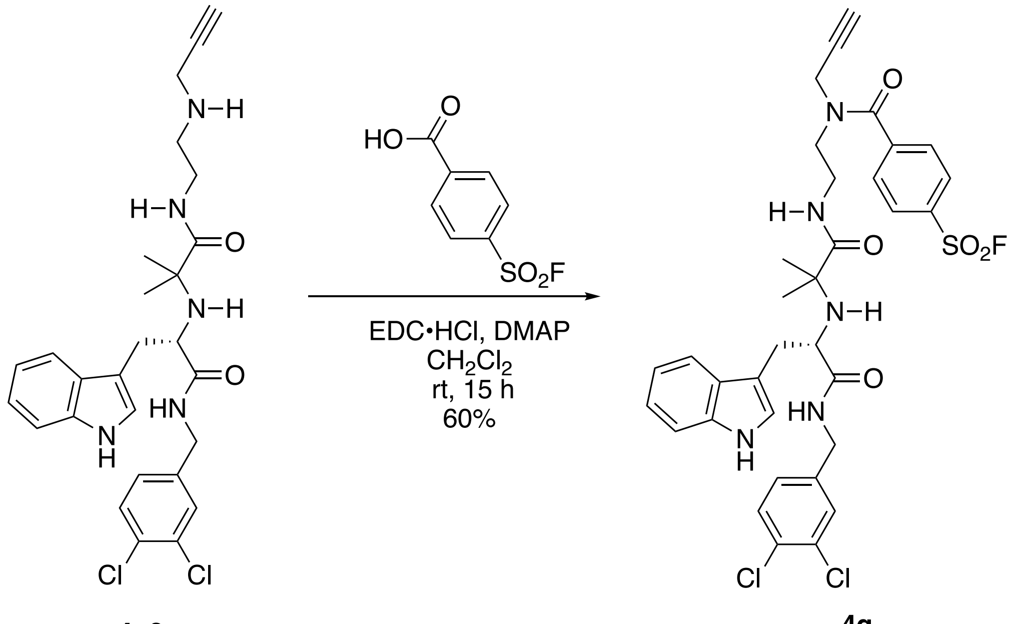

4g2
(PJDU-219)

4g
(PJDU-220)

*(S)-4-((2-(2-((1-((3,4-Dichlorobenzyl)amino)-3-(1H-indol-3-yl)-1-oxopropan-2-yl)amino)-2-methylpropanamido)ethyl)(prop-2-yn-1-yl)carbamoyl)benzenesulfonyl fluoride (**4g**) (PJDU-220).* A flame-dried, round-bottom flask equipped with a stir-bar, septum, and argon inlet needle was charged with 4-fluorosulfonyl benzoic acid (18 mg, 0.14 mmol, 1.5 eq.) and CH_2Cl_2 (1 mL). Next, EDC•HCl (36 mg, 0.19 mmol, 2 eq.) was added in a single portion followed by addition of DMAP (1 mg, 0.01 mmol, 0.1 eq.) in a single portion. Next, propargyl amine **4g2** (50 mg, 0.095 mmol, 1 eq.; PJDU-219) was dissolved in CH_2Cl_2 (1.4 mL)and transferred to the reaction mixture via cannula. The reaction was stirred for 15 hours at room temperature then concentrated to dryness. The crude mixture was then diluted with EtOAc (50 mL), then washed sequentially with sat. aq. $NaHCO_3$ (10 mL), deionized H_2O (10 mL), and brine (10 mL). The organic layer was then dried ($MgSO_4$), vacuum filtered, concentrated under reduced pressure, and purified by silica gel flash column chromatography (gradient elution with 70-90% EtOAc/hex) to give FFSMP **4g** (41 mg, 60%; PJDU-220) as a white, sticky solid.

R_f = 0.46 (75% EtOAc/hexanes)

1H NMR (400 MHz, $CDCl_3$) δ 8.51 (s, 1H), 8.01 (d, *J* = 8.4 Hz, 2H), 7.71 (d, *J* = 7.9 Hz, 1H), 7.59 (d, *J* = 8.1 Hz, 2H), 7.45 (t, *J* = 6.2 Hz, 1H), 7.42 – 7.33 (m, 1H), 7.29 – 7.17 (m, 3H), 7.17 – 7.06 (m, 2H), 7.06 – 6.96 (m, 1H), 6.89 (dd, *J* = 8.3, 2.1 Hz, 1H), 4.26 (qd, *J* = 14.9, 6.0 Hz, 2H), 3.90 (qd, *J* = 18.3, 2.5 Hz, 2H), 3.49 (ddd, *J* = 14.0, 8.5, 3.6 Hz, 2H), 3.36 – 3.13 (m, 2H), 3.05 (dd, *J* = 9.0, 5.3 Hz, 1H), 2.96 (dd, *J* = 14.3, 8.9 Hz, 1H), 2.88 – 2.71 (m, 1H), 2.35 (t, *J* = 2.4 Hz, 1H), 1.20 (s, 3H), 1.07 (s, 3H)

^{13}C NMR (101 MHz, $CDCl_3$) δ 177.3, 176.2, 169.8, 142.3, 138.8, 136.4, 134.3, 132.5, 131.3, 130.8, 130.5, 129.7, 129.7, 129.0, 127.8, 127.3, 127.2, 127.1, 123.9, 122.6, 120.0, 119.2, 111.6, 77.5, 77.4, 77.2, 76.8, 74.4, 59.4, 58.3, 44.7, 42.3, 41.1, 39.0, 37.0, 31.1, 24.0, 21.2

^{19}F NMR (376 MHz, $CDCl_3$) δ 66.1

HRMS (ESI+) *m/z* [M + Na] calcd for $C_{34}H_{34}Cl_2FN_5O_5S$ Na^+ 736.1534, found 736.1536

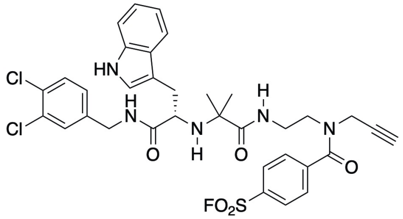

4g
(PJDU-220)
^{1}H NMR (400 MHz, $CDCl_3$ with TMS)

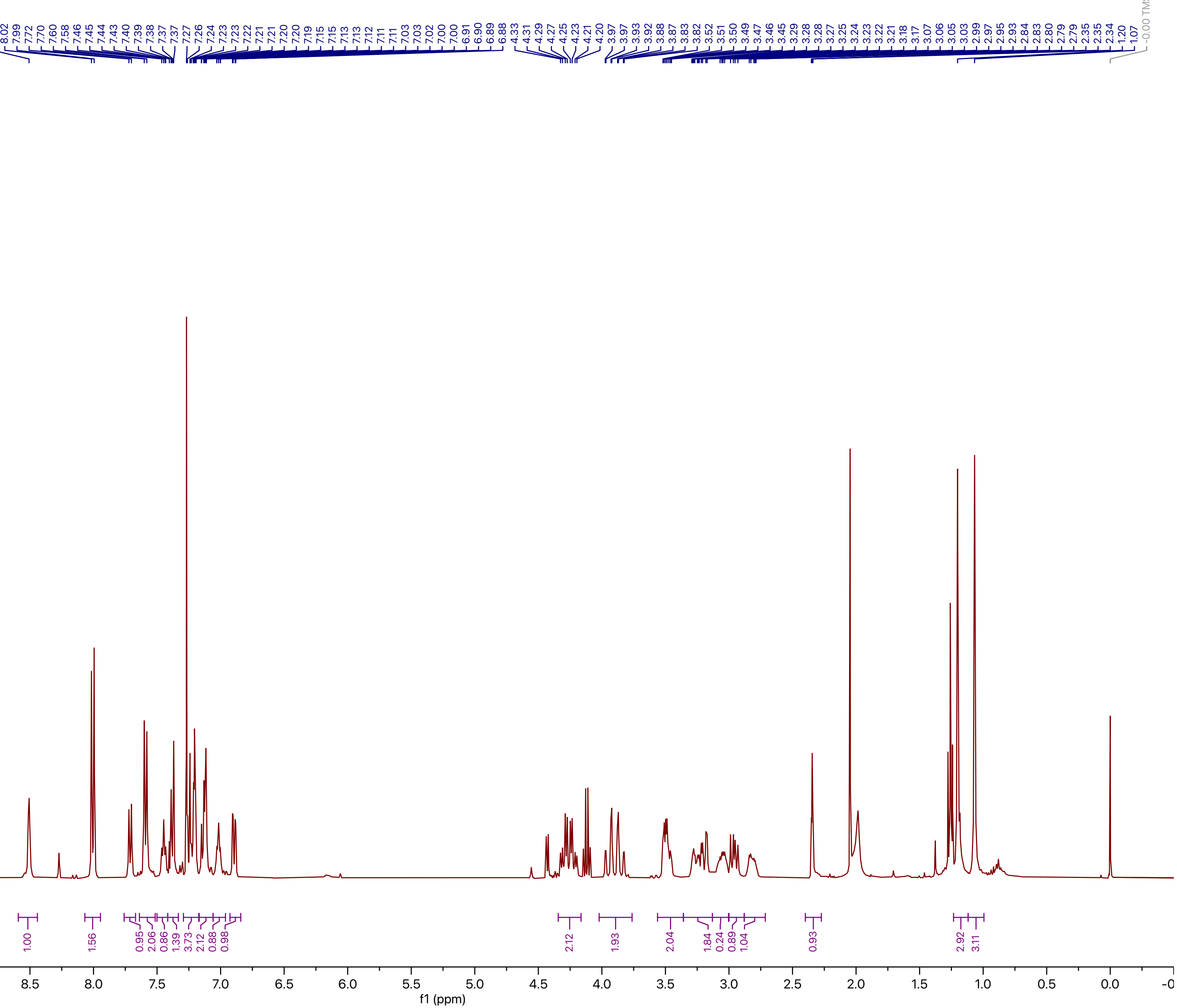

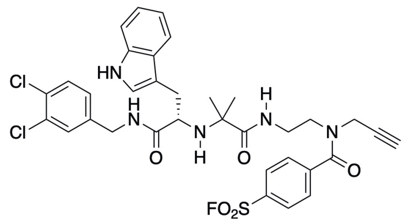

4g
(PJDU-220)
^{13}C NMR (101 MHz, $CDCl_3$ with TMS)

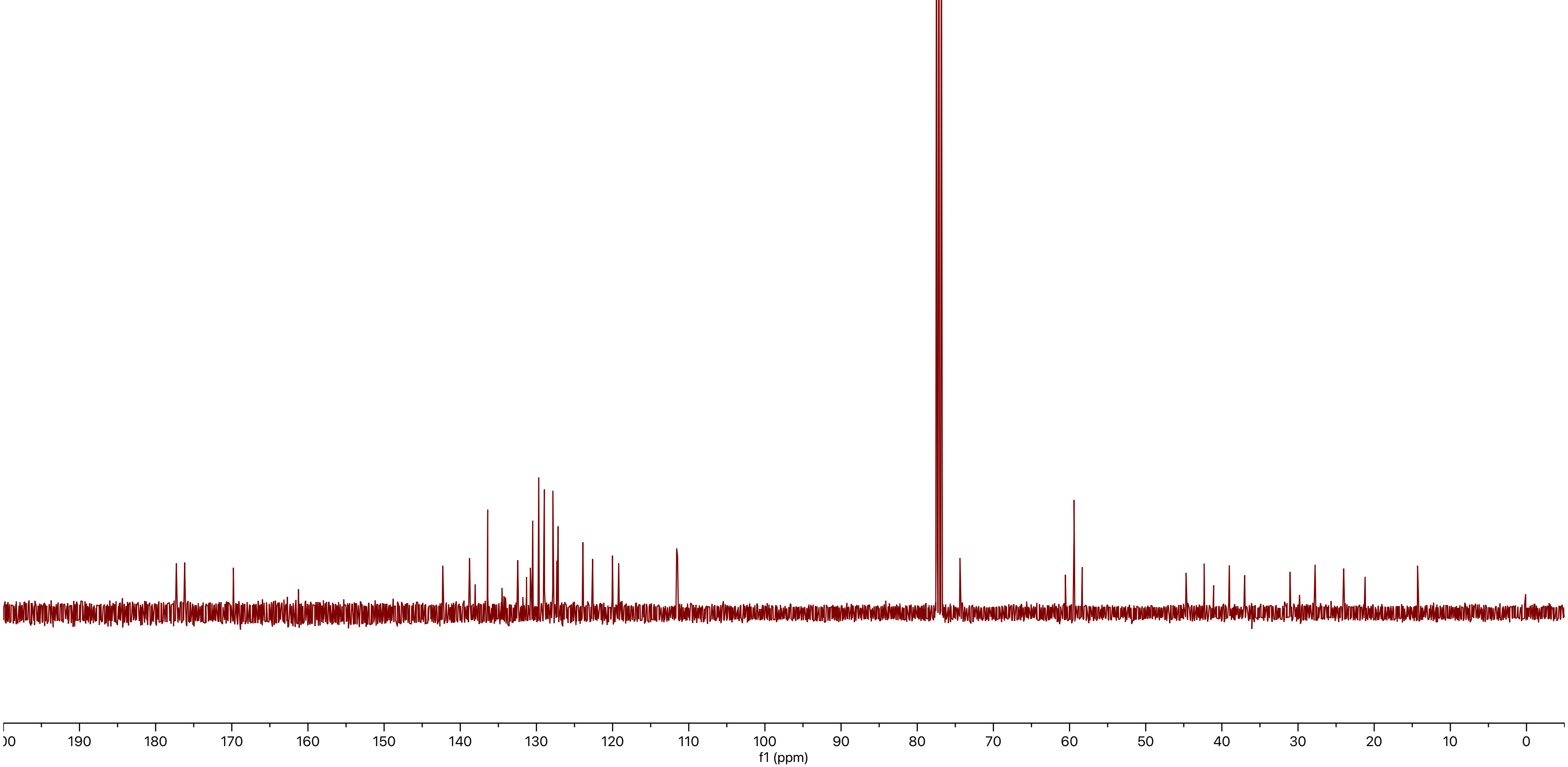

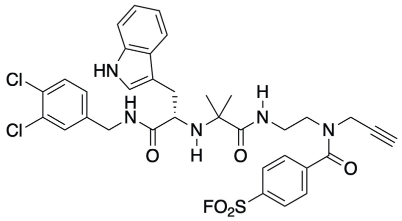

4g
(PJDU-220)
^{19}F NMR (376 MHz, $CDCl_3$ with TMS)

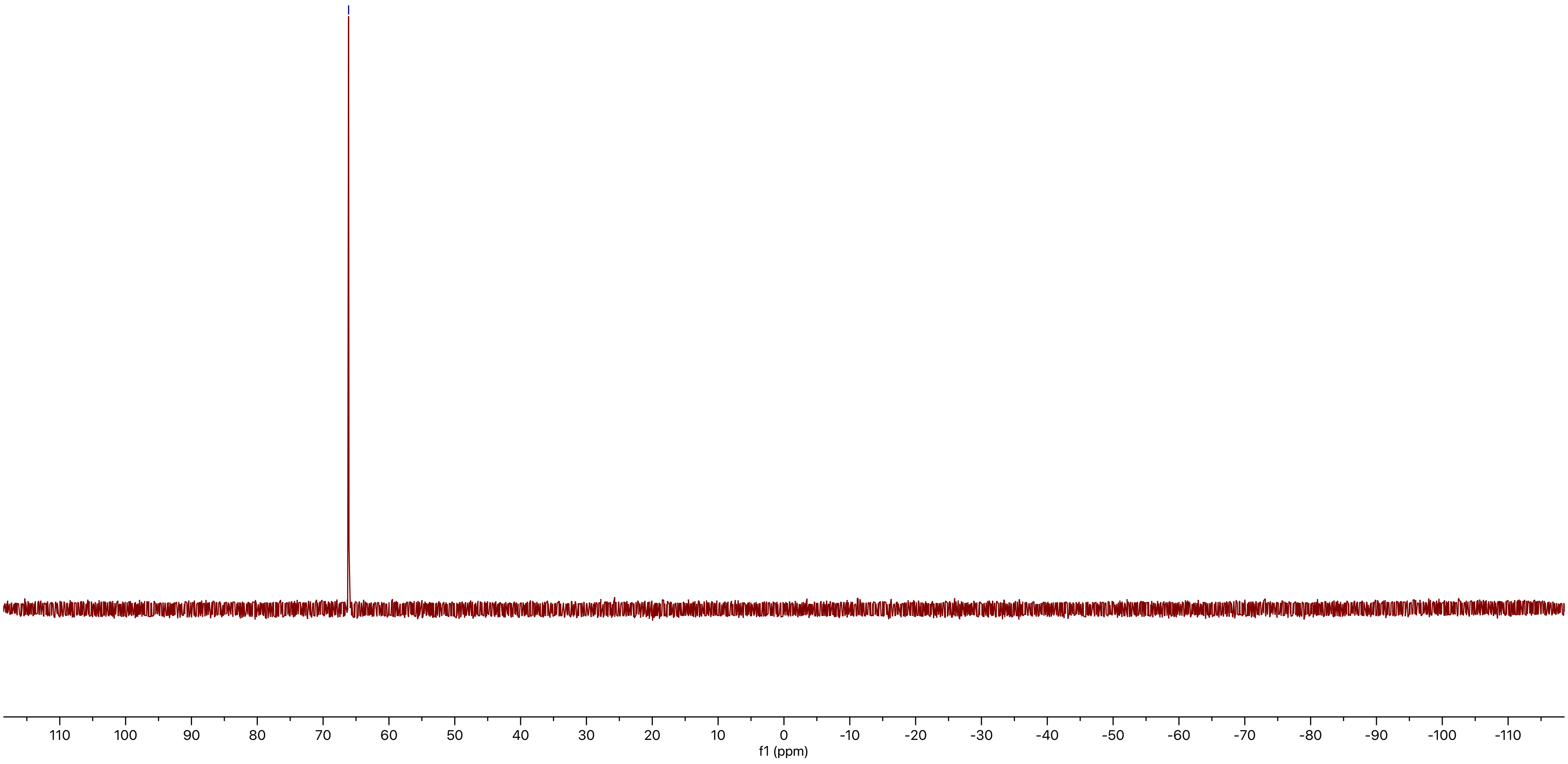

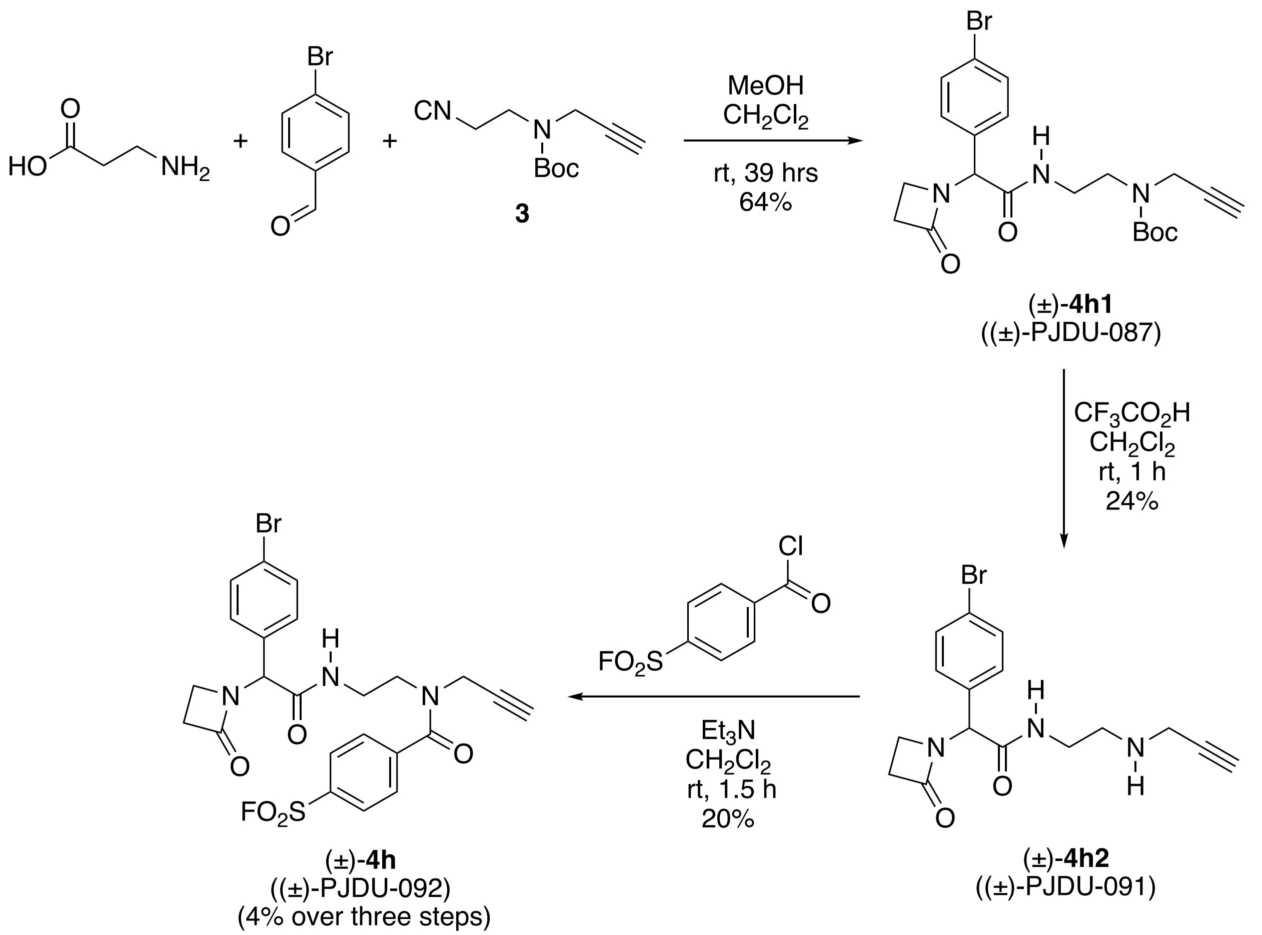

Scheme S7. *Synthesis of UBL-3CR-based FFSMP (±)-**4h** ((±)-PJDU-092).*

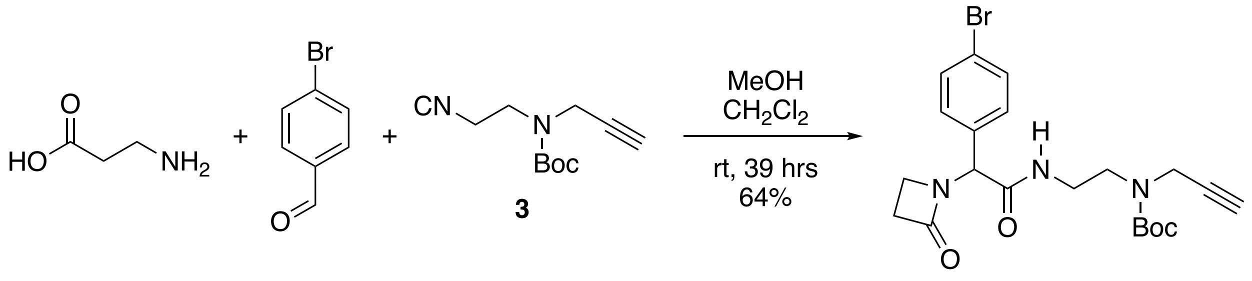

(±)-**4h1**
((±)-PJDU-087)

*(±)-tert-Butyl (2-(2-(4-bromophenyl)-2-(2-oxoazetidin-1-yl)acetamido)ethyl)(prop-2-yn-1-yl)carbamate ((±)-**4h1**) ((±)-PJDU-087).* A flame-dried, 10 mL round bottom flask equipped with a stir-bar, septum, and argon inlet needle was charged with β-alanine (35 mg, 0.39 mmol, 1.05 eq.) and 4-bromobenzaldehyde (72 mg, 0.39 mmol, 1.05 eq.). MeOH (0.8 mL) was added and this mixture was stirred for 30 minutes at room temperature. Next, isocyanide **3** (77 mg, 0.37 mmol, 1 eq.), was dissolved in CH_2Cl_2 (0.8 mL) and transferred to the reaction mixture via cannula. The reaction mixture was stirred for 39 hours at room temperature wherein TLC indicated a complete reaction. The reaction mixture was then concentrated under reduced pressure and purified by silica gel flash column chromatography (gradient elution with 25-75% EtOAc/hexanes, then 20% MeOH/$CHCl_3$) to give β-lactam (±)-**4h1** (109 mg, 64%; (±)-PJDU-087) as a pale-yellow oil.

R_f = 0.30 (75% EtOAc/hexanes)

1H NMR (400 MHz, $CDCl_3$, *peaks reported are for the major rotamer only) δ 7.50 (d, *J* = 8.4 Hz, 3H), 7.22 (d, *J* = 8.0 Hz, 2H), 5.39 (s, 1H), 3.98 (br s, 2H), 3.86 – 3.21 (m, 6H), 3.21 – 2.93 (m, 2H), 2.26 (t, *J* = 2.5 Hz, 1H), 1.43 (s, 9H)

^{13}C NMR (101 MHz, $CDCl_3$, *peaks reported are for the major rotamer only) δ 171.2, 168.5, 167.8, 133.7, 132.2, 130.1, 122.8, 81.1, 77.3, 72.0, 58.7, 45.9, 39.6, 38.7, 37.5, 36.4, 28.3

HRMS (ESI+) *m/z* [M + H] calcd for $C_{21}H_{26}BrN_3O_4$ H^+ 463.1185, found 463.1192

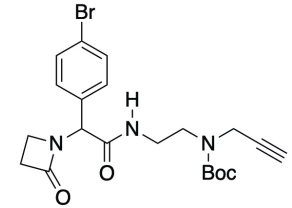

(±)-**4h1**
((±)-PJDU-087)
^{1}H NMR (400 MHz, $CDCl_3$ with TMS)

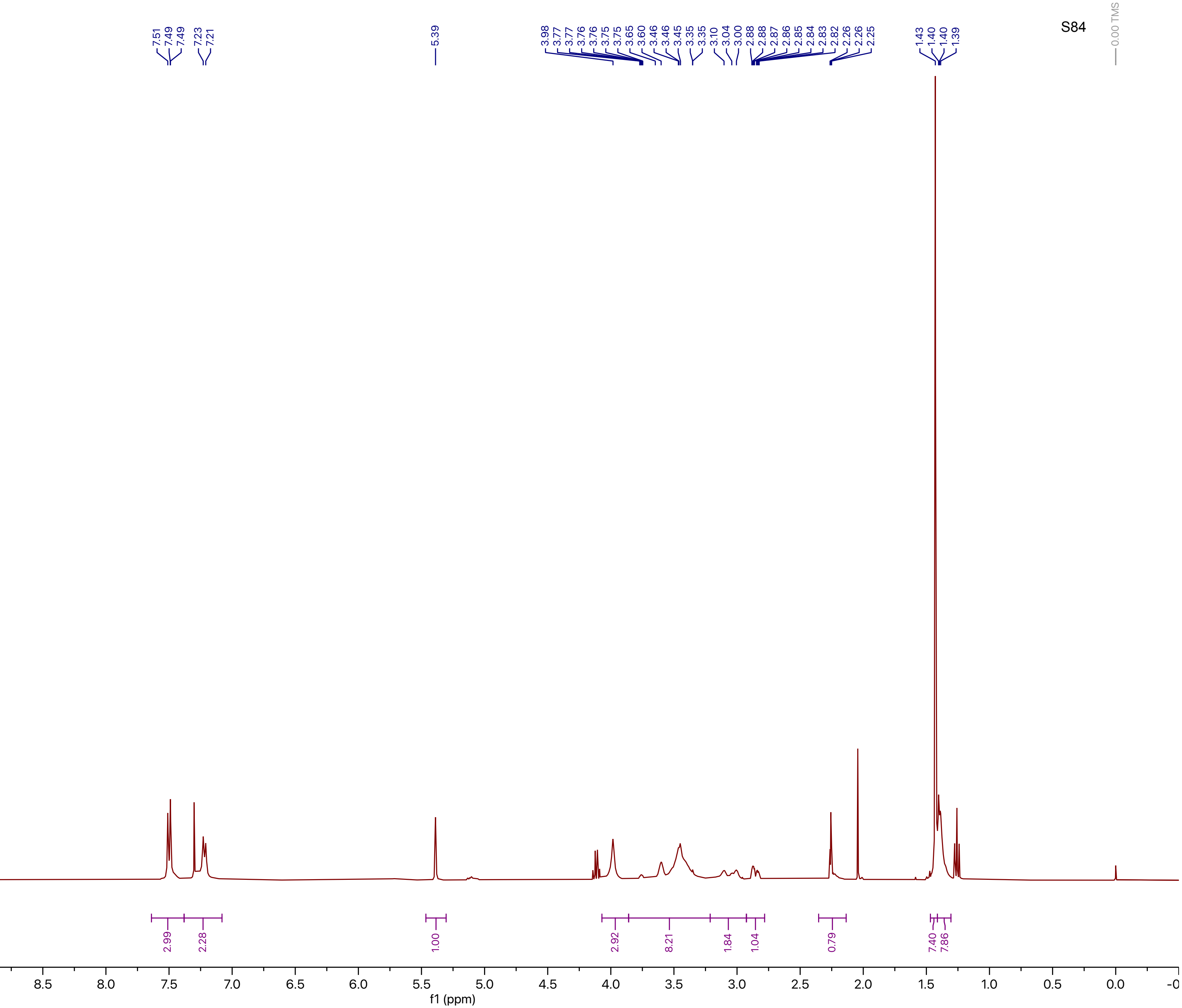

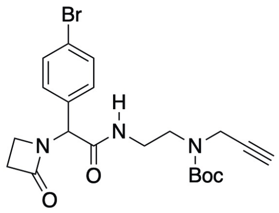

(±)-**4h1**
((±)-PJDU-087)
^{13}C NMR (101 MHz, $CDCl_3$ with TMS)

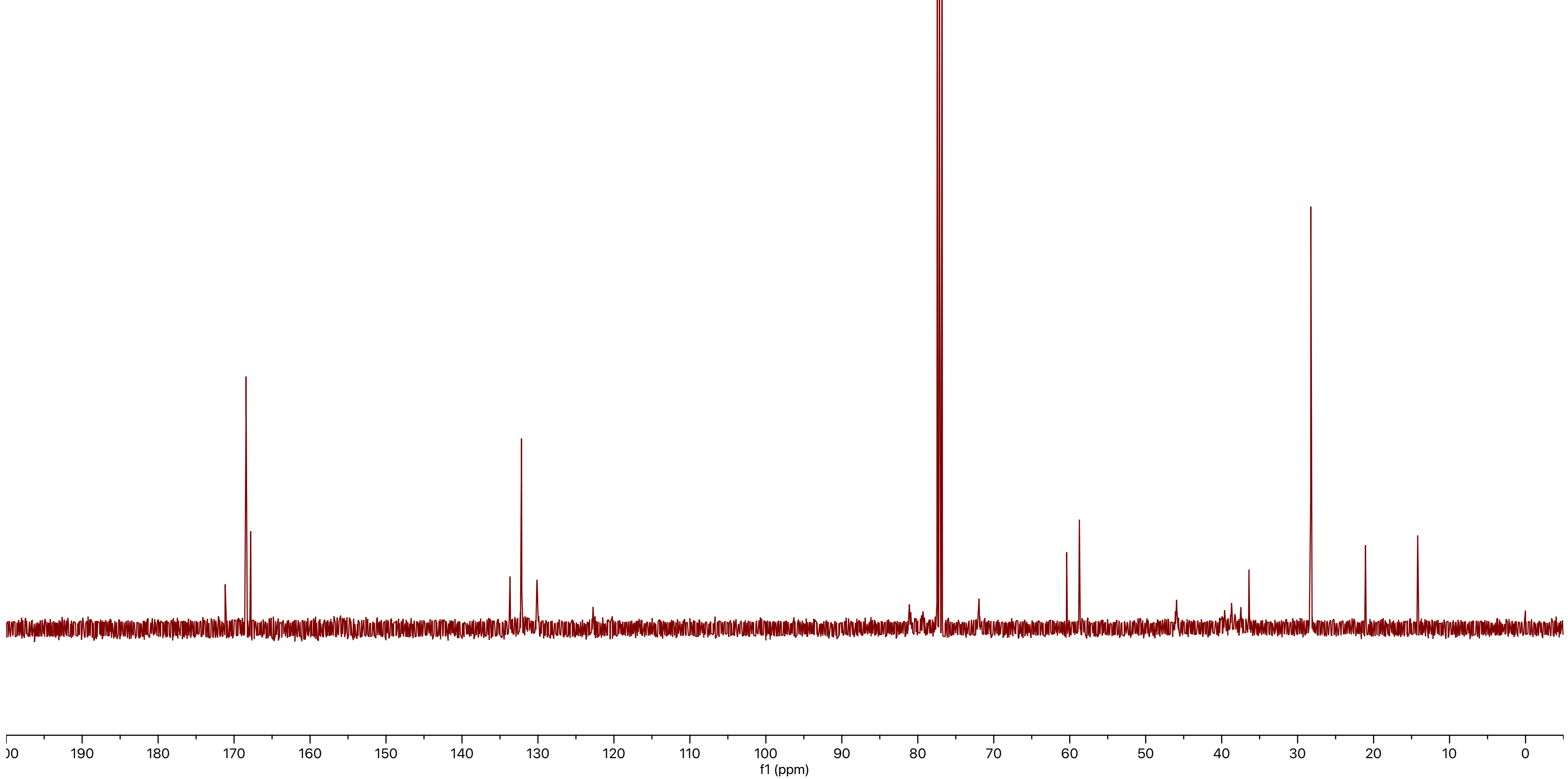

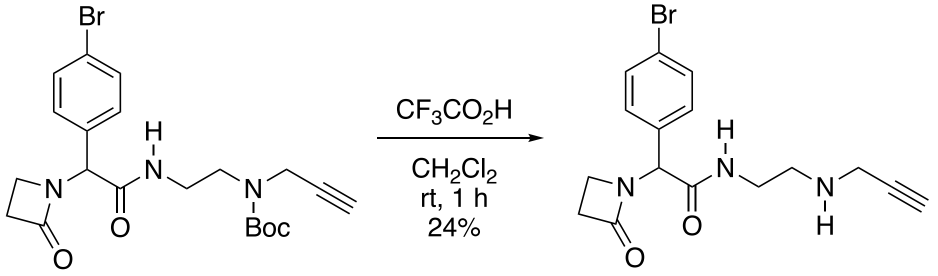

(±)-**4h1**
((±)-PJDU-087)

(±)-**4h2**
((±)-PJDU-091)

*(±)-2-(4-Bromophenyl)-2-(2-oxoazetidin-1-yl)-N-(2-(prop-2-yn-1-ylamino)ethyl)acetamide ((±)-**4h2**) ((±)-PJDU-091).* A flame-dried, round-bottom flask equipped with a stirbar, septum, and argon inlet needle was charged with Boc carbamate substrate (±)-**4h1** (107 mg, 0.23 mmol, 1 eq.; (±)-PJDU-087) and CH_2Cl_2 (2.3 mL). Trifluoroacetic acid (0.18 mL, 2.3 mmol, 10 eq.) was then added dropwise over 2 minutes. After the addition, the reaction was stirred at room temperature for 1 hour, wherein TLC indicated complete consumption of the starting Boc carbamate substrate. The reaction was then quenched by dropwise addition of sat. aq. $NaHCO_3$ (vigorous gas evolution) to the reaction mixture until gas evolution ceased (~10 mL), then 1M aq. NaOH (~4 mL) was added to raise the pH to 8-9 (checked with pH paper). This biphasic mixture was then transferred to a separatory funnel and diluted with CH_2Cl_2 (15 mL), the layers were separated, and the aqueous layer was extracted with CH_2Cl_2 (2 x 15 mL). The organic layers were combined, washed with brine (10 mL), dried ($MgSO_4$), gravity filtered, and concentrated under reduced pressure. The crude material was then purified by silica gel chromatography (elution with 5% MeOH/$CHCl_3$) to give propargyl amine (±)-**4h2** (20 mg, 24%, (±)-PJDU-091) as a light yellow sticky solid

R_f = 0.30 (10% MeOH/$CHCl_3$)

1H NMR (500 MHz, $CDCl_3$, *peaks reported are for the major rotamer only) δ 7.51 (d, *J* = 8.5 Hz, 2H), 7.26 (d, *J* = 8.3 Hz, 2H), 6.74 (t, *J* = 5.5 Hz, 1H), 5.35 (s, 1H), 3.60 (td, *J* = 5.6, 2.7 Hz, 1H), 3.45 – 3.30 (m, 4H), 3.16 (td, *J* = 5.7, 2.7 Hz, 1H), 3.01 (ddd, *J* = 14.8, 5.6, 2.8 Hz, 1H), 2.88 (ddd, *J* = 14.8, 5.7, 2.7 Hz, 1H), 2.83 (t, *J* = 5.8 Hz, 2H), 2.22 (t, *J* = 2.4 Hz, 1H), 1.71 (br s, 2H)

^{13}C NMR (126 MHz, $CDCl_3$, *peaks reported are for the major rotamer only) δ 168.8, 168.5, 134.2, 132.8, 130.5, 123.4, 82.2, 72.3, 59.7, 47.5, 39.53, 39.51, 38.2, 36.9

HRMS (ESI+) *m/z* [M + H] calcd for $C_{16}H_{18}BrN_3O_2$ H^+ 364.0655, found 364.0655

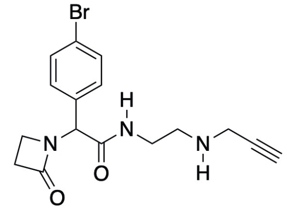

(±)-**4h2**
((±)-PJDU-091)
^{1}H NMR (500 MHz, $CDCl_3$ with TMS)

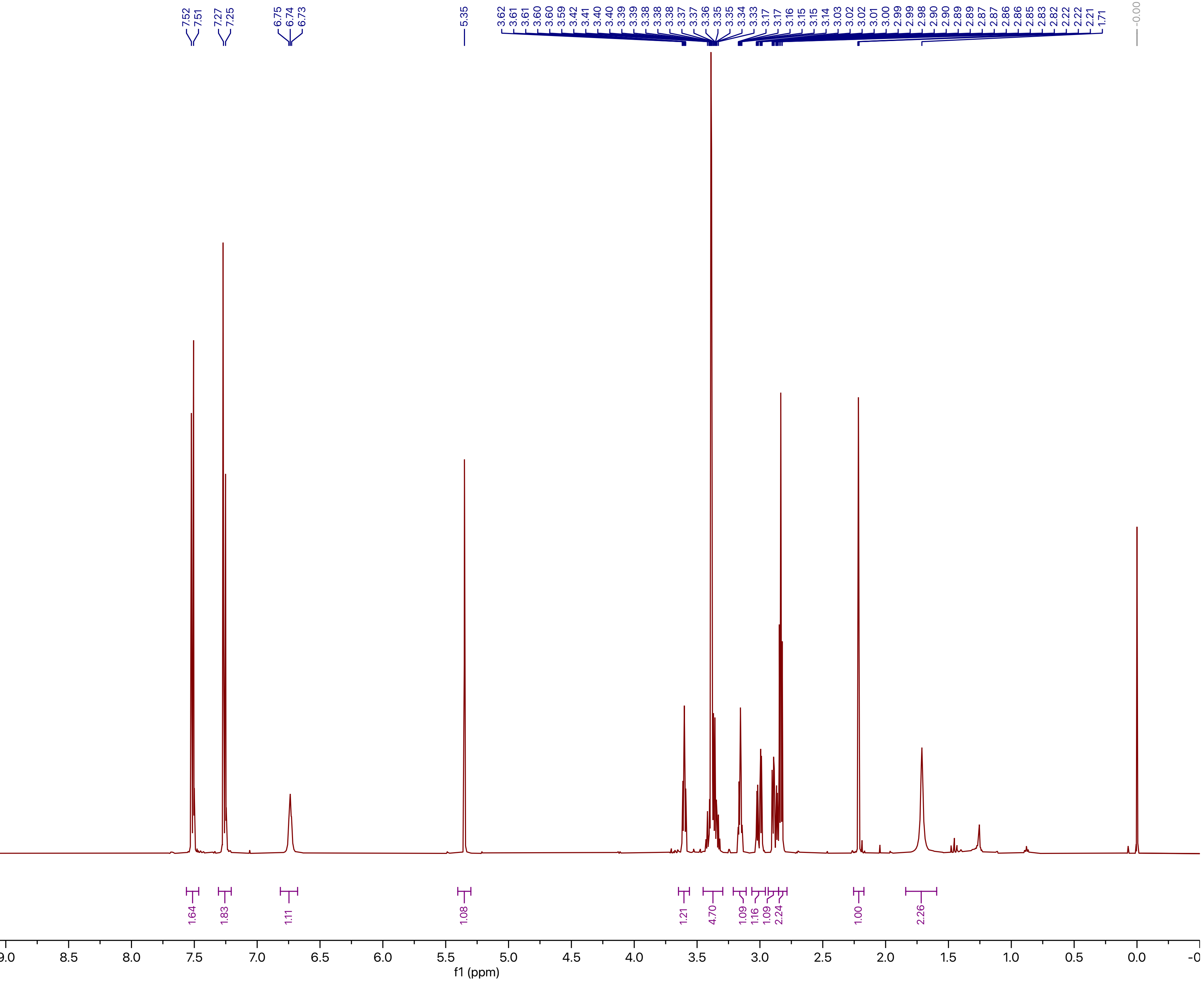

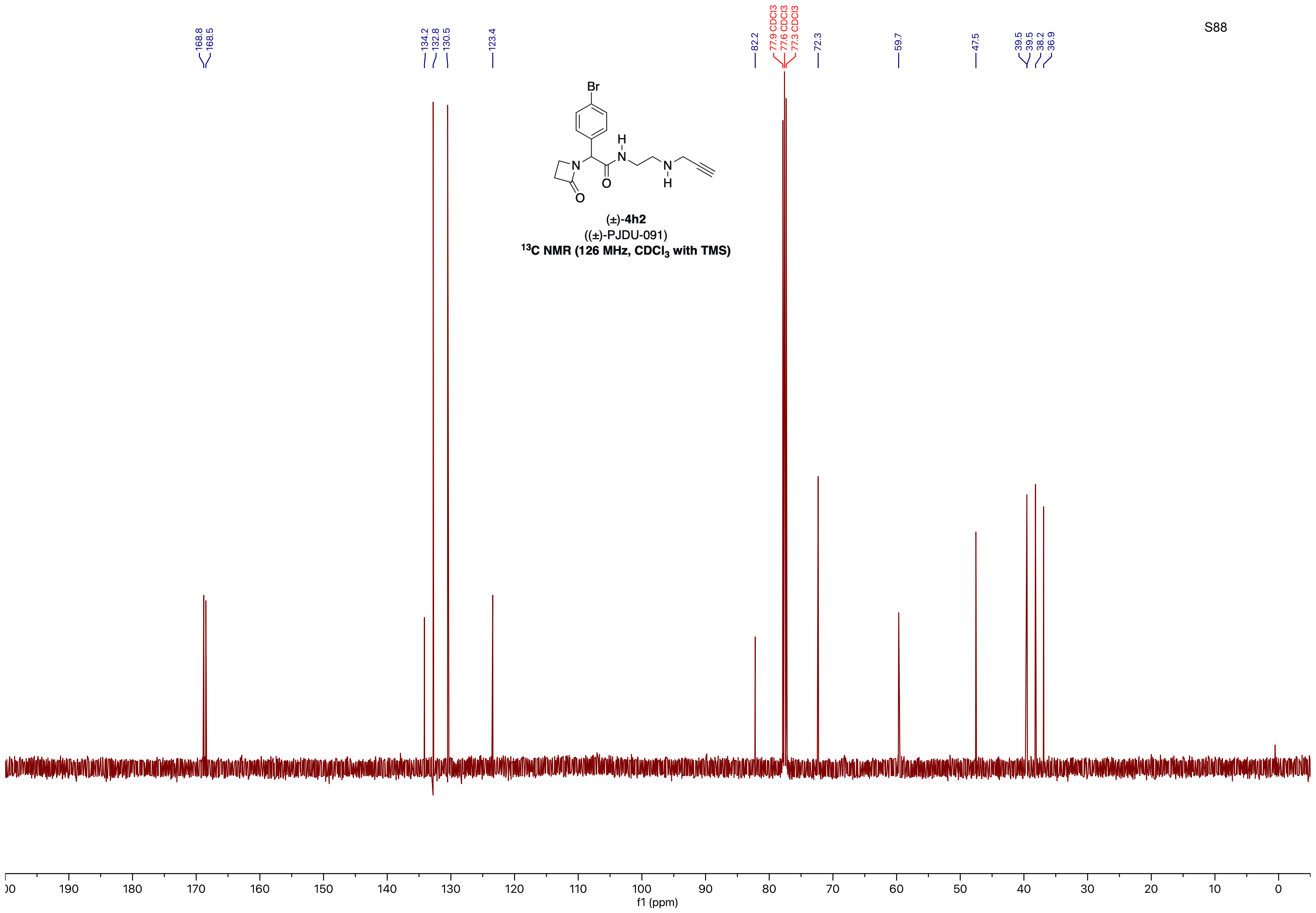

(±)-**4h2**
((±)-PJDU-091)
^{13}C NMR (126 MHz, $CDCl_3$ with TMS)

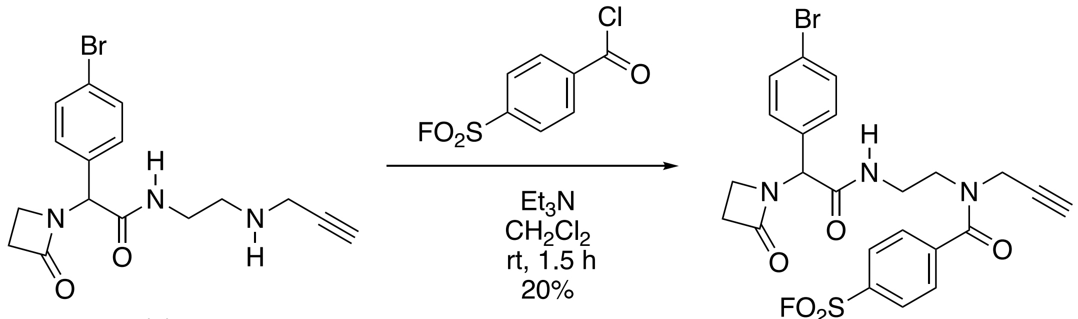

(±)-**4h2**
((±)-PJDU-091)

(±)-**4h**
((±)-PJDU-092)

*(±)-4-((2-(2-(4-Bromophenyl)-2-(2-oxoazetidin-1-yl)acetamido)ethyl)(prop-2-yn-1-yl)carbamoyl)benzenesulfonyl fluoride ((±)-**4h**) ((±)-PJDU-092).* A flame-dried round-bottom flask equipped with a stir-bar, septum, and argon inlet needle was charged with propargyl amine (±)-**4h2** (20 mg, 0.06 mmol, 1 eq.; (±)-PJDU-091) and CH_2Cl_2 (1.5 mL), then cooled to 0°C with an ice-water bath. Triethylamine (0.01 mL, 0.07 mmol, 1.3 eq.) was added dropwise over 1 min, then a solution of 4-(fluorosulfonyl)benzoyl chloride (16 mg, 0.072 mmol, 1.3 equiv) in CH_2Cl_2 (0.5 mL) was added dropwise over 1 minute. The reaction mixture was stirred for 5-10 min at 0°C, then the ice bath was removed and the reaction mixture allowed to warm to room temperature and stirred for 1.5 hours, wherein TLC analysis indicated a complete reaction. Next, the reaction mixture was transferred to a separatory funnel and washed with deionized H_2O (10 mL). The aq. layer was extracted with CH_2Cl_2 (3 x 10 mL). The combined organic layers were then washed with sat. aq. $NaHCO_3$ (10 mL), dried ($MgSO_4$), filtered, and concentrated under reduced pressure. The crude residue was then purified by silica gel flash column chromatography (gradient elution with 5-10% MeOH/$CHCl_3$) to give FFSMP (±)-**4h** (6 mg, 20%; (±)-PJDU-092) as a pale yellow oil.

R_f = 0.18 (10% MeOH/$CHCl_3$)

1H NMR (500 MHz, $CDCl_3$, *peaks reported are for the major rotamer only) δ 8.09 (d, J = 8.1 Hz, 2H), 7.70 (d, J = 8.1 Hz, 2H), 7.51 (d, J = 8.3 Hz, 2H), 7.26 – 7.17 (m, 2H), 5.29 (s, 1H), 4.04 – 3.86 (m, 2H), 3.79 (dt, J = 14.4, 5.3 Hz, 1H), 3.74 – 3.42 (m, 4H), 3.44 – 3.33 (m, 1H), 3.13 – 2.96 (m, 1H), 2.96 – 2.79 (m, 2H), 2.43 (t, J = 2.4 Hz, 1H)

^{13}C NMR (126 MHz, $CDCl_3$, *peaks reported are for the major rotamer only) δ 170.5, 169.0, 168.1, 132.3, 132.3, 130.2, 130.0, 128.9, 128.2, 123.1, 74.4, 60.0, 44.6, 39.0, 38.6, 36.4, 29.7

^{19}F NMR (376 MHz, $CDCl_3$) δ 66.06

HRMS (ESI+) m/z [M + Na] calcd for $C_{23}H_{21}BrFN_3O_5$ Na^+ 572.0262, found 572.0258

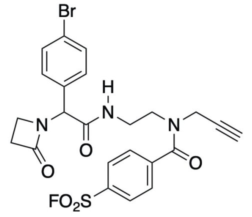

(±)-**4h**
((±)-PJDU-092)
^{1}H NMR (500 MHz, $CDCl_3$ with TMS)

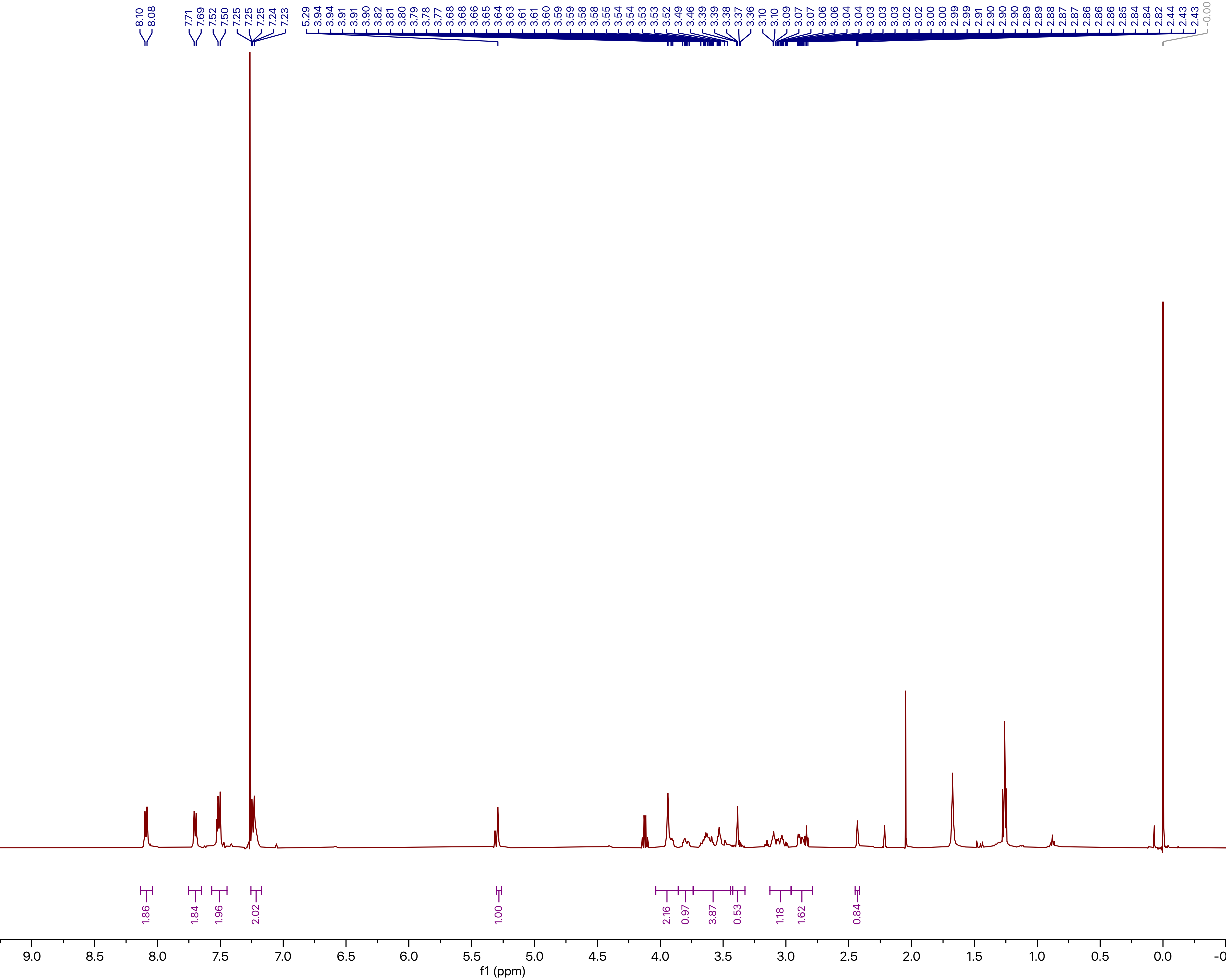

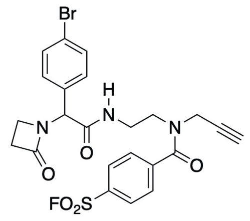

(±)-**4h**
((±)-PJDU-092)
^{13}C NMR (126 MHz, $CDCl_3$ with TMS)

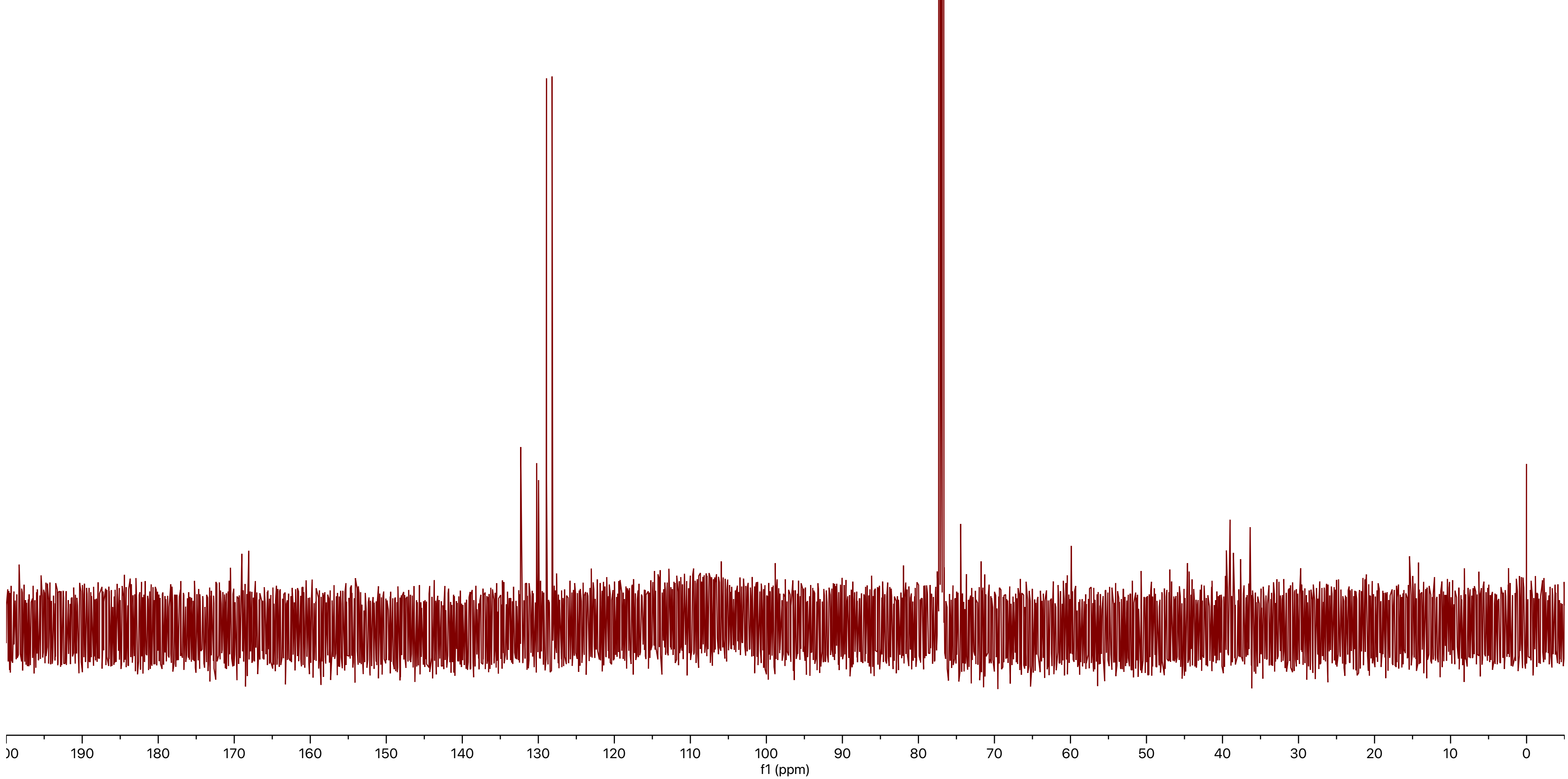

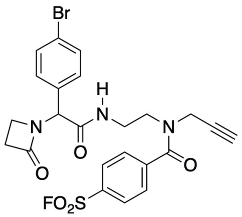

(±)-**4h**
((±)-PJDU-092)
^{19}F NMR (376 MHz, $CDCl_3$ with TMS)

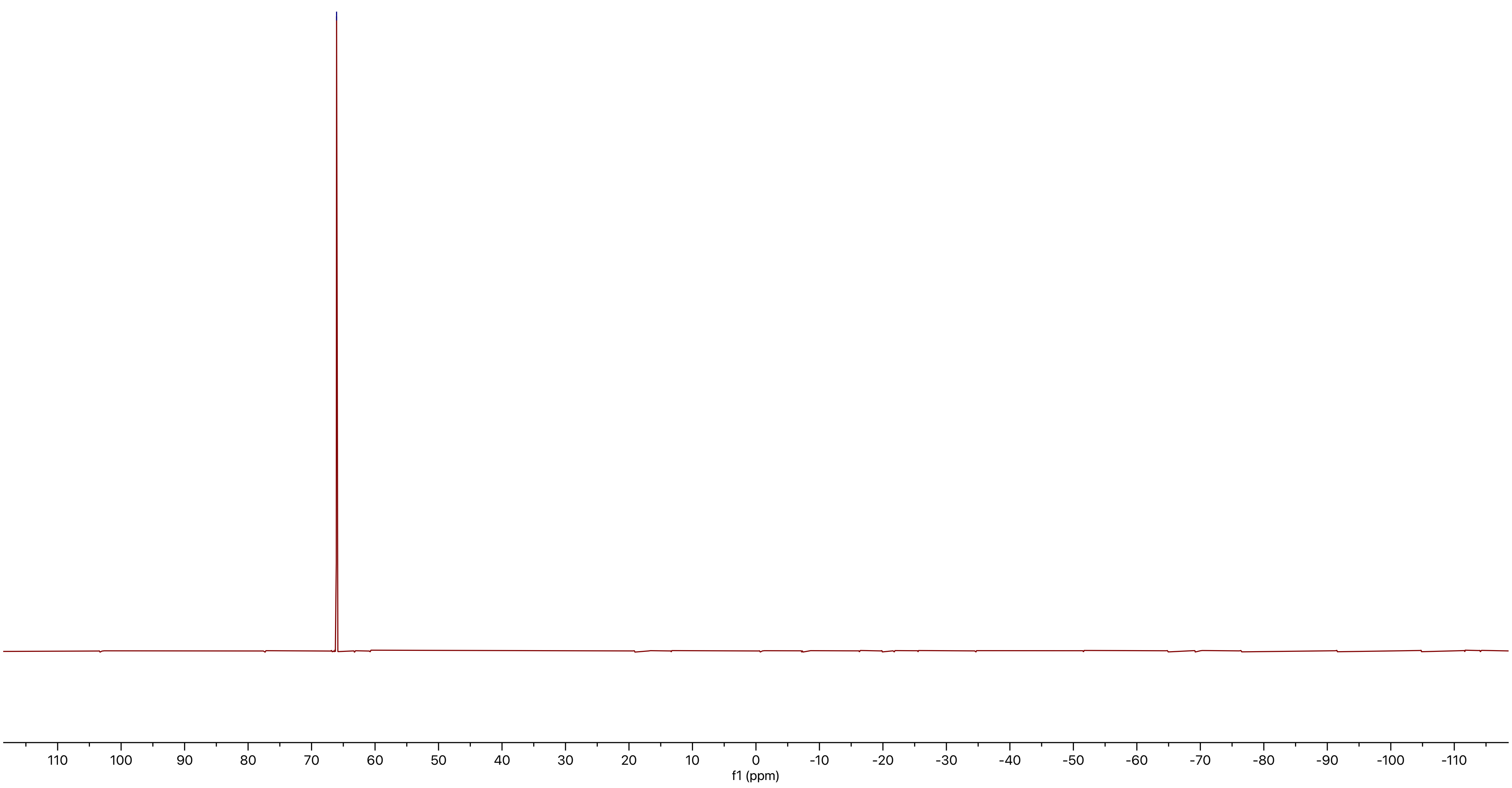

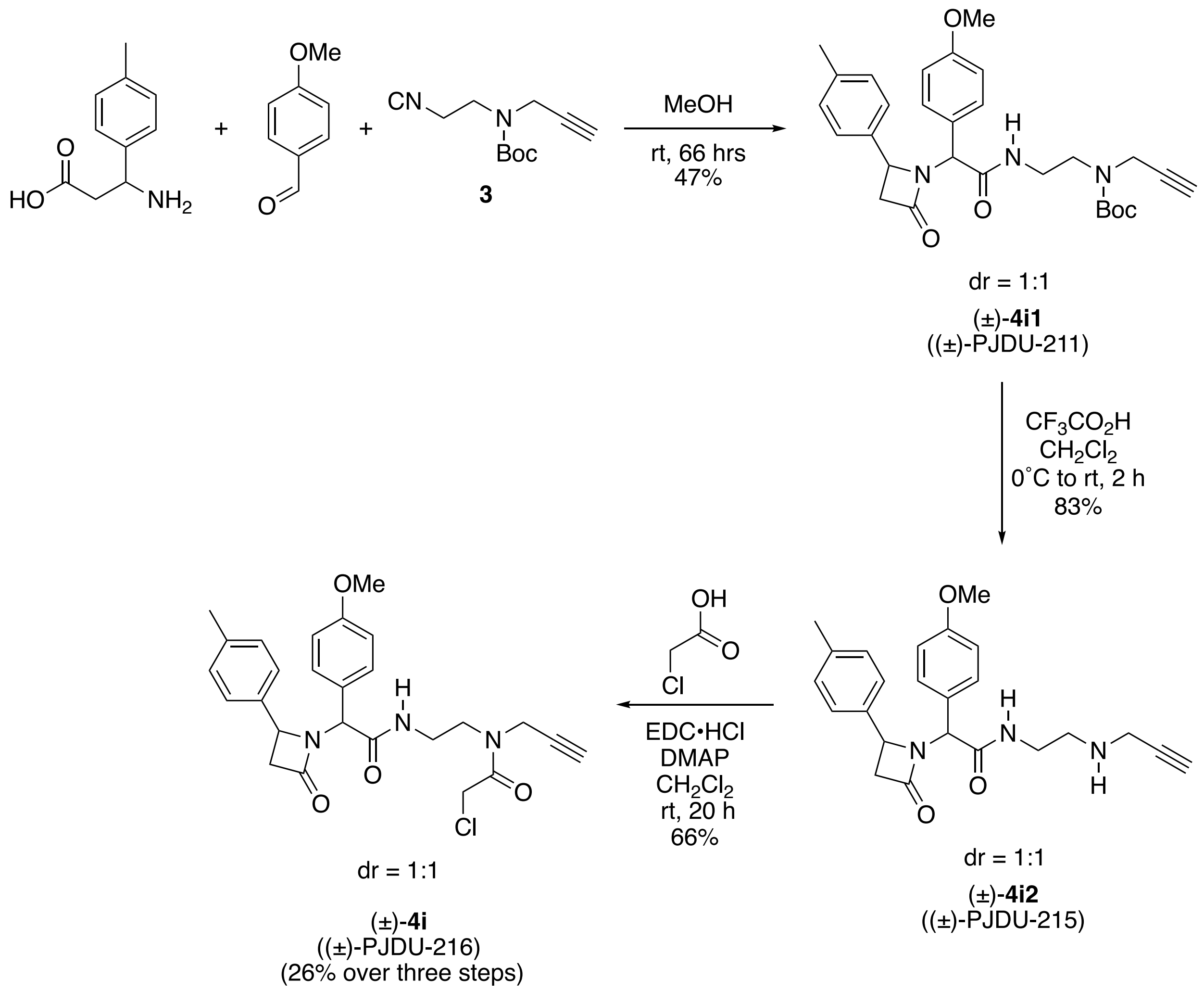

Scheme S8. *Synthesis of UBL-3CR-based FFSMP (±)-**4i** ((±)-PJDU-216).*

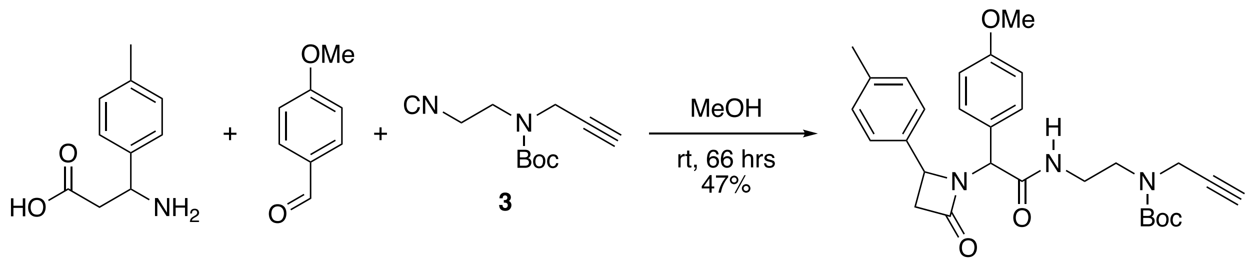

dr = 1:1

(±)-**4i1**

((±)-PJDU-211)

*(±)-tert-Butyl (2-(2-(4-methoxyphenyl)-2-(2-oxo-4-(p-tolyl)azetidin-1-yl)acetamido)ethyl)(prop-2-yn-1-yl)carbamate ((±)-**4i1**) ((±)-PJDU-211).* A flame-dried, 2 dram vial equipped with a stir-bar, septum, and argon inlet needle was charged with (±)-3-amino-3-(*p*-tolyl)propanoic acid (199 mg, 1.11 mmol, 1.1 eq.) and 4-methoxybenzaldehyde (0.14 mL, 1.11 mmol, 1.1 eq.). MeOH (1.5 mL) was added and this mixture was stirred for 30 minutes at room temperature. Next, isocyanide **3** (211 mg, 1.01 mmol, 1 eq.), was dissolved in MeOH (1.4 mL) and transferred to the reaction mixture via cannula. The reaction mixture was stirred for 66 hours at room temperature wherein TLC indicated a complete reaction. The reaction mixture was then concentrated under reduced pressure, dissolved in EtOAc, and then washed sequentially with saturated $NaHCO_3$ solution, H_2O, and brine. The EtOAc layer was then dried ($MgSO_4$), filtered, concentrated and purified by silica gel flash column chromatography (gradient elution with 40-60% EtOAc/hexanes, to give β-lactam (±)-**4i1** (240 mg, 47%; (±)-PJDU-211) as a ~1:1 mixture of diastereomers based on integration of the methoxy group via 1H NMR.

R_f = 0.16 (50% EtOAc/hexanes)

1H NMR (500 MHz, $CDCl_3$, **peaks reported are for the major rotamer of the major diastereomer, *other diastereomer where distinguishable) δ 7.31-7.01 (m, 7H), 6.83-6.81 (m, 1H), 6.66 (bs, 1H), 4.99-4.89 (m, 1H), 4.78-4.76 (m, 1H)*, 4.52-4.51 (m, 1H), 3.92 (m, 2H), 3.78 (s, 3H), 3.71 (s, 3H)*, 3.44-3.30 (m, 5H), 2.89-2.79 (m, 1H), 2.33 (s, 3H), 2.28 (s, 3H)*, 2.21 (app s, 1H), 1.33 (s, 9H), 1.42 (s, 9H)*

^{13}C NMR (126 MHz, $CDCl_3$, **peaks reported are for the major rotamer of the major diastereomer) δ 168.9, 168.2, 159.5, 155.4, 138.2, 135.1, 130.2, 129.4, 126.8, 126.4, 113.9, 80.5, 79.5, 71.6, 62.3, 61.2, 55.1, 54.6, 45.8, 39.0, 37.2, 28.2, 21.0

HRMS (ESI+) *m/z* [M + Na] calcd for $C_{29}H_{35}N_3O_5Na$ 528.2469, found 528.2473

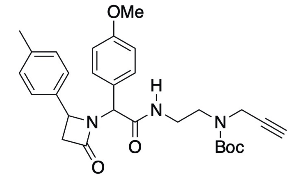

dr = 1:1

(±)-**4i1**
((±)-PJDU-211)
^{1}H NMR (500 MHz, CDCl$_3$ with TMS)

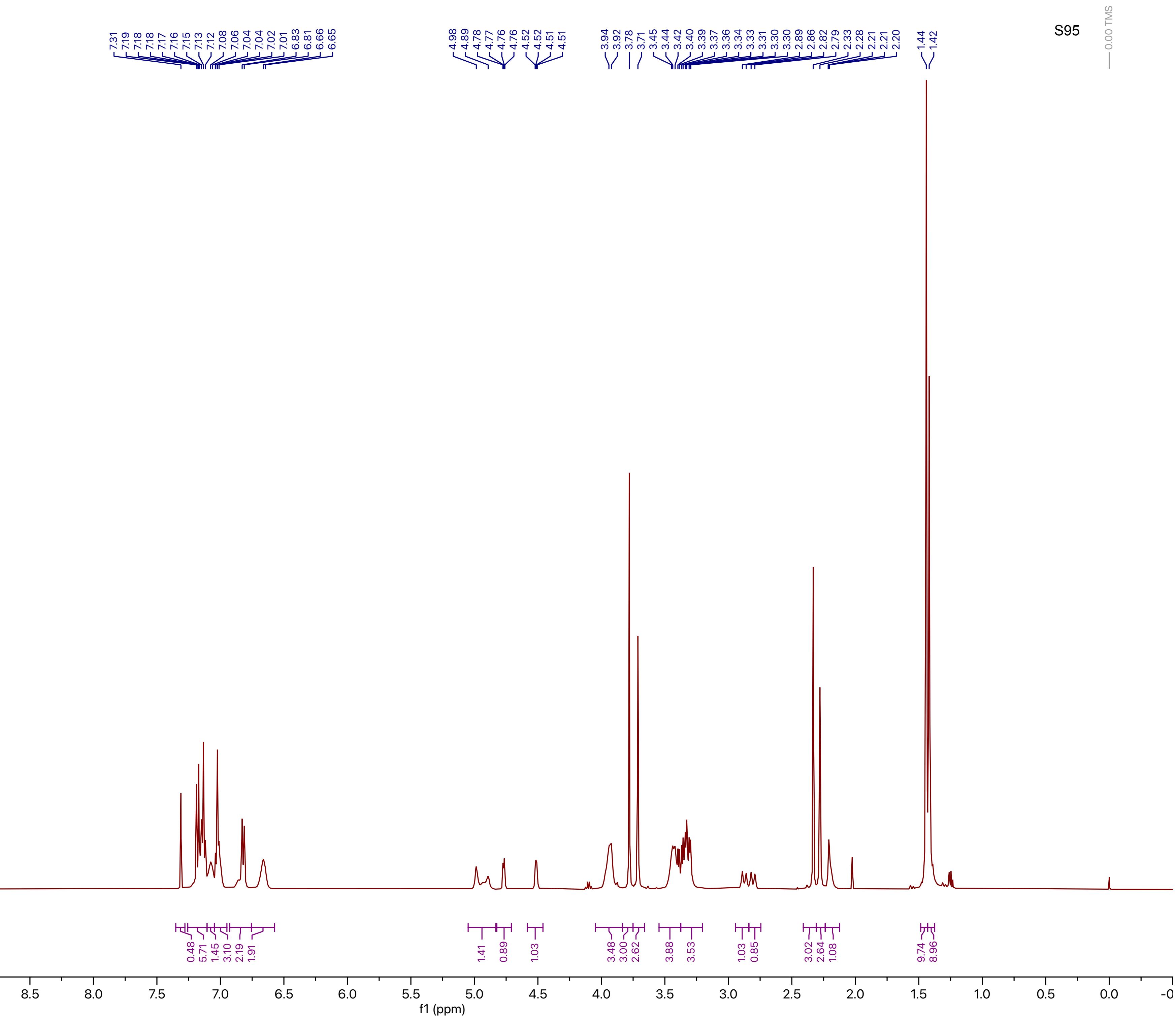

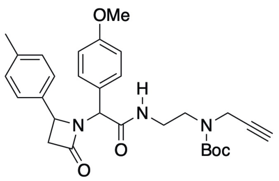

dr = 1:1

(±)-**4i1**
((±)-PJDU-211)
^{13}C NMR (126 MHz, $CDCl_3$ with TMS)

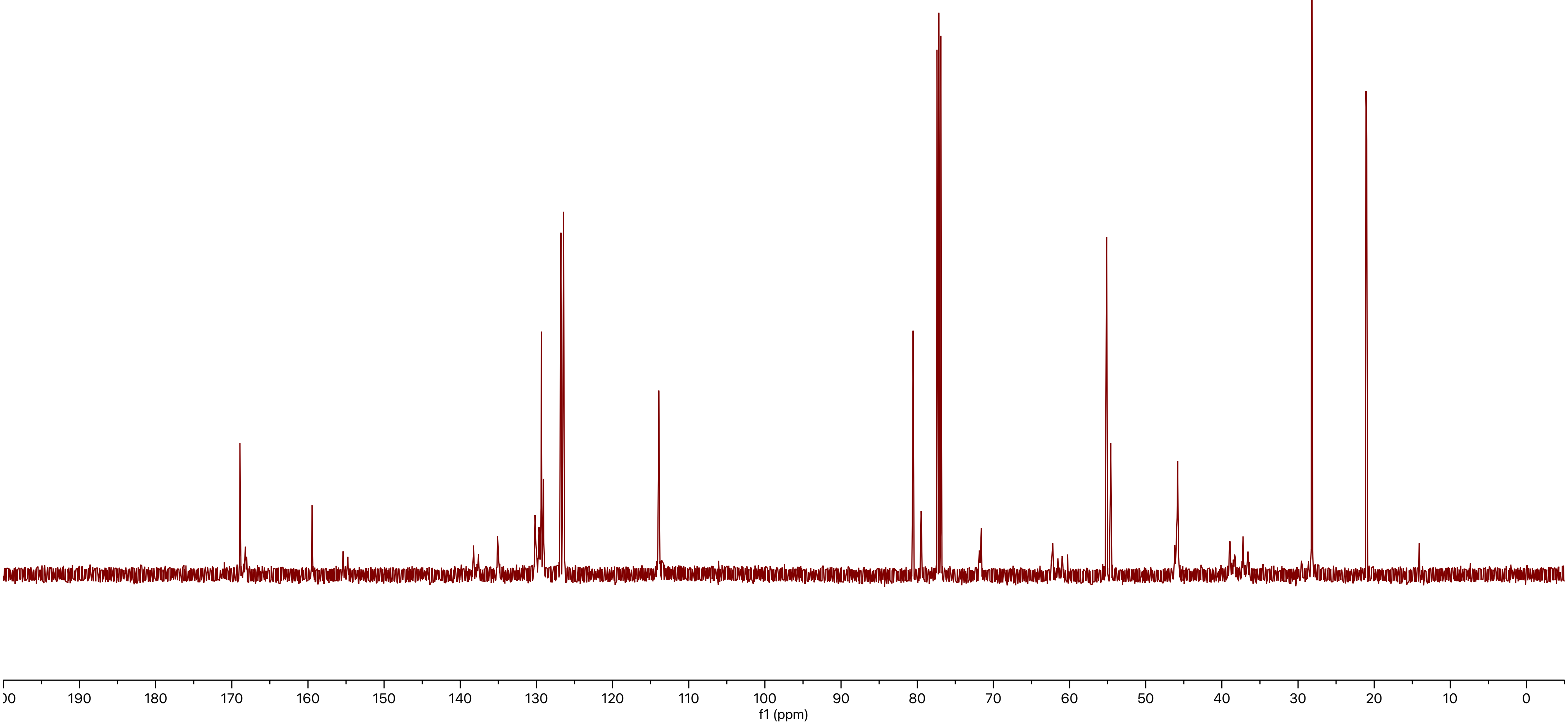

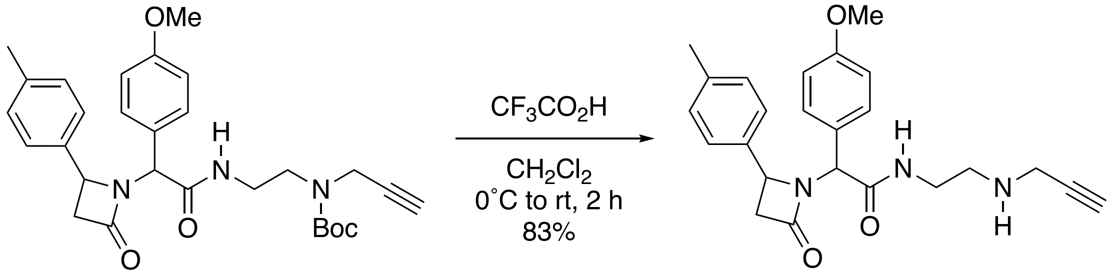

dr = 1:1 | dr = 1:1

(±)-**4i1** | (±)-**4i2**

((±)-PJDU-211) | ((±)-PJDU-215)

*(±)-2-(4-Methoxyphenyl)-2-(2-oxo-4-(p-tolyl)azetidin-1-yl)-N-(2-(prop-2-yn-1-ylamino)ethyl)acetamide ((±)-**4i2**) ((±)-PJDU-215).* A 25 mL flame-dried, round-bottom flask equipped with a stirbar, septum, and argon inlet needle was charged with Boc carbamate substrate (±)-**4i1** (240 mg, 0.48 mmol, 1 eq.; (±)-PJDU-211) and CH_2Cl_2 (4.8 mL) and cooled to 0˚C. Trifluoroacetic acid (0.44 mL, 5.7 mmol, 12 eq.) was then added dropwise over 2 minutes. After the addition, the ice bath was removed and the reaction was stirred at room temperature for 2 hours, wherein TLC indicated complete consumption of the starting Boc carbamate substrate. The reaction was then quenched by dropwise addition of sat. aq. $NaHCO_3$ (vigorous gas evolution) to the reaction mixture until gas evolution ceased (~10 mL), then 1M aq. NaOH (~5 mL) was added to raise the pH to 8-9 (checked with pH paper). This biphasic mixture was then transferred to a separatory funnel and diluted with CH_2Cl_2 (20 mL), the layers were separated, and the aqueous layer was extracted with CH_2Cl_2 (2 x 20 mL). The organic layers were combined, washed with brine (10 mL), dried ($MgSO_4$), gravity filtered, and concentrated under reduced pressure to give propargyl amine (±)-**4i2** (161 mg, 83%, (±)-PJDU-215) as a light yellow sticky solid, which proved sufficiently pure to take on to the N-acylation arming step with chloroacetic acid. 1H NMR of crude propargyl amine (±)-**4i2** indicated a ~1:1 mixture of diastereomers based on integration of the *p*-tolyl methyl and methoxy groups.

R_f = 0.27 (5% MeOH/CH_2Cl_2)

1H NMR (500 MHz, $CDCl_3$, **peaks reported for the major rotamer/diastereomer only, *other diastereomer where distinguishable δ 7.17 (m, 1H), 7.14-7.12 (m, 3H), 7.08-7.04 (m, 2H), 6.84-6.82 (m, 1H), 6.72-6.70 (m, 1H), 6.68 (t, *J* = 5.5 Hz, 1H), 4.96 (s, 1H), 4.89 (s, 1H)*, 4.72 (dd, *J* = 2.5 Hz, 1H)*, 4.47 (dd, *J* = 3.0 Hz, 1H), 3.80 (s, 3H), 3.74 (s, 3H)*, 3.39-3.26 (m, 5H), 2.93-2.90 (m, 1H), 2.86-2.82 (m, 1H), 2.72 (t, *J* = 6.0 Hz, 1H), 2.34 (s, 3H), 2.30 (s, 3H)*, 2.21 (t, *J* = 2.5 Hz, 1H), 2.20 (t, *J* = 2.5 Hz, 1H)*, 1.87 (bs, 1H)

^{13}C NMR (126 MHz, $CDCl_3$, **peaks reported for the major rotamer/diastereomer only) δ 168.9, 168.5, 159.6, 138.5, 135.1, 130.2, 129.5, 127.0, 126.6, 114.1, 81.9, 71.7, 62.6, 55.3, 54.8, 47.1, 45.8, 39.2, 37.7, 21.2

HRMS (ESI+) *m/z* [M + Na] calcd for $C_{24}H_{27}N_3O_3Na$ 428.1945, found 428.1946

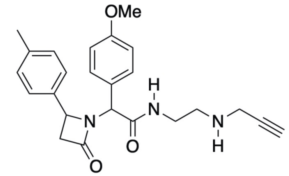

dr = 1:1

(±)-**4i2**
((±)-PJDU-215)
^{1}H NMR (500 MHz, $CDCl_3$ with TMS)

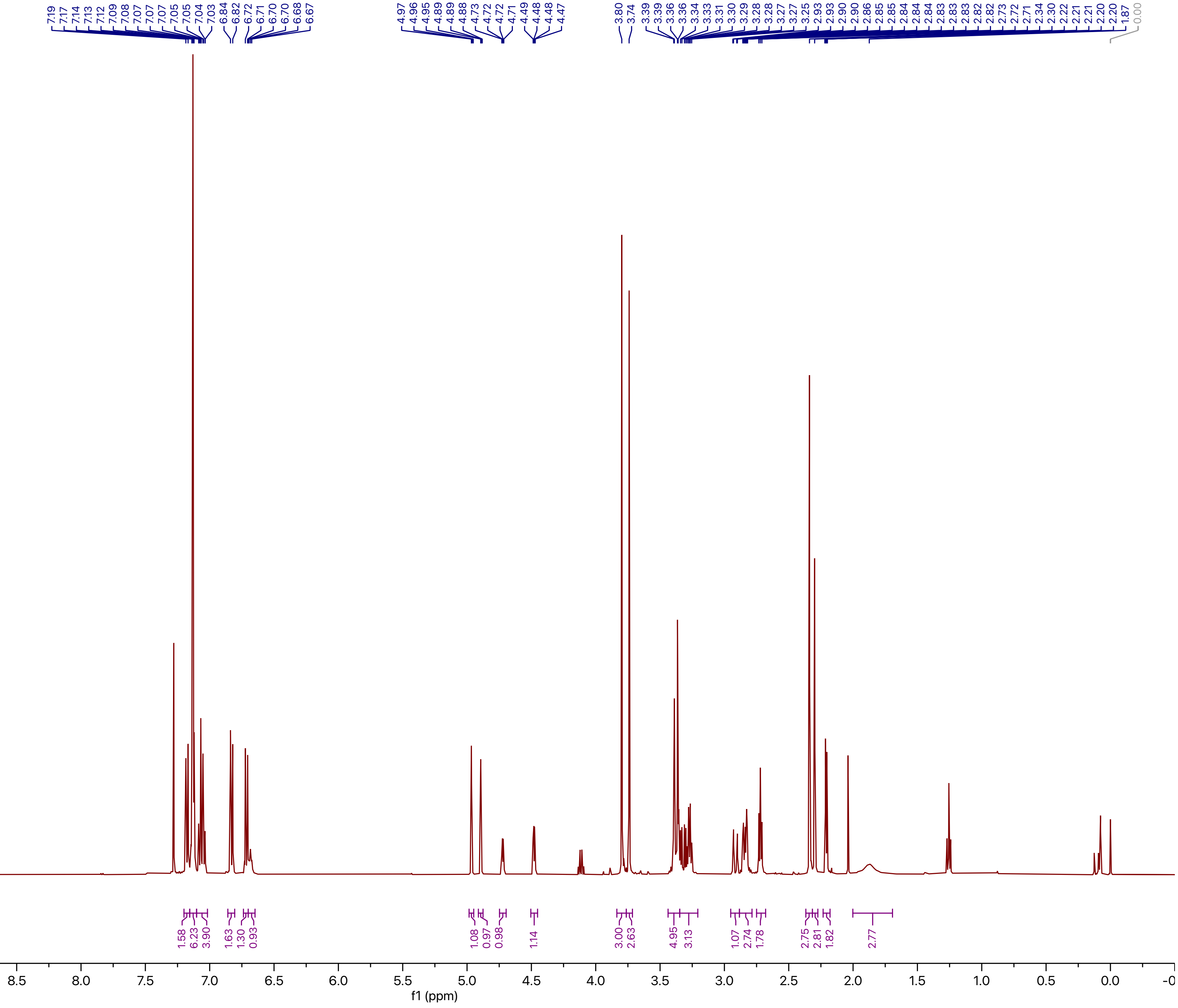

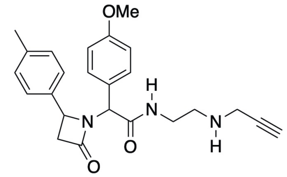

dr = 1:1

(±)-**4i2**
((±)-PJDU-215)
^{13}C NMR (126 MHz, $CDCl_3$ with TMS)

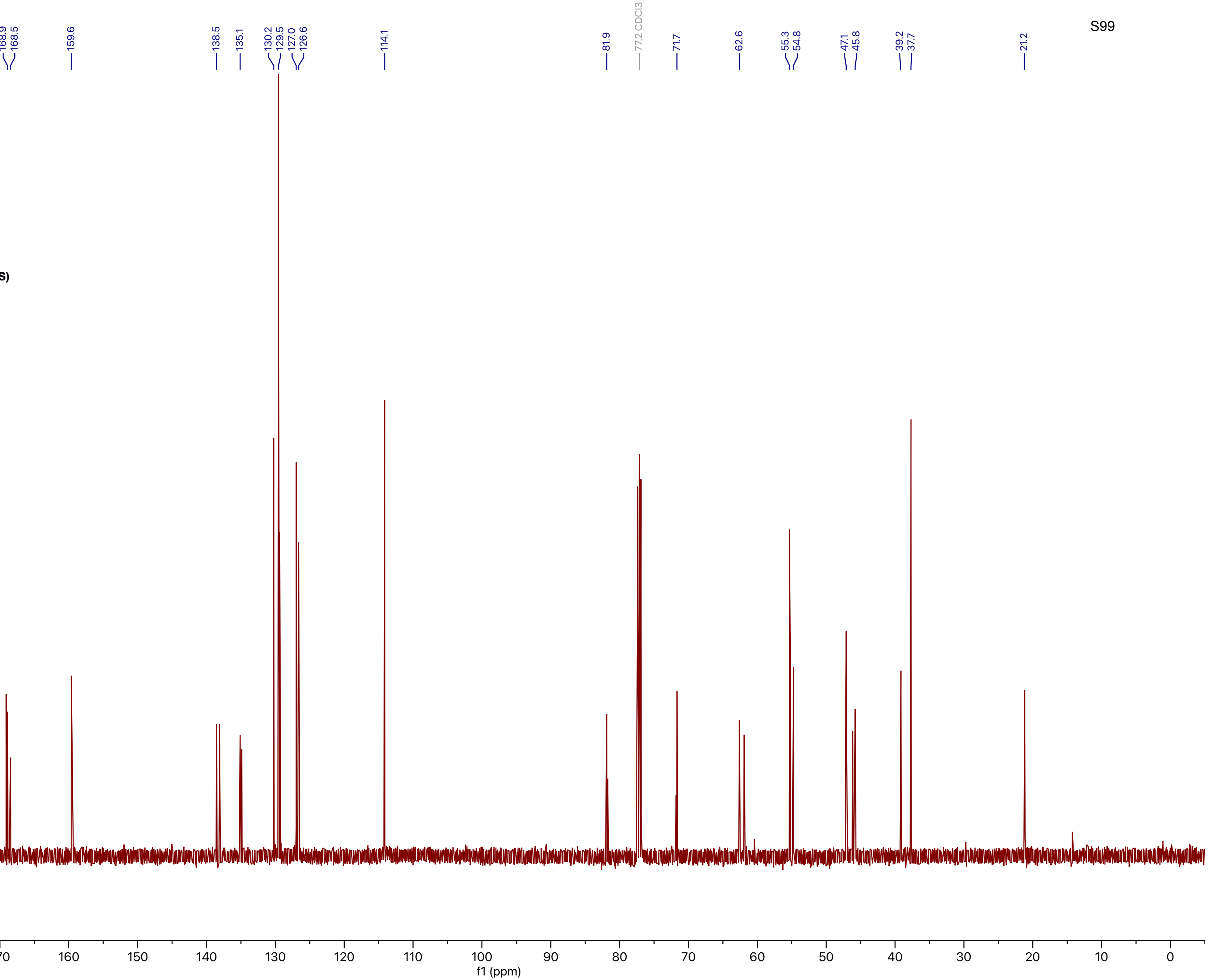

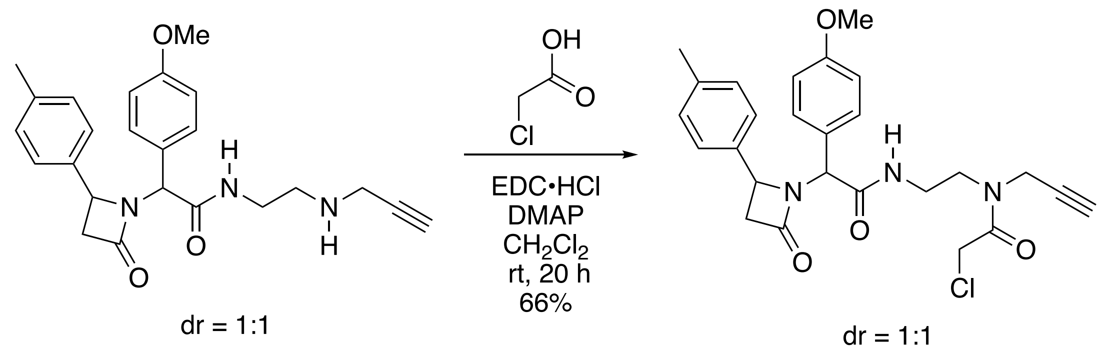

(±)-**4i2**
((±)-PJDU-215)

(±)-**4i**
((±)-PJDU-216)

*(±)-2-Chloro-N-(2-(2-(4-methoxyphenyl)-2-(2-oxo-4-(p-tolyl)azetidin-1-yl)acetamido)ethyl)-N-(prop-2-yn-1-yl)acetamide ((±)-**4i**) ((±)-PJDU-216).* A 25 mL flame-dried round-bottom flask equipped with a stir-bar, septum, and argon inlet needle was charged with 2-chloroacetic acid (31 mg, 0.33 mmol, 1.5 eq.) and CH_2Cl_2 (3.3 mL), then EDC•HCl (84 mg, 0.44 mmol, 2 eq.) and DMAP (2 mg, 0.02 mmol, 0.1 eq.) were added sequentially. Next a solution of propargyl amine (±)-**4i2** (89 mg, 0.22 mmol, 1 eq.; (±)-PJDU-215) in CH_2Cl_2 (4 mL) was added via cannula. The reaction mixture was stirred at room temperature for 20 hours, wherein TLC analysis indicated a complete reaction. Next, the reaction mixture was concentrated and then transferred to a separatory funnel with EtOAc (60 mL). The organic layers were then washed sequentially with 1M aq. HCl solution, (10 mL), H_2O (10 mL), sat. aq. $NaHCO_3$ (10 mL), and brine (10 mL) then dried ($MgSO_4$), filtered, and concentrated under reduced pressure. The crude residue was then purified by silica gel flash column chromatography (gradient elution with 75-95% EtOAc/hexanes) to give FFSMP (±)-**4i** (70 mg, 66%; (±)-PJDU-216) as a clear and colorless oil.

R_f = 0.43 (100% EtOAc)

1H NMR (500 MHz, $CDCl_3$, **peaks reported are for the major rotamer/diastereomer only) δ 7.16-7.06 (m, 7H), 6.83-6.71 (m, 2H), 4.78 (s, 1H), 4.55-4.53 (m, 1H), 4.20-3.98 (m, 4H), 3.79 (s, 3H), 3.68-3.32 (m, 5H), 2.88 (dd, *J* = 2.5, 15.0 Hz, 1H), 2.32 (s, 3H), 2.21 (t, *J* = 2.5 Hz, 1H)

^{13}C NMR (126 MHz, $CDCl_3$, **peaks reported are for the major rotamer/diastereomer only) δ 169.6, 168.7, 167.5, 159.7, 138.4, 134.8, 130.1, 129.5, 126.9, 114.1, 77.7, 73.8, 62.9, 61.8, 55.4, 54.5, 46.2, 45.6, 41.7, 37.8, 34.9, 21.2

HRMS (ESI+) *m*/*z* [M + Na] calcd for $C_{26}H_{28}ClN_3O_4Na$ 504.1661, found 504.1660

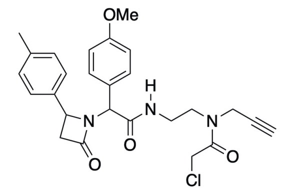

dr = 1:1

(±)-**4i**
((±)-PJDU-216)
^{1}H NMR (500 MHz, $CDCl_3$ with TMS)

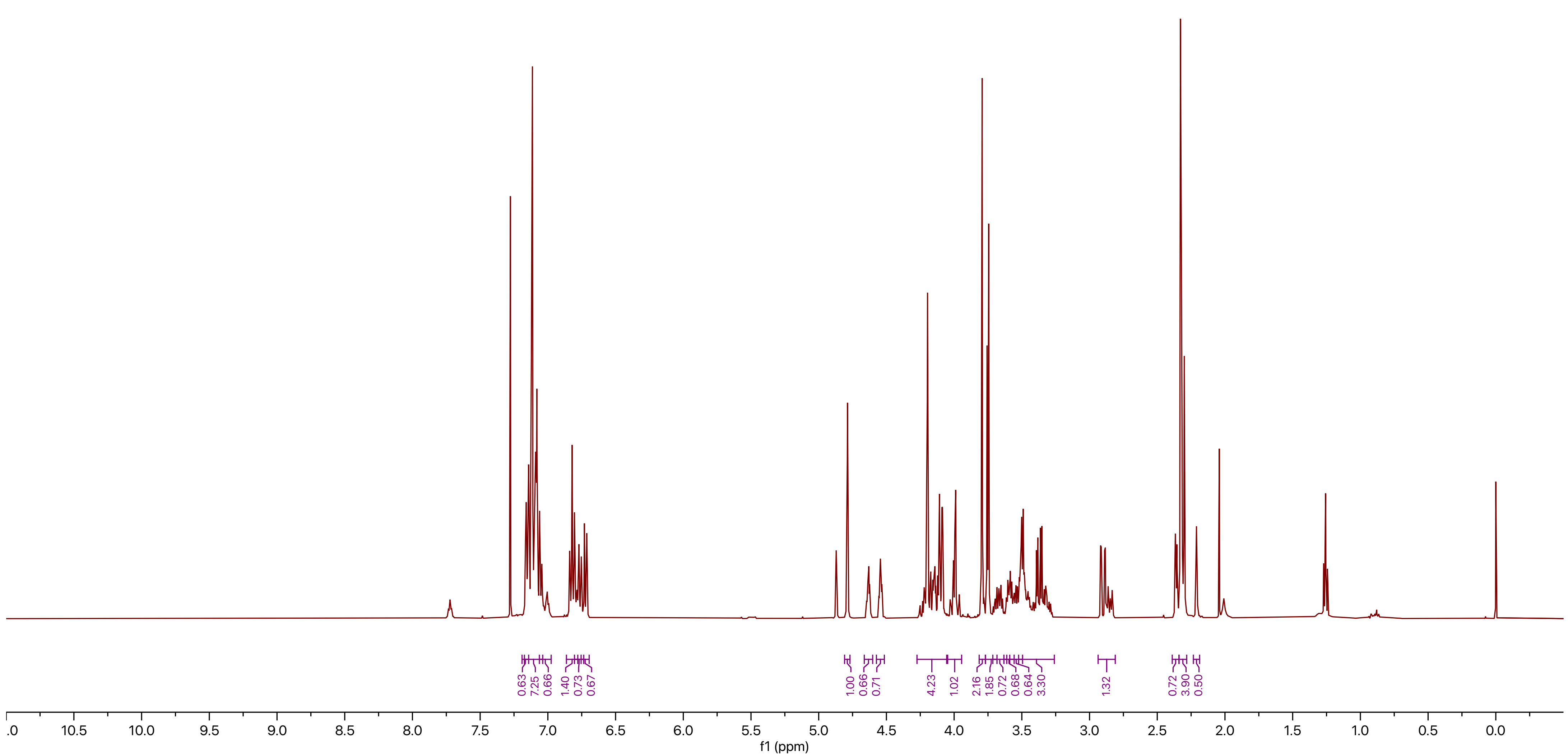

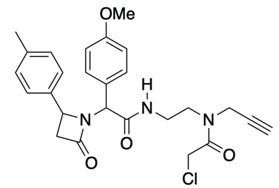

dr = 1:1

(±)-**4i**

((±)-PJDU-216)

^{13}C NMR (126 MHz, $CDCl_3$ with TMS)

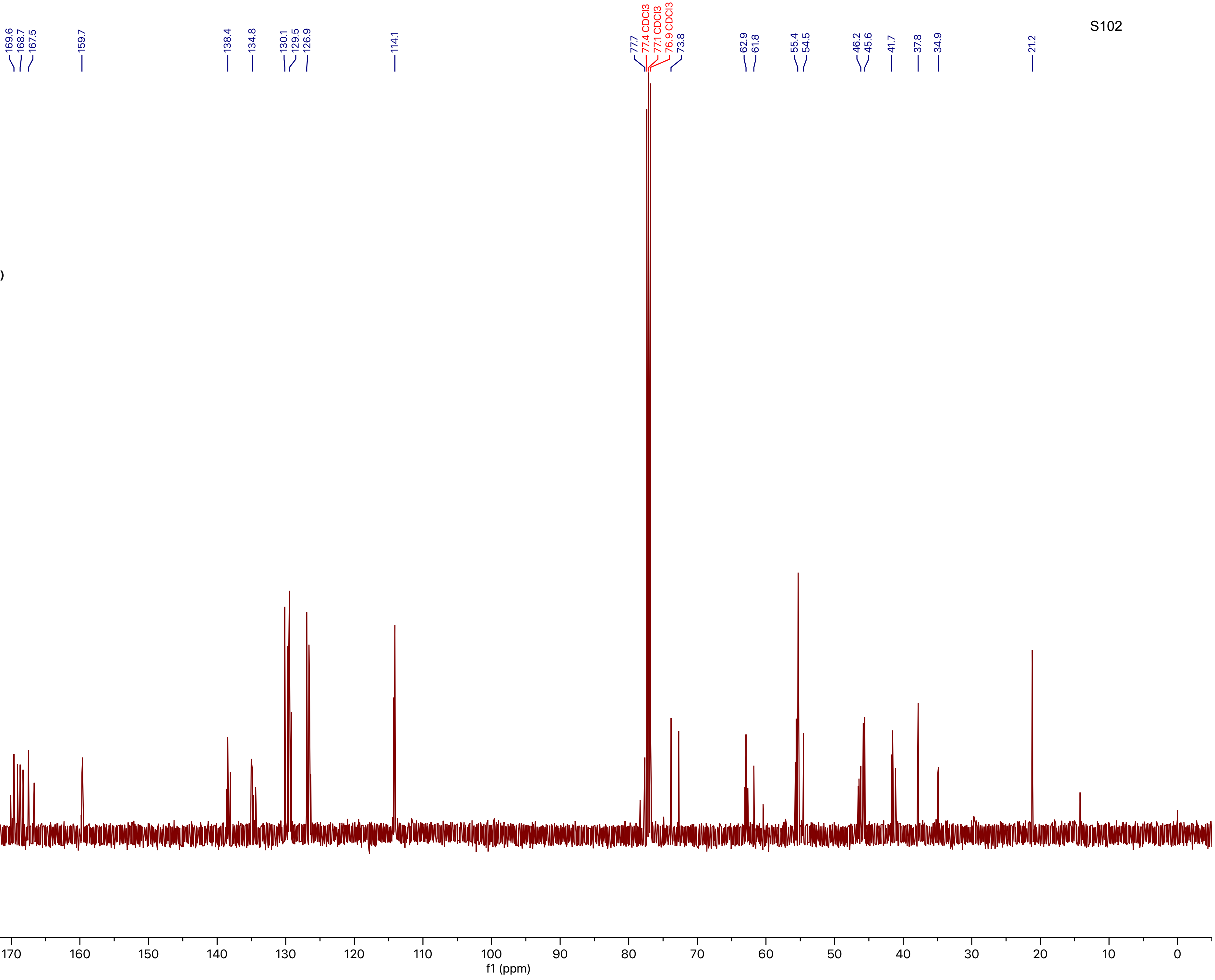

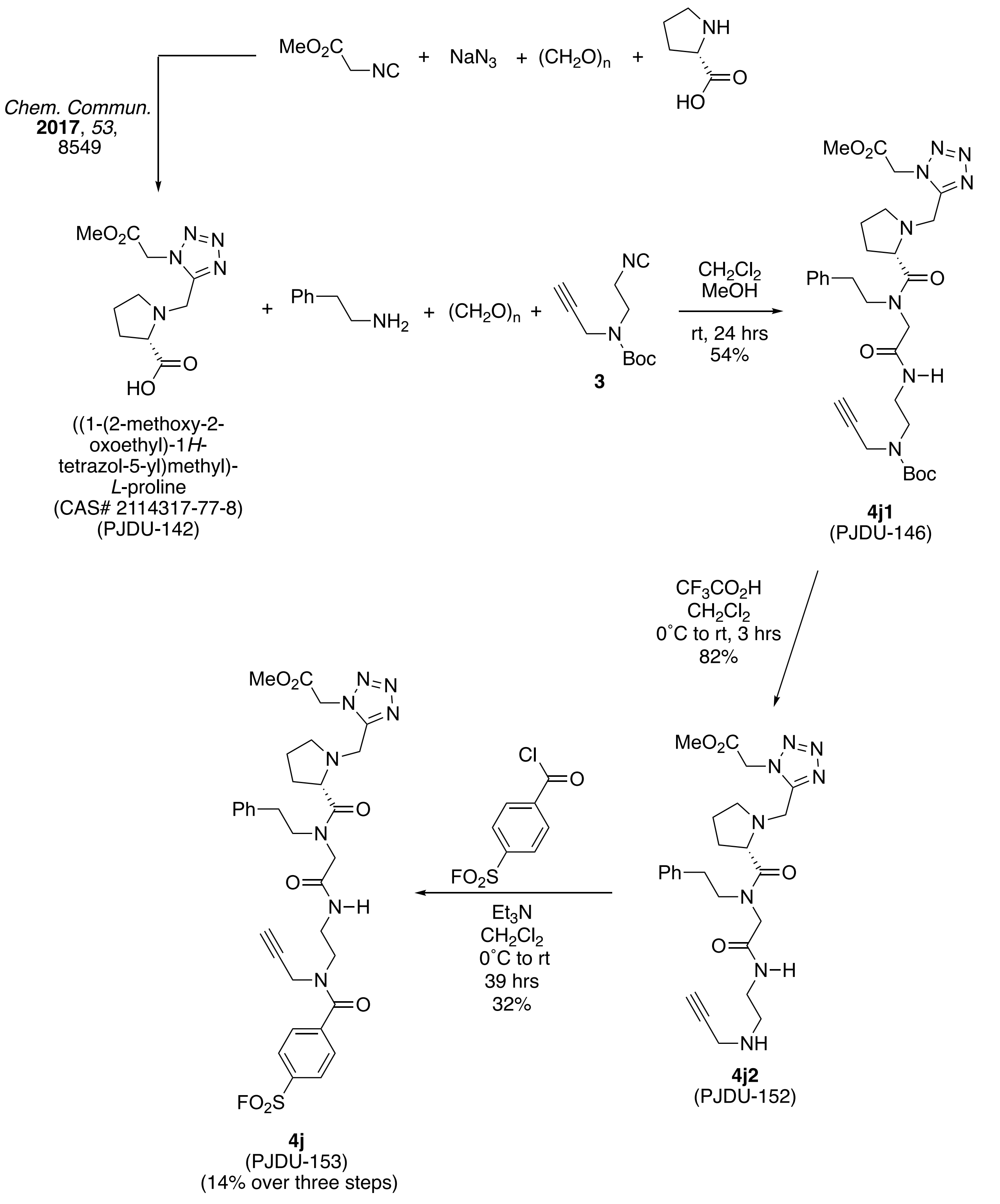

Scheme S9. *Synthesis of UT-4CR/U-4CR-based FFSMP* ***4j*** *(PJDU-153).*

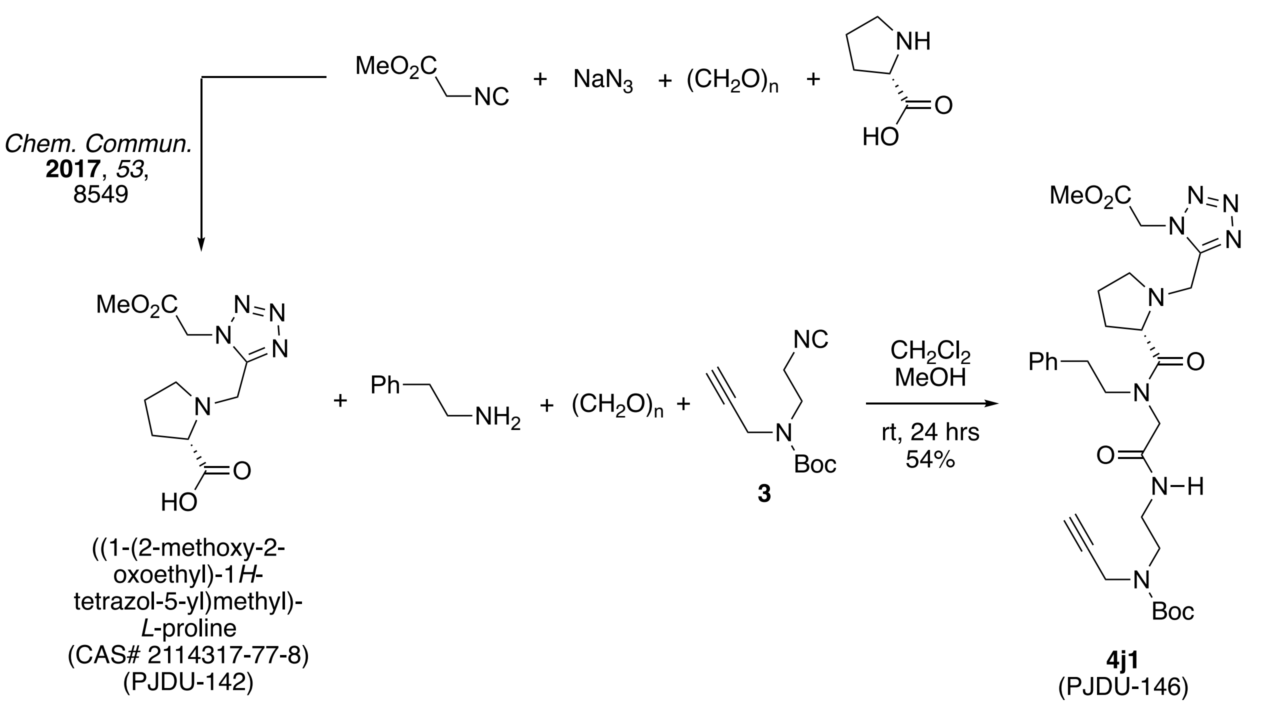

*Methyl (S)-2-(5-((2-((2-((2-((tert-butoxycarbonyl)(prop-2-yn-1-yl)amino)ethyl)amino)-2-oxoethyl)(phenethyl)carbamoyl)pyrrolidin-1-yl)methyl)-1H-tetrazol-1-yl)acetate (**4j1**) (PJDU-146).* First, carboxylic acid input ((1-(2-methoxy-2-oxoethyl)-1*H*-tetrazol-5-yl)methyl)-*L*-proline (CAS# 2114317-77-8; PJDU-142) was prepared according to *Chem. Commun.* **2017**, *53*, 8549. Next, a flame-dried, 2 dram vial equipped with a stir-bar, septum, and argon inlet needle was sequentially charged with paraformaldehyde (19 mg, 0.62 mmol, 1.0 eq.), phenethylamine (0.08 mL, 0.62 mmol, 1.0 eq.) and MeOH (0.2 mL). This mixture was stirred for 30 minutes at room temperature to form the presumed imine. Next, isocyanide **3** (1 mL of a 130 mg/mL solution in CH_2Cl_2, 0.62 mmol, 1.0 eq.) was transferred to the reaction mixture via cannula, followed by syringe addition of a solution of carboxylic acid input PJDU-142 (168 mg, 0.62 mmol, 1.0 eq.) dissolved in MeOH (0.8 mL). The reaction mixture was stirred for 24 hours at room temperature wherein TLC indicated a complete reaction. The reaction mixture was then concentrated under reduced pressure, dissolved in ~1 mL of $CHCl_3$, and purified by silica gel flash column chromatography (gradient elution with 50-100% EtOAc/hexanes, then 5% MeOH/EtOAc, to give amide **4j1** (206 mg, 54%; PJDU-146) as a white, sticky solid.

R_f = 0.07 (5% MeOH/ CH_2Cl_2)

^{1}H NMR (500 MHz, $CDCl_3$, *peaks reported are for the major rotamer only) δ 7.29-7.14 (m, 5H), 5.74-5.62 (m, 1H), 4.12-4.02 (m, 5H), 3.77 (s, 3H), 3.58-3.47 (m, 7H), 3.35-3.14 (m, 2H), 2.83-2.77 (m, 3H), 2.35-2.28 (m, 2H), 2.26 (t, J = 2.5 Hz, 1H), 2.06-2.02 (m, 1H), 1.74 (m, 3H), 1.45 (s, 9H)

^{13}C NMR (126 MHz, $CDCl_3$, *peaks reported are for the major rotamer only) δ 173.3, 168.6, 166.8, 153.5, 137.8, 128.8, 128.4, 126.9, 80.6, 79.6, 71.7, 63.9, 53.1, 52.6, 51.3, 50.2, 49.4, 48.3, 46.1, 45.7, 38.7, 37.2, 34.9, 29.3, 28.3, 23.0

HRMS (ESI+) *m/z* [M + Na] calcd for $C_{30}H_{42}N_8O_6Na$ 633.3120, found 633.3129

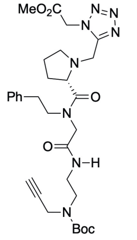

4j1
(PJDU-146)
^{1}H NMR (500 MHz, $CDCl_3$ with TMS)

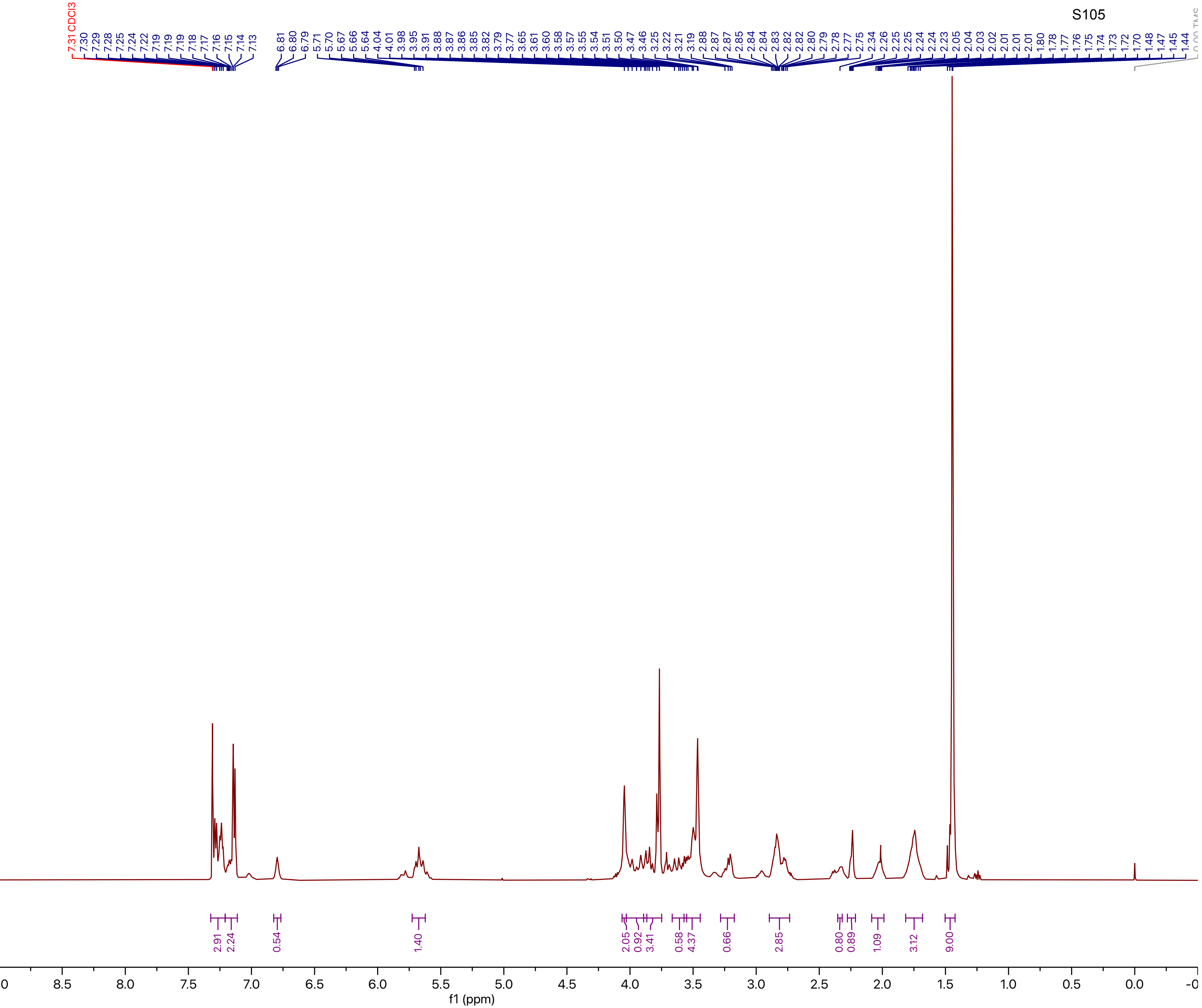

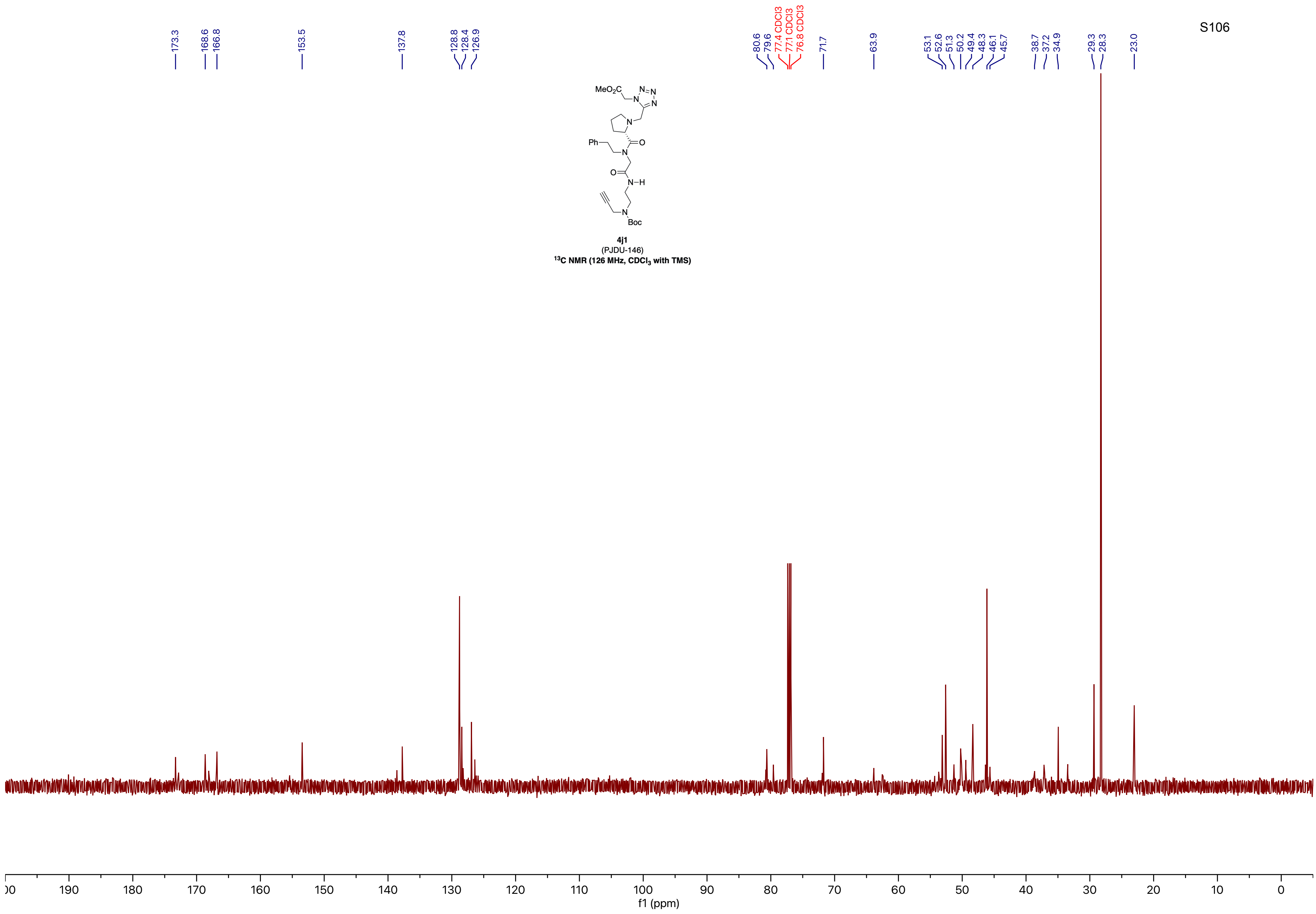

4j1
(PJDU-146)
^{13}C NMR (126 MHz, $CDCl_3$ with TMS)

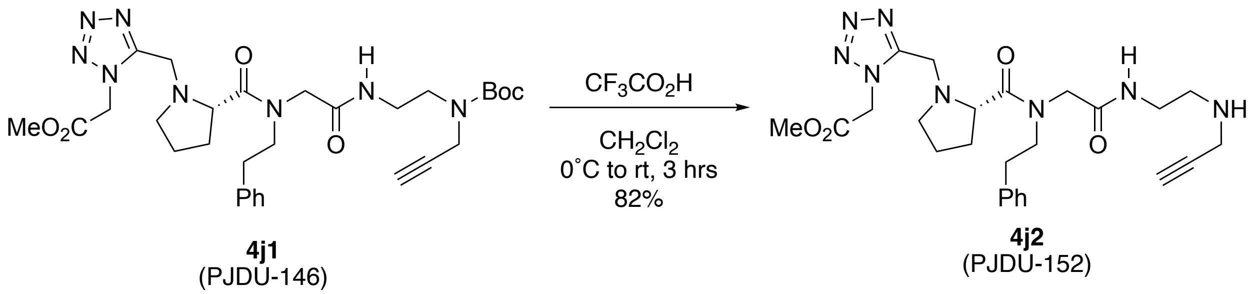

*Methyl (S)-2-(5-((2-((2-oxo-2-((2-(prop-2-yn-1-ylamino)ethyl)amino)ethyl)(phenethyl)carbamoyl)pyrrolidin-1-yl)methyl)-1H-tetrazol-1-yl)acetate (**4j2**) (PJDU-152).* A 20 mL flame-dried, scintillation vial equipped with a stirbar, septum, and argon inlet needle was charged with Boc carbamate substrate **4i1** (194 mg, 0.32 mmol, 1.0 eq.; PJDU-146) and CH_2Cl_2 (3.2 mL) and cooled to 0˚C. Trifluoroacetic acid (0.25 mL, 3.2 mmol, 10.0 eq.) was then added dropwise over 2 minutes. After the addition, the ice bath was removed and the reaction was stirred at room temperature for 3 hours, wherein TLC indicated complete consumption of the starting Boc carbamate substrate. The reaction was then quenched by dropwise addition of sat. aq. $NaHCO_3$ (vigorous gas evolution) to the reaction mixture until gas evolution ceased (~10 mL), then 1M aq. NaOH (~4 mL) was added to raise the pH to 8-9 (checked with pH paper). This biphasic mixture was then transferred to a separatory funnel and diluted with CH_2Cl_2 (15 mL) and H_2O (5 mL), the layers were separated, and the aqueous layer was extracted with CH_2Cl_2 (3 x 15 mL). The organic layers were combined, washed with brine (5 mL), dried ($MgSO_4$), gravity filtered, and concentrated under reduced pressure to give propargyl amine **4j2** (133 mg, 82%, PJDU-152) as a yellow oil, which proved sufficiently pure to take on to the N-acylation arming step.

R_f = 0.46 (10% MeOH/ CH_2Cl_2)

^{1}H NMR (400 MHz, $CDCl_3$, *peaks reported are for the major rotamer only) δ 7.23-7.19 (m, 3H), 7.18-7.06 (m, 2H), 6.79 (t, *J* = 5.4 Hz, 1H), 5.64 (ABq, *J* = 17.2 Hz, 2H), 3.92 (ABq, *J* = 15.6 Hz, 2H), 3.88-3.70 (m, 2H), 3.69 (s, 3H), 3.59-3.41 (m, 3H), 3.43-2.27 (m, 4H), 2.79-2.70 (m 5H), 2.30-2.15 (m, 1H), 2.11 (t, *J* = 2.6 Hz, 1H), 2.02-1.90 (m, 1H), 1.73-1.59 (m, 4H)

^{13}C NMR (101 MHz, $CDCl_3$, *peaks reported are for the major rotamer only) δ 173.3, 168.6, 167.0, 153.5, 137.7, 128.8, 128.7, 127.0, 81.8, 71.6, 62.7, 52.9, 52.8, 50.4, 50.3, 48.4, 47.2, 38.6, 37.6, 34.8, 29.0, 27.5, 22.9

HRMS (ESI+) *m/z* [M + Na] calcd for $C_{25}H_{34}N_8O_4Na$ 533.2595, found 533.2604

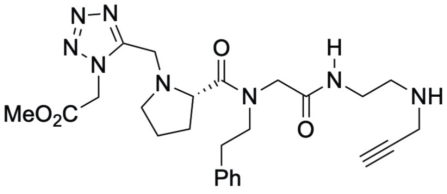

4j2

(PJDU-152)

^{1}H NMR (400 MHz, $CDCl_3$ with TMS)

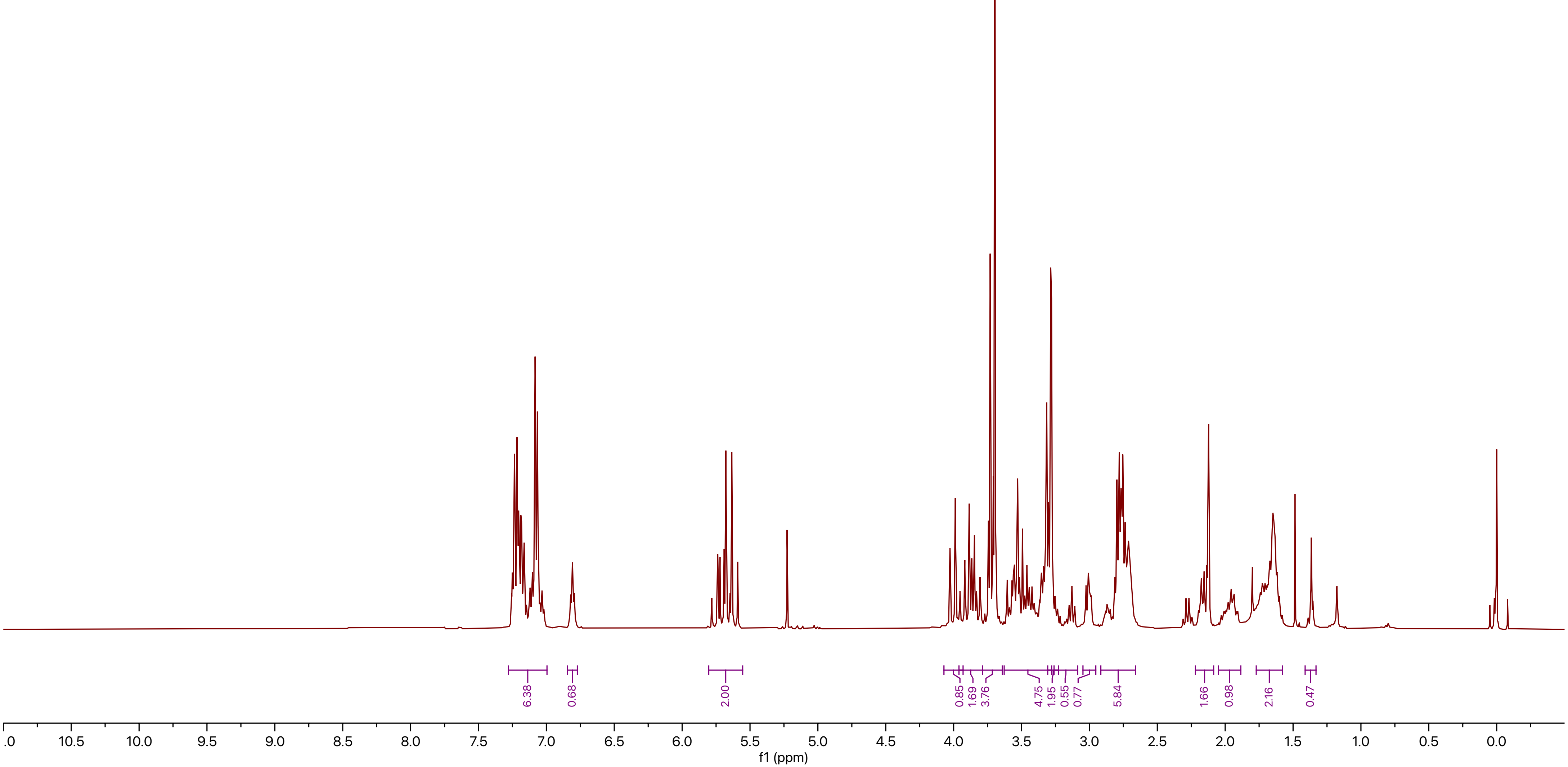

4j2
(PJDU-152)
^{13}C NMR (101 MHz, $CDCl_3$ with TMS)

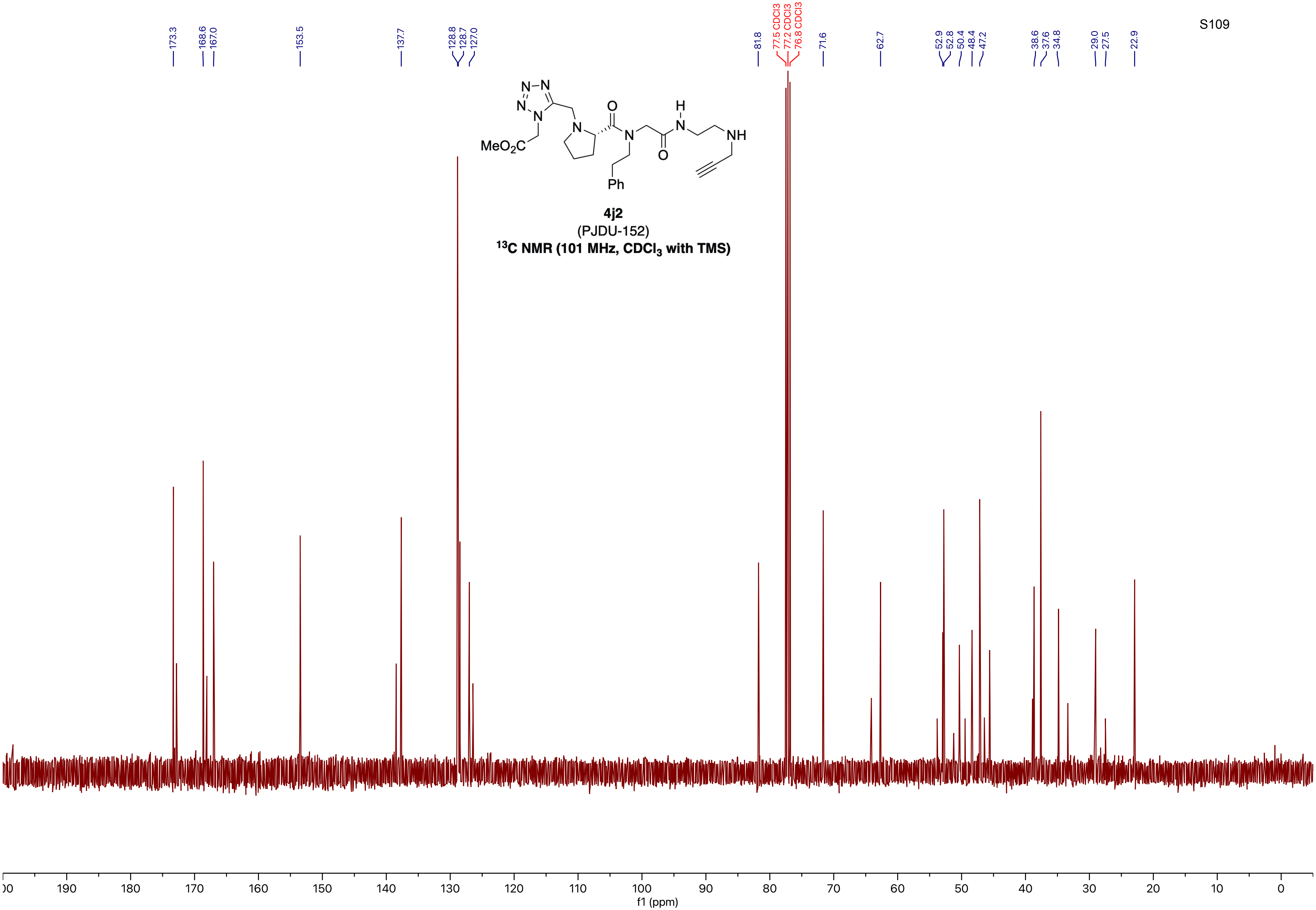

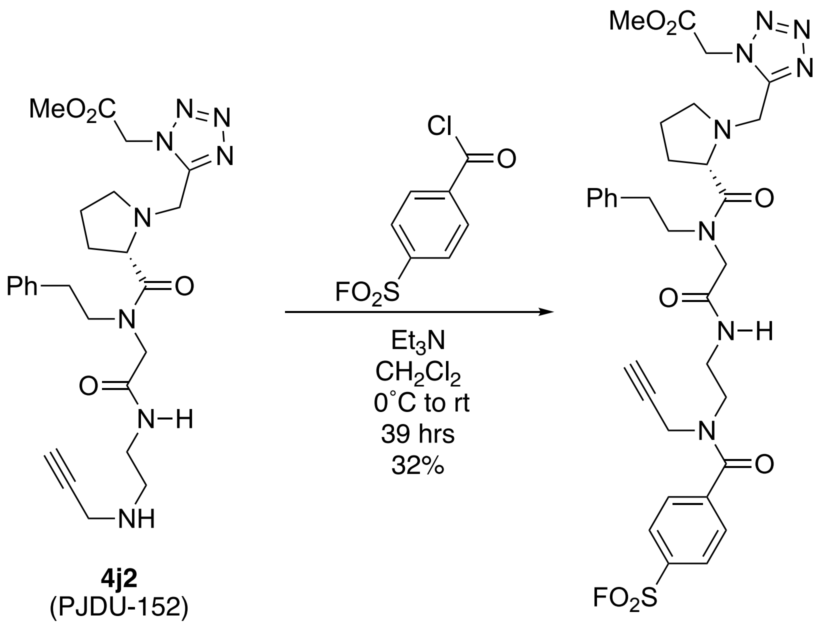

4j2
(PJDU-152)

4j
(PJDU-153)

*Methyl (S)-2-(5-((2-((2-((2-(4-(fluorosulfonyl)-N-(prop-2-yn-1-yl)benzamido)ethyl)amino)-2-oxoethyl)(phenethyl)carbamoyl)pyrrolidin-1-yl)methyl)-1H-tetrazol-1-yl)acetate (**4j**) (PJDU-153).* A 10 mL flame-dried round-bottom flask equipped with a stir-bar, septum, and argon inlet needle was sequentially charged with propargyl amine **4j2** (130 mg, 0.26 mmol, 1.0 eq., PJDU-152), CH_2Cl_2 (5 mL), and triethylamine (0.05 mL, 0.33 mmol, 1.3 eq.). The mixture was then cooled to 0˚C and treated with 4-(fluorosulfonyl)benzoyl chloride (110 mg, 0.50 mmol, 1.5 eq.). After the addition, the reaction was slowly warmed to room temperature and stirred for 20 hours, wherein TLC analysis indicated an incomplete reaction. Therefore, additional 4-(fluorosulfonyl)benzoyl chloride (20 mg, 0.09 mmol, 0.35 eq.) was added at this time, followed by stirring for additional 19 hours at room temperature. Next, the reaction mixture was quenched with H_2O (5 mL) and diluted with CH_2Cl_2 (10 mL). The separated organic layer was then washed sequentially with 1M sat. aq. $NaHCO_3$ (10 mL) and brine (10 mL), then dried ($MgSO_4$), filtered, and concentrated under reduced pressure. The crude residue was then purified by silica gel flash column chromatography (gradient elution with 2-5% MeOH/CH_2Cl_2) to give FFSMP **4j** (57 mg, 32%, PJDU-153) as a clear and colorless oil.

R_f = 0.45 (% 5% MeOH/CH_2Cl_2)

1H NMR (500 MHz, $CDCl_3$, **peaks reported are for the major rotamer only) δ 8.03 (dd, *J* = 8.2, 1.9 Hz, 1H), 7.71 (t, *J* = 7.7 Hz, 2H), 7.37 – 7.16 (m, 4H), 7.16 – 7.01 (m, 2H), 5.81 – 5.55 (m, 1H), 4.18 – 3.88 (m, 3H), 3.88 – 3.76 (m, 5H), 3.76 – 3.35 (m, 4H), 3.29 (dd, *J* = 8.8, 6.2 Hz, 1H), 3.24 – 2.96 (m, 1H), 2.96 – 2.57 (m, 3H), 2.57 – 2.28 (m, 1H), 2.12 – 1.85 (m, 1H), 1.85 – 1.55 (m, 2H)

^{13}C NMR (101 MHz, $CDCl_3$, **peaks reported are for the major rotamer only) δ 173.7, 169.8, 169.3, 167.0, 153.7, 129.0, 128.9, 128.8, 128.7, 128.2, 128.1, 127.2, 126.7, 77.8, 74.2, 61.8, 53.6, 53.0, 50.6, 50.3, 48.5, 45.4, 45.0, 39.6, 37.8, 35.1, 29.6, 23.1

HRMS (ESI+) *m/z* [M + Na] calcd for $C_{32}H_{37}FN_8O_7SNa$ 719.2382, found 719.2379

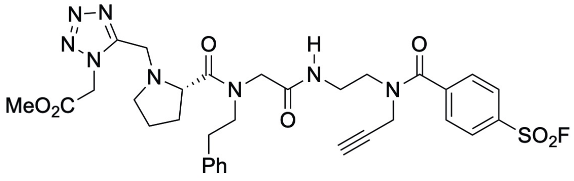

4j
(PJDU-153)
^{1}H NMR (500 MHz, $CDCl_3$ with TMS)

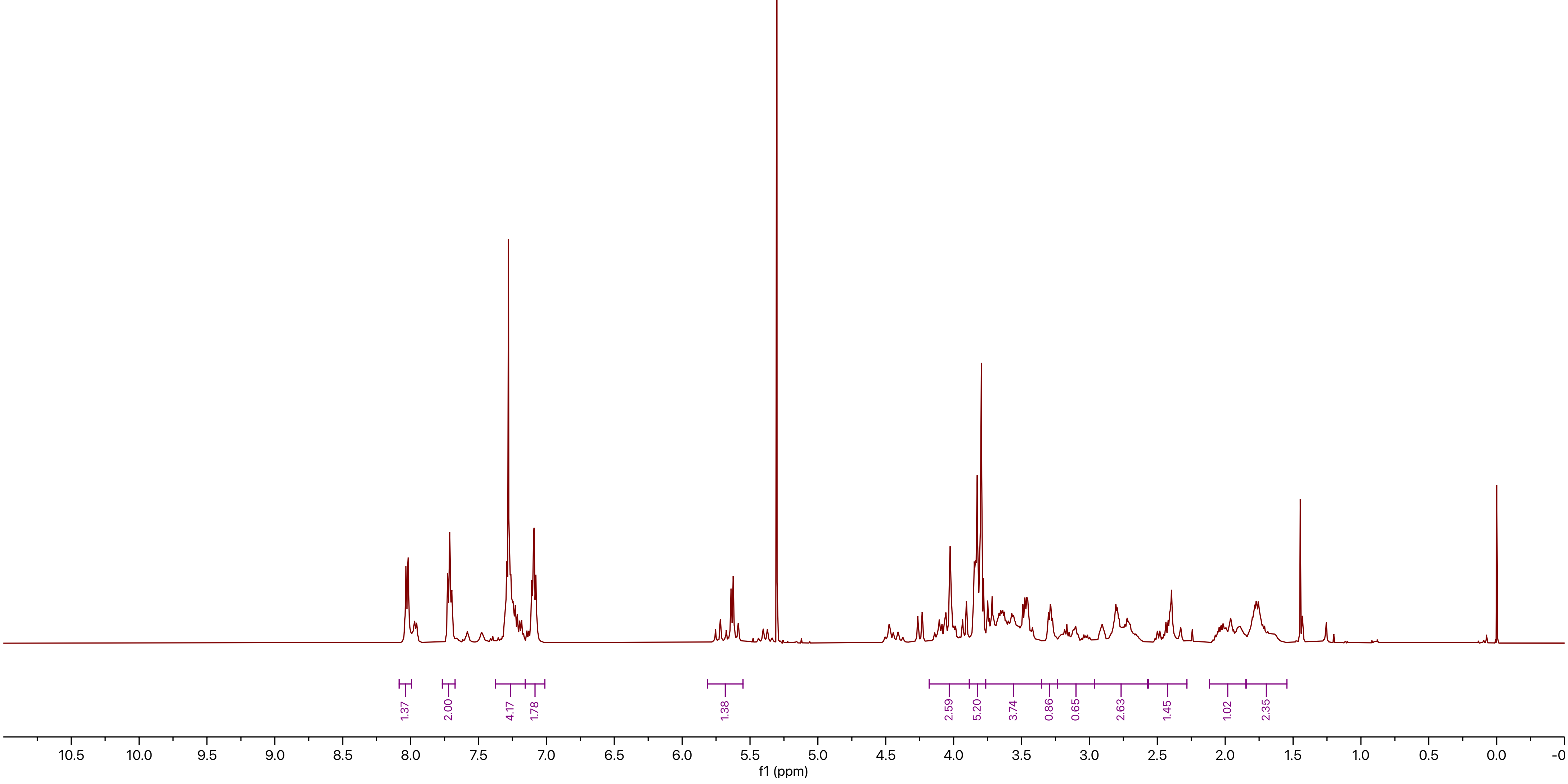

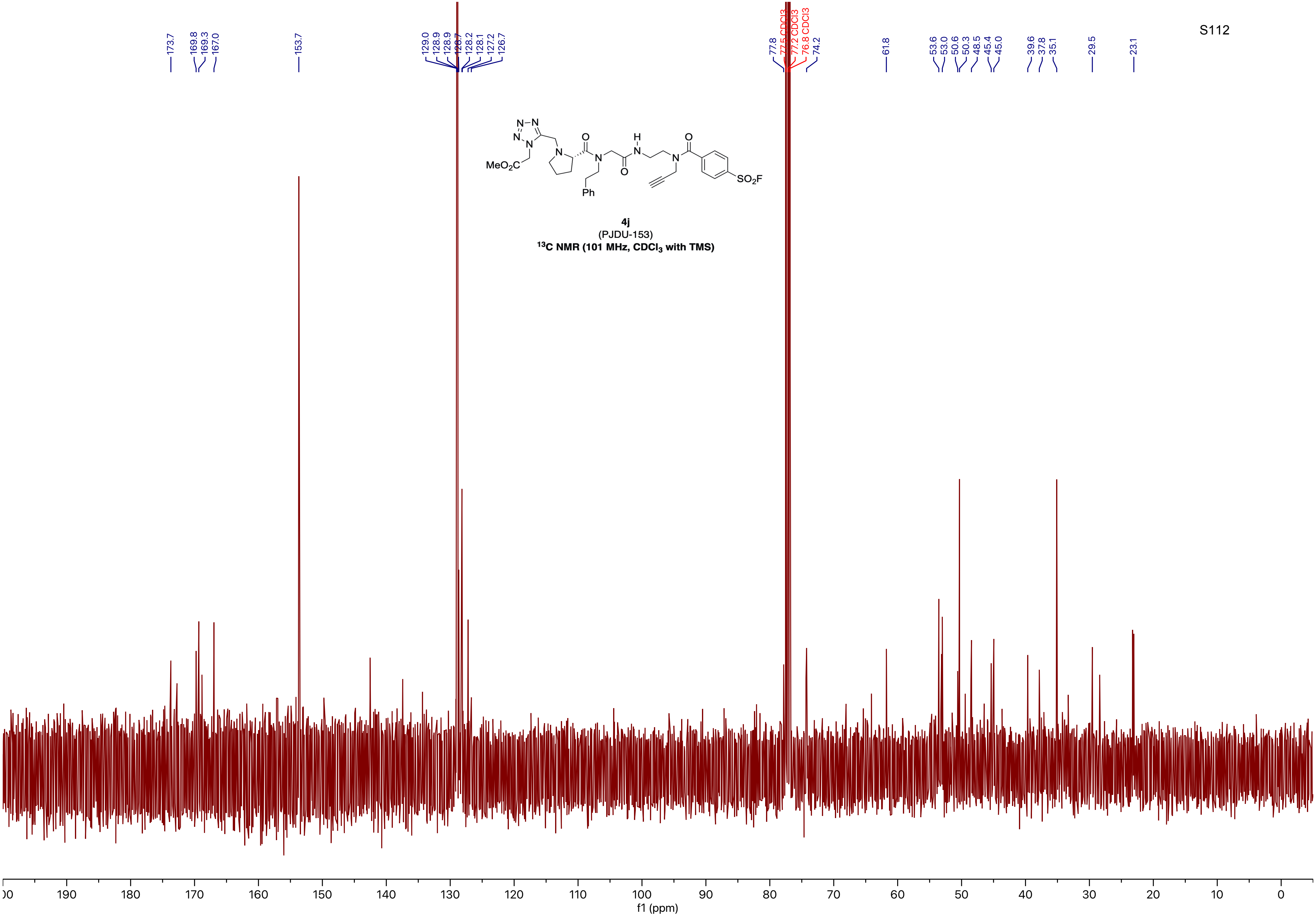
173.7
169.8
169.3
167.0
153.7
129.0
128.9
128.9
128.7
128.2
128.1
127.2
126.7
77.8
77.5 CDCl3
77.2 CDCl3
76.8 CDCl3
74.2
61.8
53.6
53.0
50.6
50.3
48.5
45.4
45.0
39.6
37.8
35.1
29.5
23.1
MeO2C
Ph
SO2F
4j
(PJDU-153)
13C NMR (101 MHz, CDCl3 with TMS)
f1 (ppm)

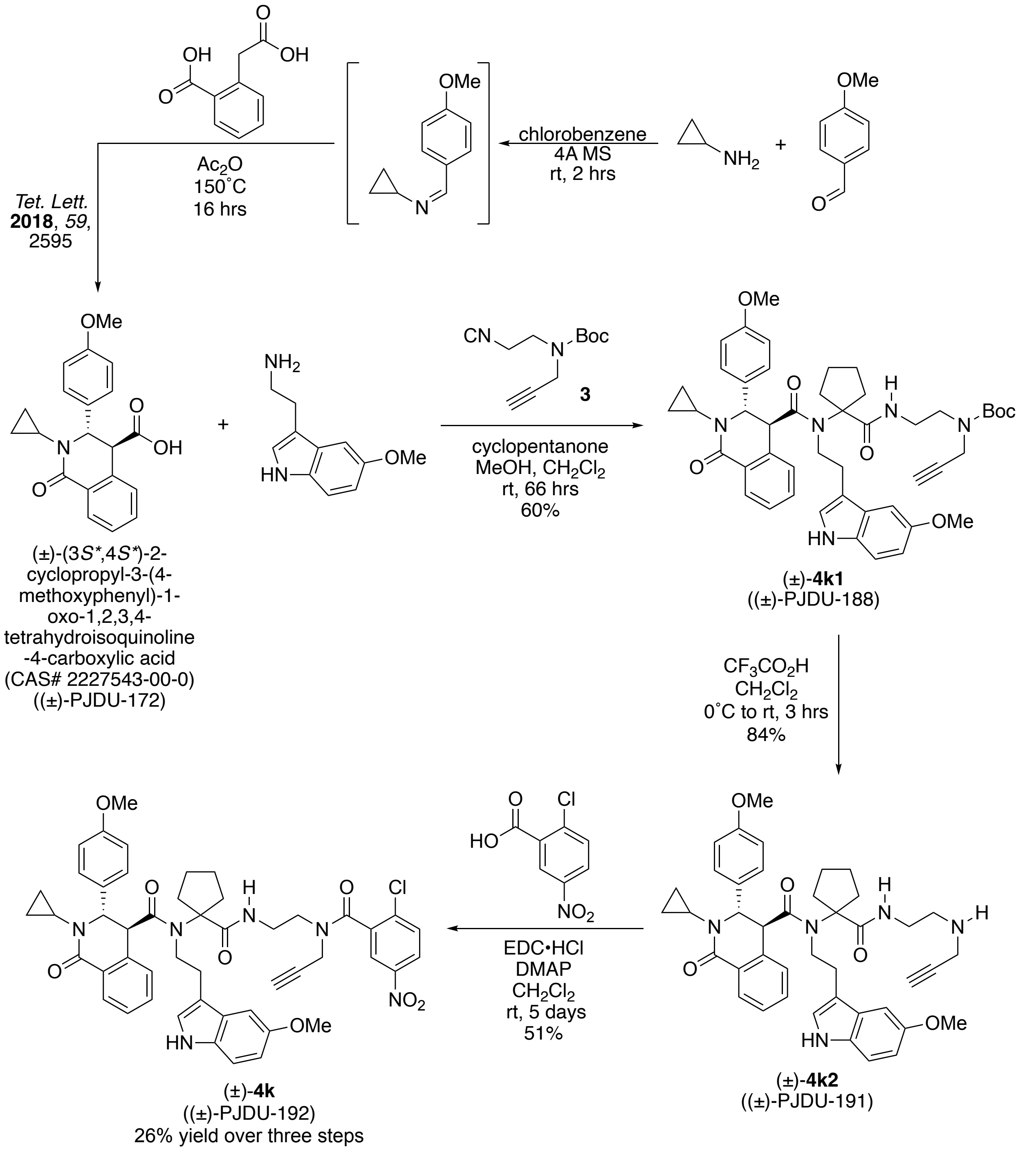

Scheme S10. *Synthesis of Castagnoli-Cushman/U-4CR-based FFSMP (±)-**4k** ((±)-PJDU-192).*

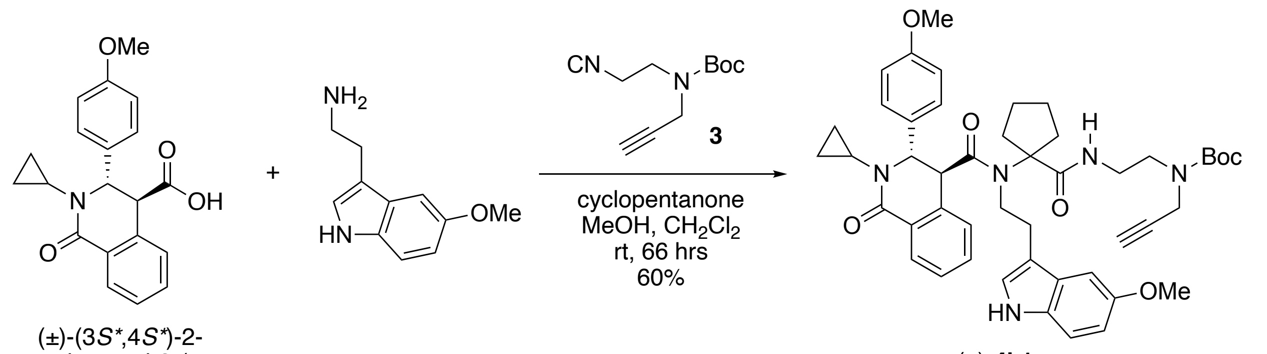

(±)-(3*S**,4*S**)-2-cyclopropyl-3-(4-methoxyphenyl)-1-oxo-1,2,3,4-tetrahydroisoquinoline-4-carboxylic acid (CAS# 2227543-00-0) ((±)-PJDU-172)

(±)-**4k1** ((±)-PJDU-188)

(±)-tert-Butyl (2-(1-((3S,4S*)-2-cyclopropyl-N-(2-(5-methoxy-1H-indol-3-yl)ethyl)-3-(4-methoxyphenyl)-1-oxo-1,2,3,4-tetrahydroisoquinoline-4-carboxamido)cyclopentane-1-carboxamido)ethyl)(prop-2-yn-1-yl)carbamate ((±)-**4k1**) ((±)-PJDU-188).* First, carboxylic acid input (±)-(3*S**,4*S**)-2-cyclopropyl-3-(4-methoxyphenyl)-1-oxo-1,2,3,4-tetrahydroisoquinoline-4-carboxylic acid (CAS# 2227543-00-0; (±)-PJDU-172) was prepared according to *Tetrahedron Letters* **2018**, *59*, 2595. Next, a flame-dried, 10 mL round-bottom flask equipped with a stir-bar, septum, and argon inlet needle was sequentially charged with 5-methoxytryptamine (137 mg, 0.72 mmol, 1.0 eq.), cyclopentanone (65 uL, 0.72 mmol, 1.0 eq.), and MeOH (1.8 mL). This mixture was stirred for 30 minutes at room temperature to form the presumed imine. Next, carboxylic acid input (±)-PJDU-172 (243 mg, 0.72 mmol, 1.0 eq.) was added, followed by the addition of isocyanide **3** (160 mg, 0.77 mmol, 1.05 eq.). The reaction mixture was then stirred for 66 hours at room temperature, wherein TLC indicated a complete reaction. The reaction mixture was subsequently concentrated under reduced pressure, dissolved in EtOAc (50 mL), and then sequentially washed with sat. aq. $NaHCO_3$ (15 mL), H_2O (15 mL), and brine (15 mL). The separated organic layer was then dried ($MgSO_4$), filtered, concentrated, and purified by silica gel flash column chromatography (gradient elution with 60-75% EtOAc/hexanes) to give amide **4k1** (348 mg, 60%, (±)-PJDU-188) as a sticky white solid.

R_f = 0.37 (75% EtOAc/hexanes)

^{1}H NMR (400 MHz, $CDCl_3$, *peaks reported are for the major rotamer only) δ 9.22 (s, 1H), 8.18 – 8.11 (m, 1H), 7.34 – 7.23 (m, 3H), 7.02 – 6.92 (m, 4H), 6.90 – 6.79 (m, 2H), 6.83 – 6.75 (m, 1H), 6.70 – 6.62 (m, 2H), 4.88 (s, 1H), 3.90 (s, 4H), 3.69 (s, 3H), 3.59 (s, 3H), 3.38 (s, 4H), 3.17 (t, *J* = 7.7 Hz, 2H), 2.63 (s, 2H), 2.50 (s, 2H), 2.19 (d, *J* = 2.6 Hz, 1H), 1.97 – 1.86 (m, 2H), 1.72 (s, 5H), 1.44 (s, 9H), 0.92 (t, *J* = 7.8 Hz, 1H), 0.78 (dt, *J* = 9.7, 4.9 Hz, 1H), 0.60 (s, 2H)

^{13}C NMR (101 MHz, $CDCl_3$, *peaks reported are for the major rotamer only) δ 165.8, 159.1, 159.0, 154.0, 132.0, 131.6, 130.8, 127.9, 127.5, 127.2, 123.5, 114.1, 112.6, 112.3, 111.1, 99.7, 80.8, 79.6, 73.3, 71.7, 63.1, 60.4, 55.9, 55.5, 55.2, 49.2, 47.6, 45.8, 39.6, 37.1, 36.3, 35.9, 30.1, 28.3, 23.5, 8.8, 6.9

HRMS (ESI+) *m/z* [M + H] calcd for $C_{47}H_{55}N_5O_7H$ 802.4174, found 802.4168

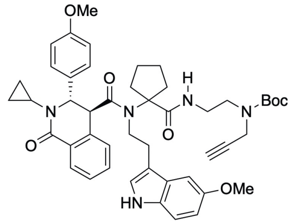

(±)-**4k1**
((±)-PJDU-188)
^{1}H NMR (400 MHz, $CDCl_3$ with TMS)

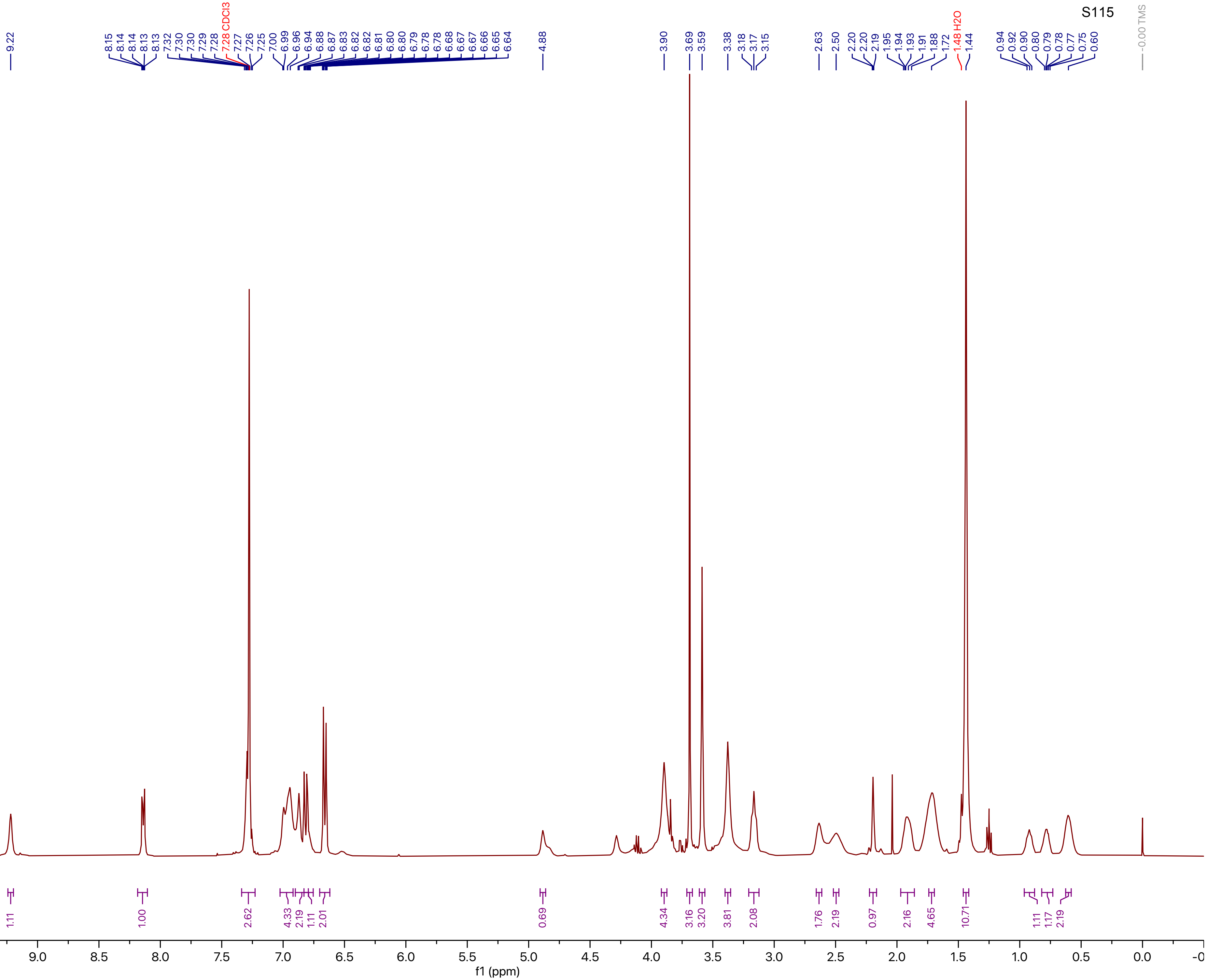

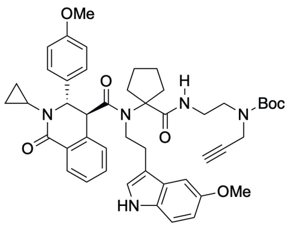

(±)-**4k1**
((±)-PJDU-188)
^{13}C NMR (101 MHz, CDCl$_3$ with TMS)

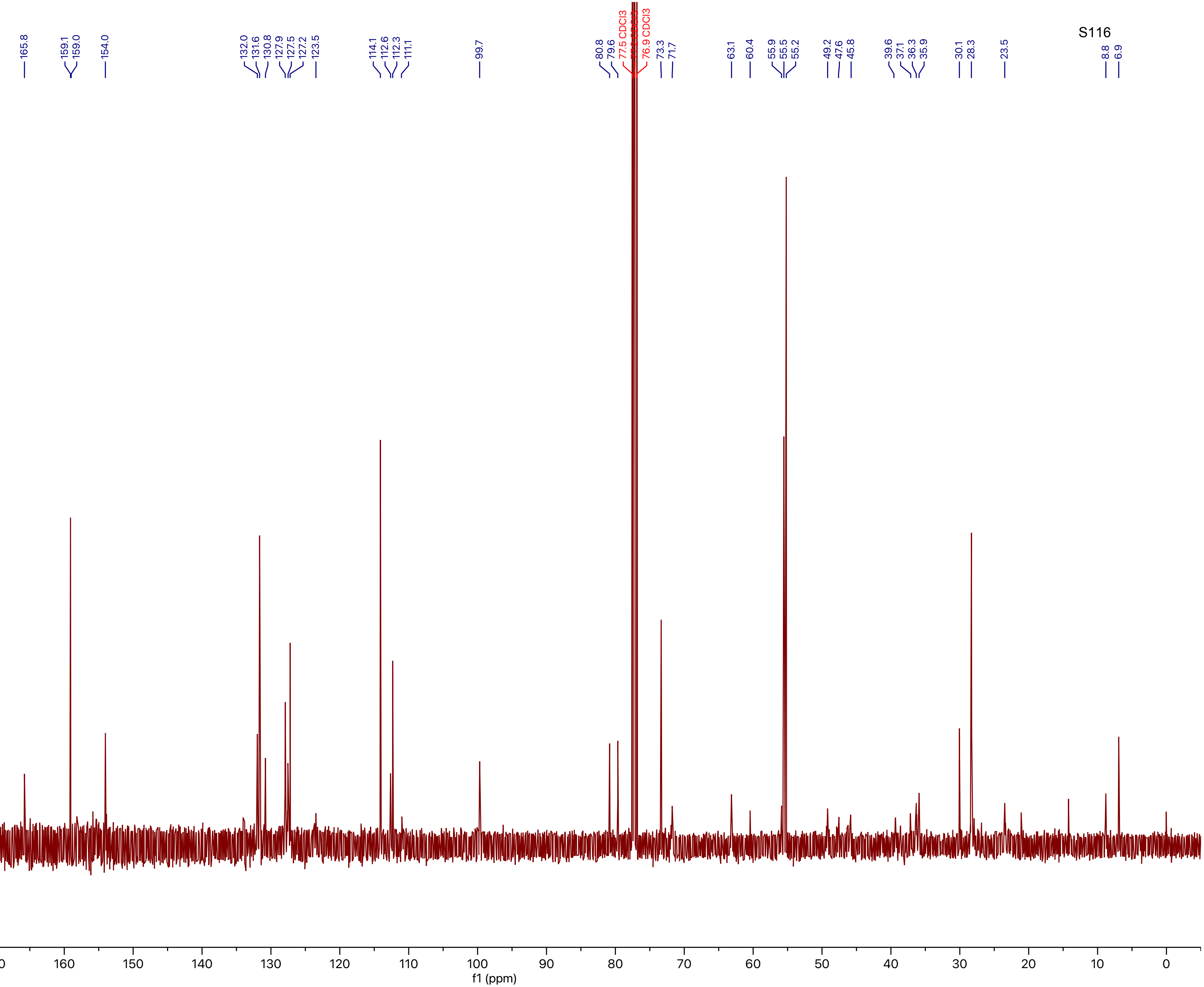

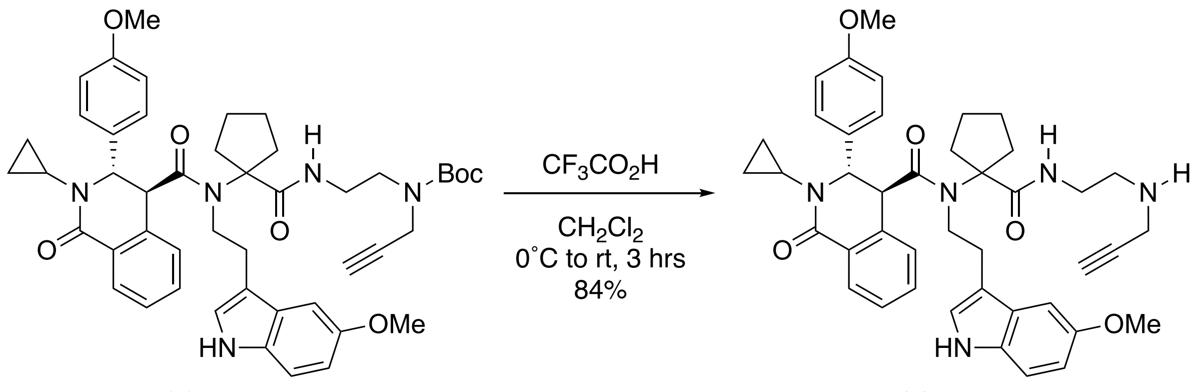

(±)-**4k1**
((±)-PJDU-188)

(±)-**4k2**
((±)-PJDU-191)

(±)-(3S,4S*)-2-Cyclopropyl-N-(2-(5-methoxy-1H-indol-3-yl)ethyl)-3-(4-methoxyphenyl)-1-oxo-N-(1-((2-(prop-2-yn-1-ylamino)ethyl)carbamoyl)cyclopentyl)-1,2,3,4-tetrahydroisoquinoline-4-carboxamide ((±)-**4k2**) ((±)-PJDU-191).* A 20 mL flame-dried, scintillation vial equipped with a stirbar, septum, and argon inlet needle was charged with Boc carbamate substrate (±)-**4k1** (345 mg, 0.43 mmol, 1.0 eq.; (±)-PJDU-188) and CH_2Cl_2 (4.3 mL) and cooled to 0˚C. Trifluoroacetic acid (0.40 mL, 5.2 mmol, 12.0 eq.) was then added dropwise over 2 minutes. After the addition, the ice bath was removed and the reaction was stirred at room temperature for 3 hours, wherein TLC indicated complete consumption of the starting Boc carbamate substrate. The reaction was then quenched by dropwise addition of sat. aq. $NaHCO_3$ (vigorous gas evolution) to the reaction mixture until gas evolution ceased (~10 mL), then 1M aq. NaOH (~4 mL) was added to raise the pH to 8-9 (checked with pH paper). This biphasic mixture was then transferred to a separatory funnel and diluted with CH_2Cl_2 (15 mL) and H_2O (5 mL), the layers were separated, and the aqueous layer was extracted with CH_2Cl_2 (3 x 15 mL). The organic layers were combined, washed with brine (5 mL), dried ($MgSO_4$), gravity filtered, and concentrated under reduced pressure to give propargyl amine **4k2** (252 mg, 84%, (±)-PJDU-191) as an off-white sticky, which proved sufficiently pure to take on to the N-acylation arming step.

R_f = 0.40 (10% MeOH/CH_2Cl_2)

^{1}H NMR (400 MHz, $CDCl_3$, *peaks reported are for the major rotamer only) δ 8.89 (s, 1H), 8.18 – 8.10 (m, 1H), 7.42 – 7.25 (m, 3H), 7.01 (dd, *J* = 7.6, 2.5 Hz, 1H), 6.99 – 6.82 (m, 4H), 6.86 – 6.79 (m, 1H), 6.73 (dd, *J* = 6.1, 2.4 Hz, 1H), 6.68 – 6.57 (m, 2H), 4.84 (d, *J* = 2.9 Hz, 1H), 4.24 (d, *J* = 3.0 Hz, 1H), 3.98 – 3.80 (m, 3H), 3.70 (d, *J* = 1.1 Hz, 3H), 3.62 (dd, *J* = 18.4, 1.2 Hz, 3H), 3.35 – 3.21 (m, 4H), 3.24 – 3.08 (m, 2H), 2.72 (t, *J* = 5.8 Hz, 2H), 2.63 (tt, *J* = 11.4, 5.2 Hz, 2H), 2.56 – 2.48 (m, 1H), 2.17 (t, *J* = 2.4 Hz, 1H), 1.91 (td, *J* = 13.9, 6.4 Hz, 1H), 1.74 (dd, *J* = 12.1, 5.0 Hz, 1H), 0.99 – 0.84 (m, 1H), 0.78 (dt, *J* = 12.1, 4.8 Hz, 1H), 0.67 – 0.52 (m, 2H)

^{13}C NMR (101 MHz, $CDCl_3$, *peaks reported are for the major rotamer only) δ 174.4, 172.5, 165.8, 159.1, 154.2, 134.0, 132.0, 131.7, 131.6, 131.0, 130.5, 128.0, 127.5, 127.3, 127.1, 123.5, 114.2, 112.6, 112.5, 111.2, 99.8, 81.8, 73.6, 71.8, 70.6, 63.2, 55.6, 55.2, 53.5, 49.3, 47.1, 39.2, 37.6, 35.9, 30.1, 27.6, 23.2, 8.8, 6.9

HRMS (ESI+) *m/z* [M + Na] calcd for $C_{42}H_{47}N_5O_5H$ 702.3650, found 702.3644

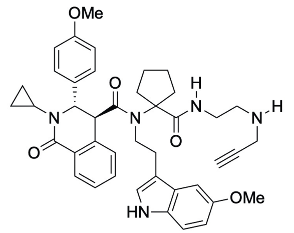

(±)-**4k2**
((±)-PJDU-191)
^{1}H NMR (400 MHz, $CDCl_3$ with TMS)

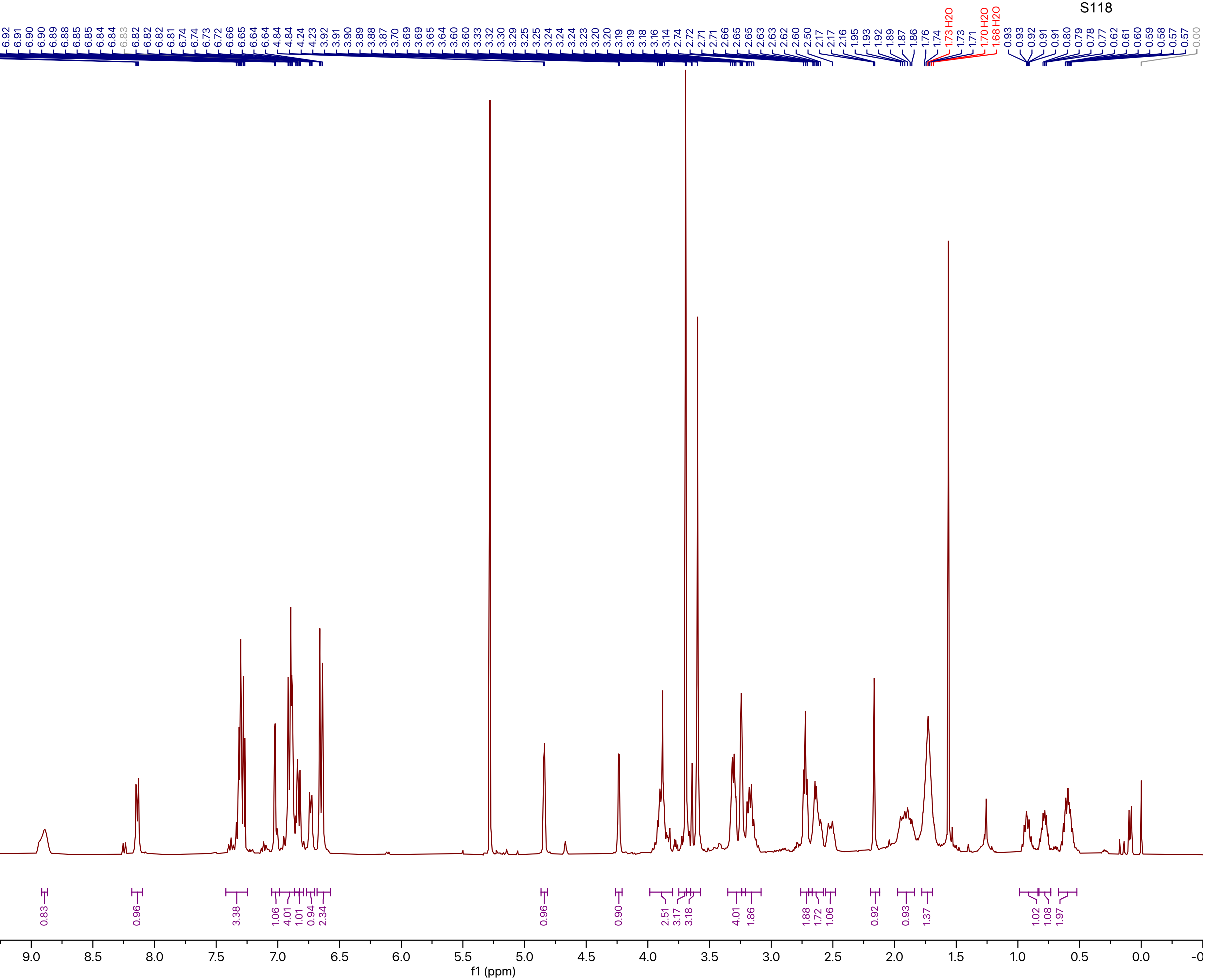

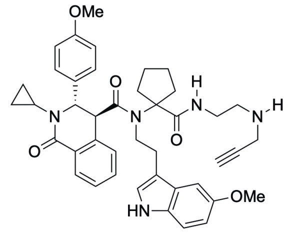

(±)-**4k2**
((±)-PJDU-191)
^{13}C NMR (101 MHz, $CDCl_3$ with TMS)

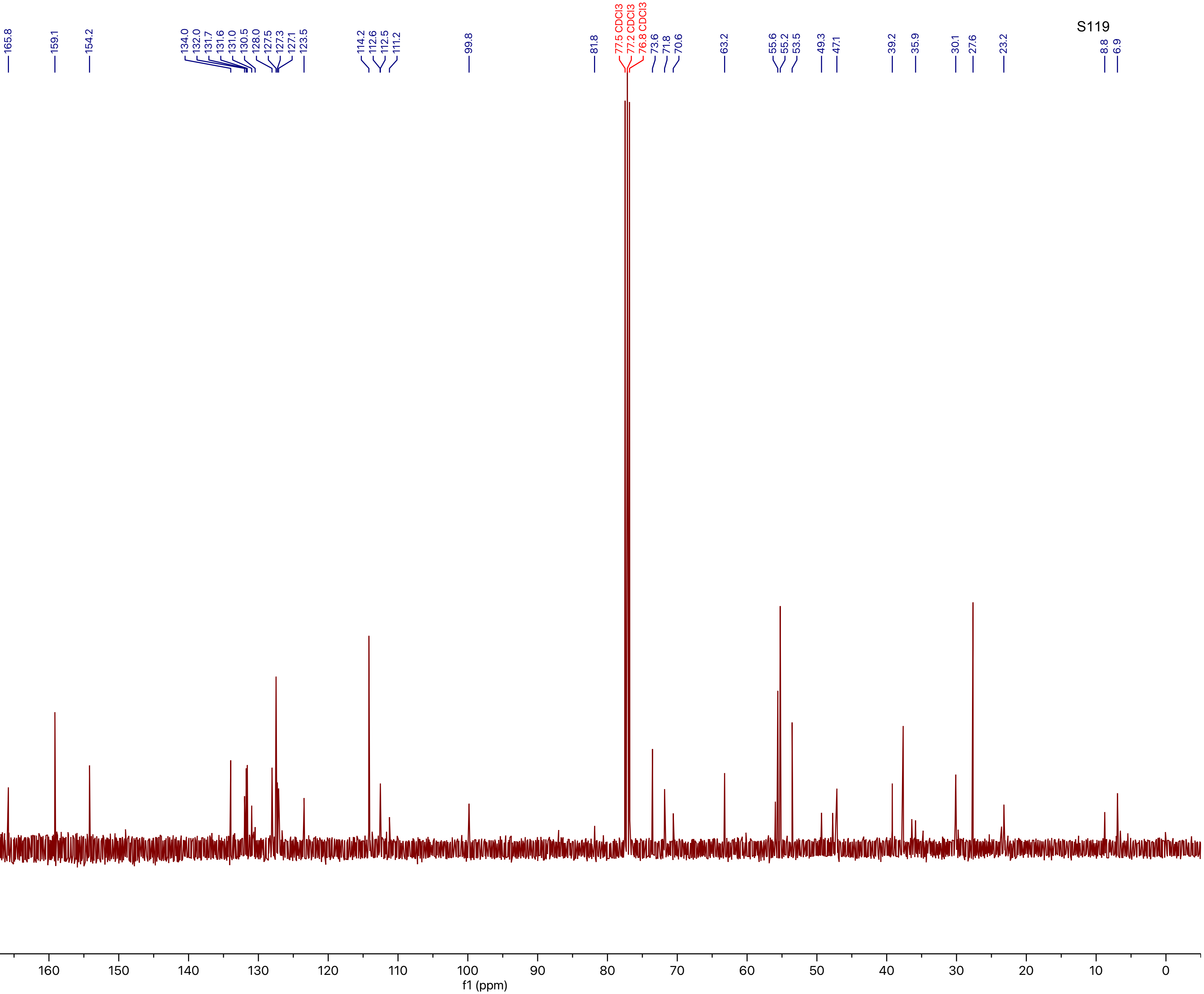

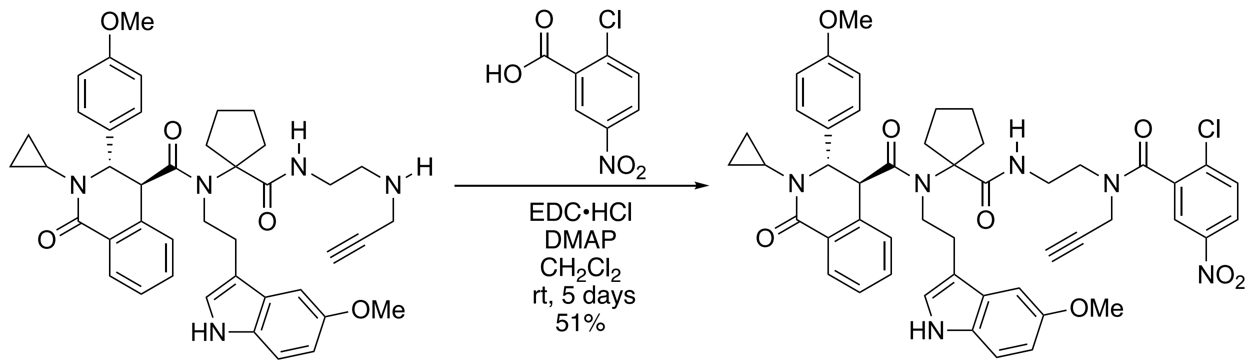

(±)-**4k2**
((±)-PJDU-191)

(±)-**4k**
((±)-PJDU-192)

(±)-(3S,4S*)-N-(1-((2-(2-chloro-5-nitro-N-(prop-2-yn-1-yl)benzamido)ethyl)carbamoyl)cyclopentyl)-2-cyclopropyl-N-(2-(5-methoxy-1H-indol-3-yl)ethyl)-3-(4-methoxyphenyl)-1-oxo-1,2,3,4-tetrahydroisoquinoline-4-carboxamide ((±)-**4k**) ((±)-PJDU-192).* A 10 mL flame-dried round-bottom flask equipped with a stir-bar, septum, and argon inlet needle was charged with 2-chloro-5-nitrobenzoic acid (40 mg, 0.20 mmol, 1.1 eq.) and CH_2Cl_2 (2 mL), then EDC•HCl (48 mg, 0.25 mmol, 1.4 eq.) and DMAP (3 mg, 0.02 mmol, 0.1 eq.) were added sequentially. Next a solution of propargyl amine (±)-**4k2** (126 mg, 0.18 mmol, 1 eq.; (±)-PJDU-191) in CH_2Cl_2 (2 mL) was added via cannula. The reaction mixture was stirred at room temperature for 5 days, wherein TLC analysis indicated a complete reaction. Next, the reaction mixture was concentrated and then transferred to a separatory funnel with EtOAc (60 mL). The organic layers were then washed sequentially with 1M aq. HCl solution (10 mL), H_2O (10 mL), sat. aq. $NaHCO_3$ (10 mL), and brine (10 mL) then dried ($MgSO_4$), filtered, and concentrated under reduced pressure. The crude residue was then purified by silica gel flash column chromatography (elution with 75% EtOAc/hexanes) to give FFSMP (±)-**4k** (81 mg, 51%; (±)-PJDU-192) as a white sticky solid.

R_f = 0.24 (75% EtOAc/hexanes)

1H NMR (500 MHz, $CDCl_3$, **peaks reported are for the major rotamer/diastereomer only) δ 8.59 (s, 1H), 8.17 – 8.10 (m, 3H), 7.55 (d, *J* = 8.9 Hz, 1H), 7.38 – 7.24 (m, 3H), 7.19 (s, 1H), 7.04 – 6.79 (m, 4H), 6.69 – 6.59 (m, 3H), 4.89 – 4.84 (s, 1H), 4.26 (s, 1H), 4.19 – 4.06 (m, 1H), 3.84 (s, 3H), 3.72 (s, 3H), 3.73 – 3.68 (m, 4H), 3.61 (s, 4H), 3.16 (s, 1H), 2.64 (t, *J* = 7.6 Hz, 1H), 2.50 (s, 3H), 2.32 (t, *J* = 2.4 Hz, 1H), 1.96 (s, 3H), 1.76 (s, 6H), 1.45 (s, 1H), 0.91 (s, 2H), 0.77 (s, 1H), 0.68 – 0.59 (m, 3H)s

^{13}C NMR (101 MHz, $CDCl_3$, **peaks reported are for the major rotamer/diastereomer only) δ 175.1, 174.7, 165.7, 159.1, 154.3, 137.4, 131.9, 131.5, 131.0, 128.1, 127.5, 127.3, 127.0, 126.9, 125.3, 123.6, 114.2, 114.1, 112.6, 112.5, 99.9, 77.4, 74.2, 73.5, 60.5, 55.6, 55.3, 49.1, 47.5, 35.3, 30.1, 28.1, 27.7, 23.6, 21.2, 7.0

HRMS (ESI+) *m/z* [M + Na] calcd for $C_{49}H_{49}ClN_6O_8Na$ 907.3193, found 907.3200

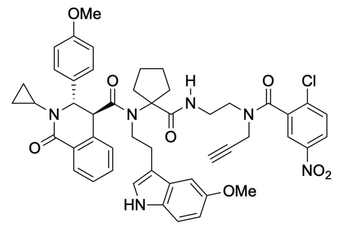

(±)-**4k**
((±)-PJDU-192)
^{1}H NMR (500 MHz, CDCl$_3$ with TMS)

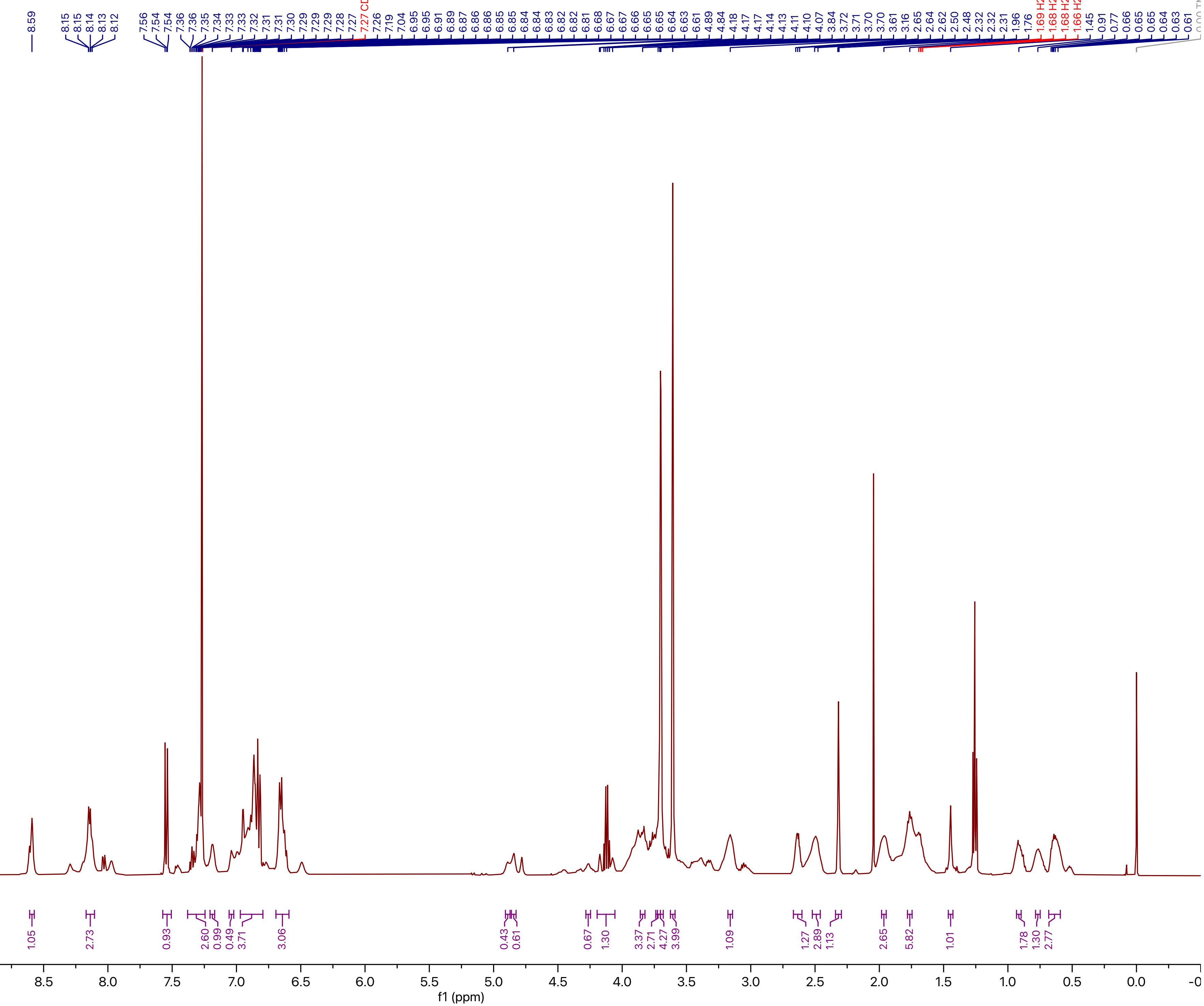

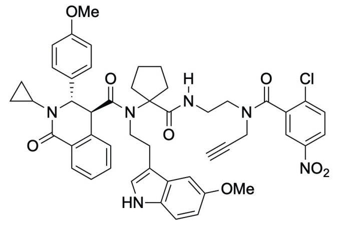

(±)-**4k**
((±)-PJDU-192)
^{13}C NMR (101 MHz, $CDCl_3$ with TMS)

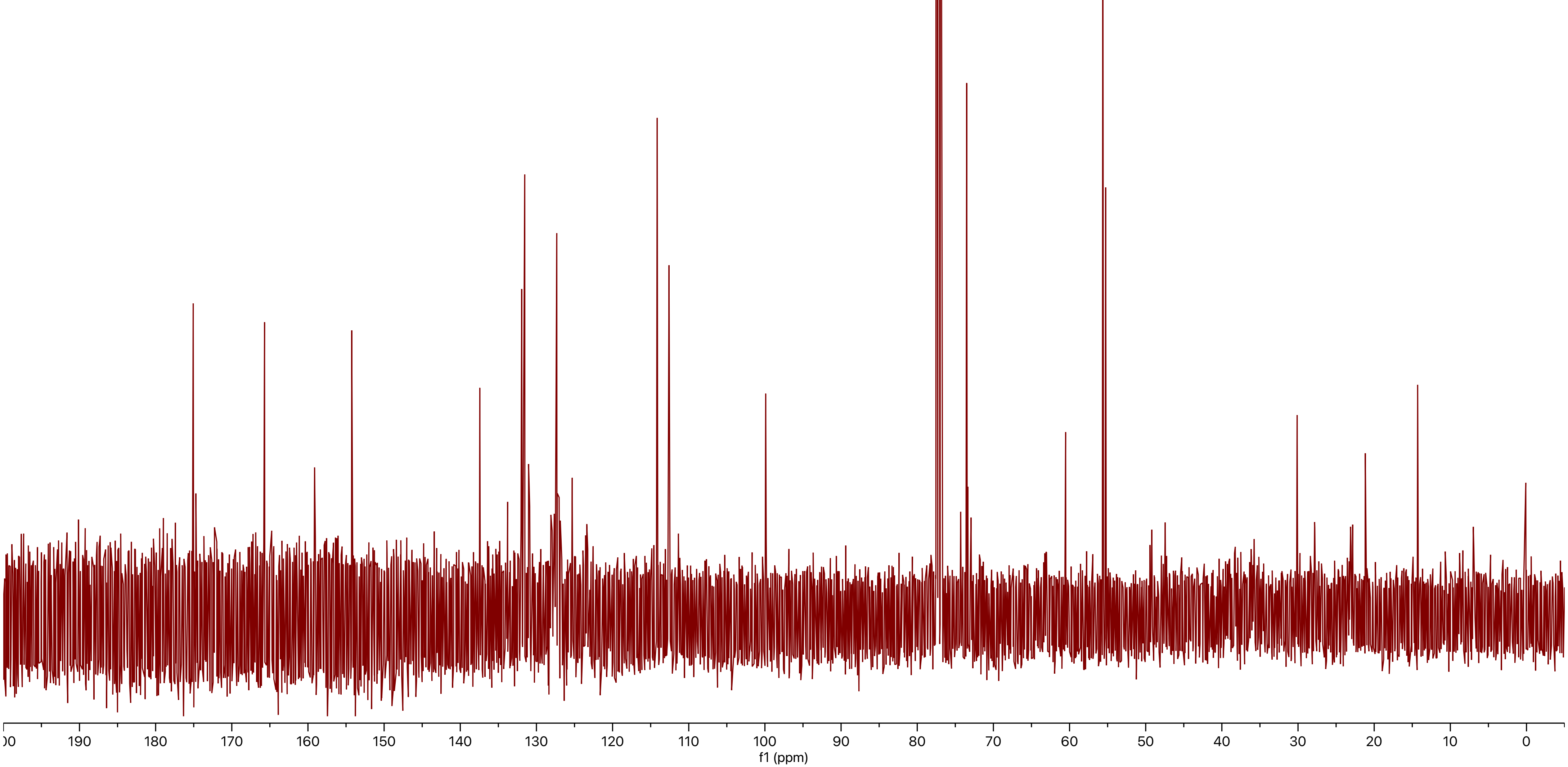

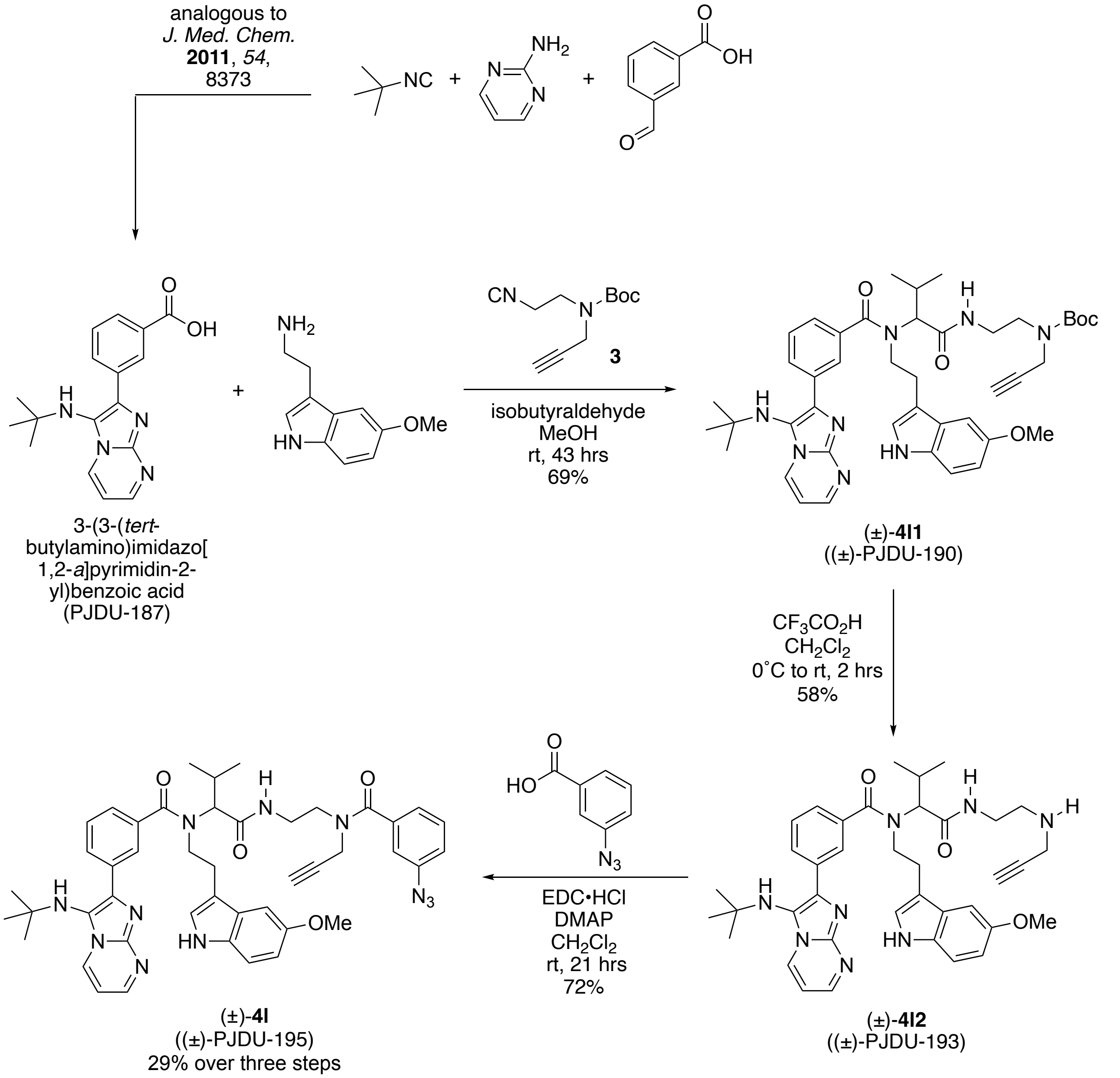

Scheme S11. *Synthesis of Groebke-Blackburn-Bienayme/U-4CR-based FFSMP (±)-**4l** ((±)-PJDU-195).*

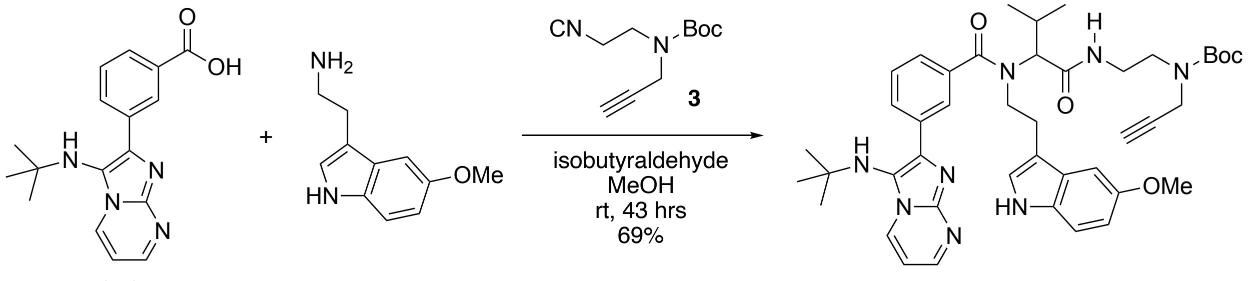

3-(3-(*tert*-butylamino)imidazo[1,2-*a*]pyrimidin-2-yl)benzoic acid (PJDU-187)

(±)-**4l1** ((±)-PJDU-190)

*(±)-tert-Butyl (2-(2-(3-(3-(tert-butylamino)imidazo[1,2-a]pyrimidin-2-yl)-N-(2-(5-methoxy-1H-indol-3-yl)ethyl)benzamido)-3-methylbutanamido)ethyl)(prop-2-yn-1-yl)carbamate ((±)-**4l1**) ((±)-PJDU-190).* First, carboxylic acid input 3-(3-(*tert*-butylamino)imidazo[1,2-*a*]pyrimidin-2-yl)benzoic acid (PJDU-187) was prepared by a Groebke-Blackburn-Bienayme reaction analogous to that described for the preparation of 3-(3-(*tert*-butylamino)imidazo[1,2-*a*]pyridin-2-yl)benzoic acid (CAS# 1350320-94-3) according to *J. Med. Chem.* **2011**, *54*, 8373, namely by using pyrimidin-2-amine as the 1˚ aromatic amine input instead of pyridin-2-amine. Next, a flame-dried, 10 mL round-bottom flask equipped with a stir-bar, septum, and argon inlet needle was sequentially charged with 5-methoxytryptamine (161 mg, 0.85 mmol, 1.0 eq.), isobutyraldehyde (60 uL, 0.89 mmol, 1.05 eq.), and MeOH (2.2 mL). This mixture was stirred for 30 minutes at room temperature to form the presumed imine. Next, carboxylic acid input PJDU-187 (263 mg, 0.85 mmol, 1.0 eq.) was added, followed by the addition of isocyanide **3** (185 mg, 0.89 mmol, 1.05 eq.). The reaction mixture was then stirred for 43 hours at room temperature, wherein TLC indicated a complete reaction. The reaction mixture was subsequently concentrated under reduced pressure, dissolved in EtOAc (50 mL), and then sequentially washed with sat. aq. $NaHCO_3$ (10 mL), H_2O (10 mL), and brine (10 mL). The separated organic layer was then dried ($MgSO_4$), filtered, concentrated, and purified by silica gel flash column chromatography (gradient elution with 50-75% EtOAc/hexanes) to give amide (±)-**4l1** (450 mg, 69%, (±)-PJDU-190) as a sticky yellow solid.

R_f = 0.38 (85% EtOAc/hexanes)

^{1}H NMR (400 MHz, $CDCl_3$, *peaks reported are for the major rotamer only) δ 9.36 (bs, 1H), 8.26-8.05 (m, 3H), 7.59-7.46 (m, 3H), 7.37-7.00 (m, 3H), 6.79-6.60 (m, 3H), 4.15-4.05 (m, 2H), 3.82-3.72 (m, 1H), 3.66 (s, 3H), 3.59-3.50 (m, 5H), 3.28- 3.17 (m, 2H), 2.91-2.72 (m, 2H), 2.30 (app s, 1H), 1.41 (s, 9H), 1.06 (app s, 3H), 1.00 (s, 9H), 0.73 (m, 3H)

^{13}C NMR (101 MHz, $CDCl_3$, *peaks reported are for the major rotamer only) δ 173.6, 171.6, 169.8, 155.0, 153.5, 142.2, 142.0, 138.1, 136.2, 135.7, 131.3, 129.2, 128.2, 127.3, 126.4, 125.2, 124.3, 123.7, 123.4, 117.1, 111.9, 111.4, 111.0, 99.9, 80.6, 79.5, 71.8, 68.6, 60.3, 56.2, 45.8, 43.5, 37.9, 36.9, 30.3, 28.2, 26.4, 22.8, 20.1, 19.1

HRMS (ESI+) *m*/*z* [M + H] calcd for $C_{44}H_{55}N_7O_5H$ 762.4337, found 762.4335

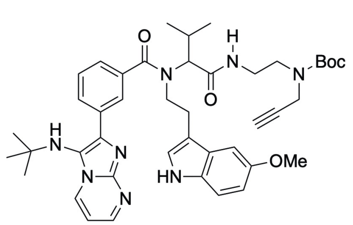

(±)-**4l1**
((±)-PJDU-190)
^{1}H NMR (400 MHz, $CDCl_3$ with TMS)

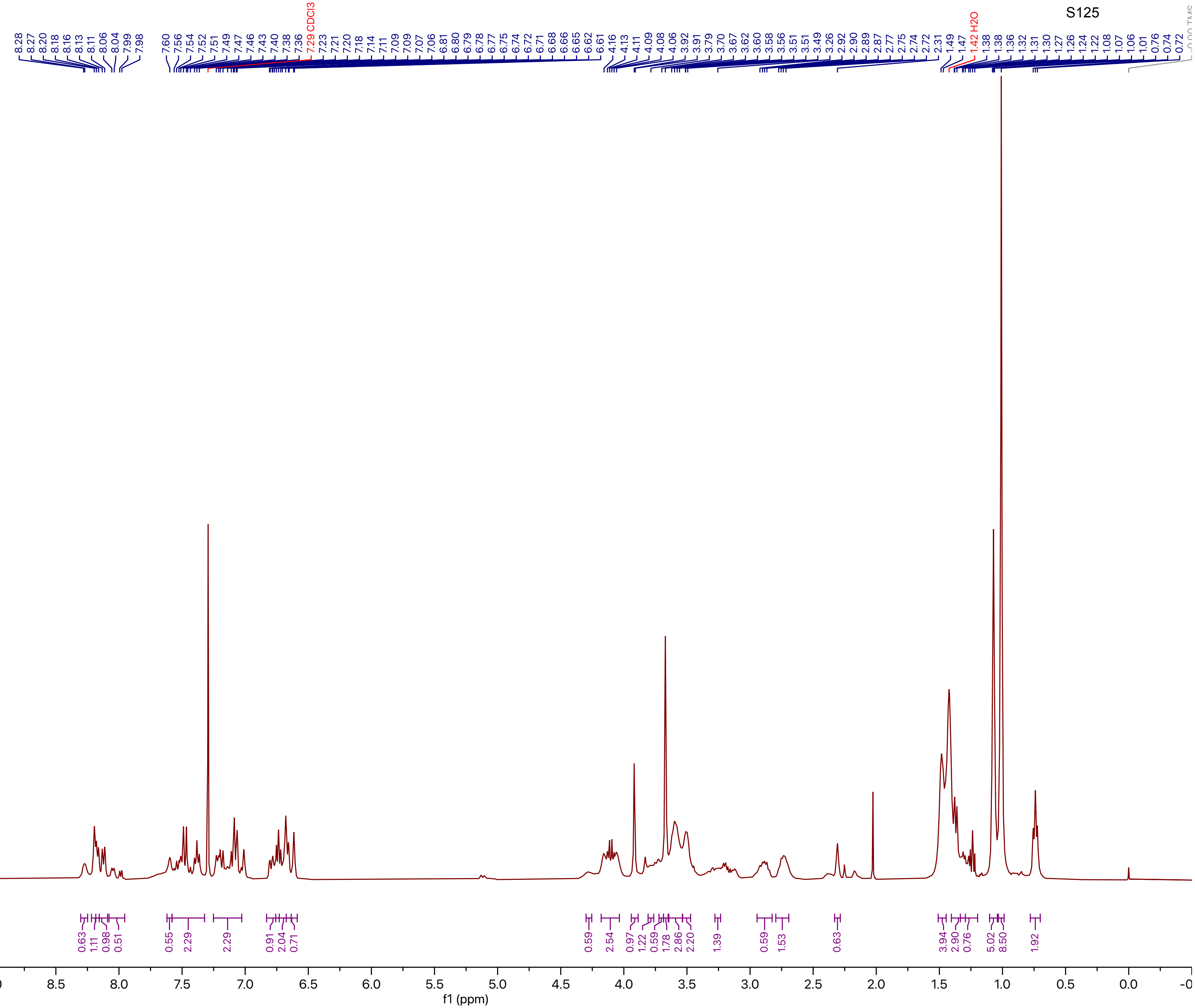

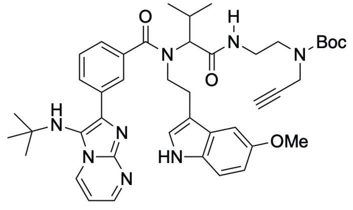

(±)-**4l1**
((±)-PJDU-190)
^{13}C NMR (101 MHz, $CDCl_3$ with TMS)

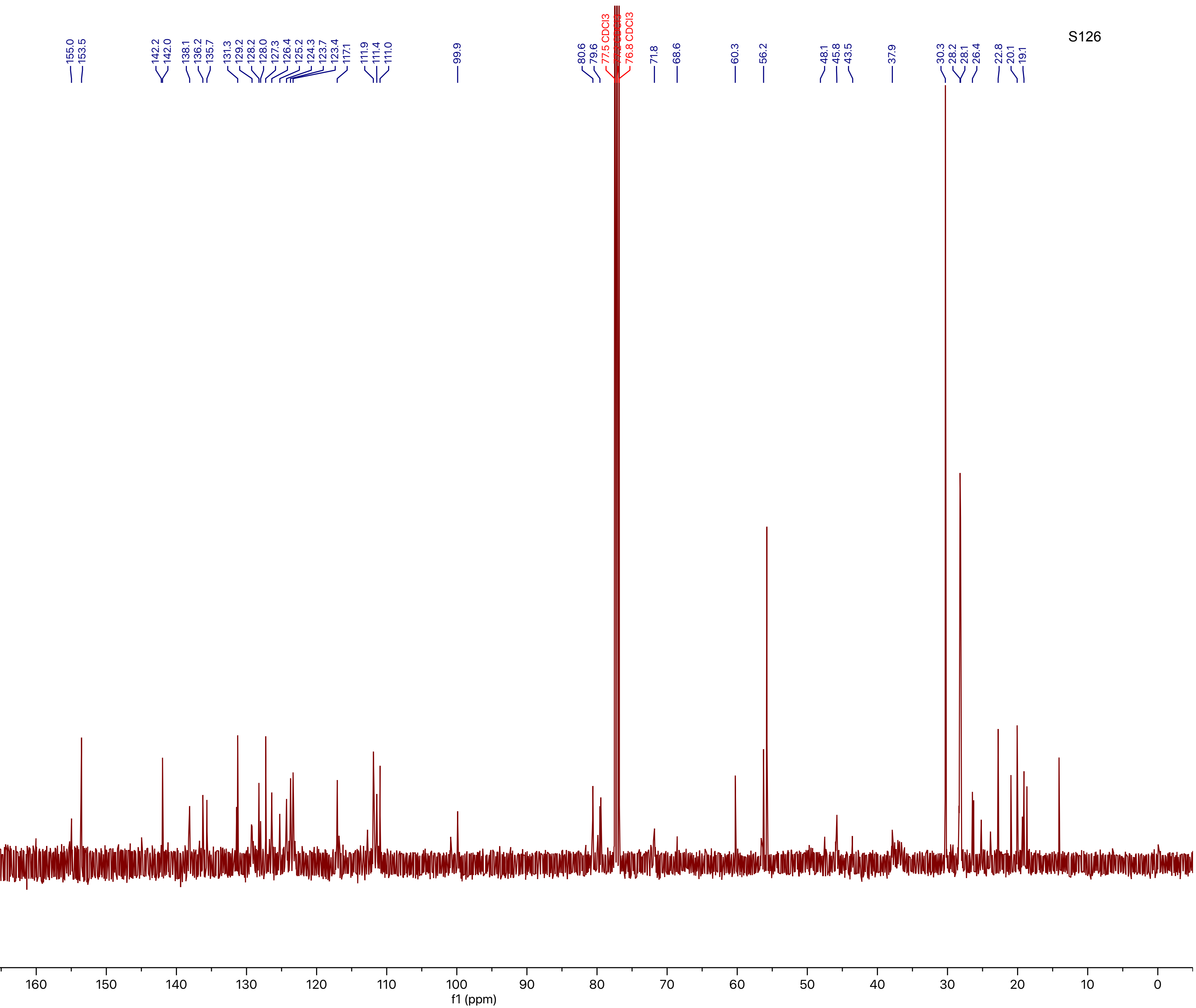

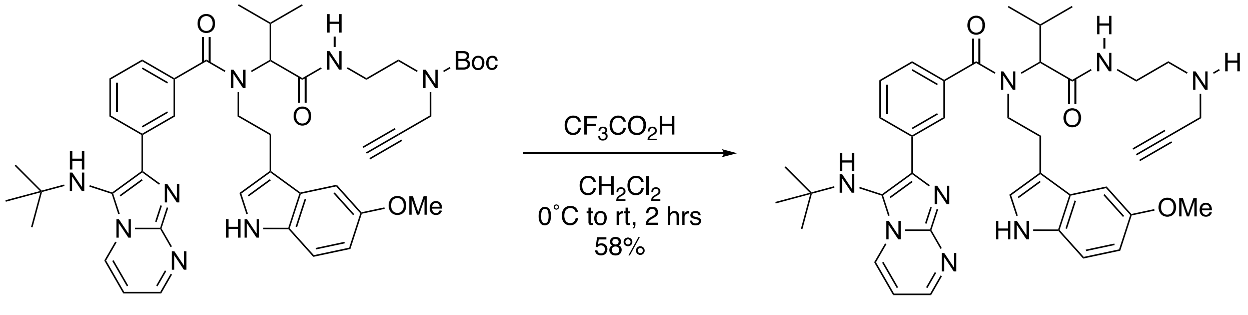

(±)-**4l1**
((±)-PJDU-190)

(±)-**4l2**
((±)-PJDU-193)

*(±)-3-(3-(tert-Butylamino)imidazo[1,2-a]pyrimidin-2-yl)-N-(2-(5-methoxy-1H-indol-3-yl)ethyl)-N-(3-methyl-1-oxo-1-((2-(prop-2-yn-1-ylamino)ethyl)amino)butan-2-yl)benzamide ((±)-**4l2**) ((±)-PJDU-193).* A 20 mL flame-dried, scintillation vial equipped with a stirbar, septum, and argon inlet needle was charged with Boc carbamate substrate (±)-**4l1** (450 mg, 0.59 mmol, 1.0 eq.; (±)-PJDU-190) and CH_2Cl_2 (6 mL) and cooled to 0˚C. Trifluoroacetic acid (0.54 mL, 7.1 mmol, 12.0 eq.) was then added dropwise over 2 minutes. After the addition, the ice bath was removed and the reaction was stirred at room temperature for 2 hours, wherein TLC indicated complete consumption of the starting Boc carbamate substrate. The reaction was then quenched by dropwise addition of sat. aq. $NaHCO_3$ (vigorous gas evolution) to the reaction mixture until gas evolution ceased (~10 mL), then 1M aq. NaOH (~5 mL) was added to raise the pH to 8-9 (checked with pH paper). This biphasic mixture was then transferred to a separatory funnel and diluted with CH_2Cl_2 (15 mL) and H_2O (5 mL), the layers were separated, and the aqueous layer was extracted with CH_2Cl_2 (3 x 15 mL). The combined organic layers were washed with brine (10 mL), dried ($MgSO_4$), gravity filtered, concentrated under reduced pressure, and purified by silica gel chromatography (elution with 100% EtOAc, then 5-10% MeOH/EtOAc) to give propargyl amine (±)-**4l2** (226 mg, 58%, (±)-PJDU-193) as a sticky yellow solid, which proved sufficiently pure to take on to the N-acylation arming step.

R_f = 0.34 (10% MeOH/EtOAc)

^{1}H NMR (400 MHz, $CDCl_3$, *peaks reported are for the major rotamer only) δ 9.19 (bs, 1H), 8.16-8.08 (m, 3H), 7.54-7.36 (m, 3H), 7.17-6.99 (m, 3H), 6.78-6.58 (m, 3H), 3.88-3.72 (m, 1H), 3.65 (s, 3H), 3.59-3.58 (m, 2H), 3.41-3.32 (m, 4H), 3.03-2.94 (m, 2H), 2.87-2.67 (m, 4H), 2.21 (t, *J* = 2.5 Hz, 1H), 1.03 (m, 3H), 0.98 (s, 9H), 0.75-0.71 (m, 3H)

^{13}C NMR (101 MHz, $CDCl_3$, *peaks reported are for the major rotamer only) δ 173.7, 171.5, 153.6, 142.1, 138.1, 136.4, 135.7, 131.3, 129.2, 128.3, 127.3, 126.4, 125.4, 124.4, 123.7, 123.4, 117.2, 112.0, 111.8, 111.5, 100.2, 82.0, 71.8, 68.5, 56.4, 55.9, 47.5, 43.7, 39.0, 37.7, 30.4, 26.5, 25.3, 20.1, 19.2

HRMS (ESI+) *m/z* [M + H] calcd for $C_{39}H_{47}N_7O_3H$ 662.3813, found 662.3823

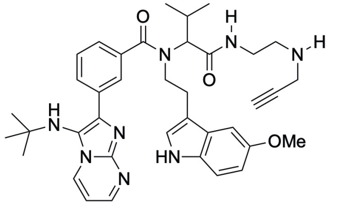

(±)-**4l2**
((±)-PJDU-193)
^{1}H NMR (400 MHz, $CDCl_3$ with TMS)

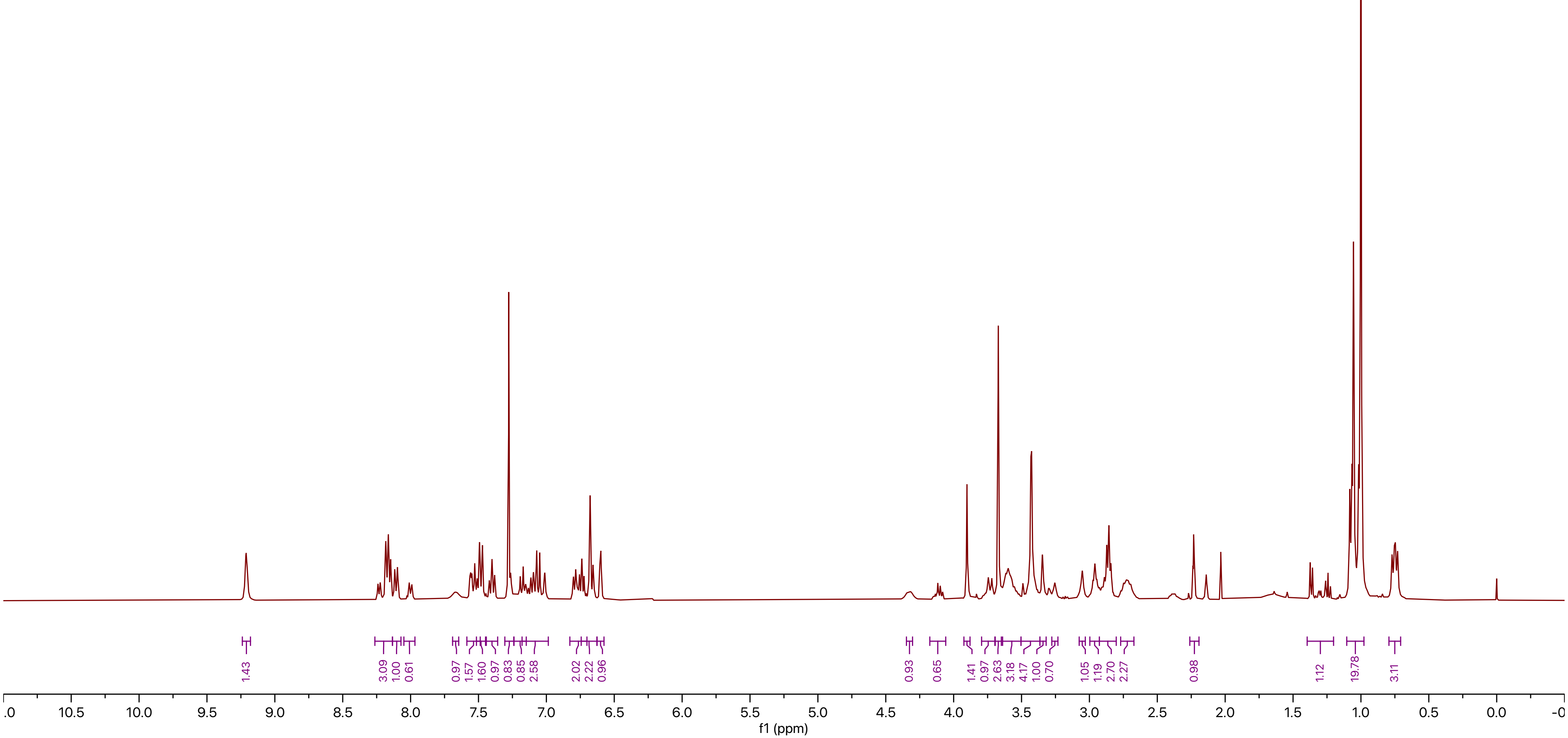

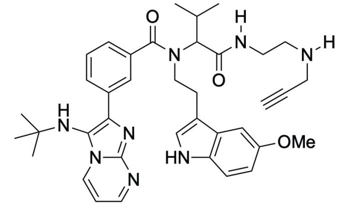

(±)-**4l2**
((±)-PJDU-193)
^{13}C NMR (101 MHz, $CDCl_3$ with TMS)

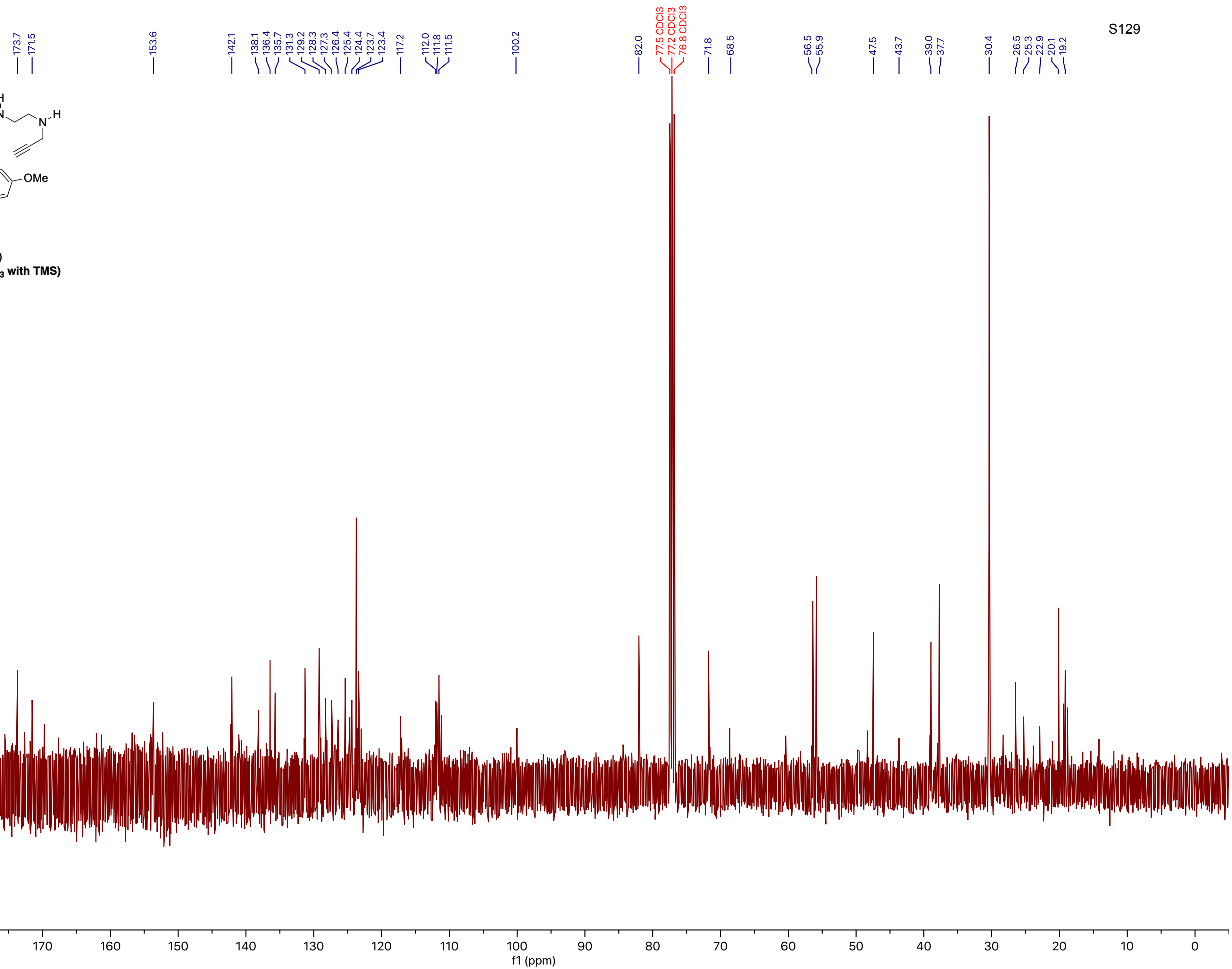

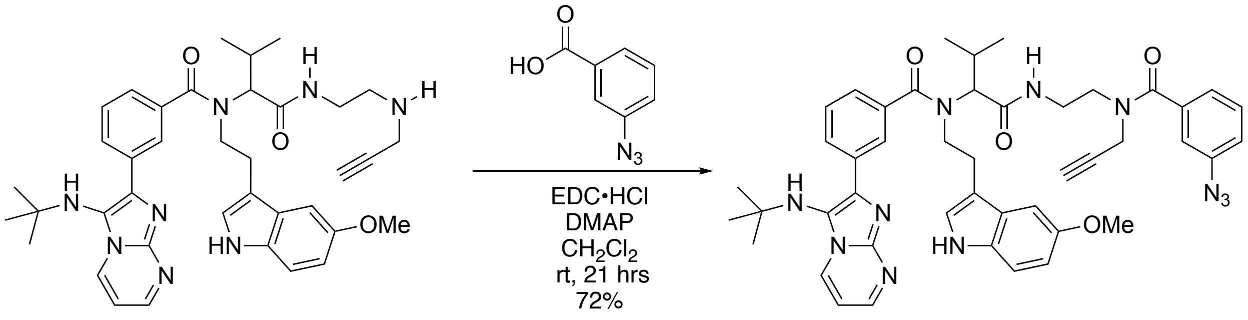

(±)-**4l2**
((±)-PJDU-193)

(±)-**4l**
((±)-PJDU-195)

(±)-3-Azido-*N*-(2-(2-(3-(3-(*tert*-butylamino)imidazo[1,2-*a*]pyrimidin-2-yl)-*N*-(2-(5-methoxy-1*H*-indol-3-yl)ethyl)benzamido)-3-methylbutanamido)ethyl)-*N*-(prop-2-yn-1-yl)benzamide ***((±)-4l) ((±)-PJDU-195).*** A 25 mL flame-dried round-bottom flask equipped with a stir-bar, septum, and argon inlet needle was charged with 3-azidobenzoic acid (44 mg, 0.27 mmol, 1.5 eq.) and CH_2Cl_2 (6 mL), then EDC•HCl (69 mg, 0.36 mmol, 2.0 eq.) and DMAP (3 mg, 0.02 mmol, 0.1 eq.) were added sequentially. Next, propargyl amine (±)-**4l2** (120 mg, 0.18 mmol, 1.0 eq.; (±)-PJDU-193) was added. The reaction mixture was stirred at room temperature for 21 hours, wherein TLC analysis indicated a complete reaction. Next, the reaction mixture was concentrated and then transferred to a separatory funnel with EtOAc (50 mL). The organic layers were then washed sequentially with sat. aq. $NaHCO_3$ (10 mL), H_2O (10 mL), and brine (10 mL) then dried ($MgSO_4$), filtered, and concentrated under reduced pressure. The crude residue was then purified by silica gel flash column chromatography (elution with 80% EtOAc/hexanes) to give FFSMP (±)-**4l** (105 mg, 72%; (±)-PJDU-195) as a yellow sticky solid.

R_f = 0.18 (85% EtOAc/hexanes)

1H NMR (400 MHz, $CDCl_3$, **peaks reported are for the major rotamer only) δ 8.54 (bs, 1H), 8.25-8.00 (m, 3H), 7.58-7.31 (m, 4H), 7.23-7.02 (m, 6H), 6.80-6.62 (m, 4H), 4.03 (m, 2H), 3.84-3.80 (m, 3H), 3.69 (s, 3H), 3.69-3.58 (m, 4H), 2.90-2.78 (m, 3H), 2.37 (app s, 1H), 1.08-1.01 (m, 3H), 0.95 (s, 9H), 0.78-0.76 (m, 3H)

^{13}C NMR (101 MHz, $CDCl_3$, **peaks reported are for the major rotamer only) δ 172.0, 168.8, 154.0, 153.9, 142.0, 140.2, 138.0, 136.3, 135.5, 134.4, 131.4, 131.3, 129.6, 128.4, 128.2, 127.5, 126.3, 124.8, 124.1, 123.7, 123.2, 122.7, 120.3, 120.2, 117.1, 113.2, 112.3, 111.9, 100.2, 78.5, 74.1, 68.6, 56.0, 44.8, 39.5, 37.1, 30.3, 26.6, 25.3, 20.3, 19.7

HRMS (ESI+) *m/z* [M + H] calcd for $C_{46}H_{50}N_{10}O_4H$ 807.4097, found 807.4084

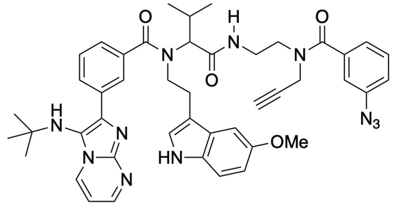

(±)-**4l**
((±)-PJDU-195)
^{1}H NMR (400 MHz, $CDCl_3$ with TMS)

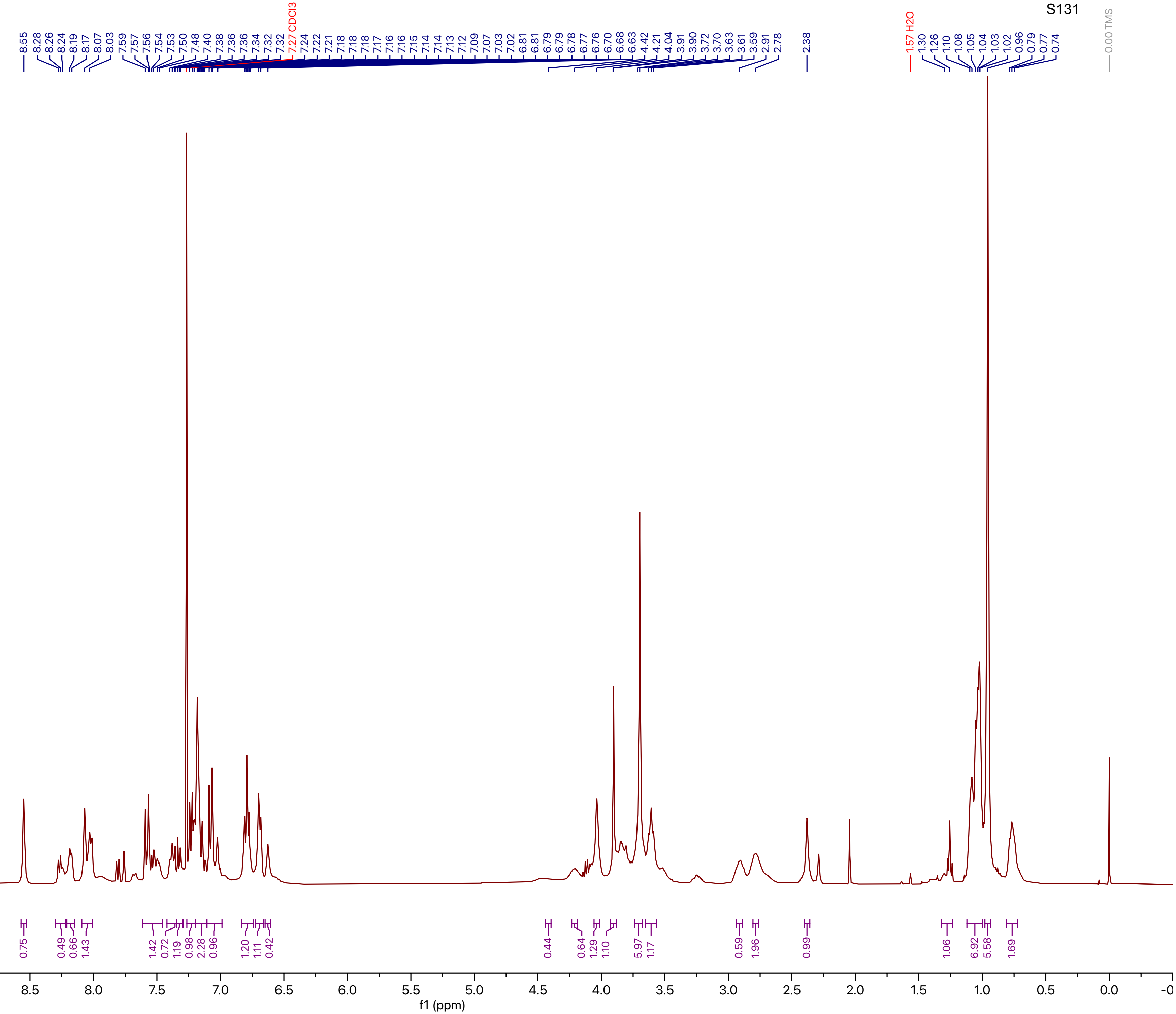

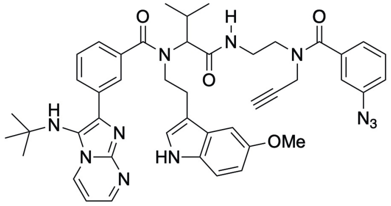

(±)-**4l**
((±)-PJDU-195)
^{13}C NMR (101 MHz, $CDCl_3$ with TMS)

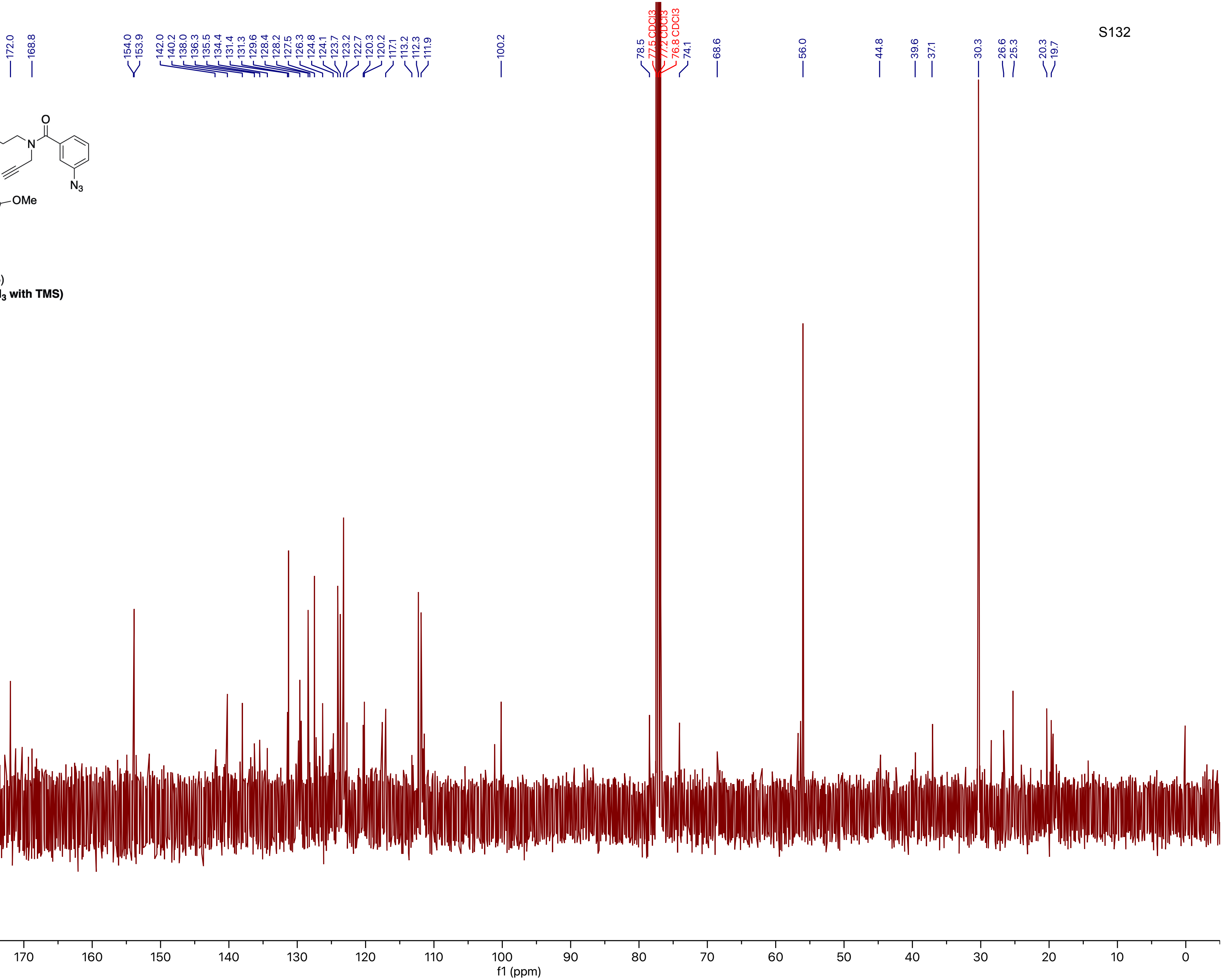

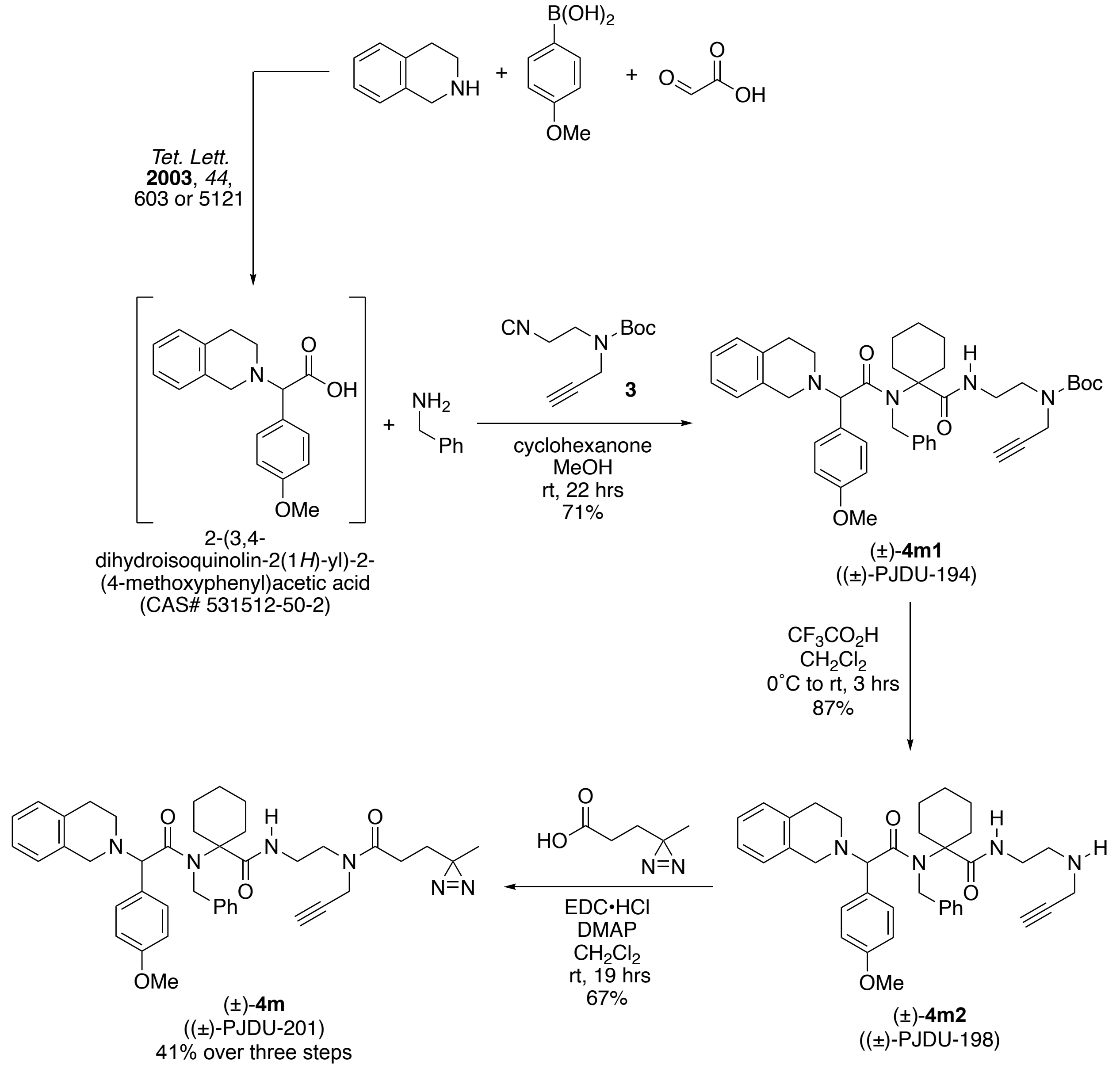

Scheme S12. *Synthesis of Petasis/U-4CR-based FFSMP (±)-**4m** ((±)-PJDU-201).*

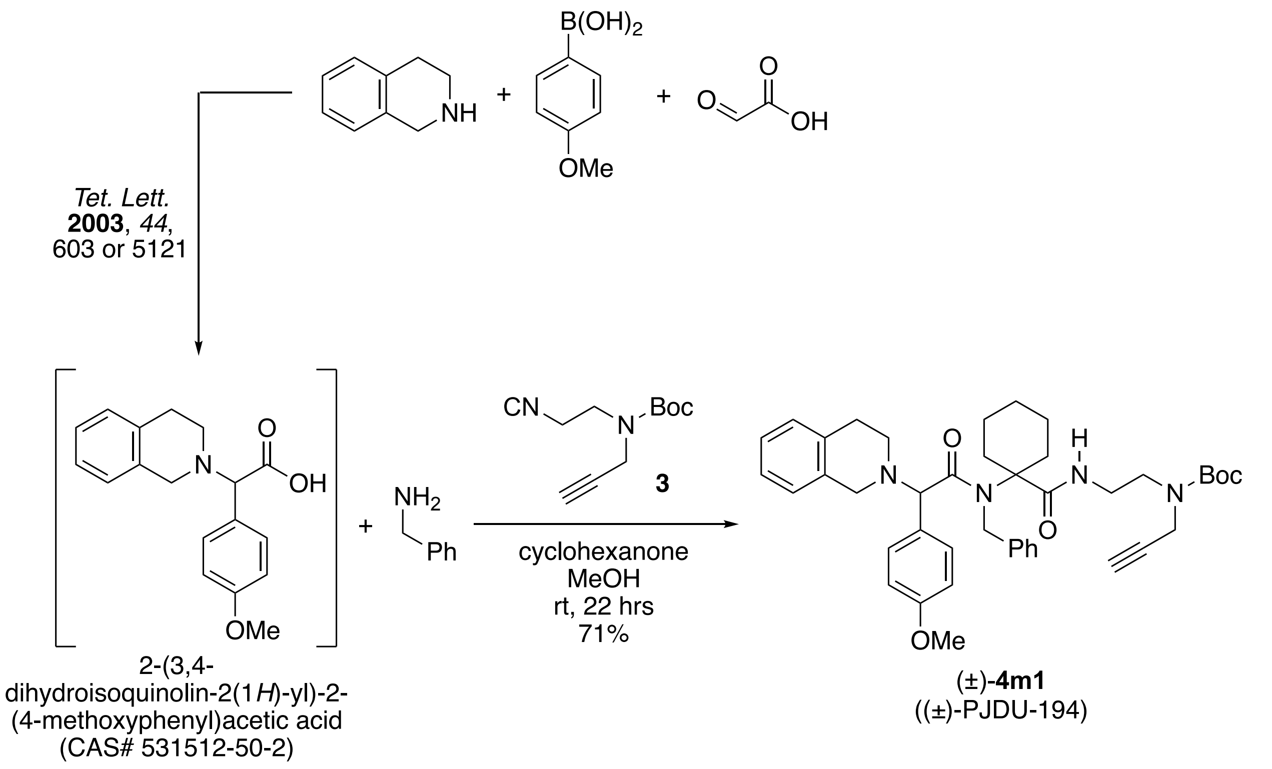

*(±)-tert-Butyl (2-(1-(N-benzyl-2-(3,4-dihydroisoquinolin-2(1H)-yl)-2-(4-methoxyphenyl)acetamido)cyclohexane-1-carboxamido)ethyl)(prop-2-yn-1-yl)carbamate ((±)-**4m1**) ((±)-PJDU-194).* Petasis-derived carboxylic acid 2-(3,4-dihydroisoquinolin-2(1*H*)-yl)-2-(4-methoxyphenyl)acetic acid (CAS# 531512-50-2) was prepared according to *Tetrahedron Letters* **2003**, *44*, 603 or 5121. Specifically, a flame-dried, 10 mL round-bottom flask equipped with a stir-bar, septum, and argon inlet needle was charged with glyoxylic acid monohydrate (88 mg, 0.95 mmol, 1.1 eq.) and dissolved in CH_2Cl_2 (4.7 mL,). Next, 1,2,3,4-tetrahydroisoquinoline (0.12 mL, 0.95 mmol, 1.1 eq.) was added all at once, followed by addition of 4-methoxyphenylboronic acid (144 mg, 0.95 mmol, 1.1 eq.) in a single portion. This mixture was stirred for 44 hours at room temperature, then concentrated to give the crude carboxylic acid 2-(3,4-dihydroisoquinolin-2(1*H*)-yl)-2-(4-methoxyphenyl)acetic acid (CAS# 531512-50-2), which was used directly in the subsequent Ugi four-component reaction. Specifically, a flame-dried 2-dram vial equipped with a stir-bar was charged sequentially with benzylamine (0.10 mL, 0.95 mmol, 1.1 eq.), cyclohexanone (0.10 mL, 0.95 mmol, 1.1 eq.), and MeOH (0.1 mL). This mixture was stirred for 1 hour at room temperature to form the presumed imine. Next, the imine solution was diluted with MeOH (0.5 mL) and transferred via cannula to the 10 mL round-bottom flask containing the crude carboxylic acid 2-(3,4-dihydroisoquinolin-2(1*H*)-yl)-2-(4-methoxyphenyl)acetic acid (CAS# 531512-50-2). The vial and cannula were then rinsed with MeOH (0.5 mL). Next, isocyanide **3** (180 mg, 0.86 mmol, 1 eq.) was dissolved in MeOH (1 mL) and transferred to the reaction flask via cannula (i.e., total of 2.1 mL of MeOH). The reaction mixture was then stirred at room temperature for 22 hours, wherein TLC analysis indicated a complete reaction. Next, the reaction mixture was concentrated under reduced pressured, and then the crude residue was dissolved in EtOAc (75 mL) and transferred to a separatory funnel. The organic layer was washed sequentially with sat. aq. $NaHCO_3$ (15 mL), H_2O (15 mL), and brine (15 mL), dried ($MgSO_4$), filtered, concentrated under reduced pressure, and purified by silica gel flash column chromatography (gradient elution with 20-40% EtOAc/hexanes) to provide amide (±)-**4m1** (426 mg, 71%, (±)-PJDU-194) as a pale yellow oil.

R_f = 0.48 (50% EtOAc/hexanes)

^{1}H NMR (400 MHz, $CDCl_3$, *peaks reported are for the major rotamer only) δ 7.42-7.35 (m, 4H), 7.30-7.24 (m, 3H), 7.04-7.00 (m, 3H), 6.90-6.86 (m, 3H), 6.57 (bs, 1H), 4.55 (s, 2H), 4.25 (s, 1H), 4.11-4.07 (m, 2H), 3.78 (s, 3H), 3.65 (m, 2H), 3.51 (m, 4H), 2.80-2.75 (m, 4H), 2.44 (m, 2H), 2.17 (app s, 1H), 1.55 (m, 6H), 1.47 (m, 11H)

^{13}C NMR (101 MHz, $CDCl_3$, *peaks reported are for the major rotamer only) δ 174.0, 173.2, 159.4, 155.6, 138.8, 135.0, 134.5, 130.5, 128.7, 128.5, 127.3, 127.1, 126.4, 125.8, 125.7, 125.2, 113.8, 80.3, 79.8, 71.4, 69.7, 66.0, 60.2, 55.1, 53.1, 47.4, 46.7, 45.8, 39.0, 37.0, 33.1, 31.2, 29.2, 28.2, 25.3, 22.8

HRMS (ESI+) *m*/*z* [M + H] calcd for $C_{42}H_{52}N_4O_5H$ 693.4010, found 693.4020

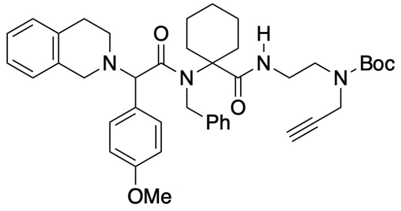

(±)-**4m1**
((±)-PJDU-194)
^{1}H NMR (400 MHz, $CDCl_3$ with TMS)

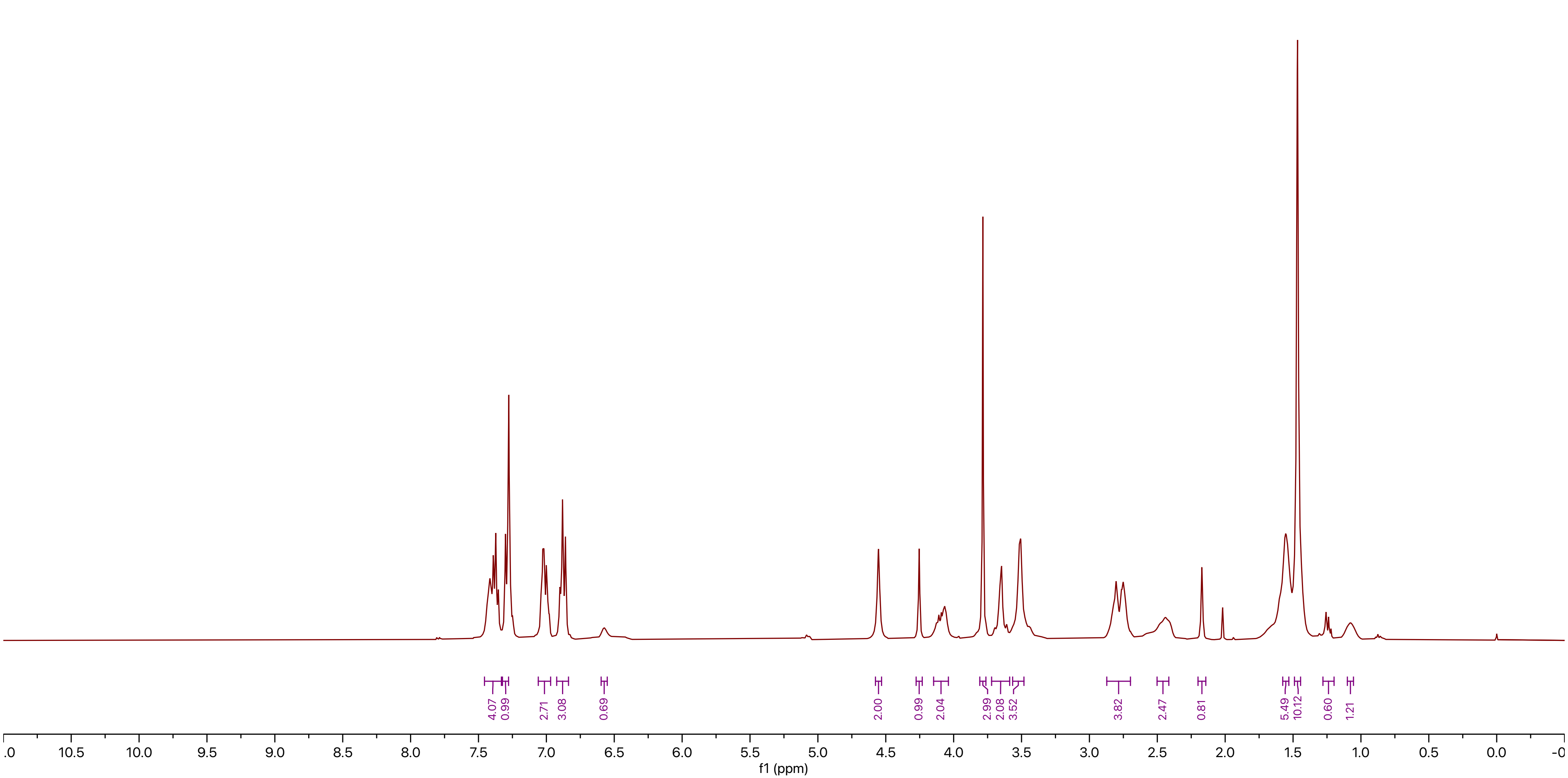

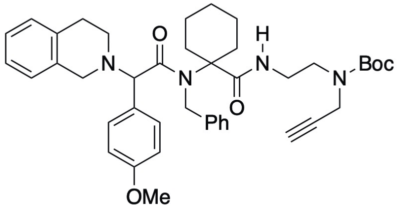

(±)-**4m1**
((±)-PJDU-194)
^{13}C NMR (101 MHz, $CDCl_3$ with TMS)

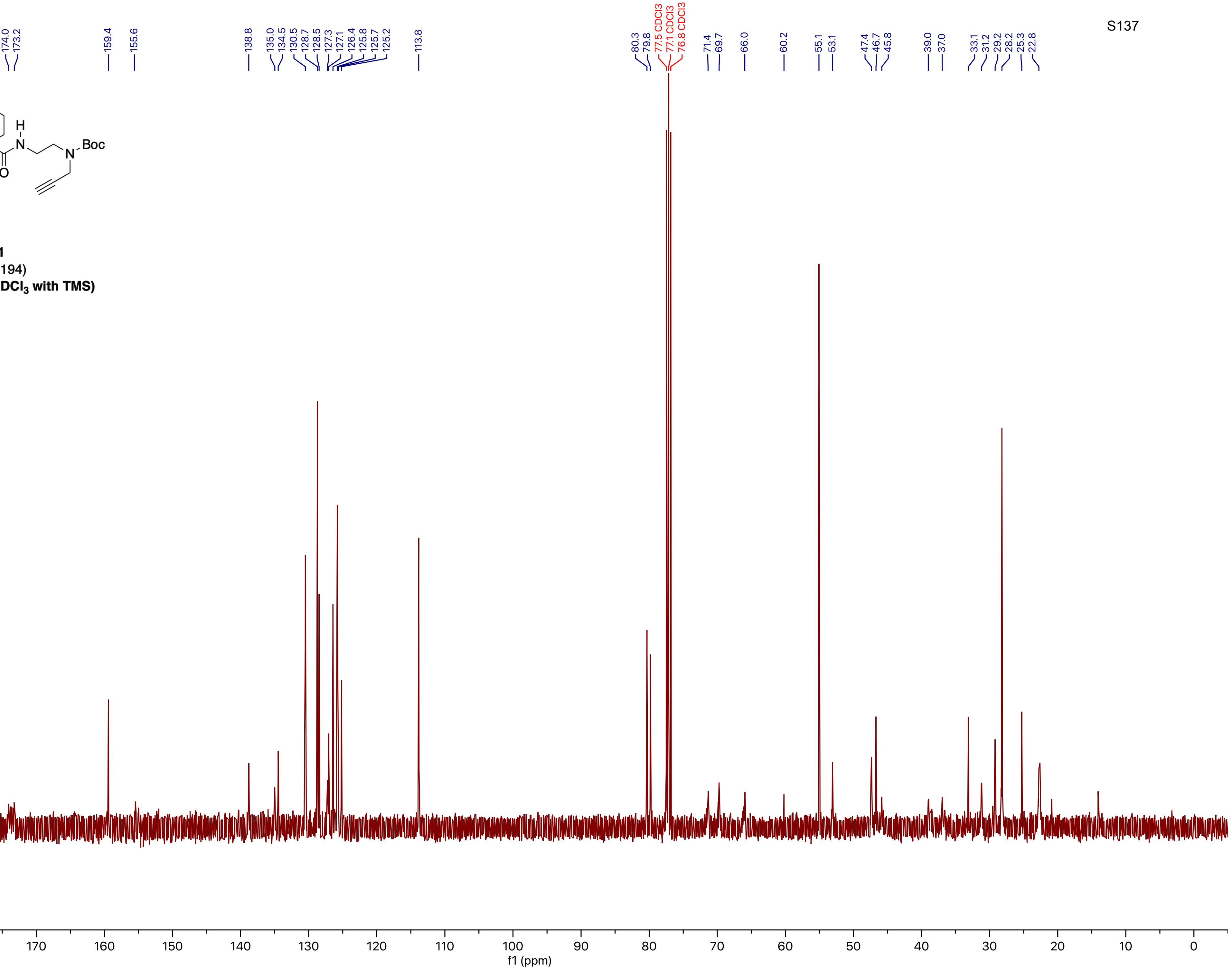

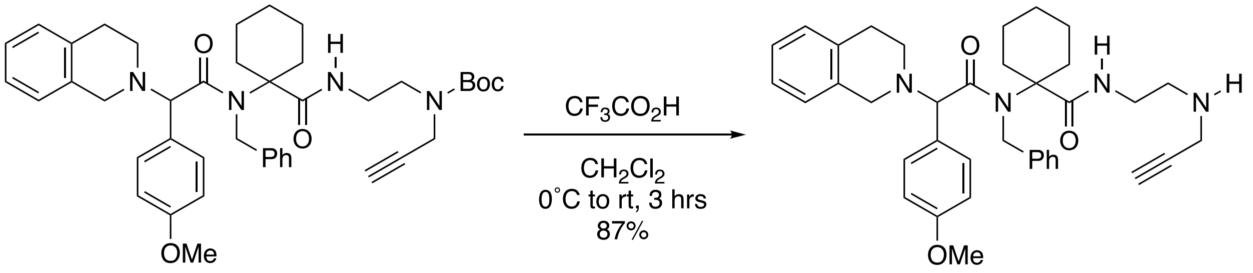

(±)-**4m1**
((±)-PJDU-194)

(±)-**4m2**
((±)-PJDU-198)

*(±)-1-(N-Benzyl-2-(3,4-dihydroisoquinolin-2(1H)-yl)-2-(4-methoxyphenyl)acetamido)-N-(2-(prop-2-yn-1-ylamino)ethyl)cyclohexane-1-carboxamide ((±)-**4m2**) ((±)-PJDU-198).* A 25 mL flame-dried, round-bottom flask equipped with a stirbar, septum, and argon inlet needle was charged with Boc carbamate substrate (±)-**4m1** (426 mg, 0.62 mmol, 1.0 eq.; (±)-PJDU-194) and CH_2Cl_2 (6 mL) and cooled to 0˚C. Trifluoroacetic acid (0.56 mL, 7.4 mmol, 12.0 eq.) was then added dropwise over 2 minutes. After the addition, the ice bath was removed and the reaction was stirred at room temperature for 3 hours, wherein TLC indicated complete consumption of the starting Boc carbamate substrate. The reaction was then quenched by dropwise addition of sat. aq. $NaHCO_3$ (vigorous gas evolution) to the reaction mixture until gas evolution ceased (~10 mL), then 1M aq. NaOH (~5 mL) was added to raise the pH to 8-9 (checked with pH paper). This biphasic mixture was then transferred to a separatory funnel and diluted with CH_2Cl_2 (15 mL) and H_2O (5 mL), the layers were separated, and the aqueous layer was extracted with CH_2Cl_2 (3 x 15 mL). The combined organic layers were washed with brine (10 mL), dried ($MgSO_4$), gravity filtered, concentrated under reduced pressure to give propargyl amine (±)-**4m2** (317 mg, 87%, (±)-PJDU-198) as a yellow oil, which proved sufficiently pure to take on to the N-acylation arming step.

R_f = 0.55 (10% MeOH/ CH_2Cl_2)

^{1}H NMR (400 MHz, $CDCl_3$, *peaks reported are for the major rotamer only) δ 7.41 (dt, *J* = 14.9, 7.7 Hz, 4H), 7.33 – 7.20 (m, 3H), 7.03 (pt, *J* = 5.9, 3.0 Hz, 3H), 6.90 (s, 1H), 6.93 – 6.84 (m, 2H), 6.58 (t, *J* = 5.6 Hz, 1H), 4.55 (d, *J* = 4.0 Hz, 2H), 4.33 (s, 1H), 3.80 (s, 3H), 3.72 (d, *J* = 14.7 Hz, 1H), 3.62 (d, *J* = 14.8 Hz, 1H), 3.42 (q, *J* = 5.8 Hz, 2H), 3.34 (d, *J* = 2.5 Hz, 2H), 2.89 (dt, *J* = 16.7, 5.9 Hz, 3H), 2.74 (d, *J* = 11.0 Hz, 3H), 2.41 (dd, *J* = 19.1, 10.8 Hz, 2H), 2.14 (t, *J* = 2.4 Hz, 1H), 1.71 (d, *J* = 12.8 Hz, 1H), 1.54 (d, *J* = 6.7 Hz, 3H), 1.49 – 1.36 (m, 1H), 1.28 – 1.21 (m, 1H), 1.13 – 1.03 (m, 1H).

^{13}C NMR (101 MHz, $CDCl_3$, *peaks reported are for the major rotamer only) δ 173.9, 173.5, 159.5, 138.9, 135.0, 134.5, 130.7, 130.4, 128.9, 128.6, 127.2, 127.0, 126.5, 125.9, 125.3, 113.9, 81.8, 71.6, 69.6, 66.3, 55.2, 53.1, 47.4, 46.9, 46.8, 39.2, 37.6, 33.3, 31.3, 29.4, 25.4, 22.8, 22.6

HRMS (ESI+) *m/z* [M + H] calcd for $C_{37}H_{44}N_4O_3H$ 593.3486, found 593.3496

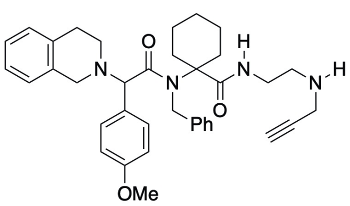

(±)-**4m2**
((±)-PJDU-198)
^{1}H NMR (400 MHz, $CDCl_3$ with TMS)

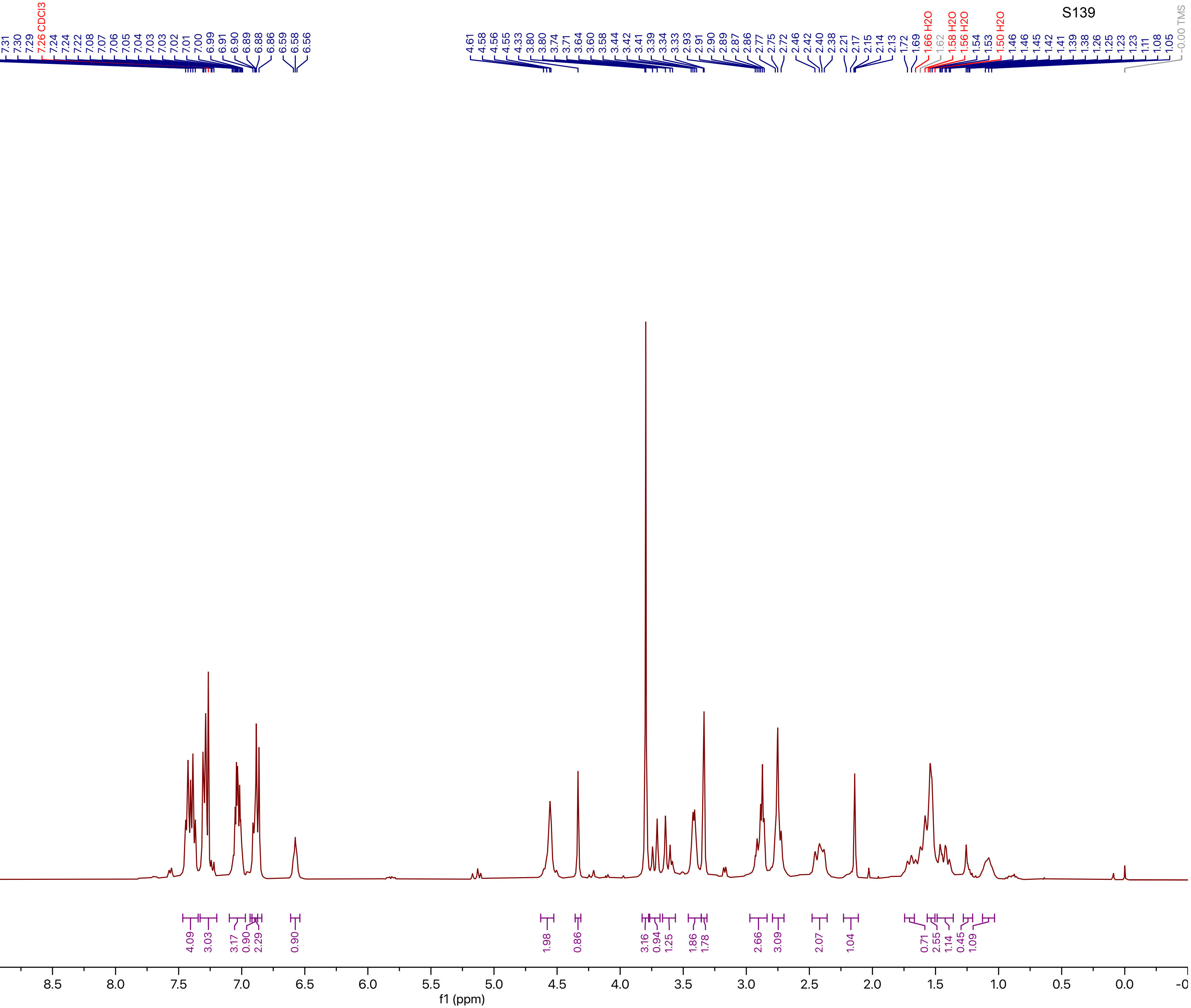

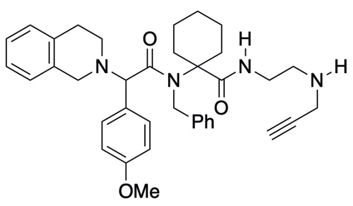

(±)-**4m2**
((±)-PJDU-198)
^{13}C NMR (101 MHz, $CDCl_3$ with TMS)

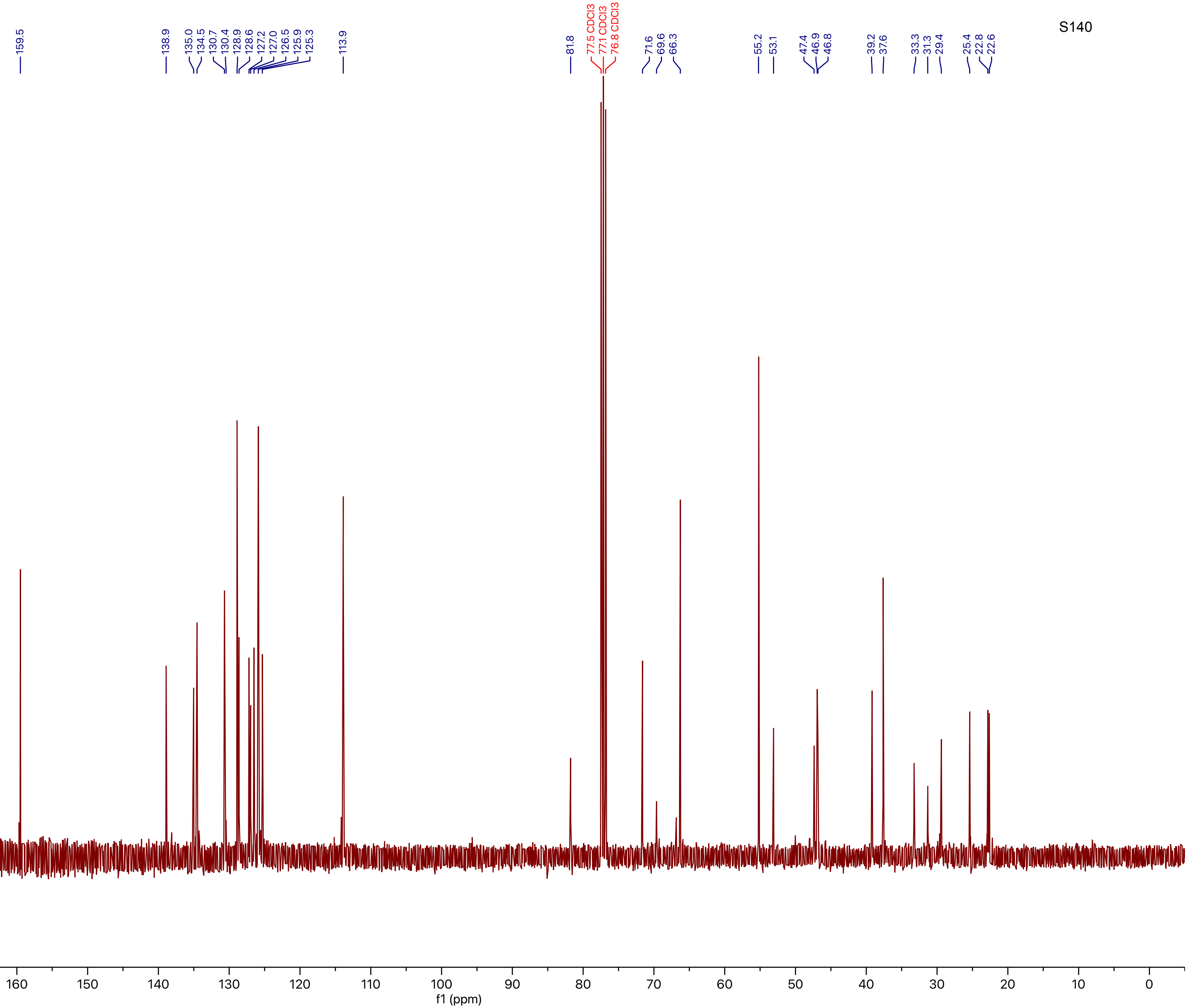

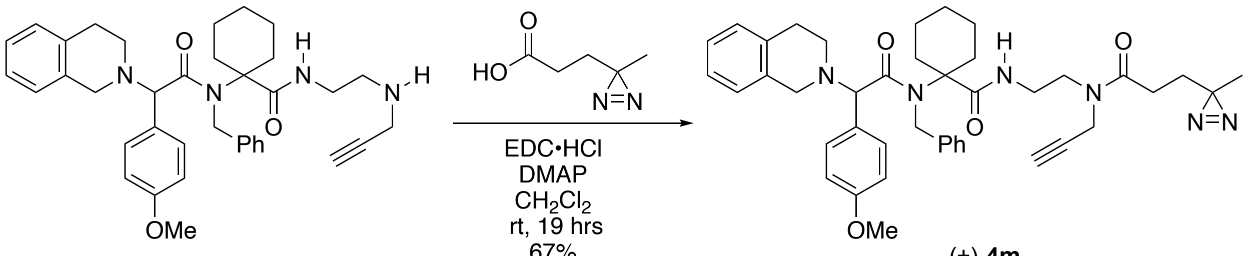

(±)-**4m2**
((±)-PJDU-198)

(±)-**4m**
((±)-PJDU-201)

*(±)-1-(N-Benzyl-2-(3,4-dihydroisoquinolin-2(1H)-yl)-2-(4-methoxyphenyl)acetamido)-N-(2-(3-(3-methyl-3H-diazirin-3-yl)-N-(prop-2-yn-1-yl)propanamido)ethyl)cyclohexane-1-carboxamide ((±)-**4m**) ((±)-PJDU-201).* A 25 mL flame-dried round-bottom flask equipped with a stir-bar, septum, and argon inlet needle was charged with 3-(3-methyl-3*H*-diazirin-3-yl)propanoic acid (55 mg, 0.43 mmol, 1.5 eq.) and CH_2Cl_2 (3 mL), then EDC•HCl (110 mg, 0.57 mmol, 2.0 eq.) and DMAP (4 mg, 0.03 mmol, 0.1 eq.) were added sequentially. Next, a solution of propargyl amine (±)-**4m2** (170 mg, 0.29 mmol, 1.0 eq.; (±)-PJDU-198) in CH_2Cl_2 (4 mL) was added via cannulation. The reaction mixture was stirred at room temperature for 19 hours, wherein TLC analysis indicated a complete reaction. Next, the reaction mixture was concentrated and then transferred to a separatory funnel with EtOAc (50 mL). The organic layers were then washed sequentially with sat. aq. $NaHCO_3$ (10 mL), H_2O (10 mL), and brine (10 mL) then dried ($MgSO_4$), filtered, and concentrated under reduced pressure. The crude residue was then purified by silica gel flash column chromatography (elution with 50-60% EtOAc/hexanes) to give FFSMP (±)-**4m** (135 mg, 67%; (±)-PJDU-201) as a colorless oil.

R_f = 0.45 (75% EtOAc/hexanes)

1H NMR (400 MHz, $CDCl_3$, **peaks reported are for the major rotamer only) δ 7.46-7.36 (m, 4H), 7.30-7.25 (m, 3H), 7.05-7.01 (m, 3H), 6.90-6.87 (m, 3H), 6.51 (bs, 1H), 4.53 (s, 2H), 4.24-4.23 (m, 2H), 4.11-4.08 (m, 2H), 3.81 (s, 3H), 3.64-3.46 (m, 6H), 2.78-2.77 (m, 4H), 2.39-2.30 (m, 2H), 2.28-2.25 (m, 3H), 1.78-1.72 (m, 2H), 1.59-1.45 (m, 8H), 1.02 (s, 3H)

^{13}C NMR (101 MHz, $CDCl_3$, **peaks reported are for the major rotamer only) δ 174.4, 173.3, 172.6, 159.5, 139.0, 135.2, 134.7, 130.6, 128.9, 128.7, 127.5, 127.2, 126.6, 126.0, 125.8, 125.4, 114.0, 79.6, 78.7, 72.9, 69.8, 66.0, 55.3, 53.2, 47.5, 46.7, 45.6, 38.7, 37.9, 35.2, 33.2, 31.0, 29.6, 27.5, 25.5, 22.9, 22.6, 20.0

HRMS (ESI+) *m/z* [M + H] calcd for $C_{42}H_{50}N_6O_4H$ 703.3966, found 703.3964

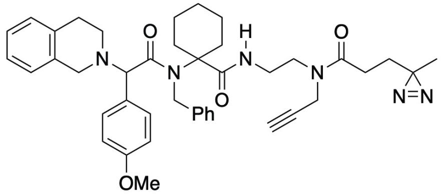

(±)-**4m**
((±)-PJDU-201)
^{1}H NMR (400 MHz, $CDCl_3$ with TMS)

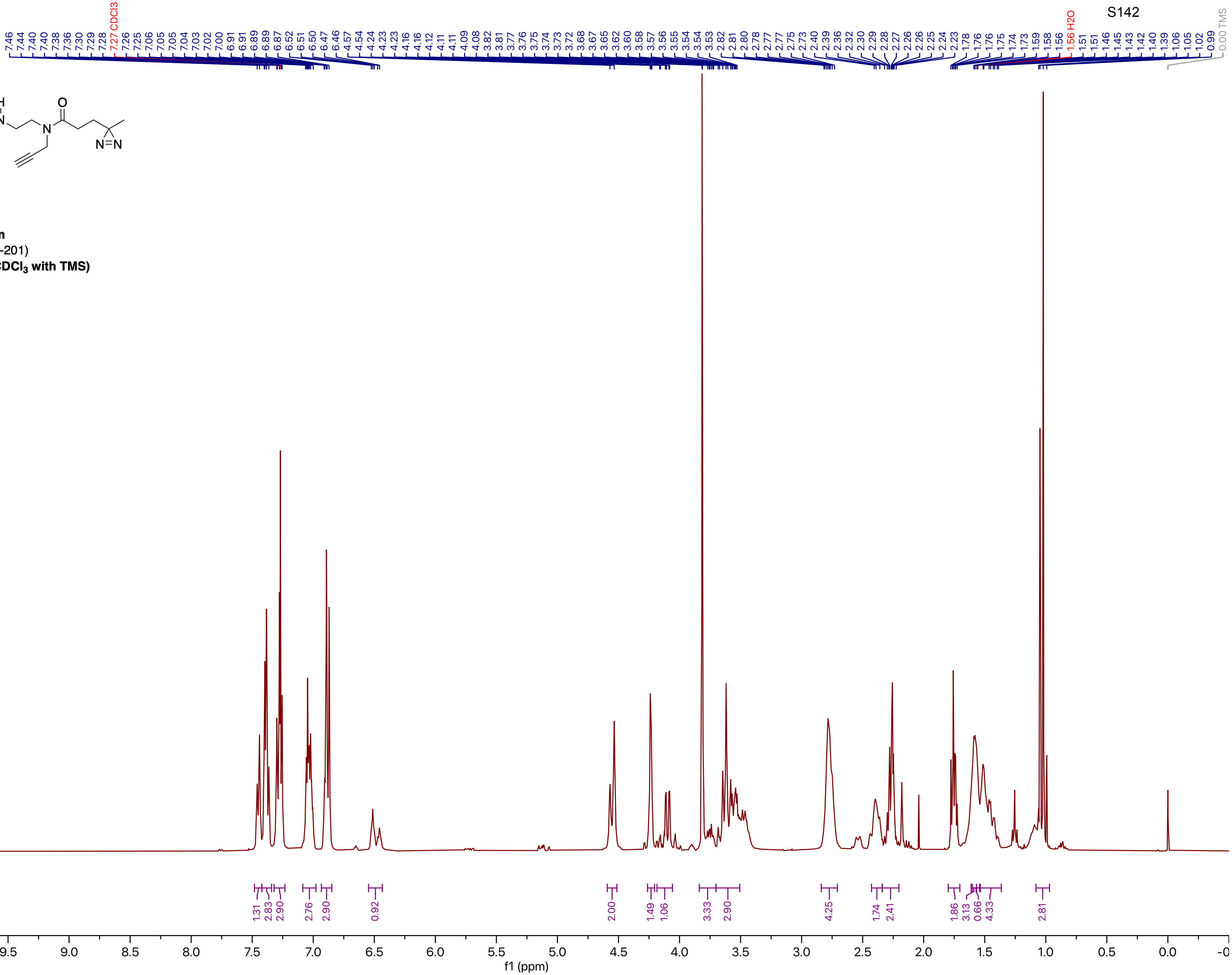

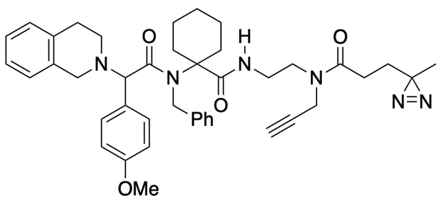

(±)-**4m**
((±)-PJDU-201)
^{13}C NMR (101 MHz, $CDCl_3$ with TMS)

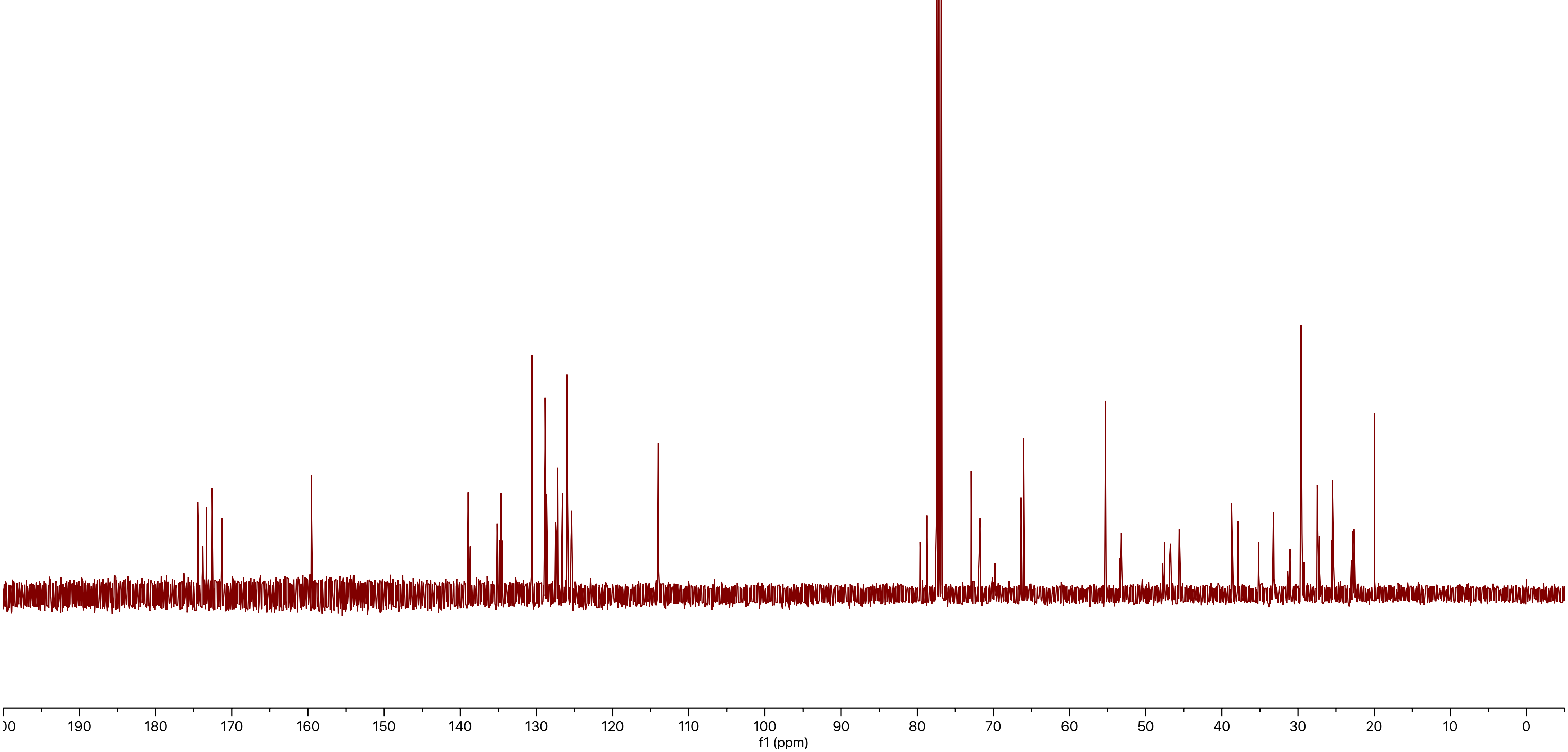

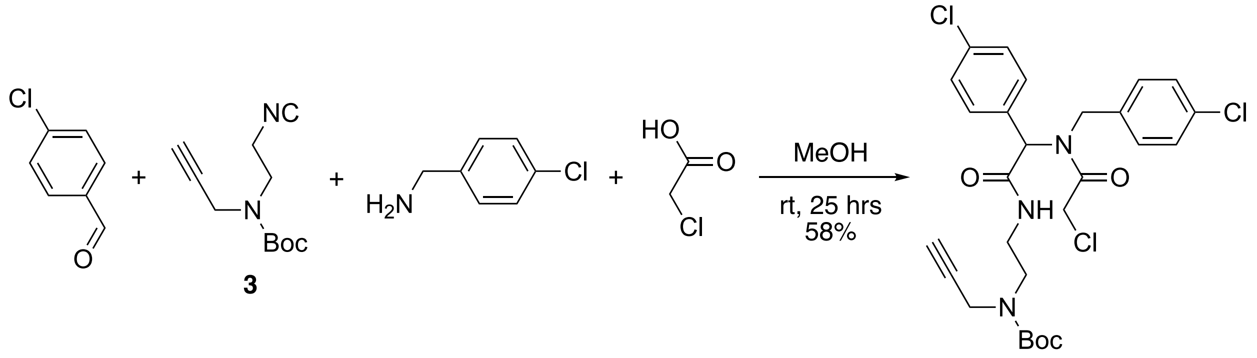

(±)-**10**
((±)-PJDU-200)

*(±)-tert-Butyl (2-(2-(2-chloro-N-(4-chlorobenzyl)acetamido)-2-(4-chlorophenyl)acetamido)ethyl)(prop-2-yn-1-yl)carbamate ((±)-**10**) ((±)-PJDU-200).* A flame-dried 2-dram vial equipped with a stir-bar was charged sequentially with 4-chlorobenzaldehyde (140 mg, 0.99 mmol, 1.1 eq.), 4-chlorobenzylamine (0.12 mL, 0.99 mmol, 1.1 eq.), and MeOH (1 mL). This mixture was stirred for 15 minutes at room temperature to form the presumed imine. Next, a solution of isocyanide **3** (197 mg, 0.95 mmol, 1 eq.) dissolved in MeOH (1.5 mL) was added to the reaction flask via cannula. After 15 minutes, 2-chloroacetic acid (94 mg, 0.99 mmol, 1.1 eq.) was added to the reaction flask, followed by stirring at room temperature for 25 hours, wherein TLC analysis indicated a complete reaction. Next, the reaction mixture was concentrated under reduced pressured, and then the crude residue was dissolved in EtOAc (75 mL) and transferred to a separatory funnel. The organic layer was washed sequentially with sat. aq. $NaHCO_3$ (15 mL), H_2O (15 mL), aq. 1M HCl (15 mL), and brine (15 mL), then dried ($MgSO_4$), filtered, concentrated under reduced pressure, and purified by silica gel flash column chromatography (gradient elution with 25-50% EtOAc/hexanes) to provide amide (±)-**10** (309 mg, 58%, (±)-PJDU-200) as a sticky white solid.

R_f = 0.43 (50% EtOAc/hexanes)

^{1}H NMR (400 MHz, $CDCl_3$, *peaks reported are for the major rotamer only) δ 7.27 (dd, *J* = 24.0, 15.6 Hz, 2H), 7.20 (d, *J* = 8.2 Hz, 3H), 7.03 (s, 1H), 6.97 (d, *J* = 8.0 Hz, 2H), 6.85 (s, 1H), 5.94 (s, 1H), 4.73 (s, 1H), 4.58 (s, 1H), 4.05 (s, 1H), 3.98 (s, 2H), 3.95 (d, *J* = 14.1 Hz, 1H), 3.52 – 3.33 (m, 4H), 2.22 (s, 1H), 1.39 (s, 9H)

^{13}C NMR (101 MHz, $CDCl_3$, *peaks reported are for the major rotamer only) δ 168.7, 168.0, 155.5, 135.0, 134.8, 133.2, 132.7, 130.9, 129.0, 128.7, 127.3, 80.7, 79.4, 71.8, 62.3, 49.2, 45.8, 42.0, 39.2, 37.3, 28.1

HRMS (ESI+) *m/z* [M + Na] calcd for $C_{27}H_{30}Cl_3N_3O_4Na$ 588.1194, found 588.1196

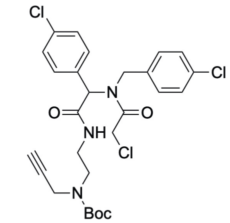

(±)-**10**
((±)-PJDU-200)
^{1}H NMR (400 MHz, $CDCl_3$ with TMS)

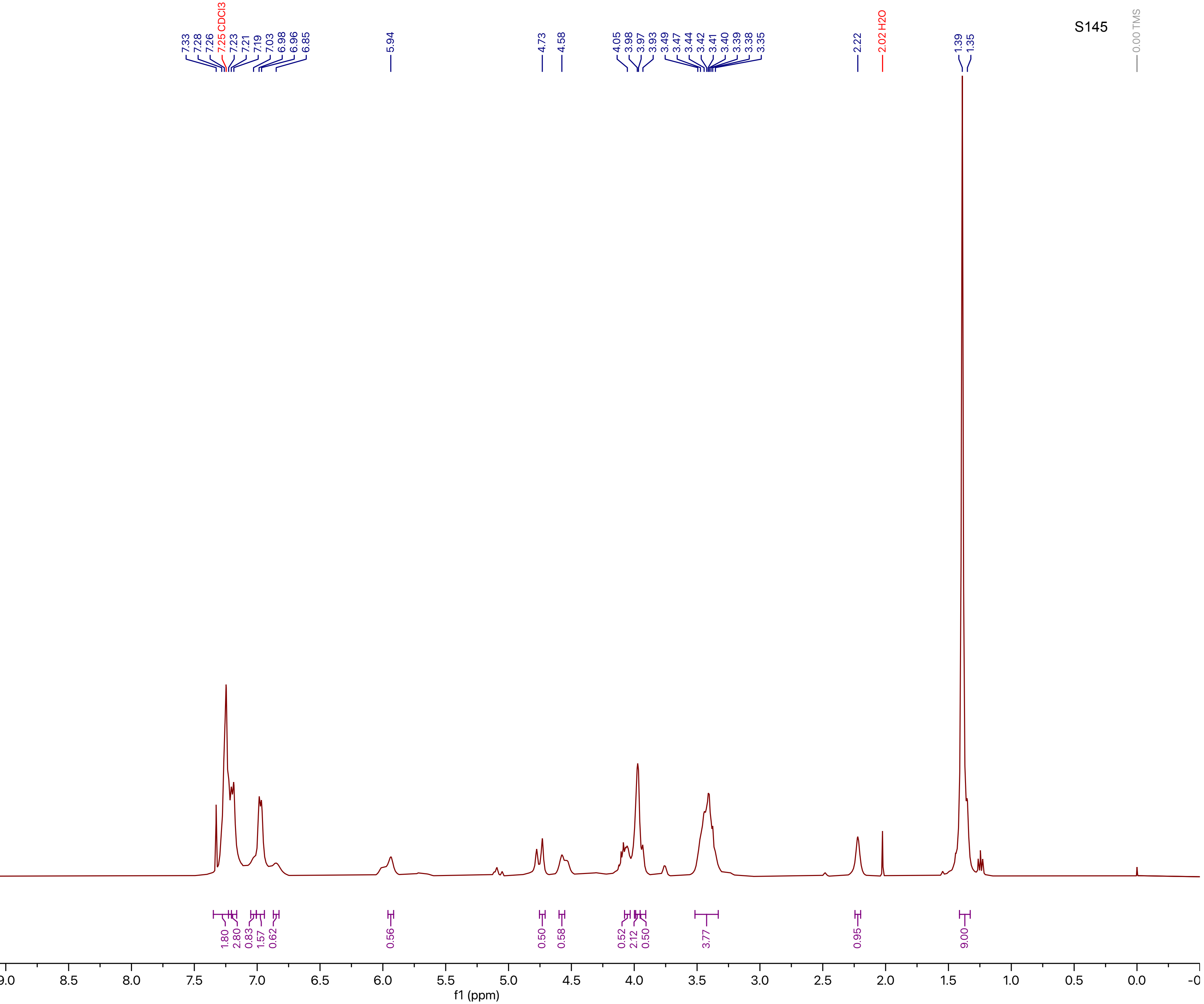

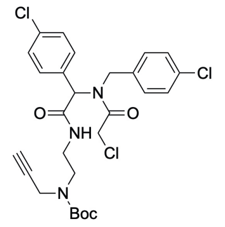

(±)-**10**
((±)-PJDU-200)
^{13}C NMR (101 MHz, $CDCl_3$ with TMS)

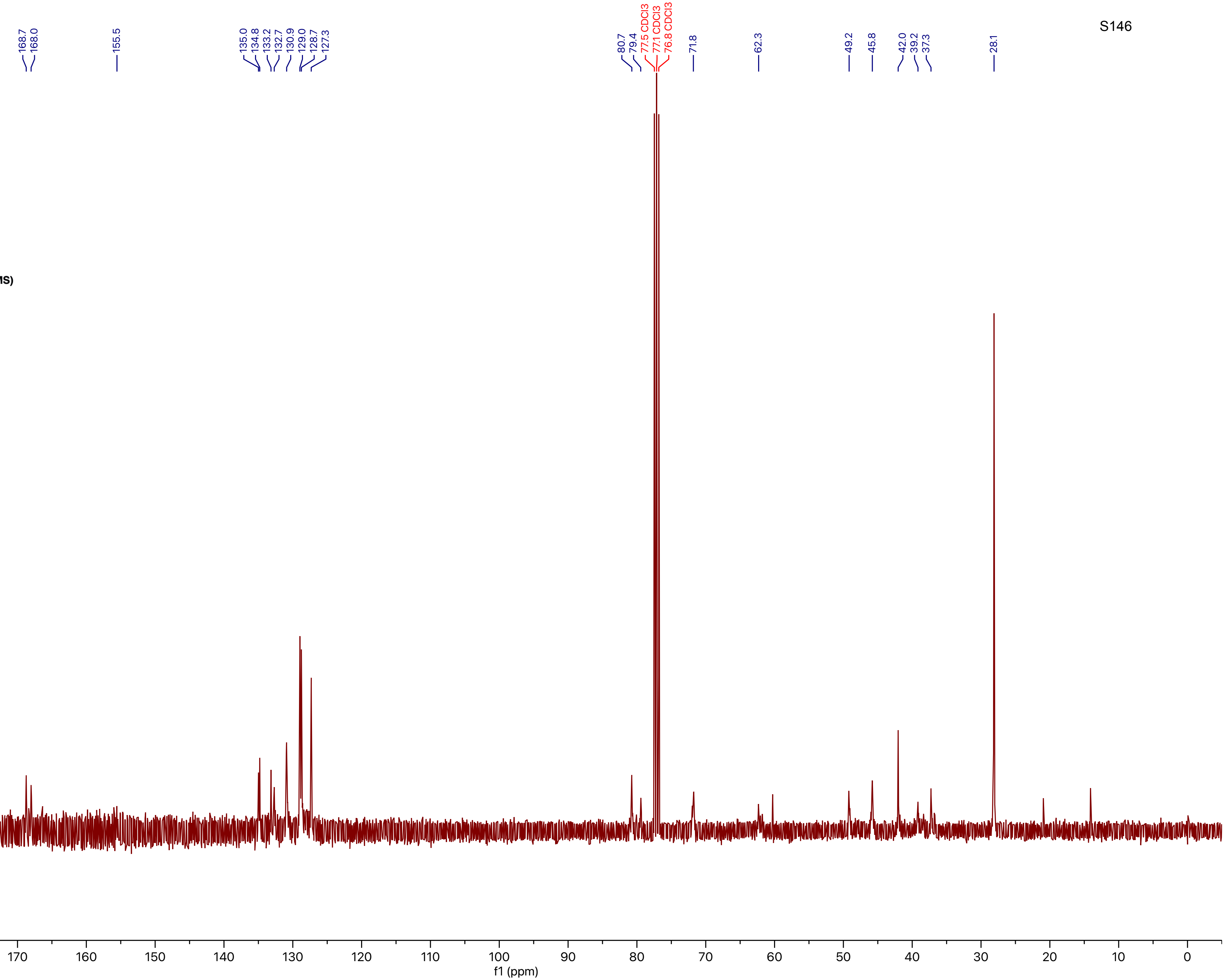

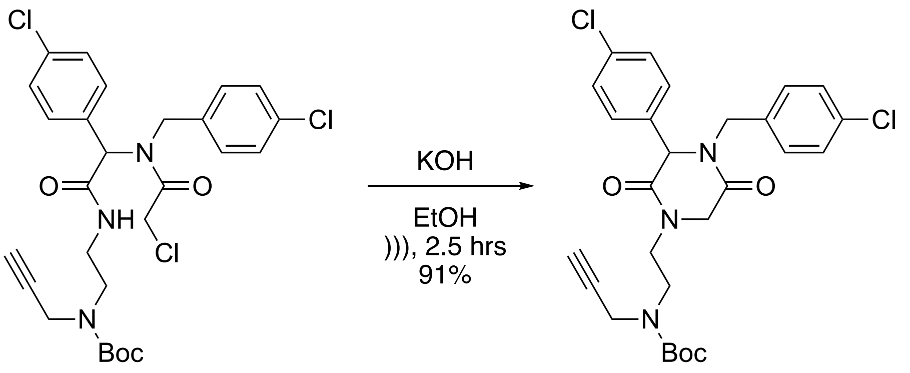

(±)-**10**
((±)-PJDU-200)

(±)-**11**
((±)-PJDU-202)

*(±)-tert-Butyl (2-(4-(4-chlorobenzyl)-3-(4-chlorophenyl)-2,5-dioxopiperazin-1-yl)ethyl)(prop-2-yn-1-yl)carbamate ((±)-**11**) ((±)-PJDU-202).* A 25 mL round-bottom flask was charged with α-chloro amide (±)-**10** (305 mg, 0.54 mmol, 1 eq., (±)-PJDU-200) and anhydrous EtOH (4 mL). Potassium hydroxide (33 mg, 0.59 mmol, 1.1 eq.) was added in a single portion and the reaction flask was placed in a sonicator for 2.5 h, after which TLC indicated consumption of the starting material. The reaction mixture was then transferred to a separatory funnel and partitioned between H_2O (20 mL) and EtOAc (75 mL). The layers were separated and the organic layer was subsequently washed with brine (10 mL), dried ($MgSO_4$), filtered, and concentrated under reduced pressure to provide diketopiperazine (±)-**11** (258 mg, 91%) as a sticky yellow solid requiring no further purification.

R_f = 0.53 (50% EtOAc/hexanes)

^{1}H NMR (500 MHz, $CDCl_3$, *peaks reported are for the major rotamer only) δ 7.37 (d, *J* = 8.0 Hz, 2H), 7.31 – 7.20 (m, 4H), 7.18 – 7.10 (m, 2H), 5.40 (d, *J* = 14.8 Hz, 1H), 4.83 (s, 1H), 4.32 – 4.22 (m, 1H), 4.15 (s, 1H), 3.95 (s, 1H), 3.89 (s, 1H), 3.61 – 3.45 (m, 2H), 3.51 (s, 3H), 2.19 (t, *J* = 2.4 Hz, 1H), 1.47 – 1.37 (m, 9H)

^{13}C NMR (126 MHz, $CDCl_3$, *peaks reported are for the major rotamer only) δ 164.3, 164.2, 154.9, 135.0, 134.1, 133.5, 130.0, 129.5, 129.1, 128.0, 80.8, 79.3, 72.0, 62.4, 50.1, 46.7, 44.6, 43.1, 36.8, 28.2

HRMS (ESI+) *m/z* [M + Na] calcd for $C_{27}H_{29}Cl_2N_3O_4Na$ 552.1427, found 552.1428

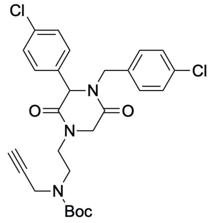

(±)-**11**
((±)-PJDU-202)
^{1}H NMR (500 MHz, $CDCl_3$ with TMS)

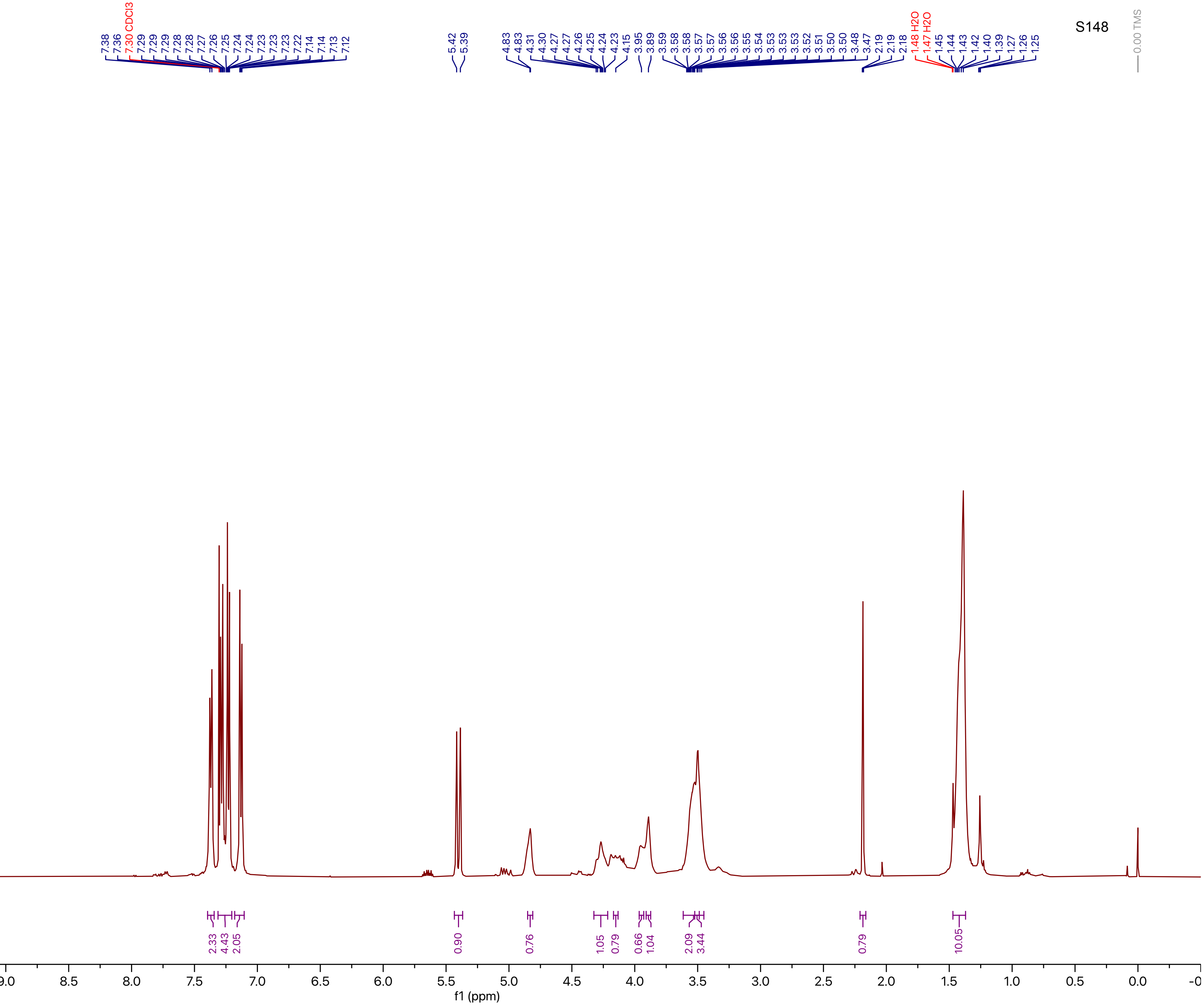

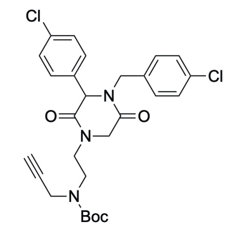

(±)-**11**
((±)-PJDU-202)
^{13}C NMR (126 MHz, $CDCl_3$ with TMS)

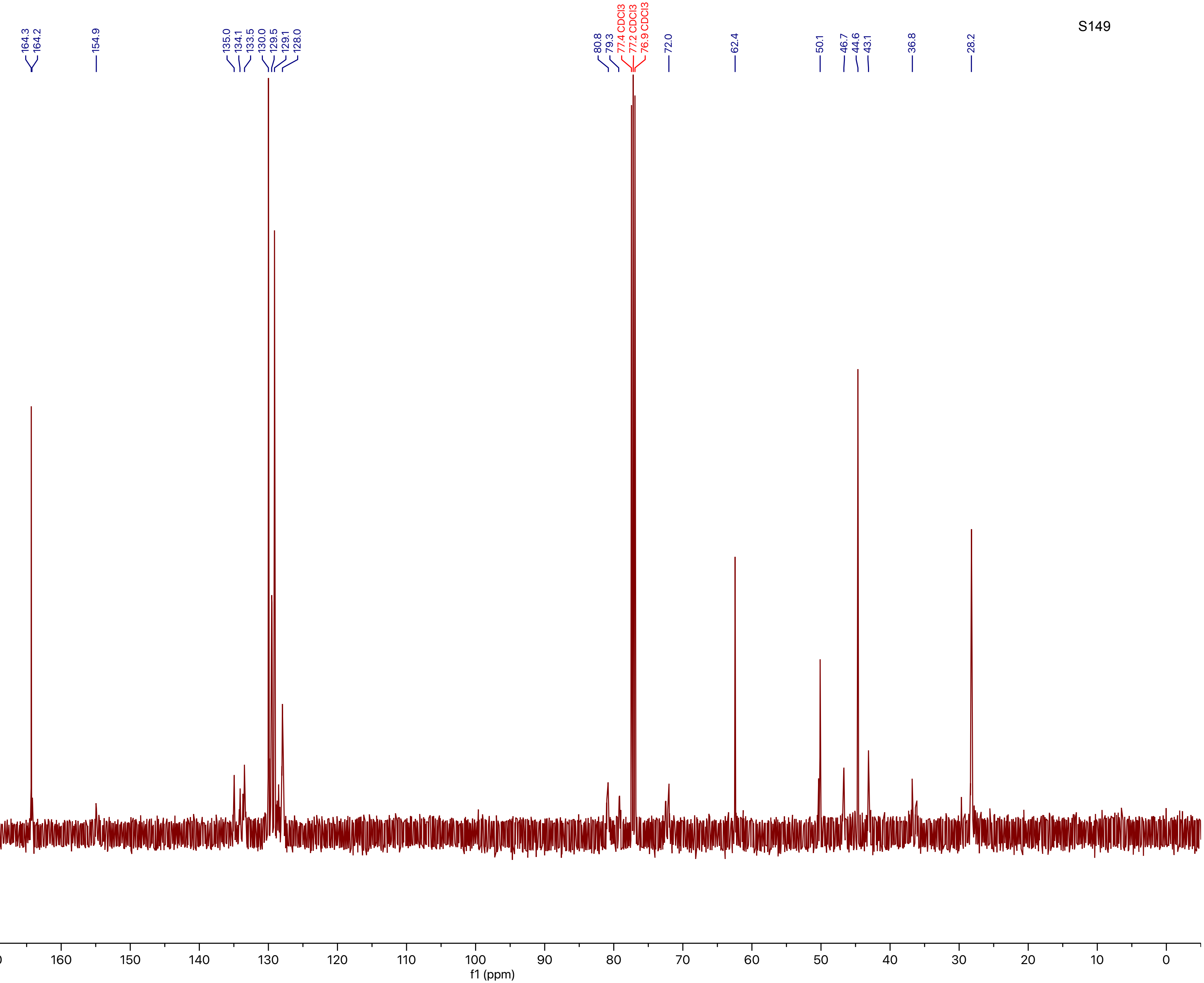

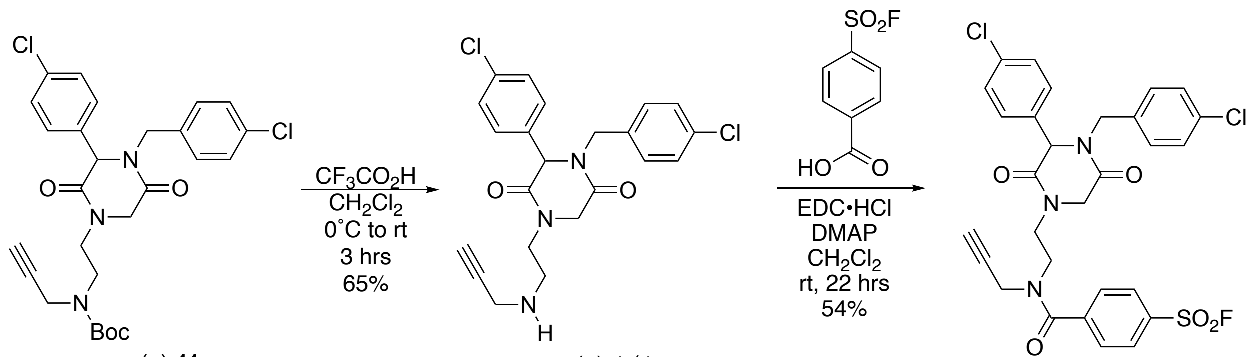

(±)-**11** ((±)-PJDU-202)

(±)-4-(4-chlorobenzyl)-3-(4-chlorophenyl)-1-(2-(prop-2-yn-1-ylamino)ethyl)piperazine-2,5-dione ((±)-PJDU-203)

(±)-**4n** ((±)-PJDU-204) 35% over two steps

(±)-4-(4-Chlorobenzyl)-3-(4-chlorophenyl)-1-(2-(prop-2-yn-1-ylamino)ethyl)piperazine-2,5-dione ((±)-PJDU-203). A 25 mL flame-dried, round-bottom flask equipped with a stirbar, septum, and argon inlet needle was charged with Boc carbamate substrate (±)-**11** (250 mg, 0.47 mmol, 1.0 eq.; (±)-PJDU-202) and CH_2Cl_2 (5 mL) and cooled to 0˚C. Trifluoroacetic acid (0.43 mL, 5.7 mmol, 12.0 eq.) was then added dropwise over 2 minutes. After the addition, the ice bath was removed and the reaction was stirred at room temperature for 3 hours, wherein TLC indicated complete consumption of the starting Boc carbamate substrate. The reaction was then quenched by dropwise addition of sat. aq. $NaHCO_3$ (vigorous gas evolution) to the reaction mixture until gas evolution ceased (~10 mL), then 1M aq. NaOH (~5 mL) was added to raise the pH to 8-9 (checked with pH paper). This biphasic mixture was then transferred to a separatory funnel and diluted with CH_2Cl_2 (30 mL), the layers were separated, and the aqueous layer was extracted with CH_2Cl_2 (3 x 15 mL). The combined organic layers were washed with brine (10 mL), dried ($MgSO_4$), gravity filtered, and concentrated under reduced pressure. The crude residue was then subjected to silica gel chromatography (gradient elution with 85-95% EtOAc/hexanes, then 5% MeOH/EtOAc) to give propargyl amine (±)-PJDU-203 (131 mg, 65%) as a sticky white solid.

R_f = 0.06 (85% EtOAc/hexanes)

^{1}H NMR (500 MHz, $CDCl_3$, *peaks reported are for the major rotamer only) δ 7.40 – 7.34 (m, 2H), 7.32 – 7.18 (m, 4H), 7.18 – 7.10 (m, 2H), 5.40 (d, *J* = 14.9 Hz, 1H), 4.84 (s, 1H), 4.34 (d, *J* = 17.6 Hz, 1H), 4.11 (d, *J* = 17.6 Hz, 1H), 3.64 (dt, *J* = 14.0, 6.2 Hz, 1H), 3.57 (d, *J* = 14.9 Hz, 1H), 3.32 (s, 1H), 3.31 (s, 1H), 3.32 – 3.24 (m, 1H), 2.86 (t, *J* = 6.1 Hz, 2H), 2.19 (t, *J* = 2.4 Hz, 1H), 1.28 (s, 1H)

^{13}C NMR (126 MHz, $CDCl_3$, *peaks reported are for the major rotamer only) δ 164.5, 164.3, 134.9, 134.1, 133.8, 133.5, 129.9, 129.5, 129.1, 128.1, 81.7, 71.7, 62.5, 50.4, 46.7, 45.8, 45.1, 37.7

HRMS (ESI+) *m/z* [M + H] calcd for $C_{22}H_{21}Cl_2N_3O_2H$ 430.1084, found 430.1085

*(±)-4-((2-(4-(4-Chlorobenzyl)-3-(4-chlorophenyl)-2,5-dioxopiperazin-1-yl)ethyl)(prop-2-yn-1-yl)carbamoyl)benzenesulfonyl fluoride ((±)-**4n**) ((±)-PJDU-204).* A 25 mL flame-dried round-bottom flask equipped with a stir-bar, septum, and argon inlet needle was charged with 4-fluorosulfonyl benzoic acid (50 mg, 0.39 mmol, 1.5 eq.) and CH_2Cl_2 (2 mL), then EDC•HCl (99 mg, 0.52 mmol, 2 eq.) and DMAP (4 mg, 0.03 mmol, 0.1 eq.) were added sequentially. Next a solution of propargyl amine (±)-PJDU-203 (111 mg, 0.26 mmol, 1 eq.) in CH_2Cl_2 (4.5 mL) was added via cannula. The reaction mixture was stirred at room temperature for 22 hours, wherein

TLC analysis indicated a complete reaction. Next, the reaction mixture was concentrated and then transferred to a separatory funnel with EtOAc (50 mL). The organic layers were then washed sequentially with 1M aq. HCl solution (10 mL), H_2O (10 mL), sat. aq. $NaHCO_3$ (10 mL), and brine (10 mL) then dried ($MgSO_4$), filtered, and concentrated under reduced pressure. The crude residue was then purified by silica gel flash column chromatography (elution with 50-60% EtOAc/hexanes) to give FFSMP (±)-**4n** (86 mg, 54%; (±)-PJDU-204) as a white sticky solid.

R_f = 0.33 (75% EtOAc/hexanes)

^{1}H NMR (500 MHz, $CDCl_3$, *peaks reported are for the major rotamer only) δ 7.91 – 7.86 (m, 2H), 7.32 – 7.21 (m, 2H), 7.24 – 7.17 (m, 4H), 7.14 – 7.08 (m, 2H), 7.05 (d, *J* = 8.3 Hz, 2H), 5.44 (d, *J* = 14.8 Hz, 1H), 4.77 (s, 1H), 4.71 (d, *J* = 17.4 Hz, 1H), 4.23 (ddd, *J* = 13.7, 9.6, 3.0 Hz, 1H), 4.19 – 4.06 (m, 2H), 4.00 (dd, *J* = 18.3, 2.5 Hz, 1H), 3.87 (dd, *J* = 17.9, 2.4 Hz, 1H), 3.63 (ddd, *J* = 14.4, 5.0, 3.1 Hz, 1H), 3.40 (d, *J* = 14.8 Hz, 1H), 3.29 (ddd, *J* = 14.4, 5.0, 3.1 Hz, 1H), 2.33 (t, *J* = 2.4 Hz, 1H)

^{13}C NMR (126 MHz, $CDCl_3$, *peaks reported are for the major rotamer only) δ 169.1, 165.1, 163.9, 141.7, 135.2, 134.2, 134.0, 133.6, 130.1, 129.5, 129.2, 128.7, 128.6, 127.7, 77.2, 74.4, 62.6, 49.8, 46.4, 43.8, 40.5, 38.6

HRMS (ESI+) *m/z* [M + Na] calcd for $C_{29}H_{24}Cl_2FN_3O_5SNa$ 638.0690, found 638.0689

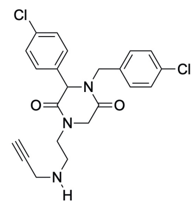

(±)-4-(4-chlorobenzyl)-3-(4-chlorophenyl)-1-(2-(prop-2-yn-1-ylamino)ethyl)piperazine-2,5-dione
((±)-PJDU-203)
^{1}H NMR (500 MHz, $CDCl_3$ with TMS)

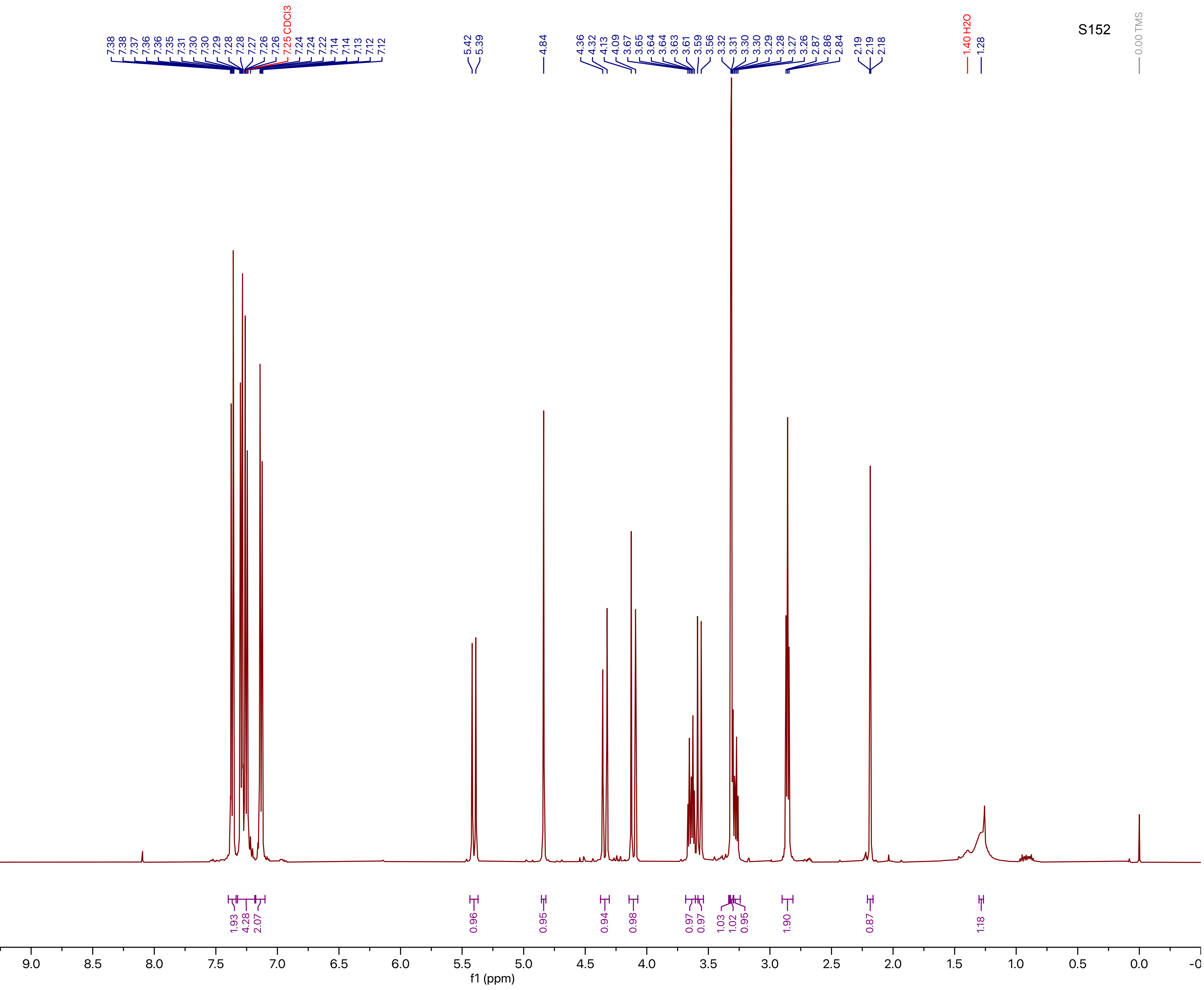

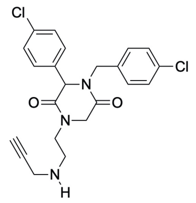

(±)-4-(4-chlorobenzyl)-3-(4-chlorophenyl)-1-(2-(prop-2-yn-1-ylamino)ethyl)piperazine-2,5-dione
((±)-PJDU-203)
^{13}C NMR (126 MHz, $CDCl_3$ with TMS)

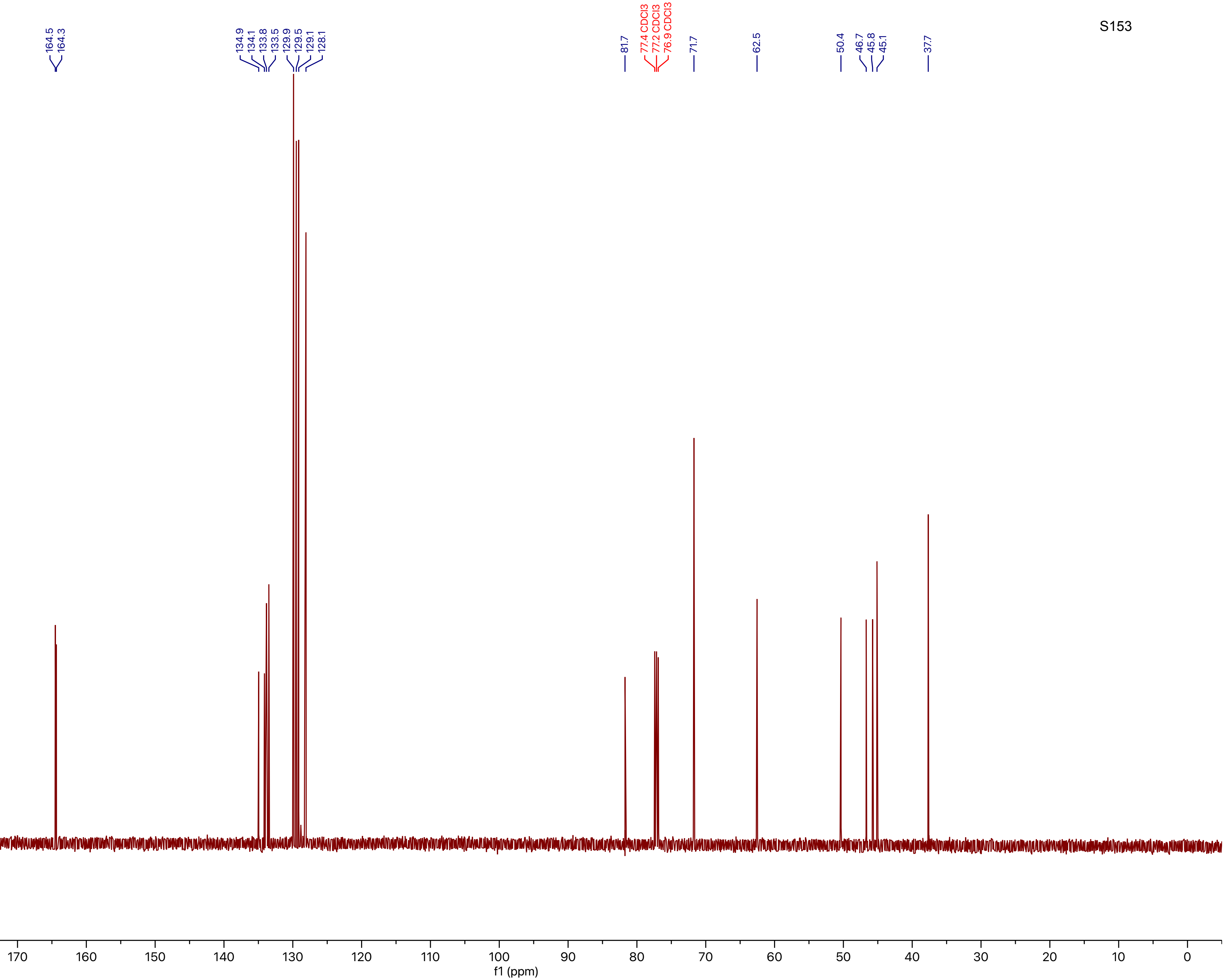

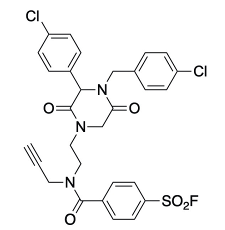

(±)-**4n**
((±)-PJDU-204)
^{1}H NMR (500 MHz, $CDCl_3$ with TMS)

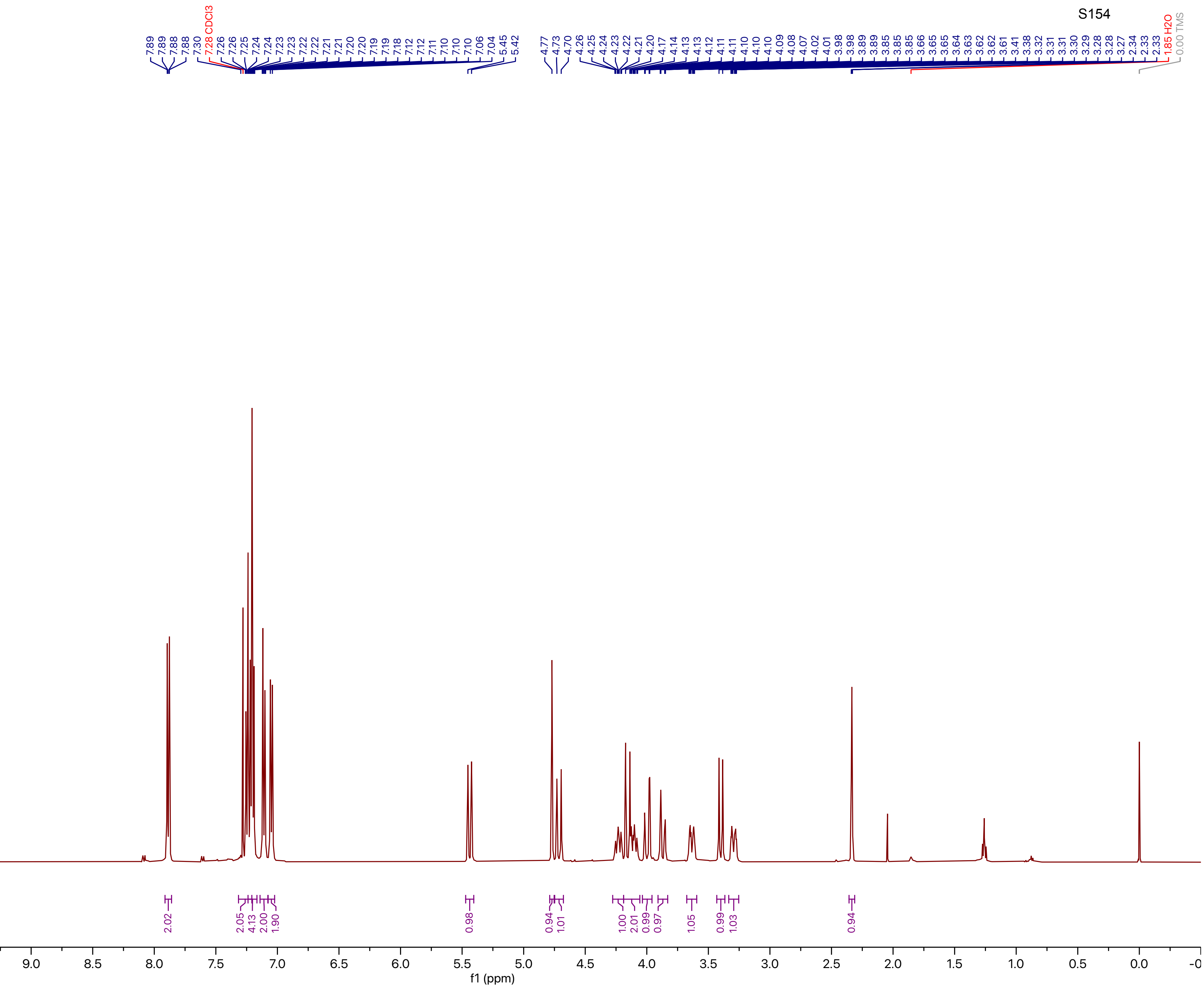

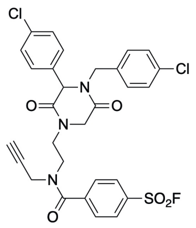

(±)-**4n**
((±)-PJDU-204)
^{13}C NMR (126 MHz, $CDCl_3$ with TMS)

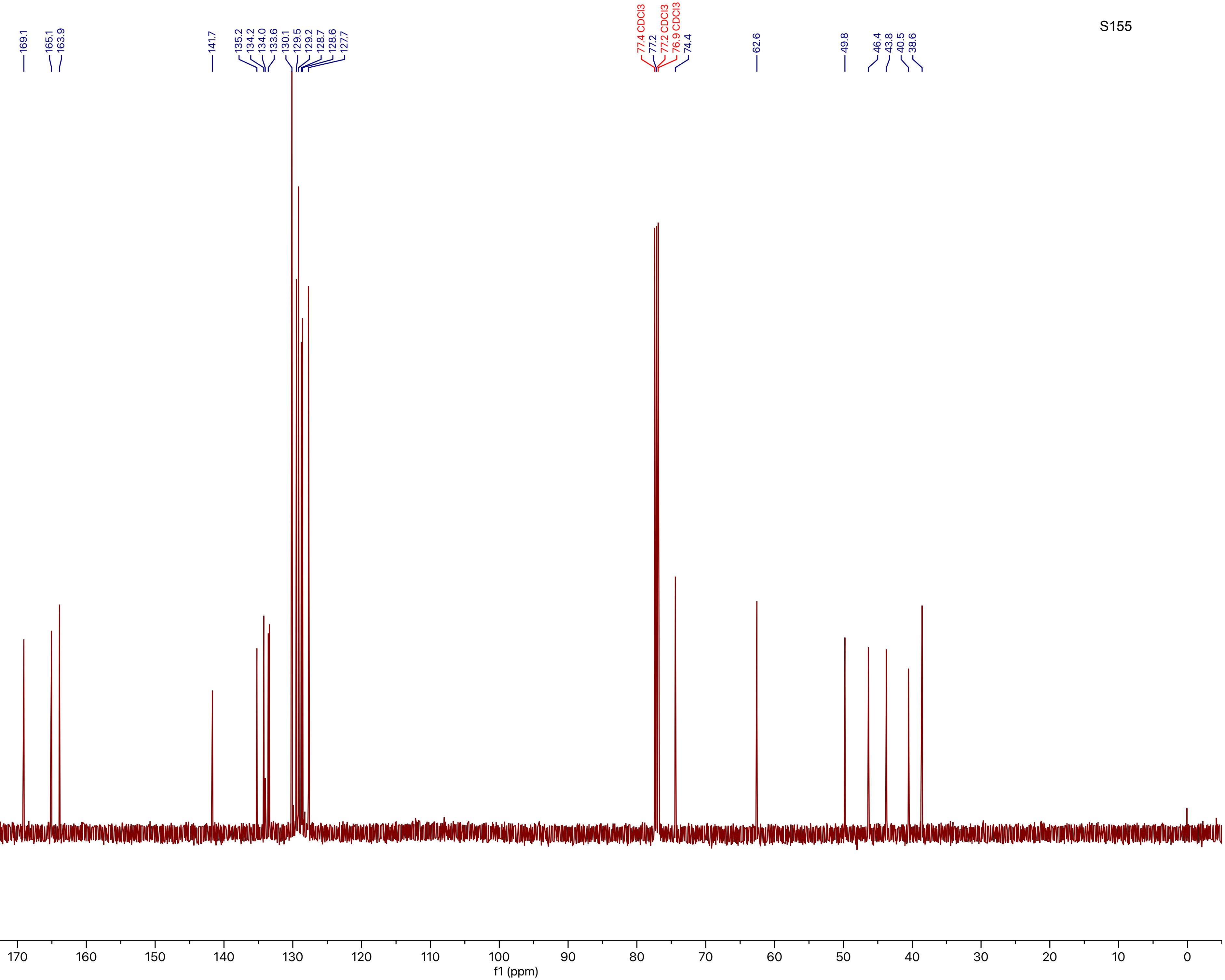

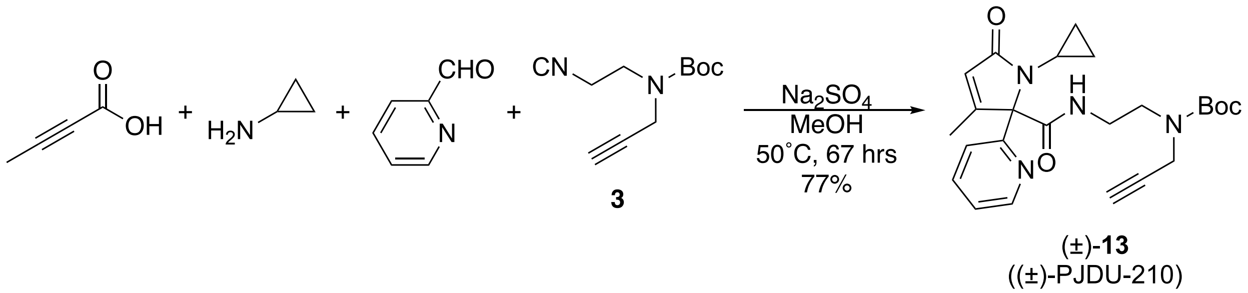

*(±)-tert-Butyl (2-(1-cyclopropyl-3-methyl-5-oxo-2-(pyridin-2-yl)-2,5-dihydro-1H-pyrrole-2-carboxamido)ethyl)(prop-2-yn-1-yl)carbamate ((±)-**13**) ((±)-PJDU-210).* A 2-dram vial equipped with a stir-bar was charged with 2-pyridine carboxaldehyde (0.08 mL, 0.83 mmol, 1 eq.) and MeOH (0.6 mL). Next, sodium sulfate (300 mg, 1.67 mmol, 1.3 eq.) was added all at once, followed by addition of cyclopropylamine (0.07 mL, 0.99 mmol, 1.2 eq.) in a single portion. The resulting mixture was stirred for 30 minutes at room temperature to form the presumed imine, then 2-butynoic acid (83 mg, 0.99 mmol, 1.2 eq.) was added in a single portion, followed immediately by addition of a solution of isocyanide **3** (207 mg, 0.99 mmol, 1.2 eq.) in MeOH (1.4 mL) via cannula. Following the addition, the reaction vial was capped and heated to 50°C and stirred for 67 hours, wherein TLC indicated a complete reaction. Next, the crude reaction mixture was diluted with MeOH (5 mL) and then transferred to a separatory funnel containing a solution of sat. aq. sodium bisulfite (25 mL). The separatory funnel was shaken for 30 seconds giving a homogenous brown solution, which was extracted with EtOAc (3 x 40 mL). The combined organic layers were washed sequentially with sat. aq. $NaHCO_3$ (15 mL) and brine (15 mL), dried ($MgSO_4$), filtered, and concentrated under reduced pressure. The resulting crude residue was purified by silica gel flash column chromatography (gradient elution with 50-100% EtOAc/hexanes) to give lactam (±)-**13** (282 mg, 77%, (±)-PJDU-210) as a yellow, sticky solid.

R_f = 0.14 (100% EtOAc)

^{1}H NMR (400 MHz, $CDCl_3$, *peaks reported are for the major rotamer only) δ 10.23-10.06 (m, 1H), 8.63 (d, *J* = 4.0 Hz, 1H), 7.78 (t, *J* = 7.4 Hz, 1H), 7.36 (d, *J* = 8.0 Hz, 2H), 5.94 (d, *J* = 1.2 Hz, 1H), 4.17-4.07 (m, 2H), 3.63 (m, 4H), 2.47 (m, 1H), 2.30 (app s, 1H), 1.79 (d, *J* = 1.2 Hz, 3H), 1.46 (s, 9H), 0.82-0.78 (m, 1H), 0.58-0.53 (m, 2H), 0.51-0.49 (m, 1H)

^{13}C NMR (101 MHz, $CDCl_3$, *peaks reported are for the major rotamer only) δ 174.8, 166.9, 166.7, 160.1, 156.3, 155.0, 147.6, 137.7, 123.2, 122.9, 80.5, 79.4, 71.7, 60.2, 45.4, 38.3, 36.7, 28.2, 24.7, 13.3, 4.6, 4.4

HRMS (ESI+) *m/z* [M + Na] calcd for $C_{24}H_{30}N_4O_4Na$ 461.2159, found 461.2161

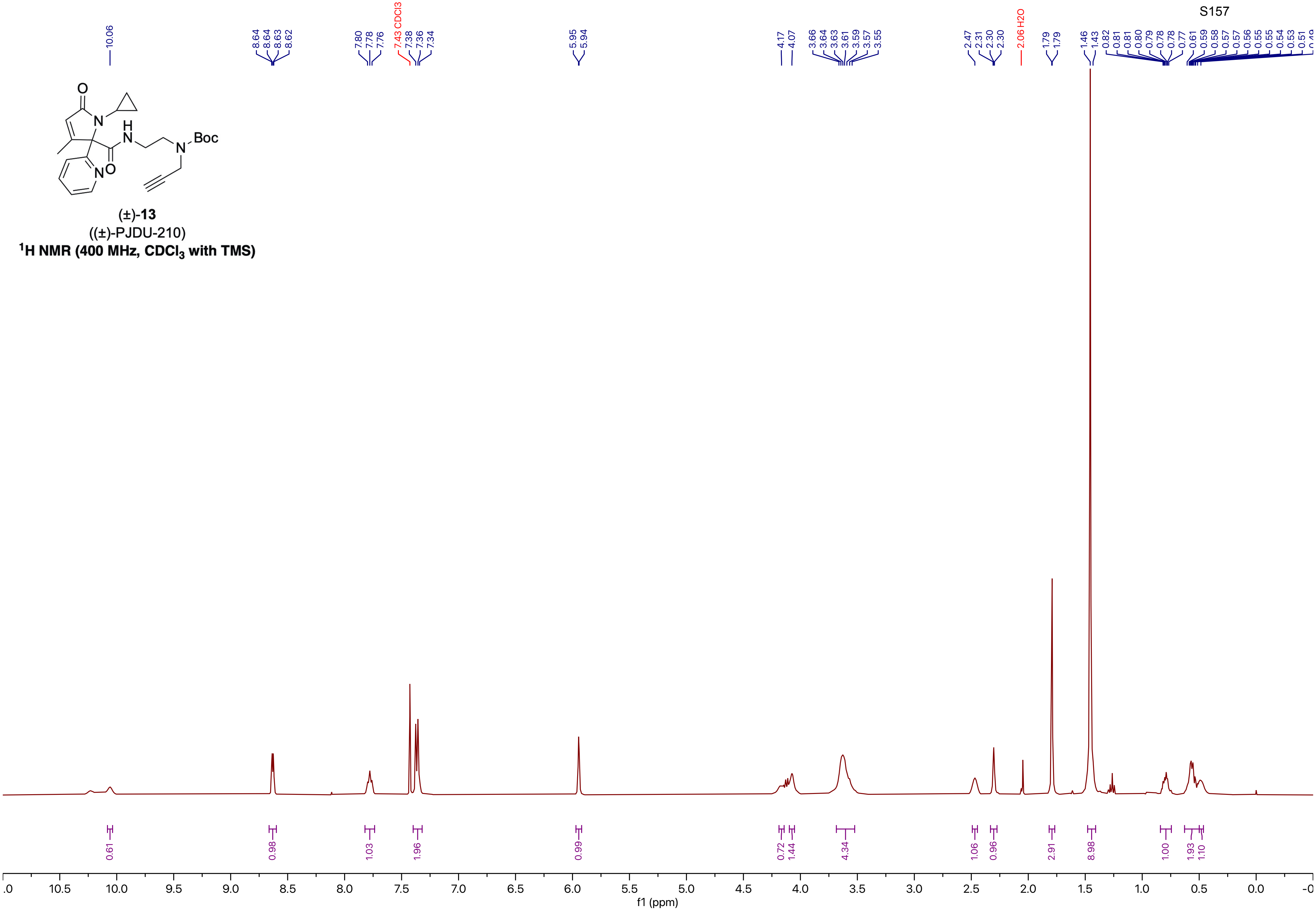

(±)-**13**
((±)-PJDU-210)
^{1}H NMR (400 MHz, $CDCl_3$ with TMS)

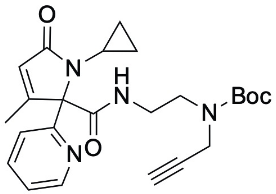

(±)-**13**
((±)-PJDU-210)
^{13}C NMR (101 MHz, $CDCl_3$ with TMS)

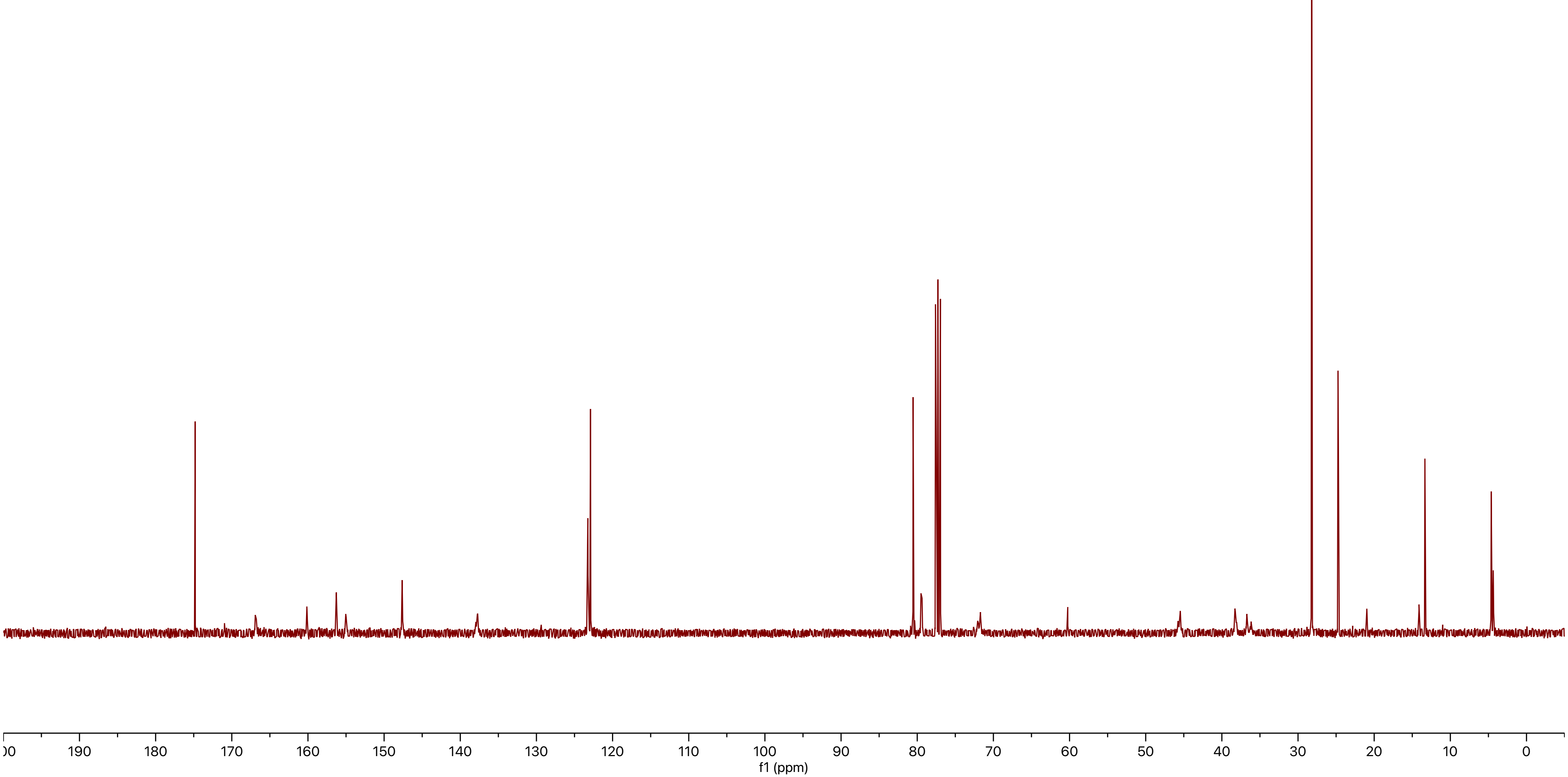

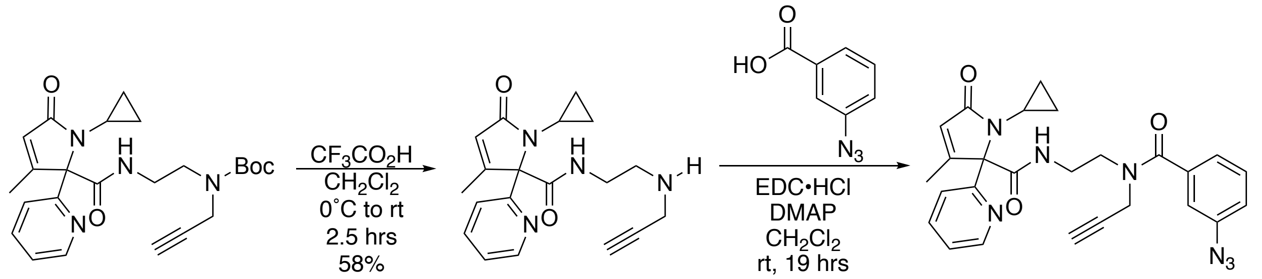

(±)-**13**
((±)-PJDU-210)

(±)-1-cyclopropyl-3-methyl-5-oxo-*N*-(2-(prop-2-yn-1-ylamino)ethyl)-2-(pyridin-2-yl)-2,5-dihydro-1*H*-pyrrole-2-carboxamide
((±)-PJDU-213)

(±)-**4o**
((±)-PJDU-214)
33% over two steps

(±)-1-Cyclopropyl-3-methyl-5-oxo-N-(2-(prop-2-yn-1-ylamino)ethyl)-2-(pyridin-2-yl)-2,5-dihydro-1H-pyrrole-2-carboxamide ((±)-PJDU-213). A 25 mL flame-dried, round-bottom flask equipped with a stirbar, septum, and argon inlet needle was charged with Boc carbamate substrate (±)-**13** (275 mg, 0.63 mmol, 1.0 eq.; (±)-PJDU-210) and CH_2Cl_2 (6.3 mL) and cooled to 0˚C. Trifluoroacetic acid (0.58 mL, 7.5 mmol, 12.0 eq.) was then added dropwise over 2 minutes. After the addition, the ice bath was removed and the reaction was stirred at room temperature for 2.5 hours, wherein TLC indicated complete consumption of the starting Boc carbamate substrate. The reaction was then quenched by dropwise addition of sat. aq. $NaHCO_3$ (vigorous gas evolution) to the reaction mixture until gas evolution ceased (12 mL), then 1M aq. NaOH (~5 mL) was added to raise the pH to 8-9 (checked with pH paper). This biphasic mixture was then transferred to a separatory funnel and diluted with CH_2Cl_2 (20 mL), the layers were separated, and the aqueous layer was extracted with CH_2Cl_2 (2 x 20 mL). The combined organic layers were washed with brine (10 mL), dried ($MgSO_4$), gravity filtered, and concentrated under reduced pressure to give propargyl amine (±)-PJDU-213 (124 mg, 58%) as a sticky yellow solid, which proved sufficiently pure to take on to the N-acylation arming step.

R_f = 0.15 (5% MeOH/ CH_2Cl_2)

^{1}H NMR (500 MHz, $CDCl_3$, *peaks reported are for the major rotamer only) δ 9.84 (t, *J* = 5.0 Hz, 1H), 8.61 (ddd, *J* = 1.0, 2.0, 5.0 Hz, 1H), 7.75 (td, *J* = 2.0, 8.0 Hz, 1H), 7.38 (dt, *J* = 1.0, 8.0 Hz, 1H), 7.32 (ddd, *J* = 1.0, 5.0, 8.0 Hz, 1H), 5.95 (q, *J* = 1.5 Hz, 1H), 3.61-3.55 (m, 1H), 3.52-3.48 (m, 1H), 3.47 (d, *J* = 2.5 Hz, 2H), 2.96-2.93 (m, 2H), 2.46-2.42 (m, 1H), 2.25 (t, *J* = 2.5 Hz, 1H), 1.81 (d, *J* = 1.5 Hz, 3H), 1.60 (bs, 1H), 0.83-0.79 (m, 1H), 0.59-0.55 (m, 2H), 0.54-0.50 (m, 1H)

^{13}C NMR (126 MHz, $CDCl_3$, *peaks reported are for the major rotamer only) δ 175.1, 166.8, 160.4, 156.4, 147.8, 137.7, 123.3, 123.2, 123.1, 81.9, 79.7, 71.6, 47.4, 39.6, 37.8, 24.9, 13.5, 4.63, 4.60

HRMS (ESI+) *m/z* [M + Na] calcd for $C_{19}H_{22}N_4O_2Na$ 361.1635, found 361.1638

*(±)-N-(2-(3-Azido-N-(prop-2-yn-1-yl)benzamido)ethyl)-1-cyclopropyl-3-methyl-5-oxo-2-(pyridin-2-yl)-2,5-dihydro-1H-pyrrole-2-carboxamide ((±)-**4o**) ((±)-PJDU-214).* A 25 mL flame-dried round-bottom flask equipped with a stir-bar, septum, and argon inlet needle was charged with 3-azidobenzoic acid (87 mg, 0.53 mmol, 1.5 eq.) and CH_2Cl_2 (5 mL), then EDC•HCl (134 mg, 0.70 mmol, 2 eq.) and DMAP (4 mg, 0.04 mmol, 0.1 eq.) were added sequentially. Next a solution of propargyl amine (±)-PJDU-213 (120 mg, 0.35 mmol, 1 eq.) in CH_2Cl_2 (6 mL) was added via cannula. The reaction mixture was stirred at room temperature for 19 hours, wherein TLC analysis indicated a complete reaction. Next, the reaction mixture was concentrated and then transferred

to a separatory funnel with EtOAc (60 mL). The organic layers were then washed sequentially with H_2O (10 mL), sat. aq. $NaHCO_3$ (10 mL), H_2O (10 mL), and brine (10 mL) then dried ($MgSO_4$), filtered, and concentrated under reduced pressure. The crude residue was then purified by silica gel flash column chromatography (elution with 75-100% EtOAc/hexanes, then 10% MeOH/EtOAc) to give FFSMP (±)-**4o** (97 mg, 57%; (±)-PJDU-214) as a white sticky solid.

R_f = 0.14 (5% MeOH/CH_2Cl_2)

^{1}H NMR (400 MHz, $CDCl_3$, *peaks reported are for the major rotamer only) δ 10.32 (bs, 1H), 8.63-8.62 (m, 1H), 7.76 (t, *J* = 7.4 Hz, 1H), 7.41-7.09 (m, 6H), 5.95 (s, 1H), 4.54-3.93 (m, 3H), 3.76 (m, 3H), 2.50 (m, 1H), 2.43 (app s, 1H), 1.77 (s, 3H), 0.77 (m, 1H), 0.59-0.47 (m, 3H)

^{13}C NMR (101 MHz, $CDCl_3$, *peaks reported are for the major rotamer only) δ 174.9, 170.7, 167.3, 160.1, 156.4, 147.7, 140.7, 138.0, 136.9, 130.1, 123.5, 123.3, 123.2, 122.8, 120.8, 117.5, 79.5, 78.4, 73.9, 44.3, 39.5, 37.7, 28.3, 13.4, 4.9

HRMS (ESI+) *m/z* [M + Na] calcd for $C_{26}H_{25}N_7O_3Na$ 506.1911, found 506.1915

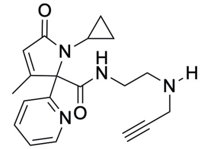

(±)-1-cyclopropyl-3-methyl-5-oxo-*N*-(2-(prop-2-yn-1-ylamino)ethyl)-2-(pyridin-2-yl)-2,5-dihydro-1*H*-pyrrole-2-carboxamide

((±)-PJDU-213)

^{1}H NMR (500 MHz, $CDCl_3$ with TMS)

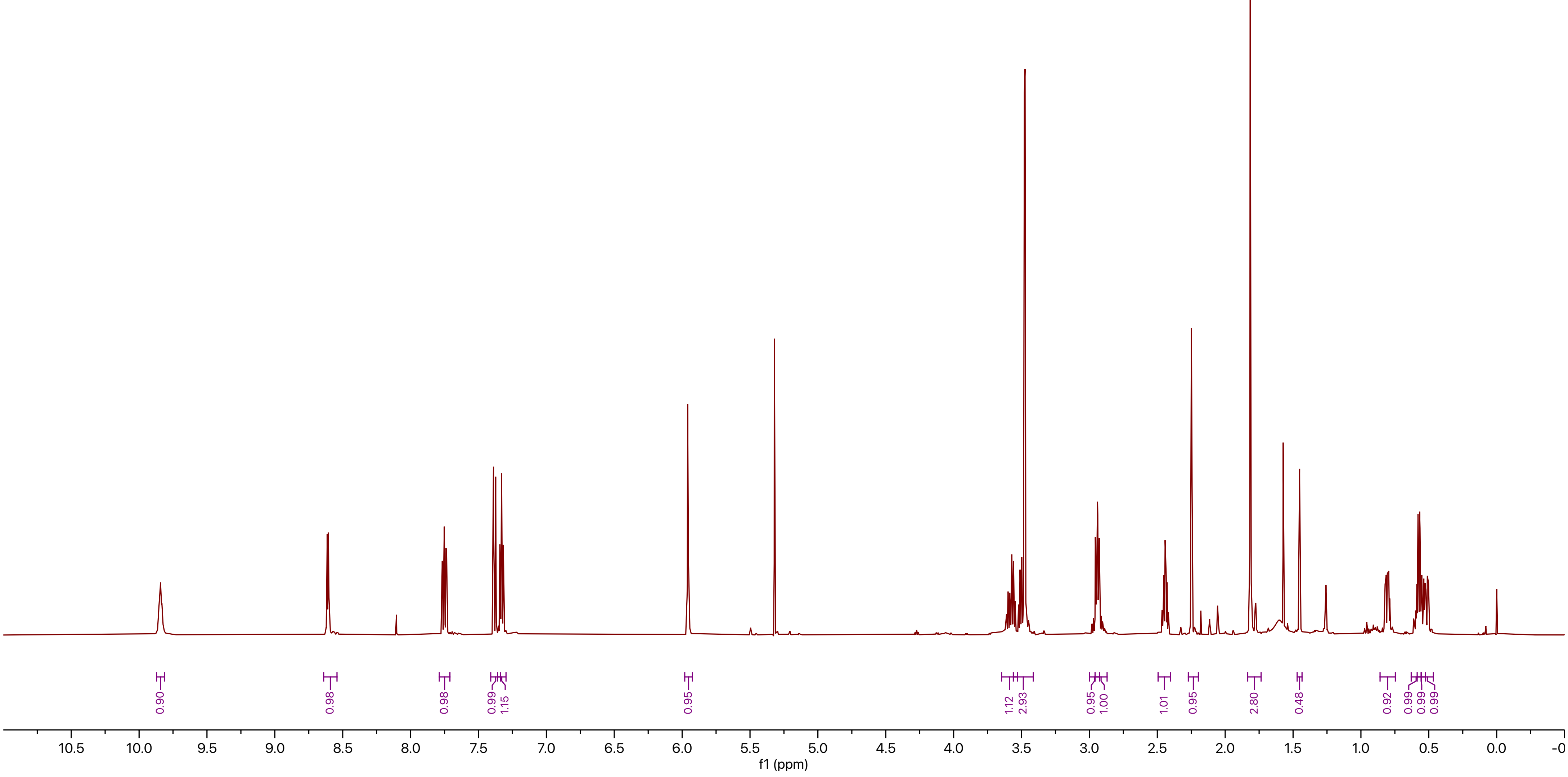

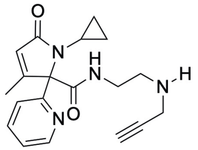

(±)-1-cyclopropyl-3-methyl-5-oxo-*N*-(2-(prop-2-yn-1-ylamino)ethyl)-2-(pyridin-2-yl)-2,5-dihydro-1*H*-pyrrole-2-carboxamide
((±)-PJDU-213)
^{13}C NMR (126 MHz, $CDCl_3$ with TMS)

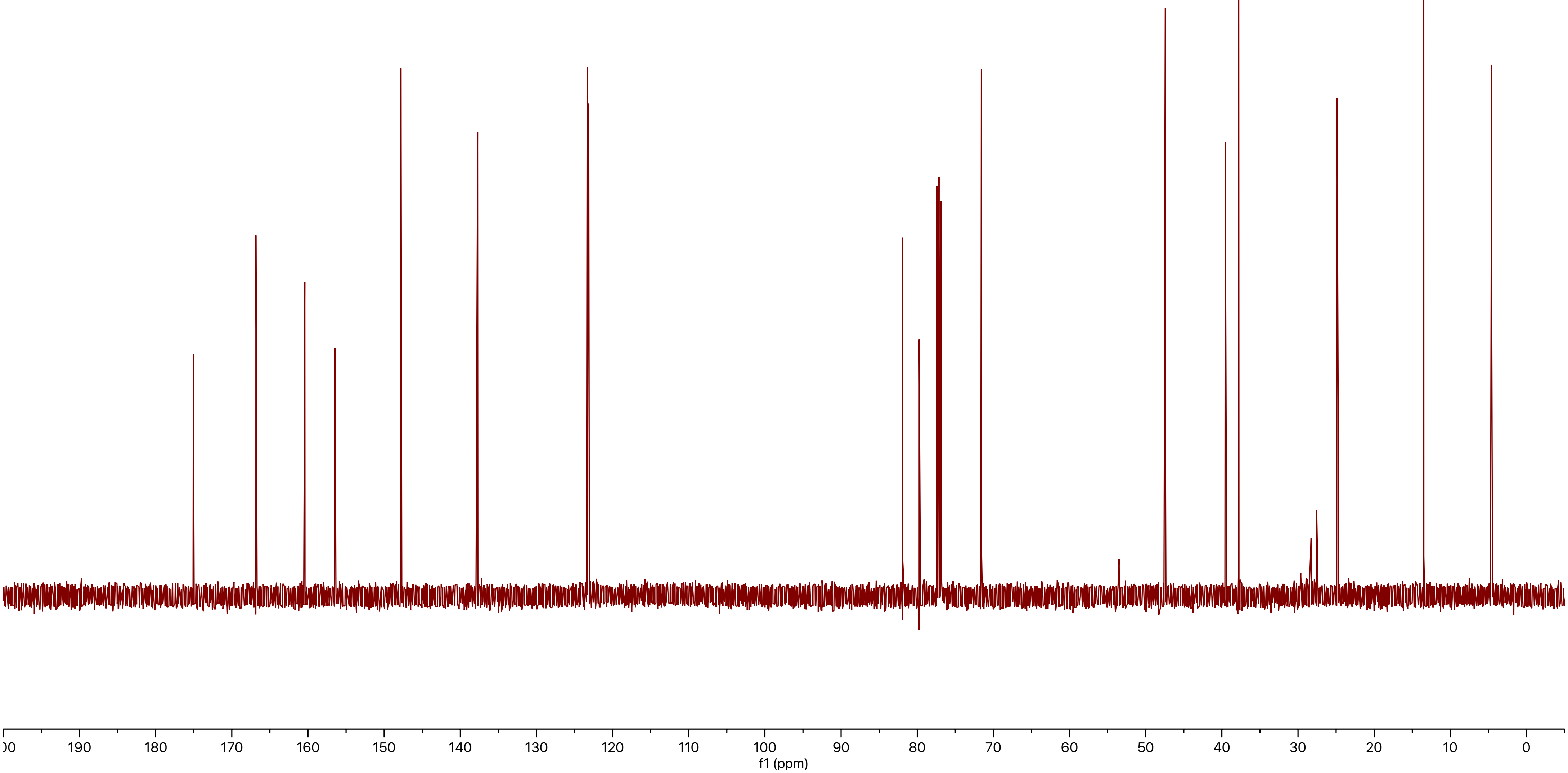

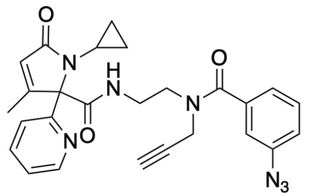

(±)-**4o**
((±)-PJDU-214)
^{1}H NMR (400 MHz, $CDCl_3$ with TMS)

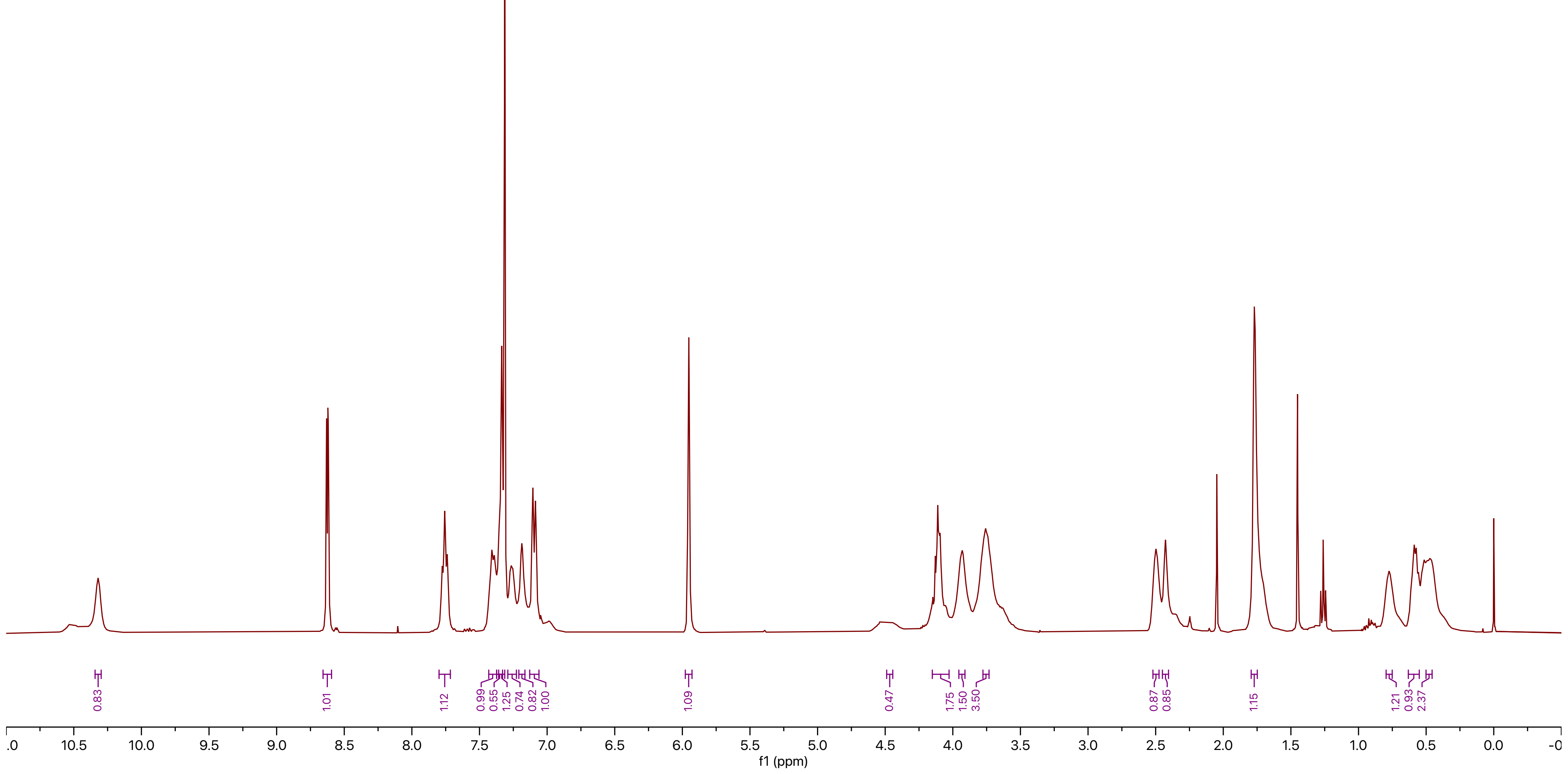

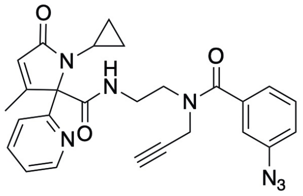

(±)-**4o**
((±)-PJDU-214)
^{13}C NMR (101 MHz, $CDCl_3$ with TMS)

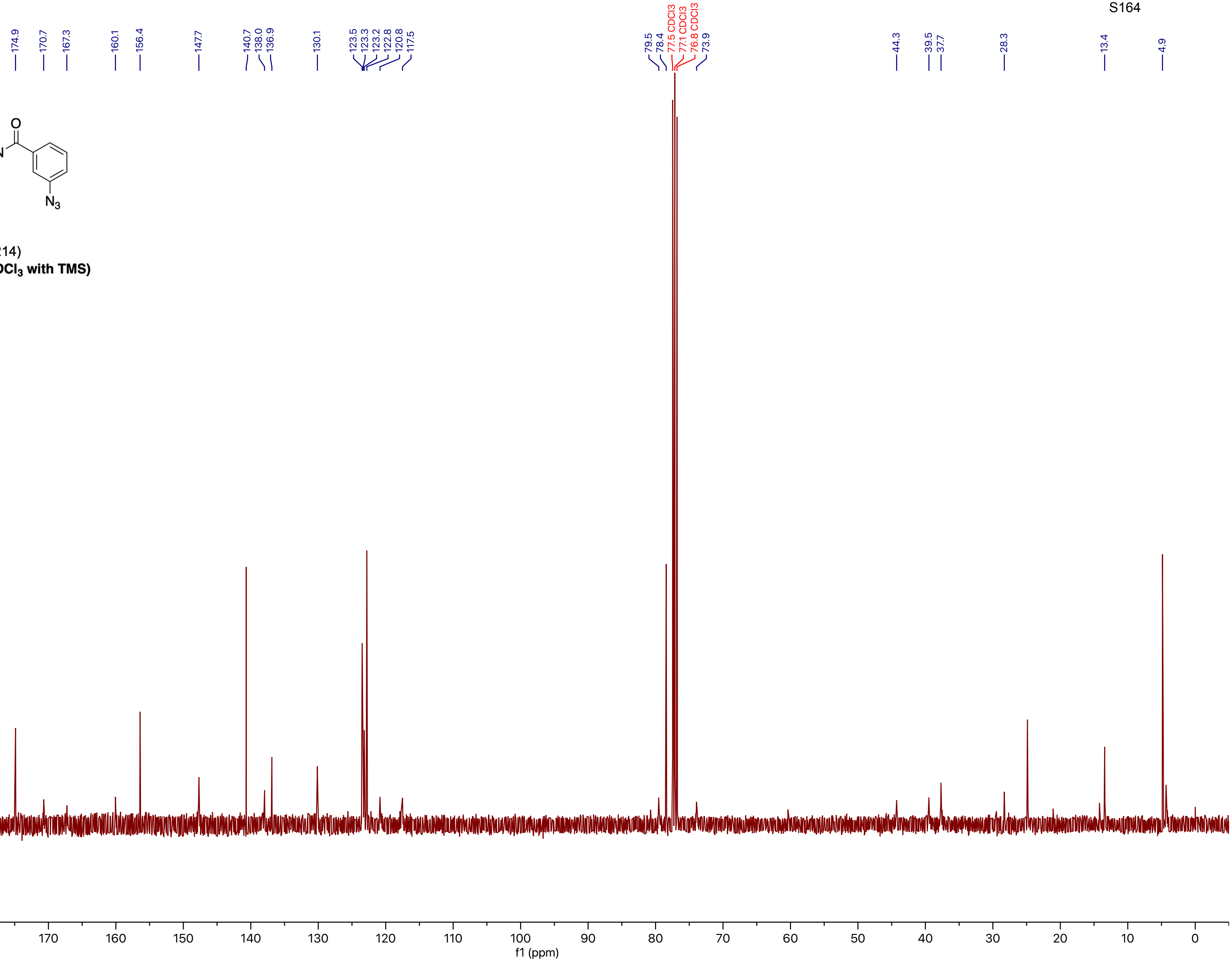

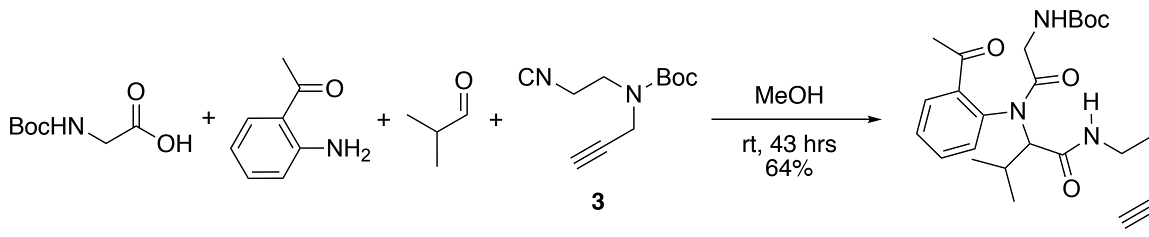

(±)-**14**
((±)-PJDU-218)

*(±)-tert-Butyl (8-(2-acetylphenyl)-9-isopropyl-2,2-dimethyl-4,7,10-trioxo-3-oxa-5,8,11-triazatridecan-13-yl)(prop-2-yn-1-yl)carbamate ((±)-**14**) ((±)-PJDU-218).* A flame-dried 10 mL round-bottom flask equipped with a stir-bar was charged sequentially with 2-aminoacetophenone (0.18 mL, 1.47 mmol, 1.1 eq.), isobutyraldehyde (0.09 mL, 1.47 mmol, 1.1 eq.) and MeOH (1 mL). The resulting mixture was stirred for 30 minutes at room temperature to form the presumed imine, then (*tert*-butoxycarbonyl)glycine (258 mg, 1.47 mmol, 1.1 eq.) was added in a single portion, followed immediately by addition of a solution of isocyanide **3** (280 mg, 1.34 mmol, 1.0 eq.) in MeOH (2 mL) via cannula. Following the addition, the reaction mixture was stirred at room temperature for 43 hours, wherein TLC analysis indicated a complete reaction. Next, the crude reaction mixture was concentrated to dryness and then dissolved with EtOAc (75 mL). The organic layer was then washed sequentially with sat. aq. $NaHCO_3$ (15 mL), 1M aq. HCl (15 mL), H_2O (15 mL) and brine (15 mL), then dried ($MgSO_4$), filtered, and concentrated under reduced pressure. The resulting crude residue was purified by silica gel flash column chromatography (gradient elution with 50-70% EtOAc/hexanes) to give amide (±)-**14** (493 mg, 64%, (±)-PJDU-218) as a white, sticky solid.

R_f = 0.20 (50% EtOAc/hexanes)

^{1}H NMR (400 MHz, $CDCl_3$, *peaks reported are for the major rotamer only) δ 7.80 – 7.69 (m, 2H), 7.52 (dp, *J* = 25.4, 7.8 Hz, 2H), 7.38 – 7.28 (m, 1H), 5.58 (s, 1H), 4.83 (d, *J* = 10.8 Hz, 1H), 4.17 – 4.07 (m, 2H), 4.04 (s, 1H), 3.65 (dt, *J* = 17.0, 8.8 Hz, 1H), 3.56 – 3.43 (m, 3H), 3.42 – 3.31 (m, 1H), 3.16 (s, 1H), 2.89 (s, 0H), 2.59 – 2.54 (m, 3H), 2.33 – 2.24 (m, 1H), 1.80 (dq, *J* = 17.4, 6.3 Hz, 1H), 1.50 (d, *J* = 10.1 Hz, 9H), 1.40 (d, *J* = 8.0 Hz, 9H), 1.09 (d, *J* = 6.5 Hz, 3H), 0.86 (t, *J* = 7.2 Hz, 3H)

^{13}C NMR (101 MHz, $CDCl_3$, *peaks reported are for the major rotamer only) δ 198.7, 171.0, 168.6, 155.6, 155.0, 133.5, 132.3, 131.3, 130.0, 129.3, 129.1, 80.6, 79.8, 79.2, 71.9, 63.9, 45.9, 44.0, 37.8, 37.6, 37.0, 29.0, 28.3, 26.7, 19.9, 18.9

HRMS (ESI+) *m/z* [M + Na] calcd for $C_{30}H_{44}N_4O_7Na$ 595.3102, found 595.3109

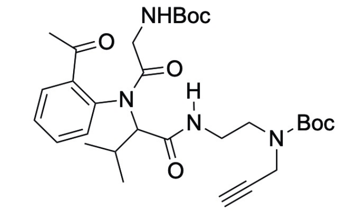

(±)-**14**

((±)-PJDU218)

^{1}H NMR (400 MHz, $CDCl_3$ with TMS)

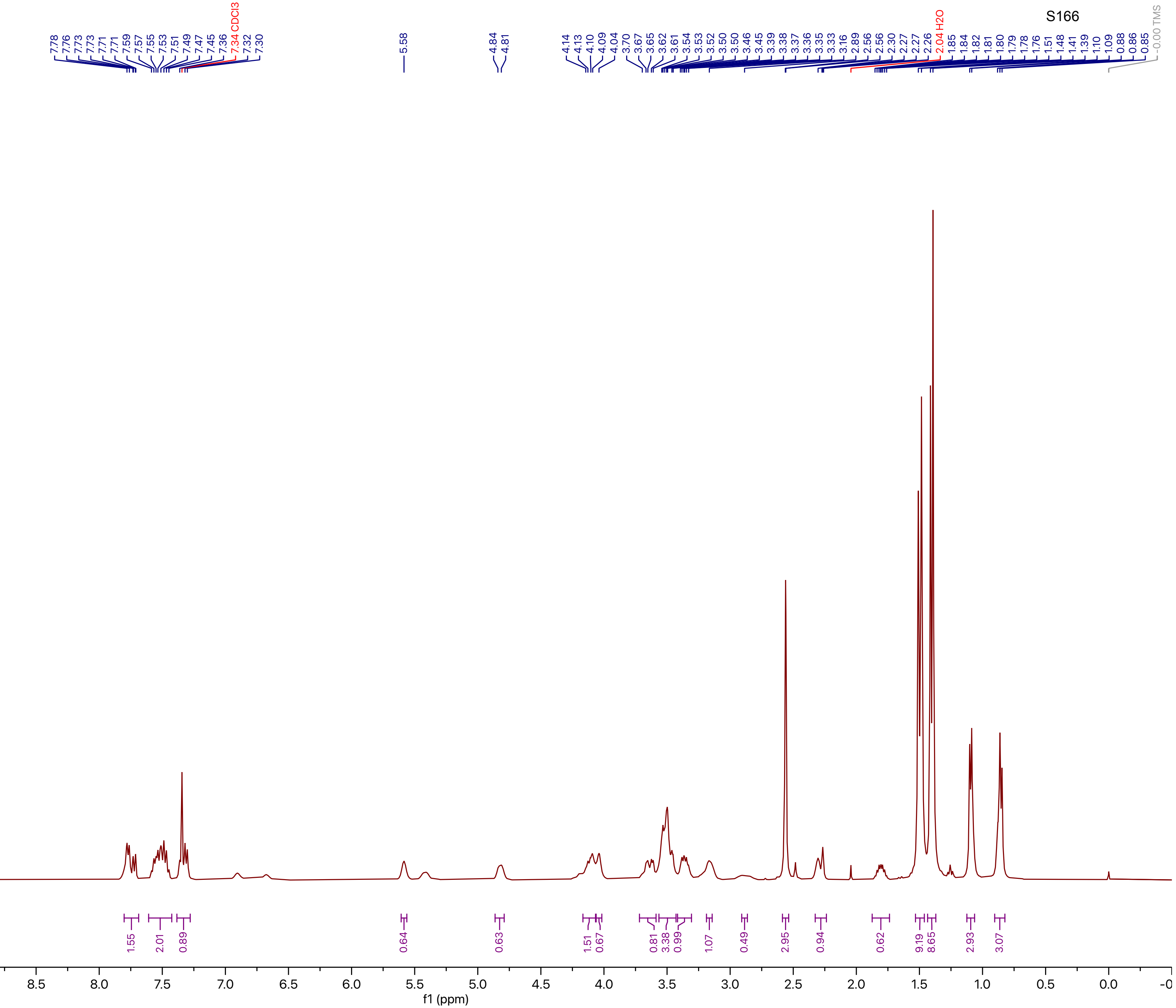

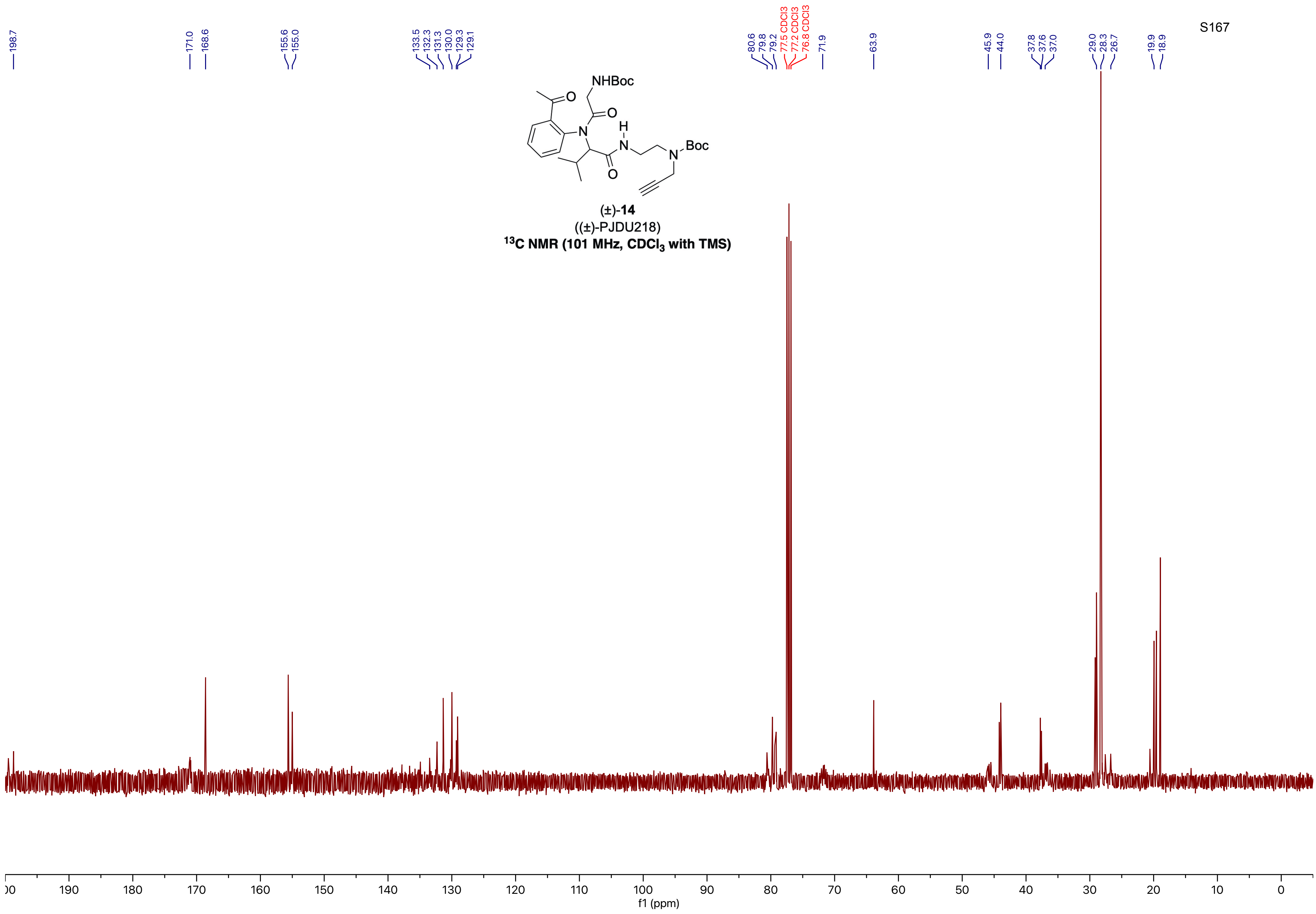
198.7
171.0
168.6
155.6
155.0
133.5
132.3
131.3
130.0
129.3
129.1
80.6
79.8
79.2
77.5 CDCl3
77.2 CDCl3
76.8 CDCl3
71.9
63.9
45.9
44.0
37.8
37.6
37.0
29.0
28.3
26.7
19.9
18.9
NHBoc
O
O
N
H
N
N
Boc
O
(±)-14
((±)-PJDU218)
13C NMR (101 MHz, CDCl3 with TMS)
f1 (ppm)

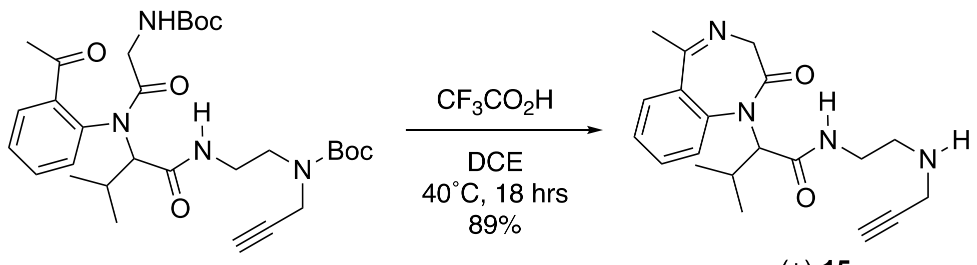

(±)-**14**
((±)-PJDU-218)

(±)-**15**
((±)-PJDU-221)

*(±)-3-Methyl-2-(5-methyl-2-oxo-2,3-dihydro-1H-benzo[e][1,4]diazepin-1-yl)-N-(2-(prop-2-yn-1-ylamino)ethyl)butanamide ((±)-**15**) ((±)-PJDU-221).* A flame-dried, 10 mL round-bottom flask equipped with a stir-bar, septum, and argon inlet needle was charged with dicarbamate (±)-**14** (270 mg, 0.47 mmol, 1.0 eq., (±)-PJDU-218) and 1,2-dichloroethane (3 mL). Next, trifluoracetic acid (0.36 mL, 4.7 mmol, 10 eq.) was added dropwise over 1 minute and then the reaction mixture was heated to 40 °C and stirred for 18 hours, wherein TLC analysis indicated a complete reaction. The reaction mixture was subsequently quenched by the addition of sat. aq. sodium $NaHCO_3$ (12 mL) dropwise over 5 minutes, wherein vigorous gas evolution was observed. Next, 1 M aq. NaOH (5 mL) was added to bring the pH up to 8-9 (checked with pH paper), and then the biphasic mixture was transferred to a separatory funnel and diluted with CH_2Cl_2 (20 mL). The layers were separated and the aqueous layer was extracted with CH_2Cl_2 (2 x 20 mL). The combined organic layers were then washed with brine (10 mL), dried($MgSO_4$), filtered, and concentrated under reduced pressure to give benzodiazepine (±)-**15** (148 mg, 89%, (±)-PJDU-218) as a light yellow solid, which proved sufficiently pure to be used in the subsequent N-acylation arming step.

R_f = 0.42 (5% MeOH/ CH_2Cl_2)

^{1}H NMR (500 MHz, $CDCl_3$, *peaks reported are for the major rotamer only) δ 8.14-8.12 (m, 1H), 7.53-7.46 (m, 2H), 7.33-7.29 (m, 1H), 7.27 (t, J = 5.5 Hz, 1H), 4.53 (d, J = 11.2 Hz, 1H), 3.66-3.63 (m, 2H), 3.45-3.34 (m, 3H), 2.89-2.88 (m, 2H), 2.46 (d, J = 1.5 Hz, 3H), 2.19 (t, J = 2.5 Hz, 1H), 2.18-2.14 (m, 1H), 1.66 (bs, 1H), 0.96 (d, J = 6.5 Hz, 3H), 0.42 (d, J = 6.5 Hz, 3H)

^{13}C NMR (101 MHz, $CDCl_3$, *peaks reported are for the major rotamer only) δ 172.5, 170.9, 170.3, 138.3, 131.7, 130.6, 127.1, 125.7, 124.6, 82.0, 71.5, 64.8, 56.4, 47.3, 43.5, 39.1, 37.7, 25.1, 20.1, 18.4

HRMS (ESI+) m/z [M + Na] calcd for $C_{20}H_{26}N_4O_2Na$ 377.1948, found 377.1949

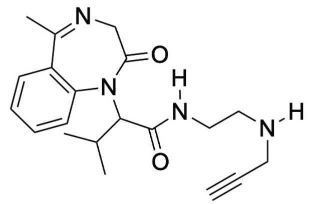

(±)-**15**
((±)-PJDU-221)
^{1}H NMR (500 MHz, $CDCl_3$ with TMS)

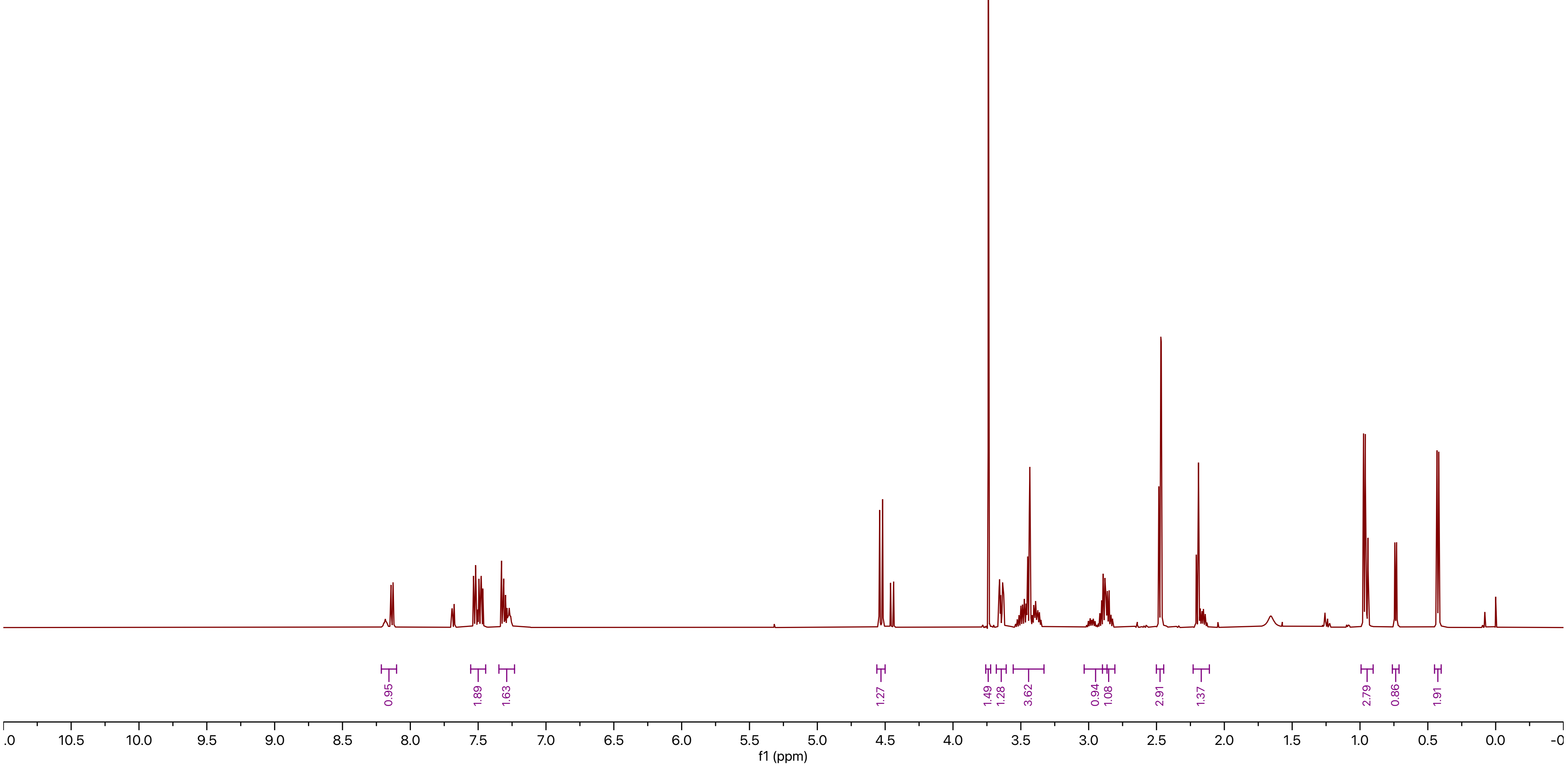

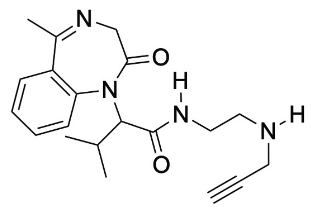

(±)-**15**
((±)-PJDU-221)
^{13}C NMR (126 MHz, $CDCl_3$ with TMS)

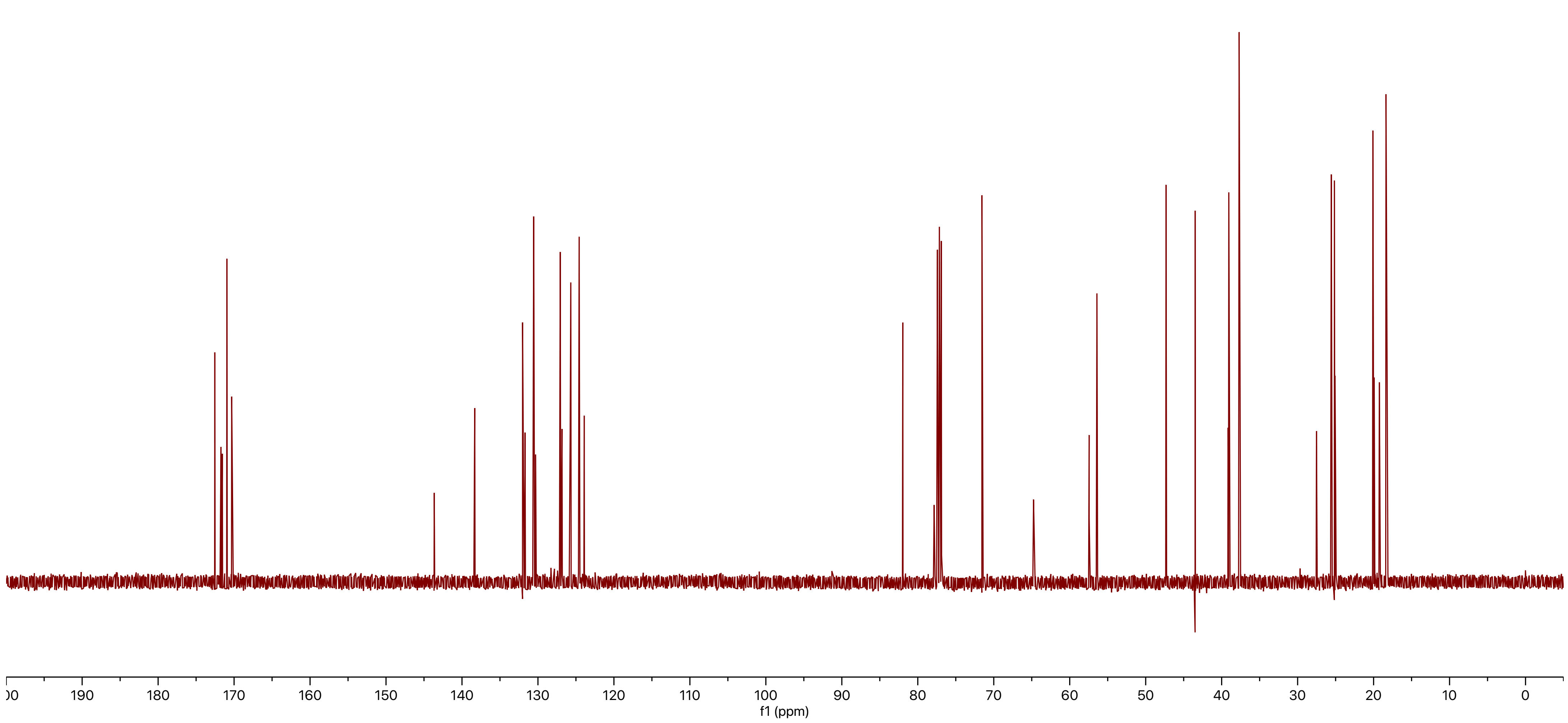

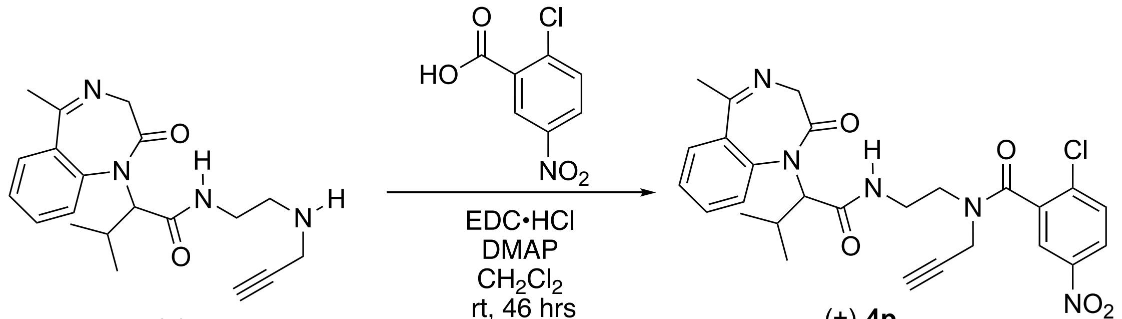

(±)-**15**
((±)-PJDU-221)

(±)-**4p**
((±)-PJDU-223)

*(±)-2-Chloro-N-(2-(3-methyl-2-(5-methyl-2-oxo-2,3-dihydro-1H-benzo[e][1,4]diazepin-1-yl)butanamido)ethyl)-5-nitro-N-(prop-2-yn-1-yl)benzamide ((±)-**4p**) ((±)-PJDU-223).* A 25 mL flame-dried round-bottom flask equipped with a stir-bar, septum, and argon inlet needle was charged with 2-chloro-5-nitrobenzoic acid (124 mg, 0.61 mmol, 1.5 eq.) and CH_2Cl_2 (3 mL), then EDC•HCl (157 mg, 0.82 mmol, 2 eq.) and DMAP (5 mg, 0.04 mmol, 0.1 eq.) were added sequentially. Next a solution of propargyl amine (±)-**15** (145 mg, 0.41 mmol, 1.0 eq., (±)-PJDU-221) in CH_2Cl_2 (5.2 mL) was added via cannula. The reaction mixture was stirred at room temperature for 46 hours, wherein TLC analysis indicated a complete reaction. Next, the reaction mixture was concentrated and then transferred to a separatory funnel with EtOAc (50 mL). The organic layers were then washed sequentially with H_2O (10 mL), sat. aq. $NaHCO_3$ (10 mL), H_2O (10 mL), and brine (10 mL) then dried ($MgSO_4$), filtered, and concentrated under reduced pressure. The crude residue was then purified by silica gel flash column chromatography (elution with 75% EtOAc/hexanes) to give FFSMP (±)-**4p** (124 mg, 56%; (±)-PJDU-223) as a white foamy solid.

R_f = 0.17 (75% EtOAc/hexanes)

^{1}H NMR (500 MHz, $CDCl_3$, *peaks reported are for the major rotamer only) δ 8.46-8.31 (m, 1H), 8.20-8.16 (m, 2H), 7.60-7.56 (m, 1H), 7.48-7.40 (m, 4H), 4.50 (d, *J* = 11.5 Hz, 1H), 4.43-4.35 (m, 2H), 4.01-3.88 (m, 3H), 3.67-3.45 (m, 3H), 2.42-2.40 (m, 3H), 2.36-2.32 (m, 1H), 2.17-2.11 (m, 1H), 0.93 (d, *J* = 6.0 Hz, 3H) 0.37 (d, *J* = 6.0 Hz, 3H)

^{13}C NMR (126 MHz, $CDCl_3$, *peaks reported are for the major rotamer only) δ 172.9, 172.0, 171.4, 170.2, 166.3, 146.5, 143.4, 137.4, 132.0, 130.6, 130.3, 127.1, 125.8, 125.3, 124.3, 123.6, 77.6, 74.4, 57.4, 56.4, 45.1, 37.4, 34.2, 27.5, 25.2, 20.2, 18.3

HRMS (ESI+) *m/z* [M + Na] calcd for $C_{26}H_{25}N_7O_3Na$ 506.1911, found 506.1915

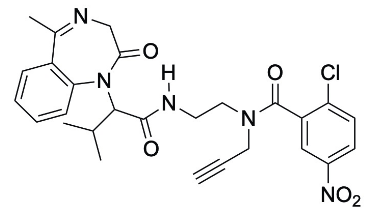

(±)-**4p**
((±)-PJDU-223)
^{1}H NMR (500 MHz, $CDCl_3$ with TMS)

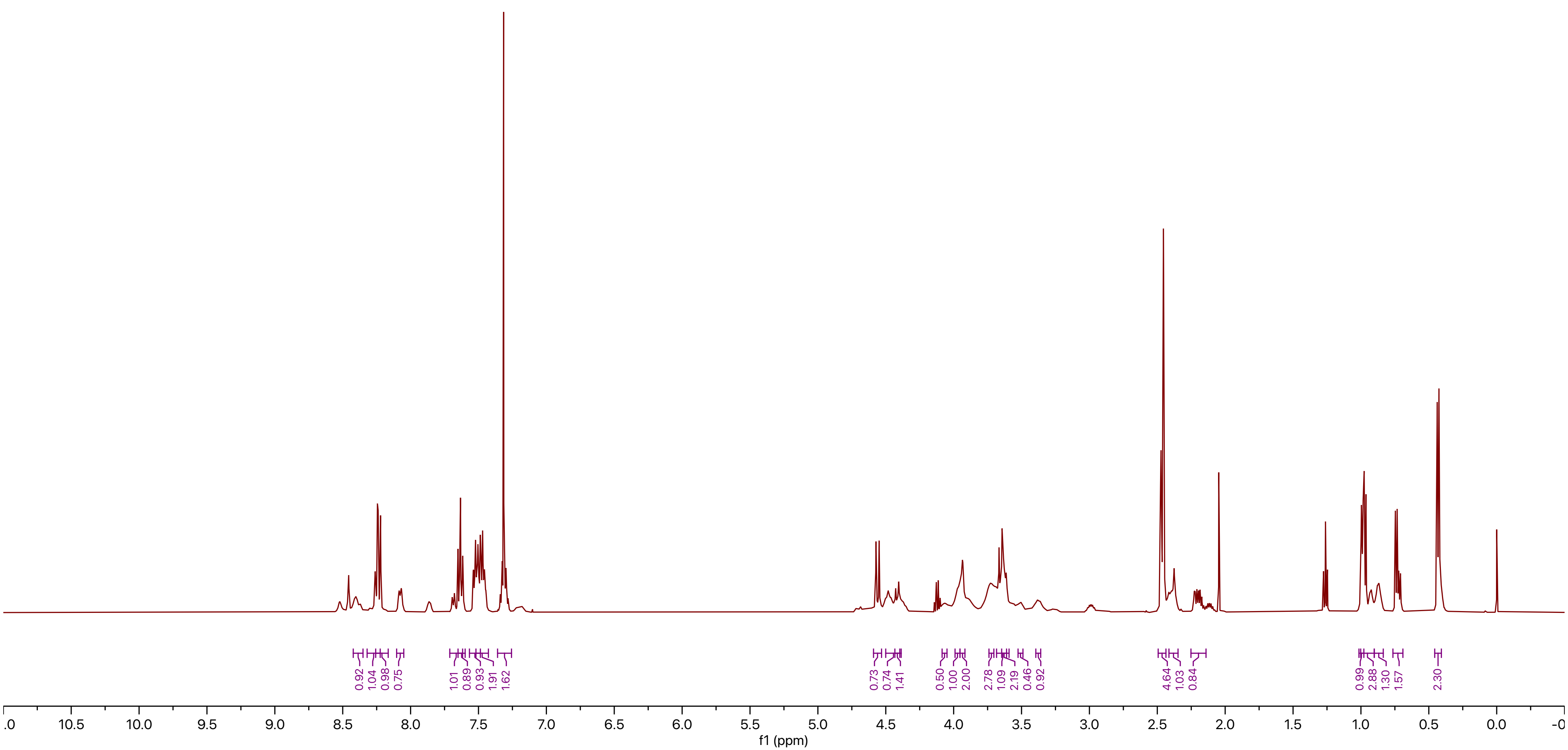

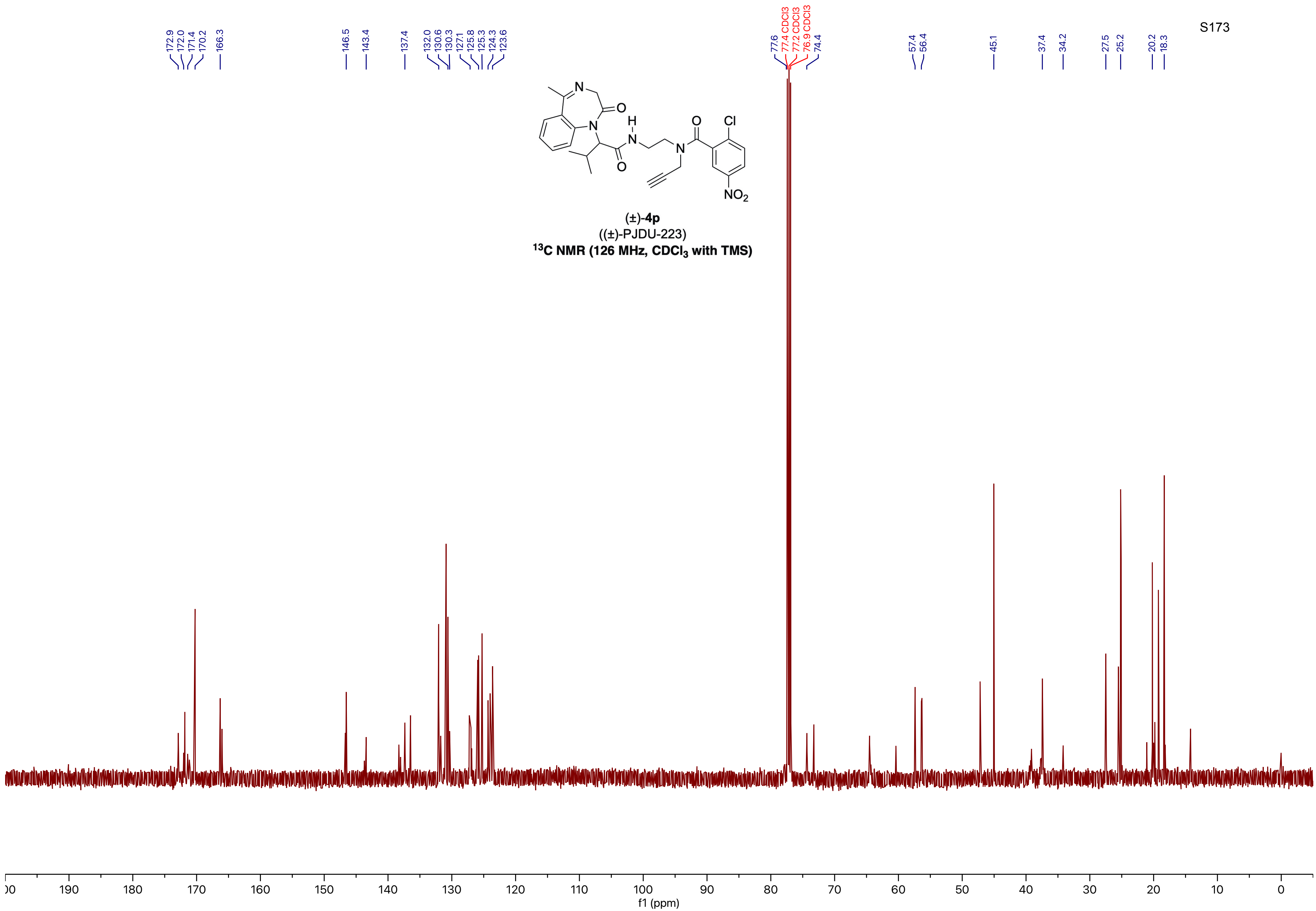
172.9
172.0
171.4
170.2
166.3
146.5
143.4
137.4
132.0
130.6
130.3
127.1
125.8
125.3
124.3
123.6
77.6
77.4 CDCl3
77.2 CDCl3
76.9 CDCl3
74.4
57.4
56.4
45.1
37.4
34.2
27.5
25.2
20.2
18.3
N
O
H
N
N
O
Cl
O
NO2
(±)-4p
((±)-PJDU-223)
13C NMR (126 MHz, CDCl3 with TMS)
190
180
170
160
150
140
130
120
110
100
90
80
70
60
50
40
30
20
10
0
f1 (ppm)

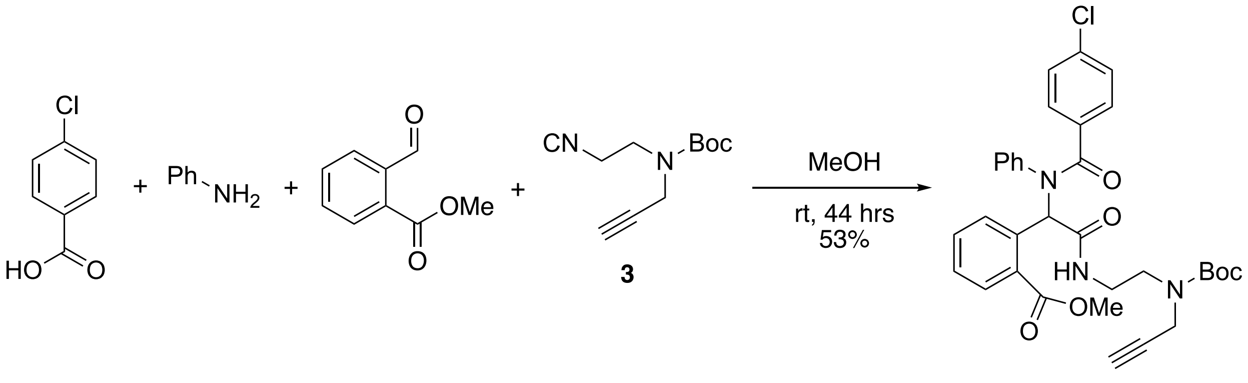

(±)-**16**
((±)-PJDU-207)

*(±)-Methyl 2-(1-(4-chlorophenyl)-11,11-dimethyl-1,4,9-trioxo-2-phenyl-8-(prop-2-yn-1-yl)-10-oxa-2,5,8-triazadodecan-3-yl)benzoate ((±)-**16**) ((±)-PJDU-207).* A flame-dried 25 mL round-bottom flask equipped with a stir-bar was charged sequentially with methyl 2-formylbenzoate (190 mg, 1.16 mmol, 1.1 eq.), aniline (0.11 mL, 1.16 mmol, 1.1 eq.) and MeOH (3 mL). The resulting mixture was stirred for 30 minutes at room temperature to form the presumed imine, then 4-chlorobenzoic acid (181 mg, 1.16 mmol, 1.1 eq.) was added in a single portion, followed immediately by addition of a solution of isocyanide **3** (230 mg, 1.1 mmol, 1.0 eq.) in MeOH (8 mL) via cannula. Following the addition, the reaction mixture was stirred at room temperature for 44 hours, wherein TLC analysis indicated a complete reaction. Next, the crude reaction mixture was concentrated to dryness and then dissolved with EtOAc (75 mL). The organic layer was then washed sequentially with sat. aq. $NaHCO_3$ (10 mL), 1M aq. HCl (10 mL), H_2O (10 mL), and brine (10 mL), then dried ($MgSO_4$), filtered, and concentrated under reduced pressure. The resulting crude residue was purified by silica gel flash column chromatography (gradient elution with 25-45% EtOAc/hexanes) to give amide (±)-**16** (350 mg, 53%, (±)-PJDU-207) as a yellow, sticky solid.

R_f = 0.49 (50% EtOAc/hexanes)

^{1}H NMR (400 MHz, $CDCl_3$, *peaks reported are for the major rotamer only) δ 7.82 (dd, *J* = 6.9, 3.0 Hz, 1H), 7.25 (d, *J* = 2.6 Hz, 5H), 7.10 – 7.00 (m, 4H), 6.97 – 6.90 (m, 3H), 3.97 (t, *J* = 3.0 Hz, 2H), 3.90 (s, 3H), 3.54 (s, 2H), 3.49 – 3.38 (m, 3H), 2.19 (d, *J* = 10.7 Hz, 1H), 1.39 (s, 9H)

^{13}C NMR (101 MHz, $CDCl_3$, *peaks reported are for the major rotamer only) δ 170.2, 169.5, 167.3, 155.4, 140.4, 135.0, 134.6, 131.7, 131.2, 131.1, 130.5, 130.3, 129.8, 128.4, 128.3, 128.1, 127.7, 127.1, 80.5, 79.7, 71.5, 62.7, 52.4, 46.3, 39.1, 37.5, 28.1

HRMS (ESI+) *m/z* [M + Na] calcd for $C_{33}H_{34}ClN_3O_6Na$ 626.2028, found 626.2030

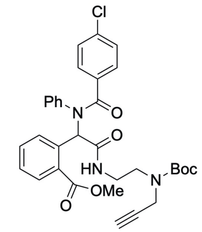

(±)-**16**
((±)-PJDU-207)
^{1}H NMR (400 MHz, $CDCl_3$ with TMS)

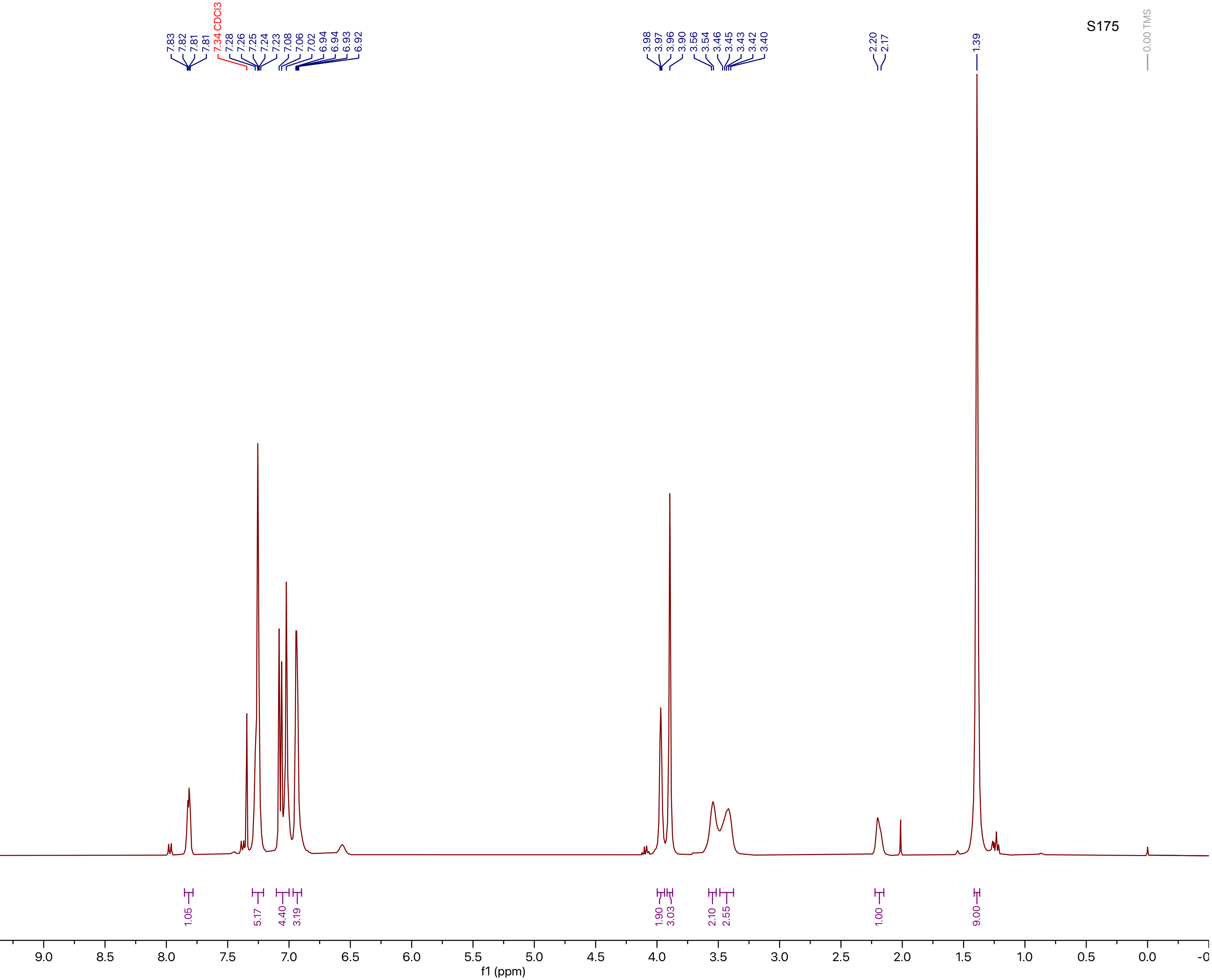

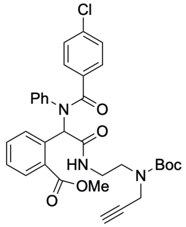

(±)-**16**
((±)-PJDU-207)
^{13}C NMR (101 MHz, $CDCl_3$ with TMS)

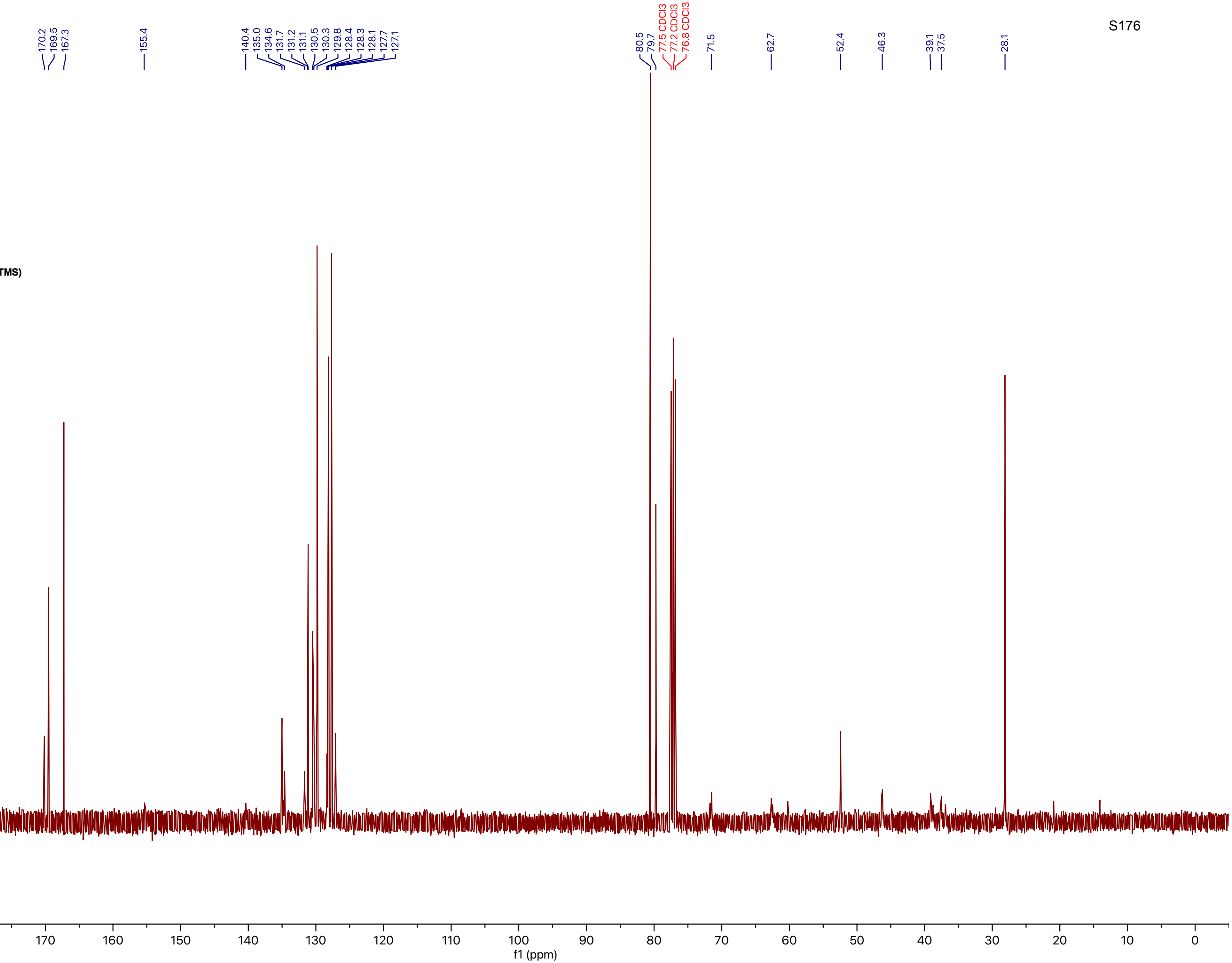

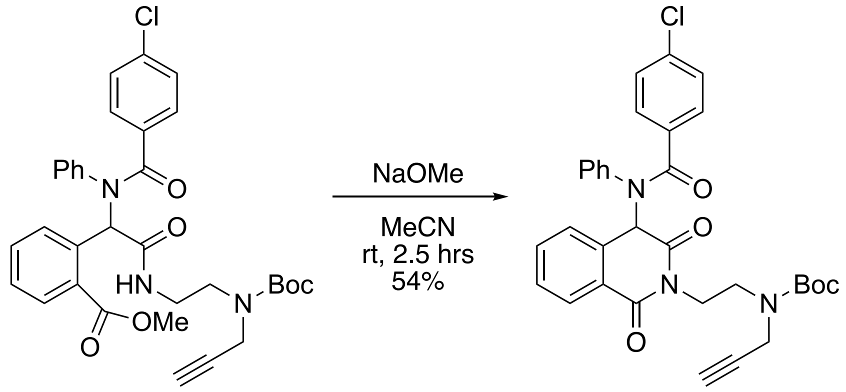

(±)-**16**
((±)-PJDU-207)

(±)-**17**
((±)-PJDU-208)

*(±)-tert-Butyl (2-(4-(4-chloro-N-phenylbenzamido)-1,3-dioxo-3,4-dihydroisoquinolin-2(1H)-yl)ethyl)(prop-2-yn-1-yl)carbamate ((±)-**17**) ((±)-PJDU-208).* A flame-dried, 25 mL round-bottom flask equipped with a stir-bar, septum, and argon inlet needle was charged with ester (±)-**16** (350 mg, 0.58 mmol, 1.0 eq., (±)-PJDU-207) and MeCN (3 mL). Next, NaOMe (110 mg, 2.0 mmol, 3.4 eq.) was added in a single portion. The reaction mixture was stirred for 30 minutes at room temperature, then additional NaOMe was added (30 mg, 0.58 mmol, 1 eq.) was added and the reaction was stirred at 2 hours at room temperature, wherein TLC analysis indicated a complete reaction. Next, the crude reaction mixture was concentrated under reduced pressure and purified by silica gel flash column chromatograph (gradient elution with 25-50% EtOAc/hexanes) to provide isoquinoline dione (±)-**17** (180 mg, 54%, (±)-PJDU-208) as a beige foamy solid.

R_f = 0.74 (50% EtOAc/hexanes)

^{1}H NMR (400 MHz, $CDCl_3$, *peaks reported are for the major rotamer only) δ 8.25 (dd, *J* = 11.3, 7.5 Hz, 1H), 7.65 (dd, *J* = 13.4, 7.7 Hz, 2H), 7.49 (dq, *J* = 15.0, 7.6, 6.8 Hz, 1H), 7.38 – 7.27 (m, 4H), 7.25 (d, *J* = 15.0 Hz, 3H), 7.14 (d, *J* = 8.6 Hz, 2H), 5.86 – 5.71 (m, 1H), 4.22 (tt, *J* = 10.4, 3.8 Hz, 1H), 4.10 (dd, *J* = 18.7, 13.3 Hz, 1H), 3.60 (dq, *J* = 14.1, 7.7, 7.2 Hz, 1H), 2.20 (app s, 1H), 1.38 (s, 4H), 1.26 (s, 1H), 1.21 (s, 4H)

^{13}C NMR (101 MHz, $CDCl_3$, *peaks reported are for the major rotamer only) δ 169.4, 168.8, 164.0, 155.1, 136.6, 134.0, 132.8, 130.5, 129.7, 129.4, 128.6, 128.3, 128.2, 127.7, 127.4, 125.2, 124.7, 124.3, 80.2, 79.6, 71.8, 62.8, 43.4, 38.4, 36.7, 28.0

HRMS (ESI+) *m*/*z* [M + Na] calcd for $C_{32}H_{30}ClN_3O_5Na$ 594.1766, found 594.1769

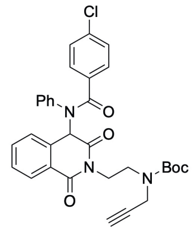

(±)-**17**
((±)-PJDU-208)
^{1}H NMR (400 MHz, $CDCl_3$ with TMS)

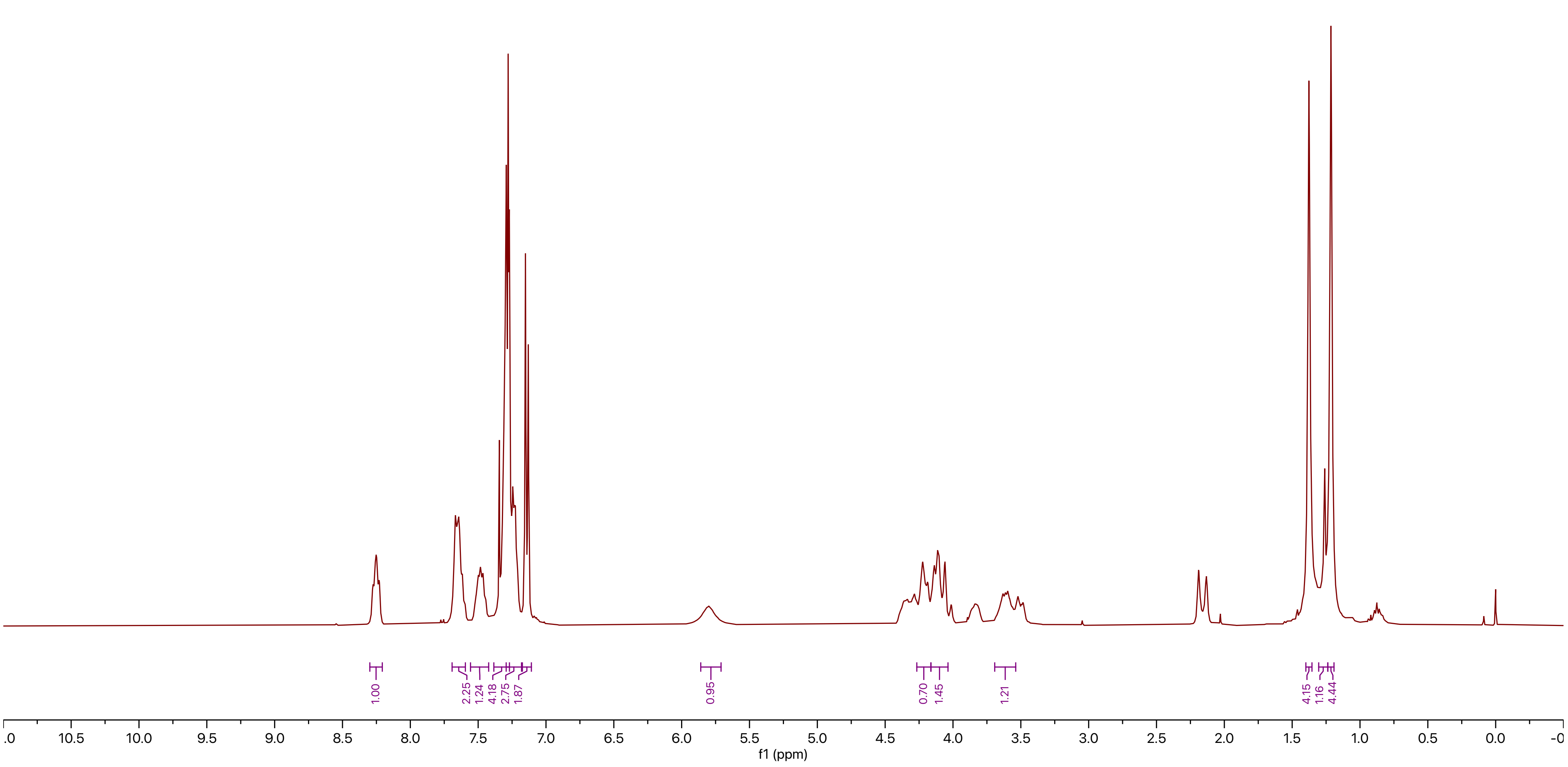

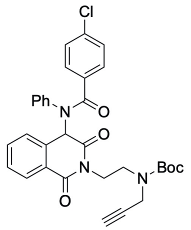

(±)-**17**
((±)-PJDU-208)
^{13}C NMR (101 MHz, $CDCl_3$ with TMS)

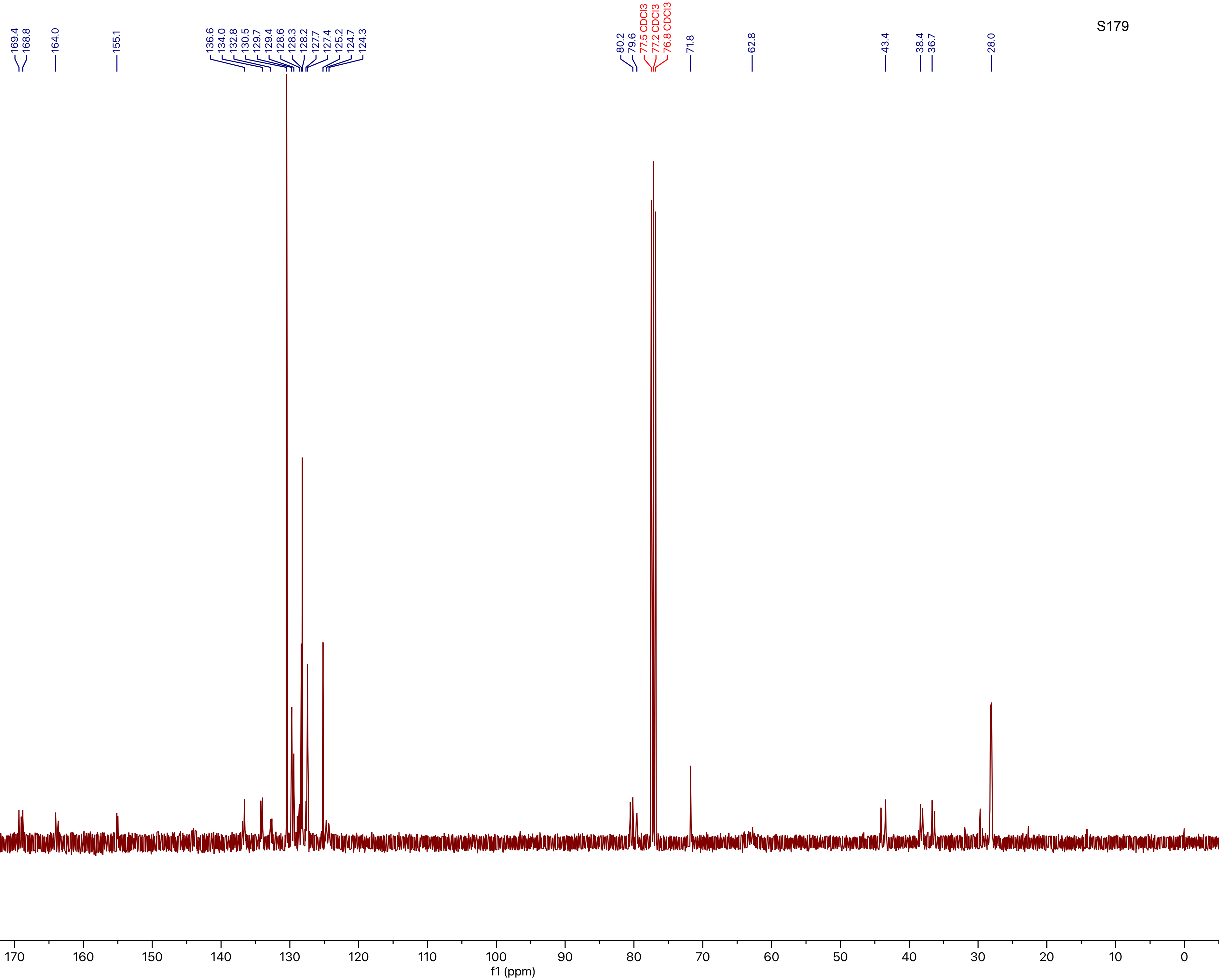

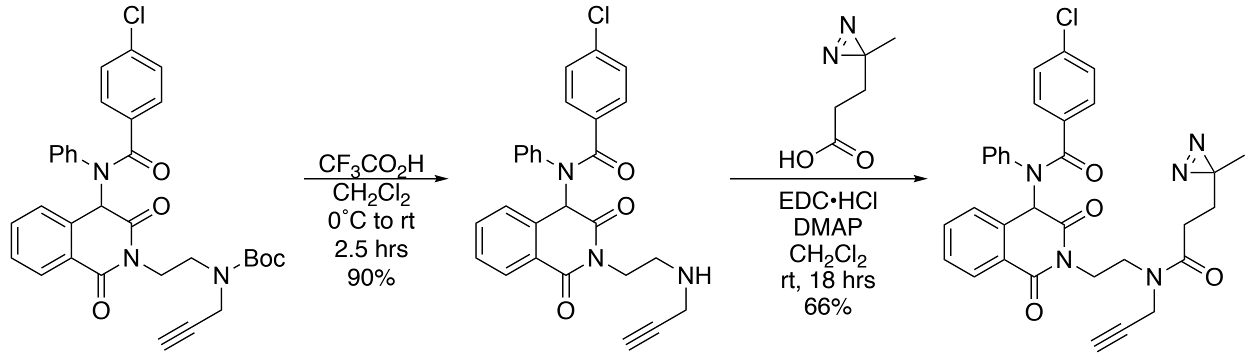

(±)-**17**
((±)-PJDU-208)

(±)-4-chloro-*N*-(1,3-dioxo-2-(2-(prop-2-yn-1-ylamino)ethyl)-1,2,3,4-tetrahydroisoquinolin-4-yl)-*N*-phenylbenzamide
((±)-PJDU-209)

(±)-**4q**
((±)-PJDU-212)
59% over two steps

(±)-4-Chloro-N-(1,3-dioxo-2-(2-(prop-2-yn-1-ylamino)ethyl)-1,2,3,4-tetrahydroisoquinolin-4-yl)-N-phenylbenzamide ((±)-PJDU-209). A 25 mL flame-dried, round-bottom flask equipped with a stirbar, septum, and argon inlet needle was charged with Boc carbamate substrate (±)-**17** (177 mg, 0.31 mmol, 1.0 eq.; (±)-PJDU-208) and CH_2Cl_2 (3.1 mL) and cooled to 0˚C. Trifluoroacetic acid (0.28 mL, 3.7 mmol, 12.0 eq.) was then added dropwise over 2 minutes. After the addition, the ice bath was removed and the reaction was stirred at room temperature for 2.5 hours, wherein TLC indicated complete consumption of the starting Boc carbamate substrate. The reaction was then quenched by dropwise addition of sat. aq. $NaHCO_3$ (vigorous gas evolution) to the reaction mixture until gas evolution ceased (10 mL), then 1M aq. NaOH (4 mL) was added to raise the pH to 8-9 (checked with pH paper). This biphasic mixture was then transferred to a separatory funnel and diluted with CH_2Cl_2 (30 mL), the layers were separated, and the aqueous layer was extracted with CH_2Cl_2 (3 x 20 mL). The combined organic layers were washed with brine (10 mL), dried ($MgSO_4$), gravity filtered, and concentrated under reduced pressure to give propargyl amine (±)-PJDU-209 (132 mg, 90%) as a sticky yellow solid, which proved sufficiently pure to take on to the N-acylation arming step.

R_f = 0.09 (5% MeOH/ CH_2Cl_2)

^{1}H NMR (500 MHz, DMSO-d$_6$, *peaks reported are for the major rotamer only) δ 7.79 (dd, *J* = 8.1, 1.5 Hz, 1H), 7.46 – 7.39 (m, 4H), 7.29 – 7.23 (m, 3H), 7.21 – 7.15 (m, 2H), 7.08 (t, *J* = 7.8 Hz, 2H), 6.68 (td, *J* = 7.3, 6.7, 1.1 Hz, 1H), 4.22 (ddd, *J* = 13.6, 6.4, 4.2 Hz, 1H), 4.13 (ddd, *J* = 13.6, 6.6, 4.2 Hz, 1H), 3.93 – 3.80 (m, 2H), 3.68 – 3.63 (m, 1H), 3.06 (dqt, *J* = 14.3, 9.6, 3.8 Hz, 2H)

^{13}C NMR (126 MHz, DMSO-d$_6$, *peaks reported are for the major rotamer only) δ 171.7, 163.1, 157.7, 143.7, 138.9, 137.7, 133.4, 132.0, 128.7, 128.1, 127.8, 127.0, 125.0, 124.6, 117.0, 116.8, 114.9, 99.6, 78.8, 75.8, 46.8, 36.8, 35.8

HRMS (ESI+) *m/z* [M + H] calcd for $C_{27}H_{22}ClN_3O_3H$ 472.1422, found 472.1431

*(±)-4-Chloro-N-(2-(2-(3-(3-methyl-3H-diazirin-3-yl)-N-(prop-2-yn-1-yl)propanamido)ethyl)-1,3-dioxo-1,2,3,4-tetrahydroisoquinolin-4-yl)-N-phenylbenzamide ((±)-**4q**) ((±)-PJDU-212).* A 10 mL flame-dried round-bottom flask equipped with a stir-bar, septum, and argon inlet needle was charged with 3-(3-methyl-3*H*-diazirin-3-yl)propanoic acid (35 mg, 0.27 mmol, 1.5 eq.) and CH_2Cl_2 (1.5 mL), then EDC•HCl (69 mg, 0.36 mmol, 2 eq.) and DMAP (3 mg, 0.02 mmol, 0.1 eq.) were added sequentially. Next a solution of propargyl amine (±)-PJDU-209 (86 mg, 0.18 mmol, 1 eq.) in CH_2Cl_2 (3 mL) was added via cannula. The reaction mixture was stirred at room temperature

for 18 hours, wherein TLC analysis indicated a complete reaction. Next, the reaction mixture was concentrated and then transferred to a separatory funnel with EtOAc (50 mL). The organic layers were then washed sequentially with H_2O (10 mL), sat. aq. $NaHCO_3$ (10 mL), H_2O (10 mL), and brine (10 mL) then dried ($MgSO_4$), filtered, and concentrated under reduced pressure. The crude residue was then purified by silica gel flash column chromatography (elution with 50-70% EtOAc/hexanes) to give FFSMP (±)-**4q** (69 mg, 66%; (±)-PJDU-212) as a light yellow sticky solid.

R_f = 0.49 (75% EtOAc/hexanes)

^{1}H NMR (500 MHz, $CDCl_3$, *peaks reported are for the major rotamer only) δ 8.30-8.22 (m, 1H), 7.70-7.63 (m, 2H), 7.54-7.48 (m, 1H), 7.34-7.27 (m, 7H), 7.17-7.15 (m, 2H), 5.75 (s, 1H), 4.34-4.18 (m, 2H), 4.08-3.94 (m, 2H), 3.66-3.56 (m, 2H), 2.20 (t, *J* = 2.5 Hz, 1H), 2.08-2.05 (m, 2H), 1.77-1.72 (m, 2H), 0.85 (s, 3H)

^{13}C NMR (126 MHz, $CDCl_3$, *peaks reported are for the major rotamer only) δ 171.9, 169.5, 168.8, 164.2, 137.0, 136.8, 134.1, 132.7, 130.5, 129.8, 129.5, 128.4, 128.3, 127.7, 127.5, 124.9, 124.8, 124.2, 78.3, 71.9, 43.8, 38.6, 37.7, 35.0, 29.6, 27.4, 25.3, 19.9

HRMS (ESI+) *m/z* [M + Na] calcd for $C_{32}H_{28}ClN_5O_4Na$ 604.1722, found 604.1722

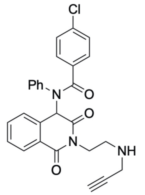

(±)-4-chloro-*N*-(1,3-dioxo-2-(2-(prop-2-yn-1-ylamino)ethyl)-1,2,3,4-tetrahydroisoquinolin-4-yl)-*N*-phenylbenzamide
((±)-PJDU-209)
^{1}H NMR (500 MHz, DMSO-d_6 with TMS)

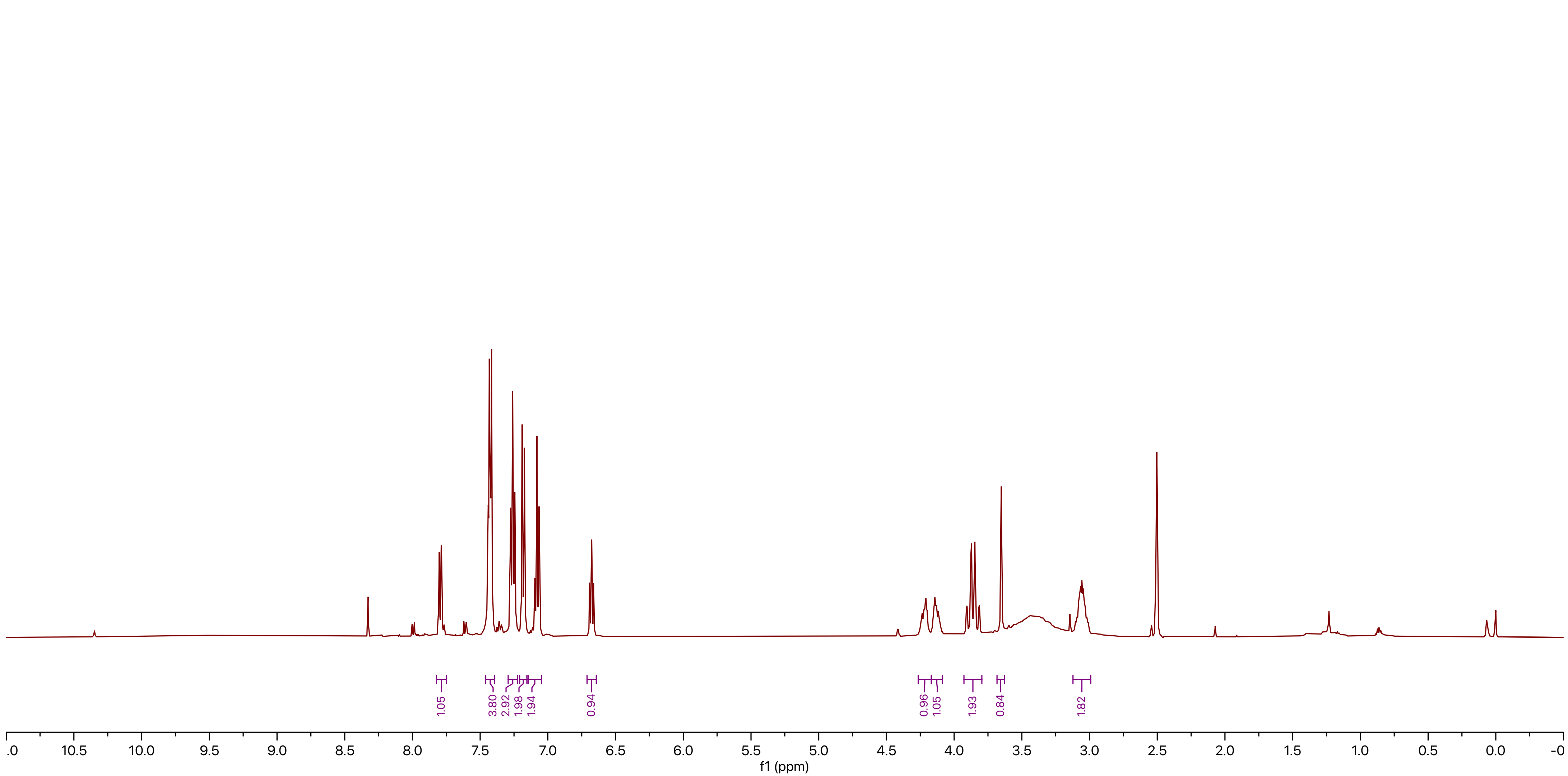

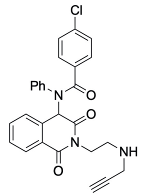

(±)-4-chloro-*N*-(1,3-dioxo-2-(2-(prop-2-yn-1-ylamino)ethyl)-1,2,3,4-tetrahydroisoquinolin-4-yl)-*N*-phenylbenzamide
((±)-PJDU-209)
^{13}C NMR (126 MHz, DMSO-d_6 with TMS)

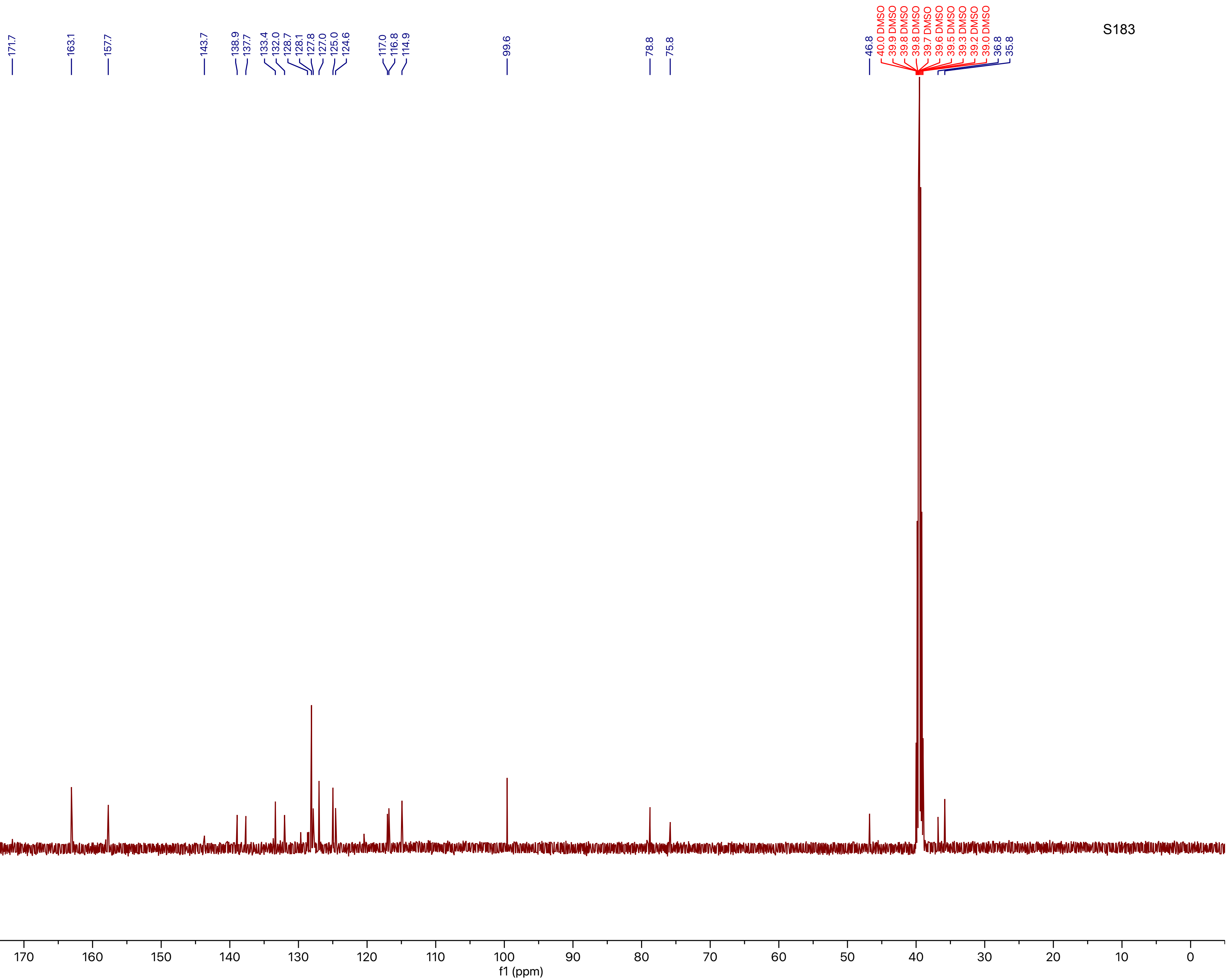

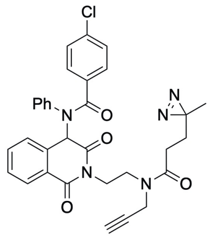

(±)-**4q**
((±)-PJDU-212)
^{1}H NMR (500 MHz, $CDCl_3$ with TMS)

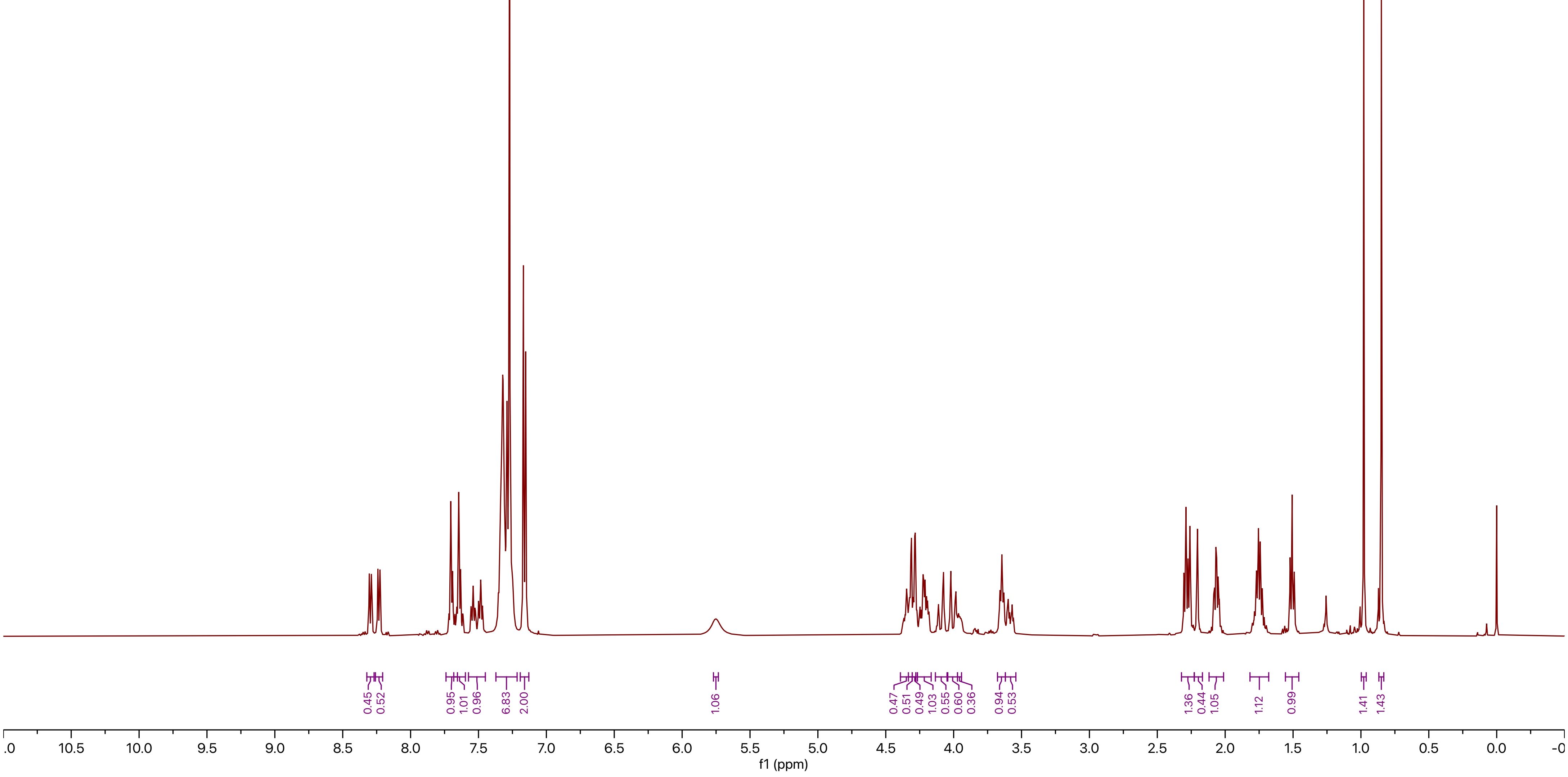

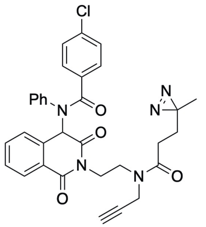

(±)-**4q**
((±)-PJDU-212)
^{13}C NMR (126 MHz, $CDCl_3$ with TMS)

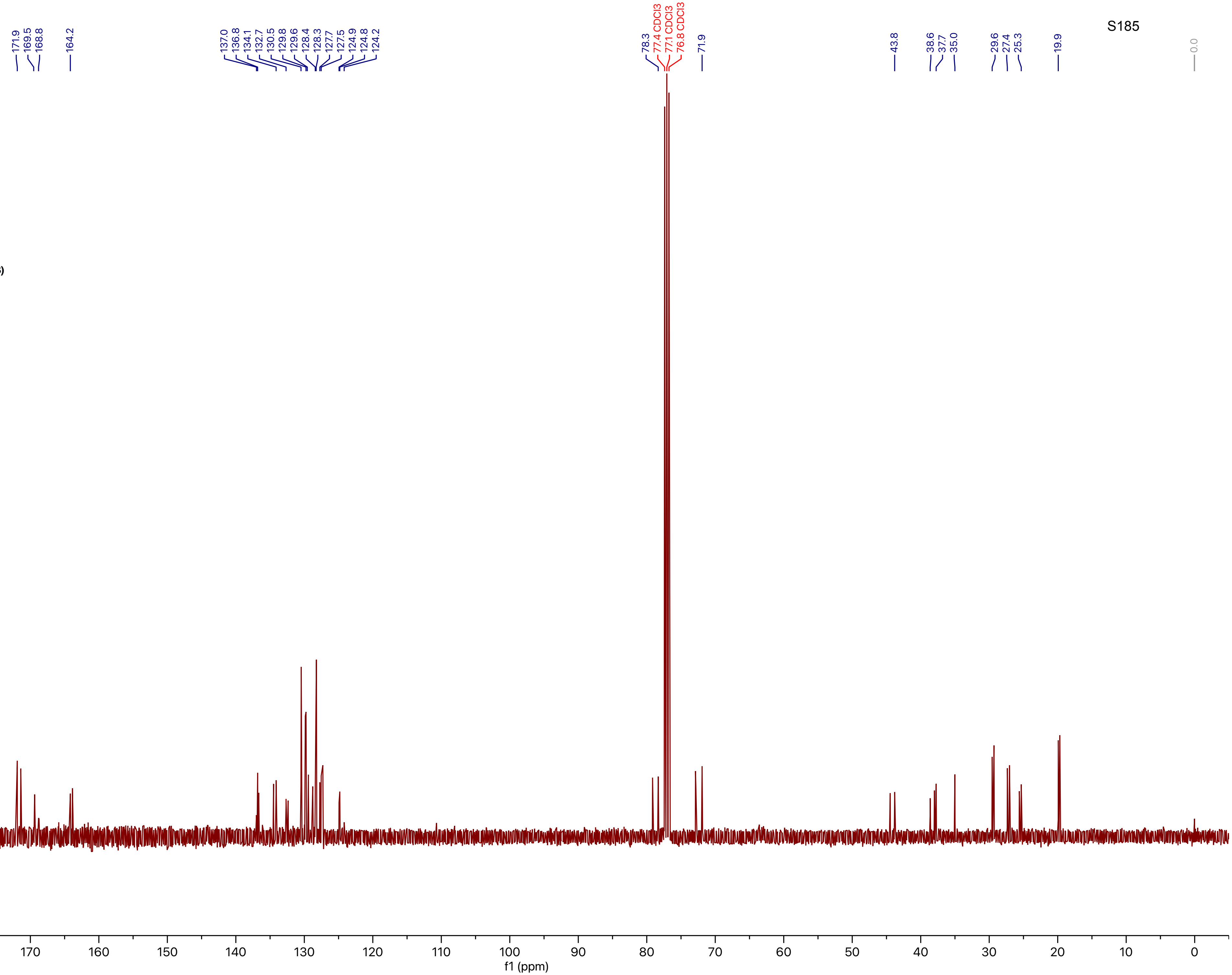

Supporting Information – Biological Testing of Compounds and Chemoproteomic Experiments

Lapinsky Lab Compound Designation = Compound Designation in the Manuscript

PJDU-112 = Compound **4a**
VPDU-229 = Compound **4c**
PJDU-220 = Compound **4g**
(±)-PJDU-216 = Compound (±)-**4i**
(±)-PJDU-204 = Compound (±)-**4n**

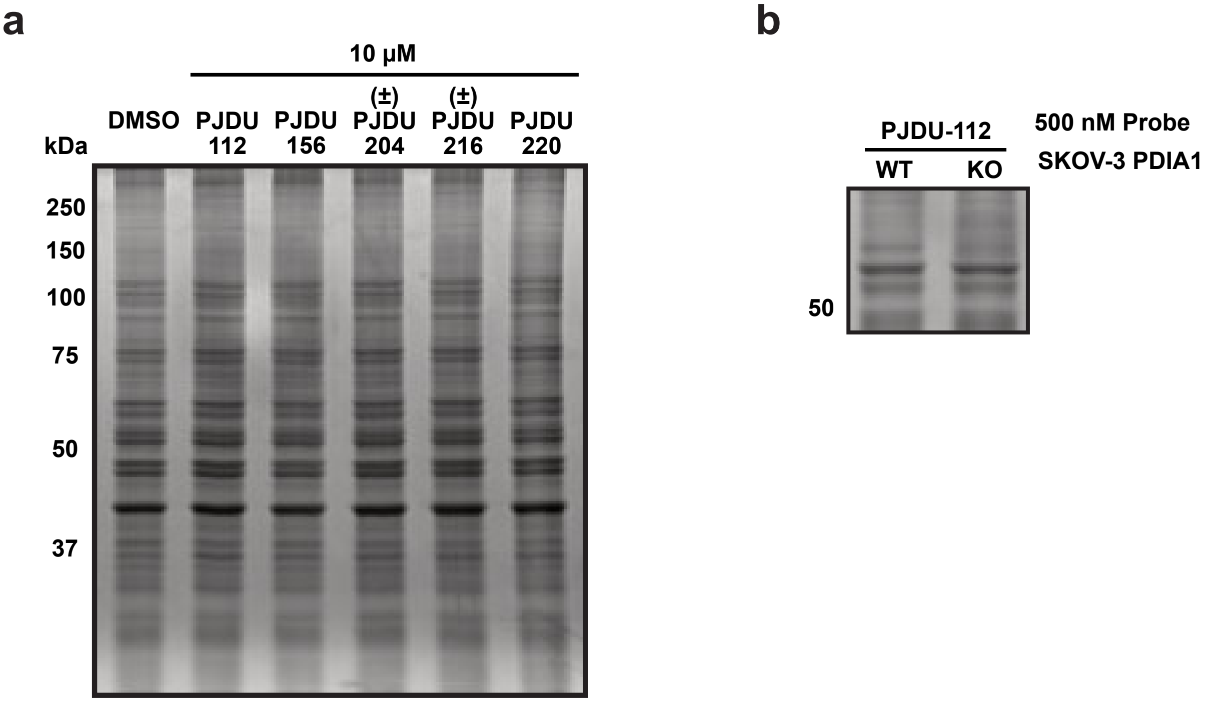

Figure S1a. Coomassie stained SDS-PAGE gels for protein loading controls corresponding to Figure 1b.

Figure S1b. Coomassie stained SDS-PAGE gels for protein loading controls corresponding to Figure 2c.

Experimental Materials and Methods

Biological Materials

Chemicals were obtained from Sigma-Aldrich and Fisher Scientific and used without further purification, unless otherwise noted. Phosphate buffered saline, PBS, was purchased from Fisher Scientific.

Biological Assays

Mammalian cell culture

MCF10CA1a and MCF10A cell lines were maintained under 37°C and 5% CO_2 in DMEM:F12 media (Invitrogen) supplemented with 5% horse serum (Invitrogen), 25 µg/mL of Amphotericin B, 10,000 units/mL of penicillin, and 10,000 µg/mL streptomycin (1%, Anti-Anti, Gibco), 10 µg/mL insulin (from bovine pancreas, Sigma Aldrich), 0.5 mg/mL hydrocortisone (Sigma Aldrich), 20 ng/mL epidermal growth factor (EGF, Sigma-Aldrich), 100 ng/mL cholera toxin (Sigma-Aldrich). SKOV-3 wild-type (American Type Culture Collection) and SKOV-3 PDIA1 CRISPR-Cas9 knockout (generated in house) lines were maintained under the same conditions in RPMI media (Corning) supplemented with 10% FBS and 1% Anti-Anti. HEK293T cells were grown under the same conditions in DMEM media supplemented with 10% FBS, and 1% Anti-Anti.

Following various treatments, cells were harvested and washed with PBS. To collect cell lysates, pellets were resuspended in PBS and lysed by ultrasonic tip sonicator (Cole Parmer). Soluble lysate fractions were obtained after separation by spinning at 45,000 rpm for 45 minutes at 4°C and pelleted insoluble fractions were discarded. To determine protein concentrations, DC Protein Assay kit (Bio-Rad) was performed.

MTT cell viability assay

MCF10CA1a cells were seeded at 1,000 cells/well to each well of a 96-well plate in 100 µL DMEM:F12 complete media. After 24 hours, 50 µM or 25 µM compound or DMSO in 100 µL fresh media was added with a no cell control with DMSO and media. Cells were grown for an additional 24 or 48 hours, after which, each well was aspirated. To each well was added 110 µL of MTT (0.45 mg/ml) in dye-free RPMI-1640 (Fisher Scientific) and cells were incubated for 4hr, after which 100 µL 10% SDS solution in 0.01% aqueous HCl was added to each well. The solution was covered and left at room temperature overnight and absorption was measured at 570 nm with SpectraMax M5 plate reader (Molecular Devices). Relative viability was determined by subtracting background and normalizing to DMSO treated control cells. Averaging three replicates gave reported relative cell viability values with error bars of stand error of the mean.

In situ labeling and fluorescence gel analysis

MCF10CA1a, and SKOV-3 (WT and PDIA1 KO) cells were grown to 100% confluency and treated with probe in the respective fresh media for one hour at 37°C and 5% CO_2. Cells were harvested and soluble fractions were collected, as described. Probe-labeled proteins were functionalized with a fluorophore (tetramethylrhodamine-azide; TAMRA-N_3, Click Chemistry Tools) by CuAAC[1] by addition of TAMRA-N_3 (25 µM, 100x stock in DMSO), TCEP (1 mM, 50x stock in water), TBTA (100 µM, 17x stock in *t*-BuOH:DMSO 4:1), and copper (II) sulfate (1 mM, 50x stock in water) at room temperature for one hour, vortexing every 15 minutes. Reactions were quenched by the addition of equivalent volume of reducing SDS-loading dye (2x concentrated). Proteome solutions were then separated on 10% SDS-PAGE gel at 100 volts for ~2.5 hours and rhodamine fluorescence was visualized on a ChemiDoc MP imaging system (BioRad, Hercules, CA). Gels were then coomassie stained for one hour and destained overnight, following a standard procedure, and visualized on the imager.

Transient transfection

HEK293T cells were grown on 10 cm plates to 60% confluency at 37 °C under 5% CO_2 in DMEM media supplemented with 10% fetal bovine serum (FBS, Atlanta Biologicals), and 1% Anti-Anti. Serum free (sf) DMEM media (1.5 mL) was combined with either lipofectamine 2000 (40 µL) or 8.8 µg pcDNA3.1(+)-myc mammalian expression vectors with either PDIA1 wild-type (WT), cysteine 53 alanine (C53A) or cysteine 397 alanine (C397A) mutants. Lipofectamine 2000 and pcDNA containing sf DMEM were mixed (3 mL total volume) and incubated for 7 minutes before the dropwise addition to each HEK293T plate. Plates were incubated for 24 hours at 37 °C under 5% CO_2. Cell lysates were prepared followed by conjugation to TAMRA-N_3 and fluorescent gel analysis, as previously described.

Western blot analysis

SDS-PAGE gels were transferred to nitrocellulose membranes by electrophoresis at 75 volts for 140 min. Membranes were washed with tris-buffered saline with 1% Tween 20 (TBS-T) three times for 5 minutes followed by blocking with 5% (w/v) non-fat dry milk in TBS-T at room temperature for 1 hour. Following additional washes with TBS-T, membranes were incubated with primary anti-myc tag rabbit antibody (Cell Signaling, 1:1000) overnight at 4°C. Following removal and an additional three washes with TBS-T, the membrane was incubated with secondary anti-rabbit-HRP conjugated antibody for 2 hours at room temperature. After final washes with TBS-T, membranes were developed with HRP-super signal chemiluminescence reagents and imaged on the same imager described above.

Tandem mass spectrometry preparation and analysis

MCF10CA1a cells were grown to 100% confluency in 20 cm tissue culture plates and treated with 500 nM PJDU-112 (Compound **4a**) or VPDU-229 (Compound **4c**). Cells were incubated at 37°C under at 5% CO_2 for 1 hour before being harvested. Soluble labeled lysates were prepared as described previously. Probe-labeled MCF10CA1a lysates in PBS (500 µL, 2 mg mL^{-1}) underwent CuAAC with biotin-azide (200 µM, 100x stock in DMSO), TCEP (1 mM, 50x stock in water), TBTA (100 µM, 17x stock in t-BuOH:DMSO 4:1), and copper (II) sulfate (1 mM, 50x stock in water). The reaction proceeded at room temperature for 1 hour with vortexing every 15 minutes. Samples were centrifuged (5 min, 4500 g, 4°C) pelleting the precipitated proteins. Protein pellets were resuspended in ice cold MeOH by tip sonication followed by spun and a second MeOH wash. The pellets were solubilized in 1.2% (w/v) SDS in PBS by sonication and heating (5 minutes at 80°C). Copper was then pelleted by spinning of the solubilized probe-labeled proteomes at 6500 g for 5 minutes. The supernatant was diluted with 5 mL PBS giving a final concentration of 0.2% SDS. 100 µL Streptavidin agarose resin (Thermo Scientific) was added to the samples, incubating overnight at 4°C. Solutions were resolublized at room temperature for 2.5 hours and the beads were washed with 0.2% SDS/PBS (5 mL) for 10 minutes followed by PBS (3 x 5 mL) and water (3 x 5 mL). Between washes, beads were pelleted by spinning in a centrifuge (1400 g for 3 minutes).

After the final wash, the beads were resuspended in 6 M Urea/PBS (500 µL) and 10 mM DTT (20x stock in water) and incubated at 65°C for 20 minutes. Iodoacetamide (10 mM, 50x stock in water) was then added and the samples reacted in the dark at room temperature for 30 minutes. Next, beads were diluted with 950 µL PBS, pelleted (1400 g for 3 min) and washed in 100 mM TEAB buffer. Subsequent resuspension in 200 µL of 2 M Urea in 100 mM triethyl ammonium bicarbonate (TEAB) buffer, 1 mM $CaCl_2$ (100x stock in water), and 2 µg trypsin in PBS (Promega,

Madison, WI) allowed for on-bead trypsin digestions to proceed overnight at 37°C with shaking. Supernatants were collected and placed in new tubes for reductive dimethylation (ReDiMe) labeling. PJDU-112/**4a**-enriched peptides were dimethylated with 'heavy' formaldehyde, (16 µL, 4% stock, $^{3}CD_2O$) and sodium cyanoborodeuteride (16 µL, 0.6M stock, $NaBD_3CN$), while, VPDU-229/**4c**-enriched peptides were dimethylated with 'light' formaldehyde (16 µL, 4%, CH_2O), and sodium cyanoborohydride, (16 µL, 0.6 M stock, $NaBH_3CN$) for 2 hours at room temperature. After the reactions were quenched with 3 µL 30% NH_4OH for 10 minutes at 0°C, PJDU-112/**4a**-enriched heavy dimethylated peptides and VPDU-229/**4c**-enriched light dimethylated peptides were combined (1:1 ratio). Formic acid (Thermo Fisher Scientific) was added to each sample for a final concentration of 5%. Samples were desalted on sepPak 1 cc C18 columns (50 mg, Waters) and dried overnight by speed vac. Peptide samples were resuspended in 95% water, 5% acetonitrile, and 0.1% formic acid, and the samples were stored at -20°C until mass spectrometry analysis.

LC/LC-MS/MS analysis was performed by Multidimensional Protein Identification Technology (MudPIT) on an LTQ Orbitrap Discovery mass spectrometer (Thermo Fisher) with separation coupled to an Agilent 1200 series HPLC (Agilent Technologies). ReDiMe labeled tryptic peptides were pressure loaded onto a 250 µm fused silica desalting column packed with 4 cm of Aqua C18 reversed phase resin (Phenomenex). Peptides were then eluted onto a biphasic 100 µm fused silica column with a 5 µm tip, packed with 10 cm of C18 and 4 cm of Partisphere strong cation exchange resin (SCX, Whatman). Gradients 5-100% Buffer B in Buffer A (Buffer A: 95% water, 5% acetonitrile, 0.1% formic acid; Buffer B: 20% water, 80% acetonitrile, 0.1% formic acid), eluted peptides from the desalting columns displacing them on to the biphasic column. Before transfer to the mass spectrometer, peptides were further separated by elutions from the SCX to the C18 resin using four salt pushes.[2] Flow rates of buffer through the column was set to ~0.25 µL/min with spray voltage of 2.75 kV. One full MS scan (400 – 1800 MW) was followed by 8 data dependent scans of the 8th most intense ions with dynamic exclusion enabled.

Two biological replicates of combined PJDU-112/**4a**-enriched heavy dimethylated peptides and VPDU-229/**4c**-enriched light dimethylated peptides from MCF10CA1a proteome were subjected to tandem mass spectrometric analysis described. The generated tandem MS data was searched using the SEQUEST algorithm against the human UniProt database. Peptides reported were required to be tryptic whilst also containing the differential modifications on lysine or the N-terminus of +36.07567 for 'heavy' dimethylation of PJDU-112/**4a** given peptides and +28.0313 for 'light' dimethylation of VPDU-229/**4c** given peptides. Each search included a static modification of +57.0215 on cysteine to account for alkylation of cysteine residues with iodoacetamide. For spectral count analysis, the SEQUEST output files were then filtered using DTASelect 2.0 to produce a curation of proteins identified with a false-discovery rate of less than 5%. The presented proteins were filtered to include only samples with an average of 10 spectral counts or greater in two PJDU-112/**4a** samples. For these proteins surpassing the provided criteria, the change in spectral counts between PJDU-112/**4a**-treated proteome and VPDU-229/**4c**-treated proteome was calculated. For quantitative analysis of heavy:light (H:L) ratios, fold enrichment of proteins labeled by PJDU-112/**4a** compared to VPDU-229/**4c**, was calculated through cimage quantification package.[2] Spectral counts and H:L ratios are presented in sorted mass spectrometry data in SI Table 1.

Recombinant Expression of PDIA1

pET23a C-terminal His_6 tag-fused PDIA1 WT was transformed in *Escherichia coli*. BL21 cells grown in Luria-Bertani broth with 100 µg/mL ampicillin at 37°C to A_{600} = 0.8. Expression was induced by 0.6 mM isopropyl β-D-1-thiogalactopyranoside (IPTG) and incubated overnight at

27°C. Cells were pelleted by centrifugation, lysed open by tip sonication in PBS, and soluble fraction collected. PDIA1 WT was isolated by Ni-NTA column and purified by PD10 size exclusion column.

Protein disulfide isomerase reductase assay

Preparation of the insulin turbidity[3] PDI activity assay began with 25 µL aliquots of 5 µM PDIA1 in PBS incubated with inhibitors, PJDU-112 (**4a**), KSC-34, or DMSO (0.5 µL of 50x) with final concentrations ranging from 1 µM to 25 µM for 30 minutes. Upon brief mixing, 10 µL of the solutions were added to 70 µL of freshly prepared assay buffer (100 mM K_2HPO_4, 2 mM EDTA, pH 7.0) in one well of a 96 well plate. 10 µL of bovine insulin solubilized in 0.1 N HCl (10x, 1.6 mM, Sigma-Aldrich) was also added to each reaction and the samples were shaken on the plate reader. Assay reactions were initiated by the addition of 10 µL dithiothreitol (DTT, 10x, 10 mM). Included was a control sample without PDIA1 containing DTT. Absorbances were read at 650 nm every 25 seconds for 30 minutes with plate shaking between each read. Assay final concentrations for each well included 0.5 µM PDIA1 WT, 0.16 mM bovine insulin, and 1 mM DTT. Background absorbances (from the sample without PDIA1) were subtracted for each sample at each time point read. Absorbance vs time plots were generated and the linear slopes from each plot were illustrating the decline in PDIA1 activity upon inhibitor treatments.

References - Biological Testing of Compounds and Chemoproteomic Experiments

1.) Rostovtsev, V. V.; Green, L. G.; Fokin, V. V.; Sharpless, K. B., A stepwise Huisgen cycloaddition process: Copper(I)-catalyzed regioselective "ligation" of azides and terminal alkynes. *Angew Chem Int Edit* **2002,** *41* (14), 2596-+.
2.) Weerapana, E.; Wang, C.; Simon, G. M.; Richter, F.; Khare, S.; Dillon, M. B. D.; Bachovchin, D. A.; Mowen, K.; Baker, D.; Cravatt, B. F., Quantitative reactivity profiling predicts functional cysteines in proteomes. *Nature* **2010,** *468* (7325), 790-U79.
3.) Holmgren, A., Thioredoxin Catalyzes the Reduction of Insulin Disulfides by Dithiothreitol and Dihydrolipoamide. *Journal of Biological Chemistry* **1979,** *254* (19), 9627-9632.

Protein Identification			Spectral Counts						Heavy : Light ReDiMe Ratios		
UniProt ID	Protein	Description	PJDU-112_1	PJDU-112_2	AVG PJDU-112	VPDU-229_1	VPDU-229_2	AVG VPDU-229	Ratio H:L_1	Ratio H:L_2	AVG Ratio H:L
P07237	P4HB	Protein disulfide-isomerase	473	483	478	3	0	1.5	20	20	20
P49327	FASN	Fatty acid synthase	122	110	116	47	33	40	3.5	3.49	3.5
Q16881	TXNRD1	Thioredoxin reductase 1, cytoplasmic	80	83	81.5	0	0	0	20	20	20
O75369	FLNB	Filamin-B	78	57	67.5	45	23	34	2	2.1	2.05
P60709	ACTB	Actin, cytoplasmic 1	64	53	58.5	26	23	24.5	2.29	2.52	2.41
P13639	EEF2	Elongation factor 2	58	52	55	33	21	27	1.88	2.56	2.22
P35579	MYH9	Myosin-9	69	29	49	26	10	18	1.81	2.01	1.91
P06733	ENO1	Alpha-enolase	33	65	49	18	21	19.5	1.53	3.08	2.31
P68371	TUBB4B	Tubulin beta-4B chain	51	46	48.5	32	20	26	1.69	1.94	1.82
P07437	TUBB	Tubulin beta chain	50	45	47.5	31	21	26	1.97	2.06	2.02
Q5TFE4	NT5DC1	5'-nucleotidase domain-containing protein 1	57	32	44.5	11	4	7.5	8.28	6.47	7.38
P78417	GSTO1	Glutathione S-transferase omega-1	44	43	43.5	0	0	0	20	20	20
P68366	TUBA4A	Tubulin alpha-4A chain	42	45	43.5	25	20	22.5	1.57	1.77	1.67
Q9H7Z7	PTGES2	Prostaglandin E synthase 2	38	43	40.5	0	0	0	20	20	20
Q71U36	TUBA1A	Tubulin alpha-1A chain	33	46	39.5	22	19	20.5	1.57	1.72	1.65
P08238	HSP90AB1	Heat shock protein HSP 90-beta	48	22	35	25	11	18	2.55	3.13	2.84
P16615	ATP2A2	arcoplasmic/endoplasmic reticulum calcium ATPase	37	30	33.5	6	9	7.5	5.62	4.77	5.2
P14618	PKM	Pyruvate kinase isozymes M1/M2	27	34	30.5	12	13	12.5	1.9	3.13	2.52
Q13509	TUBB3	Tubulin beta-3 chain	28	33	30.5	16	12	14	1.57	1.98	1.78
P07900	HSP90AA1	Heat shock protein HSP 90-alpha	47	12	29.5	25	4	14.5	2.46	2.9	2.68
P53396	ACLY	ATP-citrate synthase	26	27	26.5	6	10	8	2.58	2.9	2.74
P30519	HMOX2	Heme oxygenase 2	29	22	25.5	0	0	0	20	16.07	18.04
P68104	EEF1A1	Elongation factor 1-alpha 1	28	15	21.5	17	0	8.5	1.88	2.55	2.22
P07355	ANXA2	Annexin A2	19	20	19.5	12	6	9	1.94	3.88	2.91
P31947	SFN	14-3-3 protein sigma	27	9	18	3	2	2.5	9.15	6.06	7.61
P10809	HSPD1	60 kDa heat shock protein, mitochondrial	15	21	18	10	8	9	1.61	3.55	2.58
P78371	CCT2	T-complex protein 1 subunit beta	9	25	17	6	5	5.5	2.49	4.11	3.3
P11142	HSPA8	Heat shock cognate 71 kDa protein	18	16	17	9	8	8.5	1.93	3.28	2.61
P04075	ALDOA	Fructose-bisphosphate aldolase A	18	16	17	13	8	10.5	1.41	2.7	2.06
Q9BVL4	SELO	Selenoprotein O	17	16	16.5	0	0	0	20	20	20
O00410	IPO5	Importin-5	20	12	16	2	0	1	7	12.58	9.79
Q9BUF5	TUBB6	Tubulin beta-6 chain	15	17	16	5	8	6.5	1.68	1.94	1.81
P30101	PDIA3	Protein disulfide-isomerase A3	18	13	15.5	2	0	1	16.33	20	18.17
P11021	HSPA5	78 kDa glucose-regulated protein	13	16	14.5	2	5	3.5	1.67	4.41	3.04
P29401	TKT	Transketolase	13	16	14.5	5	4	4.5	1.84	2.87	2.36
P37802	TAGLN2	Transgelin-2	11	16	13.5	0	4	2	1.82	2.98	2.4
P19338	NCL	Nucleolin	10	17	13.5	4	3	3.5	1.23	3.91	2.57
Q92696	RABGGTA	Geranylgeranyl transferase type-2 subunit alpha	19	6	12.5	0	0	0	20	20	20
P78527	PRKDC	DNA-dependent protein kinase catalytic subunit	14	11	12.5	0	0	0	5.11	5.27	5.19
P55072	VCP	Transitional endoplasmic reticulum ATPase	10	15	12.5	4	0	2	2.09	2.48	2.29
O75153	KIAA0664	Clustered mitochondria protein homolog	14	10	12	0	0	0	20	20	20
P34932	HSPA4	Heat shock 70 kDa protein 4	12	12	12	0	0	0	1.73	2.21	1.97
Q15084	PDIA6	Protein disulfide-isomerase A6	10	14	12	0	0	0	20	20	20
Q01581	HMGCS1	Hydroxymethylglutaryl-CoA synthase, cytoplasmic	14	10	12	6	2	4	2.33	4.01	3.17
P12814	ACTN1	Alpha-actinin-1	8	15	11.5	3	0	1.5	1.88	3.24	2.56
P04083	ANXA1	Annexin A1	14	9	11.5	10	3	6.5	1.76	3.44	2.6
P49356	FNTB	Protein farnesyltransferase subunit beta	16	6	11	0	0	0	20	20	20
P14625	HSP90B1	Endoplasmin	10	12	11	4	4	4	2.53	4.53	3.53
P22314	UBA1	Ubiquitin-like modifier-activating enzyme 1	11	11	11	4	7	5.5	2.32	2.87	2.6
Q92598	HSPH1	Heat shock protein 105 kDa	16	5	10.5	5	0	2.5	2.58	2.86	2.72
Q16658	FSCN1	Fascin	7	14	10.5	7	0	3.5	1.68	3.13	2.41
P50990	CCT8	T-complex protein 1 subunit theta	7	13	10	2	0	1	1.51	3.76	2.64
P36969	GPX4	holipid hydroperoxide glutathione peroxidase, mitoch	16	4	10	0	0	0	20	20	20
P07195	LDHB	L-lactate dehydrogenase B chain	9	11	10	2	2	2	2.82	3.74	3.28
P29034	S100A2	Protein S100-A2	16	4	10	4	0	2	2.44	3.78	3.11
Q01813	PFKP	6-phosphofructokinase type C	12	8	10	5	0	2.5	2.54	2.44	2.49
P11216	PYGB	Glycogen phosphorylase, brain form	11	9	10	5	4	4.5	2.08	3.35	2.72

Table S1. Mass spectrometry data for 500 nM PJDU-112 (Compound **4a**) and VPDU-229 (Compound **4c**) treated MCF10CA1a cells. Proteins are sorted by highest average spectral counts in the PJDU-112 (Compound **4a**) treated samples.

Chemical Structures of All FFSMPs Produced During This Work

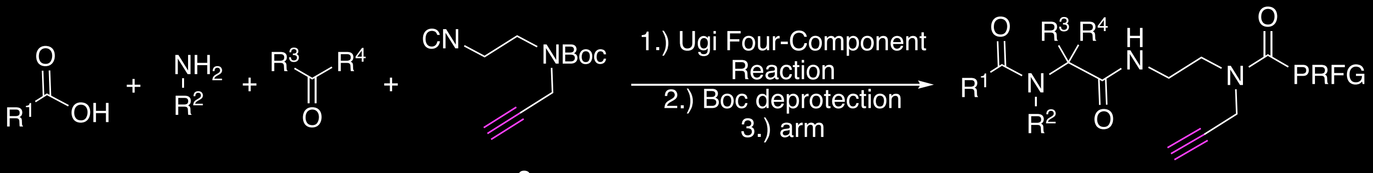

3

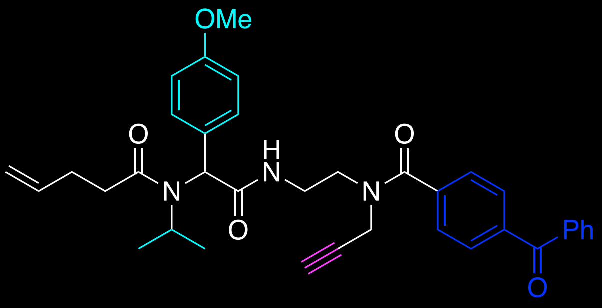

(±)-PJDU-075

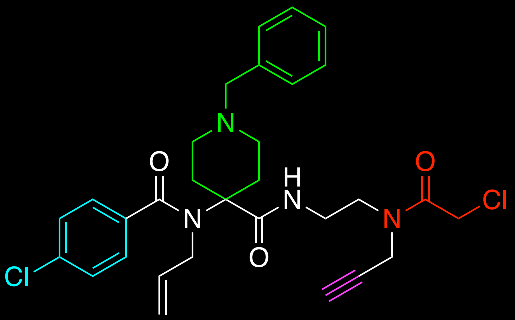

4a (PJDU-112)

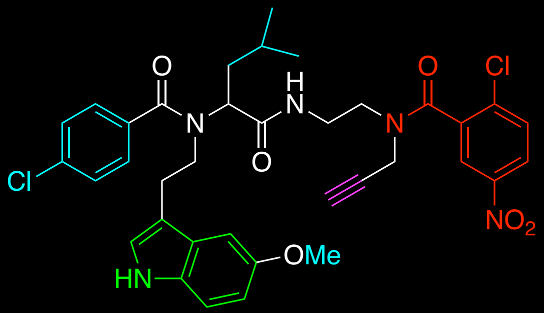

(±)-VPDU-242

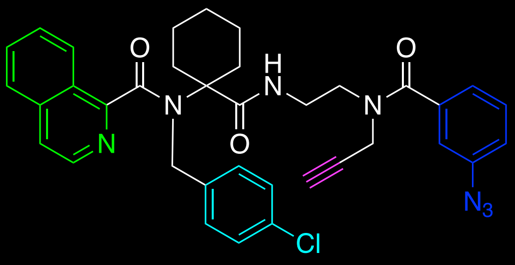

VPDU-253

Figure S2. *Ugi Four-Component Reaction-Derived FFSMPs Stemming From Isocyanide **3**.*

- terminal alkyne in magenta for click chemistry-based proteomics
- electrophilic protein-reactive functional group (PRFG) for affinity labeling in red
- photoreactive protein-reactive functional group (PRFG) for photoaffinity labeling in blue
- privileged structure to sample biologically relevant chemical space in green
- Topliss moiety for medicinal chemistry optimization in cyan

- **note:** N-benzyl piperidines are a privileged structure that also contain a benzyl substituent for Topliss opimization

Chemical Structures of All FFSMPs Produced During This Work

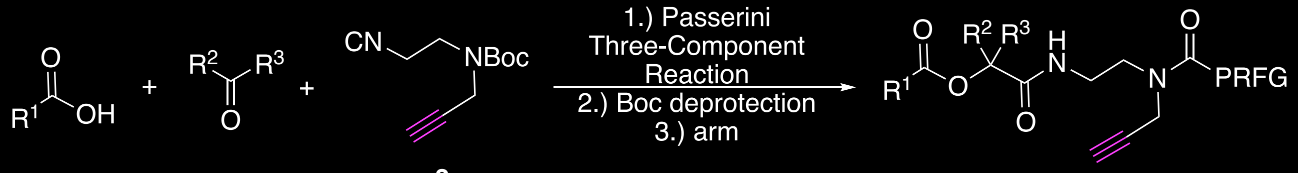

3

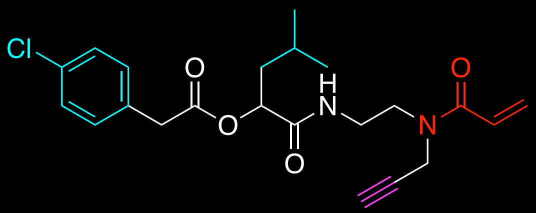

(±)-**4b ((±)-PJDU-083)**

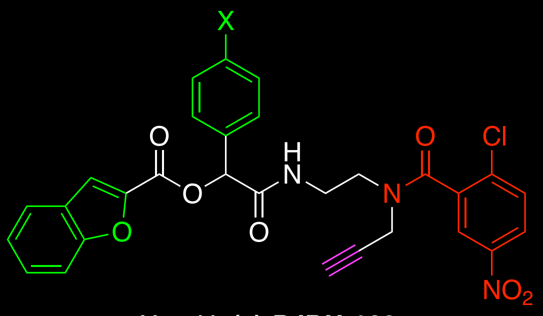

X = -H; **(±)-PJDU-162**
X = -Cl; **(±)-PJDU-161**

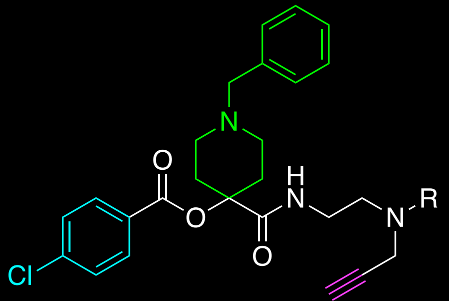

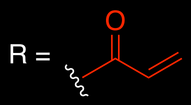

4c (VPDU-229)

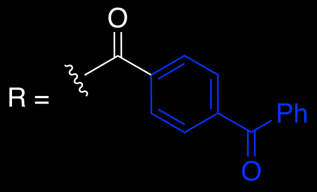

4d (VPDU-256)

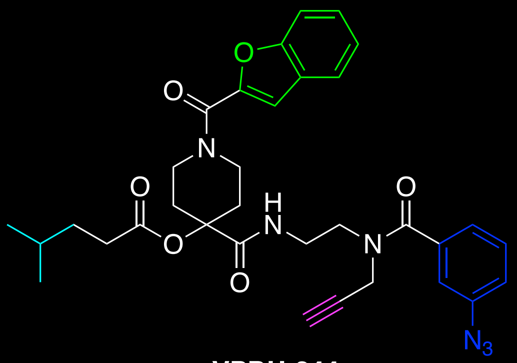

VPDU-244

Figure S3. *Passerini Three-Component Reaction-Derived FFSMPs Stemming From Isocyanide* ***3****.*

- terminal alkyne in magenta for click chemistry-based proteomics
- electrophilic protein-reactive functional group (PRFG) for affinity labeling in red
- photoreactive protein-reactive functional group (PRFG) for photoaffinity labeling in blue
- privileged structure to sample biologically relevant chemical space in green
- Topliss moiety for medicinal chemistry optimization in cyan

- **note:** N-benzyl piperidines are a privileged structure that also contain a benzyl substituent for Topliss opimization

Chemical Structures of All FFSMPs Produced During This Work

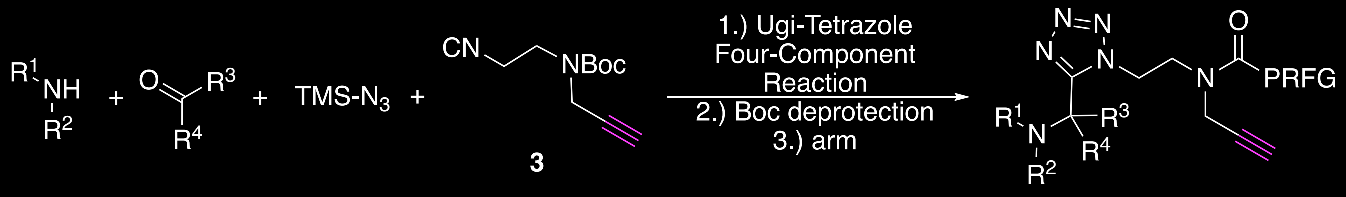

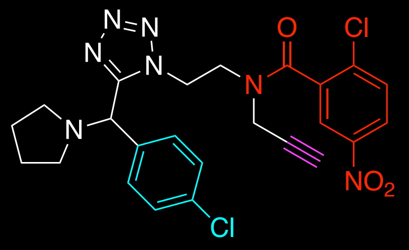

(±)-4e ((±)-PJDU-095)

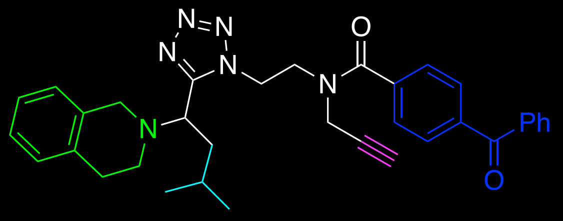

(±)-VPDU-233

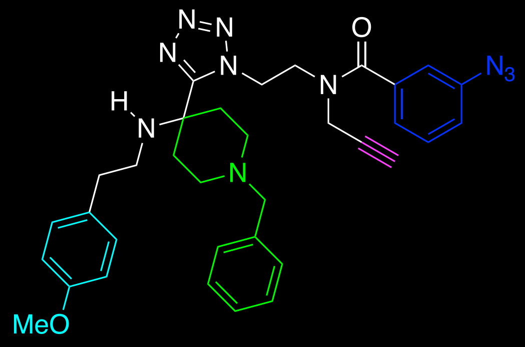

PJDU-110

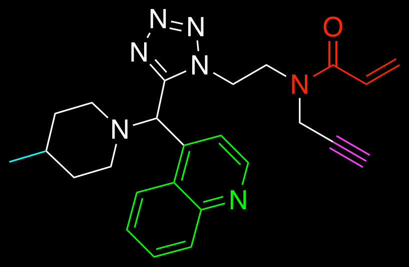

(±)-VPDU-247

Figure S4. *Ugi-Tetrazole Four-Component Reaction-Derived FFSMPs Stemming From Isocyanide* ***3****.*

- terminal alkyne in magenta for click chemistry-based proteomics
- electrophilic protein-reactive functional group (PRFG) for affinity labeling in red
- photoreactive protein-reactive functional group (PRFG) for photoaffinity labeling in blue
- privileged structure to sample biologically relevant chemical space in green
- Topliss moiety for medicinal chemistry optimization in cyan

- **note:** N-benzyl piperidines are a privileged structure that also contain a benzyl substituent for Topliss opimization

Chemical Structures of All FFSMPs Produced During This Work

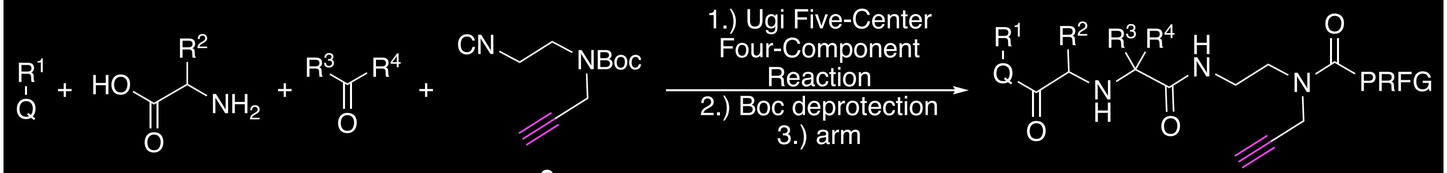

3

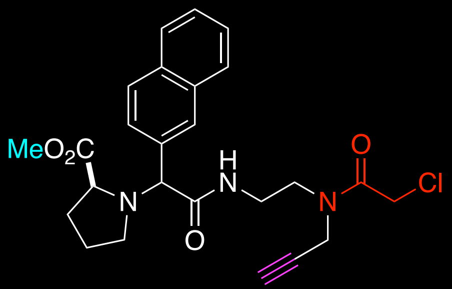

(±)-4f ((±)-PJDU-090)

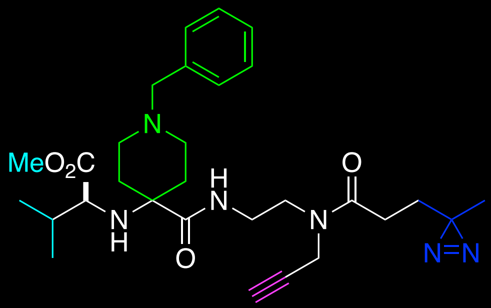

PJDU-150

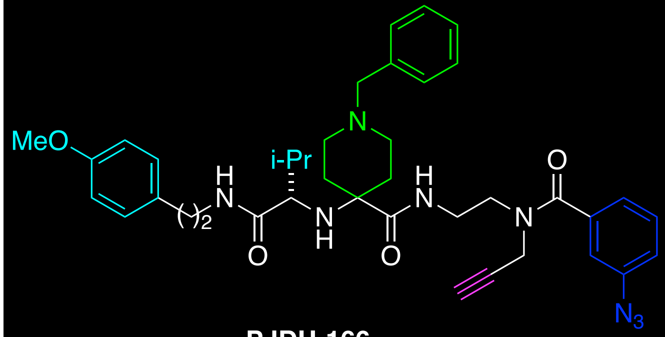

PJDU-166

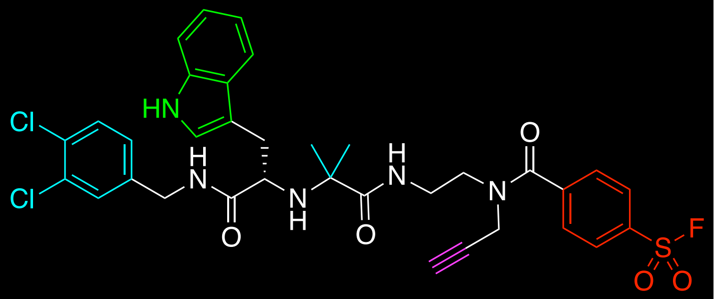

4g (PJDU-220)

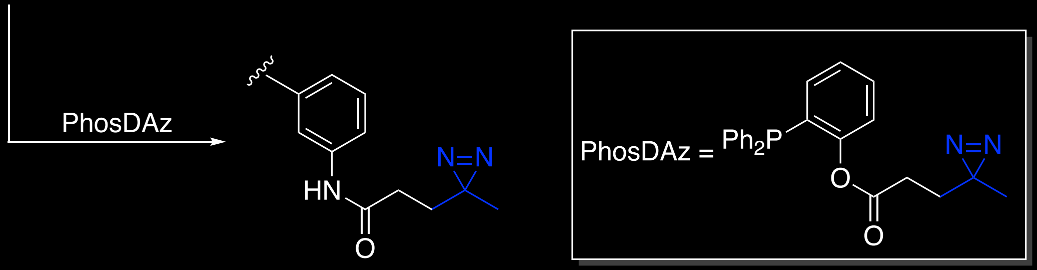

PJDU-167

Figure S5. *Ugi Five-Center Four-Component Reaction-Derived FFSMPs Stemming From Isocyanide **3**.*

- terminal alkyne in magenta for click chemistry-based proteomics
- electrophilic protein-reactive functional group (PRFG) for affinity labeling in red
- photoreactive protein-reactive functional group (PRFG) for photoaffinity labeling in blue
- privileged structure to sample biologically relevant chemical space in green
- Topliss moiety for medicinal chemistry optimization in cyan

- **note:** N-benzyl piperidines are a privileged structure that also contain a benzyl substituent for Topliss opimization

Chemical Structures of All FFSMPs Produced During This Work

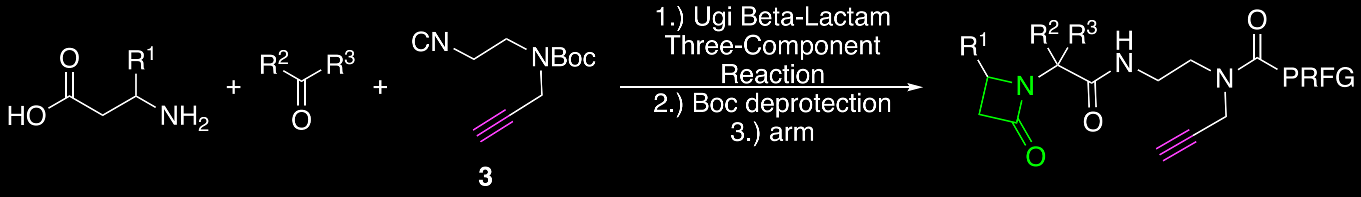

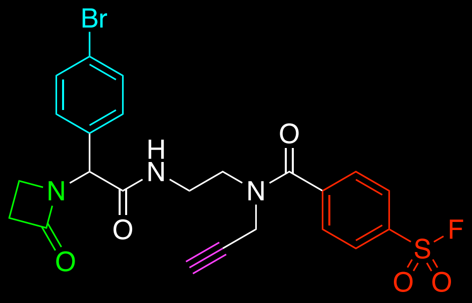

(±)-4h ((±)-PJDU-092)

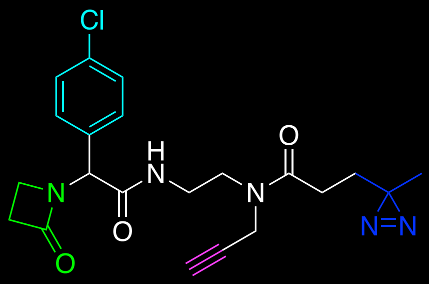

(±)-PJDU-141

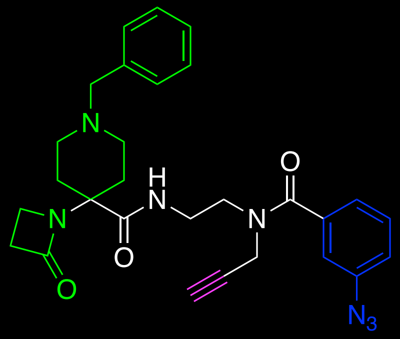

PJDU-134

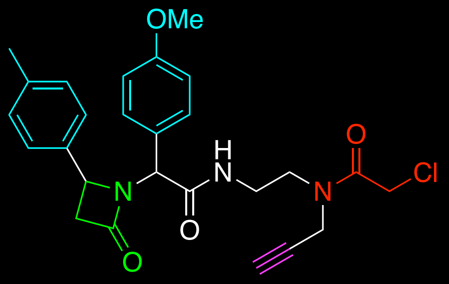

(±)-4i ((±)-PJDU-216)

Figure S6. *Ugi Beta-Lactam Three-Component Reaction-Derived FFSMPs Stemming From Isocyanide* ***3****.*

- terminal alkyne in magenta for click chemistry-based proteomics
- electrophilic protein-reactive functional group (PRFG) for affinity labeling in red
- photoreactive protein-reactive functional group (PRFG) for photoaffinity labeling in blue
- privileged structure to sample biologically relevant chemical space in green
- Topliss moiety for medicinal chemistry optimization in cyan

- **note:** N-benzyl piperidines are a privileged structure that also contain a benzyl substituent for Topliss opimization

- **note:** beta-lactams are a privileged structure that are also an electrophilic protein-reactive functional group (PRFG) for affinity labeling

Chemical Structures of All FFSMPs Produced During This Work

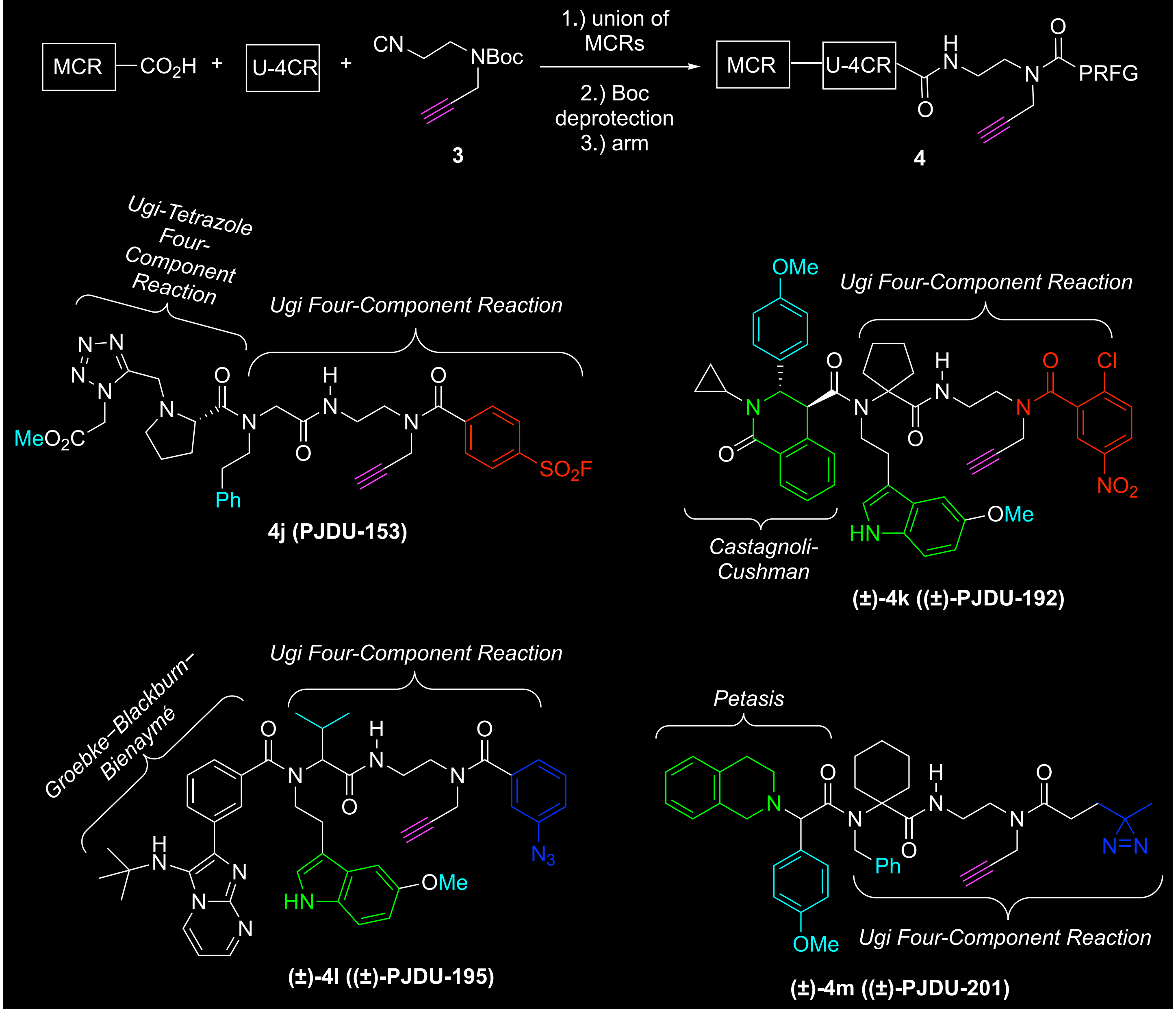

Figure S7. *FFSMPs Stemming From Isocyanide **3** Via a Union of Multi-Component Reaction (MCR) Approach.*

- terminal alkyne in magenta for click chemistry-based proteomics
- electrophilic protein-reactive functional group (PRFG) for affinity labeling in red
- photoreactive protein-reactive functional group (PRFG) for photoaffinity labeling in blue
- privileged structure to sample biologically relevant chemical space in green
- Topliss moiety for medicinal chemistry optimization in cyan

Chemical Structures of All FFSMPs Produced During This Work

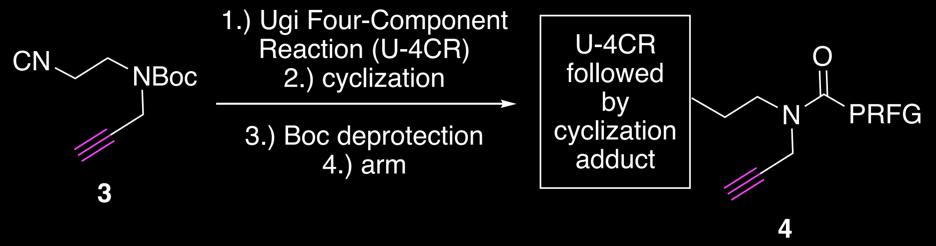

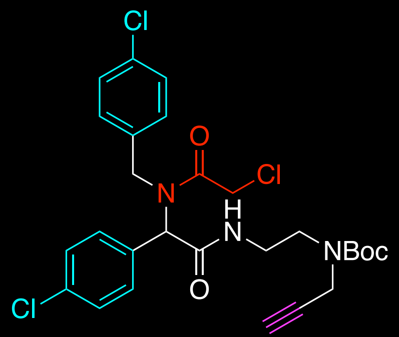

(±)-10 (±)-PJDU-200)

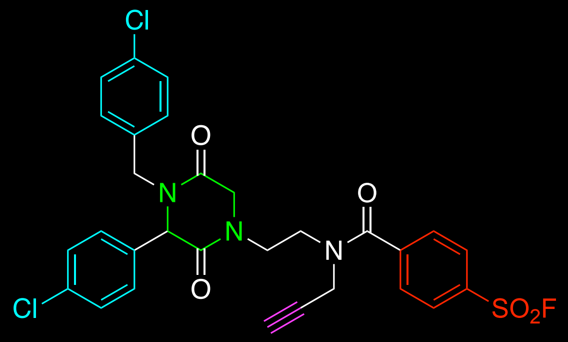

(±)-4n ((±)-PJDU-204)

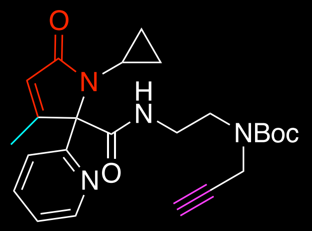

(±)-13 ((±)-PJDU-210)

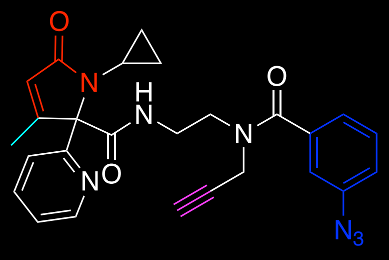

(±)-4o ((±)-PJDU-214)

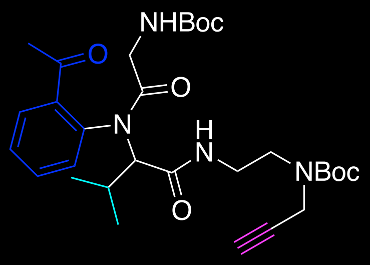

(±)-14 ((±)-PJDU-218)

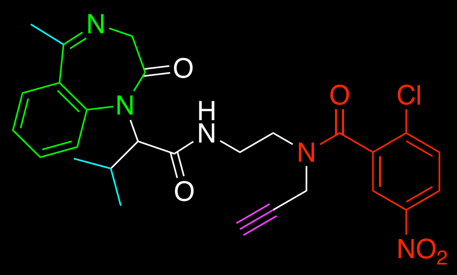

(±)-4p ((±)-PJDU-223)

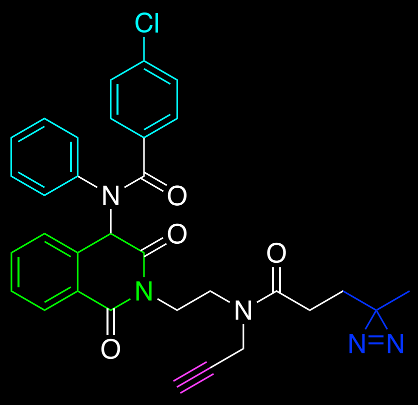

(±)-4q ((±)-PJDU-212)

Figure S8. *FFSMPs Stemming From Isocyanide* ***3*** *Via a Ugi Four-Component Reaction (U—4CR)-Intramolecular Cyclization Approach.*

- terminal alkyne in magenta for click chemistry-based proteomics
- electrophilic protein-reactive functional group (PRFG) for affinity labeling in red
- photoreactive protein-reactive functional group (PRFG) for photoaffinity labeling in blue
- privileged structure to sample biologically relevant chemical space in green
- Topliss moiety for medicinal chemistry optimization in cyan
